# The Small RNA Universe of *Capitella teleta*

**DOI:** 10.3389/fmolb.2022.802814

**Published:** 2022-02-25

**Authors:** Sweta Khanal, Beatriz Schueng Zancanela, Jacob Oche Peter, Alex Sutton Flynt

**Affiliations:** Cellular and Molecular Biology, University of Southern Mississippi, Hattiesburg, MS, United States

**Keywords:** miRNA, piRNA, RNAi, annelid, development

## Abstract

RNAi is an evolutionarily fluid mechanism with dramatically different activities across animal phyla. One major group where there has been little investigation is annelid worms. Here, the small RNAs of the polychaete developmental model *Capitella teleta* are profiled across development. As is seen with nearly all animals, nearly 200 microRNAs were found with 58 high-confidence novel species. Greater miRNA diversity was associated with later stages consistent with differentiation of tissues. Outside miRNA, a distinct composition of other small RNA pathways was found. Unlike many invertebrates, an endogenous siRNA pathway was not observed, indicating pathway loss relative to basal planarians. No processively generated siRNA-class RNAs could be found arising from dsRNA precursors. This has a significant impact on RNAi technology development for this group of animals. Unlike the apparent absence of siRNAs, a significant population of piRNAs was observed. For many piRNAs, phasing and ping-pong biogenesis pathways were identified. Interestingly, piRNAs were found to be highly expressed during early development, suggesting a potential role in regulation in metamorphosis. Critically, the configuration of RNAi factors in *C. teleta* is found in other annelids and mollusks, suggesting that similar biology is likely to be present in the wider clade. This study is the first in providing comprehensive analysis of small RNAs in annelids.

## Introduction

RNA interference (RNAi) is a widely applied genetic technology based on fundamental gene regulatory mechanisms, where small RNAs induce complementary transcript degradation or destruction. There are also numerous examples of small RNAs directing epigenetic alterations. However, the behavior of RNAi pathways varies from species to species, making application of a single paradigm inappropriate (Flynt, 2021). Among invertebrate animals, RNAi pathways appear to be mainly plastic, indicating that at a minimum an order—if not family-level investigation of biogenesis mechanisms is necessary for effective development of the gene-silencing technology.

Animal small RNAs belong to three classes: microRNAs (miRNAs), PIWI-interacting RNAs (piRNAs), and small-interfering RNAs (siRNAs) (Kim et al., 2009). Among the three major classes of RNAi, miRNAs are the most conserved with sequences shared in nearly all animals (Wienholds and Plasterk, 2005). In comparison, both endogenously expressed siRNAs (endo-siRNAs) and piRNAs show almost no conservation even at the species level, presumably due to their role in silencing invasive nucleic acids (Wynant et al., 2017). In addition to these conservation patterns, each class is defined by loading into a distinct class of Argonaute/PIWI (Ago/PIWI) proteins. Work from arthropods, where each class is well-represented, provides definitions where Ago1 (miAgo), Ago2 (siAgo), and PIWI proteins load miRNAs, siRNAs, and piRNAs, respectively. Arthropod PIWI proteins include PIWI, Aubergine (Aub), and Ago3. In contrast, even though vertebrate genomes encode multiple Agos, usually four, they do not have dedicated siAgos (Müller et al., 2020). Thus, RNAi/siRNA technology in vertebrates is based on miRNA mimicry and is distinct from siRNAs used for RNAi in flies and nematodes (Doench et al., 2003). Vertebrates do possess numerous PIWI proteins as arthropods. This highlights the lability of distinct siRNA pathways. Indeed, analysis of selection in different Drosophilid pathway components shows miAgos are the most stable, followed by PIWI proteins, with siAgos being the most rapidly evolving (Palmer et al., 2018).

In addition to association with distinct effectors, RNAi pathways are also defined by biogenesis. miRNAs are derived from short hairpins that are initially cropped from heterogenous transcripts by the RNase III enzyme Drosha, followed by export and final maturation by a second RNase III enzyme Dicer (Bartel, 2018). miRNA hairpin precursors have features that include asymmetric bulges with larger terminal loops of ∼10 nt depending on the species. Pre-miRNA stem sequences are deeply conserved, while the terminal loop sequence is less so (Berezikov and Plasterk, 2005). siRNAs are also produced by Dicer and are typically 20–24 nt but are instead derived from long double-stranded RNA (dsRNA) molecules (Lee et al., 2004). Various sources yield siRNAs that include viruses along with may endogenous species such as hairpin RNAs, *cis*-NATs, and transposable elements (Okamura and Lai, 2008).

piRNA biogenesis occurs from two distinct processes: phasing and ping-pong amplification. Both phasing-dependent piRNAs (phasi-piRNAs) and ping-pong piRNAs are created independent of the RNase III activity, with processing driven by the “slicer” RNase activity intrinsic to PIWIs (Zamore, 2010). A consequence of this is a different size range (25–32 nt) compared to miRNAs and siRNAs (20–24 nt). phasi-piRNAs are produced from single-stranded RNAs (ssRNAs) that are initially cleaved by a PIWI protein, which leads to further processing by the endonuclease Zucchini (Ross et al., 2014; Weick and Miska, 2014). Phasi-piRNAs are defined by 1U bias and close proximity (1–3 nt) between the 3′ end of an upstream piRNA and the 5′ end of a downstream piRNA. Ping-pong piRNAs are created in an amplifying loop where partner PIWI proteins, Aub and Ago3 in *Drosophila*, slice transcripts that subsequently load into partners becoming new piRNAs (Brennecke et al., 2007). As ping-pong piRNAs are generated by slicing of complementary transcripts, they can be identified by 10 nt overhangs between piRNAs. Phasi-piRNA and ping-pong piRNA biogenesis collaborate to generate piRNAs to suppress the expression of unlicensed transcripts.

The paradigm for piRNA function is suppression of transposable elements (TEs) both in arthropods and vertebrates. In these species, large piRNA clusters serve as repositories of forbidden elements (Yamanaka et al., 2014). piRNA clusters are found both in uni-strand and dual-strand arrangements that yield phasi-piRNAs, which subsequently participate in the ping-pong cycle (Yamanaka et al., 2014). There are also genic piRNAs produced from UTRs by phasing biogenesis, which are likewise found in vertebrates and invertebrates (Robine et al., 2009). Initially, piRNAs were viewed as exclusive to germlines; however, for many invertebrates, piRNAs are also present in soma where they not only appear to suppress TEs but also participate in gene regulatory networks (Lewis et al., 2018). In contrast, *C. elegans* piRNAs have a completely distinct biogenesis, with each piRNA being produced from short autonomous transcriptional units, defined by a specific motif ∼ 50 nt upstream of the piRNA transcriptional start site (Billi et al., 2013).

RNAi pathways are well-documented in model organisms such as *Drosophila melanogaster* and *Caenorhabditis elegans*, as well as in vertebrates like mice and humans. In comparison, there has been substantially less investigation of RNAi in spiralians, one of three animal superphyla that include flatworms, annelids, and mollusks, which branched ∼700–850 million years from Ecdysozoa. Research has mostly been in planarians which found all three small RNA classes with a pronounced expansion of piRNAs (Cao et al., 2020). Other studies have investigated small RNAs in mollusks that likewise noted substantial expansion of piRNAs (Jehn et al., 2018). These leave one major spiralian group, annelids, where there has been no comprehensive investigation of small RNA classes. Here, we describe small RNA expression and biogenesis in the annelid developmental model *Capitella teleta*. From these efforts, we find numerous novel miRNAs and as seen with other spiralians, a substantial collection of somatic piRNAs. Interestingly, we do not find endogenous siRNAs, suggesting that following the split from planarians, lophotrochozoans (annelids and mollusks) lost a distinct siRNA pathway. This greatly impacts RNAi approaches in these animals, informing gene-silencing approaches that would be beneficial for manipulating a variety of economically significant organisms.

## Materials and Methods

### *Capitella* Acquisition and Culture

*Capitella teleta* (juveniles and adults) were obtained from Dr. Elaine Seaver’s lab at the Whitney Laboratory for Marine Bioscience at the University of Florida. They were grown in organically enriched mud from Biloxi Bay, Mississippi. Around 20 adult worms (∼10 of each male and females) were placed in a 500 ml container with a tablespoon full of mud and 200 ml sea water. Adults were fed once a week, and juveniles were fed once every 2 weeks, by adding a scoop of new mud to the containers. Worms were kept in a growth chamber maintained at 20°C. Adult containers were routinely checked for the presence of brood tubes and embryos. Once found, about 25 larvae were transferred into new containers and placed in a growth chamber and fed weekly with new mud.

### RNA Extraction From Developmental Stages of *Capitella*

Early embryos were acquired by separating sexually mature males and females for 5–7 days and then keeping them together for 10–12 h. Containers were then checked for the brood tubes containing new laid eggs. Early embryos (2-cell stage) from two brood tubes were collected for RNA extraction using 100 µl of TRI Reagent LS. Similarly, late-stage embryo, larva (swimmers), three adult male anteriors (containing sperm sac), and three adult female anteriors were collected in 100 µl of TRI Reagent LS. After grinding, 100 µl of deionized water and 800 µl of TRIzol LS were added, and the extraction was completed, following manufacturer protocols. Addition of deionized water was necessary to mitigate excess salts for efficient purification of RNAs. For posterior RNA extractions, additional purification using the mirVana™ miRNA Isolation Kit was performed to address contaminants, likely from fecal matter that interfered with nucleic acid–manipulating enzymes and possibly gel electrophoresis. RNAs extracted from the seven different tissues were then subjected to small RNA-sequencing utilizing Illumina NextSeq500 after library construction with the Illumina TruSeq small RNA cloning kit. Two rounds of library construction and sequencing were used for adult posterior libraries. The quality of the datasets was validated using the miRTrace tool (Supplementary File S10) (Kang et al., 2018). All libraries were positively identified as lophotrochozoan with very little contamination by rRNA. For both embryo stages, where piRNAs are dominant, fewer miRNA-sized reads were recovered as a percent of the library. All clipped, unfiltered data are available through the NCBI SRA database under the bioproject #PRJNA777269. The *S. mediterranea* dataset was acquired from NCBI SRA under the bioproject # PRJNA117181. Planarian small RNA libraries were generated using protocols similar to what we used for *C. teleta* (TRIzol extraction (Invitrogen) and T4 RNA ligase 2 (Rnl2(1–249)K227Q) for library preparation).

### Small RNA Analysis Pipeline

Genome sequence and genome annotation files for *C. teleta* were acquired from Ensembl Metazoa (Capitella_teleta_v1.0). Small RNA analysis was carried out using pipelines diagrammed in supplement (Supplementary Figure S9). Small RNA loci were identified using bowtie alignments converted to the bedgraph format and filtered based on coverage using awk. Similarly, size distribution was determined using awk to quantify the length of reads extracted from alignments to single loci.

miRNAs in *C. teleta* were investigated using miRDeep2 and standard parameters (Friedländer et al., 2008). Annotations from MirGeneDB were used to guide annotations (Fromm et al., 2021). Novel miRNAs were assessed by manual curation. miRDeep2 calls were evaluated for presence of RNase III cleavage (2 nt 3′ overhangs) between mature and star strands. Potential miRNAs were also assessed for 5′ processing precision where a >90% of reads aligning to a hairpin arm share a 5′ base. Loci that met these criteria were classified as confident. If an miRNA did not meet both standards or if star reads did not exceed a coverage of eight, the miRNA was considered candidate. If the potential miRNA failed both criteria, it was labeled a false positive and not reported. miRDeep2 annotation provided outputs for known, confident, and candidate miRNAs (Supplementary Files S3, S5, S7, S8)

For heatmaps, libraries were normalized for the number of reads mapped to the genome. A python-based algorithm was used to find the overlapping read pairs that represent Dicer and ping-pong signatures (Antoniewski and Werner, 2014). Small RNAs of 15–31 nt were used to find targets (same length) having a 10 nt overlap, indicating the ping-pong signature, while the overlap of 2 nt less than a query indicates Dicer signatures. piRNA loci were analyzed for phasing using piPipes (Han et al., 2015). Graphics and visualizations were obtained using ggplot2, gplot, sushi plot, and pheatmap.

## Results

### *C. teleta* Global Small RNA Populations

**FIGURE 1:**
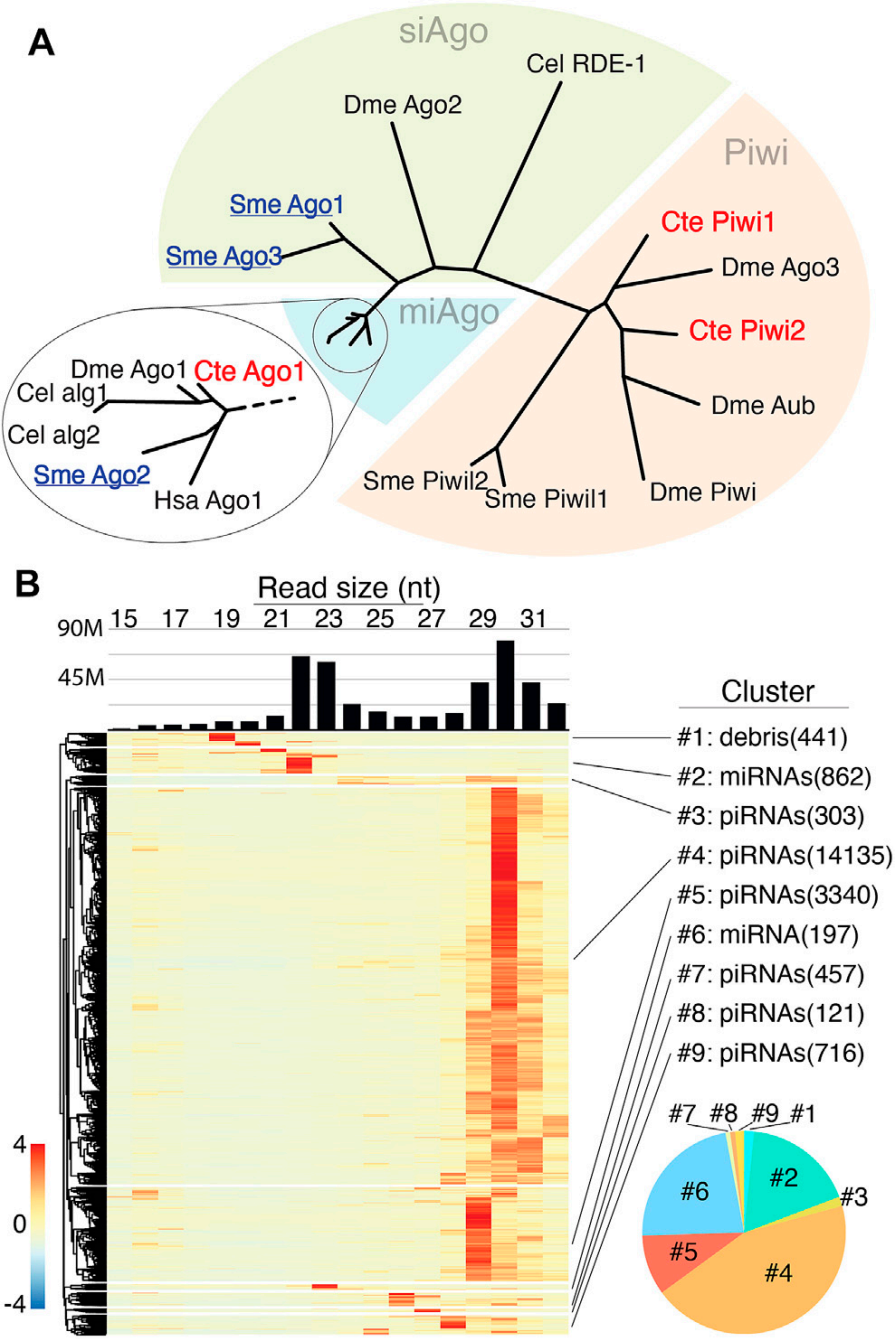
Small RNA populations of *C. teleta* **(A)** Phylogenetic comparison of Argonaute protein from various organisms (*C. elegans, S. mediterranea, D. melanogaster*, and *H. sapiens)* with *C. teleta. C. teleta* Argonaute protein clusters together with miAgos. **(B)** Size distribution of small RNA from >20000 small RNA producing loci. The bar graph shows bulk reads across all libraries. The heatmap is row normalized with small RNA expressions per locus. Loci were clustered into groups (#1–9), depending on the length of the RNA expressed at loci. Clusters of miRNAs contain 21–23 nt reads while piRNA clusters contain 28–31 nt reads. Cluster #1 was characterized as debris (degradation products) based on the small length of the RNAs; however, a single annotated miRNA species (*bantam*) was found within the cluster. The pie chart (bottom right) shows the relative abundances of piRNAs (warm colors) and miRNAs (cool colors) based per cluster.

We began assessment of *C. teleta* RNAi pathways by examining this annelid’s Ago proteins (Figure 1A). Surprisingly, only three Ago/PIWI proteins are encoded in the genome. To predict likely functions, a homology to select Ago/PIWI proteins from *Drosophila melanogaster*, *Caenorhabditis elegans*, *Homo sapiens*, and *Schmidtea mediterranea* was assessed*.* In this analysis, one *C. teleta* Ago clustered with miAgos, while the other two fell in the PIWI clade. *C. teleta* PIWI1 and PIWI2 group with *D. melanogaster* ping-pong partners Ago3 and Aub, respectively, suggesting piRNA biogenesis is active. Missing from *C. teleta* was a siAgo, which is present in basal planarians. The absence of siAgo is correlated with *C. teleta* only having a single Dicer protein that is related to miRNA processing rather than siRNA processing (Figure 1A). In contrast, *S. mediterranea* possesses two, one which groups with miRNA-processing Dicer and the other with siRNA-processing Dicer (Supplementary Figure S1).

To understand the Universe of small RNAs coordinated by these factors, small RNA sequencing was performed on RNAs extracted from early embryos (1–4 cell), late embryos (mid gastrula), larvae, and adult anterior (mouth-segment 12) and posteriors (segment 12-anus) separately for males and females. *C. teleta* ovaries are in posterior segments, while testes are in anterior segments; thus, the four adult libraries represent separately male and female gonads, as well as soma only anterior and posterior. In total, 187M small reads were acquired across the libraries, for which 84% were mapped to the *C.teleta* genome. Highly redundant sequences that reported more than 100 alignments per read were excluded from mapping (Supplementary Table S1). For initial assessment of small RNA species, all libraries were combined, followed by mapping of 15–32 nt reads and application of a small RNA loci finding strategy based on read sites with coverage ≥ 200 reads and merging of features within 500 bp. From this, >20,000 locus-expressing small RNAs were identified (Supplementary File S1). The size distribution of mapping reads to all loci showed two peaks, one at 22–23 nt and another at 29–31 nt (Figure 1B). Based on observations from other invertebrates, these two peaks likely represent Ago-loaded Dicer products (miRNAs/siRNAs) and piRNAs, respectively. The next size distribution per locus was calculated, which allowed segregation into nine clusters (Figure 1B). A super majority (∼90%) of loci represented by six of the nine clusters showed mapping of 29–31 nt reads, suggesting that piRNA-producing regions are the most common small RNA loci in *C. teleta*. The remaining three loci seem to either represent miRNAs or the results of degradation (debris). Intersection of known miRNAs from public annotations found that ∼70% were in cluster 2 and ∼30% were in cluster 4. A single known miRNA (*bantam*) was found in cluster 1.

When comparing the total alignments from each cluster, the most significant were the two largest apparent piRNA-representing clusters and the two containing most known miRNAs. The others that showed heterogenous sizes (clusters: 3, 7, 8, and 9) or mapping of small, ≤ 20 nt sizes (cluster 1) comprised ∼ 6% of all alignments. Next, we applied seqlogo analysis to further characterize small RNAs represented in each group (Supplementary Figure S2). Cluster 1 was predominantly represented by the sequence of the miRNA *bantam*—the one miRNA found in this group. The other miRNA clusters had little bias, which 1T (or 1U) bias was seen for several putative piRNA groupings, such as cluster #4. Together, these results show the expected miRNA but also abundant piRNA populations in *C. teleta* datasets.

### *C. teleta* miRNAs

To further examine *C. teleta* small RNA classes, we first sought to annotate miRNAs using the miRDeep2 algorithm (Supplementary File S2–S8) (Friedländer et al., 2008). In total, ∼700 potential miRNAs were found by the method based on alignments from combined small RNA sequencing guided by existing annotations from *C. teleta*, *Eisenia fetida*, *Crassostrea gigas*, and *Lottia gigantea* (Bhambri et al., 2018; Kozomara et al., 2018; Fromm et al., 2021). Of the 102 annotations in MirGeneDB, 99 were found (Supplementary File S3). The three missing miRNAs (miR-2-o36, miR-2690, and miR-33) were represented by reads in the combined sequencing libraries and were likely overlooked by the algorithm due to duplicates in the case of miR-2 or other issues identifying hairpin folds (Supplementary File S4). Nevertheless, this shows that the depth of sequencing described in this study is sufficient to uncover known *C. teleta* small RNAs. Alongside confirming known miRNAs, 19 “homology rescued” species were found either as novel duplicates of *C. teleta* miRNAs or having similarity to known miRNAs from *Eisenia fetida*, *Crassostrea gigas*, and *Lottia gigantea* (Supplementary File S5). In some cases, these homology-rescued annotations appear to result from near identical duplication of *C. teleta* miRNAs or to have arisen earlier in the annelid lineage (Supplementary File S6).

**FIGURE 2:**
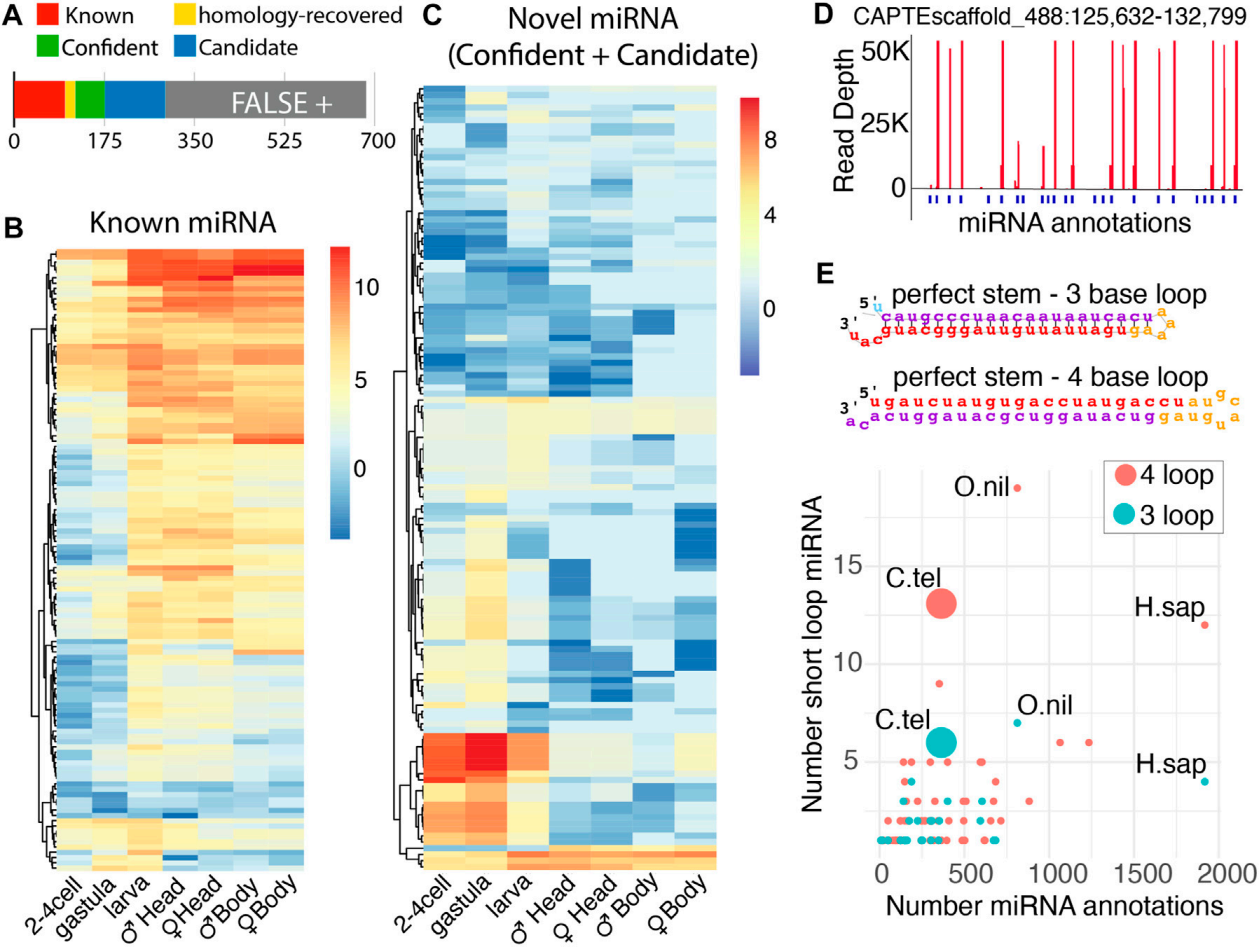
Identification of known and novel miRNAs in *Capitella* by miRDeep2. **(A)** miRNAs discovered through the mirDeep2 algorithm. The number of miRNAs below the bar indicates cumulative miRNAs in each group. Roughly half of candidate miRNAs were rejected as false positives. **(B–C)** Distribution per loci of miRNAs across developmental stages for both known **(B)** and novel miRNAs **(C)**. Adult male and female samples were divided into anterior and posterior ends (the head and body, respectively). The color scale indicates log2 (read per million mapped) **(D)** *C. teleta* clustered the miRNA locus that encodes many embryo-expressed miRNAs. **(E)** Distribution of miRNA hairpins with small terminal loops. Hairpins were divided in 3 nt or 4 nt loops and had characteristic perfect base-pairing in the stem. The number of miRNA annotations against the number of short-loop miRNAs. Other organisms are listed for comparison (*H. sapiens* and *O. niloticus*).

Through this analysis, we were able to annotate novel miRNAs, which were individually vetted based on the expression, presence of Drosha and Dicer cleavage signatures, and precision of 5′ end processing (Figure 2A) (Supplementary File S5–S7) (Mohammed et al., 2018). Out of 569 predicted novel miRNAs, 58 showed all features and were categorized as confident (Supplementary File S7), and 117 were denoted as candidates due to suboptimal features that were not indicative of RNase III processing (Supplementary File S8). The last group of 390 was labeled as false positives and was excluded from further analysis. Significant duplication was seen in novel *C. teleta* miRNAs with only 32 unique confident and 91 unique candidate species identified. Taking the redundancy of known and homology-rescued miRNAs into consideration, there are 99 confident unique miRNA species and 190, if candidate miRNAs are included. Additional sequencing may promote the identity of candidate miRNAs to a confident status and uncover additional miRNAs. Based on efforts in *Drosophila,* saturating the sequencing depth for recovery of all miRNAs in the 333 Mb genome of *C. teleta* would be achieved with ∼600 million reads (Mohammed et al., 2018). This study only provides 30% of the requisite depth to exhaustively annotate miRNAs—particularly low abundance non-canonical species.

Examining the expression pattern of known miRNAs across development showed a general trend where a greater collection of known miRNAs increases over time, comparing embryonic stages to larval and adult stages (Figure 2B). This has been observed in other species and reflects the greater cell type diversity that arises during differentiation (Flynt et al., 2007). The greater known miRNA diversity is found in *C. teleta* anterior regions also likely due to the greater number of cell types found in tissues like the brain and pharynx. Minor differences are seen between genders for both posterior and anterior segments. The expression of miRNAs is strikingly similar between the male and female body, suggesting only a negligible role in ovary development. An opposite arrangement was seen with novel miRNAs. Here, a substantial fraction was more abundant during the post-zygotic stage through larval stages. They were not observed in the gravid female body sample, suggesting they are either produced from embryonic transcription or maternally deposited as intact precursors. The discovery of these miRNAs is likely due to sampling of embryonic stages, which was not performed for other annelids or mollusks. Considering their prolonged expressions, these miRNAs may have a role in regulating metamorphosis. Consistent with this, GO analysis of targets predicted by the TargetScan algorithm found enrichment for response to oxygen, nitrogen, peptides, hormones, and insulin signaling (Supplementary Figure S3A) (Agarwal et al., 2015). Ingestion does not begin until swimming larvae settle and metamorphose; therefore, at early stages, animals may have a modulated insulin signaling pathway.

Several of these early-staged novel miRNAs were noted as having a nearly identical expression during early embryogenesis (Figure 2D). Upon further inspection, many highly abundant miRNAs were found to be encoded on a single scaffold (CAPTEscaffold_488). Many of these miRNAs appear to be tandem duplications reminiscent of the miR-430 cluster in zebrafish that eliminates maternal messages and the various miRNA clusters found expressed in *Drosophila* testis (Mohammed et al., 2014; Liu et al., 2020). Targets of these miRNAs are enriched for cytoskeletal and histone regulators, which may be involved in the morphological and gene expression changes associated with metamorphosis (Supplementary Figure S3B). It will be intriguing if this miRNA cluster arrangement is present in the genome of other metamorphosing annelids. In addition to this highly expressed early-stage cluster, four other major clusters were annotated on CAPTEscaffold_6, CAPTEscaffold_324, and CAPTEscaffold_60 (Supplementary Figure S4).

During our curation of novel *C. teleta* miRNAs, we noticed an abundance of unusual precursors. In the predicted miRNAs, there were many species where the stem loop exhibited no bulges, and the loop was minimal with either a three- or four-base loop (Figure 2E). In the *C. teleta* genome, we observed 13 species with a four-base loop and six species with a three-base loop. Both were reminiscent of miR-451, which is processed in a Dicer-independent manner (Cifuentes et al., 2010; Yang et al., 2010). However, for these miRNAs, this does not appear to be the case. They exhibited strong evidence of the Dicer cleavage at their loops with reads aligning with precise 3’ nt overhangs. To confirm the abundance of these hairpins was, in fact, unusual; we assessed the abundance of similar precursor miRNAs reported for all species in the miRbase (Kozomara et al., 2018). Out of 271 organisms, only 28 had any three-base loop miRNAs, while only 65 had four-base loops. For both configurations, the only species that had more of these tight loop hairpins was the Nile tilapia, *Oreochromis niloticus*, which appears to have an unusual miRNA biology (Pinhal et al., 2018). The next closest was humans, likely the most deeply sequenced species, with nearly 2000 reported miRNAs. In comparison with the 302 potential miRNAs (confident, known, homology-recovered, and candidate) we reported for *C. teleta*, we recovered even more than the perhaps over-aggressively annotated human genome.

### *C. teleta* piRNAs

**FIGURE 3:**
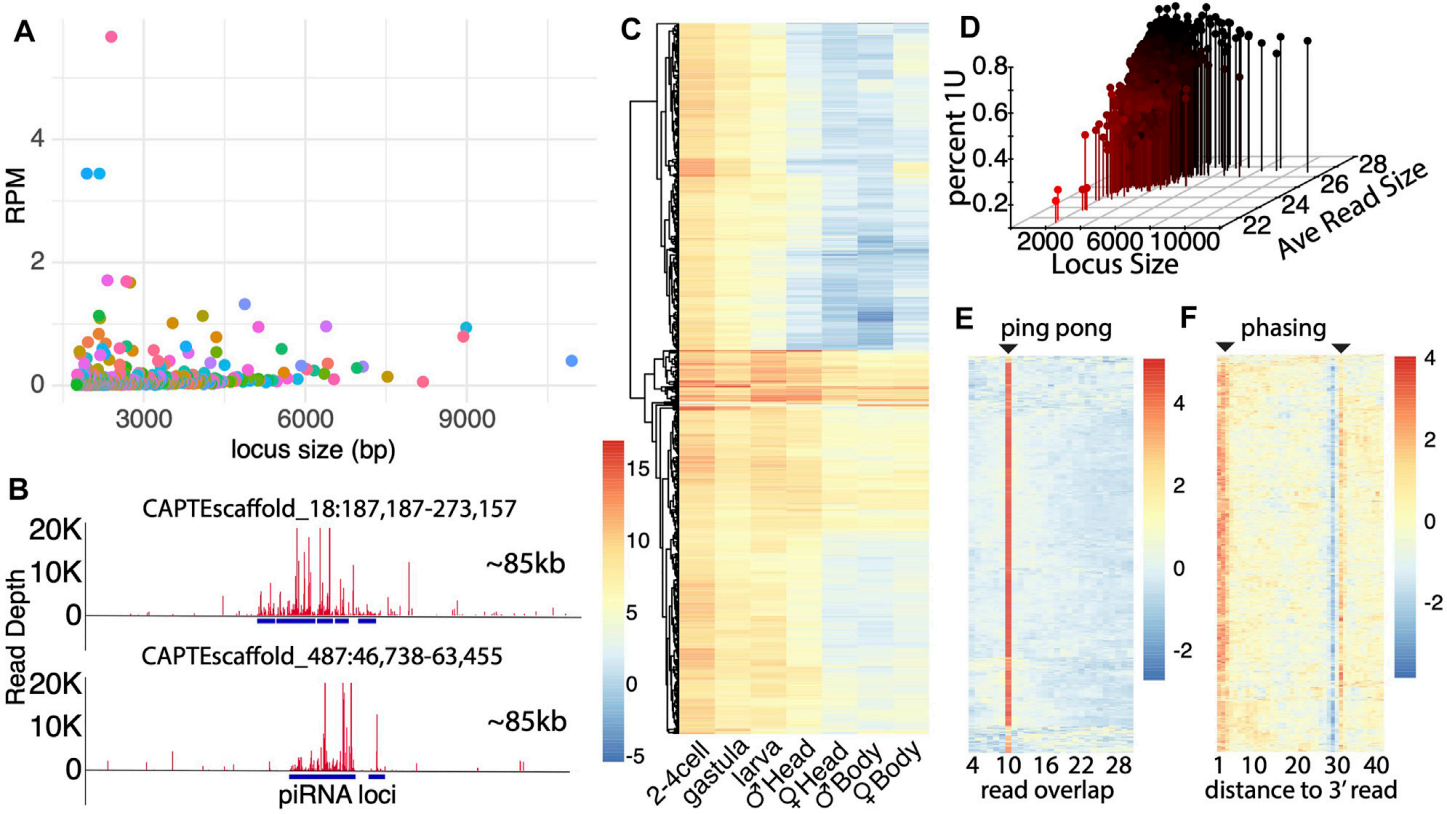
*Capitella* genome contains extensive piRNA loci. **(A)** Size distribution of piRNA loci plotted against read expressions in each locus (Reads per million). Majority of loci are smaller in length with not as much depth. Alternating colors are applied to assist datapoint visualization. **(B)** Graphic representation of two of the largest piRNA loci located in scaffold 18 and 487. The entire window represents 85 Kb and a highest depth of reads of 20k each. **(C)** piRNA expression across developmental stages. Adult male and female samples were further divided into anterior and posterior ends (the head and body, respectively) to record the expression in gonads (male gonads are present in the anterior end, and female gonads are present in the posterior end). Scores are based on normalized log2(RPM) values. **(D)** Percentage of the uridine bias at first position for each piRNA locus. Percentage of bias is plotted against the piRNA locus size and average read size. **(E)** Ping-pong signature in piRNA loci. The horizontal axis shows the number of nucleotides overlap between reads in each locus. There are few locus exceptions (bottom) that do express the 10 nt overlap. Values are based on the z-score. **(F)** Phasing signature in piRNA loci based on the expression of U bias at each nt position. There is a strong presence of U bias at the first position. The low expression at position 29 marks the splicing site for the RNA read, and the strong U bias at position 31 is indicative of the first nucleotide of the next read.

Even more abundant than miRNAs in datasets were the apparent piRNAs in *C. teleta*, both in terms of the number of putative piRNA loci and abundance of piRNA molecules (Figure 1B). To analyze all possible piRNAs in *C. teleta*, we identified all alignments of 15–31 nt reads that exhibit 10 nt overlaps, which is indicative of ping-pong processing and trigger the piRNA-mediated cleavage that initiates phasi-piRNAs (Mohn et al., 2015a). This read mapping profile was overlaid with all loci with 15–31 nt aligning reads that were over 1,000 bp long. From this, 976 loci were found that had both significant read accumulation and exhibited read mapping with 10 overlaps. To assess these piRNA loci, we first compared the size and expression of each (Figure 3A). Loci run the gamut from 1,000 to 10,000 bp with a relatively low expression for any given locus. This was rather unexpected. In other invertebrates, massive loci are present that generate extensive amounts of piRNAs. An example of this is the ∼180 kb “piRNA cluster” flamenco locus, which in *Drosophila* serves as a repository of unlicensed transcripts (Goriaux et al., 2014). No such locus was found in *C. teleta*. The most extensive piRNA loci found in our efforts were less than 20 kb (Figure 3B). This could be a consequence of the relatively poor assembly of the *C. teleta* genome; however, even in significantly poorer assembled genomes, multiple such small RNA loci can be identified (Mondal et al., 2018).

Another unexpected aspect of the piRNA expression in *C. teleta* is the greater expression of piRNA in early developmental stages vs. later stages (Figure 3C). Comparing expressions across development found the highest level of expression in embryo stages, which was not seen in gravid females. Thus, like the cluster miRNAs, piRNAs are either the product of early transcription or post-fertilization processing. Here, more so the cluster miRNA expression is higher than in embryonic stages, suggesting the later–post-fertilization processing. This is similar to what is seen in *Drosophila*, where maternally deposited piRNA precursors serve to propagate piRNAs in the germline (Fabry et al., 2021). Nevertheless, some of the most highly expressed piRNA loci are expressed throughout development in all conditions. We also observed that gonad-containing tissues (the male head and female body) have higher expressions of piRNAs, consistent with the presence of PIWI1-positive cells in germ cells (Giani et al., 2011). However, even the non–gonad-containing tissues have abundant piRNAs, which might be associated with regeneration processes based on the PIWI1 expression in the posterior growth zone, for example.

To further characterize biogenesis of putative piRNAs, we compared parameters that were previously described for piRNA processing. First, we simultaneously calculated the percent of reads aligning to each locus, where the first base was a “U”, the average length of mapping reads, and locus length. By intersecting these parameters, we found that longer loci and reads with higher average read lengths exhibit greater 1U bias. Thus, like other species, piRNAs are characterized by longer reads (26+ bp) and 1U bias. Several short-read loci were recovered that did not follow this trend. They appear to be loci where degradation fragments are abundant but nevertheless may be the targets of piRNA subjects to turnover by the piRNA-mediated cleavage. In addition to the first base bias, we also examined ping-pong and phasing biogenesis (Figures 3E,F). Using a similar computational approach to find 10 nt overlap loci, we sought to visualize ping-pong biogenesis in all loci. Unsurprisingly, in these loci, we observed that most have a very clear signature of 10 nt overlaps (Figure 3E). However, a handful of loci are present that do not have a clear signature. Next, we also assessed the presence of piRNA phasing (Figure 3F). Here, we found a strong phasing signature both at the 1–3 nt position of 1U reads and at the following 30 nt position. Just like the ping-pong signature, there are some clear exceptions. Thus, while piRNA biogenesis described in systems such as *Drosophila* predominates, a handful of exceptions remain.

**FIGURE 4:**
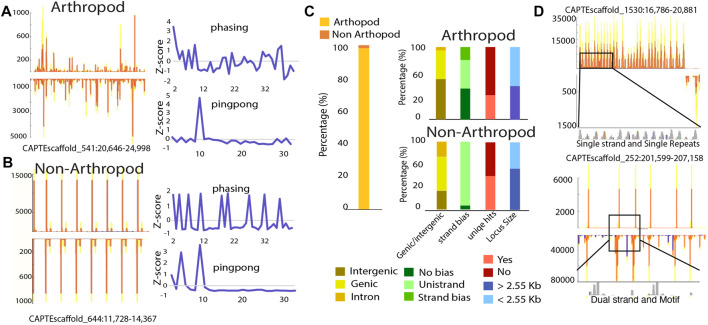
Non-canonical piRNA loci consist of repetitive sequences. **(A–B)** Graphical representation of arthropod-like and non–arthropod-like loci, respectively. Non–arthropod-like loci are characterized by the presence and expression of repetitive patterns with reads containing the same sequence. **(C)** Characterization of arthropod- and non–arthropod-like loci. Percentage of piRNA loci that are arthropod- or non–arthropod-like (left). For each category, they were subdivided based on the physical features of each locus (right). Genomic location was divided into intergenic regions, genic (loci spanned through exons and introns), or intronic (loci were located inside a large intronic region) region. The transcriptional bias was divided into no bias (total transcripts for one strand < 5x total transcripts of the opposite strand), strand bias (total transcripts of one strand ≥ 5x but ≤ 10x total transcripts of the opposite strand), and unistrand (total transcripts of one strand > 10x total transcripts of the opposite strand). Loci were also divided based on the presence of novel mapped reads (unique hits) and by the locus size (larger or smaller than 2.55 Kb). **(D)** Non-arthropod loci were also divided into the type of repetition present at each locus (single read repeats or motifs with multiple repetitive reads) and into the presence of repeats on one strand or both strands (single or dual strand).

Comparing piRNA biogenesis patterns in *C. teleta*, we found that arthropods with ∼98% of loci had shared characteristics such as clear phasing and ping-pong signatures (Figure 4A). For these loci, there is a seemingly random accumulation of reads in the region. The remaining 2%, however, showed a highly distinct expression pattern (Figures 4A,B) (Supplementary File S9). Unlike classic arthropod-type piRNA loci, these atypical small RNA regions present as cluster-like repeating sites. Despite this unusual configuration, these loci share some features with canonical piRNA loci such as partial ping-pong overlaps; however, phasing is not shared. This is likely due to the distinct peaks of piRNAs that are clearly not the product of processive cleavages mediated by Zuc (Supplementary Figure S5).

To further characterize these loci, we curated features of both arthropod and non-arthropod varieties (Figure 4B). For both locus types, they are found within intergenic, genic, and in intronic regions. Proportionally many non-arthropod types are encoded within gene annotations, indicating they are associated with *bona fide* transcripts. Classic piRNA loci are found in all configurations where piRNAs are produced from both strands, biased towards one strand, or only expressed from a single strand. In contrast, the majority of atypical loci are uni-strand. A major concern with annotation of piRNAs is multi-mapping of piRNA-derived reads. We found that for both types, unique mapping hits are present in roughly half of the non-arthropod loci. This further suggests that the atypical loci are confident sources of piRNAs and are not found due to mapping artifacts. Finally, we found that both types of loci have similar sizes.

Inspection of sequence identity at non-arthropod loci revealed that for some, they consist of short repeat elements, while others have more complex sequences (Figure 4D). Even for highly repetitive loci, polymorphisms are present within different repeat elements that when perfect mapping is enforced, the expression profile is retained; all 16 loci retained their profiles. Thus, it would further appear that the expression of these unusual piRNAs is not a mapping artifact but represent an alternate biogenesis mechanism. Moreover, similar loci have been noted in the pacific oyster, *C. gigas*, suggesting that this undescribed piRNA biogenesis mechanism may be present in multiple organisms (Huang et al., 2019).

### A Distinct siRNA Pathway Is Absent in *C. teleta*

**FIGURE 5:**
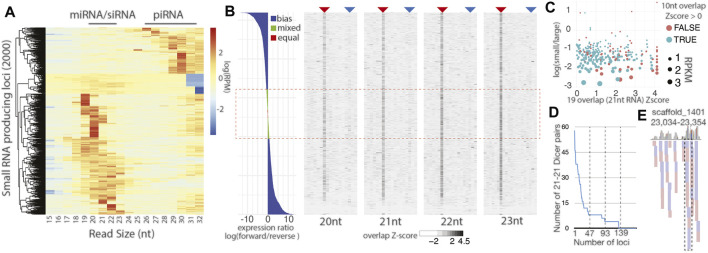
Little evidence for distinct siRNA biogenesis in *C*.*teleta*. **(A)** Size distribution of small RNA species in *S. mediterranea*. Compared to a similar analysis in Figure 1B, there is a substantially greater fraction of small RNA loci with a distribution suggesting the miRNA/siRNA identity. **(B)** Characterization of all *C. teleta* loci with the alignment of small 20–23 nt reads with a coverage > 30. The left panel is strand bias expressed as the log ratio of forward and reverse mapping reads. Right four panels show the Z-score for read overlaps starting from 4-base overlaps to full-read overlaps. The analysis is performed separately for reads of different lengths (20 nt, 21 nt, 22 nt, and 23 nt). The red arrow shows the 10 nt ping-pong overlap, and blue arrow dicer overlaps (2 less than the full overlap). All panels represent the same sorted loci top to bottom. The red dashed box indicates reads with mixed or equal number of alignments on both strands. **(C)** Scatterplot of loci with 21 nt reads with the Dicer-processing signature and Mixed/Equal read mapping. The Z-score of the Dicer overlap compared to the log of small (20–23 nt) to large (26–32 nt). Points are color-based on whether a positive ping-pong Z-score was also observed and sized scaled by RPKM for locus. **(D)** Quantification of 21 nt reads overlapping with Dicer overhangs at loci with a Dicer signature Z-score > 1. **(E)** Alignment of 21 nt reads overlapping by 19 bases at the locus measured in part D with the highest number of 21 nt−21 nt pairs. The dashed box shows the only example of a phased set of small RNAs.

Unlike miRNAs and piRNAs, our investigation did not uncover apparent endogenous siRNAs. This is apparent when *C. teleta* small RNA loci are compared to those of the planarian, *Schmidtea mediterranea,* a related bilateral animal where RNAi induced by long dsRNA is confidently validated (Rouhana et al., 2013). Small RNA studies in this planarian report all three classes of small RNAs (miRNAs, siRNAs, and piRNAs). There are substantial differences in *S. mediterranea* RNAi machinery, where there are 2 Dicers and 3 Agos compared to *C. teleta* 1 Dicer and 1 Ago (Figure 1A; Supplementary Figure S1). To further probe the differences in small RNA biogenesis between annelids and platyhelminths, we compared read size patterns of high-expressing small RNA loci found with the method described in Figure 1B for *S. mediterranea*, using a public small RNA dataset (Friedländer et al., 2009) (Figures 1B, 5A). In the planarian analysis, most loci have mapping in the 19–23 nt size range and are likely siRNAs or miRNAs. Only a quarter of the small RNA loci were 29–30 nt piRNAs, a striking difference to *C. teleta*.

Next, we sought small RNA loci with Dicer-processing signatures based on the presence of reads with 2 nt overhangs that range from 20 to 23 nt long (Mondal et al., 2020). These alignments were intersected with highly expressed loci (Figures 1B, 5A). Comparison of the 500 loci with the highest small RNA expression by size and Dicer overhang mapping revealed a substantially different distribution of loci when compared to *S. mediterranea* (Supplementary Figure S6A). *C. teleta* loci were substantially shorter than some examples in *S. mediterranea* that were longer than 40 kb. In *C.teleta*, the loci typically corresponded to miRNAs, while in *S. mediterranea* they are larger, encoding a processively cleaved dsRNA. The largest locus from each species was compared to assess Dicer signatures (Supplementary Figures S6B, S6C). In the locus from *C. teleta*, a couple isolated mappings were seen whereas the *S. mediterranea* locus showed 21 nt abundant reads that overlapped with 2 nt 3′overhangs. Thus, siRNA biology in annelids is, at a minimum, significantly reduced after diverging from planarians, which have impressive siRNA-generating loci.

To further probe the apparent absence of siRNA-class small RNA in *C. teleta*, we assessed all possible sources involving dsRNA formed from dual strand transcription. Arthropods have 100s of such endogenous siRNA-loci that correspond to *cis*-NAT transcripts and transposable elements, as well as cryptic dsRNA-producing loci (Okamura and Lai, 2008). Due to the incomplete nature of *C. teleta* gene annotations, only 122 instances of overlapping gene annotations are reported, and among those, only 22 are overlaps greater than 40 bases (Supplementary Figure 6D). Alignments to the overlapping gene regions are limited with very few showing alignments of small RNAs in the size range of Dicer products (20–23 nt). Even if these reads are present, they do not coincide with opposite strand alignments that would be expected from the Dicer cleavage (Supplementary Figure 6E). To circumvent this limitation, we identified all regions of the *C. teleta* genome with a mapping of 20-23 nt reads and greater than 30-read coverage that did not correspond to miRNAs (Figure 5B). For each of the 5,191 loci, the ratio of forward to reverse reads was calculated and binned in to three categories: “bias” where there was 2-fold greater alignments on one strand, “mixed” where the ratio fell between 1.2 and 1.9 times greater on one strand, and “Equal” with the ratio being 1–1.1 (Zhang and Ruvkun, 2012). The alignment of 20–23 nt reads, at these loci, were simultaneously assessed for read overlaps such as 10 nt overlaps seen for ping-pong processing and 2 nt less than full overlaps associated with the Dicer cleavage. Loci were sorted by the log strand bias ratio and visualized as a bar plot for bias and heatmaps for read overlap Z-scores (Figure 5B). Combined mixed and equal loci were roughly a quarter (24%) of the loci. In the overlap heatmaps, a strong ping-pong signature was observed, suggesting that these 20–23 nt reads are predominantly piRNA-type possibly truncated by trimming. Indeed, ∼60% of the loci overlap with cluster 4 from Figure 1B. Simultaneously, a very minor signal was observable at the Dicer overlap position, particularly for 21 nt reads (Figure 5B). However, these overlaps were not more prevalent in mixed or equal strand alignments with only 21% of loci with positive Dicer overlap Z-score values in these categories. Enrichment would be observed if the portion with the Dicer signature exceeded 24%, suggesting no correlation between Dicer signatures and sites of potential dual strand transcription.

Next, we focused on loci from Figure 5B that had mixed or equal strand mapping and a positive Z-score for Dicer overlaps in 21 nt reads (19 nt overlaps) (Figure 5C). For this subset, we sought to understand the identity and expression of small RNAs by comparing RPKM values, the presence of the ping-pong signature, and the relative abundance of short (20–23 nt) reads, relative to long (26–32 nt) reads. Nearly all loci had greater alignment of longer reads than shorter reads with only 5 (1.8%) with more short *vs.* long. Consistent with the bias towards longer reads, ∼80% of loci also had positive Z-scores for ping-pong processing. Further highlighting the bias towards piRNAs, the expression per locus tapered significantly away from those with a greater portion of piRNA-sized read alignments. Next, we quantified the number of 21 nt read pairs that overlap by 19 nt. Calculation of Z-scores in Figure 5B reports overlap biases of the indicated read size paired with a read of any other size; thus, we sought to quantify 21 nt reads paired with other 21 nts (Figure 5D). From this, we found that over 75% of loci in Figure 5C have less than 10 unique 21 nt reads (5 pairs). To further probe these loci for evidence of Dicer processing, we inspected the 25% of loci that had the highest number of 21–21 nt pairs overlapping by 19 nt. Nearly all pair alignments were isolated, suggesting distributive processing (Figure 5E; Supplementary Figure S7). Indeed, of the 47 loci examined, only six had phased reads that would occur from processive cleavage. Together, there is little evidence for siRNAs processed from long dsRNAs. Instead, our results suggest the *C. teleta* Dicer may be involved with the cleavage of some substrates but may only be engaging in distributive processing.

To examine potential siRNA-related enzymatic activities in greater detail, we characterized domains and catalytic residues in *C. teleta* Dicer and Ago (Supplementary Figure S8). A key domain involved in the processive Dicer activity is the N-terminal helicase domain that hydrolyzes ATP as a part of substrate engagement (Sinha et al., 2018). While *C. teleta* Dicer has a recognized helicase domain, several of the key residues involved in ATP hydrolysis are altered in comparison to *D. melanogaster* Dicer2 and *H. sapiens* Dicer, which both show a processive behavior. *C. teleta* Ago possesses the same slicer residues as *D. melanogaster* Ago1 and Ago2; however, this is expected as miRNAs, when pairing extensively with a target, can direct slicing.

## Discussion

This comprehensive analysis of small RNA populations in the marine annelid *C. teleta* shows the presence of only two RNA classes, miRNAs and piRNAs, with an apparent loss of endogenous siRNAs. This is consistent with the absence of siAgo proteins and was borne out by lack of compelling signatures of Dicer processing. Similar configurations of RNAi pathway components are found in other lophotrochozoans, such as gastropods, cephalopods, and brachiopods, and leeches (Jehn et al., 2018). The exception might be bivalves where there are two Ago proteins; however, they seem to be the result of duplication of a miAgo and not a distinct miAgo/siAgo pair. The benefit of losing the siRNA pathway in these animals is not clear. In ecdysozoans, siRNAs have a significant role in antiviral defense (Gammon and Mello, 2015). It is curious that a useful mechanism would be jettisoned by *C. teleta* and possibly other lophotrochozoans. However, a similar event occurred independently in the deuterosomes, including basal echinoderms (Waldron et al., 2018). In the place of siRNAs, in both lophotrochozoans and echinoderms, piRNAs appear to take the place of viral siRNAs. Poriferans, in contrast, seem to mount an siRNA response to viruses indicating that piRNA-mediated antiviral defense has evolved independently.

In addition to the loss of siRNAs, we observed unusual miRNA hairpins that do not exhibit unpaired stem bases with extremely short hairpins. More so than nearly all other species, they are abundant in *C. teleta*. The exception is the Nile tilapia, which has an unusual collection of miRNAs. Further research is needed to establish whether this is coincidental or a product of changes in the Dicer enzymatic activity. Interestingly, these hairpins would be excellent candidates for an miR-451-like Dicer-independent biogenesis; however, they do not appear to mature through this pathway. Reads align to hairpins in a pattern expected for Dicer processing, suggesting a competency for *C. teleta* Dicer to process this type of hairpin that is not present in vertebrate Dicer proteins. It will also be interesting as to whether other annelids have these same tight hairpins found in *C. teleta* or if this worm is an outlier like the Nile tilapia.

As we found with other small RNA classes, we also observed some unexpected features of *C. teleta* piRNAs. First, we did not observe a massive piRNA cluster which is observed in a variety of animals. Instead, we found loci that reached a maximum of 10kb, none of which exhibited high expression. This does not suggest, however, that piRNA function has diverged significantly from arthropods. Recent efforts to delete large piRNA clusters from *Drosophila* found that they were dispensable for fertility and transposon control (Gebert et al., 2021). Thus, in *C. teleta*, even though there is no large piRNA clusters, piRNAs can be expected to retain their role in transposon control. However, when this might happen during *C. teleta* development is questionable. Unlike many animals, we did not observe that the piRNA expression correlated with gonads. In this worm, we found a greater expression in the early embryos. Thus, germline defense may occur in post-fertilization vs. during gametogenesis, and the collection of inherited piRNAs peaks at a different point in this animal’s life cycle.

In this species, we also observed unusual piRNA loci that are produced by an undescribed mechanism. Unlike many piRNAs, these are derived from simple repeat sequences. Tests that involve examining perfect and unique read mapping found that these piRNA loci were not annotated as a result of read mapping artifacts. Also these types of loci are found in related species (Huang et al., 2019). Processing mechanisms of these piRNAs are unknown and will require genetic studies that define both processing but also the function of piRNAs derived from these loci. It will also be intriguing to see if they have a distinct activity such as the trigger, responder, and trailer piRNAs described in *Drosophila* (Mohn et al., 2015b). Alternately, these piRNAs may be like *C. elegans* piRNAs produced by discrete transcriptional units.

Similarly, the most impactful outcome of this report is the guidance offered for developing RNAi approaches in animals that share *C. teleta* small RNA biology. The lack of a dedicated siRNA pathway suggests that long dsRNA approaches used in ecdysozoans and planarians may not be advisable. It would be unsurprising that dsRNA molecules have become a pathogen pattern and could lead to an antiviral response instead of a specific gene knockdown. As an alternative, technology that exploits miRNA biology, as it is deployed in vertebrates, should be effective. The single Ago in *C. teleta* has slicer residues; thus, potent gene silencing should be possible. Further, the unusual miRNA structures we observed may provide additional configurations for exploiting the miRNA pathway. Finally, it may also be possible to exploit piRNA mechanisms by introducing synthetic RNAs that possess complementarity to known piRNAs (Mondal et al., 2020). This species has abundant piRNAs which would allow a variety of cell types to be targeted. In addition, there are two mechanisms of action (phasing and ping-pong amplification) that could be modeled for gene silencing technology.

## Data Availability

The datasets presented in this study can be found in online repositories. The names of the repository/repositories and accession number(s) can be found below: https://www.ncbi.nlm.nih.gov/sra/PRJNA777269.
