# Supplementary material for: The Small RNA Universe of Capitella teleta
Source: Front Mol Biosci. 2022 Feb 25;9:802814. doi: 10.3389/fmolb.2022.802814 (PMC8915122; doi:10.3389/fmolb.2022.802814)
Supplement: Supplementary file 1 [file DataSheet1.ZIP › Supplement/Supplementary_info.docx]

**SUPPLEMENTAL FIGURES**


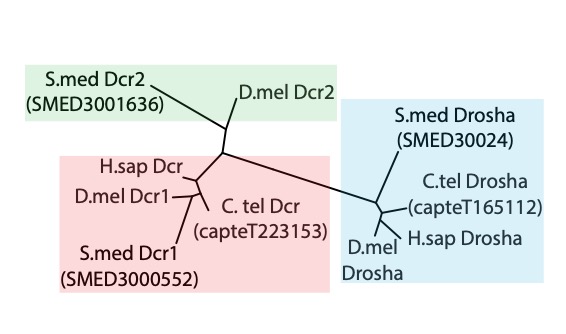


**Supplemental Figure 1**. Comparison of C. teleta, H. sapien, D. melanogaster, and S. mediterranea RNaseIII containing protein. miRNA Dicer group indicated by red box, Drosha, by blue box, and siRNA Dicer by green.

**Supplementary Figure 2.** Seqlogo analysis showing nucleotide bias of first 15 bases of reads aligning to clusters indicated in Figure 1B. Cluster 1 is dominated by bantam miRNA. Cluster 8 is dominated by tRNA-Glu fragments. Distribution is given in bits.


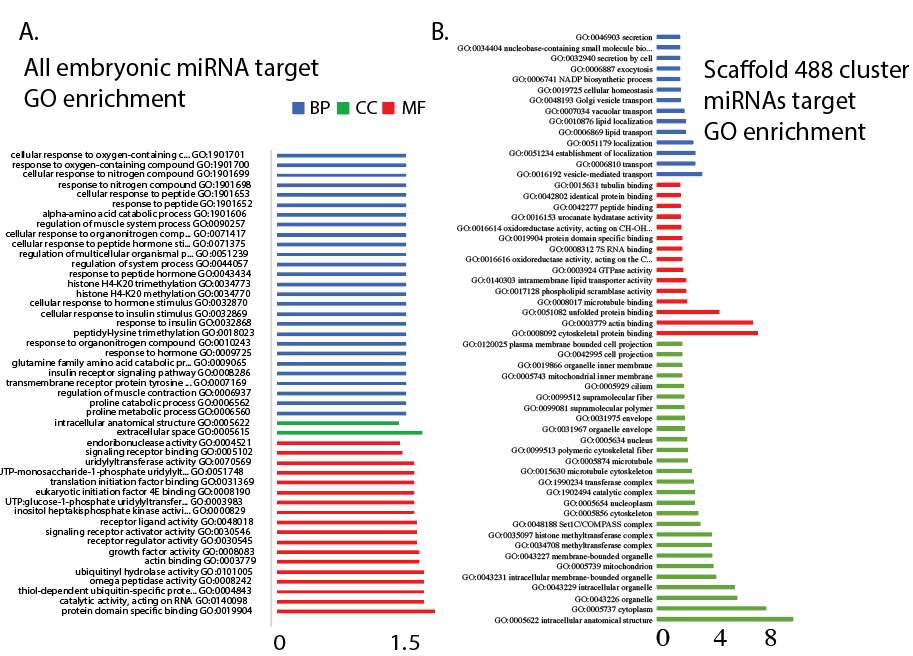


**Supplementary Figure 3**: GO Analysis of predicted target of embryonic novel miRNAs. Targets were chosen based on p-value < 0.05. Scale is given in -log values. Legend: Biological Process (blue), Cellular Component (green), and Molecular Function (red). A) Target GO enrichment for all embryo (early and late stage) miRNAs, and Target GO enrichment for Scaffold_488 cluster.

**Supplementary Figure 4. Other miRNA clusters in C. teleta.** Red density plots indicate read coverage. Blue dashes confirmed miRNA annotations recovered from mirDeep2.

**Supplementary Figure 5. Diagram of Arthropod and non-Arthropod loci.** Sense strand indicated by blue and antisense by red. Grey box indicate pingpong signature. In arthropod like loci sense RNAs are seen in a distributed phasing arrangement beginning in different registers. Antisense RNAs derived from the ping pong cycle are complementary. In non-arthropod loci discrete regions are found that precisely produce sense RNAs. Antisense RNAs can be found that have the overlap of ping pong processing.

**Supplemental Figure 6.** A) Dicer alignment signatures scatterplot from *C. tel* (red) and *S. med* (blue)*.* The 500 largest loci were analyzed in order to observe presence of Dicer cleavage. B) *S. med* locus screenshot from IGV containing siRNAs with Dicer signature, marked by the presence of 21nt reads and 2nt overhangs. C) *C. teleta* locus screenshot from IGV showing scarce presence of Dicer reads and no 2nt overhang or overlap of reads, thus indicating absence of siRNAs. D) 37 gene annotations that overlap by greater than 30 bases. Blue bars show quantification of all reads from 15-32 bases aligning to overlaps. Green bars indicate short reads (20-23nt) aligning to loci. E) example of locus indicated by red arrow in part D. A single read accumulation is seen without overlaps with complementary reads.

**Supplemental Figure 7.** Additional examples of candidate siRNAs with phased small RNAs. In all cases phasing did not proceed past two consecutive reads, indicating that *C. teleta* Dicer has extremely limited processive activity.

**Supplemental Figure 8.** Enzymatic features of *C. teleta* Dicer and Ago. A) Domains of *C. teleta* Dicer. B) Catalytic residues in C. teleta Dicer helicase domain response for ATP hydrolysis and processive dsRNA cleavage. C) Catalytic residues present in C. teleta Ago indicate intact Slicer activity.

**Supplemental Figure 9.** Computational Pipeline for smallRNA sequencing analysis. Parameters used in software packages indicated in boxes. Grey boxes indicate core, shared computational methods such as mapping parameters and fastq file subsetting. Yellow boxes show miRNA annotation methods involving standard miRDeep2 mapping parameters. Violet boxes show methods for determining size distribution of reads in Figure 1B. Teal boxes describe piRNA identification approaches used for figure 3 and 4. Orange boxes are siRNA biogenesis analysis in Figure5 and Supplemental figure 6.

**SUPPLEMENTARY TABLES:**

**Supplementary Table 1. Mapping statistics**

|  |  | Mapping | | Suppressed due to -m100 | |
| --- | --- | --- | --- | --- | --- |
| Libraries | Total reads | Reads | Percentage (%) | Reads | Percentage (%) |
| Early embryo | 32063210 | 25628334 | 79.93 | 4513387 | 14.08 |
| Lateembryo | 27379019 | 16426942 | 60 | 8956016 | 32.71 |
| Larvae | 24056642 | 21067556 | 87.57 | 2419890 | 10.06 |
| Malehead | 35642435 | 32856837 | 92.18 | 2087423 | 5.86 |
| Malebody | 18608098 | 15654895 | 84.13 | 729243 | 3.92 |
| Female head | 24300814 | 22650529 | 93.21 | 722143 | 2.97 |
| Female body | 25481648 | 22549775 | 88.49 | 918359 | 3.60 |
| combined | 187531866 | 156834868 | 83.63 | 17926571 | 9.56 |

**SUPPLEMENTARY FILES**

**Supplementary File 1** **(SmallRNA_loci.txt)** Loci from Fig 1B, segregated into clusters based on read size distribution. First column is scaffold, follow by start and stop coordinates columns. Fourth column indicates cluster the region was placed into.

**Supplementary File 2 (miRNA_summary.xlsx)** Summary of miRNA annotation. Each miRNA was given a unique identifier (column 1). Duplicates were labeled by same number, distinct letter. Columns 2-4 represent genomic coordinates (chromosome, begin, end). Columns 5-8 are expression of different features (total, mature, star, and loop) as calculated by miRdeep2. Column 9 is classification into known, homology-rescued, confident and candidate. Column 10 indicates miRNAs from other species that was determined to have homology by miRdeep2.

**Supplementary File 3 (known.html) mirDeep2 output for known miRNAs**

**Supplementary File 4 (knownMissing.pdf) mirDeep2 output for missing miRNAs**

**Supplementary File 5 (homolResc.html) mirDeep2 output for homology rescued miRNAs**

**Supplementary File 6 (homologRescuedAlignments)** Alignment of novel conserved miRNAs to homologs from different species.

**Supplementary File 7 (confident.html) mirDeep2 output for confident miRNAs**

**Supplementary File 8 (candidate.html) mirDeep2 output for candidate miRNAs**

**Supplementary File 9 (Non-arthrod_piRNAloci.txt)** A list of regions encoding non-arthropod like piRNA producing loci in BED format.

**Supplementary File 10 (mirtrace-report.html)** Small RNA library quality assessment
