# Supplementary material for: The Small RNA Universe of Capitella teleta
Source: Front Mol Biosci. 2022 Feb 25;9:802814. doi: 10.3389/fmolb.2022.802814 (PMC8915122; doi:10.3389/fmolb.2022.802814)
Supplement: Supplementary file 1 [file DataSheet1.ZIP › Supplement/SupFile4_knownMissing.pdf]

miRBase precursor : Cte-Mir-2690_pre
Total read count : 10986
Cte-Mir-2690_3p read count 6331
remaining reads : 4655

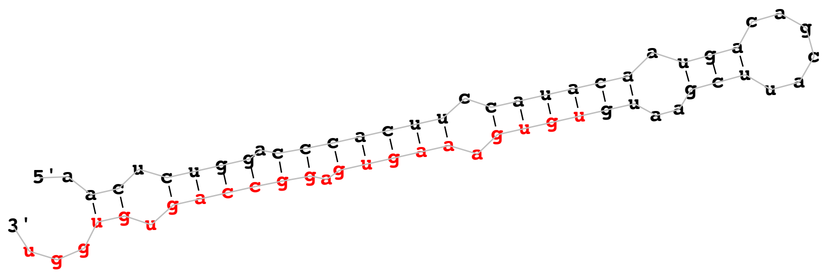

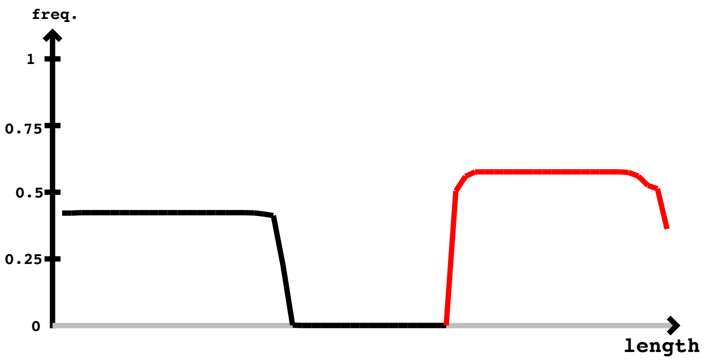

Cte-Mir-2690_3p

5'- aacucuggacccacuuccauacaaugacagcauucgaaugugugaaagugaggccagugugguu -3' exp

.((.((((.(((((((.((((((.(((......)))..)))))).))))).)))))).))...	reads	mm	sample
aacucuggacccacuucca..	9	0	seq
aGcucuggacccacuucca..	1	1	seq
aacucuggacccacuuccau...	45	0	seq
aacucuUgacccacuuccau...	1	1	seq
aGcucuggacccacuuccaua..	2	1	seq
aacucuggacccacuuccaua..	65	0	seq
aacucuggaUccacuuccaua..	1	1	seq
aacCcuggacccacuuccauac...	1	1	seq
aGcucuggacccacuuccauac...	14	1	seq
aCcucuggacccacuuccauac...	1	1	seq
aacuAuggacccacuuccauac...	1	1	seq
aacucuggacccacuuAcauac...	1	1	seq
aacucuggacccacuuccauaA...	2	1	seq
aacucuggaUccacuuccauac...	1	1	seq
aacucuggacccacuGccauac...	1	1	seq
aacucuggaAccacuuccauac...	1	1	seq
aacAcuggacccacuuccauac...	2	1	seq
aacucuggacccacuuccauac...	1983	0	seq
aacucuggacAcacuuccauac...	3	1	seq
aacucuggUcccacuuccauac...	1	1	seq
aacucuggacccacuuUcauac...	1	1	seq
aacucuggaccUacuuccauac...	3	1	seq
aacucuggacccacuCccauac...	1	1	seq
aacucuggacccacuAccauac...	3	1	seq
aaAucuggacccacuuccauac...	1	1	seq
aacucuggacccacuuccauaU...	1	1	seq
Gacucuggacccacuuccauac...	4	1	seq
aacucuAgacccacuuccauac...	8	1	seq
aacucuggacccacuucUauac...	1	1	seq
aacucuggacccUcuuccauac...	1	1	seq
aacuAuggacccacuuccauaca..	1	1	seq
aacucuggacccacuuccaCaca..	1	1	seq
aacucuggacAcacuuccauaca..	3	1	seq
aacucuggacccacuucUauaca..	3	1	seq
Gacucuggacccacuuccauaca..	2	1	seq

Cte-Mir-2690_3p

aacucuggacccacuuccauacaaugacagcauucgaaugugugaaagugaggccaguguggu

aacucCggacccacuuccauaca..	1	1	seq
aacucuggaccAacuuccauaca..	1	1	seq
aacucuggacccacuuccauaca..	2075	0	seq
aacucuAgacccacuuccauaca..	5	1	seq
aacucuggacccacAuccauaca..	2	1	seq
aacucuggacccacuuAcauaca..	2	1	seq
aacucuggacccacuuccauacU..	287	1	seq
aacucuggaAccacuuccauaca..	1	1	seq
aacucuggacccacuuccauaAa..	1	1	seq
aGcucuggacccacuuccauaca..	39	1	seq
aacucuggacccacuAccauaca..	2	1	seq
aacCcuggacccacuuccauaca..	1	1	seq
aacucuggacccacuuccauacC..	27	1	seq
aacAcuggacccacuuccauaca..	2	1	seq
aacucuggacccGcuuccauaca..	1	1	seq
aacucAggacccacuuccauaca..	1	1	seq
aacucuggacccacuuccauacaU.......................................	12	1	seq
aacucuggacccacuuccauacaa.......................................	4	0	seq
.aUucuggacccacuuccauaca..	1	1	seq
.Gcucuggacccacuuccauaca..	1	1	seq
.acucuggacccacuuccauaca..	15	0	seq
.....................caaugacagcauucgaaugug.....................	3	0	seq
......................aaugacagcauucgaaugu......................	1	0	seq
....ugugaaagugaggccagu.....	24	0	seq
....ugugaaagugaggccagug....	81	0	seq
....uguCaaagugaggccagug....	1	1	seq
....ugugaaagugaggccCgug....	1	1	seq
....ugugaaagugaggcUagugu...	1	1	seq
....Agugaaagugaggccagugu...	2	1	seq
....ugugaaagugaggccagugu...	345	0	seq
....ugugaaagugGggccagugu...	3	1	seq
....ugugaaagugaggUcagugu...	1	1	seq
....ugugaaagugaggccagugG...	19	1	seq
....ugugaaagugaggccGgugu...	2	1	seq
....ugugaaagugaggAcagugu...	1	1	seq
....ugugaaagugaggccagugug..	123	0	seq
....uguAaaagugaggccagugug..	1	1	seq
....ugugaaagugaggccaguguU..	5	1	seq
....ugugaaagugaggccGgugug..	1	1	seq
....ugugaaagugaggcAagugugg.	1	1	seq
....uguAaaagugaggccagugugg.	3	1	seq
....ugCgaaagugaggccagugugg.	1	1	seq
....ugugaaagugaggccGgugugg.	1	1	seq
....ugugaaaguAaggccagugugg.	1	1	seq
....ugGgaaagugaggccagugugg.	1	1	seq
....ugugaaagugaggccaAugugg.	1	1	seq
....ugugaaaAugaggccagugugg.	1	1	seq
....ugugaaagugaggccagAgugg.	2	1	seq
....ugugaaagugaggccaguguAg.	4	1	seq
....ugugaaagugaggccagGgugg.	1	1	seq
....ugugaaagugaggccagugugg.	1424	0	seq
....ugugaUagugaggccagugugg.	1	1	seq
....ugugaaagugaggccaguguggU.	21	1	seq
....Agugaaagugaggccagugugg.	2	1	seq
....ugugaaagugaggccagugugA.	5	1	seq
....ugugaGagugaggccagugugg.	1	1	seq
....ugugaaagugaggAcagugugg.	1	1	seq
....ugugaaagugGggccagugugg.	11	1	seq
....ugugaaagugaggccagugAgg.	1	1	seq
....Ggugaaagugaggccagugugg.	3	1	seq
....ugugaaagugaggcUagugugg.	1	1	seq
....ugugaaaguAaggccaguguggu	6	1	seq
....ugugaaagugaggccGguguggu	6	1	seq
....Agugaaagugaggccaguguggu	10	1	seq
....ugugaaagugagCccaguguggu	1	1	seq
....ugugGaagugaggccaguguggu	1	1	seq
....ugugaaagugUggccaguguggu	1	1	seq
....ugugaaagugaggccagugCggu	1	1	seq
....Ggugaaagugaggccaguguggu	4	1	seq
....ugugaaaAugaggccaguguggu	1	1	seq

Cte-Mir-2690_3p

```
aacucuggacccacuuccauacaaugacagcauucgaaugugugaaagugaggccaguguggu
........................................uAugaaagugaggccaguguggu  1     1  seq
........................................ugugaaagugaggccagugAggu  2     1  seq
........................................ugugaaagugaAgccaguguggu  1     1  seq
........................................ugugaaagCgaggccaguguggu  2     1  seq
........................................ugugaaagugaggccaguguAgu  2     1  seq
........................................ugugaaagugaggccagugugAu  1     1  seq
........................................ugugaaagugaggccaguguggA  10    1  seq
........................................ugugaaagugaggccaCuguggu  1     1  seq
........................................ugugUaagugaggccaguguggu  2     1  seq
........................................ugugaGagugaggccaguguggu  2     1  seq
........................................ugugaaagugaggccagAguggu  1     1  seq
........................................ugugaaagugaggcUaguguggu  1     1  seq
........................................ugugaaagugaggccaguUuggu  1     1  seq
........................................ugugaaagugaggccaguguggG  5     1  seq
........................................ugugaaagugGggccaguguggu  18    1  seq
........................................ugugaaagugaggccaguguggu  3323  0  seq
........................................ugugaaagugaggccagGguggu  2     1  seq
........................................ugAgaaagugaggccaguguggu  16    1  seq
........................................ugugaaagGgaggccaguguggu  1     1  seq
........................................ugugaaagugaggccagugugCu  1     1  seq
........................................ugugaaagugaggccUguguggu  1     1  seq
........................................ugugaaaguUaggccaguguggu  1     1  seq
........................................ugugaaagugaggccaguAuggu  1     1  seq
........................................uguAaaagugaggccaguguggu  7     1  seq
........................................ugugaaagugaggcAaguguggu  2     1  seq
........................................ugugaaGgugaggccaguguggu  1     1  seq
........................................ugugaaagugagUccaguguggu  1     1  seq
........................................ugugaaagugaggccagCguggu  1     1  seq
........................................ugugaUagugaggccaguguggu  1     1  seq
.........................................gugaaagugaggccagug....  68    0  seq
.........................................gugaaagugaggccagugu...  1     0  seq
.........................................gugaaagugaggccagugug..  4     0  seq
.........................................gugaaagugaggccaAugugg.  1     1  seq
.........................................gugaaagugaggccaguguggu.  150   0  seq
.........................................gugaaagugaggccaguguAg.  1     1  seq
.........................................gugaaagugaggccaguUuggu  1     1  seq
.........................................guAaaagugaggccaguguggu  1     1  seq
.........................................gugaaaCugaggccaguguggu  1     1  seq
.........................................gugaaagugaggccaguguggG  1     1  seq
.........................................gGgaaagugaggccaguguggu  1     1  seq
.........................................gugaaagugaggccaguguggA  1     1  seq
.........................................Uugaaagugaggccaguguggu  1     1  seq
.........................................gugaGagugaggccaguguggu  2     1  seq
.........................................gugaaagugaAgccaguguggu  1     1  seq
.........................................gugaaagugaggccaguguAgu  1     1  seq
.........................................gugaaagugaggccaguguggu  358   0  seq
.........................................gugaaagugGggccaguguggu  7     1  seq
..........................................ugaaagugaggccagugu...  9     0  seq
..........................................uAaaagugaggccagugugg.  1     1  seq
..........................................ugaaagugaggccagugugg.  18    0  seq
..........................................Cgaaagugaggccagugugg.  1     1  seq
..........................................ugaaagugGggccaguguggu  8     1  seq
..........................................ugaaagugaggccaguguggG  1     1  seq
..........................................Agaaagugaggccaguguggu  2     1  seq
..........................................ugaaagugaggccaguguggu  151   0  seq
```

miRBase precursor : Cte-Mir-2-o36_pre
Total read count : 4054
Cte-Mir-2-o36_3p read count3984
remaining reads : 70

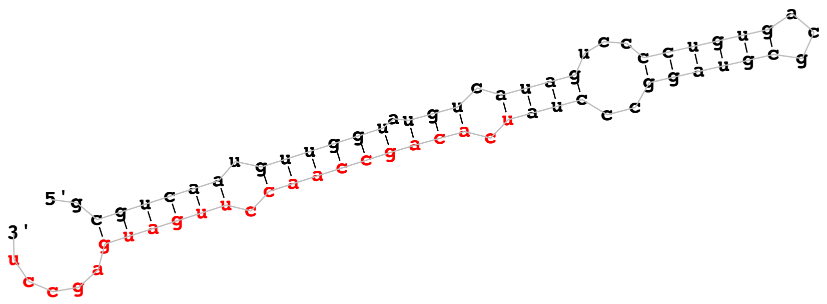

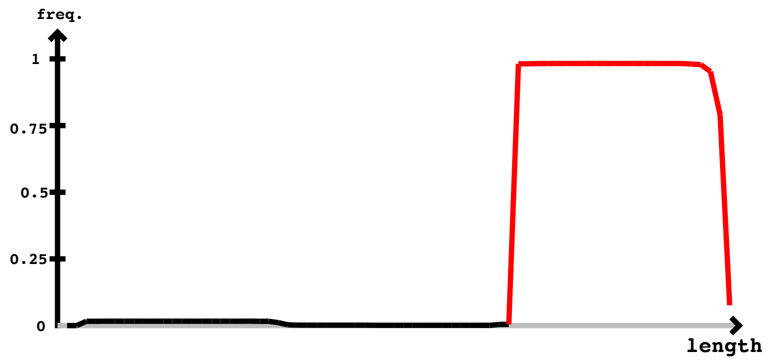


```
                                              Cte-Mir-2-o36_3p
5'- gcgucaauguugguaugucauaguccccugugacgcguaggcccuaucacagccaaccuugaugagccu  -3'    exp
    .((((((.((((((..(((((((....)))))))((....)).........)))))).)))))).....    reads    mm    sample
    .cgucaauguugguauguc..................................................    2        0     seq
    .cgucaauguugguaugucau................................................    23       0     seq
    .cgucaauguugguaugucaua...............................................    31       0     seq
    .cgCcaauguugguaugucaua...............................................    1        1     seq
    .cgucaauguugguaugAcaua...............................................    1        1     seq
    .cgucaauguugguaugucauaU..............................................    4        1     seq
    .cgucaauguugguaugucauagu.............................................    1        0     seq
    .cgucaauguugguaugucauaguccccugu......................................    2        0     seq
    ...ucaauguugguaugucauagu.............................................    1        0     seq
    ..................cauaguccccugugacgcguaggcc..........................    1        0     seq
    .....................aguccccugugacgcguaggccc.........................    1        0     seq
    .....................aguccccuguAacgcguaggccc.........................    1        1     seq
    ........................ccccugugacgcguaggcccu........................    1        0     seq
    ............................................uaucacagccaaccuugaug.....    5        0     seq
    ............................................uaucacagccaaccuugauga....    4        0     seq
    ............................................uaucacagccaaccuugaugag...    1        0     seq
    ............................................uaucacagccaaccuugaugagU..    3        1     seq
    ............................................uaucacagccaaccuugaugagc..    3        0     seq
    .............................................Cucacagccaaccuugaugagcc.    3        1     seq
    ..............................................ucacagccaaccuugaug.....    4        0     seq
    ..............................................ucacagccaaccuugauga....    4        0     seq
    ..............................................ucacagccaacAuugaugag...    1        1     seq
    ..............................................ucacagccaaccuugaugag...    103      0     seq
    ..............................................ucacagccaaccAugaugag...    1        1     seq
    ..............................................ucacagccaaccuugaCgagc..    1        1     seq
    ..............................................ucacagccaaccuugaugagU..    2        1     seq
    ..............................................ucacagcUaaccuugaugagc..    1        1     seq
    ..............................................ucacagccaaccuugaAgagc..    1        1     seq
    ..............................................ucacagccaaccuugaugagA..    3        1     seq
    ..............................................ucacagccaaccAugaugagc..    2        1     seq
    ..............................................Acacagccaaccuugaugagc..    6        1     seq
    ..............................................ucacagccaaccuugaugagc..    628      0     seq
    ..............................................ucacagccaaccuugaugUgc..    1        1     seq
    ..............................................ucacagccaaccuCgaugagc..    1        1     seq
    ..............................................ucacagccaaccuugauAagc..    1        1     seq
```

Cte-Mir-2-o36_3p

```
gcgucaauguugguaugucauaguccccugugacgcguaggcccuaucacagccaaccuugaugagccu
..............................................ucacagccaaccuCgaugagcc.    1      1      seq
..............................................ucacagcAaaccuugaugagcc.    3      1      seq
..............................................ucacagccaUccuugaugagcc.    1      1      seq
..............................................ucacagccaaccuugaugagcc.    2809   0      seq
..............................................ucacaUccaaccuugaugagcc.    1      1      seq
..............................................ucacagccaaccuAgaugagcc.    2      1      seq
..............................................uAacagccaaccuugaugagcc.    1      1      seq
..............................................ucacagccaaccuuAaugagcc.    2      1      seq
..............................................ucacagccGaccuugaugagcc.    2      1      seq
..............................................ucacagGcaaccuugaugagcc.    2      1      seq
..............................................ucacagccaacUuugaugagcc.    1      1      seq
..............................................ucGcagccaaccuugaugagcc.    3      1      seq
..............................................ucacaCccaaccuugaugagcc.    1      1      seq
..............................................uUacagccaaccuugaugagcc.    1      1      seq
..............................................ucacagccaaccGugaugagcc.    1      1      seq
..............................................Gcacagccaaccuugaugagcc.    2      1      seq
..............................................ucacagccaaccuugauAagcc.    8      1      seq
..............................................ucacagccaaccuugaugagcU.    8      1      seq
..............................................ucacagccaacAuugaugagcc.    2      1      seq
..............................................ucacagccaGccuugaugagcc.    2      1      seq
..............................................ucacagccaaccuugaugagcA.    5      1      seq
..............................................ucacagcUaaccuugaugagcc.    1      1      seq
..............................................ucacagccaaccuugaAgagcc.    3      1      seq
..............................................Ccacagccaaccuugaugagcc.    1      1      seq
..............................................ucacGgccaaccuugaugagcc.    1      1      seq
..............................................ucacagccaaccuugaugagAc.    2      1      seq
..............................................ucacagccaaccuuCaugagcc.    2      1      seq
..............................................Acacagccaaccuugaugagcc.    19     1      seq
..............................................ucacagccaaccuugUugagcc.    1      1      seq
..............................................ucacagAcaaccuugaugagcc.    5      1      seq
..............................................ucacaUccaaccuugaugagccu    1      1      seq
..............................................ucacagccaaccuugaugagccu    253    0      seq
..............................................ucacagccaaccuugaugagccC    1      1      seq
..............................................ucacagccaaccuugauAagccu    2      1      seq
..............................................Acacagccaaccuugaugagccu    1      1      seq
..............................................ucacagccaaccuugaugagccA    40     1      seq
..............................................ucacagAcaaccuugaugagccu    2      1      seq
..............................................ucacagccaaccuuAaugagccu    1      1      seq
..............................................ucacagccaaccuugaugagccG    6      1      seq
................................................acagccaaccuugaugagc..    1      0      seq
................................................Ucagccaaccuugaugagc..    1      1      seq
................................................acagccaaccuugaugagccu    2      0      seq
..................................................agccaaccuugaugagcc.    1      0      seq
```

miRBase precursor : Cte-Mir-33_pre
Total read count : 771
Cte-Mir-33_5p read count : 752
remaining reads : 19

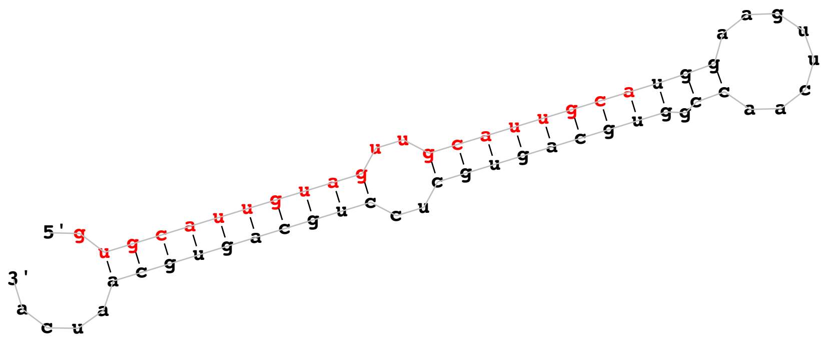

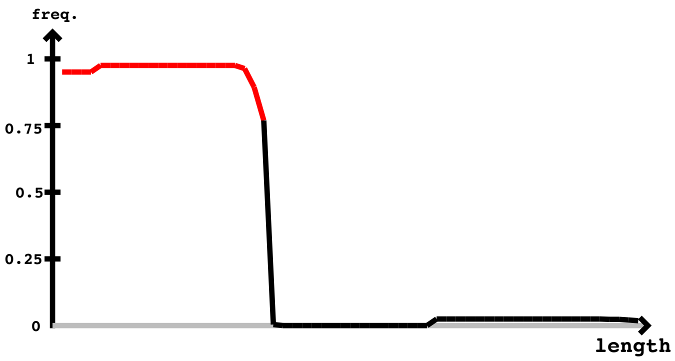

Cte-Mir-33_5p

```
5'- gugcauuguaguugcauugcauggaaguucaaccggugcagugcuccugcagugcaauca   -3'    exp
    .((((((((((..(((((((((((........)).)))))))))..))))))))))....    reads      mm         sample
    gugcauuguaguugcauu..........................................    9          0          seq
    gugcauuguaguugcauug.........................................    54         0          seq
    gugcauuguaguugcauugc........................................    94         0          seq
    gugUauuguaguugcauugc........................................    1          1          seq
    gugcauuguaguugcauugA........................................    1          1          seq
    gugGauuguaguugcauugca.......................................    1          1          seq
    gugcauuguagAugcauugca.......................................    2          1          seq
    gugcauuguaguugcauCgca.......................................    1          1          seq
    gugcauuguaguugcauugca.......................................    557        0          seq
    gugAauuguaguugcauugca.......................................    1          1          seq
    gugUauuguaguugcauugca.......................................    5          1          seq
    gugcauuguaguugcauugAa.......................................    2          1          seq
    gugcauugAaguugcauugca.......................................    1          1          seq
    gugcauuguUguugcauugca.......................................    1          1          seq
    gugcauuguaguugcauugcaA......................................    3          1          seq
    ...cauuguaguugcauugca.......................................    19         0          seq
    ......................................cagugcuccugcagugca....    1          0          seq
    ......................................cagugcuccugcagugcaau..    2          0          seq
    ......................................cagugcuccugcagugcaauc.    2          0          seq
    ......................................cagugcuccugcagugcaauca    14         0          seq
```
