# Supplementary material for: The Small RNA Universe of Capitella teleta
Source: Front Mol Biosci. 2022 Feb 25;9:802814. doi: 10.3389/fmolb.2022.802814 (PMC8915122; doi:10.3389/fmolb.2022.802814)
Supplement: Supplementary file 1 [file DataSheet1.ZIP › Supplement/SupFile8_candidate.html]

miRDeep2
| miRDeep2 version | 2.0.1.3 |
  
  

## Candidate novel miRNAs

  


provisional idthis is a provisional miRNA name assigned by miRDeep2. The first part of the id designates the chromosome or genome contig on which the miRNA gene is located. The second part is a running number that is added to avoid identical ids. The running number is incremented by one for each potential miRNA precursor that is excised from the genome. Clicking this field will display a pdf of the structure, read signature and score breakdown of the reported miRNA. | miRDeep2 scorethe log-odds score assigned to the hairpin by miRDeep2 | estimated probability that the miRNA candidate is a true positivethe estimated probability that a predicted novel miRNA with a score of this or higher is a true positive. To see exactly how this probability is estimated, mouse over the 'novel miRNAs, true positives' in the table at the top of the webpage. | rfam alertthis field indicates if the predicted miRNA hairpin has sequence similarity to reference rRNAs or tRNAs. Warnings in this field should overrule the estimated probability that a reported miRNA is a true positive (previous field). | total read countthis is the sum of read counts for the predicted mature, loop and star miRNAs. | mature read countthis is the number of reads that map to the predicted miRNA hairpin and are contained in the sequence covered by the predicted mature miRNA, including 2 nts upstream and 5 nts downstream. | loop read countthis is the number of reads that map to the predicted miRNA hairpin and are contained in the sequence covered by the predicted miRNA loop, including 2 nts upstream and 5 nts downstream. | star read countthis is the number of reads that map to the predicted miRNA hairpin and are contained in the sequence covered by the predicted star miRNA, including 2 nts upstream and 5 nts downstream. | significant randfold p-valuethis field indicates if the estimated randfold p-value of the excised potential miRNA hairpin is equal to or lower than 0.05 (see Bonnet et al., Bioinformatics, 2004). | MirGeneDB miRNAthis field displays the ids of any reference mature miRNAs for the species that map perfectly (full length, no mismatches) to the reported miRNA hairpin. If this is the case, the reported miRNA hairpin is assigned as a known miRNA. If not, it is assigned as a novel miRNA. If more than one reference mature miRNA maps to the miRNA hairpin, then only the id of the reference miRBase miRNA that matches the predicted mature sequence is output. | example MirGeneDB miRNA with the same seedthis field displays the ids of any reference mature miRNAs from related species that have a seed sequence identical to that of the reported mature miRNA. The seed is here defined as nucleotides 2-8 from the 5' end of the mature miRNA. If more than one reference mature miRNA have identical seed, then only the id of the miRNA that occurs last in the input file of reference mature miRNAs from related species is displayed. | UCSC browserif a species name was input to miRDeep2, then clicking this field will initiate a UCSC blat search of the consensus precursor sequence against the reference genome. | NCBI blastnclicking this field will initiate a NCBI blastn search of the consensus precursor sequence against the nr/nt database (non-redundant collection of all NCBI nucleotide sequences). | consensus mature sequencethis is the consensus mature miRNA sequence as inferred from the deep sequencing reads. | consensus star sequencethis is the consensus star miRNA sequence as inferred from the deep sequencing reads. | consensus precursor sequencethis is the consensus precursor miRNA sequence as inferred from the deep sequencing reads. Note that this is the inferred Drosha hairpin product, and therefore does not include substantial flanking genomic sequence as does most miRBase precursors. | precursor coordinateThe given precursor coordinates refer do absolute position in the mapped reference sequence || CAPTEscaffold\_488\_22707 | 2.0e+4 | 0.87 ± 0.03 |  | 40572 | 40568 | 0 | 4 | yes |  |  |  | blast | uaagcauuacgaguacugguag | cccgguacuugggcugcuuau | cccgguacuugggcugcuuaugcaguuuuaaagcucauaagcauuacgaguacugguag | CAPTEscaffold\_488:126121..126180:+ |--
| CAPTEscaffold\_394\_20722 | 9.5e+3 | 0.87 ± 0.03 |  | 18657 | 18640 | 0 | 17 | no |  |  |  | blast | cuagucaggaugaugcaucccu | aggugcaucaccauggcuacgg | aggugcaucaccauggcuacggugcguuaugaucaggccuagucaggaugaugcaucccu | CAPTEscaffold\_394:5237..5297:- |
| CAPTEscaffold\_12304\_44295 | 9.5e+3 | 0.87 ± 0.03 |  | 18657 | 18640 | 0 | 17 | no |  |  |  | blast | cuagucaggaugaugcaucccu | aggugcaucaccauggcuacgg | aggugcaucaccauggcuacggugcguuaugaucaggccuagucaggaugaugcaucccu | CAPTEscaffold\_12304:225..285:+ |--
| CAPTEscaffold\_324\_18348 | 5.8e+3 | 0.87 ± 0.03 |  | 11508 | 11481 | 4 | 23 | yes |  |  |  | blast | uaagcauugauaguacagggau | uccguguacugcaugugcuuuug | uccguguacugcaugugcuuuuguaaaggaccauucgaucauaagcauugauaguacagggau | CAPTEscaffold\_324:170405..170468:- |--
| CAPTEscaffold\_7256\_41364 | 5.5e+3 | 0.87 ± 0.03 |  | 10977 | 10960 | 0 | 17 | yes |  |  |  | blast | uaagcauuguuaguacagggau | cuguguacugcaugugcuuuug | cuguguacugcaugugcuuuugugaugaaccauuugaucauaagcauuguuaguacagggau | CAPTEscaffold\_7256:6311..6373:- |
| CAPTEscaffold\_1308\_33676 | 5.5e+3 | 0.87 ± 0.03 |  | 10909 | 10908 | 0 | 1 | yes |  |  |  | blast | agaagauuuaauaagauugggg | ccaauuuuauuaaauauucuag | ccaauuuuauuaaauauucuaggugcuuugaaacaauagaagauuuaauaagauugggg | CAPTEscaffold\_1308:9125..9184:- |--
| CAPTEscaffold\_488\_22705 | 3.6e+3 | 0.87 ± 0.03 |  | 7114 | 6948 | 0 | 166 | yes |  |  |  | blast | uaagcacuaagaguacugguag | uccugguacucuggcugcuugu | uccugguacucuggcugcuugugcuguuuuaaagcucauaagcacuaagaguacugguag | CAPTEscaffold\_488:125853..125913:+ |--
| CAPTEscaffold\_17\_2729 | 1.6e+3 | 0.87 ± 0.03 |  | 3171 | 3164 | 2 | 5 | yes |  |  |  | blast | augaugucacaauugacugca | cagucagugaugacaucauu | augaugucacaauugacugcaguugauguugaguccuuuggugaugcagucagugaugacaucauu | CAPTEscaffold\_17:755876..755942:- |--
| CAPTEscaffold\_1401\_33836 | 1.1e+3 | 0.87 ± 0.03 |  | 2293 | 2285 | 0 | 8 | no |  |  |  | blast | aaagcuuuaucgauugagaugguu | cuacucaucuauaugguauucu | aaagcuuuaucgauugagaugguuguguucuuuauugauucaacuacucaucuauaugguauucu | CAPTEscaffold\_1401:15993..16058:+ |
| CAPTEscaffold\_1755\_32145 | 1.1e+3 | 0.87 ± 0.03 |  | 2286 | 2278 | 0 | 8 | no |  |  |  | blast | aaagcuauaucgauugaaaugguu | cuacucaucuauaugguauucu | aaagcuauaucgauugaaaugguuguguucuuuauugauucaacuacucaucuauaugguauucu | CAPTEscaffold\_1755:9490..9555:- |
| CAPTEscaffold\_9984\_43267 | 1.1e+3 | 0.87 ± 0.03 |  | 2286 | 2278 | 0 | 8 | no |  |  |  | blast | aaagcuauaucgauugaaaugguu | cuacucaucuauaugguauucu | aaagcuauaucgauugaaaugguuguguucuuuauugauucaacuacucaucuauaugguauucu | CAPTEscaffold\_9984:412..477:- |
| CAPTEscaffold\_9804\_43110 | 1.1e+3 | 0.87 ± 0.03 |  | 2286 | 2278 | 0 | 8 | no |  |  |  | blast | aaagcuauaucgauugaaaugguu | cuacucaucuauaugguauucu | aaagcuauaucgauugaaaugguuguguucuuuauugauucaacuacucaucuauaugguauucu | CAPTEscaffold\_9804:955..1020:+ |--
| CAPTEscaffold\_234\_15402 | 1.1e+3 | 0.87 ± 0.03 |  | 2197 | 1904 | 0 | 293 | yes |  |  |  | blast | auugguguaauuaugaugucuucg | uaaggcaucguuuacaccacgu | auugguguaauuaugaugucuucguacguaaauacaucuaaggcaucguuuacaccacgu | CAPTEscaffold\_234:20072..20132:+ |--
| CAPTEscaffold\_530\_24266 | 9.7e+2 | 0.87 ± 0.03 |  | 1905 | 1892 | 0 | 13 | yes |  |  |  | blast | acuccuuucggcuggcuccugu | aggagccagccgaaaggagugg | acuccuuucggcuggcuccugugucgcuucagccuuccaagcgacgcaaggagccagccgaaaggagugg | CAPTEscaffold\_530:26802..26872:+ |--
| CAPTEscaffold\_234\_15398 | 8.0e+2 | 0.87 ± 0.03 |  | 1578 | 1570 | 0 | 8 | no |  |  |  | blast | agguuuugugagauguucauug | augaucauccuacaaaagcca | agguuuugugagauguucauuggauaagcacgcgccaaugaucauccuacaaaagcca | CAPTEscaffold\_234:18734..18792:+ |--
| CAPTEscaffold\_60\_5425 | 4.6e+2 | 0.87 ± 0.03 |  | 905 | 807 | 0 | 98 | yes |  |  |  | blast | uaagccccuuugguauaagaag | uucuugaaccucgggugcuugu | uucuugaaccucgggugcuuguguuucaauugcaucauaagccccuuugguauaagaag | CAPTEscaffold\_60:19777..19836:+ |
| CAPTEscaffold\_877\_30112 | 4.5e+2 | 0.87 ± 0.03 |  | 920 | 912 | 0 | 8 | no |  |  |  | blast | augccugucaugcucuuuguuu | acucugagcugagcagucuuca | acucugagcugagcagucuucaugcaacucuaaucgugaugccugucaugcucuuuguuu | CAPTEscaffold\_877:6375..6435:- |--
| CAPTEscaffold\_168\_12407 | 4.3e+2 | 0.87 ± 0.03 |  | 846 | 805 | 0 | 41 | yes |  |  |  | blast | uucccucggacagaaacucuga | ucgagcuucgucagagagacg | ucgagcuucgucagagagacgaggacgcaauggcagcccuuuucccucggacagaaacucuga | CAPTEscaffold\_168:42411..42474:+ |--
| CAPTEscaffold\_94\_8175 | 4.0e+2 | 0.87 ± 0.03 |  | 801 | 799 | 0 | 2 | yes |  |  |  | blast | caccauuucuaggacuacguuu | agguaguccuaggaauggugg | agguaguccuaggaauggugguucuacagaccaccauuucuaggacuacguuu | CAPTEscaffold\_94:270320..270373:+ |
| CAPTEscaffold\_69\_6469 | 3.9e+2 | 0.87 ± 0.03 |  | 779 | 773 | 0 | 6 | yes |  |  |  | blast | aaucaauaggacaauugacugcu | cuuucaauuuccuguugguu | aaucaauaggacaauugacugcuuaucauuuuucucagcuuucaauuuccuguugguu | CAPTEscaffold\_69:404369..404427:- |--
| CAPTEscaffold\_55\_4683 | 3.6e+2 | 0.87 ± 0.03 |  | 711 | 702 | 0 | 9 | yes |  |  |  | blast | gccacuuugucagagucgagccu | gcucgacugcgacaaaggcca | gccacuuugucagagucgagccugcggugacacauugcaaugggcucgacugcgacaaaggcca | CAPTEscaffold\_55:341808..341872:- |
| CAPTEscaffold\_563\_23774 | 3.5e+2 | 0.87 ± 0.03 |  | 702 | 559 | 0 | 143 | yes |  |  |  | blast | uucuagaacuuugccaagaacu | guauuggcacugguucuagaaca | guauuggcacugguucuagaacauccccaagaacuacaacuacaccugggaaugguauuggcacugguucuagaacuuugccaagaacu | CAPTEscaffold\_563:100472..100561:- |--
| CAPTEscaffold\_973\_30395 | 2.4e+2 | 0.87 ± 0.03 |  | 471 | 449 | 0 | 22 | yes |  |  |  | blast | uccgaggagcugagaaaagua | uuuucucagcuuccucggauga | uccgaggagcugagaaaaguaccauuuauaaaugguacuuuucucagcuuccucggauga | CAPTEscaffold\_973:20676..20736:- |--
| CAPTEscaffold\_6\_914 | 2.2e+2 | 0.87 ± 0.03 |  | 444 | 440 | 0 | 4 | yes |  |  |  | blast | uagaacuggaugguuucuggu | caggaguuauucaguucuggc | caggaguuauucaguucuggcauaccauauccaaauuaugaugcuagaacuggaugguuucuggu | CAPTEscaffold\_6:831316..831381:- |
| CAPTEscaffold\_251\_15906 | 2.2e+2 | 0.87 ± 0.03 |  | 448 | 447 | 0 | 1 | no |  |  |  | blast | ucgggugucccuguaggugggcc | uccccuacugguccccuccuc | ucgggugucccuguaggugggcccaugaaaagguccccuacugguccccuccuc | CAPTEscaffold\_251:215738..215792:+ |--
| CAPTEscaffold\_377\_19163 | 1.9e+2 | 0.87 ± 0.03 |  | 390 | 386 | 0 | 4 | yes |  |  |  | blast | ucucccguugcucgauauucc | aauauugaacuuucgggaagca | ucucccguugcucgauauuccaugcaggaauauugaacuuucgggaagca | CAPTEscaffold\_377:186350..186400:- |--
| CAPTEscaffold\_6\_893 | 1.6e+2 | 0.87 ± 0.03 |  | 316 | 312 | 0 | 4 | yes |  |  |  | blast | uagcacuggaugguuucuggu | caugagugauucaguucuggc | caugagugauucaguucuggcauaccauauccaaauuauggugcuagcacuggaugguuucuggu | CAPTEscaffold\_6:826956..827021:- |--
| CAPTEscaffold\_324\_18360 | 1.1e+2 | 0.87 ± 0.03 |  | 225 | 210 | 0 | 15 | yes |  |  |  | blast | ucuuggaccucgggugcuugu | uaagccccuuugguaugagaaa | ucuuggaccucgggugcuuguguuucaauugcaucauaagccccuuugguaugagaaa | CAPTEscaffold\_324:173382..173440:- |--
| CAPTEscaffold\_60\_5428 | 1.0e+2 | 0.87 ± 0.03 |  | 212 | 206 | 0 | 6 | yes |  |  |  | blast | uagcacacaauguacuucccg | ggauuuacauuuugagcuca | ggauuuacauuuugagcucaugacaccaagcauuagcacacaauguacuucccg | CAPTEscaffold\_60:20441..20495:+ |
| CAPTEscaffold\_324\_18310 | 1.0e+2 | 0.87 ± 0.03 |  | 212 | 206 | 0 | 6 | yes |  |  |  | blast | uagcacacaauguacuucccg | ggauuuacauuuugagcuca | ggauuuacauuuugagcucaugacaccaagcauuagcacacaauguacuucccg | CAPTEscaffold\_324:218550..218604:+ |
| CAPTEscaffold\_60\_5416 | 1.0e+2 | 0.87 ± 0.03 |  | 212 | 206 | 0 | 6 | yes |  |  |  | blast | uagcacacaauguacuucccg | ggauuuacauuuugagcuca | ggauuuacauuuugagcucaugacaccaagcauuagcacacaauguacuucccg | CAPTEscaffold\_60:18292..18346:+ |--
| CAPTEscaffold\_60\_5424 | 1.0e+2 | 0.87 ± 0.03 |  | 208 | 202 | 0 | 6 | yes |  |  |  | blast | uagcacacaauguacuucccg | ggauuuacauuuugagcuca | ggauuuacauuuugagcucaugacaccaagcauuagcacacaauguacuucccg | CAPTEscaffold\_60:19360..19414:+ |--
| CAPTEscaffold\_757\_28366 | 1.0e+2 | 0.87 ± 0.03 |  | 204 | 202 | 0 | 2 | no |  |  |  | blast | ccagagggcguuggcagugagg | ucgcugugacggcccuucugca | ccagagggcguuggcagugagggcucgaucaccaaacucgcugugacggcccuucugca | CAPTEscaffold\_757:7907..7966:+ |
| CAPTEscaffold\_96\_9145 | 9.9e+1 | 0.87 ± 0.03 |  | 193 | 181 | 0 | 12 | yes |  |  |  | blast | uggcaucugacuugagacaauuc | uugucuccagucagauguccaua | uugucuccagucagauguccauauugcuucguccuaguggcaucugacuugagacaauuc | CAPTEscaffold\_96:283300..283360:+ |--
| CAPTEscaffold\_264\_14680 | 8.8e+1 | 0.87 ± 0.03 |  | 173 | 171 | 0 | 2 | yes |  |  |  | blast | uuggcacuguguccgugagcgu | cgcgcacugacgcugucgucaag | cgcgcacugacgcugucgucaagaaacaugcaaaagcuauuugagcuuggcacuguguccgugagcgu | CAPTEscaffold\_264:192009..192077:- |
| CAPTEscaffold\_292\_14542 | 8.4e+1 | 0.87 ± 0.03 |  | 165 | 128 | 0 | 37 | yes |  |  |  | blast | caccuguugugcgggguauuacu | auuuaccccguauuauagg | auuuaccccguauuauagguauuacauaacaccuguugugcgggguauuacu | CAPTEscaffold\_292:163856..163908:- |--
| CAPTEscaffold\_32\_3388 | 7.8e+1 | 0.87 ± 0.03 |  | 152 | 135 | 0 | 17 | yes |  |  |  | blast | aagcacuguauacauguauuucu | aacauuuuucugcagugcucag | aagcacuguauacauguauuucugccguacuuaaggauuucagaaacauuuuucugcagugcucag | CAPTEscaffold\_32:517845..517911:+ |
| CAPTEscaffold\_795\_28880 | 7.1e+1 | 0.87 ± 0.03 |  | 137 | 129 | 0 | 8 | yes |  |  |  | blast | ugaucuaugugaccuaugaccu | gucauaggucgcauaggucaca | ugaucuaugugaccuaugaccuaugcauguaggucauaggucgcauaggucaca | CAPTEscaffold\_795:18362..18416:+ |--
| CAPTEscaffold\_146\_11393 | 6.3e+1 | 0.87 ± 0.03 |  | 123 | 115 | 0 | 8 | yes |  |  |  | blast | agcgaugaagauaaggacacac | gugccauuaucuucaucgcugc | agcgaugaagauaaggacacaccguaaacaugcaugaugagugugccauuaucuucaucgcugc | CAPTEscaffold\_146:6886..6950:- |
| CAPTEscaffold\_44\_4619 | 5.6e+1 | 0.87 ± 0.03 |  | 108 | 107 | 0 | 1 | yes |  |  |  | blast | uuugugaucuuguguaauacu | auuacacaagaccacaaaacc | uuugugaucuuguguaauacugguaucauacaguauuacacaagaccacaaaacc | CAPTEscaffold\_44:534613..534668:- |--
| CAPTEscaffold\_189\_13539 | 4.6e+1 | 0.87 ± 0.03 |  | 90 | 88 | 0 | 2 | yes |  |  |  | blast | acaugcaugccgugucuccauc | uggcgccacggcaugcuucgucu | acaugcaugccgugucuccaucucucguggcuuaauugcggguggcgccacggcaugcuucgucu | CAPTEscaffold\_189:103030..103095:- |--
| CAPTEscaffold\_24\_2918 | 4.3e+1 | 0.87 ± 0.03 |  | 83 | 81 | 0 | 2 | yes |  |  |  | blast | aggauuuuagcaacacccgaga | cuaggguguugcuaaaaaccuu | cuaggguguugcuaaaaaccuuccgaaacuuauuaggucucauuaagauuuggaaggauuuuagcaacacccgaga | CAPTEscaffold\_24:74834..74910:- |--
| CAPTEscaffold\_281\_15882 | 4.0e+1 | 0.87 ± 0.03 |  | 88 | 87 | 0 | 1 | no |  |  |  | blast | cuggacgcgggugacuaccucu | aaccagucagccggucucagg | aaccagucagccggucucagggucauugacccccuggacgcgggugacuaccucu | CAPTEscaffold\_281:77120..77175:- |--
| CAPTEscaffold\_958\_29875 | 3.2e+1 | 0.87 ± 0.03 |  | 64 | 35 | 0 | 29 | yes |  |  |  | blast | agcaaauauuuuucgucggcgc | aucgacgaucaauacuugcca | aucgacgaucaauacuugccacaaaucagcaaauauuuuucgucggcgc | CAPTEscaffold\_958:53172..53221:- |
| CAPTEscaffold\_755\_28348 | 2.9e+1 | 0.87 ± 0.03 |  | 55 | 54 | 0 | 1 | yes |  |  |  | blast | uuacgaacuaucagcaauacugu | guauggcugauaguucguaagc | guauggcugauaguucguaagcaugcacguaguucaaugaggaaucauguucaugcuuacgaacuaucagcaauacugu | CAPTEscaffold\_755:56869..56948:- |
| CAPTEscaffold\_123\_10154 | 2.8e+1 | 0.87 ± 0.03 |  | 64 | 63 | 0 | 1 | no |  |  |  | blast | aagcaaucccggccagagugac | ucauucugucuugggucgcauucu | ucauucugucuugggucgcauucuggaucaguaucgaagcaaucccggccagagugac | CAPTEscaffold\_123:12956..13014:- |--
| CAPTEscaffold\_2\_177 | 2.8e+1 | 0.87 ± 0.03 |  | 57 | 50 | 0 | 7 | yes |  |  |  | blast | ugcacucgcuagagcccacacaccu | cuguguggguguagauucuugugccaca | ugcacucgcuagagcccacacaccuuuuaaugcuguguggguguagauucuugugccaca | CAPTEscaffold\_2:301375..301435:- |
| CAPTEscaffold\_1153\_32192 | 2.7e+1 | 0.87 ± 0.03 |  | 53 | 52 | 0 | 1 | yes |  |  |  | blast | cacagauuguuccaucucucagu | ugagagagggaacaauuugugu | ugagagagggaacaauuuguguacuugucaaguacacagauuguuccaucucucagu | CAPTEscaffold\_1153:10027..10084:- |--
| CAPTEscaffold\_20545\_46059 | 2.4e+1 | 0.87 ± 0.03 |  | 57 | 50 | 0 | 7 | no |  |  |  | blast | ugcacucgcuagagcccacacaccu | cuguguggguguagauucuugugccaca | ugcacucgcuagagcccacacaccuuuuaaugcuguguggguguagauucuugugccaca | CAPTEscaffold\_20545:210..270:- |
| CAPTEscaffold\_377\_19161 | 2.4e+1 | 0.87 ± 0.03 |  | 47 | 46 | 0 | 1 | yes |  |  |  | blast | ugugagaacaccacuugagcucug | gaggucaguggagcuccacguga | ugugagaacaccacuugagcucuguauccaaucagaggucaguggagcuccacguga | CAPTEscaffold\_377:177544..177601:- |--
| CAPTEscaffold\_35\_3565 | 2.2e+1 | 0.87 ± 0.03 |  | 42 | 29 | 0 | 13 | yes |  |  |  | blast | ucuaccugagaauauccacauu | guagauuuucucagguagacc | guagauuuucucagguagaccguguagacgguagacggucuaccugagaauauccacauu | CAPTEscaffold\_35:182585..182645:+ |--
| CAPTEscaffold\_475\_21078 | 2.0e+1 | 0.87 ± 0.03 |  | 37 | 32 | 0 | 5 | yes |  |  |  | blast | uuugcuagaaaccuuccgaaacu | uuucggaagguuuuagcaccacc | uuugcuagaaaccuuccgaaacuuauugagaccuaauaaguuucggaagguuuuagcaccacc | CAPTEscaffold\_475:12876..12939:+ |
| CAPTEscaffold\_51\_5558 | 2.0e+1 | 0.87 ± 0.03 |  | 37 | 32 | 0 | 5 | yes |  |  |  | blast | uuugcuagaaaccuuccgaaacu | uuucggaagguuuuagcaccacc | uuugcuagaaaccuuccgaaacuuauugagaccuaauaaguuucggaagguuuuagcaccacc | CAPTEscaffold\_51:566613..566676:+ |
| CAPTEscaffold\_1377\_33899 | 1.9e+1 | 0.87 ± 0.03 |  | 36 | 35 | 0 | 1 | yes |  |  |  | blast | uuuuguaacugcauaauucuacu | uaaauauucagcuacagaauc | uuuuguaacugcauaauucuacucggcuuugccucggaguaaauauucagcuacagaauc | CAPTEscaffold\_1377:23530..23590:- |
| CAPTEscaffold\_2218\_35830 | 1.8e+1 | 0.87 ± 0.03 |  | 51 | 29 | 0 | 22 | no |  |  |  | blast | gugaguuugauuuuucugaauu | uucggaauuuuuuuuaaacuuc | gugaguuugauuuuucugaauuaggauuuguaauuaauucggaauuuuuuuuaaacuuc | CAPTEscaffold\_2218:7363..7422:+ |
| CAPTEscaffold\_4928\_39836 | 1.8e+1 | 0.87 ± 0.03 |  | 34 | 19 | 0 | 15 | yes |  |  |  | blast | gacuuucauggucaggaucaua | ugaucaugaugguggcaguca | ugaucaugaugguggcagucacccagugcagucggucauaauacaugaaugggggacuuucauggucaggaucaua | CAPTEscaffold\_4928:164..240:- |
| CAPTEscaffold\_281\_15857 | 1.8e+1 | 0.87 ± 0.03 |  | 34 | 19 | 0 | 15 | yes |  |  |  | blast | gacuuucauggucaggaucaua | ugaucaugaugguggcaguca | ugaucaugaugguggcagucacccagugcagucggucauaauacaugaaugggggacuuucauggucaggaucaua | CAPTEscaffold\_281:1081..1157:+ |
| CAPTEscaffold\_276\_16091 | 1.7e+1 | 0.87 ± 0.03 |  | 34 | 19 | 0 | 15 | yes |  |  |  | blast | gacuuucauggucaggaucaua | ugaucaugaugguggcaguca | ugaucaugaugguggcagucacccagugcagucggucauaauacaugaaugggggacuuucauggucaggaucaua | CAPTEscaffold\_276:79587..79663:+ |--
| CAPTEscaffold\_137\_8834 | 1.7e+1 | 0.87 ± 0.03 |  | 34 | 33 | 0 | 1 | yes |  |  |  | blast | ugaauuaugaauucccugccaga | gugagcggggaagaguaauuauc | gugagcggggaagaguaauuaucauaugauuaauuaugaauuaugaauucccugccaga | CAPTEscaffold\_137:353803..353862:- |--
| CAPTEscaffold\_815\_29074 | 1.4e+1 | 0.87 ± 0.03 |  | 26 | 15 | 0 | 11 | yes |  |  |  | blast | auauacauguauagcucagaau | ucugagcuauacauguauuaucu | ucugagcuauacauguauuaucuagucacuagauauacauguauagcucagaau | CAPTEscaffold\_815:15754..15808:- |--
| CAPTEscaffold\_394\_20707 | 1.4e+1 | 0.87 ± 0.03 |  | 27 | 19 | 0 | 8 | yes |  |  |  | blast | ugcuuugaaacauuacuccuca | gggacaaugccucaaagcagu | gggacaaugccucaaagcagucaaaaggaacugcuuugaaacauuacuccuca | CAPTEscaffold\_394:5119..5172:+ |--
| CAPTEscaffold\_759\_23463 | 1.3e+1 | 0.87 ± 0.03 |  | 30 | 29 | 0 | 1 | yes |  |  |  | blast | auugauauucuguugacacauc | uguaucaacuaaauaucaacau | auugauauucuguugacacaucagccaaauaucauuauugauggauguaucaacuaaauaucaacau | CAPTEscaffold\_759:5264..5331:- |--
| CAPTEscaffold\_3352\_39088 | 1.3e+1 | 0.87 ± 0.03 |  | 24 | 22 | 0 | 2 | yes |  |  |  | blast | aucguucuugguaacauuucca | aaauguuaccaagaacgggccca | aucguucuugguaacauuuccaucagcggaucagauggaaauguuaccaagaacgggccca | CAPTEscaffold\_3352:965..1026:+ |
| CAPTEscaffold\_107\_7101 | 1.3e+1 | 0.87 ± 0.03 |  | 24 | 15 | 0 | 9 | yes |  |  |  | blast | acagcuccuccuggugaugcug | gcaucacaaucaggagcugggu | acagcuccuccuggugaugcugcugcccugggcauccagcaucacaaucaggagcugggu | CAPTEscaffold\_107:27651..27711:- |
| CAPTEscaffold\_644\_25854 | 1.2e+1 | 0.87 ± 0.03 |  | 26 | 22 | 0 | 4 | yes |  |  |  | blast | guacgaaugcugaucacgugcu | ugcgucuucugcauucguugcu | guacgaaugcugaucacgugcucgguuccguugcgucuucugcauucguugcu | CAPTEscaffold\_644:86275..86328:+ |
| CAPTEscaffold\_1214\_32128 | 1.2e+1 | 0.87 ± 0.03 |  | 22 | 20 | 0 | 2 | yes |  |  |  | blast | cacguggcuacuggcagugcagg | gcacugccagaugccauguagg | cacguggcuacuggcagugcaggugggucaucugagccaauagccgcauuccugcacugccagaugccauguagg | CAPTEscaffold\_1214:16914..16989:+ |
| CAPTEscaffold\_328\_19497 | 1.2e+1 | 0.87 ± 0.03 |  | 23 | 21 | 0 | 2 | yes |  |  |  | blast | ucugcaaucauaacgcacccc | agugcguuaugauugcagaga | agugcguuaugauugcagagaaauaauaucugcaaucauaacgcacccc | CAPTEscaffold\_328:103473..103522:+ |
| CAPTEscaffold\_344\_18510 | 1.2e+1 | 0.87 ± 0.03 |  | 23 | 19 | 0 | 4 | yes |  |  |  | blast | uguuguuaggcugucgagccaga | ugacuugaucacuaaccgcaug | ugacuugaucacuaaccgcaugcuaauuuuccuuugcauguuguuaggcugucgagccaga | CAPTEscaffold\_344:152302..152363:- |
| CAPTEscaffold\_25769\_46663 | 1.1e+1 | 0.87 ± 0.03 |  | 22 | 15 | 0 | 7 | yes |  |  |  | blast | acacaauuggcaaaguuacauu | uguaacuuuaccaauuguguau | uguaacuuuaccaauuguguauauuaauuauacaauauacacaauuggcaaaguuacauu | CAPTEscaffold\_25769:437..497:+ |
| CAPTEscaffold\_2743\_37448 | 1.1e+1 | 0.87 ± 0.03 |  | 22 | 15 | 0 | 7 | yes |  |  |  | blast | acacaauuggcaaaguuacauu | uguaacuuuaccaauuguguau | uguaacuuuaccaauuguguauauuaauuauacaauauacacaauuggcaaaguuacauu | CAPTEscaffold\_2743:8316..8376:- |--
| CAPTEscaffold\_69\_6453 | 1.1e+1 | 0.87 ± 0.03 |  | 21 | 19 | 0 | 2 | yes |  |  |  | blast | accggagagcggguagugugga | cacucuaccgagcccucuaagcc | accggagagcggguaguguggagccgucagcccuccacucuaccgagcccucuaagcc | CAPTEscaffold\_69:241597..241655:- |--
| CAPTEscaffold\_154\_12069 | 1.1e+1 | 0.87 ± 0.03 |  | 20 | 14 | 0 | 6 | yes |  |  |  | blast | ugagcagucauagcccgcggcc | agcgggcuaugacuguucgu | agcgggcuaugacuguucgugguuaaaacaguuaugaccaugagcagucauagcccgcggcc | CAPTEscaffold\_154:17893..17955:+ |
| CAPTEscaffold\_130\_11176 | 1.0e+1 | 0.87 ± 0.03 |  | 21 | 17 | 0 | 4 | yes |  |  |  | blast | caccgcuguuuaucaccaugugc | cacgguaguaaacagcgguauc | cacgguaguaaacagcgguaucacauuaaauuugacaccgcuguuuaucaccaugugc | CAPTEscaffold\_130:25878..25936:- |--
| CAPTEscaffold\_655\_26868 | 1.0e+1 | 0.87 ± 0.03 |  | 17 | 16 | 0 | 1 | yes |  |  |  | blast | ccugucaaucugagcacuugcc | aagugcucagauugacaaguac | ccugucaaucugagcacuugccauaaaguuuauggcaagugcucagauugacaaguac | CAPTEscaffold\_655:40905..40963:+ |
| CAPTEscaffold\_452\_22339 | 1.0e+1 | 0.87 ± 0.03 |  | 16 | 8 | 0 | 8 | yes |  |  |  | blast | ucggcggcaguggauugugacg | aucacaguccacugccgccga | ucggcggcaguggauugugacgauguagcucaucacaguccacugccgccga | CAPTEscaffold\_452:122249..122301:- |
| CAPTEscaffold\_2741\_37338 | 8.9 | 0.87 ± 0.03 |  | 17 | 14 | 0 | 3 | yes |  |  |  | blast | ucccgucaauccauggaucua | gucuuuuggguucacggugauc | gucuuuuggguucacggugaucugaaucacagacuuugaucccgucaauccauggaucua | CAPTEscaffold\_2741:892..952:- |
| CAPTEscaffold\_147\_11165 | 8.7 | 0.87 ± 0.03 |  | 17 | 9 | 0 | 8 | yes |  |  |  | blast | aucgagaucuaucagaauuaccu | aaauucugacagaucucaaacug | aucgagaucuaucagaauuaccuuuugaugcaaaaagcaaauucugacagaucucaaacug | CAPTEscaffold\_147:241439..241500:- |
| CAPTEscaffold\_488\_22733 | 8.5 | 0.87 ± 0.03 |  | 25 | 13 | 0 | 12 | no |  |  |  | blast | uaagcaccgguggagugccccau | ggagccuucgauuguugcuugug | ggagccuucgauuguugcuuguguugauuaaggccauaagcaccgguggagugccccau | CAPTEscaffold\_488:129344..129403:+ |--
| CAPTEscaffold\_650\_25107 | 8.2 | 0.87 ± 0.03 |  | 15 | 13 | 0 | 2 | yes |  |  |  | blast | gaugacgucugggugacggaau | uuacgucaccaagaugccaucu | uuacgucaccaagaugccaucuauaugauauugcagaugacgucugggugacggaau | CAPTEscaffold\_650:130497..130554:+ |
| CAPTEscaffold\_329\_16948 | 8.1 | 0.87 ± 0.03 |  | 13 | 11 | 0 | 2 | yes |  |  |  | blast | caauucauugcucgaacccucc | aggguucaagcaaugaauugug | caauucauugcucgaacccuccuauaauauuggaggguucaagcaaugaauugug | CAPTEscaffold\_329:160788..160843:+ |
| CAPTEscaffold\_493\_23921 | 8.0 | 0.87 ± 0.03 |  | 15 | 9 | 0 | 6 | yes |  |  |  | blast | aucgagaucuaucagaauuaccu | uaauucugacagaucucuaacug | aucgagaucuaucagaauuaccuuuugaugcaaaaagcuaauucugacagaucucuaacug | CAPTEscaffold\_493:103264..103325:- |--
| CAPTEscaffold\_13771\_44694 | 6.0 | 0.87 ± 0.03 |  | 11 | 10 | 0 | 1 | yes |  |  |  | blast | ucgaaaaugaaagaacgcacc | ugccuucuuuuguuuucgaca | ucgaaaaugaaagaacgcaccauuaguaauggugccuucuuuuguuuucgaca | CAPTEscaffold\_13771:2129..2182:+ |
| CAPTEscaffold\_284\_15974 | 5.9 | 0.87 ± 0.02 |  | 11 | 10 | 0 | 1 | yes |  |  |  | blast | ugccuuacgacagccggaacug | agcacuggcuucguguggccgg | ugccuuacgacagccggaacugcagcucauucaacagcacuggcuucguguggccgg | CAPTEscaffold\_284:108021..108078:+ |--
| CAPTEscaffold\_769\_27752 | 5.6 | 0.87 ± 0.02 |  | 10 | 7 | 0 | 3 | yes |  |  |  | blast | uauguaagcguuugugucacacc | ugcugacugcaacgcgcuugcuugc | uauguaagcguuugugucacaccuggauuccaaccaggugcugacugcaacgcgcuugcuugc | CAPTEscaffold\_769:53917..53980:+ |--
| CAPTEscaffold\_488\_22749 | 5.4 | 0.87 ± 0.02 |  | 19 | 13 | 0 | 6 | no |  |  |  | blast | uaagcaccgguggagugccccau | ggagcuuucgauuguugcuugug | ggagcuuucgauuguugcuuguguugauuaaggccauaagcaccgguggagugccccau | CAPTEscaffold\_488:131590..131649:+ |--
| CAPTEscaffold\_78\_6772 | 5.4 | 0.87 ± 0.02 |  | 8 | 7 | 0 | 1 | yes |  |  |  | blast | auuaugaucaugguaacagaau | ucuguuaccaugaucauaauc | ucuguuaccaugaucauaaucauaaugaucauuaugauuaugaucaugguaacagaau | CAPTEscaffold\_78:235077..235135:- |--
| CAPTEscaffold\_222\_14831 | 5.2 | 0.87 ± 0.02 |  | 8 | 5 | 0 | 3 | yes |  |  |  | blast | aacgcguuugguuacuacaa | uguaguaaccaaacgcguugugu | uguaguaaccaaacgcguuguguuagcugcuaacacaacgcguuugguuacuacaa | CAPTEscaffold\_222:108046..108102:+ |--
| CAPTEscaffold\_988\_28522 | 4.3 | 0.87 ± 0.02 |  | 7 | 6 | 0 | 1 | yes |  |  |  | blast | gaggucgguugagcuuuguucu | aaugaggcugacgccagacccu | aaugaggcugacgccagacccucuuuagaugaagucagagaggucgguugagcuuuguucu | CAPTEscaffold\_988:29063..29124:- |--
| CAPTEscaffold\_361\_15273 | 3.8 | 0.84 ± 0.03 |  | 8 | 7 | 0 | 1 | yes |  |  |  | blast | uucacguauagaauuguuaau | ucagcaauuccauacgugaaau | ucagcaauuccauacgugaaauaagaauuucuauuauuauuuuucacguauagaauuguuaau | CAPTEscaffold\_361:160710..160773:+ |--
| CAPTEscaffold\_179\_12875 | 3.2 | 0.84 ± 0.03 |  | 14 | 12 | 0 | 2 | no |  |  |  | blast | ugaccggacagugcaaccugauu | aucagauuggcugucuggcauu | ugaccggacagugcaaccugauuaugucgguguuuuuaucagauuggcugucuggcauu | CAPTEscaffold\_179:132900..132959:- |--
| CAPTEscaffold\_329\_17006 | 2.3 | 0.85 ± 0.02 |  | 26 | 26 | 0 | 0 | yes |  |  |  | blast | aggguucgagcaaugaauugug | caauucauugcuugaacccucc | caauucauugcuugaacccuccaauauuauaggaggguucgagcaaugaauugug | CAPTEscaffold\_329:160786..160841:- |--
| CAPTEscaffold\_302\_17352 | 2.1 | 0.85 ± 0.02 |  | 66 | 55 | 0 | 11 | yes |  |  |  | blast | uaauuuggcuccacaaucacccga | ugauuguggagccagauaacc | ugauuguggagccagauaaccgaacucgucuugaucgguaauuuggcuccacaaucacccga | CAPTEscaffold\_302:138776..138838:- |--
| CAPTEscaffold\_25974\_46680 | 2.0 | 0.85 ± 0.02 |  | 44 | 43 | 0 | 1 | yes |  |  |  | blast | aaaggagcucgaggcacuugau | aucaagugccucgagcuccu | aucaagugccucgagcuccuauauuuuuauaaaggagcucgaggcacuugau | CAPTEscaffold\_25974:247..299:- |--
| CAPTEscaffold\_519\_23023 | 2.0 | 0.85 ± 0.02 |  | 44 | 43 | 0 | 1 | yes |  |  |  | blast | aaaggagcucgaggcacuugau | aucaagugccucgagcuccu | aucaagugccucgagcuccuauauuuuuauaaaggagcucgaggcacuugau | CAPTEscaffold\_519:138437..138489:+ |--
| CAPTEscaffold\_297\_17284 | 1.9 | 0.87 ± 0.02 |  | 272 | 271 | 0 | 1 | yes |  |  |  | blast | uacguacguacugauaauggcc | auuaucaguacguacguaug | uacguacguacugauaauggccuaacuaaggccauuaucaguacguacguaug | CAPTEscaffold\_297:147069..147122:+ |--
| CAPTEscaffold\_95\_7968 | 1.9 | 0.87 ± 0.02 |  | 19 | 17 | 0 | 2 | yes |  |  |  | blast | agagcgcaucgcugauuggcu | ccaaucagcgacgcgcucu | agagcgcaucgcugauuggcugggggucagucagagccaaucagcgacgcgcucu | CAPTEscaffold\_95:37985..38040:+ |--
| CAPTEscaffold\_35\_3577 | 1.8 | 0.87 ± 0.02 |  | 362 | 303 | 0 | 59 | yes |  |  |  | blast | ucuguggcuccgagcuccucgucu | ggggagcuucugucacugga | ggggagcuucugucacuggaccucuuggcugguucuguggcuccgagcuccucgucu | CAPTEscaffold\_35:443742..443799:+ |--
| CAPTEscaffold\_60\_5445 | 1.8 | 0.87 ± 0.02 |  | 28 | 25 | 0 | 3 | no |  |  |  | blast | uuagcacucaauguaauucgcg | gguuuuacauuuugagcucaua | gguuuuacauuuugagcucauaagacuaagcauuagcacucaauguaauucgcg | CAPTEscaffold\_60:275031..275085:+ |--
| CAPTEscaffold\_463\_22033 | 1.6 | 0.87 ± 0.02 |  | 37 | 22 | 0 | 15 | yes |  |  |  | blast | ugaaugacgucagugagccgaa | cucggcucacugacaucauuga | cucggcucacugacaucauugacaaugagugaaugacgucagugagccgaa | CAPTEscaffold\_463:120222..120273:+ |--
| CAPTEscaffold\_20012\_46009 | 1.6 | 0.87 ± 0.02 |  | 18 | 17 | 0 | 1 | yes |  |  |  | blast | uauagugaugcugauugaaugu | gcauucaaucagcaucacuau | gcauucaaucagcaucacuauaauauuauuauagugaugcugauugaaugu | CAPTEscaffold\_20012:327..378:+ |--
| CAPTEscaffold\_60\_5459 | 1.5 | 0.87 ± 0.02 |  | 6560 | 6395 | 0 | 165 | yes |  |  |  | blast | agaagcacaugauguuggccacg | cggggucaacuuugugugcuug | cggggucaacuuugugugcuuguagcuguguaugcagaagcacaugauguuggccacg | CAPTEscaffold\_60:277970..278028:+ |--
| CAPTEscaffold\_488\_22752 | 1.5 | 0.87 ± 0.02 |  | 630 | 490 | 0 | 140 | yes |  |  |  | blast | gaagcaccugugguagaguuag | ugaacucuaccauguuugcuuu | ugaacucuaccauguuugcuuuuguccgcauuaaguccagaagcaccugugguagaguuag | CAPTEscaffold\_488:131766..131827:+ |--
| CAPTEscaffold\_324\_18354 | 1.5 | 0.87 ± 0.02 |  | 6560 | 6395 | 0 | 165 | yes |  |  |  | blast | agaagcacaugauguuggccacg | cggggucaacuuugugugcuug | cggggucaacuuugugugcuuguagcuguguaugcagaagcacaugauguuggccacg | CAPTEscaffold\_324:171477..171535:- |--
| CAPTEscaffold\_46\_3553 | 1.5 | 0.87 ± 0.02 |  | 29 | 15 | 0 | 14 | yes |  |  |  | blast | uucgaagucaacugauuagcga | uaaucaguugacuucguugac | uucgaagucaacugauuagcgaugacgccaguggcuaaucaguugacuucguugac | CAPTEscaffold\_46:542942..542998:- |--
| CAPTEscaffold\_15480\_45261 | 1.5 | 0.87 ± 0.02 |  | 630 | 490 | 0 | 140 | yes |  |  |  | blast | gaagcaccugugguagaguuag | ugaacucuaccauguuugcuuu | ugaacucuaccauguuugcuuuuguccgcauuaaguccagaagcaccugugguagaguuag | CAPTEscaffold\_15480:2091..2152:- |--
| CAPTEscaffold\_65\_5264 | 1.1 | 0.87 ± 0.02 |  | 10 | 9 | 0 | 1 | no |  |  |  | blast | augucaaaagagaaccuccugc | cggacgcuccucuugaccucc | cggacgcuccucuugaccuccuguucagucagaaauggaugucaaaagagaaccuccugc | CAPTEscaffold\_65:270173..270233:- |--
| CAPTEscaffold\_488\_22736 | 1.0 | 0.87 ± 0.02 |  | 631 | 491 | 0 | 140 | yes |  |  |  | blast | gaagcaccugugguagaguuag | ugaacucuaccauguuugcuuu | ugaacucuaccauguuugcuuuuguccgcaucaccgcaucaaauccggaagcaccugugguagaguuag | CAPTEscaffold\_488:129520..129589:+ |--
| CAPTEscaffold\_409\_18892 | 1.0 | 0.87 ± 0.02 |  | 11 | 10 | 0 | 1 | yes |  |  |  | blast | ucgaaaaugaaagaacgcacc | ccuucuuucguuuucgacauua | ucgaaaaugaaagaacgcaccauuaguaauggugccuucuuucguuuucgacauua | CAPTEscaffold\_409:91750..91806:+ |--
| CAPTEscaffold\_71\_6097 | 1.0 | 0.87 ± 0.02 |  | 780 | 775 | 0 | 5 | yes |  |  |  | blast | aauuuauugugugguugcagcuu | uguuccucgcaauauauuuug | aauuuauugugugguugcagcuuuuguuaacuuugaauggcuguuccucgcaauauauuuug | CAPTEscaffold\_71:310907..310969:- |--
| CAPTEscaffold\_8946\_42599 | 1.0 | 0.87 ± 0.02 |  | 780 | 775 | 0 | 5 | yes |  |  |  | blast | aauuuauugugugguugcagcuu | uguuccucgcaauauauuuug | aauuuauugugugguugcagcuuuuguuaacuuugaauggcuguuccucgcaauauauuuug | CAPTEscaffold\_8946:3443..3505:- |--
| CAPTEscaffold\_22815\_46346 | 1.0 | 0.87 ± 0.02 |  | 11 | 10 | 0 | 1 | yes |  |  |  | blast | ucgaaaaugaaagaacgcacc | ccuucuuucguuuucgacauua | ucgaaaaugaaagaacgcaccauuaguaauggugccuucuuucguuuucgacauua | CAPTEscaffold\_22815:717..773:+ |
| CAPTEscaffold\_335\_18093 | 1.0 | 0.87 ± 0.02 |  | 961 | 562 | 1 | 398 | yes |  |  |  | blast | uaaggcaaauuccaucugcuca | ugaacagaaguaaagugccuua | ugaacagaaguaaagugccuuacaguacacaagaauaguaaggcaaauuccaucugcuca | CAPTEscaffold\_335:224484..224544:+ |--
| CAPTEscaffold\_224\_14422 | 0.8 | 0.71 ± 0.02 |  | 91 | 90 | 0 | 1 | yes |  |  |  | blast | uacggaaauaaaauuuaguuuu | cuaaauuuuauuuccguacgcu | uacggaaauaaaauuuaguuuuaauacguauuaaaacuaaauuuuauuuccguacgcu | CAPTEscaffold\_224:193706..193764:+ |--
| CAPTEscaffold\_14983\_45140 | 0.7 | 0.71 ± 0.02 |  | 81 | 62 | 0 | 19 | yes |  |  |  | blast | ucacagaaucgauucgcuaagc | agcagaaccaauucugugag | agcagaaccaauucugugagcuuauggaauauuuuagagauuccguaauaagcucacagaaucgauucgcuaagc | CAPTEscaffold\_14983:78..153:- |--
| CAPTEscaffold\_228\_13639 | 0.5 | 0.71 ± 0.02 |  | 780 | 775 | 0 | 5 | yes |  |  |  | blast | aauuuauugugugguugcagcuu | uguuccucgcaauauauuuug | aauuuauugugugguugcagcuuuuguuaacuuugaauggcuguuccucgcaauauauuuug | CAPTEscaffold\_228:321..383:- |--
| CAPTEscaffold\_41\_4379 | 0.4 | 0.71 ± 0.02 |  | 18 | 13 | 2 | 3 | no |  |  |  | blast | ggagauuugaucgugcucggcga | caccagcacagcauuucuugac | caccagcacagcauuucuugacgaauuugaagaguacuugacaagccucuuuauuagaauuucuggagauuugaucgugcucggcga | CAPTEscaffold\_41:329981..330068:+ |--
| CAPTEscaffold\_27263\_46801 | 0.3 | 0.71 ± 0.02 |  | 780 | 775 | 0 | 5 | yes |  |  |  | blast | aauuuauugugugguugcagcuu | uguuccucgcaauauauuuug | aauuuauugugugguugcagcuuuuguuaauuuugaauggcuguuccucgcaauauauuuug | CAPTEscaffold\_27263:796..858:- |
