# Supplementary material for: The Small RNA Universe of Capitella teleta
Source: Front Mol Biosci. 2022 Feb 25;9:802814. doi: 10.3389/fmolb.2022.802814 (PMC8915122; doi:10.3389/fmolb.2022.802814)
Supplement: Supplementary file 1 [file DataSheet1.ZIP › Supplement/SupFile10_mirtrace-report.html]

PHRED Score Distribution

Percentage of nucleotides with given PHRED score.

Read Length Distribution

Percentage of reads of each length.

Quality Control Statistics

Percentage of reads with given quality control status.

RNA Type

Percentage of reads of each RNA type.

miRNA Complexity

Number of detected distinct miRNA genes as function of read depth.

Contamination

Percentage of clade-specific miRNA-reads belonging to each clade.

Sample Statistics

Usage Advice

Note: the **counts on top of each sample bar** show the number of reads used as input for this module.

Sample filtering tip: **samples can be selected by left-clicking** the sample bar (use the ctrl/command key to select multiple).

Sample filtering tip: selecting one or more samples will update the legends to show statistics for only these samples.

Navigation tip: to **quickly navigate** between the report types **use the arrow keys** (also the WASD and HJKL sets of keys work).

Navigation tip: Selected samples can be **shifted using the left/right arrow keys.**

Layout tip: when using the **"Compress reports" button** in the top bar, the page switches to a compact representation.

For more information, see the PDF manual or go to the miRTrace web site.

| Friedländer Lab
