# Supplementary material for: The Small RNA Universe of Capitella teleta
Source: Front Mol Biosci. 2022 Feb 25;9:802814. doi: 10.3389/fmolb.2022.802814 (PMC8915122; doi:10.3389/fmolb.2022.802814)
Supplement: Supplementary file 1 [file DataSheet1.ZIP › Supplement/SupFile6_homolRescAlignments.docx]

6 homolog rescued

**HC-Lgi-Mir-96-P3_5p2 -UAUGGCACCU--GGAGAACU----AGAA--**

**HC-Lgi-Mir-96-P3_5p3 -UAUGGCACCU--GGAGAACU----AGAA--**

**HC-Lgi-Mir-96-P3_5p1 -AAUGGCACU--GGUAGAAU---UCACGG--**

Efe-Mir-96-P2n_5p -UUUGGCACU--UGUAGAAU---UCACUGA-

Efe-Mir-96-P1_5p -AUUGGCACU--GGCGGAAU-AGUCAC----

Cte-Mir-96-P1_5p -CUUGGCACU--GGCGGAAUUAU-CAC----

**HC-Lgi-Mir-96-P3_5p5 -UAUGGCACU--AUUAGAAU---UCACGG--**

Cte-Mir-96-P2_5p -CUUGGCACU--GGUAGAAU---UCACUGA-

**HC-Lgi-Mir-96-P1i_5p -CUUGGCACU-UUUUUGGU----CCACA---**

Efe-Mir-96-P2o_5p -UUUGGCACU-U-AUAGAAU---UCACUGA-

**HC-Lgi-Mir-96-P3_5p4 -CAUGGCACCG----AGUAU---GAGCUUAU**

****** * .

Six homolog recovered miRNAs related to miR-96. Each differs from current C. teleta miR-96, suggesting they evolved through duplication that happened prior to divergence from sister species.

1 homolog rescued 2 novel duplicates

**NovelDup-Cte-Mir-2-o40_3p1 AAUCACAGCCUGCUU---UG--GUCAUU**

Cte-Mir-2-o40_3p AAUCACAGCCUGCUU---UG--GUCAUU

Cte-Mir-2-o39_3p UAUCACAGCCCGCUU---UGUUGACU--

Cte-Mir-2-o36_3p --UCACAGCCAACCU---UGAUGAGCCU

Cte-Mir-2-o38_3p UAUCACAGCCAGCUU---UGAUAAG---

Efe-Mir-2-o47_3p CAUCACAGCCUGCUU---UGUCUGGA--

Efe-Mir-2-o50_3p UAUCACAGCCUGCUU---UGACGAGC--

Efe-Mir-2-o44_3p UAUCACAGCCAGCUU---GGAUCAGG--

Efe-Mir-2-o46_3p UAUCACAGCCUGCUU---GGAUUAUG--

Cte-Mir-2-o37_3p UAUCACAGACCGCUU---GGAUCACA--

Efe-Mir-2-o48_3p UAUCACAGCCGCUU----UGACUGGU--

Efe-Mir-2-o49_3p UAUCACAGCCGCUU----UGACUGGU--

**HC-Lgi-Mir-2-o14_3p3 UAUCACAGCCGCUUUG-AAGACCC----**

Cte-Mir-2-o41_3p UAUCACAGCUAUU----UUGAUCAUU--

Efe-Mir-2-o45_3p UAUCACAGCCAAU----UUGGUCAUC--

Cte-Mir-2-o42_3p UAUCACAGUGGAUUU---GGUUUAU---

**novelDup-Cte-Mir-2-o43_3p2 UAUCACAGUCAAUGCUUUGGGCU-----**

Cte-Mir-2-o43_3p UAUCACAGUCAAUGCUUUGGGCUC----

****** *

Additional copies of miR-2 were found either through recognition of homology to C. teleta orthologs or to other lophotrochozoans. The novel duplicates were copies of mir-2-o40, and the other a duplicate of mir-2-o43. The last more closely resembled miR-o49 from E. fetida.

2 homolog rescued

Efe-Mir-193-P2f-v1_3p -GAAUGCCCUGUGAAAUCCU--GUCU

Efe-Mir-193-P2f-v2_3p --AAUGCCCUGUGAAAUCCU--GUCU

Efe-Mir-193-P2d-v2_3p AUAAUGCCCUUUGAAAUCCU--GAA-

Efe-Mir-193-P2e-v2_3p AUAAUGCCCUUUGAAAUCCU--GAA-

Efe-Mir-193-P2e-v1_3p -UAAUGCCCUUUGAAAUCCU--GAA-

Efe-Mir-193-P2d-v1_3p -UAAUGCCCUUUGAAAUCCU--GAA-

Efe-Mir-193-P2c-v2_3p AUAAUGCCCUUCGAAAUCCU--CUAU

Efe-Mir-193-P2c-v1_3p -UAAUGCCCUUCGAAAUCCU--CUAU

Cgi-Mir-193-P1_3p -UACUGGCCUGCAAAAUCCC--AAA-

**HC-Efe-Mir-193-P2f-v2_3p --CAUGCCCU-AACAAUAAU--CACU**

Cte-Mir-193-P1_3p -AACUGGCCCGUCAAGU-CCC--UCC-

**HC-Lgi-Mir-193-P1_3p -UACUGGCC-GCCACCGAUUGGC-**

***** .. . .

Two additional homologs of miR-193 were found, one that appears similar to E. fetida miR-193-v2 and another is duplicated from C. teleta miR-193-P1

2 homolog rescued

Efe-Mir-10-P2i_5p --AACCCGU-ACAACCGAACUUGUGC-

Cte-Mir-10-P2_5p --AACCCGU-ACAACCGAACUUGUG--

Efe-Mir-10-P2g_5p --AACCCGU-ACUACCGAAUUUGUG--

Efe-Mir-10-P2h_5p --AACCCGU-AGAACCGAACUUGUGCC

Efe-Mir-10-P2j_5p --AACCCGU-AAUACCAGACUUGUG--

Efe-Mir-10-P3g_5p ---UCCCUG-AGACCUUAACUUGUGG-

Efe-Mir-10-P3i_5p ---UCCCUG-AGACCUUAACCUGUGU-

Efe-Mir-10-P3h_5p ---UCCCUG-AGACUUUAACUUGUGA-

Cgi-Mir-10-P3_5p ---UCCCUG-AGACCAUAACUUGUGA-

Cte-Mir-10-P3_5p ---UCCCUG-AGACCCUAACUUGUGA-

Efe-Mir-10-P2k_5p --AACCCGU-ACGCUUGAACUUGUG--

Cgi-Mir-10-P2_5p --AACCCGU-AGAUCCGAACUUGUG--

Efe-Mir-10-P1i_5p --UACCCUGUAGAUCCGAAUU--UGUG

Cgi-Mir-10-P1_5p --UACCCUGUAGAUCCGAAUU--UGU-

Cte-Mir-10-P1_5p --UACCCUGUAGUUCCGGAUU--UGU-

**HC-Lgi-Mir-10-P1_5p --AACCCUGUGGAUCCGGGUU--UGA-**

Efe-Mir-10-P5a_5p -UUACCCUGAAAAACCGAGUU--UGU-

Efe-Mir-10-P1j_5p --UACCCUGUAGAACCGAGCUUGU---

Cte-Mir-10-P7_5p --GACCCUGUAGAACCGAGCUUGUG--

**HC-Efe-Mir-10-P7_5p --UACACUGUAGAAUAGGCUUGU-G--**

Efe-Mir-10-P7_5p --UACACUGUAGAAUAGGCUUGU-G--

Efe-Mir-10-P6_5p AU-ACCCUGU-GAGUAG-GGAAC-GU-

Cgi-Mir-10-P6_5p CUUACCCUGU-AAAUCG-GAGAA-GU-

Cte-Mir-10-P6_5p -UUACCCUGUUACAUUGUAGAAU-GC-

Efe-Mir-10-P5b_5p -UUACCCUGAAGAACCGGACAAGUGU-

Cte-Mir-10-P5_5p -UUACCCUGUCGAACCGAGCGAGUG--

Cgi-Mir-10-P5_5p -UUACCCUGUAGAACCGAGCGAGUG--

*.*

Two homologs of miR-2 were found through homology recovery. One resembles E. fetida mir-10-P2K, and the other related mir-10-P7. This suggests that C. teleta shares some the dramatic duplication of miR-10 seen in *E. fetida.*

1 homolog rescued

Lan-Mir-1990_5p* UGUAAGUUUACAUAGUCCCAGG--

Cte-Mir-1990-P2_5p UGUAAGUUGACAUAGUCCCAGG--

Efe-Mir-1990-P2_5p UGUAAGUUGACAUAGUCCCAGG--

Cte-Mir-1990-P1_5p --UAAGUUGACGUAGUCCCAGGGU

Efe-Mir-1990-P1_5p --UAAGUUGACAUAGUCCCAAGGU

**HC-Lgi-Mir-1990_5p UGUAAGUUGAUAUAGUCCCACAGU**

Cgi-Mir-1990_5p* AGUAAGUUGAUGGGGUCCCAGG--

Npo-Mir-1990_5p* AGUAAGUUGAUGGGGUCCCAGG--

Lgi-Mir-1990_5p AGUAAGUUGAUGGGGUCCCAGG--

****** * . .****** .

An ortholog of miR-1990-5p was found, which had a high degree of similarity to C. teleta and E. fetida. A single base distinguished this copy.

1 homolog rescued

Cte-Mir-1990-P1_3p* ACUUGUGGUUACGUCAGCUUUGC-------

Efe-Mir-1990-P1_3p* UCUUGAGGCUGUGUACGCUU-AC-------

Lan-Mir-1990_3p -C--GGGACUAUGUCAACUUCCAGC---

Efe-Mir-1990-P2_3p* -C--GGGACUUUAGCAACUUCCAGC---

Cgi-Mir-1990_3p -C--GGGACUACGUCAACUACUUGC---

Lgi-Mir-1990_3p* -C--GGGACUACGUCAACUACUAGC---

Npo-Mir-1990_3p -C--GGGACUACGUCAACUGCUGG----

Cte-Mir-1990-P2_3p* -C--GGGACUACGUUAACUUCCAGC---

**HC-Cgi-Mir-1990_3p -U--GGGACUAUGUCAACUUACAAC---**

Efe-Mir-1990-P3_3p UC--GGGACUACAUAGAUUUACGCG----

****** . ..*

One a separate contig, another version of miR-1990 was found that was somewhat divergent from the other miR-1990 genes.

1 homolog rescued

**HC-Efe-Novel-3-P1_3p UGUAGCCAAAGUUUGGGUCCG-**

Efe-Novel-3 UGUAGCCAGAGCUUGGGCCUGC

********.** ***** * *

A copy of a novel E. fetida miRNA was found

1 homolog rescued

Efe-Mir-2691-P1_3p UUUUGCAAUGUUUCACGGUCGGU

Efe-Mir-2691-P2_3p UUUUGCAAUGCAUCACGGUCAGU

Efe-Mir-2691-P4_3p UUUUGCAAUGUAUCACAG-CUGG

Cte-Mir-2691_3p UUUUGCAAAGUAUCACAGCCUGU

Efe-Mir-2691-P3_3p UUUUGCAAAGUAUCACUGCUU--

**HC-Efe-Mir-2691-P1_3p UUUUGCAAAUUU-CAGAUUCAUU**

******** **

Another copy of miR-2691 was found that reflects duplication of this miRNA in E. fetida

1 homolog rescued

Efe-Mir-12-P1_5p UGAGUAUUACAUCAGGUACUUC

Efe-Mir-12-P2_5p UGAGUAUUACUUCAGGUACUGA

Cte-Mir-12_5p UGAGUAUUACAUCAGGUACUGA

Cgi-Mir-12_5p UGAGUAUUACAUCAGGUACUGA

**HC-Lgi-Mir-12_5p CGAGUAUUUCAGCAGGCCCUGA**

******* * **** .** .

Another copy of miR-12 was uncovered, that was divergent outside seed region

1 novel duplicate

Lgi-Mir-92-o28_3p UAUUGCACUUG-UCCCGGCCUUU

Lgi-Mir-92-o29-v2_3p -AUUGCACUUG-UCCCGGCCUGC

Lgi-Mir-92-o31_3p AAUUGCACUUG-UCCCGGCCUUC

Lgi-Mir-92-o30_3p AAUUGCACUUG-UCCCGGCCUGC

Cte-Mir-92-o36_3p AAUUGCACU-GUUCCCGGCCUGC

Lgi-Mir-92-o29-v1_3p AAUUGCACUUGU-CCCGGCCUGC

**NovelDup-Cte-Mir-92-o38_3p AAUUGCACUUGU-CCCGGCCUGC**

Efe-Mir-92-o40_3p AAUUGCACUAGU-CCCGGCCUGC

Cte-Mir-92-o38_3p AAUUGCACUUGU-CCCGGCCUGC

Cte-Mir-92-o37_3p GAUUGCACUAGU-CCCGGCCUUC

Efe-Mir-92-o39_3p AAUUGCACUGAU-CCCGGCCUGC

******** . ********

A duplication of miR-92-o38 was observed.
