# Supplementary material for: The Small RNA Universe of Capitella teleta
Source: Front Mol Biosci. 2022 Feb 25;9:802814. doi: 10.3389/fmolb.2022.802814 (PMC8915122; doi:10.3389/fmolb.2022.802814)
Supplement: Supplementary file 1 [file DataSheet1.ZIP › Supplement/candidate/CAPTEscaffold_769_27752.pdf]

Provisional ID	:	CAPTEscaffold_769_27752
Score total	:	5.6
Score for star read(s)	:	3.9
Score for read counts	:	-0.8
Score for mfe	:	1.6
Score for randfold	:	1.6
Score for cons. seed	:	-0.6
Total read count	:	10
Mature read count	:	7
Loop read count	:	0
Star read count	:	3

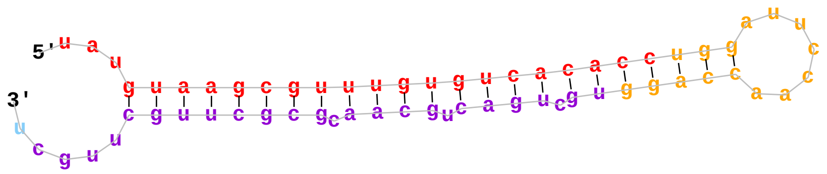

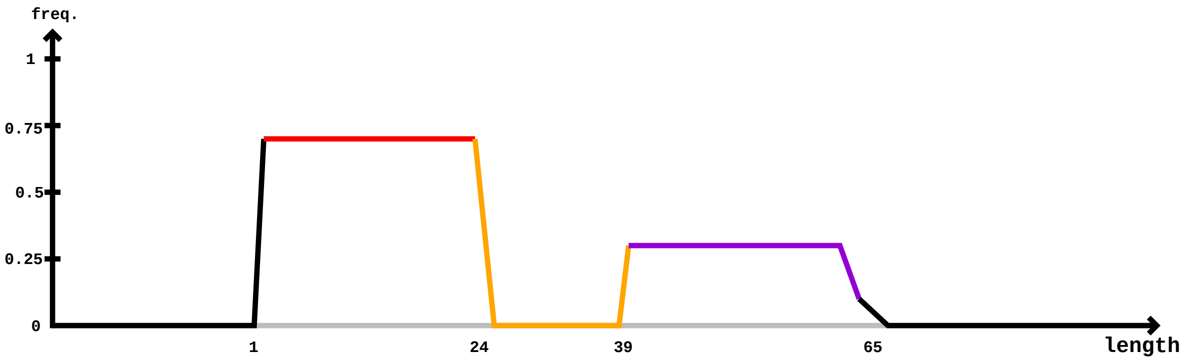

Mature **Star**

```
5'- uguuuguuggagaauacuguuauguaagcguuugugucacaccuggauuccaaccaggugcugacugcaacgcgcuugcuugcugcuaucucgaucggcuucaucggcuucug   -3'    obs
    uguuuguuggagaauacuguuauguaagcguuugugucacaccuggauuccaaccaggugcugacugcaacgcgcuugcuugcugcuaucucgaucggcuucaucggcuucug          exp
    .....(.(.((((.....((...(((((((((((((((((((((((.......))))))).)))).)))).))))))))..)).....)))).).)((((.....))))....    reads      mm        sample
    ....................uauguaagcguuugugucacacc......................................................................    7          0         seq
    ..........................................................ugcugacugcaacgcgcuugcuu................................    1          0         seq
    ..........................................................ugcugacugcaacgcgcuugcuAg...............................    1          1         seq
    ..........................................................ugcugacugcaacgcgcuugcuAgc..............................    1          1         seq
```
