# Supplementary material for: The Small RNA Universe of Capitella teleta
Source: Front Mol Biosci. 2022 Feb 25;9:802814. doi: 10.3389/fmolb.2022.802814 (PMC8915122; doi:10.3389/fmolb.2022.802814)
Supplement: Supplementary file 1 [file DataSheet1.ZIP › Supplement/candidate/CAPTEscaffold_1401_33836.pdf]

Provisional ID	:	CAPTEscaffold_1401_33836
Score total	:	1162.5
Score for star read(s)	:	3.9
Score for read counts	:	1163
Score for mfe	:	-1.5
Score for randfold	:	-2.2
Score for cons. seed	:	-0.6
Total read count	:	2293
Mature read count	:	2285
Loop read count	:	0
Star read count	:	8

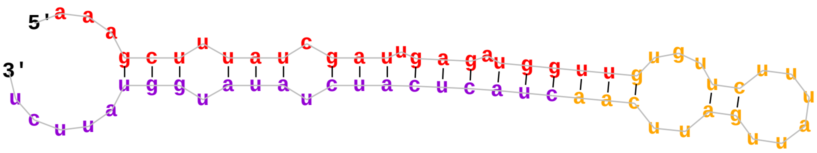

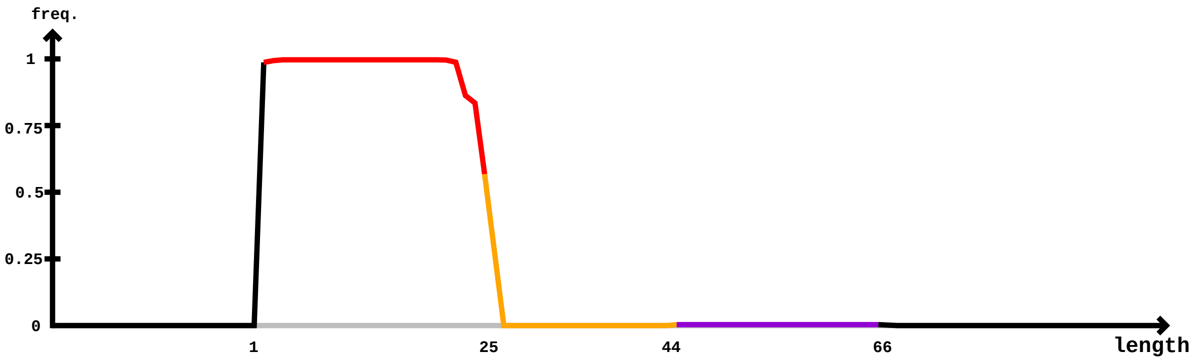


```
                   Mature                                             Star
5'- caaagacaagugaauggucgaaagcuuuaucgauugagaugguuguguucuuuauugauucaacuacucaucuauaugguauucuggcaugaucacuacugacuucaaagcaaa   -3'   obs
    caaagacaagugaauggucgaaagcuuuaucgauugagaugguuguguucuuuauugauucaacuacucaucuauaugguauucuggcaugaucacuacugacuucaaagcaaa         exp
    ..(((.(((((((...((((((.(((.(((.(((.(((.((((((...((......))..)))))))))))).))).))).))).)))....)))))..)).)))........   reads   mm   sample
    ....................aaagcuAuaucgauugaga...........................................................................   2       1    seq
    ....................aaagcuAuaucgauugagau..........................................................................   19      1    seq
    ....................aaagcuAuaucgauugagaug.........................................................................   281     1    seq
    ....................aaagcuAuaucgauugagaugg........................................................................   65      1    seq
    ....................aaagcuAuaucgauugagauggu.......................................................................   609     1    seq
    ....................aaagcuuuaucgauuAagauggu.......................................................................   1       1    seq
    ....................aaagcuuuaucgauugagaugguu......................................................................   20      0    seq
    ....................aaagcuAuaucgauugagaugguu......................................................................   1266    1    seq
    .....................aagcuAuaucgauugagaug.........................................................................   4       1    seq
    .....................aagcuAuaucgauugagauggu.......................................................................   1       1    seq
    .....................aagcuuuaucgauugagaugguu......................................................................   8       0    seq
    .....................aagcuAuaucgauugagaugguu......................................................................   2       1    seq
    ......................agcuAuaucgauugagaug.........................................................................   1       1    seq
    ......................agcuAuaucgauugagaugguu......................................................................   5       1    seq
    ......................agcuuuaucgauugagaugguu......................................................................   1       0    seq
    ...............................................................cuacucaucuauaugguCuucu.............................   7       1    seq
    ...............................................................cuacucaucuauaugguauucu.............................   1       0    seq
```
