# Supplementary material for: The Small RNA Universe of Capitella teleta
Source: Front Mol Biosci. 2022 Feb 25;9:802814. doi: 10.3389/fmolb.2022.802814 (PMC8915122; doi:10.3389/fmolb.2022.802814)
Supplement: Supplementary file 1 [file DataSheet1.ZIP › Supplement/candidate/CAPTEscaffold_94_8175.pdf]

Provisional ID	: CAPTEscaffold_94_8175
Score total	: 409.6
Score for star read(s)	: 3.9
Score for read counts	: 402.4
Score for mfe	: 2.3
Score for randfold	: 1.6
Score for cons. seed	: -0.6
Total read count	: 801
Mature read count	: 799
Loop read count	: 0
Star read count	: 2

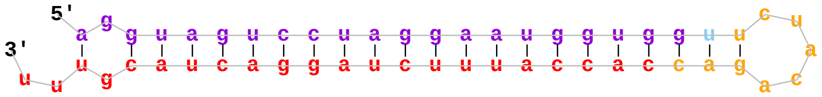

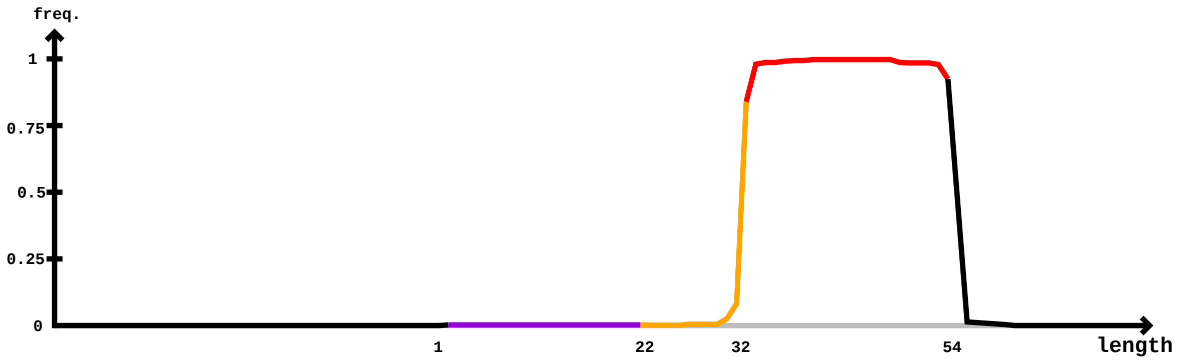

Star Mature

```
5'- aauaaaaccgauguaacggcuuguauaguccuacugaaaagguaguccuaggaaugguggguucuacagaccaccauuucuaggacuacguuuucuacuaggauuugacuacu  -3'  obs
    aauaaaaccgauguaacggcuuguauaguccuacugaaaagguaguccuaggaaugguggguucuacagaccaccauuucuaggacuacguuuucuacuaggauuugacuacu       exp
    .......(((......)))...((..(((((((..(((((.(((((((((((((((((((((.....))))))))))))))))))))).)))))...)))))))..))....   reads   mm   sample
    .......................................agguaguccuaggaauggugC....................................................   2   1   seq
    .............................................................ucuacagaccaccauuucuaggacuacguuu....................   1   0   seq
    ................................................................acagaccaccauuucuaggacu..........................   3   0   seq
    ....................................................................accaccauuucuaggacu..........................   6   0   seq
    ....................................................................accaccauuucuaggacua.........................   1   0   seq
    ....................................................................accaccauuucuaggacuacgu.......................   2   0   seq
    ....................................................................accaccauuucuaggacuacguu......................   5   0   seq
    ....................................................................accaccauuucuaggacuacguuA.....................   1   1   seq
    ....................................................................accaccauuucuaggacuacguuu....................   2   0   seq
    .....................................................................ccaccauuucuaggacuacgu.......................   1   0   seq
    .....................................................................ccaccauuucuaggacuacguu......................   14   0   seq
    .....................................................................ccaccauuucuaggacuacguuu....................   10   0   seq
    .....................................................................ccaccauuucuaggacuacguuA.....................   20   1   seq
    ......................................................................caccauuucuaggacuacgu.......................   2   0   seq
    ......................................................................caccauuucuaggacuacguu......................   18   0   seq
    ......................................................................Aaccauuucuaggacuacguuu....................   1   1   seq
    ......................................................................caccauuucuaggacuacguuA....................   31   1   seq
    ......................................................................caccauuucuagAacuacguuu....................   1   1   seq
    ......................................................................Uaccauuucuaggacuacguuu....................   1   1   seq
    ......................................................................caccauuucuaggacuaUguuu....................   1   1   seq
    ......................................................................caccauuuUuaggacuacguuu....................   1   1   seq
    ......................................................................caccauuucuaggacuacguGu....................   1   1   seq
    ......................................................................caccauuucuaggacuacguuu....................   536   0   seq
    ......................................................................caccauuucuaggaUuacguuu....................   1   1   seq
    ......................................................................caccauuucuaggacuaGguuu....................   1   1   seq
    ......................................................................cacUauuucuaggacuacguuu....................   1   1   seq
    ......................................................................caccauuucuaggacuacguuuu...................   1   0   seq
    ......................................................................caccauuucuaggacuacguuuA...................   13   1   seq
    ......................................................................caccauuucuaggacuacguuuuA..................   2   1   seq
    .......................................................................accauuucuaggacuacguu......................   2   0   seq
    .......................................................................accauuucuaggacuacguuA.....................   8   1   seq
    .......................................................................accauuucuaAgacuacguuu....................   1   1   seq
    .......................................................................accauuucuaggacuacguuu....................   100   0   seq
```

```
                                        Star                             Mature
aauaaaaccgauguaacggcuuguauaguccuacugaaaagguaguccuaggaaugguggauucuacagaccaccauuucuaggacuacguuuucuacuaggauuugacuacu
.......................................................................accauuucuaggacuacguuuu...................    3    0    seq
........................................................................ccauuucuaggacuacguu.....................    5    0    seq
..........................................................................auuucuaggacuacguuuuUu.................    1    1    seq
..........................................................................auuucuaggacuacguuuucu.................    1    0    seq
..........................................................................auuucuaggacuacguuuucuU................    2    1    seq
...........................................................................uuucuaggacuacguuuucuac...............    2    0    seq
.............................................................................ucuaggacuacguuuucuacu..............    3    0    seq
```
