# Supplementary material for: The Small RNA Universe of Capitella teleta
Source: Front Mol Biosci. 2022 Feb 25;9:802814. doi: 10.3389/fmolb.2022.802814 (PMC8915122; doi:10.3389/fmolb.2022.802814)
Supplement: Supplementary file 1 [file DataSheet1.ZIP › Supplement/candidate/CAPTEscaffold_1377_33899.pdf]

Provisional ID	:	CAPTEscaffold_1377_33899
Score total	:	19.2
Score for star read(s)	:	3.9
Score for read counts	:	12.4
Score for mfe	:	1.9
Score for randfold	:	1.6
Score for cons. seed	:	-0.6
Total read count	:	36
Mature read count	:	35
Loop read count	:	0
Star read count	:	1

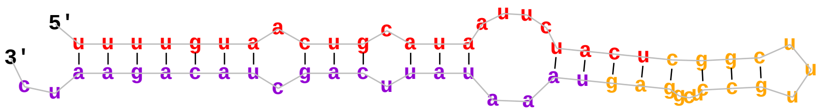

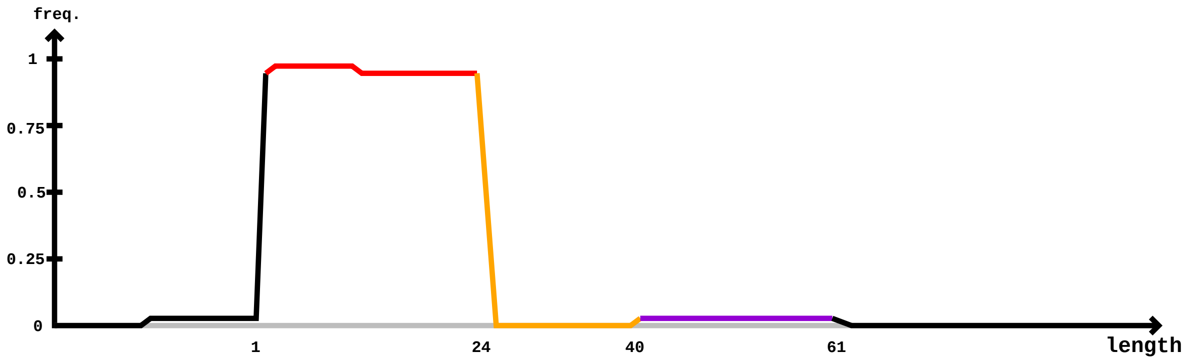

Mature Star

```
5'- caucugggauggccuagugauuuuguaacugcauaauucuacucggcuuugccucggaguaaauauucagcuacagaaucacucggccguccgauccgacaauaaaagccucc -3'   obs
    caucugggauggccuagugauuuuguaacugcauaauucuacucggcuuugccucggaguaaauauucagcuacagaaucacucggccguccgauccgacaauaaaagccucc         exp
    ....(.((((((((.((((((((((((.(((.(((....((((((((...)))...)))))..))).))).))))))))))))).)))))))).)..................    reads   mm   sample
    ........auggccuagugauuCuguaacu...................................................................................    1       1    seq
    ....................uuuuguaacugcauaauucuacu......................................................................    18      0    seq
    ....................uuuuguaacugAauaauucuacu......................................................................    13      1    seq
    ....................uuuuguaacugAauaauucuacuc.....................................................................    3       1    seq
    .....................uuuguaacugcauaauucuacu......................................................................    1       0    seq
    ...........................................................uaaauauucagcuacagaauc.................................    1       0    seq
```
