# Supplementary material for: The Small RNA Universe of Capitella teleta
Source: Front Mol Biosci. 2022 Feb 25;9:802814. doi: 10.3389/fmolb.2022.802814 (PMC8915122; doi:10.3389/fmolb.2022.802814)
Supplement: Supplementary file 1 [file DataSheet1.ZIP › Supplement/candidate/CAPTEscaffold_20012_46009.pdf]

Provisional ID	:	CAPTEscaffold_20012_46009
Score total	:	1.6
Score for star read(s)	:	-1.3
Score for read counts	:	0
Score for mfe	:	1.9
Score for randfold	:	1.6
Score for cons. seed	:	-0.6
Total read count	:	18
Mature read count	:	17
Loop read count	:	0
Star read count	:	1

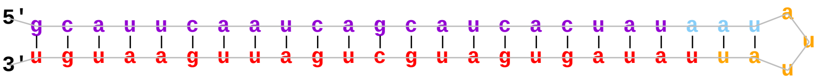

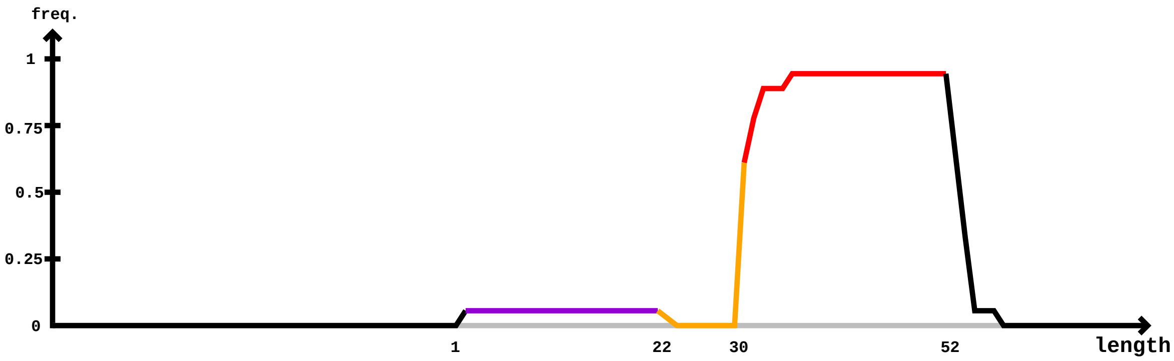

Star Mature

```
5'- aacucgaucacggcccucaaccgcaaaacauaauauguuuugcauucaaucagcaucacuauaauauuauuauagugaugcugauugaaugucauugaauacuuggcauuga   -3'    obs
    aacucgaucacggcccucaaccgcaaaacauaauauguuuugcauucaaucagcaucacuauaauauuauuauagugaugcugauugaaugucauugaauacuuggcauuga          exp
    .......(((..(((.......(((((((((...)))))))))((((((((((((((((((((((...))))))))))))))))))))))..............)))..)))   reads   mm   sample
    .........................................gcauucaaucagcaucacuau..................................................   1       0    seq
    ......................................................................uauagugaugcugauugaaugu....................   10      0    seq
    ......................................................................uauagugaugcugauugaauguc...................   1       0    seq
    .......................................................................auagugaugcugauugaauguca..................   3       0    seq
    ........................................................................uagugaugcugauugaauguca..................   2       0    seq
    ...........................................................................ugaugcugauugaaugucauug...............   1       0    seq
```
