# Supplementary material for: The Small RNA Universe of Capitella teleta
Source: Front Mol Biosci. 2022 Feb 25;9:802814. doi: 10.3389/fmolb.2022.802814 (PMC8915122; doi:10.3389/fmolb.2022.802814)
Supplement: Supplementary file 1 [file DataSheet1.ZIP › Supplement/candidate/CAPTEscaffold_60_5425.pdf]

Provisional ID	:	CAPTEscaffold_60_5425
Score total	:	461.9
Score for star read(s)	:	3.9
Score for read counts	:	455.4
Score for mfe	:	1.6
Score for randfold	:	1.6
Score for cons. seed	:	-0.6
Total read count	:	905
Mature read count	:	807
Loop read count	:	0
Star read count	:	98

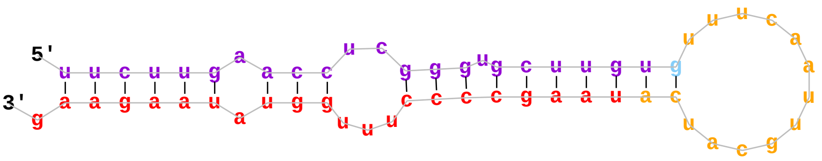

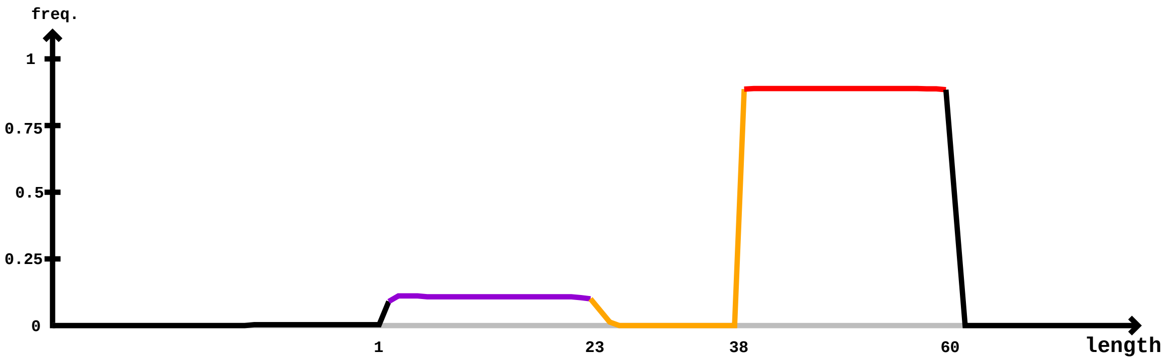


```
                            Star                                  Mature
5'- cugaucaagacuuauuugaguaugauuggcucuuucuugaaccucgggugcuuguguuucaauugcaucauaagccccuuugguauaagaagaagccgaagcucggucugc  -3'   obs
    cugaucaagacuuauuugaguaugauuggcucuuucuugaaccucgggugcuuguguuucaauugcaucauaagccccuuugguauaagaagaagccgaagcucggucugc         exp
    .......(((((.....((((....((((((.(((((((.(((..(((.(((((((............))))))))))...))).))))))).)))))).)))))))))..   reads   mm   sample
    ...................guaugauuggcucuuucu..........................................................................   2       0    seq
    ...................gCaugauuggcucuuucu..........................................................................   1       1    seq
    .................................uucuugaaccucgggugcuu..........................................................   3       0    seq
    .................................uucuugaaccucgggugcuug.........................................................   4       0    seq
    .................................uucuugaaccucgggugcuugu........................................................   50      0    seq
    .................................Aucuugaaccucgggugcuugu........................................................   1       1    seq
    .................................uucuugaaccucggguCcuugu........................................................   1       1    seq
    .................................uucuugaaccucgggugcuuguA.......................................................   1       1    seq
    .................................uucuugaaccucgggugcuugug.......................................................   9       0    seq
    .................................uucuugaaccucgggugcuugugA......................................................   8       1    seq
    .................................uucuugaaccucgggugcuugugG......................................................   2       1    seq
    ..................................ucuugaaccucgggugcuugu........................................................   4       0    seq
    ..................................ucuugaaccucgggugcuuCu........................................................   3       1    seq
    ..................................ucuugaaccucgggugcuuCug.......................................................   6       1    seq
    ..................................ucuugaaccucgggugcuugug.......................................................   4       0    seq
    ..................................ucuugaaccucgggugcuugugA......................................................   1       1    seq
    ..................................ucuugaaccucgggugcuuCugu......................................................   1       1    seq
    ......................................................................uaagccccuuugguauaag......................   1       0    seq
    ......................................................................uaagccccuuugguauaagaa....................   3       0    seq
    ......................................................................uaagccccuuugguauaagaUg...................   1       1    seq
    ......................................................................uaagcUccuuugguauaagaag...................   1       1    seq
    ......................................................................uaagccccuuugguGuaagaag...................   1       1    seq
    ......................................................................uaagccccuuugguauaagaaC...................   10      1    seq
    ......................................................................uaaCccccuuugguauaagaag...................   1       1    seq
    ......................................................................uaagccccuuugguauaGgaag...................   1       1    seq
    ......................................................................uaagccccuAugguauaagaag...................   1       1    seq
    ......................................................................uaagccccuuugguauaagaGg...................   3       1    seq
    ......................................................................uaagcccUuuugguauaagaag...................   2       1    seq
    ......................................................................uaagccccuuuAguauaagaag...................   3       1    seq
    ......................................................................uaagccccuuugguauaagaag...................   750     0    seq
    ......................................................................uaagccccuuAgguauaagaag...................   1       1    seq
    ......................................................................uaagccccuuuUguauaagaag...................   1       1    seq
    ......................................................................Aaagccccuuugguauaagaag...................   5       1    seq
```

```
                                Star                                Mature
cugaucaagacuuauuugaguaugauuggcucuuucuugaaccucgggugcuuguguuucaauugcaucauaagccccuuugguauaagaagaagccgaagcucggucugc
......................................................................uaagccccuuugguauGagaag...................   13          1           seq
......................................................................uaagccccuuugguauaagaaga..................   7           0           seq
.......................................................................aagccccuuugguauaagaag...................   1           0           seq
.......................................................................aagccccuuugguauaagaaga..................   1           0           seq
```
