# Supplementary material for: The Small RNA Universe of Capitella teleta
Source: Front Mol Biosci. 2022 Feb 25;9:802814. doi: 10.3389/fmolb.2022.802814 (PMC8915122; doi:10.3389/fmolb.2022.802814)
Supplement: Supplementary file 1 [file DataSheet1.ZIP › Supplement/candidate/CAPTEscaffold_179_12875.pdf]

Provisional ID	:	CAPTEscaffold_179_12875
Score total	:	3.2
Score for star read(s)	:	3.9
Score for read counts	:	1.1
Score for mfe	:	1
Score for randfold	:	-2.2
Score for cons. seed	:	-0.6
Total read count	:	14
Mature read count	:	12
Loop read count	:	0
Star read count	:	2

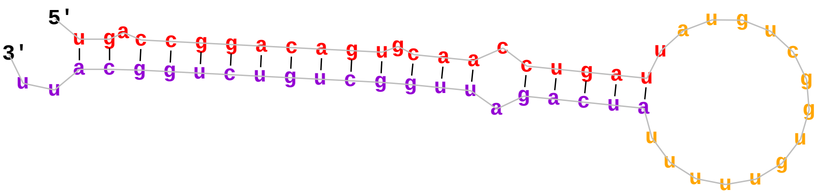

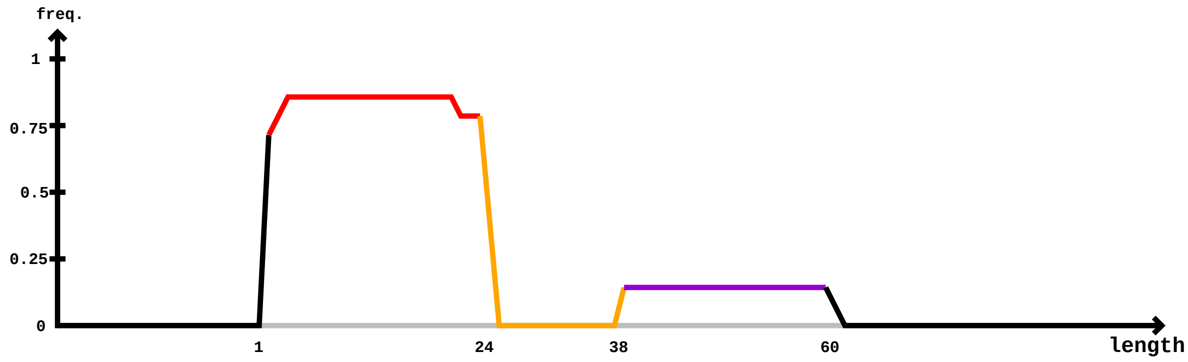

Mature Star

```
5'- cgcucaccuggcggucaauuugaccggacagugcaaccugauuaugucgguguuuuuaucagauuggcugucuggcauuuggggcaccagacugauugaaauccuggauuaaa   -3'   obs
    cgcucaccuggcggucaauuugaccggacagugcaaccugauuaugucgguguuuuuaucagauuggcugucuggcauuuggggcaccagacugauugaaauccuggauuaaa         exp
    .......((((.(.((....((.(((((((((.(((.(((((...............))))).))))))))))))))....)).).))))..(((((.(.....).)))))..   reads   mm   sample
    ....................ugaccggacagugcaaccugauu......................................................................   9       0    seq
    ....................ugaccggacagugcaaccugauua.....................................................................   1       0    seq
    .....................gaccggacagugcaaccugauu......................................................................   1       0    seq
    ......................accggacagugcaaccug.........................................................................   1       0    seq
    .........................................................aucagauuggcugucuggcauu..................................   2       0    seq
```
