# Supplementary material for: The Small RNA Universe of Capitella teleta
Source: Front Mol Biosci. 2022 Feb 25;9:802814. doi: 10.3389/fmolb.2022.802814 (PMC8915122; doi:10.3389/fmolb.2022.802814)
Supplement: Supplementary file 1 [file DataSheet1.ZIP › Supplement/candidate/CAPTEscaffold_189_13539.pdf]

Provisional ID	:	CAPTEscaffold_189_13539
Score total	:	46.9
Score for star read(s)	:	3.9
Score for read counts	:	39.9
Score for mfe	:	2.1
Score for randfold	:	1.6
Score for cons. seed	:	-0.6
Total read count	:	90
Mature read count	:	88
Loop read count	:	0
Star read count	:	2

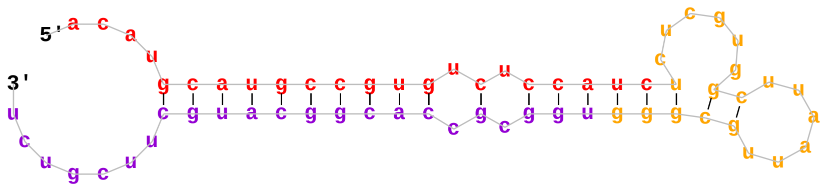

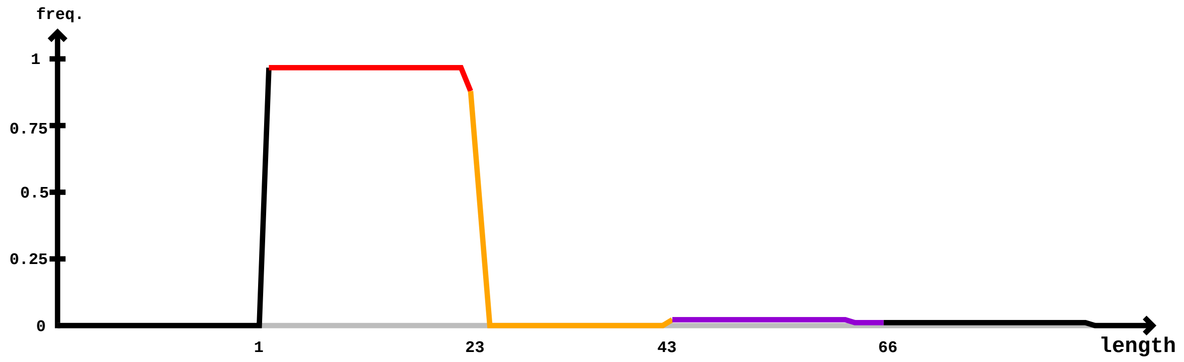

Mature Star

```
5'- cucuguuggcgcaugcgcagacaugcaugccgugucuccaucucucguggcuuaauugcggguggcgccacggcaugcuucgucuggacacggcggccccaacaccuuuguu   -3'   obs
    cucuguuggcgcaugcgcagacaugcaugccgugucuccaucucucguggcuuaauugcggguggcgccacggcaugcuucgucuggacacggcggccccaacaccuuuguu         exp
    ...(((((((((.((..(((((..((((((((((.(.((((((......((......)))))))).).))))))))))...)))))..))..)))...))))))........   reads   mm   sample
    ....................acaugcaugccgugucuccau.......................................................................   6       0    seq
    ....................acaugcaugccCugucuccau.......................................................................   2       1    seq
    ....................acaugcaugccCugucuccauc......................................................................   11      1    seq
    ....................acaugcaugccgugucuccaAc......................................................................   1       1    seq
    ....................acaugcaugccgugucuccauU......................................................................   1       1    seq
    ....................acaugcaugccgugucuccauc......................................................................   67      0    seq
    ..............................................................uggcgccacggcaugcuuc...............................   1       0    seq
    ..............................................................uggcgccacggcaugcuucgucu...........................   1       0    seq
    .....................................................................................ggacacggcggccccaacacc......   1       0    seq
```
