# Supplementary material for: The Small RNA Universe of Capitella teleta
Source: Front Mol Biosci. 2022 Feb 25;9:802814. doi: 10.3389/fmolb.2022.802814 (PMC8915122; doi:10.3389/fmolb.2022.802814)
Supplement: Supplementary file 1 [file DataSheet1.ZIP › Supplement/candidate/CAPTEscaffold_324_18348.pdf]

Provisional ID	:	CAPTEscaffold_324_18348
Score total	:	5867.6
Score for star read(s)	:	3.9
Score for read counts	:	5861.1
Score for mfe	:	1.6
Score for randfold	:	1.6
Score for cons. seed	:	-0.6
Total read count	:	11508
Mature read count	:	11481
Loop read count	:	4
Star read count	:	23

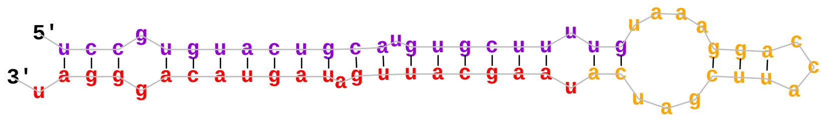

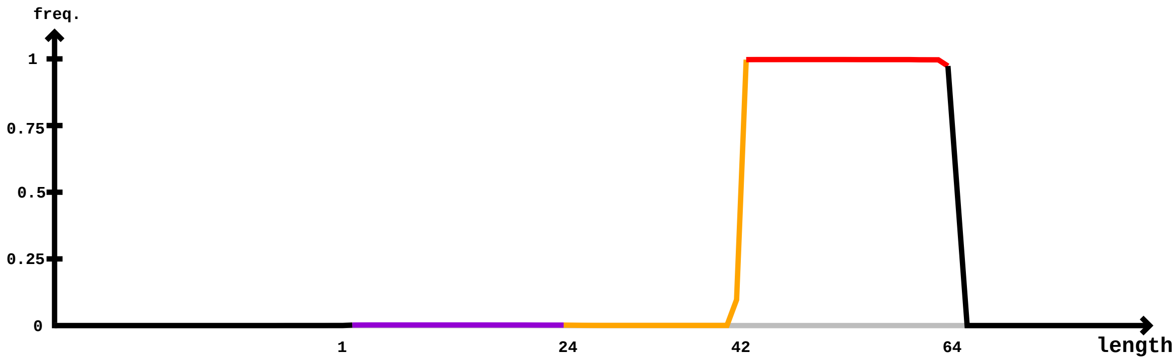


```
                              Star                                      Mature
5'- agcgcagaacuugccuugacacucacucuuccguguacugcaugugcuuuuguaaaggaccauucgaucauaagcauugauaguacagggaugggugauguugguacagcca   -3'   obs
    agcgcagaacuugccuugacacucacucuuccguguacugcaugugcuuuuguaaaggaccauucgaucauaagcauugauaguacagggaugggugauguugguacagcca         exp
    ........((((((..((((.((((((.(((.(((((((((.((((((.((....(((...)))...)).)))))))).))))))).))).))))))))))))))).))...   reads   mm   sample
    ......................ucacucuuccguguacugcaug....................................................................   1       0    seq
    ...........................cuuccguguacugcaugugcuu...............................................................   1       0    seq
    .............................uccguguacugcaugugcu................................................................   1       0    seq
    .............................uccguguacugcaugCgcuuuug............................................................   2       1    seq
    .............................uccguguacugcaugugcuuuug............................................................   12      0    seq
    .............................uccguguacugcaugugcuuuugA...........................................................   2       1    seq
    .............................uccguguacugcaugugcuuuugAa..........................................................   2       1    seq
    .............................uccguguacugcaugugcuuuugAaa.........................................................   1       1    seq
    ..............................ccguguacugcaugugcu................................................................   1       0    seq
    ..............................ccguguacugcaugugcuuuug............................................................   1       0    seq
    ...............................cguguacugcaugugcuuuugu...........................................................   1       0    seq
    ....................................................uaaaggaccauucgauca..........................................   4       0    seq
    ....................................................uaaaggaccauucgaucauaagcauugCu...............................   1       1    seq
    ....................................................uaaaggaccauucgaucauaagcauugCua..............................   1       1    seq
    .....................................................................auaagcauugCuaguacag........................   4       1    seq
    .....................................................................auaagcauugCuaguacagg.......................   1       1    seq
    .....................................................................auaagcauugCuaguacaggg......................   1       1    seq
    .....................................................................auaagcauugCuaguacaggga.....................   30      1    seq
    .....................................................................auaagcauugCuaguacagggau...................   1062    1    seq
    .....................................................................auaagcauugCuaguacagggaug..................   12      1    seq
    ......................................................................uaagcauugCuaguacag........................   3       1    seq
    ......................................................................uaagcauugauaguacaggga.....................   13      0    seq
    ......................................................................uaagcauugCuaguacaggga.....................   226     1    seq
    ......................................................................uaagcauugCuaguacagggau....................   8824    1    seq
    ......................................................................uaagcauugauaguacagggau....................   599     0    seq
    ......................................................................uaagcauugauaguacagggaA....................   1       1    seq
    ......................................................................uaagcauAgauaguacagggau....................   1       1    seq
    ......................................................................uaagcauugauaguacagggaC....................   1       1    seq
    ......................................................................Aaagcauugauaguacagggau....................   4       1    seq
    ......................................................................uaagcauugGuaguacagggau....................   2       1    seq
    ......................................................................uaagcaAugauaguacagggau....................   1       1    seq
    ......................................................................uaagAauugauaguacagggau....................   1       1    seq
    ......................................................................uaaUcauugauaguacagggaug...................   1       1    seq
```

Star Mature

```
agcgcagaacuugccuugacacucacucuuccguguacugcaugugcuuuuguaaaggaccauucgaucauaagcauugauaguacagggaugggugauguugguacagcca
......................................................................uaagcauugGuaguacagggaug...................   1     1     seq
......................................................................uaagcauugauaguacagggauA...................   10    1     seq
......................................................................uaagcauugauaguacagggauU...................   30    1     seq
......................................................................uaagcauugCuaguacagggaug...................   646   1     seq
......................................................................uaagcauugauaguacagggaug...................   4     0     seq
.......................................................................aagcauugCuaguacagggau....................   2     1     seq
..........................................................................cauugCuaguacagggau....................   1     1     seq
```
