# Supplementary material for: The Small RNA Universe of Capitella teleta
Source: Front Mol Biosci. 2022 Feb 25;9:802814. doi: 10.3389/fmolb.2022.802814 (PMC8915122; doi:10.3389/fmolb.2022.802814)
Supplement: Supplementary file 1 [file DataSheet1.ZIP › Supplement/candidate/CAPTEscaffold_795_28880.pdf]

Provisional ID	:	CAPTEscaffold_795_28880
Score total	:	71.3
Score for star read(s)	:	3.9
Score for read counts	:	63.9
Score for mfe	:	2.5
Score for randfold	:	1.6
Score for cons. seed	:	-0.6
Total read count	:	137
Mature read count	:	129
Loop read count	:	0
Star read count	:	8

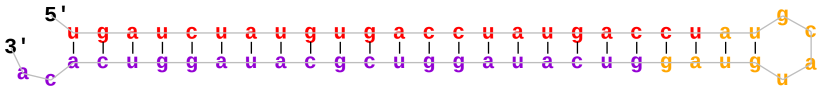

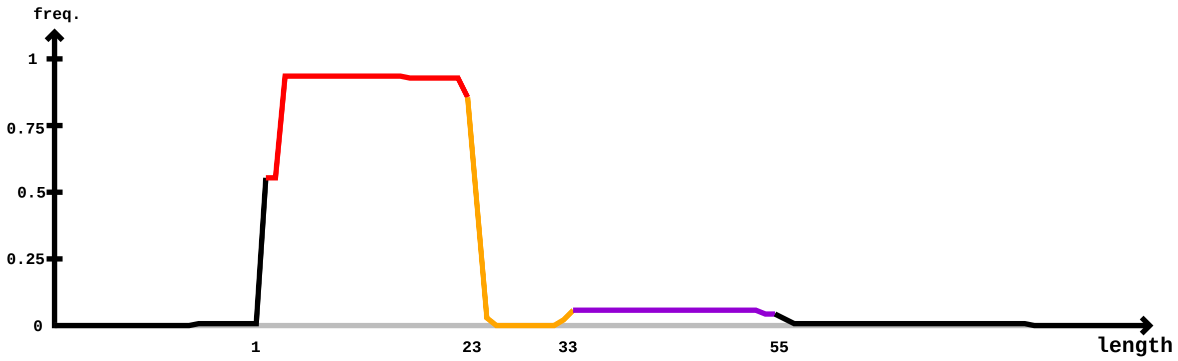

Mature Star

```
5'- ccaccagguuggacagacugugaucuaugugaccuaugaccuaugcauguaggucauaggucgcauaggucacagucugaccuacaugcuacccaugcagcggaacucaagu   -3'    obs
    ccaccagguuggacagacugugaucuaugugaccuaugaccuaugcauguaggucauaggucgcauaggucacagucugaccuacaugcuacccaugcagcggaacucaagu          exp
    .......((.((.(((((((((((((((((((((((((((((((....))))))))))))))))))))))))))))))).)).)).(((.......)))............. reads     mm    sample
    .............cagacugugaucuaugugaccu.............................................................................  1         0     seq
    ....................ugaucuaugugaccuaugacc.......................................................................  4         0     seq
    ....................Agaucuaugugaccuaugaccu......................................................................  1         1     seq
    ....................ugauUuaugugaccuaugaccu......................................................................  8         1     seq
    ....................ugaucuaugugaccuaugaccu......................................................................  42        0     seq
    ....................ugaucuaugugaccuGugaccu......................................................................  21        1     seq
    ......................aucuaugugaccuaugacc.......................................................................  5         0     seq
    ......................aucuaugugaccuGugacc.......................................................................  1         1     seq
    ......................auUuaugugaccuaugaccu......................................................................  2         1     seq
    ......................aucuaugugaccuaugaccu......................................................................  20        0     seq
    ......................aucuaugugaccuaugaccCa.....................................................................  1         1     seq
    ......................auUuaugugaccuaugaccua.....................................................................  1         1     seq
    ......................aucuaugugaccuaCgaccua.....................................................................  1         1     seq
    ......................aucuaugugaccuaugaccuG.....................................................................  1         1     seq
    ......................aucuaugugaccuaugaccua.....................................................................  17        0     seq
    ......................aucuaugugaccuaugaccuau....................................................................  4         0     seq
    ...................................................ggucauaggucgcauagguca........................................  3         0     seq
    ....................................................gucauaggucgcauaggucaca......................................  3         0     seq
    ....................................................gucauGggucgcauaggucaca......................................  2         1     seq
    ........................................................................cagucugaccuacaugcuacccaugAag............  1         1     seq
```
