# Supplementary material for: The Small RNA Universe of Capitella teleta
Source: Front Mol Biosci. 2022 Feb 25;9:802814. doi: 10.3389/fmolb.2022.802814 (PMC8915122; doi:10.3389/fmolb.2022.802814)
Supplement: Supplementary file 1 [file DataSheet1.ZIP › Supplement/candidate/CAPTEscaffold_9984_43267.pdf]

Provisional ID	:	CAPTEscaffold_9984_43267
Score total	:	1158.8
Score for star read(s)	:	3.9
Score for read counts	:	1159.5
Score for mfe	:	-1.6
Score for randfold	:	-2.2
Score for cons. seed	:	-0.6
Total read count	:	2286
Mature read count	:	2278
Loop read count	:	0
Star read count	:	8

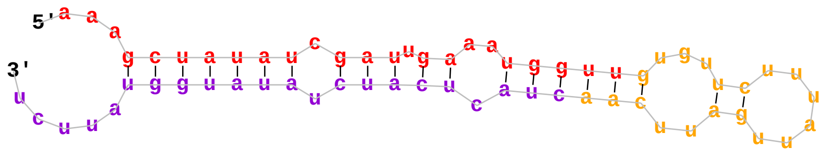

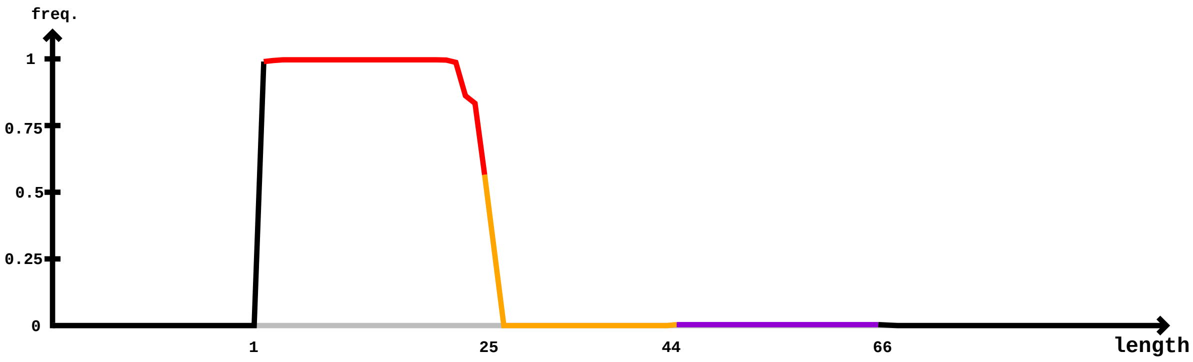

Mature   Star

```
5'- caaagacacgugaauggucgaaagcuauaucgauugaaaugguuguguucuuuauugauucaacuacucaucuauaugguauucuggcacgaucacuacugacuucaaagcaaa  -3'  obs
    caaagacacgugaauggucgaaagcuauaucgauugaaaugguuguguucuuuauugauucaacuacucaucuauaugguauucuggcacgaucacuacugacuucaaagcaaa       exp
    ..(((.((.((((.((((((((.(((((((.(((.((..((((((...((......))..)))))).))))).))))))).))).))).)).))))...)).))).........  reads  mm  sample
    ....................aaagcuauaucgauugaGa...........................................................................  2      1   seq
    ....................aaagcuauaucgauugaGau..........................................................................  19     1   seq
    ....................aaagcuauaucgauugaaau..........................................................................  1      0   seq
    ....................aaagcuauaucgauugaGaug.........................................................................  281    1   seq
    ....................aaagcuauaucgauugaGaugg........................................................................  65     1   seq
    ....................aaagcuauaucgauugaaauggu.......................................................................  2      0   seq
    ....................aaagcuauaucgauugaGauggu.......................................................................  609    1   seq
    ....................aaagcuauaucgauugaaaugguu......................................................................  16     0   seq
    ....................aaagcuauaucgauugaaauAguu......................................................................  1      1   seq
    ....................aaagcuGuaucgauugaaaugguu......................................................................  1      1   seq
    ....................aaagcuauaucgauugaGaugguu......................................................................  1266   1   seq
    .....................aagcuauaucgauugaGaug.........................................................................  4      1   seq
    .....................aagcuauaucgauugaGauggu.......................................................................  1      1   seq
    .....................aagcuauaucgauugaGaugguu......................................................................  2      1   seq
    .....................aagcuauaucgauugaaaugguu......................................................................  2      0   seq
    ......................agcuauaucgauugaGaug.........................................................................  1      1   seq
    ......................agcuauaucgauugaGaugguu......................................................................  5      1   seq
    ...............................................................cuacucaucuauaugguCuucu.............................  7      1   seq
    ...............................................................cuacucaucuauaugguauucu.............................  1      0   seq
```
