# Supplementary material for: The Small RNA Universe of Capitella teleta
Source: Front Mol Biosci. 2022 Feb 25;9:802814. doi: 10.3389/fmolb.2022.802814 (PMC8915122; doi:10.3389/fmolb.2022.802814)
Supplement: Supplementary file 1 [file DataSheet1.ZIP › Supplement/candidate/CAPTEscaffold_41_4379.pdf]

Provisional ID	:	CAPTEscaffold_41_4379
Score total	:	0.4
Score for star read(s)	:	3.9
Score for read counts	:	3.2
Score for mfe	:	-3.8
Score for randfold	:	-2.2
Score for cons. seed	:	-0.6
Total read count	:	18
Mature read count	:	13
Loop read count	:	2
Star read count	:	3

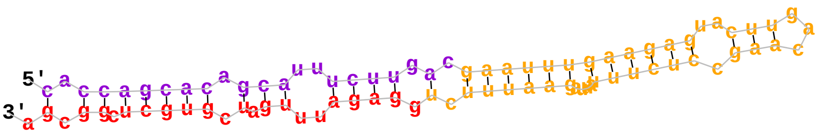

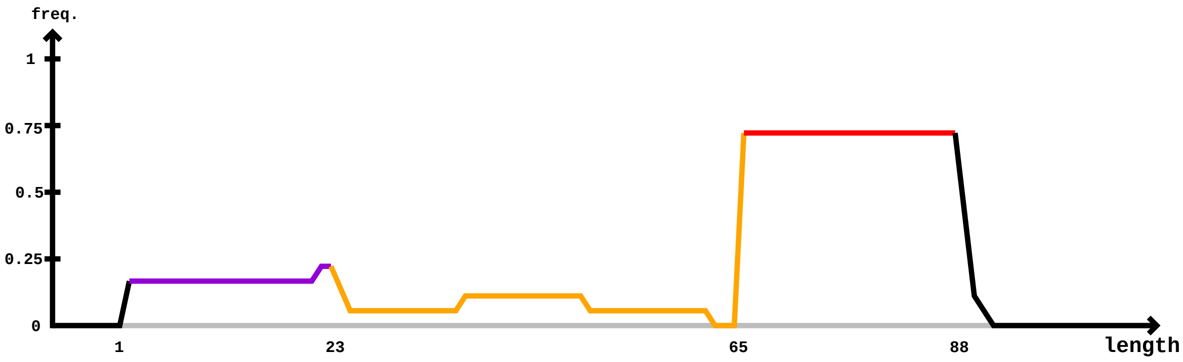

Star **Mature**

```
5'- cagauacaccagcacagcauuucuugacgaauuugaagaguacuugacaagccucuuuauuagaauuucuggagauuugaucgugcucggcgauuuuacucuauacuuugaaa   -3'    obs
    cagauacaccagcacagcauuucuugacgaauuugaagaguacuugacaagccucuuuauuagaauuucuggagauuugaucgugcucggcgauuuuacucuauacuuugaaa          exp
    .((((.(.(((((((.(((..((((.(.((((((((((((..(((...))).))))))....)))))).).))))..)).).))))).)).)))))................. reads  mm  sample
    ......caccagcacagcauuucuugac..................................................................................... 2      0   seq
    ......caccagcacagcauuucuugacg.................................................................................... 1      0   seq
    ..........................acgaauuugaagaguacuugacaagccu........................................................... 1      0   seq
    .........................................acuugacaagccucuuuauuagaauu.............................................. 1      0   seq
    ......................................................................ggagauuugaucgugcucggcga.................... 10     0   seq
    ......................................................................ggagauuugaucgugcucggcgau................... 1      0   seq
    ......................................................................ggagauuugaucgugcucggcgauu.................. 1      0   seq
    ......................................................................ggagauuugaucgugcucggcgauuu................. 1      0   seq
```
