# Supplementary material for: The Small RNA Universe of Capitella teleta
Source: Front Mol Biosci. 2022 Feb 25;9:802814. doi: 10.3389/fmolb.2022.802814 (PMC8915122; doi:10.3389/fmolb.2022.802814)
Supplement: Supplementary file 1 [file DataSheet1.ZIP › Supplement/candidate/CAPTEscaffold_958_29875.pdf]

Provisional ID	:	CAPTEscaffold_958_29875
Score total	:	32.1
Score for star read(s)	:	3.9
Score for read counts	:	26.6
Score for mfe	:	0.6
Score for randfold	:	1.6
Score for cons. seed	:	-0.6
Total read count	:	64
Mature read count	:	35
Loop read count	:	0
Star read count	:	29

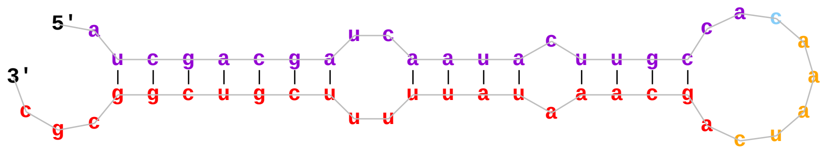

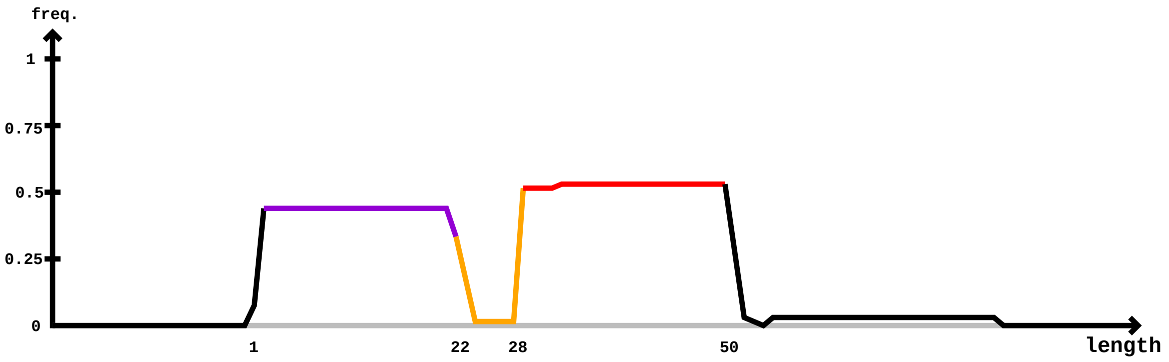

Star Mature

```
5'- aguguuaucacaccagauguaucgacgaucaauacuugccacaaaucagcaaauauuuuucgucggcgccucuuccggaccugcgggaaaacccaguauccacuggaacgu -3'    obs
    aguguuaucacaccagauguaucgacgaucaauacuugccacaaaucagcaaauauuuuucgucggcgccucuuccggaccugcgggaaaacccaguauccacuggaacgu        exp
    .((((....))))..((.((.(((((((..((((.((((.........)))).))))..))))))).)).))(((((......)))))....(((((....))))).....    reads    mm    sample
    ...................uaucgacgaucaauacuugcca......................................................................    5        0     seq
    ....................aucgacgaucaauacuugcc.......................................................................    7        0     seq
    ....................Gucgacgaucaauacuugcca......................................................................    1        1     seq
    ....................aucgacgaucaauacuugcca......................................................................    15       0     seq
    ....................aucgacgaucaauacuugccacaaauc................................................................    1        0     seq
    ...............................................agcaaauauuuuucgucggcgc..........................................    8        0     seq
    ...............................................agcaaauauuGuucgucggcgc..........................................    23       1     seq
    ...............................................agcaaauauuGuucgucggcgcc.........................................    1        1     seq
    ...............................................agcaaauauuuuucgucggcgcU.........................................    1        1     seq
    ...............................................agcaaauauuuuucgucggcgccu........................................    1        0     seq
    ...................................................aauauuGuucgucggcgccuc.......................................    1        1     seq
    .........................................................................uccggaccugcgggaaaacccagu..............    2        0     seq
```
