# Supplementary material for: The Small RNA Universe of Capitella teleta
Source: Front Mol Biosci. 2022 Feb 25;9:802814. doi: 10.3389/fmolb.2022.802814 (PMC8915122; doi:10.3389/fmolb.2022.802814)
Supplement: Supplementary file 1 [file DataSheet1.ZIP › Supplement/candidate/CAPTEscaffold_147_11165.pdf]

Provisional ID	:	CAPTEscaffold_147_11165
Score total	:	8.7
Score for star read(s)	:	3.9
Score for read counts	:	2.7
Score for mfe	:	1.2
Score for randfold	:	1.6
Score for cons. seed	:	-0.6
Total read count	:	17
Mature read count	:	9
Loop read count	:	0
Star read count	:	8

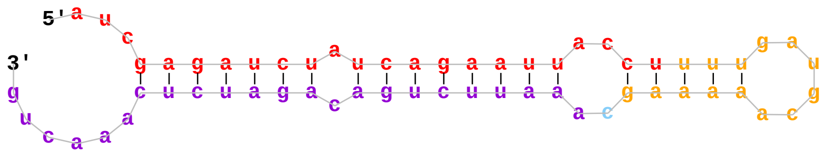

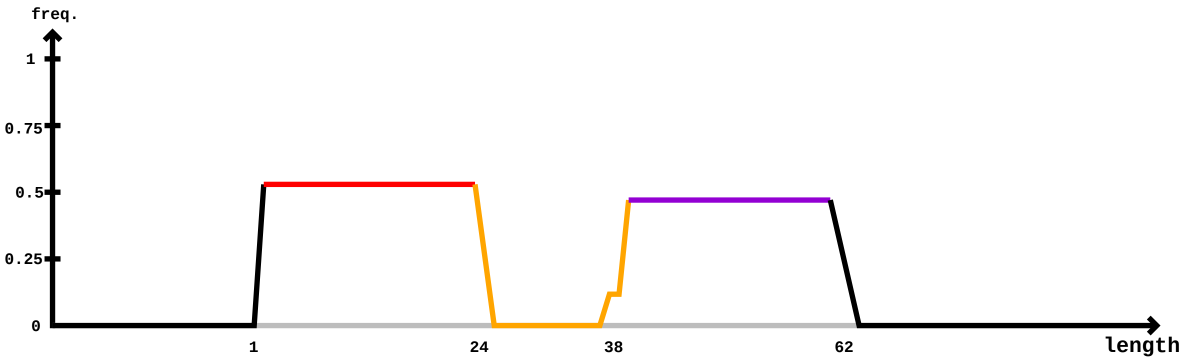

Mature **Star**

```
5'- ugacaacaacaagacaauggaucgagaucuaucagaauuaccuuuugaugcaaaaagcaaauucugacagaucucaaacuguuguccaagcagcgauucgauuaaagaaagcg   -3'    obs
    ugacaacaacaagacaauggaucgagaucuaucagaauuaccuuuugaugcaaaaagcaaauucugacagaucucaaacuguuguccaagcagcgauucgauuaaagaaagcg          exp
    ...........((((((((...(((((((.((((((((..(((((......)))))..)))))))).)))))))...))))))))......((..(((.......))).)).    reads      mm          sample
    ....................aucgagaucuaucagaauuaccu......................................................................    9          0           seq
    ........................................................gcaaauucugacagaucucaaacu.................................    2          0           seq
    ..........................................................Uaauucugacagaucucaaacu.................................    2          1           seq
    ..........................................................Uaauucugacagaucucaaacug................................    4          1           seq
```
