# Supplementary material for: The Small RNA Universe of Capitella teleta
Source: Front Mol Biosci. 2022 Feb 25;9:802814. doi: 10.3389/fmolb.2022.802814 (PMC8915122; doi:10.3389/fmolb.2022.802814)
Supplement: Supplementary file 1 [file DataSheet1.ZIP › Supplement/candidate/CAPTEscaffold_488_22752.pdf]

Provisional ID	:	CAPTEscaffold_488_22752
Score total	:	1.5
Score for star read(s)	:	-1.3
Score for read counts	:	0
Score for mfe	:	1.8
Score for randfold	:	1.6
Score for cons. seed	:	-0.6
Total read count	:	630
Mature read count	:	490
Loop read count	:	0
Star read count	:	140

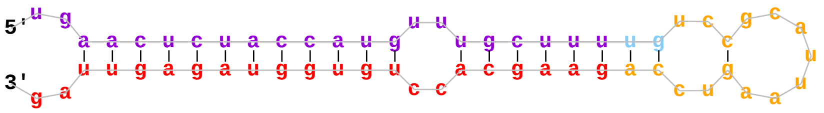

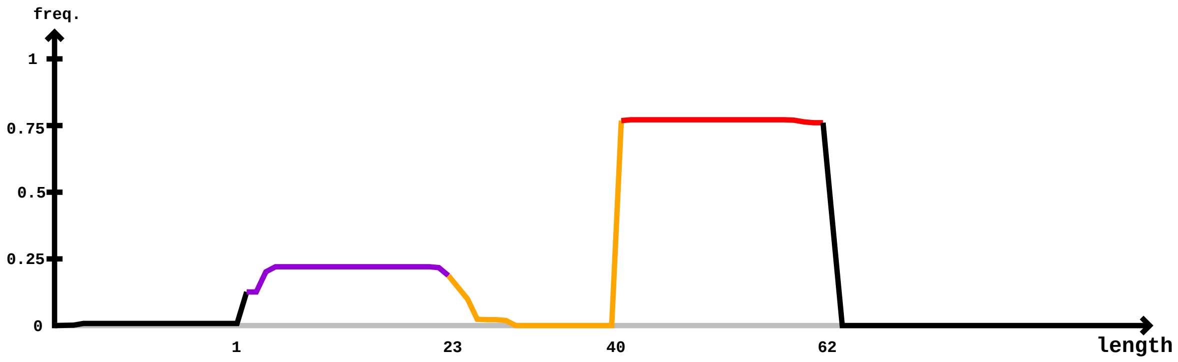


```
                    Star                                       Mature
5'- ucauguuuaaacucucccugaacucuaccauguuugcuuuuguccgcauuaaguccagaagcaccugugguagaguuaggggcggggucuaaaucagcuacagaugcaaaaa   -3'   obs
    ucauguuuaaacucucccugaacucuaccauguuugcuuuuguccgcauuaaguccagaagcaccugugguagaguuaggggcggggucuaaaucagcuacagaugcaaaaa         exp
    ....(((((.(((((((((.((((((((((((..((((((((..(.......)..))))))))..))))))))))))).)))).))))).))))).((.......)).....   reads   mm   sample
    ucauguuuaaacucucccug............................................................................................   1       0    seq
    .cauguuuaaacucucccug............................................................................................   4       0    seq
    ..................ugaacucuaccauguuugcu..........................................................................   2       0    seq
    ..................uAaacucuaccauguuugcuu.........................................................................   1       1    seq
    ..................ugaacucuaccauguuugcuu.........................................................................   17      0    seq
    ..................ugaacucuaGcauguuugcuu.........................................................................   1       1    seq
    ..................ugaacucuaccauguuugcuuu........................................................................   8       0    seq
    ..................ugaacucuaGcauguuugcuuu........................................................................   44      1    seq
    ..................ugaacucuaGcauguuugcuuuu.......................................................................   2       1    seq
    ....................aacucuaccauguuugcuuuu.......................................................................   2       0    seq
    ....................aacucuaccauguuugcuuuug......................................................................   45      0    seq
    ....................aacucuGccauguuugcuuuug......................................................................   1       1    seq
    ....................aacucuaGcauguuugcuuuug......................................................................   2       1    seq
    ....................aacucuaccauguuugcuuuugA.....................................................................   1       1    seq
    ....................aacucuaccauguuugcuuuugucc...................................................................   1       0    seq
    ....................aacucuaccauguuugcuuuuguccg..................................................................   1       0    seq
    .....................acucuaccauguuugcuuuugucc...................................................................   1       0    seq
    .....................acucuaccauguuugcuuuuguccg..................................................................   11      0    seq
    .........................................................gaagcaccugugguagag.....................................   1       0    seq
    .........................................................gaagcaccugugguagagu....................................   4       0    seq
    .........................................................gaagcaccugugguagaguu...................................   2       0    seq
    .........................................................gaagcaccugugguagaAuuag.................................   1       1    seq
    .........................................................gaagcaccugugguagaguuag.................................   445     0    seq
    .........................................................gaagcaccugugguaAaguuag.................................   2       1    seq
    .........................................................gaagcaccugugguUgaguuag.................................   1       1    seq
    .........................................................Uaagcaccugugguagaguuag.................................   5       1    seq
    .........................................................gaagcaccuguAguagaguuag.................................   2       1    seq
    .........................................................Aaagcaccugugguagaguuag.................................   1       1    seq
    .........................................................gaagcaccugugguaCaguuag.................................   1       1    seq
    .........................................................gaagcaccugugguagaguuagU................................   3       1    seq
    .........................................................gaagcaccugugguagaguuagA................................   20      1    seq
    ..........................................................aagcaccugugguagaguuag.................................   2       0    seq
```
