# Supplementary material for: The Small RNA Universe of Capitella teleta
Source: Front Mol Biosci. 2022 Feb 25;9:802814. doi: 10.3389/fmolb.2022.802814 (PMC8915122; doi:10.3389/fmolb.2022.802814)
Supplement: Supplementary file 1 [file DataSheet1.ZIP › Supplement/candidate/CAPTEscaffold_2_177.pdf]

Provisional ID	:	CAPTEscaffold_2_177
Score total	:	28.1
Score for star read(s)	:	3.9
Score for read counts	:	23.1
Score for mfe	:	0.1
Score for randfold	:	1.6
Score for cons. seed	:	-0.6
Total read count	:	57
Mature read count	:	50
Loop read count	:	0
Star read count	:	7

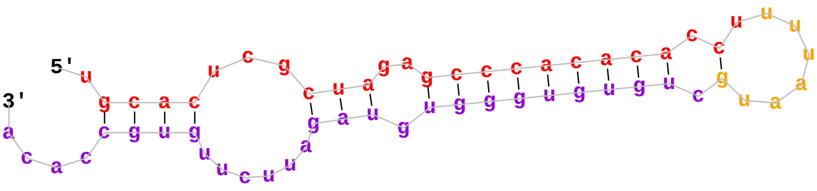

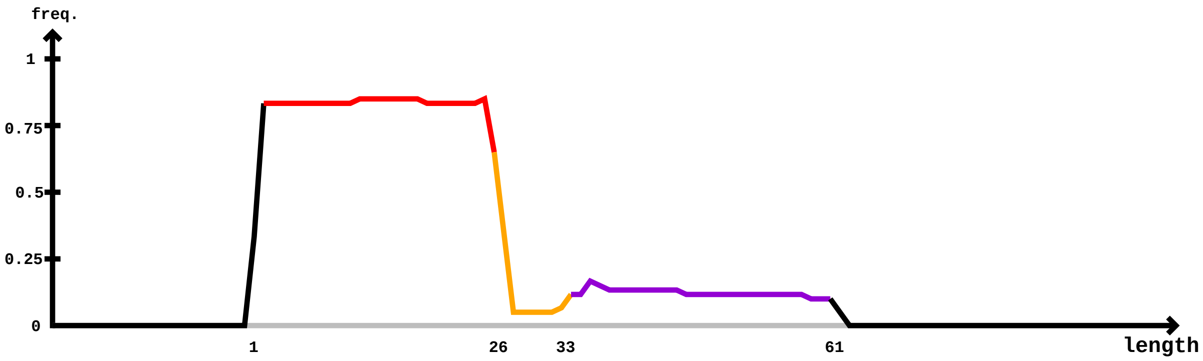


```
                      Mature                 Star
5'- aacauuaacaaaguuucgagugcacucgcuagagcccacacaccuuuuaaugcuguguggguguagauucuugugccacaguaauucagcaacuucuccccguuguuucauuauc   -3'    obs
    aacauuaacaaaguuucgagugcacucgcuagagcccacacaccuuuuaaugcuguguggguguagauucuugugccacaguaauucagcaacuucuccccguuguuucauuauc          exp
    ...................((((((...(((..(((((((((.(.......).))))))))).)))......))).))).(((((..((((((........))))))..))))).   reads   mm   sample
    ...................gugcacucgcuagagccc..............................................................................   2       0    seq
    ...................gugcacucgcuagagcccacacacc.......................................................................   5       0    seq
    ...................gGgcacucgcuagagcccacacacc.......................................................................   1       1    seq
    ...................gGgcacucgcuagagcccacacaccu......................................................................   1       1    seq
    ...................gugcacucgcuagagcccacacaccu......................................................................   11      0    seq
    ....................ugcacucgcuagagcccacacacc.......................................................................   6       0    seq
    ....................ugcacucgcuagagcccacacaccu......................................................................   24      0    seq
    ..............................agagcccacacaccuuuuaaugcugu...........................................................   1       0    seq
    .....................................acacaccuuuuaaugcug............................................................   1       0    seq
    ...........................................cuuuuaaugcuguguggugu...................................................   1       0    seq
    ...................................................gcuguguggguguagauucuugugccaca...................................   1       0    seq
    ....................................................cuguguggguguagauucuugugccaca...................................   3       0    seq
    ........................................................guggguguagauucuugugcc......................................   1       0    seq
    ........................................................guggguguagauucuugugccaca...................................   2       0    seq
```
