# Supplementary material for: The Small RNA Universe of Capitella teleta
Source: Front Mol Biosci. 2022 Feb 25;9:802814. doi: 10.3389/fmolb.2022.802814 (PMC8915122; doi:10.3389/fmolb.2022.802814)
Supplement: Supplementary file 1 [file DataSheet1.ZIP › Supplement/candidate/CAPTEscaffold_1308_33676.pdf]

Provisional ID	:	CAPTEscaffold_1308_33676
Score total	:	5562
Score for star read(s)	:	3.9
Score for read counts	:	5555.7
Score for mfe	:	1.4
Score for randfold	:	1.6
Score for cons. seed	:	-0.6
Total read count	:	10909
Mature read count	:	10908
Loop read count	:	0
Star read count	:	1

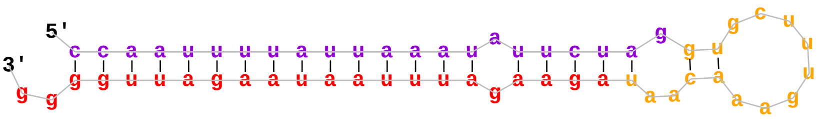

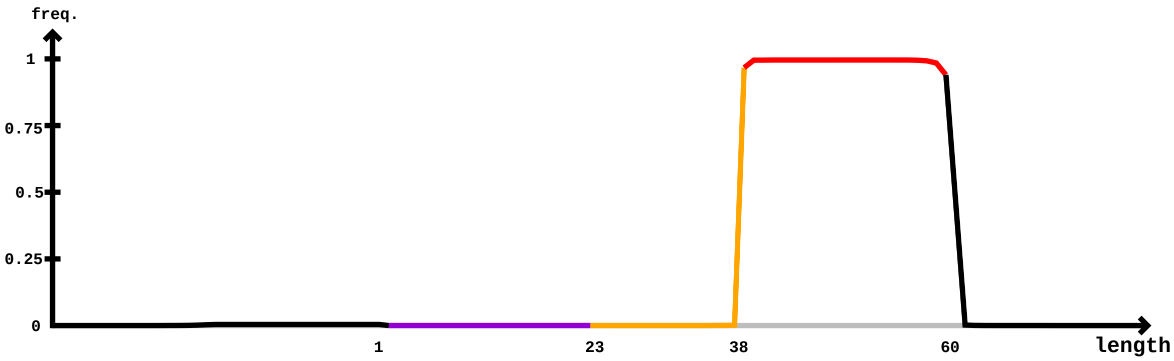


```
                                                    Star                                   Mature
5'- uuccauaaagaacaauuaguucgugccgcucgccaauuuuauuaaaauauucuaggugcuuugaaacaauagaagauuuaauaagauuggggaguugcucgacucgacgucg   -3'    obs
    uuccauaaagaacaauuaguucgugccgcucgccaauuuuauuaaaauauucuaggugcuuugaaacaauagaagauuuaauaagauuggggaguugcucgacucgacgucg          exp
    ..................((((((.((.((((.(((((((((((((((.(((((.((........))..))))).))))))))))))))).)))).)).)))...)))....   reads      mm        sample
    ..........aacaauuaguucgugccgcucg................................................................................   2          0         seq
    ...........acaauuaguucgugccgcucg................................................................................   1          0         seq
    ............caauuaguucgugccgcucg................................................................................   4          0         seq
    .............aauuaguucgugccgcucg................................................................................   9          0         seq
    ..............auuaguucgugccgcucg................................................................................   17         0         seq
    ...............uuaguucgugccgcucg................................................................................   12         0         seq
    ................................ccaauuuuauuaaaauauucuag.........................................................   1          0         seq
    ...................................................................aauagaagauuuaauaaga..........................   1          0         seq
    ...................................................................aauagaagauuuaauaagauug.......................   3          0         seq
    ...................................................................aauagaagauuuaauaagauugg......................   2          0         seq
    .....................................................................uagaagauuuaauaagauugg......................   1          0         seq
    .....................................................................uagaagauuuaauaagauuggg.....................   1          0         seq
    .....................................................................uagaagauuuaauaagauugggg....................   2          0         seq
    .....................................................................Cagaagauuuaauaagauugggg....................   3          1         seq
    ......................................................................agaagauuuaauaagauu........................   8          0         seq
    ......................................................................agaagauuuaauaagauug.......................   22         0         seq
    ......................................................................agaagauuuaGuaagauugg......................   1          1         seq
    ......................................................................agaagauuuaauaagauugg......................   86         0         seq
    ......................................................................agaagauuuGauaagauuggg.....................   1          1         seq
    ......................................................................agaagauuuaauaagauGggg.....................   1          1         seq
    ......................................................................agaagauuuaauaagauugAg.....................   1          1         seq
    ......................................................................agaagauAuaauaagauuggg.....................   1          1         seq
    ......................................................................Ggaagauuuaauaagauuggg.....................   16         1         seq
    ......................................................................agaagauuuaauaagauuCgg.....................   1          1         seq
    ......................................................................agaGgauuuaauaagauuggg.....................   1          1         seq
    ......................................................................agaagauuuaauaagauuggg.....................   460        0         seq
    ......................................................................agaagauuuaauaagauuAgg.....................   2          1         seq
    ......................................................................agaagauuuaauaagauuggUg....................   3          1         seq
    ......................................................................Cgaagauuuaauaagauugggg....................   1          1         seq
    ......................................................................agaagauuuaauaagauuAggg....................   41         1         seq
    ......................................................................agaagauuuaaGaagauugggg....................   1          1         seq
    ......................................................................agaagaAuuaauaagauugggg....................   10         1         seq
    ......................................................................agaagauuuaauaagauuUggg....................   1          1         seq
```

Star Mature

```
uuccauaaagaacaauuaguuucgugccgcucgccaauuuuauuaaaauauucuaggugcuuugaaacaauagaagauuuaauaagauugggg aguugcucgacucgacgucg
.......................................................................agaagauuGaauaagauugggg....................   1      1   seq
.......................................................................agaagauuuaauaagauuggAg....................   12     1   seq
.......................................................................agaagauuuaUuaagauugggg....................   2      1   seq
.......................................................................agaagauGuaauaagauugggg....................   2      1   seq
.......................................................................Ngaagauuuaauaagauugggg....................   3      1   seq
.......................................................................agaagauuuaauaagaAugggg....................   1      1   seq
.......................................................................agaagauuuaauaagauugggg....................   9290   0   seq
.......................................................................agaagauuuaauaagauugAgg....................   18     1   seq
.......................................................................agaagauuuaauaagGuugggg....................   1      1   seq
.......................................................................agaaAauuuaauaagauugggg....................   2      1   seq
.......................................................................agaagauuCaauaagauugggg....................   4      1   seq
.......................................................................agaagauuuaauaagauuggCg....................   1      1   seq
.......................................................................agaagauuuaauaagUuugggg....................   1      1   seq
.......................................................................agaagauuuaauGagauugggg....................   6      1   seq
.......................................................................agaagauuuaauaagauuCggg....................   1      1   seq
.......................................................................agaagauuuaauaagauugggA....................   26     1   seq
.......................................................................agaagauAuaauaagauugggg....................   8      1   seq
.......................................................................agaagauuuGauaagauugggg....................   8      1   seq
.......................................................................agaagauuuaauaagaGugggg....................   2      1   seq
.......................................................................agaagGuuuaauaagauugggg....................   1      1   seq
.......................................................................agaCgauuuaauaagauugggg....................   1      1   seq
.......................................................................agaagauuuaauaagauugggC....................   9      1   seq
.......................................................................agaagauuuaauaagauAgggg....................   5      1   seq
.......................................................................agaagauuuaaAaagauugggg....................   8      1   seq
.......................................................................aAaagauuuaauaagauugggg....................   4      1   seq
.......................................................................agaagauuuaauaUgauugggg....................   1      1   seq
.......................................................................agaagauuuaauaagauGgggg....................   1      1   seq
.......................................................................agaagauuuaauaGgauugggg....................   2      1   seq
.......................................................................Ugaagauuuaauaagauugggg....................   2      1   seq
.......................................................................agaagauuuaauaaAauugggg....................   2      1   seq
.......................................................................agaagauuuaauaaUauugggg....................   2      1   seq
.......................................................................agaagauuAaauaagauugggg....................   4      1   seq
.......................................................................agaagauuuaauaagauugUgg....................   5      1   seq
.......................................................................agaagauuuaauaagauCgggg....................   1      1   seq
.......................................................................Ggaagauuuaauaagauugggg....................   144    1   seq
.......................................................................agaagUuuuaauaagauugggg....................   2      1   seq
.......................................................................agaagauuuaauaagauugggU....................   11     1   seq
.......................................................................agaagauuuUauaagauugggg....................   1      1   seq
.......................................................................agaagauuuaauaagauuggggG...................   3      1   seq
.......................................................................aNaagauuuaauaagauugggga...................   1      1   seq
.......................................................................agaagauuuaauaagauugggga...................   79     0   seq
.......................................................................agaagauuuaauaagauuggggU...................   240    1   seq
.......................................................................agaagauuuaauaagauuggggC...................   1      1   seq
.......................................................................agaaAauuuaauaagauugggga...................   1      1   seq
.......................................................................agaagauuuaauaagauuggggaU..................   7      1   seq
.......................................................................agaagauuuaauaagauuggggaA..................   1      1   seq
.......................................................................agaagauuuaauaagauuggggaCu.................   1      1   seq
.......................................................................agaagauuuaauaagauuggggaUuu................   1      1   seq
........................................................................gaagauuuaauaagauuggg.....................   2      0   seq
........................................................................gaagauuuaauaagauugggg....................   51     0   seq
........................................................................Aaagauuuaauaagauugggg....................   1      1   seq
........................................................................gaagauuuaauaagauugggA....................   1      1   seq
........................................................................gaagauuuaauaagauugggU....................   1      1   seq
........................................................................gaagauuuaauaagauuAggg....................   1      1   seq
........................................................................gaagauuuaauaagauuggggG...................   1      1   seq
........................................................................gaagauuuaauaagauuggggU...................   225    1   seq
........................................................................gaagauuuaauaagauugggga...................   17     0   seq
........................................................................Aaagauuuaauaagauugggga...................   1      1   seq
........................................................................gaagauuuaauaagauuggggaA..................   2      1   seq
........................................................................gaagauuuaauaagauuggggaU..................   4      1   seq
.........................................................................aagauuuaauaagauuggggU...................   1      1   seq
..........................................................................agauuuaauaagauugggga...................   1      0   seq
..........................................................................agauuuaauaagauuggggagu.................   4      0   seq
.................................................................................auaagauuggggaguugcucga..........   1      0   seq
```
