# Supplementary material for: The Small RNA Universe of Capitella teleta
Source: Front Mol Biosci. 2022 Feb 25;9:802814. doi: 10.3389/fmolb.2022.802814 (PMC8915122; doi:10.3389/fmolb.2022.802814)
Supplement: Supplementary file 1 [file DataSheet1.ZIP › Supplement/candidate/CAPTEscaffold_563_23774.pdf]

Provisional ID	:	CAPTEscaffold_563_23774
Score total	:	357.8
Score for star read(s)	:	3.9
Score for read counts	:	351.9
Score for mfe	:	1
Score for randfold	:	1.6
Score for cons. seed	:	-0.6
Total read count	:	702
Mature read count	:	559
Loop read count	:	0
Star read count	:	143

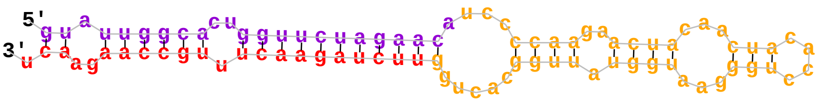

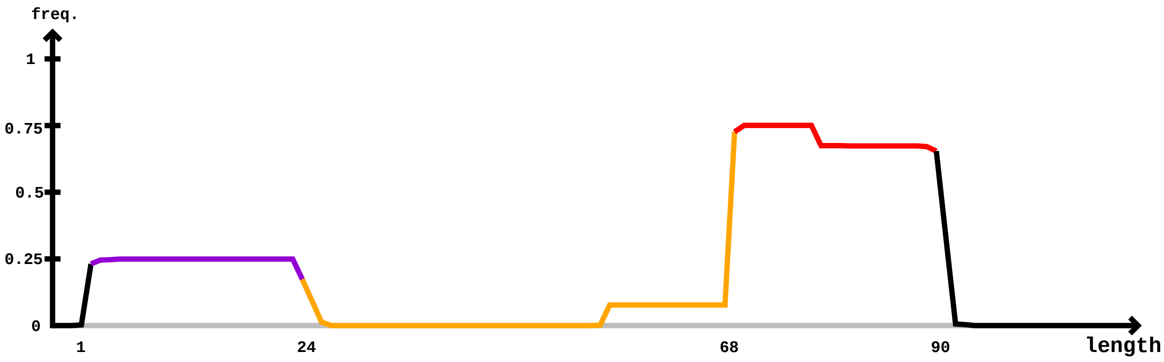

Star **Mature**

```
5'- ugguauuggcacugguucuagaacauccccaagaacuacaacuacaccugggaaugguauuggcacugguucuagaacuuugccaagaacuacaacuacaucugggaaugg  -3'  obs
    ugguauuggcacugguucuagaacauccccaagaacuacaacuacaccugggaaugguauuggcacugguucuagaacuuugccaagaacuacaacuacaucugggaaugg        exp
    ((((.((((((..(((((((((((....((((..((((...(((....)))...)))).)))).....))))))))))).))))))..))))...................   reads  mm  sample
    .gguaAuggcacugguucuagaac.......................................................................................   2      1   seq
    .gguauuggcacugguucuagaacauc....................................................................................   1      0   seq
    ..guauuggcacugguucuagaac.......................................................................................   124    0   seq
    ..Cuauuggcacugguucuagaaca......................................................................................   1      1   seq
    ..guauuggcacugguucuagaaca......................................................................................   95     0   seq
    ..Uuauuggcacugguucuagaaca......................................................................................   1      1   seq
    ..guauuggcacugguucuagaacaA.....................................................................................   16     1   seq
    ..guauuggcacugguucuagaacaC.....................................................................................   7      1   seq
    ..guauuggcacugguucuagaacau.....................................................................................   3      0   seq
    ..guauuggcacugguucuagaacauU....................................................................................   1      1   seq
    ..guauuggcacugguucuagaacauA....................................................................................   1      1   seq
    ..guauuggcacugguucuagaacaCc....................................................................................   3      1   seq
    ...uauuggcacugguucuagaaca......................................................................................   9      0   seq
    ...uauuggcacugguucuagaacaA.....................................................................................   1      1   seq
    ...uauuggcacugguucuagaacaCc....................................................................................   2      1   seq
    ....auuggcacugguucuagaacauc....................................................................................   1      0   seq
    .....uuggcacugguucuagaacauc....................................................................................   2      0   seq
    .......................................................gguaAuggcacugguucuagaac.................................   2      1   seq
    ........................................................guauuggcacugguucuagaac.................................   124    0   seq
    ........................................................guauuggcacugguucuagaacAuu..............................   1      1   seq
    .....................................................................uucuagaacuuugccaagaa......................   2      0   seq
    .....................................................................uucuagaacuuugccaagaac.....................   14     0   seq
    .....................................................................uAcuagaacuuugccaagaacu....................   1      1   seq
    .....................................................................uucuagaacuuugccaagaacu....................   501    0   seq
    .....................................................................uucuagaacuuuAccaagaacu....................   2      1   seq
    .....................................................................uucuagaacuuugccaagaacG....................   1      1   seq
    .....................................................................uucuagaacuuugccaagaaAu....................   1      1   seq
    .....................................................................uucuagaacuuugcAaagaacu....................   1      1   seq
    .....................................................................uucuagaacuuugccaagaacua...................   11     0   seq
    .....................................................................uucuagaacuuugccaagaacuaU..................   2      1   seq
    .....................................................................uucuagaacuuugccaagaacuaAa.................   3      1   seq
    ......................................................................ucuagaacuuugccaagaacu....................   19     0   seq
    ......................................................................ucuagaacuuugccaagaacua...................   1      0   seq
```

Star **Mature**

ugguauuggcacugguucuagaacaucccccaagaacuacaacuacaccugggaaugguauuggcacugguucuagaacuuugccaagaacuacaacuacaucugggaaugg
