# Supplementary material for: The Small RNA Universe of Capitella teleta
Source: Front Mol Biosci. 2022 Feb 25;9:802814. doi: 10.3389/fmolb.2022.802814 (PMC8915122; doi:10.3389/fmolb.2022.802814)
Supplement: Supplementary file 1 [file DataSheet1.ZIP › Supplement/candidate/CAPTEscaffold_644_25854.pdf]

Provisional ID	:	CAPTEscaffold_644_25854
Score total	:	12.9
Score for star read(s)	:	3.9
Score for read counts	:	7.3
Score for mfe	:	0.7
Score for randfold	:	1.6
Score for cons. seed	:	-0.6
Total read count	:	26
Mature read count	:	22
Loop read count	:	0
Star read count	:	4

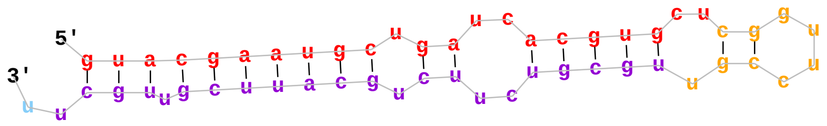

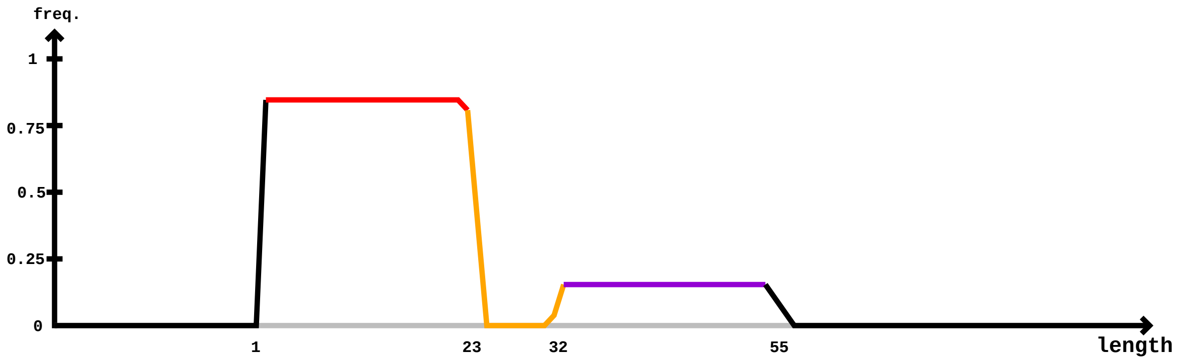

Mature Star

```
5'- uuguugauugccucauuaauguacgaaugcugaucacgugcucgguuccguugcgucuucugcauucguugcuuaauuuggcaagugugcagcauauuccgccguuuuaaga   -3'    obs
    uuguugauugccucauuaauguacgaaugcugaucacgugcucgguuccguugcgucuucugcauucguugcuuaauuuggcaagugugcagcauauuccgccguuuuaaga          exp
    ((((...(((((..(((((.((((((((((.((..(((((..((....)).)))))..)).))))))).))))))))..)))))....))))....................  reads   mm   sample
    ....................guacgaaugcugaucacgugc.......................................................................  1       0    seq
    ....................guacgaaugcugaucacgugcu......................................................................  21      0    seq
    ..................................................uugcgucuucugcauucguugcu.......................................  1       0    seq
    ...................................................ugcgucuucugcauucguugcu.......................................  3       0    seq
```
