# Supplementary material for: The Small RNA Universe of Capitella teleta
Source: Front Mol Biosci. 2022 Feb 25;9:802814. doi: 10.3389/fmolb.2022.802814 (PMC8915122; doi:10.3389/fmolb.2022.802814)
Supplement: Supplementary file 1 [file DataSheet1.ZIP › Supplement/candidate/CAPTEscaffold_530_24266.pdf]

Provisional ID	:	CAPTEscaffold_530_24266
Score total	:	972.8
Score for star read(s)	:	3.9
Score for read counts	:	965.2
Score for mfe	:	2.7
Score for randfold	:	1.6
Score for cons. seed	:	-0.6
Total read count	:	1905
Mature read count	:	1892
Loop read count	:	0
Star read count	:	13

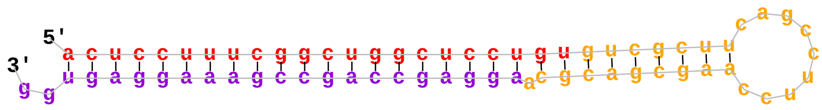

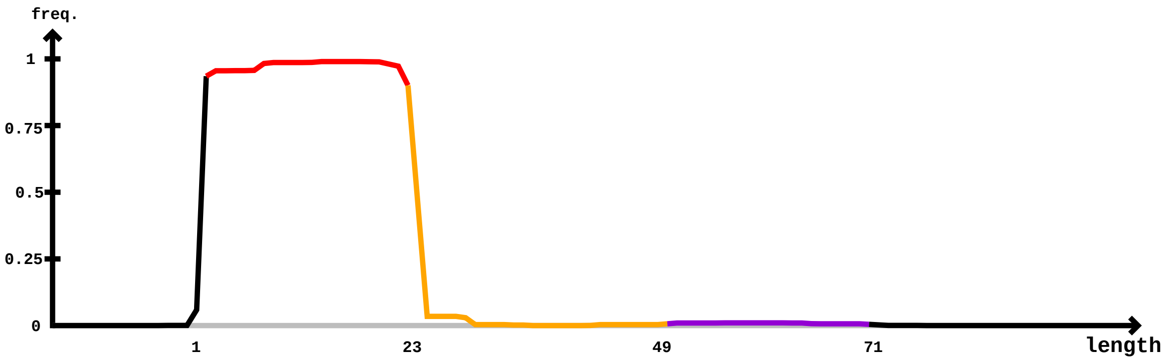

Mature Star

```
5'- gccaaauacuugucacuccuuucggcuggcuccugugucgcuucagccuuccaagcgacgcaaggagccagccgaaaggaguggcacgguuacauugugacuaguuuuaug   -3'   obs
    gccaaauacuugucacuccuuucggcuggcuccugugucgcuucagccuuccaagcgacgcaaggagccagccgaaaggaguggcacgguuacauugugacuaguuuuaug         exp
    ..........(((((((((((((((((((((((((((((((((.........))))))))).)))))))))))))))))))))))).((((((...)))))).........   reads   mm   sample
    ..........ugucacuccuuucgCcuggcuc...............................................................................   1       1    seq
    .............cGcuccuuucggcuggcu................................................................................   1       1    seq
    .............cacuccuuucggcuggcucc..............................................................................   1       0    seq
    .............cacuccuuucggcuggcuccC.............................................................................   1       1    seq
    .............cacuccuuucggcuUgcuccu.............................................................................   2       1    seq
    .............cacuccuuucggcuggcuccu.............................................................................   2       0    seq
    .............cacuccuuucggcuggcuccug............................................................................   10      0    seq
    .............cacuccuuucgAcuggcuccugu...........................................................................   98      1    seq
    ..............Ccuccuuucggcuggcucc..............................................................................   1       1    seq
    ..............acuccuuucggcuggcucc..............................................................................   7       0    seq
    ..............acuccuuucggcuggcuccu.............................................................................   2       0    seq
    ..............acuccuuucggcuggcuccGg............................................................................   1       1    seq
    ..............acuccuuuUggcuggcuccug............................................................................   1       1    seq
    ..............acuccuuucggcuggcuccuA............................................................................   3       1    seq
    ..............acuccuuucggcuggcuccug............................................................................   102     0    seq
    ..............acuccuuucgCcuggcuccug............................................................................   1       1    seq
    ..............acuccuuucgAcuggcuccugu...........................................................................   1608    1    seq
    ..............acuccuuucggcuggcuccugu...........................................................................   1       0    seq
    ...............cuccuuucggcuggcucc..............................................................................   7       0    seq
    ...............cuccCuucggcuggcuccu.............................................................................   1       1    seq
    ...............cuccuuucggcuggcuccu.............................................................................   8       0    seq
    ...............cuccuuucggcuggcuccug............................................................................   22      0    seq
    ...............Guccuuucggcuggcuccug............................................................................   1       1    seq
    ...............cuccuuucggcuggAuccugugucgcu.....................................................................   1       1    seq
    .................ccuuucggcuggcuccug............................................................................   1       0    seq
    ...................uuucggcuggcuccugugucgc......................................................................   2       0    seq
    ....................uucggcuggcuccugugucgc......................................................................   8       0    seq
    ....................uuUggcuggcuccugugucgcu.....................................................................   10      1    seq
    ....................Cucggcuggcuccugugucgcu.....................................................................   1       1    seq
    ....................uucgAcuggcuccugugucgcu.....................................................................   1       1    seq
    ....................uucggcuggcuccugugucgcu.....................................................................   31      0    seq
    .....................ucggcuggcuccugugucgcu.....................................................................   7       0    seq
    .........................cuggcuccugugucgcuucag.................................................................   1       0    seq
```

```
              Mature                                              Star
gccaaauacuugucacuccuuucggcuggcuccugugucgcuucagccuuccaagcgacgcaaggagccagccgaaaggaguggcacgguuacauugugacuaguuuuaug
..........................uggcuccugugucgcuucag.................................................................   2   0   seq
..........................uggcuccugugucgcuucagcc...............................................................   4   0   seq
......................................................gcgacgcaaggagccagccgU....................................   1   1   seq
.......................................................cgacgcaaggagccagccgaaa..................................   5   0   seq
.......................................................cgacgcaaggagccagccgaaag.................................   1   0   seq
..............................................................aggagccagccgaaaggagug............................   1   0   seq
..............................................................aggagccagccgaaaggagugg...........................   5   0   seq
...............................................................ggagccagccgaaaggagug............................   3   0   seq
...............................................................ggagccagccgaaaggagugg...........................   2   0   seq
...............................................................ggagccagccgaaaggaguggU..........................   1   1   seq
....................................................................cagccgaaaggaguggcacgg......................   1   0   seq
```
