# Supplementary material for: The Small RNA Universe of Capitella teleta
Source: Front Mol Biosci. 2022 Feb 25;9:802814. doi: 10.3389/fmolb.2022.802814 (PMC8915122; doi:10.3389/fmolb.2022.802814)
Supplement: Supplementary file 1 [file DataSheet1.ZIP › Supplement/candidate/CAPTEscaffold_377_19163.pdf]

Provisional ID	:	CAPTEscaffold_377_19163
Score total	:	199.6
Score for star read(s)	:	3.9
Score for read counts	:	192.8
Score for mfe	:	1.9
Score for randfold	:	1.6
Score for cons. seed	:	-0.6
Total read count	:	390
Mature read count	:	386
Loop read count	:	0
Star read count	:	4

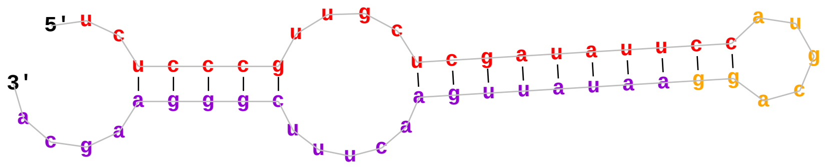

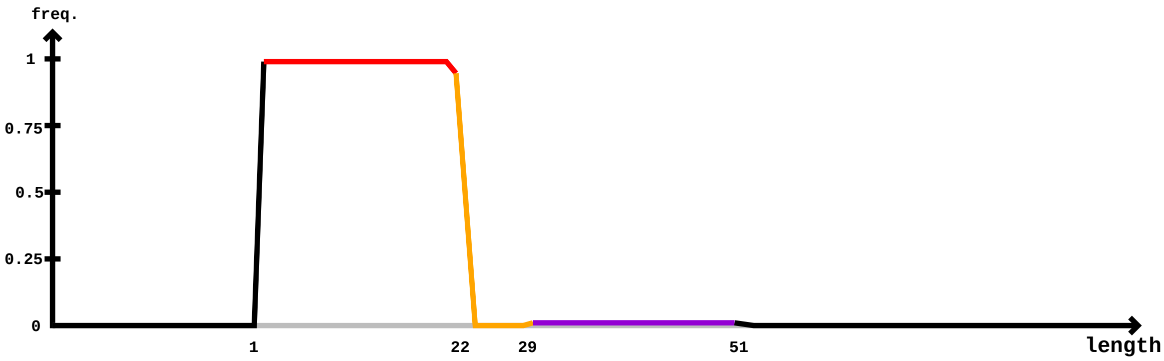

Mature **Star**

```
5'- aaagcgugcgaaucacggugucucccguugcucgauauuccaugcaggaauauugaacuuucgggaagcacugugaccugauuggucggcaacaaaguaaccgagaucgcu  -3'  obs
    aaagcgugcgaaucacggugucucccguugcucgauauuccaugcaggaauauugaacuuucgggaagcacugugaccugauuggucggcaacaaaguaaccgagaucgcu       exp
    ..((((..(...(((((((((.(((((....((((((((((.....)))))))))).....))))).)))))))))((.....))((((..((...))..)))))..))))  reads  mm  sample
    ....................ucucccguugcucgauauuc.......................................................................  17  0  seq
    ....................ucucccguugcucgauaAucc......................................................................  1   1  seq
    ....................ucucccguugcuAgauauucc......................................................................  1   1  seq
    ....................ucAcccguugcucgauauucc......................................................................  1   1  seq
    ....................ucucccguugcucgauauAcc......................................................................  1   1  seq
    ....................ucucccguugcuUgauauucc......................................................................  1   1  seq
    ....................ucucccguugcucgauauucc......................................................................  352 0  seq
    ....................ucucccguugcucAauauucc......................................................................  1   1  seq
    ....................ucucccguugAucgauauucca.....................................................................  1   1  seq
    ....................ucucccguugcucgauauuccU.....................................................................  2   1  seq
    ....................ucucccguugcucgauauucca.....................................................................  8   0  seq
    ................................................aauauugaacuuucgggaagca.........................................  4   0  seq
```
