# Supplementary material for: The Small RNA Universe of Capitella teleta
Source: Front Mol Biosci. 2022 Feb 25;9:802814. doi: 10.3389/fmolb.2022.802814 (PMC8915122; doi:10.3389/fmolb.2022.802814)
Supplement: Supplementary file 1 [file DataSheet1.ZIP › Supplement/candidate/CAPTEscaffold_377_19161.pdf]

Provisional ID	:	CAPTEscaffold_377_19161
Score total	:	24
Score for star read(s)	:	3.9
Score for read counts	:	18
Score for mfe	:	1.2
Score for randfold	:	1.6
Score for cons. seed	:	-0.6
Total read count	:	47
Mature read count	:	46
Loop read count	:	0
Star read count	:	1

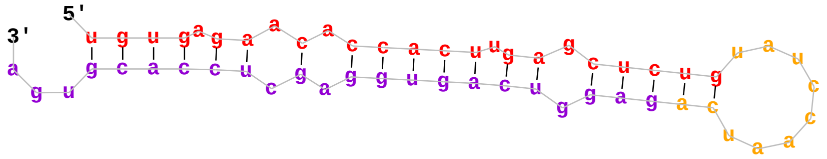

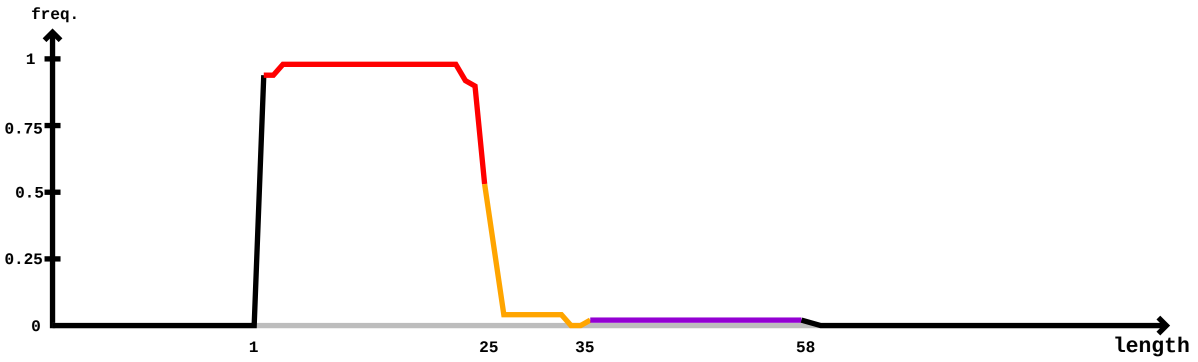

Mature Star

```
5'- gucagauguuggucucuauuugugagaacaccacuugagcucuguauccaaucagaggucaguggagcuccacgugauuacggccaucagguuucaaagcgaaagaaacugaau   -3'    obs
    gucagauguuggucucuauuugugagaacaccacuugagcucuguauccaaucagaggucaguggagcuccacgugauuacggccaucagguuucaaagcgaaagaaacugaau          exp
    ......((.(((((...(((((((.((.(.(((((.((.(((((........))))).))))))).).)))))).)))...))))).))((((((.........))))))....    reads      mm         sample
    ....................ugugagaacaccacuugagcu.........................................................................    3          0          seq
    ....................ugugagaacaccacuugagcuc........................................................................    1          0          seq
    ....................ugugUgaacaccacuugagcucu.......................................................................    1          1          seq
    ....................Ggugagaacaccacuugagcucu.......................................................................    1          1          seq
    ....................ugugagaacaccacuugagcucu.......................................................................    16         0          seq
    ....................ugugagaacaccacuugagcucug......................................................................    23         0          seq
    ....................Ggugagaacaccacuugagcucug......................................................................    1          1          seq
    ......................ugagaacaccacuugagcucuguauccaau..............................................................    2          0          seq
    ......................................................gaggucaguggagcuccacgugG.....................................    1          1          seq
```
