# Supplementary material for: The Small RNA Universe of Capitella teleta
Source: Front Mol Biosci. 2022 Feb 25;9:802814. doi: 10.3389/fmolb.2022.802814 (PMC8915122; doi:10.3389/fmolb.2022.802814)
Supplement: Supplementary file 1 [file DataSheet1.ZIP › Supplement/candidate/CAPTEscaffold_328_19497.pdf]

Provisional ID	:	CAPTEscaffold_328_19497
Score total	:	12.7
Score for star read(s)	:	3.9
Score for read counts	:	5.7
Score for mfe	:	2.1
Score for randfold	:	1.6
Score for cons. seed	:	-0.6
Total read count	:	23
Mature read count	:	21
Loop read count	:	0
Star read count	:	2

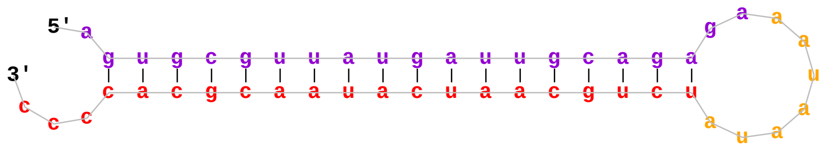

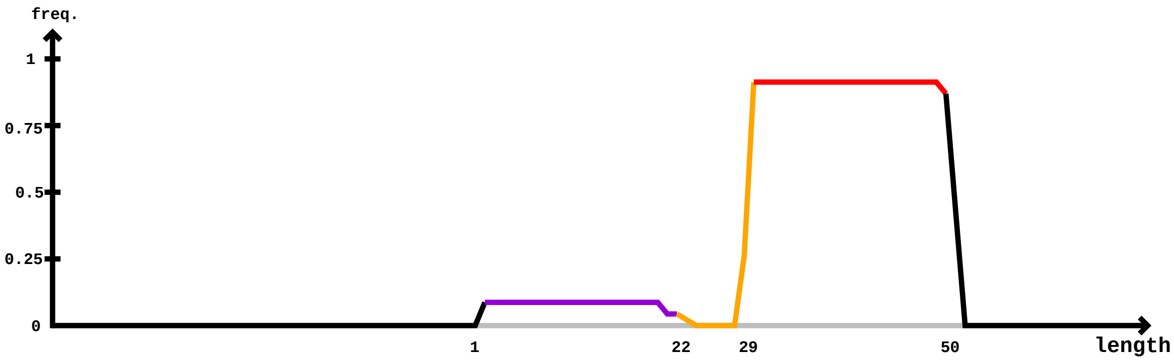


```
                                                           Star                          Mature
5'- acgcgcgucucaucgauccacggagagggacgcuucguaauauagugcguuaugauugcagagaaauaauaucugcaaucauaacgcaccccaagaggaagcaacacuucgc   -3'    obs
    acgcgcgucucaucgauccacggagagggacgcuucguaauauagugcguuaugauugcagagaaauaauaucugcaaucauaacgcaccccaagaggaagcaacacuucgc          exp
    (((.(((((((.((........))..)))))))..)))......((((((((((((((((((.........))))))))))))))))))........((((.....))))..    reads    mm    sample
    ...........................................agugcguuaugauugcaga..................................................    1        0     seq
    ...........................................agugcguuaugauugcagaga................................................    1        0     seq
    ......................................................................aucugcaaucauaacgcacccc....................    6        0     seq
    .......................................................................ucugcaaucauaacgcaccc.....................    1        0     seq
    .......................................................................ucugcaaucauaacgcGcccc....................    1        1     seq
    .......................................................................ucugcaaucauaacgcacccc....................    7        0     seq
    .......................................................................ucugcaaucauaacgcacccA....................    1        1     seq
    .......................................................................ucugcaaucauaacgcacccca...................    5        0     seq
```
