# Supplementary material for: The Small RNA Universe of Capitella teleta
Source: Front Mol Biosci. 2022 Feb 25;9:802814. doi: 10.3389/fmolb.2022.802814 (PMC8915122; doi:10.3389/fmolb.2022.802814)
Supplement: Supplementary file 1 [file DataSheet1.ZIP › Supplement/candidate/CAPTEscaffold_13771_44694.pdf]

Provisional ID	:	CAPTEscaffold_13771_44694
Score total	:	6
Score for star read(s)	:	3.9
Score for read counts	:	-0.3
Score for mfe	:	1.4
Score for randfold	:	1.6
Score for cons. seed	:	-0.6
Total read count	:	11
Mature read count	:	10
Loop read count	:	0
Star read count	:	1

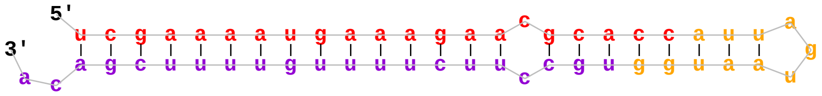

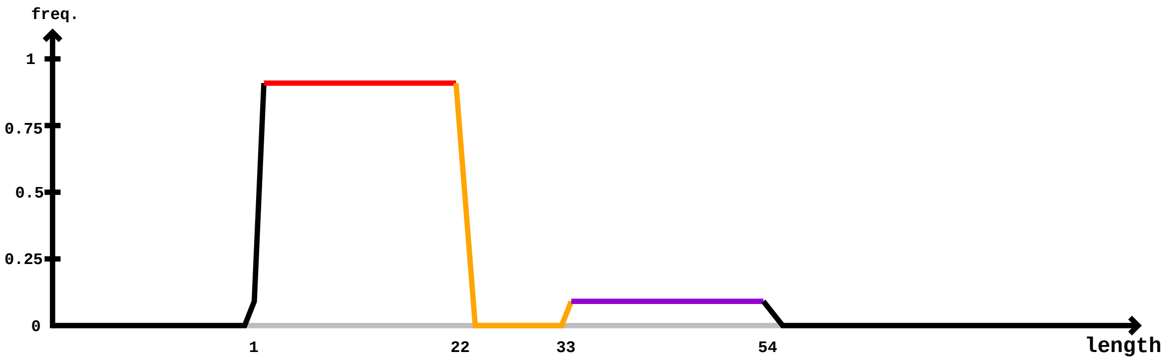

Mature Star

```
5'- gaucauguuauucagaguugucgaaaaugaaagaacgcaccauuaguaauggugccuucuuuuguuuucgacauuacucauaagucuuucucuuucaacuacuguucuacu   -3'   obs
    gaucauguuauucagaguugucgaaaaugaaagaacgcaccauuaguaauggugccuucuuuuguuuucgacauuacucauaagucuuucucuuucaacuacuguucuacu         exp
    ..............(((((((((((((((((((((.((((((((...)))))))).)))))))))))))))))..))))................................   reads   mm   sample
    ...................gucgaGaaugaaagaacgcacca.....................................................................   1       1    seq
    ....................ucgaaaaugaaagaacgcacc......................................................................   9       0    seq
    ....................................................ugcGuucuuuuguuuucgaca......................................   1       1    seq
```
