# Supplementary material for: The Small RNA Universe of Capitella teleta
Source: Front Mol Biosci. 2022 Feb 25;9:802814. doi: 10.3389/fmolb.2022.802814 (PMC8915122; doi:10.3389/fmolb.2022.802814)
Supplement: Supplementary file 1 [file DataSheet1.ZIP › Supplement/candidate/CAPTEscaffold_12304_44295.pdf]

Provisional ID	:	CAPTEscaffold_12304_44295
Score total	:	9508.3
Score for star read(s)	:	3.9
Score for read counts	:	9505.8
Score for mfe	:	1.4
Score for randfold	:	-2.2
Score for cons. seed	:	-0.6
Total read count	:	18657
Mature read count	:	18640
Loop read count	:	0
Star read count	:	17

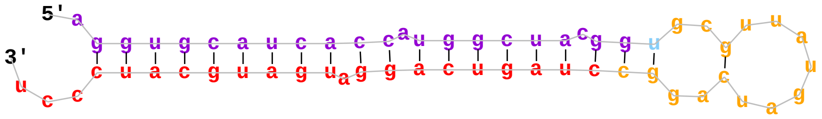

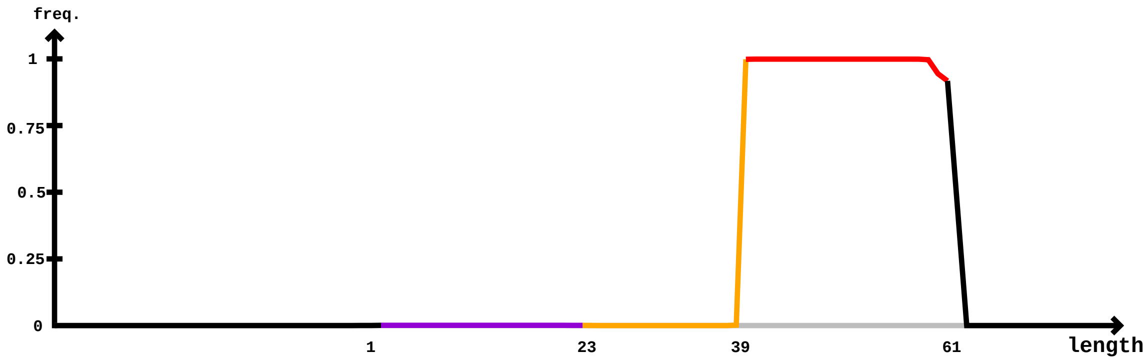

Star Mature

```
5'- aaucgagaacugaccgucagcuucagcgacagaggugcaucaccauggcuacggugcguuaugaucaggccuagucaggaugaugcaucccugcuucugcugcuucuac  -3'   obs
    aaucgagaacugaccgucagcuucagcgacagaggugcaucaccauggcuacggugcguuaugaucaggccuagucaggaugaugcaucccugcuucugcugcuucuac        exp
    ......((((((...)))).)))((((.(((((((((((((((.((((((.(((..(.......)..))))))))))).))))))......))))))).)))).....    reads   mm   sample
    ..............................agaggugcaucaccauggcuac.........................................................    5       0    seq
    ................................aggugcaucaccauggcuacg........................................................    1       0    seq
    ................................aggugcaucaccauggcuacgg.......................................................    7       0    seq
    ................................aggugcaucaccauggcuacggu......................................................    4       0    seq
    .....................................................................ccuagucaggaugaugcaucc...................    1       0    seq
    .....................................................................ccuagucaggaugaugcauccc..................    7       0    seq
    .....................................................................ccuagucaggaugaugcaucccu.................    21      0    seq
    ......................................................................cuagucaggaugaugcau.....................    2       0    seq
    ......................................................................cuagucaggaugaugcauc....................    40      0    seq
    ......................................................................cuagucaggaugaGgcauc....................    1       1    seq
    ......................................................................cuagucaggaugaugUaucc...................    1       1    seq
    ......................................................................Nuagucaggaugaugcaucc...................    1       1    seq
    ......................................................................cuaguUaggaugaugcaucc...................    1       1    seq
    ......................................................................Guagucaggaugaugcaucc...................    1       1    seq
    ......................................................................cuagucaggaugaugcaucc...................    961     0    exp
    ......................................................................cuagucaAgaugaugcaucc...................    2       1    seq
    ......................................................................cAagucaggaugaugcaucc...................    2       1    seq
    ......................................................................cuagucaggaugaugcaucU...................    7       1    seq
    ......................................................................cuagAcaggaugaugcauccc..................    1       1    seq
    ......................................................................cuagucaggauAaugcauccc..................    1       1    seq
    ......................................................................cuagucaggaugaugcauccc..................    488     0    seq
    ......................................................................cuagucaggaugaugcaAccc..................    1       1    seq
    ......................................................................cuagucaggaugaugcauccU..................    3       1    seq
    ......................................................................cuagucaAgaugaugcauccc..................    1       1    seq
    ......................................................................cuagucaggaugaugcGuccc..................    1       1    seq
    ......................................................................cAagucaggaugaugcauccc..................    1       1    seq
    ......................................................................cuagucaggaugaugcaucccG.................    116     1    seq
    ......................................................................cuagucaggUugaugcaucccu.................    2       1    seq
    ......................................................................cuagucaggaugaugcaucUcu.................    16      1    seq
    ......................................................................cuagucaggaugaugcaucGcu.................    3       1    seq
    ......................................................................cuagucaggaugaugcauAccu.................    1       1    seq
    ......................................................................cuagucaggaugGugcaucccu.................    4       1    seq
    ......................................................................cuagucaggaugaugcaAcccu.................    6       1    seq
```

Star   Mature

aaucgagaacugaccgucagcuucagcgacagaggugcaucaccauggcuacggugcguuaugaucaggccuagucaggaugaugcaucccugcuucugcugcuucuac			
....cuagucaggaugaugcauccUu.................	5	1	seq
....cuagucaggauAaugcaucccu.................	14	1	seq
....cuagAcaggaugaugcaucccu.................	13	1	seq
....cuagucaggaCgaugcaucccu.................	3	1	seq
....cuagucaggaugauUcaucccu.................	3	1	seq
....cuaguUaggaugaugcaucccu.................	7	1	seq
....cuagucaggaugaugcauccсN.................	1	1	seq
....cuaguAaggaugaugcaucccu.................	1	1	seq
....cAagucaggaugaugcaucccu.................	15	1	seq
....cuagucaggauUaugcaucccu.................	2	1	seq
....cuagucaggaugUugcaucccu.................	4	1	seq
....Auagucaggaugaugcaucccu.................	7	1	seq
....cuagucaCgaugaugcaucccu.................	3	1	seq
....cuagucCggaugaugcaucccu.................	1	1	seq
....cuagucaggNugaugcaucccu.................	1	1	seq
....Uuagucaggaugaugcaucccu.................	6	1	seq
....cuagucaggaugaugcauccAu.................	3	1	seq
....cuagCcaggaugaugcaucccu.................	2	1	seq
....cuagucaggaugaugcaucccu.................	16614	0	seq
....cuagucaggaugaugcaCcccu.................	3	1	seq
....cuagucaggaugaugcaucccC.................	6	1	seq
....cuagucaggaugaCgcaucccu.................	5	1	seq
....cuagucaggaugaugcGucccu.................	5	1	seq
....cuagucaUgaugaugcaucccu.................	1	1	seq
....cuagucaggGugaugcaucccu.................	3	1	seq
....cuagGcaggaugaugcaucccu.................	1	1	seq
....cuagucGggaugaugcaucccu.................	10	1	seq
....cuagucaggaugaugcaucAcu.................	19	1	seq
....cuagucaggaugaugAaucccu.................	5	1	seq
....cuagucaggaugaugcauUccu.................	1	1	seq
....cuagucaggaugCugcaucccu.................	2	1	seq
....cuagucaggaugaugcaucccA.................	68	1	seq
....cuagucaggauCaugcaucccu.................	2	1	seq
....cuGgucaggaugaugcaucccu.................	3	1	seq
....cuagucaggaugaugGaucccu.................	2	1	seq
....cuagucaggaugaugcUucccu.................	3	1	seq
....cuagucagCaugaugcaucccu.................	1	1	seq
....cuagucaggaugaAgcaucccu.................	6	1	seq
....cuagucagUaugaugcaucccu.................	1	1	seq
....cuagucaggaugaugcauccGu.................	1	1	seq
....cuagucaAgaugaugcaucccu.................	33	1	seq
....Guagucaggaugaugcaucccu.................	1	1	seq
....cuagucaggaugaugcaGcccu.................	2	1	seq
....cuagucaggaAgaugcaucccu.................	5	1	seq
....cuagucaggaugauAcaucccu.................	2	1	seq
....cuagucaggaugaugUaucccu.................	2	1	seq
....cuagucaggaugauCcaucccu.................	1	1	seq
....Nuagucaggaugaugcaucccu.................	4	1	seq
....cuagucUggaugaugcaucccu.................	1	1	seq
....cuagucaggaugaugcaucccug................	2	0	seq
....cuagucaggaugaugcaucccuA................	15	1	seq
....cuagucaggaugaugcaucccuU................	15	1	seq
....uagucaggaugaugcaucccu.................	11	0	seq
