# Supplementary material for: The Small RNA Universe of Capitella teleta
Source: Front Mol Biosci. 2022 Feb 25;9:802814. doi: 10.3389/fmolb.2022.802814 (PMC8915122; doi:10.3389/fmolb.2022.802814)
Supplement: Supplementary file 1 [file DataSheet1.ZIP › Supplement/candidate/CAPTEscaffold_650_25107.pdf]

Provisional ID	:	CAPTEscaffold_650_25107
Score total	:	8.2
Score for star read(s)	:	3.9
Score for read counts	:	1.7
Score for mfe	:	1.7
Score for randfold	:	1.6
Score for cons. seed	:	-0.6
Total read count	:	15
Mature read count	:	13
Loop read count	:	0
Star read count	:	2

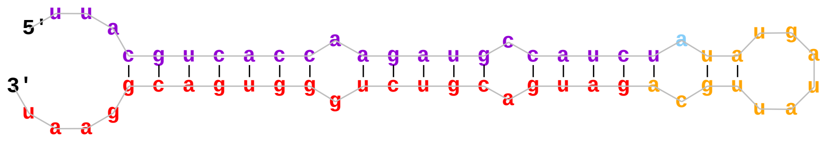

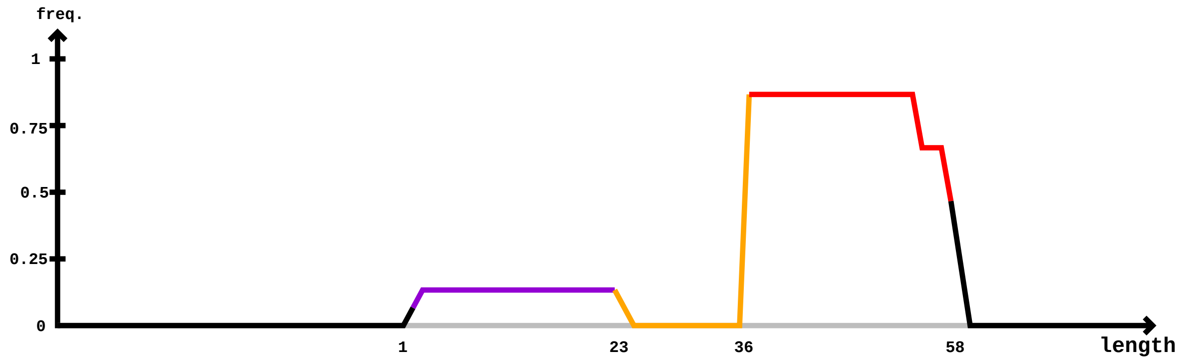


```
                                       Star                                    Mature
5'- auucacugcggagaaauuacuaaugcacaaacagauuacgucaccaagaugccaucuauaugauauugcagaugacgucugggugacggaauuugugcgcaauaucuccaaa   -3'    obs
    auucacugcggagaaauuacuaaugcacaaacagauuacgucaccaagaugccaucuauaugauauugcagaugacgucugggugacggaauuugugcgcaauaucuccaaa          exp
    .........(((((......((.(((.(..((((((..(((((((.(((((.(((((.((......)).))))).))))).)))))))..))))))).))).)))))))...   reads      mm         sample
    ...................................uuacgucaccaagaugccaucu.......................................................   1          0          seq
    ....................................uacgucaccaagaugccaucu.......................................................   1          0          seq
    ......................................................................gaugacgucugggugacg........................   3          0          seq
    ......................................................................gaugacgucugggugacAgaa.....................   3          1          seq
    ......................................................................gaugacgucugggugacAgaau....................   2          1          seq
    ......................................................................gaugacgucugggugacggaau....................   5          0          seq
```
