# Supplementary material for: The Small RNA Universe of Capitella teleta
Source: Front Mol Biosci. 2022 Feb 25;9:802814. doi: 10.3389/fmolb.2022.802814 (PMC8915122; doi:10.3389/fmolb.2022.802814)
Supplement: Supplementary file 1 [file DataSheet1.ZIP › Supplement/candidate/CAPTEscaffold_17_2729.pdf]

Provisional ID	:	CAPTEscaffold_17_2729
Score total	:	1616.8
Score for star read(s)	:	3.9
Score for read counts	:	1610.7
Score for mfe	:	1.3
Score for randfold	:	1.6
Score for cons. seed	:	-0.6
Total read count	:	3171
Mature read count	:	3164
Loop read count	:	2
Star read count	:	5

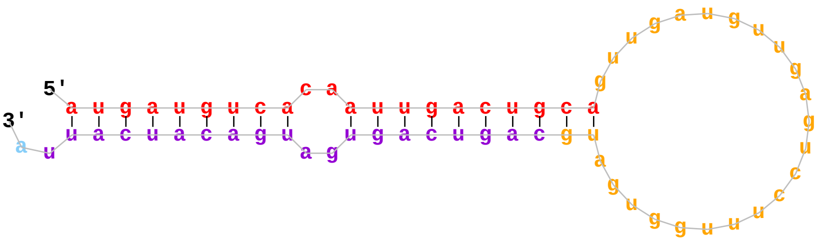

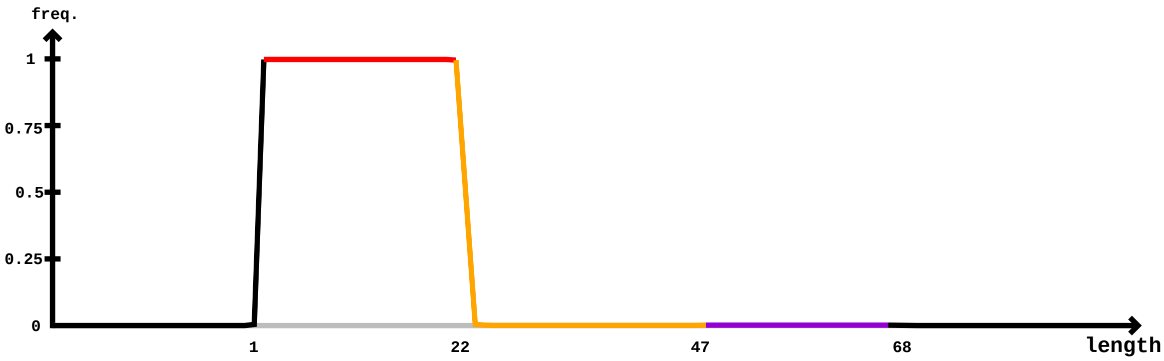

Mature **Star**

```
5'- auguccuauguaauaugugaaugaugucacaauugacugcaguugauguugaguccuuuggugaugcagucagugaugacaucauuacaauauugcucucuuacgucaugu  -3'  obs
    auguccuauguaauaugugaaugaugucacaauugacugcaguugauguugaguccuuuggugaugcagucagugaugacaucauuacaauauugcucucuuacgucaugu       exp
    ((((.....(((((((...((((((((((..((((((((((......................))))))))))..))))))))))...)))))))......)))).....  reads  mm  sample
    ...................aaugaugucacaauugacugca......................................................................  13     0   seq
    ...................aaugaugucacaauugacugcU......................................................................  1      1   seq
    ....................augaugucacaauugacugc.......................................................................  8      0   seq
    ....................augaugucacaauuAacugca......................................................................  1      1   seq
    ....................augaugucacaauCgacugca......................................................................  2      1   seq
    ....................augauUucacaauugacugca......................................................................  2      1   seq
    ....................augaugucacaauugacugca......................................................................  3039   0   seq
    ....................augaugucaAaauugacugca......................................................................  2      1   seq
    ....................augaugucacaauugacugcU......................................................................  3      1   seq
    ....................augaugucacGauugacugca......................................................................  1      1   seq
    ....................augauAucacaauugacugca......................................................................  1      1   seq
    ....................augaugucacaauugacugAa......................................................................  1      1   seq
    ....................augaugucacaauugacuCca......................................................................  2      1   seq
    ....................Nugaugucacaauugacugca......................................................................  4      1   seq
    ....................augaugucacaauugacuAca......................................................................  1      1   seq
    ....................Uugaugucacaauugacugca......................................................................  2      1   seq
    ....................augaugucGcaauugacugca......................................................................  1      1   seq
    ....................Gugaugucacaauugacugca......................................................................  1      1   seq
    ....................augaugucaGaauugacugca......................................................................  1      1   seq
    ....................aAgaugucacaauugacugca......................................................................  4      1   seq
    ....................augauguAacaauugacugca......................................................................  1      1   seq
    ....................augauguUacaauugacugca......................................................................  1      1   seq
    ....................augaugGcacaauugacugca......................................................................  1      1   seq
    ....................aNgaugucacaauugacugca......................................................................  1      1   seq
    ....................augaugAcacaauugacugca......................................................................  2      1   seq
    ....................augaugucacaauugacCgca......................................................................  1      1   seq
    ....................augaugucacaauugacuUca......................................................................  1      1   seq
    ....................auAaugucacaauugacugca......................................................................  5      1   seq
    ....................augaugucacaauAgacugca......................................................................  1      1   seq
    ....................augaugucUcaauugacugca......................................................................  1      1   seq
    ....................augaAgucacaauugacugca......................................................................  1      1   seq
    ....................augaugucacaauugacugcaU.....................................................................  40     1   seq
    ....................augaugucacaauugacugcag.....................................................................  6      0   seq
```

Mature Star

```
auguccuauguaauaugugaaugaugucacaauugacugcaguugauguugaguccuuuggugaugcagucagugaugacaucauuacaauauugcucucuuacgucaugu
....................augaugucacaauugacugcaUu....................................................................    3    1    seq
....................augaugucacaauugacugcagu....................................................................    3    0    seq
....................augaugucacaauugacugcagA....................................................................    3    1    seq
....................augaugucacaauugacugcaCuu...................................................................    3    1    seq
.........................................guugauguugaguccuuuggugaug.............................................    2    0    seq
..................................................................cagucagugaugacaucauu.........................    4    0    seq
..................................................................cagucagugaugacaucauuaU.......................    1    1    seq
```
