# Supplementary material for: The Small RNA Universe of Capitella teleta
Source: Front Mol Biosci. 2022 Feb 25;9:802814. doi: 10.3389/fmolb.2022.802814 (PMC8915122; doi:10.3389/fmolb.2022.802814)
Supplement: Supplementary file 1 [file DataSheet1.ZIP › Supplement/candidate/CAPTEscaffold_394_20722.pdf]

Provisional ID : CAPTEscaffold_394_20722
Score total : 9508.4
Score for star read(s) : 3.9
Score for read counts : 9505.8
Score for mfe : 1.5
Score for randfold : -2.2
Score for cons. seed : -0.6
Total read count : 18657
Mature read count : 18640
Loop read count : 0
Star read count : 17

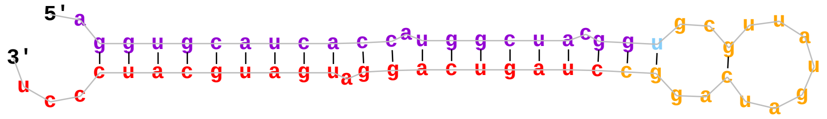

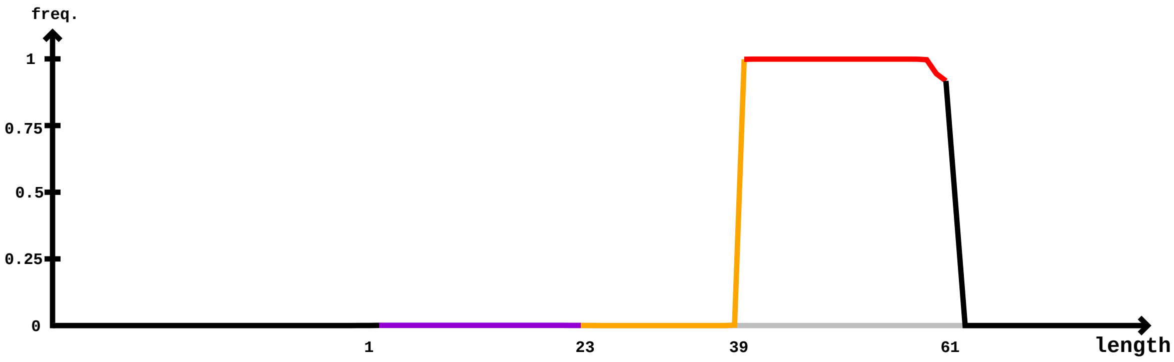


```
                                      Star                                   Mature
5'- aaucgagaacugaccgucagcuucagcgacagaggugcaucaccauggcuacggugcguuaugaucaggccuagucaggaugaugcaucccugcuucugcugcuucuacgcc  -3'  obs
    aaucgagaacugaccgucagcuucagcgacagaggugcaucaccauggcuacggugcguuaugaucaggccuagucaggaugaugcaucccugcuucugcugcuucuacgcc       exp
    .............(((.(((..(((...(((.((((((((((.((((((.(((..(.......)..)))))))))).)))))))))).)))...)))..)))...)))..  reads   mm   sample
    ..............................agaggugcaucaccauggcuac............................................................  5       0    seq
    ................................aggugcaucaccauggcuacg...........................................................  1       0    seq
    ................................aggugcaucaccauggcuacgg..........................................................  7       0    seq
    ................................aggugcaucaccauggcuacggu.........................................................  4       0    seq
    .....................................................................ccuagucaggaugaugcaucc......................  1       0    seq
    .....................................................................ccuagucaggaugaugcauccc.....................  7       0    seq
    .....................................................................ccuagucaggaugaugcaucccu....................  21      0    seq
    ......................................................................cuagucaggaugaugcau........................  2       0    seq
    ......................................................................cuagucaggaugaugcauc.......................  40      0    seq
    ......................................................................cuagucaggaugaGgcauc.......................  1       1    seq
    ......................................................................cuagucaggaugaugUaucc......................  1       1    seq
    ......................................................................cuagucaAgaugaugcaucc......................  2       1    seq
    ......................................................................cAagucaggaugaugcaucc......................  2       1    seq
    ......................................................................Nuagucaggaugaugcaucc......................  1       1    seq
    ......................................................................cuagucaggaugaugcaucU......................  7       1    seq
    ......................................................................cuaguUaggaugaugcaucc......................  1       1    seq
    ......................................................................Guagucaggaugaugcaucc......................  1       1    seq
    ......................................................................cuagucaggaugaugcaucc......................  961     0    seq
    ......................................................................cuagucaggaugaugcauccc.....................  488     0    seq
    ......................................................................cuagucaggauAaugcauccc.....................  1       1    seq
    ......................................................................cuagAcaggaugaugcauccc.....................  1       1    seq
    ......................................................................cAagucaggaugaugcauccc.....................  1       1    seq
    ......................................................................cuagucaggaugaugcaAccc.....................  1       1    seq
    ......................................................................cuagucaggaugaugcauccU.....................  3       1    seq
    ......................................................................cuagucaggaugaugcGuccc.....................  1       1    seq
    ......................................................................cuagucaAgaugaugcauccc.....................  1       1    seq
    ......................................................................cuagucaggNugaugcaucccu....................  1       1    seq
    ......................................................................cuagucaggaugaugAaucccu....................  5       1    seq
    ......................................................................cuagucaUgaugaugcaucccu....................  1       1    seq
    ......................................................................cuagucagUaugaugcaucccu....................  1       1    seq
    ......................................................................cuagucaggaugUugcaucccu....................  4       1    seq
    ......................................................................cuagucaggaugGugcaucccu....................  4       1    seq
    ......................................................................cuagucaggaugaugUaucccu....................  2       1    seq
```

Star Mature

```
aaucgagaacugaccgucagcuucagcgacagaggugcaucaccauggcuacggugcguuaugaucaggccuagucaggaugaugcaucccugcuucugcugcuucuacgcc
......................................................................cuagucaggaugauAcaucccu....................  2      1   seq
......................................................................cuagucagCaugaugcaucccu....................  1      1   seq
......................................................................Uuagucaggaugaugcaucccu....................  6      1   seq
......................................................................cAagucaggaugaugcaucccu....................  15     1   seq
......................................................................cuagucaggaugaugcauAccu....................  1      1   seq
......................................................................cuagucaggauCaugcaucccu....................  2      1   seq
......................................................................cuagAcaggaugaugcaucccu....................  13     1   seq
......................................................................cuaguUaggaugaugcaucccu....................  7      1   seq
......................................................................Nuagucaggaugaugcaucccu....................  4      1   seq
......................................................................cuagucaggaugaugcauccUu....................  5      1   seq
......................................................................cuagucGggaugaugcaucccu....................  10     1   seq
......................................................................cuagucaggaugauCcaucccu....................  1      1   seq
......................................................................cuagucaAgaugaugcaucccu....................  33     1   seq
......................................................................cuagucaggaugaugcaGcccu....................  2      1   seq
......................................................................cuagucaggaugaugcaCcccu....................  3      1   seq
......................................................................cuagucaggaugaugcauccAu....................  3      1   seq
......................................................................cuagucaggaugaugcaucAcu....................  19     1   seq
......................................................................cuagCcaggaugaugcaucccu....................  2      1   seq
......................................................................cuagucaggUugaugcaucccu....................  2      1   seq
......................................................................cuagucaggaugaugcaucUcu....................  16     1   seq
......................................................................cuagucaggauAaugcaucccu....................  14     1   seq
......................................................................cuagucCggaugaugcaucccu....................  1      1   seq
......................................................................cuagucaggaugaugcauUccu....................  1      1   seq
......................................................................cuagucaggauUaugcaucccu....................  2      1   seq
......................................................................cuagucaCgaugaugcaucccu....................  3      1   seq
......................................................................cuaguAaggaugaugcaucccu....................  1      1   seq
......................................................................cuagucaggaugCugcaucccu....................  2      1   seq
......................................................................cuagucaggaugaugcUucccu....................  3      1   seq
......................................................................cuagucaggaugaugcaucccu....................  16614  0   seq
......................................................................cuagucaggaugaCgcaucccu....................  5      1   seq
......................................................................cuagucaggaugaugGaucccu....................  2      1   seq
......................................................................cuagGcaggaugaugcaucccu....................  1      1   seq
......................................................................cuagucaggaugaugcGucccu....................  5      1   seq
......................................................................cuagucaggaugaugcauccGu....................  1      1   seq
......................................................................cuagucaggaugaugcaAcccu....................  6      1   seq
......................................................................cuagucaggaugaugcaucccG....................  116    1   seq
......................................................................cuagucaggaugaugcaucccN....................  1      1   seq
......................................................................cuagucaggaugaugcaucGcu....................  3      1   seq
......................................................................cuGgucaggaugaugcaucccu....................  3      1   seq
......................................................................cuagucaggaAgaugcaucccu....................  5      1   seq
......................................................................cuagucUggaugaugcaucccu....................  1      1   seq
......................................................................cuagucaggaugaugcaucccA....................  68     1   seq
......................................................................cuagucaggaugaAgcaucccu....................  6      1   seq
......................................................................Auagucaggaugaugcaucccu....................  7      1   seq
......................................................................cuagucaggaugauUcaucccu....................  3      1   seq
......................................................................cuagucaggaCgaugcaucccu....................  3      1   seq
......................................................................cuagucaggaugaugcaucccC....................  6      1   seq
......................................................................cuagucaggGugaugcaucccu....................  3      1   seq
......................................................................Guagucaggaugaugcaucccu....................  1      1   seq
......................................................................cuagucaggaugaugcaucccuU...................  15     1   seq
......................................................................cuagucaggaugaugcaucccug...................  2      0   seq
......................................................................cuagucaggaugaugcaucccuA...................  15     1   seq
.......................................................................uagucaggaugaugcaucccu....................  11     0   seq
```
