# Supplementary material for: The Small RNA Universe of Capitella teleta
Source: Front Mol Biosci. 2022 Feb 25;9:802814. doi: 10.3389/fmolb.2022.802814 (PMC8915122; doi:10.3389/fmolb.2022.802814)
Supplement: Supplementary file 1 [file DataSheet1.ZIP › Supplement/candidate/CAPTEscaffold_488_22733.pdf]

Provisional ID	:	CAPTEscaffold_488_22733
Score total	:	8.5
Score for star read(s)	:	3.9
Score for read counts	:	6.8
Score for mfe	:	0.6
Score for randfold	:	-2.2
Score for cons. seed	:	-0.6
Total read count	:	25
Mature read count	:	13
Loop read count	:	0
Star read count	:	12

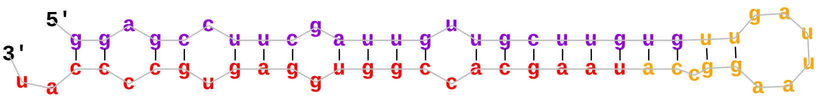

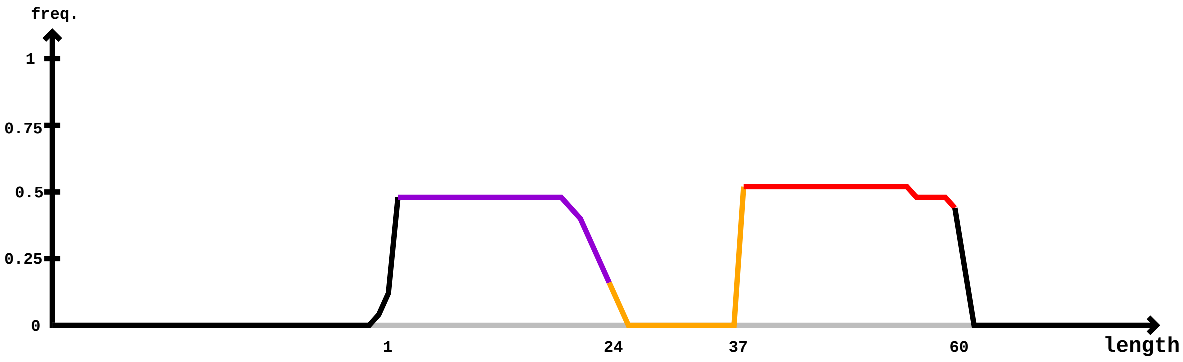

Star Mature

```
5'- acauauagauuauacaaacguucauuucaguuuaggagccuucgauuguugcuuguguugauuaaggccauaagcaccgguggagugccccaugacugaaugguagcccuaca   -3'   obs
    acauauagauuauacaaacguucauuucaguuuaggagccuucgauuguugcuuguguugauuaaggccauaagcaccgguggagugccccaugacugaaugguagcccuaca         exp
    ...................((((..(((((((..((.((.(((.((((.((((((((((......)).)))))))).)))).))).)).))..)))))))..).)))......   reads   mm   sample
    ................................uaggagccuucgauuguugcuu...........................................................   1       0    seq
    .................................aggagccuucgauuguugc.............................................................   1       0    seq
    .................................aggagccuucgauuguugcuug..........................................................   1       0    seq
    ..................................ggagccuucgauuguugcu............................................................   1       0    seq
    ..................................ggagccuucgauuguugcuu...........................................................   1       0    seq
    ..................................ggagccuucgauuguugcuug..........................................................   1       0    seq
    ..................................ggagccuucgauuguugcuugu.........................................................   2       0    seq
    ..................................ggagccuucgauuguugcuugug........................................................   4       0    seq
    ......................................................................uaagcaccgguggagugc.........................   1       0    seq
    ......................................................................uaagcaccgguggagugcccca.....................   1       0    seq
    ......................................................................uaagcaccgguggagugccccau....................   9       0    seq
    ......................................................................uaagcaccgguggagugccccaA....................   1       1    seq
    ......................................................................uaagcaccgguggagugccccauU...................   1       1    seq
```
