# Supplementary material for: The Small RNA Universe of Capitella teleta
Source: Front Mol Biosci. 2022 Feb 25;9:802814. doi: 10.3389/fmolb.2022.802814 (PMC8915122; doi:10.3389/fmolb.2022.802814)
Supplement: Supplementary file 1 [file DataSheet1.ZIP › Supplement/candidate/CAPTEscaffold_168_12407.pdf]

Provisional ID : CAPTEscaffold_168_12407
Score total : 432.2
Score for star read(s) : 3.9
Score for read counts : 425.3
Score for mfe : 2
Score for randfold : 1.6
Score for cons. seed : -0.6
Total read count : 846
Mature read count : 805
Loop read count : 0
Star read count : 41

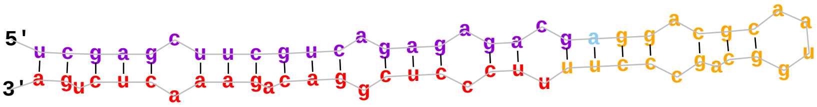

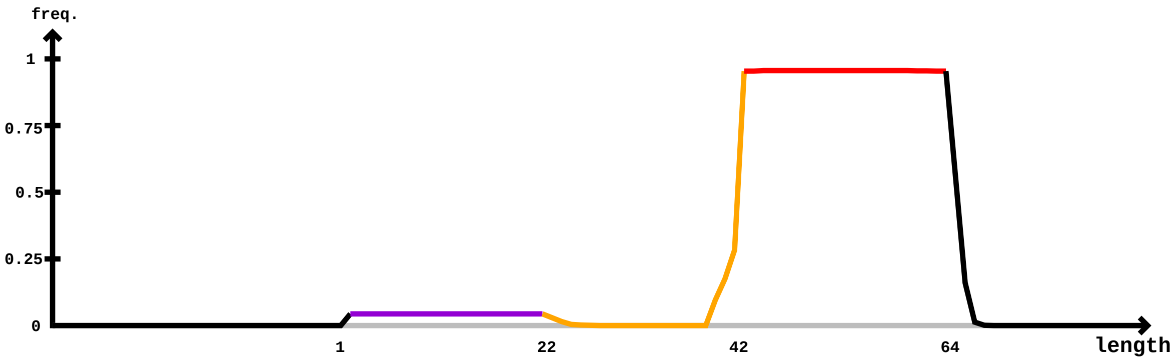


```
                                    Star                                        Mature
5'- ucagcgucggguaaucgaagucaucgacgucgagcuucgucagagagacgaggacgcaauggcagcccuuuucccucggacagaaacucugacgaagaugauuucguuuacg  -3'   obs
    ucagcgucggguaaucgaagucaucgacgucgagcuucgucagagagacgaggacgcaauggcagcccuuuucccucggacagaaacucugacgaagaugauuucguuuacg         exp
    ..........((((.((((((((((..(((((((.((((((.(((.((.((((.(((....)).).)))).)).))).))).))).))).)))).)))..))))))))))).)))).   reads   mm   sample
    .............................ucgagcuucgucagagagacg..............................................................   18      0    seq
    .............................ucgagcuucgucagagagacga.............................................................   9       0    seq
    .............................ucgagcuucgucagagagacgag............................................................   10      0    seq
    .............................ucgagcuucgucagagagacgagg...........................................................   1       0    seq
    .............................ucgagcuucgucagagagacgagU...........................................................   1       1    seq
    .............................ucgagcuucgucagagagacgagga..........................................................   1       0    seq
    .............................ucgagcuucgucagagagacgaggac.........................................................   1       0    seq
    ...................................................................cuuuucccucggacagaaacucuga....................   90      0    seq
    ....................................................................uuuucccucggacagaaacu........................   1       0    seq
    ....................................................................uuuucccucggacagaaacucu......................   1       0    seq
    ....................................................................uuuuccAucggacagaaacucuga....................   1       1    seq
    ....................................................................uuuucccucggacagaaGcucuga....................   4       1    seq
    ....................................................................uuuucccucggacagaaacucuga....................   67      0    seq
    .....................................................................uuucccucggacagaaGcucuga....................   2       1    seq
    .....................................................................uuGcccucggacagaaacucuga....................   1       1    seq
    .....................................................................uuucccucggacagaaacucuga....................   95      0    seq
    .....................................................................uuucccucggacagaaacucugacU..................   1       1    seq
    .....................................................................uuucccucggacagaaacucugacg..................   2       0    seq
    ......................................................................uucccucggacagaaacuAuga....................   1       1    seq
    ......................................................................uucccucAgacagaaacucuga....................   2       1    seq
    ......................................................................uucccuUggacagaaacucuga....................   1       1    seq
    ......................................................................uucccAcggacagaaacucuga....................   1       1    seq
    ......................................................................uucccucggacagaaGcucuga....................   4       1    seq
    ......................................................................uucccucggacagaaacucuga....................   437     0    seq
    ......................................................................uucccucggacagaaaUucuga....................   1       1    seq
    ......................................................................uucccuAggacagaaacucuga....................   1       1    seq
    ......................................................................Aucccucggacagaaacucuga....................   4       1    seq
    ......................................................................uucccucggUcagaaacucuga....................   1       1    seq
    ......................................................................uucccucggacagaaacucAga....................   1       1    seq
    ......................................................................Cucccucggacagaaacucuga....................   1       1    seq
    ......................................................................uucccucggacagaaacucugaA...................   1       1    seq
    ......................................................................uucccucggacagaaGcucugac...................   1       1    seq
    ......................................................................uucccucggacagaaacucugac...................   25      0    seq
```

Star Mature

```
ucagcgucggguaaucgaagucaucgacgucgagcuucgucagagagacgaggacgcaauggcagcccuuuucccucggacagaaacucugacgaagaugauuucguuuacg
......................................................................uucccucggacagaaacucugaU...................   1     1   seq
......................................................................uucccucggUcagaaacucugacg..................   1     1   seq
......................................................................uucccucggacagaaGcucugacg..................   3     1   seq
......................................................................uucccucggacagaaacucugacg..................   119   0   seq
......................................................................uucccucggacagaaacAcugacg..................   1     1   seq
......................................................................uucccucggacagaaacucugacU..................   10    1   seq
......................................................................uucccucggacagaaacucugacA..................   1     1   seq
......................................................................uucccucggacagaaacucugacga.................   7     0   seq
......................................................................uucccucggacagaaacucugacgU.................   2     1   seq
......................................................................uucccucggacagaaacucugacAaa................   1     1   seq
........................................................................cccucggacagaaacucugacga.................   2     0   seq
```
