# Supplementary material for: The Small RNA Universe of Capitella teleta
Source: Front Mol Biosci. 2022 Feb 25;9:802814. doi: 10.3389/fmolb.2022.802814 (PMC8915122; doi:10.3389/fmolb.2022.802814)
Supplement: Supplementary file 1 [file DataSheet1.ZIP › Supplement/candidate/CAPTEscaffold_335_18093.pdf]

Provisional ID	:	CAPTEscaffold_335_18093
Score total	:	1
Score for star read(s)	:	-1.3
Score for read counts	:	0
Score for mfe	:	1.3
Score for randfold	:	1.6
Score for cons. seed	:	-0.6
Total read count	:	961
Mature read count	:	562
Loop read count	:	1
Star read count	:	398

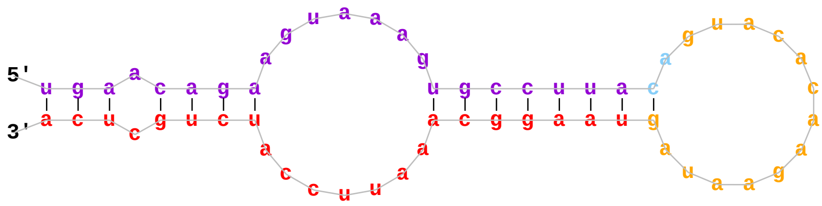

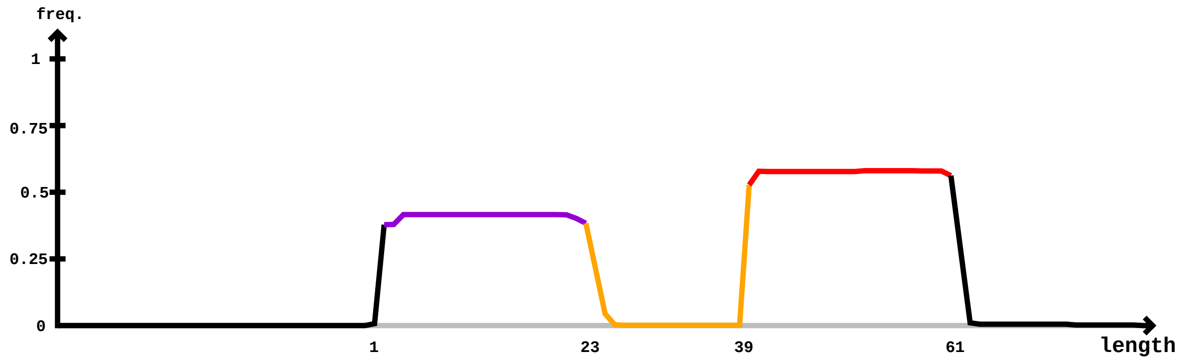


```
                                    Star                                 Mature
5'- cuacuuccuugugucuaugcaguccgaggggaugaacagaaguaaagugccuuacaguacacaagaauaguaaggcaaauuccaucugcucaucgccucugagcuugccagc   -3'   obs
    cuacuuccuugugucuaugcaguccgaggggaugaacagaaguaaagugccuuacaguacacaagaauaguaaggcaaauuccaucugcucaucgccucugagcuugccagc         exp
    .(((......))).....((((((.((((.(((((.((((.......((((((((..............)))))))).......)))).))))).)))).))..))))....    reads     mm     sample
    ...............................augaacagaaguaaagugccu............................................................    4         0      seq
    ...............................augaacagaaguaaagugccuu...........................................................    3         0      seq
    ................................ugaacagaaguaaagugcc.............................................................    1         0      seq
    ................................ugaacagaaguaaagugccu............................................................    8         0      seq
    ................................ugaacagaaguaGagugccu............................................................    1         1      seq
    ................................ugaacagaaguaaagugccuu...........................................................    15        0      seq
    ................................ugaacagaaguaaagugccuua..........................................................    316       0      seq
    ................................ugaacagaaguaaagugccuCa..........................................................    1         1      seq
    ................................Agaacagaaguaaagugccuua..........................................................    3         1      seq
    ................................ugaacagaaguaaagugccuuac.........................................................    3         0      seq
    ................................ugaacagaaguaaagugccuuaA.........................................................    7         1      seq
    ................................ugaacagaaguaaagugccuuaAa........................................................    6         1      seq
    .................................gaacagaaguaaagugccuuaca........................................................    1         0      seq
    ..................................aacagaaguaaagugccuuac.........................................................    1         0      seq
    ..................................aacagaaguaaagugccuuaca........................................................    33        0      seq
    ..................................aacagaaguaaagugccuuacag.......................................................    1         0      seq
    ..................................aacagaaguaaagugccuuacaA.......................................................    1         1      seq
    ......................................................caguacacaagaauagua........................................    1         0      seq
    ......................................................................uaaggcaaauuccaucug........................    1         0      seq
    ......................................................................uaaggcaaauuccaucugcuc.....................    17        0      seq
    ......................................................................uaaggcaaauuUcaucugcuca....................    1         1      seq
    ......................................................................uaaggcaaauuccaucugcuca....................    322       0      seq
    ......................................................................Aaaggcaaauuccaucugcuca....................    2         1      seq
    ......................................................................uaaggcGaauuccaucugcuca....................    1         1      seq
    ......................................................................uaaggcaGauuccaucugcucau...................    1         1      seq
    ......................................................................uaaggcaaauuccaucugcucau...................    57        0      seq
    ......................................................................uaaggcaaauuccaucGgcucau...................    1         1      seq
    ......................................................................uaaggcaaauuccaucugcucaA...................    104       1      seq
    ......................................................................uaaggcaaauuccaucugcucaG...................    2         1      seq
    ......................................................................uaaggcaaauuccaucugcucaC...................    2         1      seq
    ......................................................................uaaggcaaauuccaucugcucauA..................    1         1      seq
    .......................................................................aaggcGaauuccaucugcucau...................    1         1      seq
    .......................................................................aaggcaaauuccaucugcucau...................    45        0      seq
```

Star **Mature**

cuacuuccuugugucuaugcaguccgaggggaugaacagaaguaaagugccuuacaguacacaagaauaguaaggcaaauuccaucugcucaucgccucugagcuugccagc			
....aaggcaaauuccaucugcucauc..................	1	0	seq
....aaggcaaauuccaucugcucauA..................	3	1	seq
....caucugcucaucgccucugagc........	3	0	seq
....ucgccucugagcuugccag.	2	0	seq
