# Supplementary material for: The Small RNA Universe of Capitella teleta
Source: Front Mol Biosci. 2022 Feb 25;9:802814. doi: 10.3389/fmolb.2022.802814 (PMC8915122; doi:10.3389/fmolb.2022.802814)
Supplement: Supplementary file 1 [file DataSheet1.ZIP › Supplement/candidate/CAPTEscaffold_6_914.pdf]

Provisional ID	:	CAPTEscaffold_6_914
Score total	:	227.6
Score for star read(s)	:	3.9
Score for read counts	:	220.4
Score for mfe	:	2.3
Score for randfold	:	1.6
Score for cons. seed	:	-0.6
Total read count	:	444
Mature read count	:	440
Loop read count	:	0
Star read count	:	4

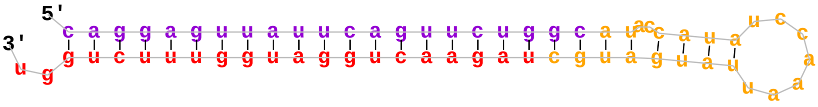

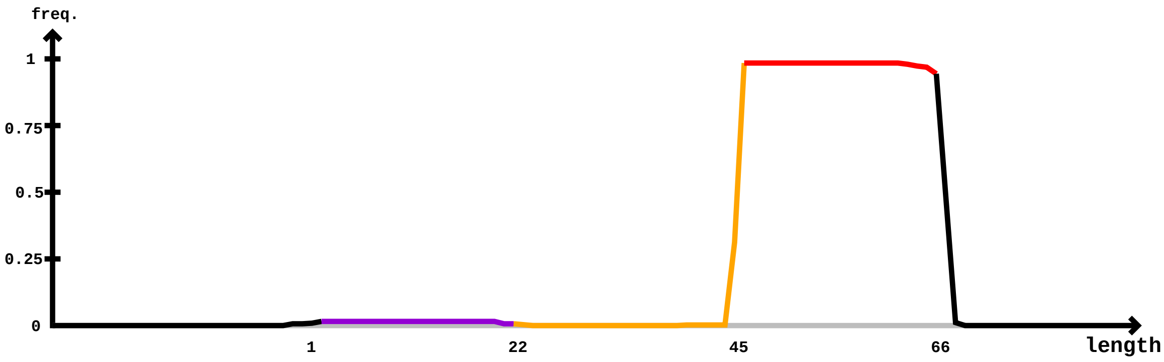

Star **Mature**

```
5'- ggagcauauaugcuggacgagcauaccaggaguuauucaguucuggcauaccauauccaaauuaugaugcuagaacuggaugguuucuggugugcccuccaagcaguacaa  -3'  obs
    ggagcauauaugcuggacgagcauaccaggaguuauucaguucuggcauaccauauccaaauuaugaugcuagaacuggaugguuucuggugugcccuccaagcaguacaa       exp
    ..........(((((((.(.(((((((((((((((((((((((((((((..((((.......))))))))))))))))))))))))))))))))))).))).))))......    reads     mm     sample
    .......................uaccaggaguuauucaguucug..................................................................    1         0      seq
    .......................uaccaggaguGauucaguucug..................................................................    2         1      seq
    .........................ccaggaguGauucaguucug..................................................................    1         1      seq
    ..........................caggaguGauucaguucuggc................................................................    2         1      seq
    ..........................caggaguGauucaguucuggca...............................................................    1         1      seq
    ................................................................ugaugcuagaacuggaugguuuc........................    1         0      seq
    .....................................................................cuagUacuggaugguuuc........................    1         1      seq
    .....................................................................cuagaacuggaugguuucu.......................    1         0      seq
    .....................................................................cuagUacuggaugguuucu.......................    2         1      seq
    .....................................................................cuagUacuggaugguuucug......................    2         1      seq
    .....................................................................cuagaacuggaugguuucugg.....................    3         0      seq
    .....................................................................cuagUacuggaugguuucugg.....................    2         1      seq
    .....................................................................cuagUacuggaugguuucuggu....................    63        1      seq
    .....................................................................cuagaacuggaugguuucuggu....................    18        0      seq
    .....................................................................cuagaacuggaugguuucuAgu....................    1         1      seq
    .....................................................................cuagaacuggaugguuucugguC...................    1         1      seq
    .....................................................................cuagUacuggaugguuucuggug...................    36        1      seq
    .....................................................................cuagaacuggaugguuucuggug...................    5         0      seq
    .....................................................................cuagUacuggaugguuucuggugu..................    4         1      seq
    ......................................................................uagUacuggaugguuucugg.....................    6         1      seq
    ......................................................................uagaacuggaugguuucuggu....................    37        0      seq
    ......................................................................uagUacuggaugguuucuggu....................    176       1      seq
    ......................................................................Aagaacuggaugguuucuggug...................    1         1      seq
    ......................................................................uagaacuggaugguuucuggug...................    30        0      seq
    ......................................................................uagUacuggaugguuucuggug...................    50        1      seq
    ......................................................................uagaacuggaugguuucuggugu..................    1         0      seq
```
