# Supplementary material for: The Small RNA Universe of Capitella teleta
Source: Front Mol Biosci. 2022 Feb 25;9:802814. doi: 10.3389/fmolb.2022.802814 (PMC8915122; doi:10.3389/fmolb.2022.802814)
Supplement: Supplementary file 1 [file DataSheet1.ZIP › Supplement/candidate/CAPTEscaffold_297_17284.pdf]

Provisional ID	:	CAPTEscaffold_297_17284
Score total	:	1.9
Score for star read(s)	:	-1.3
Score for read counts	:	0
Score for mfe	:	2.2
Score for randfold	:	1.6
Score for cons. seed	:	-0.6
Total read count	:	272
Mature read count	:	271
Loop read count	:	0
Star read count	:	1

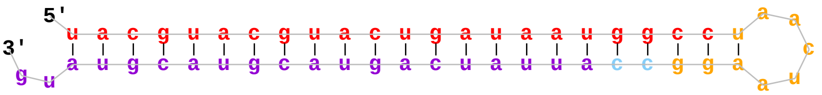

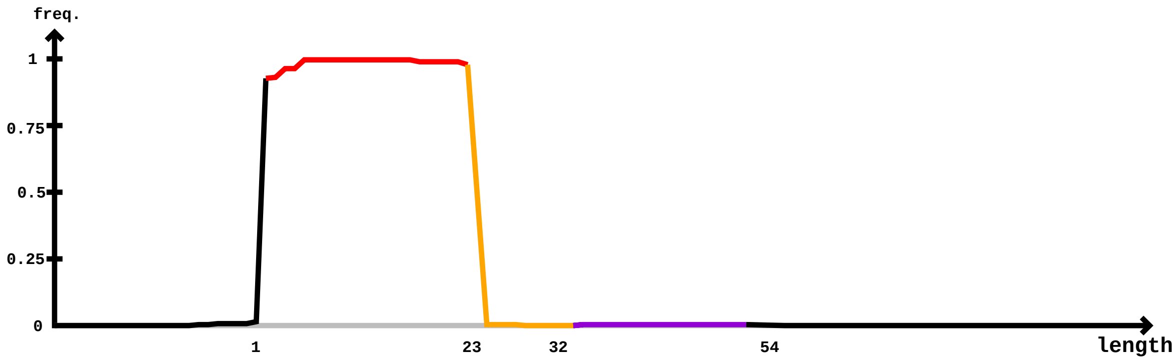

Mature **Star**

```
5'- cuucucgcugcgugcuugcauacguacguacugauaaauggccuaacuaaggccauuaucaguacguacguaugcaauacuuauuacuuacucaacuuuacuuauccggucuu  -3'  obs
    cuucucgcugcgugcuugcauacguacguacugauaaauggccuaacuaaggccauuaucaguacguacguaugcaauacuuauuacuuacucaacuuuacuuauccggucuu       exp
    ..........(((.((((((((((((((((((((((((((((((.....))))))))))))))))))))))))))))))))).............................. reads   mm   sample
    .............gcuugcauacguacguacugaua.............................................................................  1       0    seq
    ...............uugcauacguacguacugaua.............................................................................  1       0    seq
    ...................auacguacguacugauaaauggc.......................................................................  2       0    seq
    ....................uacguacguacugauaaauggc.......................................................................  1       0    seq
    ....................Nacguacguacugauaaauggcc......................................................................  1       1    seq
    ....................uacguacguacugauaaauggcc......................................................................  235     0    seq
    ....................uacguacguacugauaaauggAc......................................................................  1       1    seq
    ....................uacAuacguacugauaaauggcc......................................................................  1       1    seq
    ....................uacguacguacugauaaauggccu.....................................................................  10      0    seq
    ....................uacguacguacugauaaauggccA.....................................................................  1       1    seq
    .....................acguacguacugauaaauggccu.....................................................................  1       0    seq
    ......................cguacguacugauaaauggccu.....................................................................  8       0    seq
    ......................cguacguGcugauaaauggccu.....................................................................  1       1    seq
    ........................uacguacugauaaauggccA.....................................................................  1       1    seq
    ........................uacguacugauaaauggccu.....................................................................  7       0    seq
    ........................uacguacugauaaauggccuaacu.................................................................  1       0    seq
    ......................................................auuaucaguacguacguaug.......................................  1       0    seq
```
