# Supplementary material for: The Small RNA Universe of Capitella teleta
Source: Front Mol Biosci. 2022 Feb 25;9:802814. doi: 10.3389/fmolb.2022.802814 (PMC8915122; doi:10.3389/fmolb.2022.802814)
Supplement: Supplementary file 1 [file DataSheet1.ZIP › Supplement/candidate/CAPTEscaffold_6_893.pdf]

Provisional ID	:	CAPTEscaffold_6_893
Score total	:	162.1
Score for star read(s)	:	3.9
Score for read counts	:	155.1
Score for mfe	:	2.1
Score for randfold	:	1.6
Score for cons. seed	:	-0.6
Total read count	:	316
Mature read count	:	312
Loop read count	:	0
Star read count	:	4

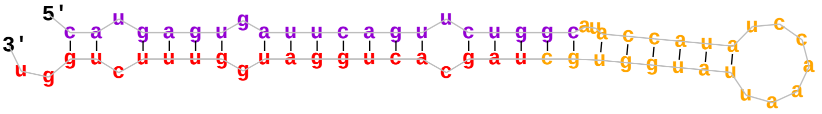

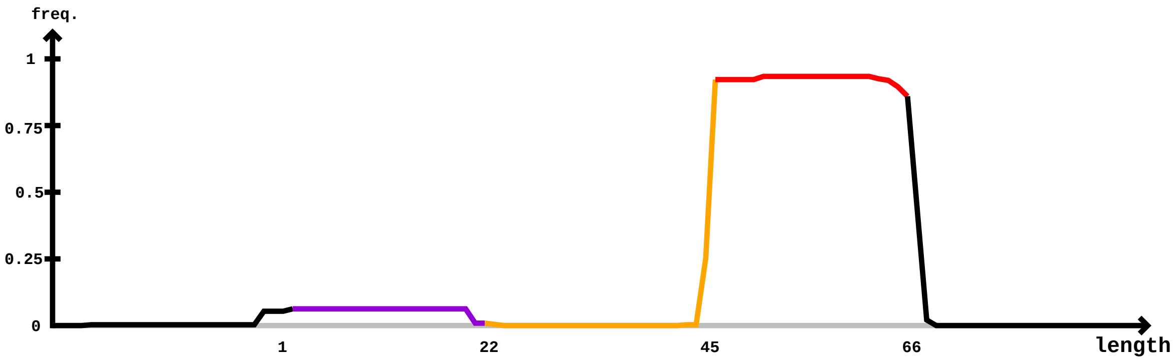

Star Mature

```
5'- gcauauaugcugggugagcauaccaugagugauucaguucuggcauaccauauccaaauuauggugcuagcacuggaugguuucugguaugccccccaugcaguguaccugg -3'    obs
    gcauauaugcugggugagcauaccaugagugauucaguucuggcauaccauauccaaauuauggugcuagcacuggaugguuucugguaugccccccaugcaguguaccugg        exp
    ((((...(((((((.(.((((((((.((((.(((((((.(((((..((((((.......))))))))))).))))))).)))).)))))))).))))).)))))))......    reads   mm   sample
    ..auauaugcugggugagcaua..........................................................................................    1       0    seq
    ....................uaccaGgagugauucaguucug......................................................................    2       1    seq
    ....................uaccaugagugauucaguucug......................................................................    12      0    seq
    ....................uaccaCgagugauucaguucug......................................................................    3       1    seq
    ......................ccaGgagugauucaguucug......................................................................    1       1    seq
    .......................caGgagugauucaguucuggc....................................................................    2       1    seq
    .......................caGgagugauucaguucuggca...................................................................    1       1    seq
    ................................................................ugcuagcacuggaugguuucugU.........................    1       1    seq
    ..................................................................cuagUacuggaugguuuc............................    1       1    seq
    ..................................................................cuagcacuggaugguuuc............................    2       0    seq
    ..................................................................cuagUacuggaugguuucu...........................    2       1    seq
    ..................................................................cuagcacuggaugguuucug..........................    2       0    seq
    ..................................................................cuagUacuggaugguuucug..........................    2       1    seq
    ..................................................................cuagcacuggaugguuucugg.........................    1       0    seq
    ..................................................................cuagUacuggaugguuucugg.........................    2       1    seq
    ..................................................................cuagcacuggaugguuucuggu........................    6       0    seq
    ..................................................................cuagUacuggaugguuucuggu........................    63      1    seq
    ..................................................................cuagcacuggaugguuucugguU.......................    1       1    seq
    ..................................................................cuagUacuggaugguuucuggua.......................    2       1    seq
    ...................................................................uagcacuggaugguuucug..........................    4       0    seq
    ...................................................................uagcacuggaugguuucugA.........................    2       1    seq
    ...................................................................uagUacuggaugguuucugg.........................    6       1    seq
    ...................................................................uagcacuggaugguuucugAu........................    12      1    seq
    ...................................................................uagUacuggaugguuucuggu........................    176     1    seq
    ...................................................................uagcacuggaugguuucuggu........................    5       0    seq
    ...................................................................uagcacuggaugguuucuggua.......................    16      0    seq
    ...................................................................uagcacuggaugguuucugguaA......................    2       1    seq
    ...................................................................uagcacuggaugguuucugguau......................    1       0    seq
    ........................................................................cuggaugguuucugguaA......................    4       1    seq
```
