# Supplementary material for: The Small RNA Universe of Capitella teleta
Source: Front Mol Biosci. 2022 Feb 25;9:802814. doi: 10.3389/fmolb.2022.802814 (PMC8915122; doi:10.3389/fmolb.2022.802814)
Supplement: Supplementary file 1 [file DataSheet1.ZIP › Supplement/candidate/CAPTEscaffold_60_5459.pdf]

Provisional ID	:	CAPTEscaffold_60_5459
Score total	:	1.5
Score for star read(s)	:	-1.3
Score for read counts	:	0
Score for mfe	:	1.8
Score for randfold	:	1.6
Score for cons. seed	:	-0.6
Total read count	:	6560
Mature read count	:	6395
Loop read count	:	0
Star read count	:	165

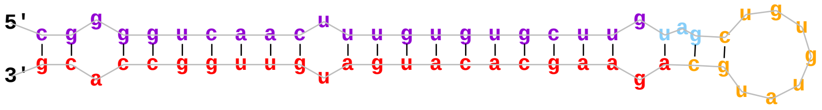

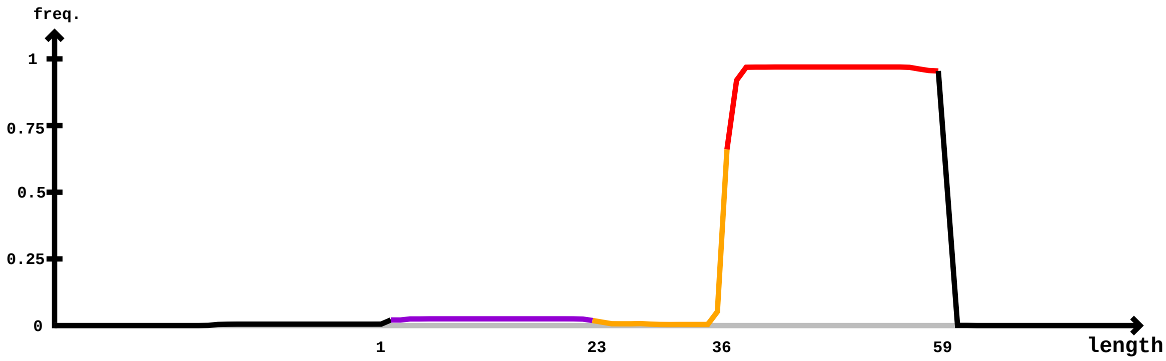

Star   Mature

```
5'- aguuucagcuuugccuauacuugggcgccacugcggggucaacuuugugugcuuguagcuguguaugcagaagcacaugauguuggccacgccgagacgucaucuuagaac  -3'   obs
    aguuucagcuuugccuauacuugggcgccacugcggggucaacuuugugugcuuguagcuguguaugcagaagcacaugauguuggccacgccgagacgucaucuuagaac         exp
    .(((((.....((((((....)))))).....(((.(((((((.((((((((((.(.((.......))).)))))))))).))))))).))).))))).............   reads   mm   sample
    ..............cuauacuugggcgccacug..............................................................................   3       0    seq
    ..............cuauacuugggcgccacugcg............................................................................   1       0    seq
    ...............Aauacuugggcgccacug..............................................................................   1       1    seq
    ...............uauacuugggcgccacug..............................................................................   10      0    seq
    ...............uauacuugggcgccacugc.............................................................................   1       0    seq
    ...............uauacuuggAcgccacugc.............................................................................   1       1    seq
    ...............uauacuugggcgccacugcg............................................................................   9       0    seq
    ...............uauacuuggAcgccacugcg............................................................................   2       1    seq
    ................auacuugggcgccacugc.............................................................................   1       0    seq
    ................auacuugggcgccacugcg............................................................................   4       0    seq
    .................uacuugggcgccacugcg............................................................................   2       0    seq
    ................................gcggggucaacuuugugugcu..........................................................   1       0    seq
    .................................cggggucaacuuugugugc...........................................................   1       0    seq
    .................................cggggucaacuuugugugcu..........................................................   4       0    seq
    .................................cggggucaacuuugugugcuu.........................................................   34      0    seq
    .................................cAgggucaacuuugugugcuu.........................................................   1       1    seq
    .................................cggggucaacuuugugugcuug........................................................   41      0    seq
    .................................cggggucaacuAugugugcuug........................................................   1       1    seq
    .................................cggggucaacuuugugugcuuC........................................................   11      1    seq
    .................................cggggucaacuuugugugcuuU........................................................   5       1    seq
    .................................cggggGcaacuuugugugcuug........................................................   1       1    seq
    .................................Nggggucaacuuugugugcuug........................................................   1       1    seq
    .................................cggggucaacuuugugugcuugA.......................................................   7       1    seq
    .................................cggggucaacuuugugugcuugu.......................................................   10      0    seq
    ..................................ggggucaacuuugugugcuugu.......................................................   2       0    seq
    ...................................gggucaUcuuugugugcuugu.......................................................   1       1    seq
    ...................................gggucaacuuugugugcuugu.......................................................   1       0    seq
    ...................................gggucaUcuuugugugcuugua......................................................   1       1    seq
    ...................................gggucaacuuugugugcuugua......................................................   1       0    seq
    ...................................gggucaUcuuugugugcuuguagc....................................................   10      1    seq
    ...................................gggucaacuuugugAgcuuguagc....................................................   1       1    seq
    ...................................gggucaacuuugugugcuuguagc....................................................   7       0    seq
    ...................................gggucaacuuugugugcuuguagU....................................................   1       1    seq
```

Star Mature

```
aguuucagcuuugccuauacuugggcgccacugcggggucaacuuugugugcuuguagcuguguaugcagaagcacaugauguuggccacgccgagacgucaucuuagaac
...................................gggucaacuuugugugcuuguagcu...................................................	10	0	seq
...................................gggucaacuuugugugcuuguagcug.................................................	7	0	seq
...................................gggucaacuuugugugcuuguagcuU.................................................	1	1	seq
...................................gggucaacuuugugugcuuguagcuCu................................................	1	1	seq
...................................gggucaacuuugugugcuuguagcugu................................................	1	0	seq
...................................gggucaUcuuugugugcuuguagcugugu...............................................	1	1	seq
.....................................gucaacuuugugugcuuguagcu...................................................	3	0	seq
.........................................................gcuguguaugcGgaagcacaugauguuggc........................	1	1	seq
...........................................................uguguaugcagaagcacaugauguuggcc.......................	18	0	seq
...........................................................uguguaugcagaagcacaugauguuggcca......................	1	0	seq
...........................................................uguguaugcagaagcacaugauguuggccU......................	3	1	seq
...........................................................uguguaugcagaagcacaugauguuggccac.....................	2	0	seq
...............................................................uaugcagaagcacaugauguuggcc.......................	1	0	seq
................................................................augcagaagcacaugauguuggcc.......................	1	0	seq
...................................................................cagaagcacaugauguuggcc.......................	16	0	seq
...................................................................cagaagcacauAauguuggcca......................	1	1	seq
...................................................................cagaagcacaugauguuggcca......................	18	0	seq
...................................................................cagaagcacaugauguuUcca......................	1	1	seq
...................................................................cagaagcacaugauguuggccac.....................	4	0	seq
...................................................................cagaagcacaugauguuAgccacg....................	1	1	seq
...................................................................cagaagcacaugauguuggcAacg....................	4	1	seq
...................................................................cagaagcacauAauguuggccacg....................	1	1	seq
...................................................................cagaagcacaugauguuggccacg....................	264	0	seq
...................................................................cagaagUacaugauguuggccacg....................	1	1	seq
...................................................................cagaagcacaugauguuggccacU....................	3	1	seq
...................................................................cagaagAacaugauguuggccacg....................	1	1	seq
...................................................................cagaagcacaugauguuggccacgA...................	3	1	seq
....................................................................agaagcacaugauguuggc........................	5	0	seq
....................................................................agaagcacaugauguuggcc.......................	2	0	seq
....................................................................agaagcacaugauguuggcca......................	10	0	seq
....................................................................agaagcacaugauguuggccac.....................	3	0	seq
....................................................................agaagcGcaugauguuggccacg....................	2	1	seq
....................................................................agaagcacaugauguuggccacg....................	3851	0	seq
....................................................................agaagcacaugaAguuggccacg....................	1	1	seq
....................................................................agaaUcacaugauguuggccacg....................	1	1	seq
....................................................................aUaagcacaugauguuggccacg....................	1	1	seq
....................................................................agaagcacaugaugCuggccacg....................	1	1	seq
....................................................................agaagcacauAauguuggccacg....................	9	1	seq
....................................................................agaagcacaugauguugAccacg....................	1	1	seq
....................................................................agaagcacaugauguuggccacA....................	1	1	seq
....................................................................agaagcacaAgauguuggccacg....................	2	1	seq
....................................................................agaGgcacaugauguuggccacg....................	2	1	seq
....................................................................agaagcacaugauUuuggccacg....................	2	1	seq
....................................................................aNaagcacaugauguuggccacg....................	1	1	seq
....................................................................agaagcacaugauguuggUcacg....................	1	1	seq
....................................................................agaagcacaugauguuggcAacg....................	110	1	seq
....................................................................agaagcacaugauAuuggccacg....................	1	1	seq
....................................................................agaagcacaugauguCggccacg....................	1	1	seq
....................................................................agaagcacaugauguuggGcacg....................	1	1	seq
....................................................................agaagcacauUauguuggccacg....................	1	1	seq
....................................................................agaagcacaugauguuggccacC....................	1	1	seq
....................................................................agaagcacaugauguuUgccacg....................	1	1	seq
....................................................................agaagcacaugauguAggccacg....................	6	1	seq
....................................................................agaagcacaugauguuggccaAg....................	2	1	seq
....................................................................agaagcacaugauguuggccGcg....................	1	1	seq
....................................................................agaagcacaugauguuAgccacg....................	2	1	seq
....................................................................agaagcacaugaugAuggccacg....................	3	1	seq
....................................................................agaagcacaugauguuCgccacg....................	1	1	seq
....................................................................agaagcacaugauguuggccaUg....................	1	1	seq
....................................................................agaagcacaugauguuggccacgA...................	4	1	seq
.....................................................................gaagcacaugauguuggc........................	2	0	seq
.....................................................................gaagcacaugauguuggcc.......................	3	0	seq
.....................................................................gaagcacaugauguuggcca......................	4	0	seq
.....................................................................gaagcacaugauguuggccac.....................	2	0	seq
.....................................................................gaagUacaugauguuggccacg....................	1	1	seq
.....................................................................gaagcacaugauguuggccaGg....................	1	1	seq
.....................................................................gaagcacaugauguAggccacg....................	2	1	seq
.....................................................................Aaagcacaugauguuggccacg....................	22	1	seq
.....................................................................gaagcacaugaugAuggccacg....................	4	1	seq
.....................................................................gaagcacauAauguuggccacg....................	3	1	seq
```

Star Mature

```
aguuucagcuuugccuauacuugggcgccacugcggggucaacuuugugugcuuguagcuguguaugcagaagcacaugauguuggccacgccgagacgucaucuuagaac
.....................................................................gaagcacaugaugCuggccacg....................    1     1    seq
.....................................................................gaagcacGugauguuggccacg....................    1     1    seq
.....................................................................gaagcacauCauguuggccacg....................    1     1    seq
.....................................................................gGagcacaugauguuggccacg....................    1     1    seq
.....................................................................gaagcacaugauguuggGcacg....................    1     1    seq
.....................................................................gaagcacaugauguuggccacA....................    1     1    seq
.....................................................................gaagcacaugauguuAgccacg....................    1     1    seq
.....................................................................gaagcacaugauguuggUcacg....................    1     1    seq
.....................................................................Uaagcacaugauguuggccacg....................    5     1    seq
.....................................................................gaagcacaugaCguuggccacg....................    1     1    seq
.....................................................................gaagcacaugauguCggccacg....................    1     1    seq
.....................................................................gUagcacaugauguuggccacg....................    1     1    seq
.....................................................................gaagcaAaugauguuggccacg....................    1     1    seq
.....................................................................gaagcGcaugauguuggccacg....................    1     1    seq
.....................................................................gaagcacaugauguuggcAacg....................    42    1    seq
.....................................................................Caagcacaugauguuggccacg....................    2     1    seq
.....................................................................gaaUcacaugauguuggccacg....................    1     1    seq
.....................................................................gaagcacaugGuguuggccacg....................    1     1    seq
.....................................................................gaagcacaugauguuCgccacg....................    1     1    seq
.....................................................................gaagcacaugauguuggccacg....................    1604  0    seq
.....................................................................gaagcacaugauguuggccacgc...................    1     0    seq
.....................................................................gaagcacaugauguuggccacUc...................    1     1    seq
......................................................................aagcacaugauguuggccac.....................    1     0    seq
......................................................................aagcacaugauguuggccacg....................    319   0    seq
......................................................................aagcacaugauguAggccacg....................    1     1    seq
......................................................................aagcacaugGuguuggccacg....................    1     1    seq
......................................................................aagcacauAauguuggccacg....................    1     1    seq
......................................................................aagcacaugauguuggccacgc...................    1     0    seq
.......................................................................agcacaugauguuggccacg....................    1     0    seq
.......................................................................agcacaugauguuggccacgc...................    1     0    seq
.......................................................................agcacaugauguuggccacgccU.................    1     1    seq
.........................................................................cacaugauguuggccacg....................    1     0    seq
.........................................................................cacaugauguuggccacgccg.................    2     0    seq
```
