# Supplementary material for: The Small RNA Universe of Capitella teleta
Source: Front Mol Biosci. 2022 Feb 25;9:802814. doi: 10.3389/fmolb.2022.802814 (PMC8915122; doi:10.3389/fmolb.2022.802814)
Supplement: Supplementary file 1 [file DataSheet1.ZIP › Supplement/candidate/CAPTEscaffold_292_14542.pdf]

Provisional ID	:	CAPTEscaffold_292_14542
Score total	:	84.7
Score for star read(s)	:	3.9
Score for read counts	:	78.1
Score for mfe	:	1.7
Score for randfold	:	1.6
Score for cons. seed	:	-0.6
Total read count	:	165
Mature read count	:	128
Loop read count	:	0
Star read count	:	37

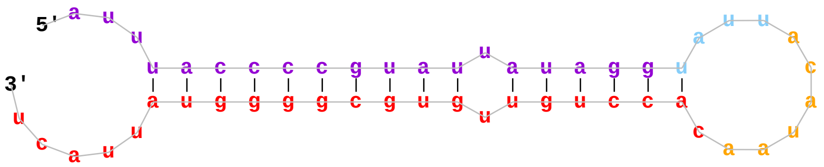

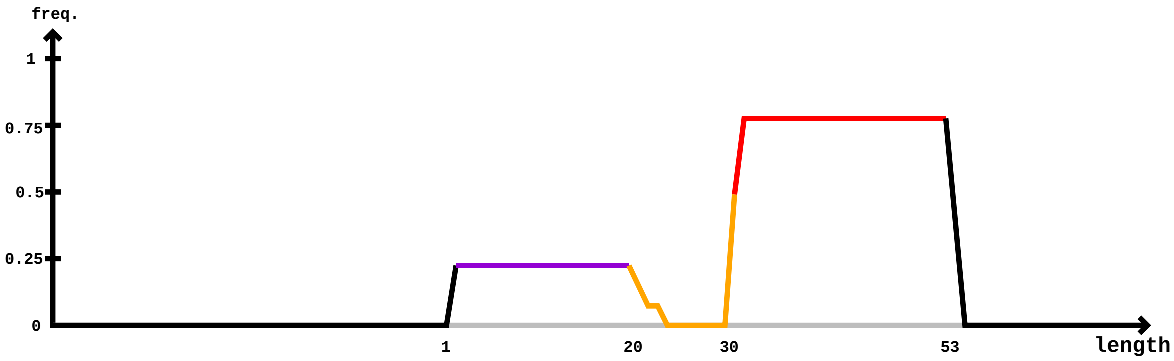

Star Mature

```
5'- uuucagauuugcagcucugcguauguaacagcgaggaauauuuaccccguauuauagguauuacauaacaccuguugugcgggguauuacuuucuugcucguugauaaucg   -3'   obs
    uuucagauuugcagcucugcguauguaacagcgaggaauauuuaccccguauuauagguauuacauaacaccuguugugcgggguauuacuuucuugcucguugauaaucg         exp
    ......((((..((((..(((....)))..(((((((((....((((((((((.((((((.........)))))).))))))))))....))))))))).))))..)))).   reads   mm   sample
    .......................................auuuaccccguauuauagg.....................................................   25      0    seq
    .......................................auuuaccccguauuauagguau..................................................   11      0    seq
    .......................................auuuacGccguauuauagguau..................................................   1       1    seq
    ....................................................................caccugAugugcgggguauuacu....................   1       1    seq
    ....................................................................caccuguugugcgggguauuacu....................   80      0    seq
    .....................................................................accuguugugcgggguauuacu....................   47      0    seq
```
