# Supplementary material for: The Small RNA Universe of Capitella teleta
Source: Front Mol Biosci. 2022 Feb 25;9:802814. doi: 10.3389/fmolb.2022.802814 (PMC8915122; doi:10.3389/fmolb.2022.802814)
Supplement: Supplementary file 1 [file DataSheet1.ZIP › Supplement/candidate/CAPTEscaffold_35_3577.pdf]

Provisional ID	:	CAPTEscaffold_35_3577
Score total	:	1.8
Score for star read(s)	:	-1.3
Score for read counts	:	0
Score for mfe	:	2.1
Score for randfold	:	1.6
Score for cons. seed	:	-0.6
Total read count	:	362
Mature read count	:	303
Loop read count	:	0
Star read count	:	59

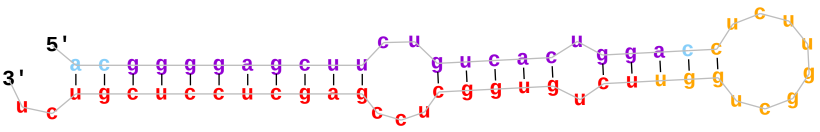

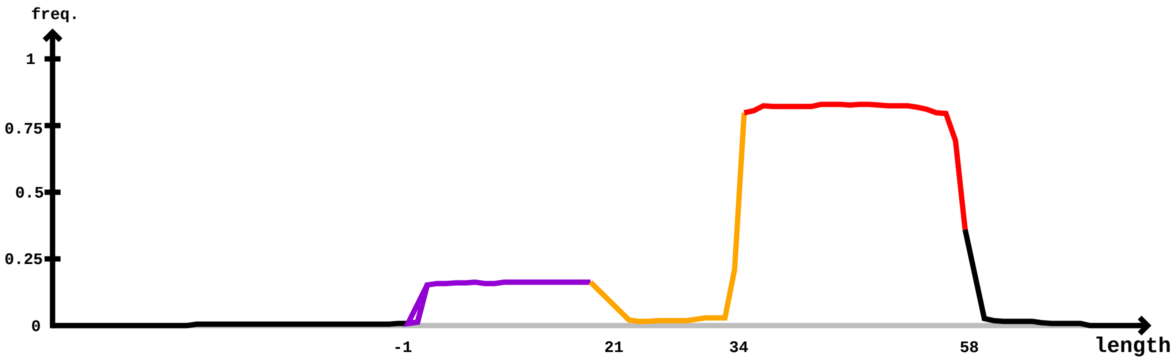

Star Mature

```
5'- acuaagcaugggaauagaagaccuugcccuguucaacggggagcuucugucacuggaccucuuggcugguucuguggcuccgagcuccucgucugaacagcugcuguccccc  -3'  obs
    acuaagcaugggaauagaagaccuugcccuguucaacggggagcuucugucacuggaccucuuggcugguucuguggcuccgagcuccucgucugaacagcugcuguccccc       exp
    .........(((.((((.((........((((((((((((((((((..(((((.(((((........))))).)))))...))))))))))).))))))))).)))).))).   reads   mm   sample
    .............auagaagaccuuAcccuguucaacggggag.....................................................................   1       1    seq
    .............auagaagaccuugcccuguucaacggggag.....................................................................   1       0    seq
    ..................................aacggggagcuucugucacuggac......................................................   1       0    seq
    ....................................cggggagcuucugucacuggac......................................................   1       0    seq
    ....................................cggggagcuucugucacuggacU.....................................................   1       1    seq
    .....................................ggggagcuucugucacugga.......................................................   52      0    seq
    .....................................ggggGgcuucugucacugga.......................................................   1       1    seq
    ......................................gggagcuucugucacuggac......................................................   1       0    seq
    ......................................gggagcuucugucacuggacc.....................................................   1       0    seq
    ........................................gagcuucugucacuggacc.....................................................   1       0    seq
    ..........................................gcuucugucacuggaccucuuggcugguucu.......................................   1       0    seq
    .............................................ucugucacuggaccucuuggcugguuc........................................   2       0    seq
    ........................................................accucuuggcugguucuguggcucc...............................   1       0    seq
    ..........................................................cucuuggcugguucuguggcuccgag............................   1       0    seq
    ...........................................................ucuuggcugguucuguggcuccgagcucc........................   1       0    seq
    .............................................................uuggcugguucuguggcuccgagcucc........................   1       0    seq
    .................................................................cugguucuguggcuccgagc...........................   1       0    seq
    .................................................................cugguucuguggcuccgagcuccucgucuga................   1       0    seq
    ..................................................................ugguucuguggcuccgagcuccucgucuga................   2       0    seq
    .....................................................................uucuguggcuccgagcuccu.......................   1       0    seq
    .....................................................................uucuguggcuccgagcuccuc......................   1       0    seq
    .....................................................................uucuguggcuccgagcuccucgu....................   9       0    seq
    .....................................................................uucuguggcuccgagcuccucgC....................   1       1    seq
    .....................................................................uucuguggcuccgagcuccucguc...................   31      0    seq
    .....................................................................uucuguggcuccgagcuccucguU...................   2       1    seq
    .....................................................................uucuguggcuccgagcuccucgucu..................   22      0    seq
    .....................................................................uucuguggcuccgagcuccucgucA..................   1       1    seq
    .....................................................................uucuguggcuccgagcuccucgucugaa...............   1       0    seq
    ......................................................................ucuguggcuccgagcuccu.......................   1       0    seq
    ......................................................................Acuguggcuccgagcuccu.......................   1       1    seq
    ......................................................................ucuguggcuccgagcuccuc......................   4       0    seq
    ......................................................................ucuguggcuccgagcuccucg.....................   1       0    seq
    ......................................................................ucuguggcuccgagcuccucgu....................   27      0    seq
```

Star Mature

```
acuaagcaugggaauagaagaccuugcccuguucaacggggagcuucugucacuggaccucuuggcugguucuguggcuccgagcuccucgucugaacagcugcuguccccc
......................................................................ucuguggcuccgagcucUucgu....................   1    1    seq
......................................................................ucuguggcuccgagcuccucgC....................   1    1    seq
......................................................................ucuguggcuccgagAuccucguc...................   1    1    seq
......................................................................ucuguggcuccgagcuccucguA...................   2    1    seq
......................................................................ucuguggcuccgagcuccucguc...................   89   0    seq
......................................................................ucuguggcuccgagcuccucguU...................   2    1    seq
......................................................................ucuguggcuccgagcuccuUgucu..................   1    1    seq
......................................................................ucuguggcuccAagcuccucgucu..................   1    1    seq
......................................................................ucuguggcuccgagcuccucgucu..................   91   0    seq
......................................................................ucuguggcuccgagcuccucgucA..................   1    1    seq
.......................................................................cuguggcuccAagcuccucgucu..................   1    1    seq
.......................................................................cuguggcuccgagcuccucgucu..................   2    0    seq
........................................................................uguggcuccgagcuccucgucu..................   6    0    seq
........................................................................uguggcuccgagcuccucgucug.................   1    0    seq
........................................................................uguggcuccgagcuccucgucugaacagc...........   2    0    seq
..............................................................................uccgagcuccucgucugaacagcugcug......   3    0    seq
..................................................................................agcuccucgucugaacagcu..........   1    0    seq
```
