# Supplementary material for: The Small RNA Universe of Capitella teleta
Source: Front Mol Biosci. 2022 Feb 25;9:802814. doi: 10.3389/fmolb.2022.802814 (PMC8915122; doi:10.3389/fmolb.2022.802814)
Supplement: Supplementary file 1 [file DataSheet1.ZIP › Supplement/candidate/CAPTEscaffold_394_20707.pdf]

Provisional ID	:	CAPTEscaffold_394_20707
Score total	:	14
Score for star read(s)	:	3.9
Score for read counts	:	7.8
Score for mfe	:	1.3
Score for randfold	:	1.6
Score for cons. seed	:	-0.6
Total read count	:	27
Mature read count	:	19
Loop read count	:	0
Star read count	:	8

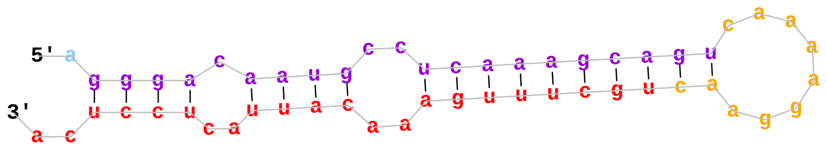

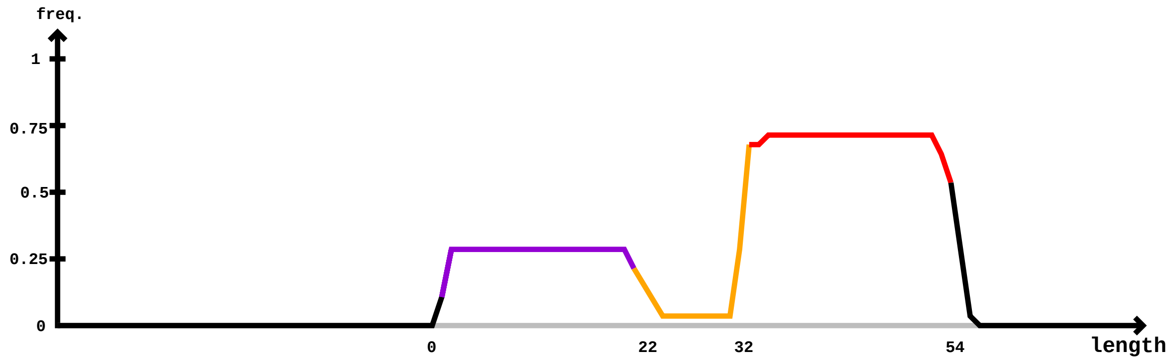


```
                                                      Star                             Mature
5'- guuccgucuucucugagcugaauugcagcccccaagugagggacaaugccucaaagcagucaaaaggaacugcuuugaaacauuacuccucacucgagugcgccuaucgcg   -3'    obs
    guuccgucuucucugagcugaauugcagcccccaaguguagggacaaugccucaaagcagucaaaaggaacugcuuugaaacauuacuccucacucgagugcgccuaucgcg          exp
    ((((.(......).))))(((...((.((.(.(.((((((((..((((..((((((((((........))))))))))..))))..)))))))).).).))))...)))..     reads        mm         sample
    ......................................agggacaaugccucaaagca.....................................................     2            0          seq
    ......................................agggacaaugccucaaagcagu...................................................     1            0          seq
    .......................................gggacaaugccucaaagcagu...................................................     3            0          seq
    .......................................gggacaaugccucaaagcaguc..................................................     2            0          seq
    ...........................................................ucaaaaggaacugcuuugaaacauuacuccu.....................     1            0          seq
    .....................................................................cugcuuugaaacauuacuccu.....................     1            0          seq
    .....................................................................cugcuuugaaacauuacuccucU...................     6            1          seq
    ......................................................................ugcuuugaaacauuacuccuc....................     3            0          seq
    ......................................................................ugcuuugaaacauuacuccucU...................     8            1          seq
    ........................................................................cuuugaGacauuacuccucacu.................     1            1          seq
```
