# Supplementary material for: The Small RNA Universe of Capitella teleta
Source: Front Mol Biosci. 2022 Feb 25;9:802814. doi: 10.3389/fmolb.2022.802814 (PMC8915122; doi:10.3389/fmolb.2022.802814)
Supplement: Supplementary file 1 [file DataSheet1.ZIP › Supplement/candidate/CAPTEscaffold_14983_45140.pdf]

Provisional ID	:	CAPTEscaffold_14983_45140
Score total	:	0.7
Score for star read(s)	:	-1.3
Score for read counts	:	0
Score for mfe	:	1
Score for randfold	:	1.6
Score for cons. seed	:	-0.6
Total read count	:	81
Mature read count	:	62
Loop read count	:	0
Star read count	:	19

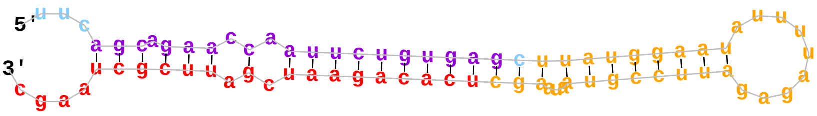

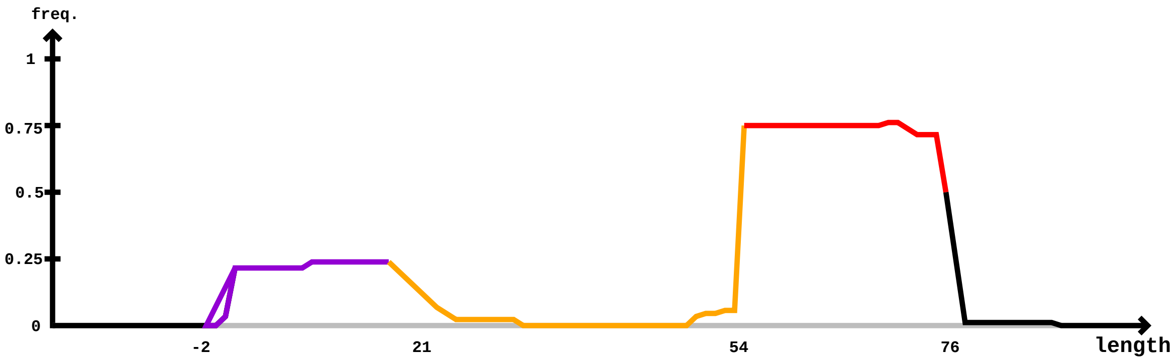

Star **Mature**

```
5'- ugaauuauaagaucuucagcagaaccaauucugugagcuuauggaauauuuuagagauuccguaauaagcucacagaaucgauucgcuaagcuaugccauucaucaacagaa   -3'    obs
    ugaauuauaagaucuucagcagaaccaauucugugagcuuauggaauauuuuagagauuccguaauaagcucacagaaucgauucgcuaagcuaugccauucaucaacagaa          exp
    (((((........(((.(((.(((.(.((((((((((((((((((((.........))))))))...)))))))))))).).))))))))).......))))).........    reads     mm     sample
    ...............cagcagaaccaauucAgugagc...........................................................................    1         1      seq
    ...............cagcagaaccaauucAgugagcu..........................................................................    2         1      seq
    ................agcagaaccaauucAgugag............................................................................    12        1      seq
    ................agcagaaccaauucAgugagc...........................................................................    2         1      seq
    ................agcagaaccaauucugugagcuu.........................................................................    1         0      seq
    ................agcagaaccaauucAgugagcuu.........................................................................    1         1      seq
    .........................caauucugugagcuuauggaau.................................................................    2         0      seq
    .................................................................uaagcucacagaaucgauucUc.........................    2         1      seq
    .................................................................uaagcucacagaaucgauucUcu........................    1         1      seq
    ..................................................................aagcucacagaaucgauucUcu........................    1         1      seq
    ....................................................................gcucacagaaucgauucgcuaag.....................    1         0      seq
    ......................................................................ucacagaaucgauucgcuaag.....................    18        0      seq
    ......................................................................ucacagaaucgauucgcuaagU....................    6         1      seq
    ......................................................................ucacagaaucgauucUcuaagc....................    1         1      seq
    ......................................................................ucacagaaucgauucgcuaagc....................    36        0      seq
    .....................................................................................Ucuaagcuaugccauuca.........    1         1      seq
```
