# Supplementary material for: The Small RNA Universe of Capitella teleta
Source: Front Mol Biosci. 2022 Feb 25;9:802814. doi: 10.3389/fmolb.2022.802814 (PMC8915122; doi:10.3389/fmolb.2022.802814)
Supplement: Supplementary file 1 [file DataSheet1.ZIP › Supplement/candidate/CAPTEscaffold_234_15402.pdf]

Provisional ID	:	CAPTEscaffold_234_15402
Score total	:	1119.8
Score for star read(s)	:	3.9
Score for read counts	:	1114.1
Score for mfe	:	0.8
Score for randfold	:	1.6
Score for cons. seed	:	-0.6
Total read count	:	2197
Mature read count	:	1904
Loop read count	:	0
Star read count	:	293

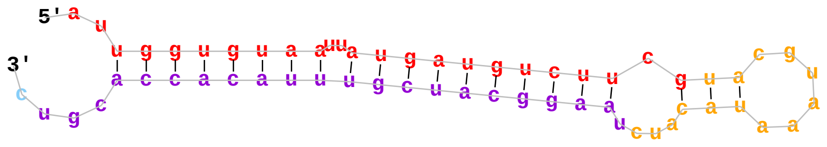

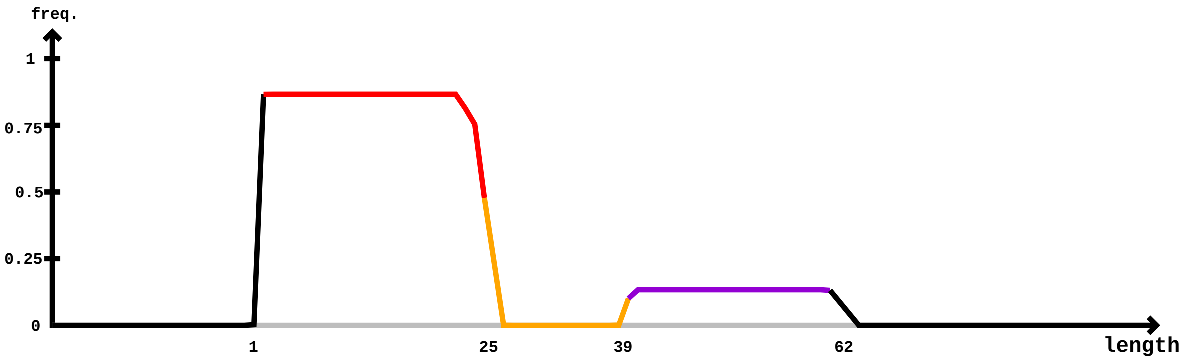

Mature   Star

```
5'- ugaagccaagcagacaguguauugguguaauuaugaugucuucguacguaaauacaucuaaggcaucguuuacaccacgucauuucugucggaauuacgucauagagaugccg   -3'    obs
    ugaagccaagcagacaguguauugguguaauuaugaugucuucguacguaaauacaucuaaggcaucguuuacaccacgucauuucugucggaauuacgucauagagaugccg          exp
    (((..((..(((((.((((...((((((((..(((((((((.(((......)))....)))))))))))))))))...))))))))).))..)))((((.....))))...   reads   mm   sample
    ...................uauugguguaauuaugaugucu........................................................................   2       0    seq
    ...................uauugguguaauuaugaugucuuc......................................................................   1       0    seq
    ...................uauugguguaauuaugaugucuucg.....................................................................   1       0    seq
    ...................Cauugguguaauuaugaugucuucgu....................................................................   1       1    seq
    ....................auugguguaauuaugaugucu........................................................................   112     0    seq
    ....................auugguguaauuaugaugAcu........................................................................   1       1    seq
    ....................auugguguaauuaugGugucuu.......................................................................   1       1    seq
    ....................auugguguaauuaugaugucuC.......................................................................   1       1    seq
    ....................auugguguaauuaugaugucuu.......................................................................   131     0    seq
    ....................auugguAuaauuaugaugucuuc......................................................................   1       1    seq
    ....................auugguguaauuaugaugCcuuc......................................................................   1       1    seq
    ....................Guugguguaauuaugaugucuuc......................................................................   1       1    seq
    ....................auugguguaauuaugaugucuuA......................................................................   1       1    seq
    ....................aAugguguaauuaugaugucuuc......................................................................   1       1    seq
    ....................Nuugguguaauuaugaugucuuc......................................................................   1       1    seq
    ....................auugguguaauuaugaugucuuc......................................................................   598     0    seq
    ....................aCugguguaauuaugaugucuuc......................................................................   1       1    seq
    ....................auuggGguaauuaugaugucuucg.....................................................................   1       1    seq
    ....................auuAguguaauuaugaugucuucg.....................................................................   1       1    seq
    ....................auugguguaauuaugaugucuucg.....................................................................   992     0    seq
    ....................auugguguaauuaugaugucuucU.....................................................................   4       1    seq
    ....................auugguguaauuaugaugucuuUg.....................................................................   6       1    seq
    ....................auugguguaauAaugaugucuucg.....................................................................   1       1    seq
    ....................auAgguguaauuaugaugucuucg.....................................................................   1       1    seq
    ....................auugguguaauuaugaugAcuucg.....................................................................   1       1    seq
    ....................auugguguaauuaugaugucuAcg.....................................................................   1       1    seq
    ....................Guugguguaauuaugaugucuucg.....................................................................   6       1    seq
    ....................auugAuguaauuaugaugucuucg.....................................................................   1       1    seq
    ....................auugguguaaGuaugaugucuucg.....................................................................   1       1    seq
    ....................auugguCuaauuaugaugucuucg.....................................................................   1       1    seq
    ....................aGugguguaauuaugaugucuucg.....................................................................   1       1    seq
    ....................auugguguaauuaugaugucuucgA....................................................................   1       1    seq
    ....................auugguguaauuaugaugucuucgu....................................................................   26      0    seq
```

Mature **Star**

```
ugaagccaagcagacaguguauugguguaauuaugaugucuucguacguaaauacaucuaaggcaucguuuacaccacgucauuucugucggaauuacgucauagagaugccg
....................auCgguguaauuaugaugucuucgu....................................................................	1	1	seq
....................auugguguaauuaugaugucuucguU...................................................................	1	1	seq
.....................uugguguaauuaugaugucuucg.....................................................................	1	0	seq
.........................................................cuaaggcaucguuuacaccacgu.................................	2	0	seq
..........................................................uaaggcaucguuuacaccacg..................................	5	0	seq
..........................................................uaaggcaucCuuuacaccacgu.................................	1	1	seq
..........................................................uaaggcaucguuuacaccacgu.................................	205	0	seq
..........................................................Aaaggcaucguuuacaccacgu.................................	4	1	seq
..........................................................uaaggcaucguuuacacUacgu.................................	1	1	seq
..........................................................uaaggcaucguuuacaccacguc................................	3	0	seq
...........................................................aaggcaucguuuacaccacgu.................................	11	0	seq
...........................................................aaggcaucguuuacacAacgu.................................	2	1	seq
...........................................................aaggcaucguuuacaccacguc................................	56	0	seq
...........................................................aaggcauAguuuacaccacguc................................	1	1	seq
...........................................................aaggcaucguuuacaccacgucU...............................	2	1	seq
```
