# Supplementary material for: The Small RNA Universe of Capitella teleta
Source: Front Mol Biosci. 2022 Feb 25;9:802814. doi: 10.3389/fmolb.2022.802814 (PMC8915122; doi:10.3389/fmolb.2022.802814)
Supplement: Supplementary file 1 [file DataSheet1.ZIP › Supplement/candidate/CAPTEscaffold_488_22707.pdf]

Provisional ID	:	CAPTEscaffold_488_22707
Score total	:	20685.6
Score for star read(s)	:	3.9
Score for read counts	:	20678.6
Score for mfe	:	2.1
Score for randfold	:	1.6
Score for cons. seed	:	-0.6
Total read count	:	40572
Mature read count	:	40568
Loop read count	:	0
Star read count	:	4

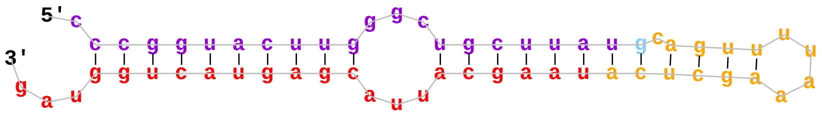

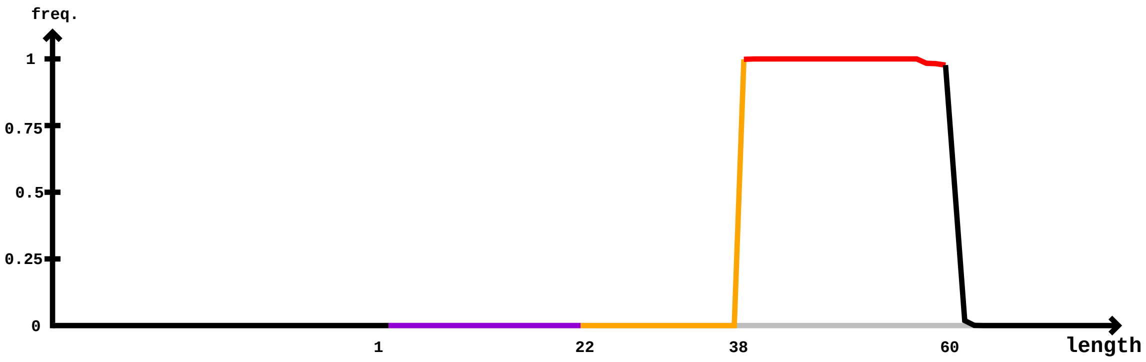

Star Mature

```
5'- gacuuagucagacacugaucgggucuugcuuuucccgguacuugggcugcuuaugcaguuuuaaagcucauaagcauuacgaguacugguagaggcaaggucuaaaacu  -3'   obs
    gacuuagucagacacugaucgggucuugcuuuucccgguacuugggcugcuuaugcaguuuuaaagcucauaagcauuacgaguacugguagaggcaaggucuaaaacu         exp
    (((((.(((((...))))).)))))((((((((.((((((((((...((((((((.((((....))))))))))))...)))))))))).))))))))...........   reads   mm   sample
    ............cacugaucgggCcuugcuuuu............................................................................   1       1    seq
    .................aucgggucuugcuuuucc..........................................................................   1       0    seq
    .................................cccgguacuugggcugcuuau.......................................................   3       0    seq
    .................................cccgguacuugggcugcuuaugA.....................................................   1       1    seq
    ......................................................................uaagcauuacgaguacug.....................   2       0    seq
    ......................................................................uaagcauuacgGguacugg....................   1       1    seq
    ......................................................................uaagcauuacgaguacugU....................   1       1    seq
    ......................................................................uaagcauuacgaguacuAg....................   3       1    seq
    ......................................................................Caagcauuacgaguacugg....................   1       1    seq
    ......................................................................uaagcauuacgaUuacugg....................   1       1    seq
    ......................................................................uaagcauuacgaguacugg....................   624     0    seq
    ......................................................................uaagcauCacgaguacugg....................   2       1    seq
    ......................................................................uaagcauuaAgaguacugg....................   17      1    seq
    ......................................................................Aaagcauuacgaguacugg....................   1       1    seq
    ......................................................................uaagcaAuacgaguacugg....................   1       1    seq
    ......................................................................uaagcauuacAaguacugg....................   5       1    seq
    ......................................................................uaagcauuacgaguacuggA...................   1       1    seq
    ......................................................................uaagcauuacgaguacuggG...................   2       1    seq
    ......................................................................uaagcauuaAgaguacuggu...................   4       1    seq
    ......................................................................uaagcauuacgaguacuggu...................   57      0    seq
    ......................................................................uaagcauuacgaguacuggua..................   145     0    seq
    ......................................................................uaagcauuGcgaguacuggua..................   1       1    seq
    ......................................................................uaagcauuacgaCuacuggua..................   1       1    seq
    ......................................................................uaagcauuacgaguacuAgua..................   1       1    seq
    ......................................................................uaagcauuacgaguacugguG..................   1       1    seq
    ......................................................................Gaagcauuacgaguacuggua..................   1       1    seq
    ......................................................................uaagcauuacAaguacuggua..................   3       1    seq
    ......................................................................uaagcauuaAgaguacuggua..................   63      1    seq
    ......................................................................uaagcauuacUaguacuggua..................   1       1    seq
    ......................................................................Naagcauuacgaguacugguag.................   12      1    seq
    ......................................................................uaagcauAacgaguacugguag.................   18      1    seq
    ......................................................................uaagcauuacgaUuacugguag.................   1       1    seq
    ......................................................................uaagcauuacgaguacugguGg.................   41      1    seq
```

Star Mature

gacuuagucagacacugaucgggucuugcuuuucccgguacuugggcugcuuaugcaguuuuaaagcucauaagcauuacgaguacugguagaggcaaggucuaaaacu

....uaagcauuaGgaguacugguag.................	4	1	seq
....uaagcauuacgGguacugguag.................	2	1	seq
....uaagcauuacgagCacugguag.................	4	1	seq
....uaagcUuuacgaguacugguag.................	2	1	seq
....uaagcauuacgaguacugguaU.................	3	1	seq
....uaagcauuacgaguUcugguag.................	3	1	seq
....uaagcauuacgaguacugAuag.................	4	1	seq
....Aaagcauuacgaguacugguag.................	126	1	seq
....uaagUauuacgaguacugguag.................	2	1	seq
....uaagGauuacgaguacugguag.................	3	1	seq
....uaagcauuacgaguacugguaA.................	42	1	seq
....uaagcauuaAgaguacugguag.................	3027	1	seq
....Caagcauuacgaguacugguag.................	6	1	seq
....uaagcaGuacgaguacugguag.................	3	1	seq
....uGagcauuacgaguacugguag.................	16	1	seq
....uaagcauuacCaguacugguag.................	1	1	seq
....uaagcauuacgaAuacugguag.................	2	1	seq
....uaagcauuacgaguGcugguag.................	6	1	seq
....uNagcauuacgaguacugguag.................	4	1	seq
....Gaagcauuacgaguacugguag.................	13	1	seq
....uaagcauuacgaguacuCguag.................	3	1	seq
....uaagcauuacgaguacugguaC.................	37	1	seq
....uaagcaAuacgaguacugguag.................	30	1	seq
....uaagcauuacgaguacugguUg.................	4	1	seq
....uaagcauuaUgaguacugguag.................	3	1	seq
....uaagcauuacgagAacugguag.................	11	1	seq
....uaagcauuacgaguacCgguag.................	4	1	seq
....uaagcauuacgUguacugguag.................	2	1	seq
....uaagcauuacUaguacugguag.................	1	1	seq
....uaUgcauuacgaguacugguag.................	1	1	seq
....uCagcauuacgaguacugguag.................	1	1	seq
....uaagcGuuacgaguacugguag.................	6	1	seq
....uaagcauuGcgaguacugguag.................	7	1	seq
....uaagcauuacgaguacugguag.................	30599	0	seq
....uaaAcauuacgaguacugguag.................	6	1	seq
....uaagcauuacgaguaGugguag.................	5	1	seq
....uaagcauuacgaguacuggGag.................	7	1	seq
....uaagcauuacgagGacugguag.................	1	1	seq
....uaagcauuacgaguacugCuag.................	1	1	seq
....uaagcauuacgaguaUugguag.................	2	1	seq
....uaagcauGacgaguacugguag.................	1	1	seq
....uaagcauuacAaguacugguag.................	166	1	seq
....uaaUcauuacgaguacugguag.................	1	1	seq
....uaagcauuacgaguacuggCag.................	2	1	seq
....uaagcauuacgaguaAugguag.................	3	1	seq
....uaaCcauuacgaguacugguag.................	1	1	seq
....uaagcauuacgaguacuUguag.................	2	1	seq
....uaagcauuacgaguacuggAag.................	7	1	seq
....uaagcauuacgaguacugUuag.................	1	1	seq
....uaGgcauuacgaguacugguag.................	1	1	seq
....uaagcauuacgaguacuAguag.................	39	1	seq
....uaagcauCacgaguacugguag.................	3	1	seq
....uaagcauuacgaguacAgguag.................	11	1	seq
....uaagcauAacgaguacugguaga................	1	1	seq
....uaagcauuacgaguacuAguaga................	1	1	seq
....uaagcauuacgaguacAgguaga................	2	1	seq
....uaagcauuacgaguacugguagC................	3	1	seq
....uaagcauuacgaguacugguGga................	1	1	seq
....uaagcauuacAaguacugguaga................	9	1	seq
....uaagcaGuacgaguacugguaga................	1	1	seq
....uaagcauuacgaguacugguaAa................	7	1	seq
....uaagcauuaGgaguacugguaga................	1	1	seq
....uaagcauuacgaguacuggAaga................	1	1	seq
....uaagcauuaAgaguacugguaga................	1046	1	seq
....uaagcaAuacgaguacugguaga................	2	1	seq
....uaagcauuacgaguacugguaga................	3277	0	seq
....uaagcauuacgaguaUugguaga................	2	1	seq
....uaagcauuacgaguacugguagU................	128	1	seq
....uaagcauuacgaUuacugguaga................	1	1	seq
....uaagcauuacgaguGcugguaga................	1	1	seq

```
                                 Star                                Mature
gacuuagucagacacugaucgggucuugcuuuucccgguacuugggcugcuuaugcaguuuuaaagcucauaagcauuacgaguacugguagaggcaaggucuaaaacu
......................................................................Aaagcauuacgaguacugguaga................    17    1    seq
......................................................................uaaAcauuacgaguacugguaga................    1     1    seq
......................................................................uaagGauuacgaguacugguaga................    1     1    seq
......................................................................uaagcauuacgaguaAugguaga................    1     1    seq
......................................................................uaagcauuaAgaguacugguagag...............    3     1    seq
......................................................................uaagcauuacgaguacugguagaU...............    2     1    seq
......................................................................uaagcauuacgaguacugguagag...............    2     0    seq
......................................................................uaagcauuacgaguacugguagaA...............    717   1    seq
......................................................................uaagcauuacgaguacugguagaC...............    4     1    seq
......................................................................uaagcauuacgaguacugguagagA..............    8     1    seq
......................................................................uaagcauuacgaguacugguagaAg..............    17    1    seq
.......................................................................aagcauuacAaguacugguag.................    2     1    seq
.......................................................................Uagcauuacgaguacugguag.................    2     1    seq
.......................................................................aagcauuacgaguacugguag.................    35    0    seq
.......................................................................aagcauuacgaguacugguaga................    11    0    seq
.......................................................................aagcauuacgaguacugguagaU...............    1     1    seq
........................................................................agcauuacgaguacugguaga................    5     0    seq
..........................................................................cauuaAgaguacugguaga................    1     1    seq
```
