# Supplementary material for: The Small RNA Universe of Capitella teleta
Source: Front Mol Biosci. 2022 Feb 25;9:802814. doi: 10.3389/fmolb.2022.802814 (PMC8915122; doi:10.3389/fmolb.2022.802814)
Supplement: Supplementary file 1 [file DataSheet1.ZIP › Supplement/candidate/CAPTEscaffold_95_7968.pdf]

Provisional ID	:	CAPTEscaffold_95_7968
Score total	:	1.9
Score for star read(s)	:	-1.3
Score for read counts	:	0
Score for mfe	:	2.2
Score for randfold	:	1.6
Score for cons. seed	:	-0.6
Total read count	:	19
Mature read count	:	17
Loop read count	:	0
Star read count	:	2

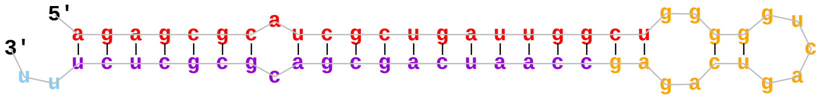

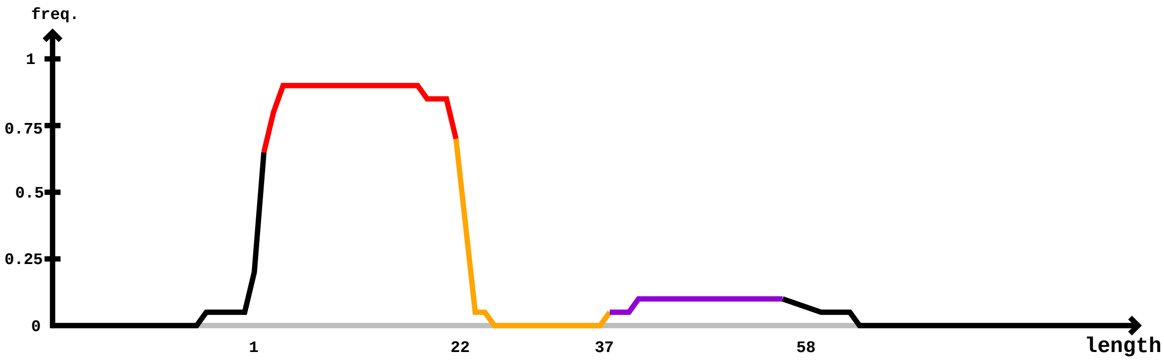

Mature **Star**

```
5'- gccacagacgucaaacaugaagagcgcaucgcugauuggcugggggucagucagagccaaucagcgacgcgcucuuuauggcgcaucugauguauuccaugacauuuaacg   -3'    obs
    gccacagacgucaaacaugaagagcgcaucgcugauuggcugggggucagucagagccaaucagcgacgcgcucuuuauggcgcaucugauguauuccaugacauuuaacg          exp
    ....((((.((....((((((((((((.(((((((((((((..((.....))..))))))))))))).))))))))))))..)).))))((((........))))......    reads      mm       sample
    ..............acaugaagagcgcaucgcugauA..........................................................................    1          1        seq
    ...................aagagcgcaucgcugauuggcu......................................................................    1          0        seq
    ...................aagagcgcGucgcugauuggcu......................................................................    2          1        seq
    ....................agagcgcGucgcugauuggc.......................................................................    2          1        seq
    ....................agagcgcaucgcugauuggc.......................................................................    1          0        seq
    ....................agagcgcaucgcugauuggcu......................................................................    6          0        seq
    .....................gagcgcaucgcugauuggcu......................................................................    1          0        seq
    .....................gagcgcGucgcugauuggcu......................................................................    2          1        seq
    ......................agcgcGucgcugauuggcu......................................................................    1          1        seq
    ......................agcgcaucgcugauuggcuggg...................................................................    1          0        seq
    ........................................................ccaaucagcgacgcgcucu....................................    1          0        seq
    ...........................................................aucagcgacgcgcucuuuauggc.............................    1          0        seq
```
