# Supplementary material for: The Small RNA Universe of Capitella teleta
Source: Front Mol Biosci. 2022 Feb 25;9:802814. doi: 10.3389/fmolb.2022.802814 (PMC8915122; doi:10.3389/fmolb.2022.802814)
Supplement: Supplementary file 1 [file DataSheet1.ZIP › Supplement/candidate/CAPTEscaffold_7256_41364.pdf]

Provisional ID	:	CAPTEscaffold_7256_41364
Score total	:	5595.6
Score for star read(s)	:	3.9
Score for read counts	:	5590.4
Score for mfe	:	0.4
Score for randfold	:	1.6
Score for cons. seed	:	-0.6
Total read count	:	10977
Mature read count	:	10960
Loop read count	:	0
Star read count	:	17

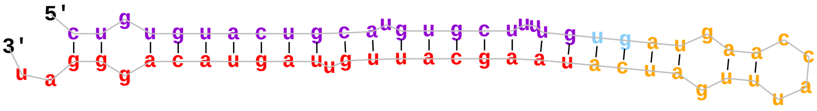

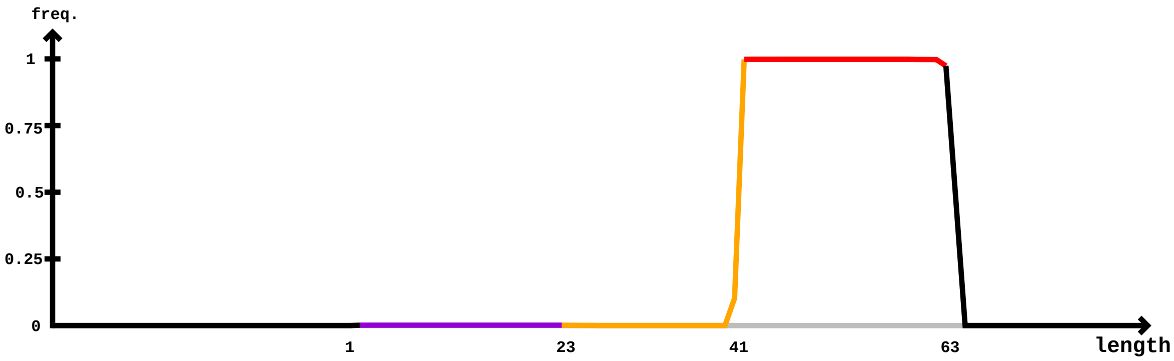

Star Mature

```
5'- ugaguuuccuauucuuuggcccucacucuucuguguacugcaugugcuuuugugaugaaccauuugaucauaagcauuguuaguacagggauggaugauguugauacagcua  -3'   obs
    ugaguuuccuauucuuuggcccucacucuucuguguacugcaugugcuuuugugaugaaccauuugaucauaagcauuguuaguacagggauggaugauguugauacagcua         exp
    ((((...((........))..)))).((((((.(((((((((.(((((..((((((.((....)).))))))))))))).))))))).))).)))....((((...))))..   reads   mm   sample
    ..............................cuguguacugcaugugcuuuu.............................................................   1       0    seq
    ..............................cuguguacugcaugugcuuuug............................................................   5       0    seq
    ..............................cuguguacugcaugugcuuuuA............................................................   1       1    seq
    ..............................cuguguacugcaugugcuuuugu...........................................................   4       0    seq
    ..............................cuguguacugcaugugcuuuuguga.........................................................   4       0    seq
    ...............................uguguacugcaugugcuuuuguga.........................................................   1       0    seq
    ...............................uguguacugcaugugcuuuugugG.........................................................   1       1    seq
    .....................................................................auaagcauugCuaguacag........................   4       1    seq
    .....................................................................auaagcauugCuaguacagg.......................   1       1    seq
    .....................................................................auaagcauugCuaguacaggg......................   1       1    seq
    .....................................................................auaagcauuguuaguacaggU......................   1       1    seq
    .....................................................................auaagcauuguuaguacaggga.....................   1       0    seq
    .....................................................................auaagcauugCuaguacaggga.....................   30      1    seq
    .....................................................................auaagcauugCuaguacagggau....................   1062    1    seq
    .....................................................................auaagcauuguuaguacagggau....................   11      0    seq
    .....................................................................auaagcauuguuaguacagggauU...................   2       1    seq
    .....................................................................auaagcauugCuaguacagggaug...................   12      1    seq
    ......................................................................uaagcauugCuaguacag........................   3       1    seq
    ......................................................................uaagcauuguuaguacaggga.....................   2       0    seq
    ......................................................................uaagcauugCuaguacaggga.....................   226     1    seq
    ......................................................................uaagcauuguuaguacagggau....................   116     0    seq
    ......................................................................uaagcauugGuaguacagggau....................   2       1    seq
    ......................................................................uaagcauugCuaguacagggau....................   8824    1    seq
    ......................................................................uaagcauugGuaguacagggaug...................   1       1    seq
    ......................................................................uaagcauuguuaguacagggauA...................   1       1    seq
    ......................................................................uaagcauuguuaguacagggaug...................   4       0    seq
    ......................................................................uaagcauugCuaguacagggaug...................   646     1    seq
    ......................................................................uaagcauuguuaguacagggauU...................   7       1    seq
    .......................................................................aagcauugCuaguacagggau....................   2       1    seq
    ..........................................................................cauugCuaguacagggau....................   1       1    seq
```
