# Supplementary material for: The Small RNA Universe of Capitella teleta
Source: Front Mol Biosci. 2022 Feb 25;9:802814. doi: 10.3389/fmolb.2022.802814 (PMC8915122; doi:10.3389/fmolb.2022.802814)
Supplement: Supplementary file 1 [file DataSheet1.ZIP › Supplement/candidate/CAPTEscaffold_35_3565.pdf]

Provisional ID	: CAPTEscaffold_35_3565
Score total	: 22.3
Score for star read(s)	: 3.9
Score for read counts	: 15.4
Score for mfe	: 2
Score for randfold	: 1.6
Score for cons. seed	: -0.6
Total read count	: 42
Mature read count	: 29
Loop read count	: 0
Star read count	: 13

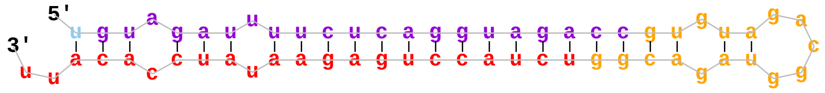

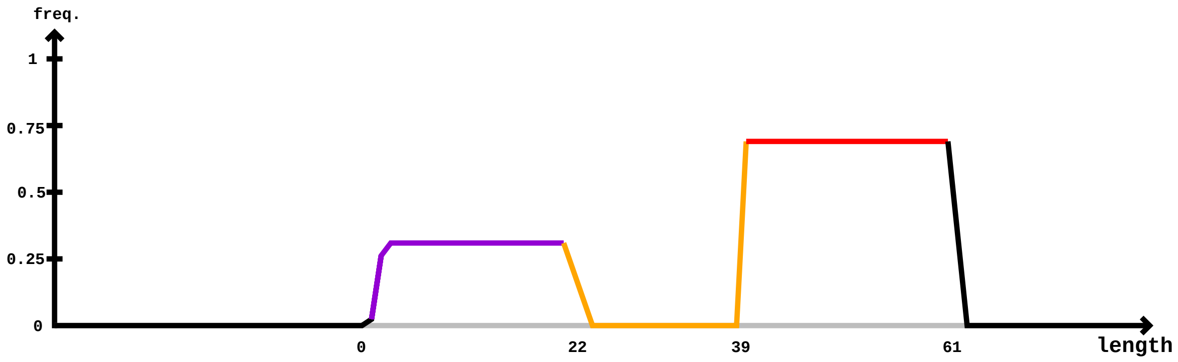

Star **Mature**

```
5'- aaguagaugaggcagggauaugauuuaaggauguagauuuucucagguagaccguguagacgguagacggucuaccugagaauauccacauuauaaucauuauggagcguau   -3'    obs
    aaguagaugaggcagggauaugauuuaaggauguagauuuucucagguagaccguguagacgguagacggucuaccugagaauauccacauuauaaucauuauggagcguau          exp
    .........(.((......((((((....(((((.(((.((((((((((((((((.((.....)).))))))))))))))))).))).)))))..))))))......)).)..  reads   mm    sample
    ...............................uguagauuuucucagguagaccg..........................................................  1       0     seq
    ................................guagauuuucucagguagacc...........................................................  10      0     seq
    .................................uagauuuucucagguagacc...........................................................  2       0     seq
    ......................................................................ucuaccugagaauauccacauu....................  29      0     seq
```
