# Supplementary material for: The Small RNA Universe of Capitella teleta
Source: Front Mol Biosci. 2022 Feb 25;9:802814. doi: 10.3389/fmolb.2022.802814 (PMC8915122; doi:10.3389/fmolb.2022.802814)
Supplement: Supplementary file 1 [file DataSheet1.ZIP › Supplement/candidate/CAPTEscaffold_2743_37448.pdf]

Provisional ID	: CAPTEscaffold_2743_37448
Score total	: 11.8
Score for star read(s)	: 3.9
Score for read counts	: 5.2
Score for mfe	: 1.7
Score for randfold	: 1.6
Score for cons. seed	: -0.6
Total read count	: 22
Mature read count	: 15
Loop read count	: 0
Star read count	: 7

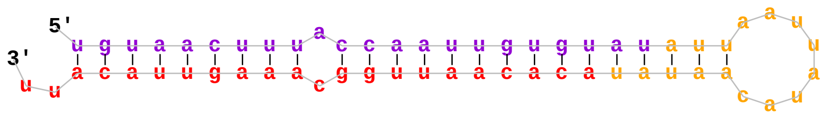

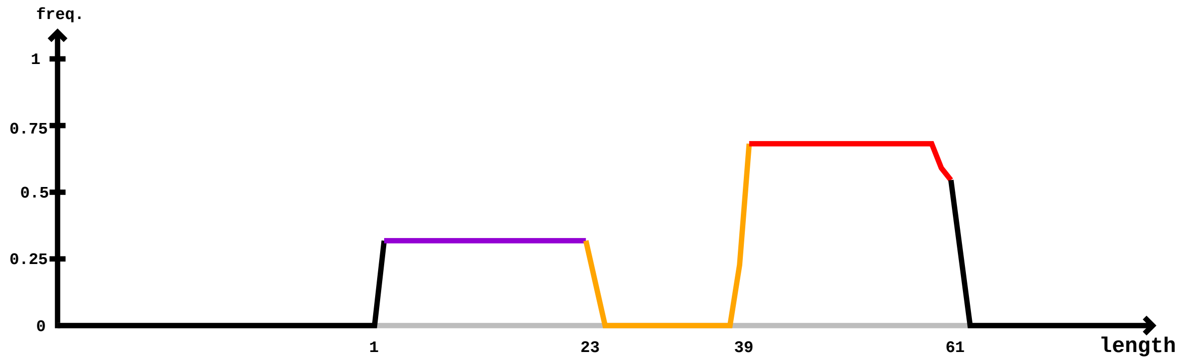

Star **Mature**

```
5'- uacacagaaaucaguaaacagcuguaucauuauguaacuuuaccaauuguguauauuaauuauacaauauacacaauuggcaaaguuacauuauuccugcuguaauuacuuc   -3'    obs
    uacacagaaaucaguaaacagcuguaucauuauguaacuuuaccaauuguguauauuaauuauacaauauacacaauuggcaaaguuacauuauuccugcuguaauuacuuc          exp
    ............((((((((((.........((((((((((.(((((((((((((((........))))))))))))))).)))))))))).......)))))..)))))..   reads      mm         sample
    ................................uguaacuuuaccaauuguguau..........................................................   7          0          seq
    .....................................................................uacacaauuggcaaaguuaca......................   2          0          seq
    .....................................................................uacacaauuAgcaaaguuacau.....................   1          1          seq
    .....................................................................uacacaauuggcaaaguuacauu....................   2          0          seq
    ......................................................................acacaauuggcaaaguuacauu....................   10         0          seq
```
