# Supplementary material for: The Small RNA Universe of Capitella teleta
Source: Front Mol Biosci. 2022 Feb 25;9:802814. doi: 10.3389/fmolb.2022.802814 (PMC8915122; doi:10.3389/fmolb.2022.802814)
Supplement: Supplementary file 1 [file DataSheet1.ZIP › Supplement/candidate/CAPTEscaffold_264_14680.pdf]

Provisional ID	:	CAPTEscaffold_264_14680
Score total	:	88.8
Score for star read(s)	:	3.9
Score for read counts	:	82.2
Score for mfe	:	1.7
Score for randfold	:	1.6
Score for cons. seed	:	-0.6
Total read count	:	173
Mature read count	:	171
Loop read count	:	0
Star read count	:	2

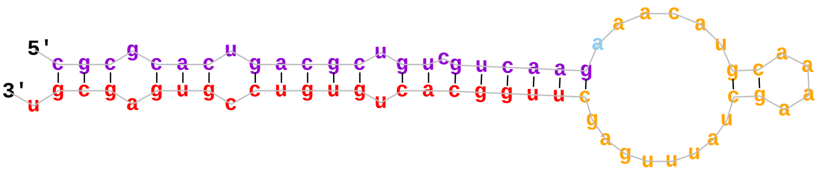

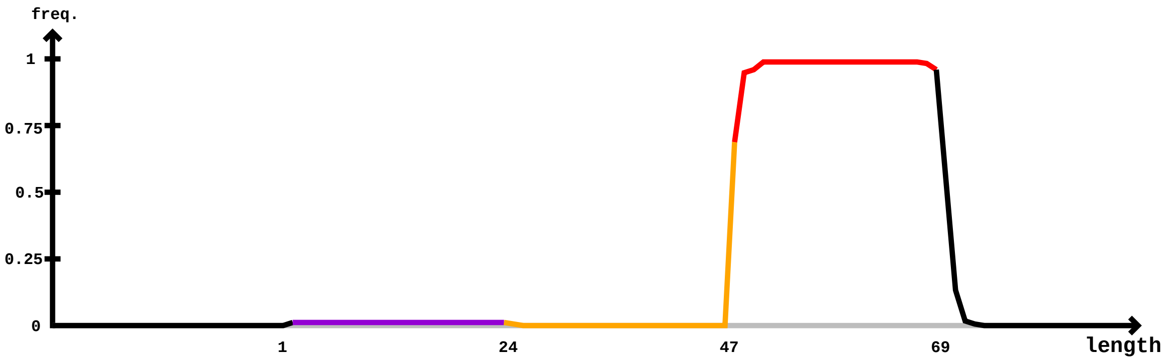

Star Mature

```
5'- aauggacgccguuggcaaggggacgcgcacugacgcugucgucaagaaacaugcaaaagcuauuugagcuuggcacuguguccgugagcgucuucuucaauuaaagcagca  -3'  obs
    aauggacgccguuggcaaggggacgcgcacugacgcugucgucaagaaacaugcaaaagcuauuugagcuuggcacuguguccgugagcgucuucuucaauuaaagcagca        exp
    .......(((...)))((((((((((.(((.(((((.((.((((((......((....))........)))))))).))))).))).))))))))))..............  reads  mm  sample
    .......................cgcgcacugacgcugucgucaag.................................................................  2      0   seq
    .....................................................................uuggcacuguguccgugagc......................  1      0   seq
    .....................................................................uuggcacuguguccgugagcU.....................  1      1   seq
    .....................................................................uuggcacuguguccgugagcg.....................  3      0   seq
    .....................................................................Auggcacuguguccgugagcgu....................  1      1   seq
    .....................................................................uuUgcacuguguccgugagcgu....................  1      1   seq
    .....................................................................uuggcacuguguccgugagcgu....................  97     0   seq
    .....................................................................uCggcacuguguccgugagcgu....................  1      1   seq
    .....................................................................uuggcacuguguccgugagcUu....................  1      1   seq
    .....................................................................uuAgcacuguguccgugagcgu....................  1      1   seq
    .....................................................................uuggcacuguguccgugagcguU...................  5      1   seq
    .....................................................................uuggcacuguguccgugagcguc...................  2      0   seq
    .....................................................................uuggcacuguguccgugagcguUu..................  3      1   seq
    .....................................................................uuggcacuguguccgugagcguUuu.................  2      1   seq
    ......................................................................uggcacugAguccgugagcgu....................  1      1   seq
    ......................................................................uggcacuguguccgugagcgu....................  6      0   seq
    ......................................................................Aggcacuguguccgugagcguc...................  1      1   seq
    ......................................................................uggcacuguguccgugagcguc...................  26     0   seq
    ......................................................................uggcacuguguccgugagcgucu..................  11     0   seq
    .......................................................................ggcacuguguccgugagcgucu..................  1      0   seq
    .......................................................................ggcacuguguccgugagcgucuuG................  1      1   seq
    ........................................................................gcacuguguccgugagcgucu..................  5      0   seq
```
