# Supplementary material for: The Small RNA Universe of Capitella teleta
Source: Front Mol Biosci. 2022 Feb 25;9:802814. doi: 10.3389/fmolb.2022.802814 (PMC8915122; doi:10.3389/fmolb.2022.802814)
Supplement: Supplementary file 1 [file DataSheet1.ZIP › Supplement/candidate/CAPTEscaffold_488_22705.pdf]

Provisional ID	:	CAPTEscaffold_488_22705
Score total	:	3627.8
Score for star read(s)	:	3.9
Score for read counts	:	3620.9
Score for mfe	:	2
Score for randfold	:	1.6
Score for cons. seed	:	-0.6
Total read count	:	7114
Mature read count	:	6948
Loop read count	:	0
Star read count	:	166

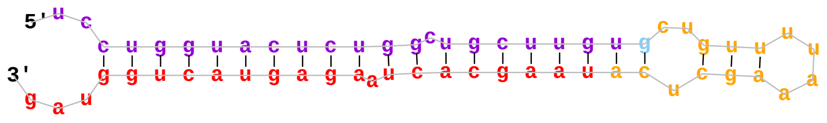

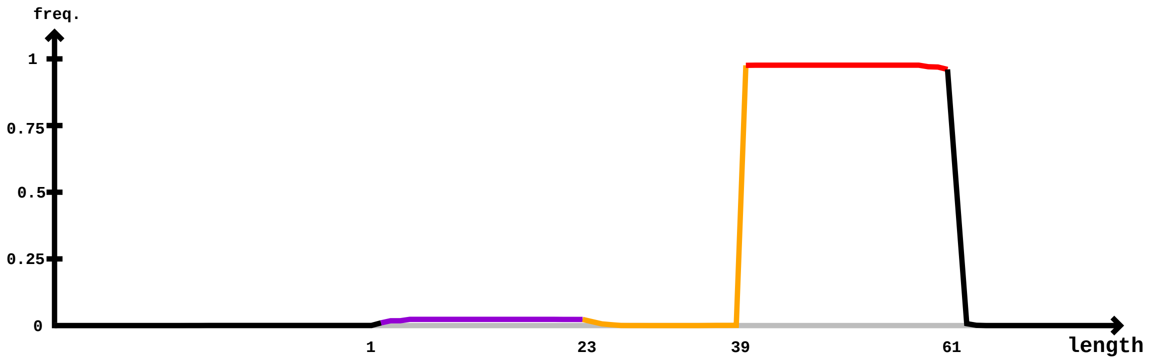


```
                                  Star                                   Mature
5'- gguggcaauuggcauguguuguaucuugccuuuccugguacucuggcugcuugugcuguuuuaaagcucauaagcacuaagaguacugguagaggcaaggacuauggcu   -3'   obs
    gguggcaauuggcauguguuguaucuugccuuuccugguacucuggcugcuugugcuguuuuaaagcucauaagcacuaagaguacugguagaggcaaggacuauggcu         exp
    .(((.(....).)))..(((((((((((((((.(((((((((((.((((((((..(((....))).))))))))).))))))))))).))))))))))..)))))).    reads   mm   sample
    ............cauguguuguaucuugccuuucc..........................................................................   1       0    seq
    ..............uguguuguaucuugccuuu............................................................................   1       0    seq
    ................................uccugguacucuggcugcuug........................................................   1       0    seq
    ................................uccugguacucuggcugcuugA.......................................................   3       1    seq
    ................................uccugguacucuggcugcuugu.......................................................   57      0    seq
    ................................uccugguacucuggcugcuuCu.......................................................   1       1    seq
    ................................uccugguacucuggcugcuuguU......................................................   2       1    seq
    ................................uccugguacucuggcugcuugug......................................................   4       0    seq
    ................................uccugguacucuggcugcuugugU.....................................................   1       1    seq
    ................................uccugguacucuggcugcuugugA.....................................................   1       1    seq
    .................................ccugguacucuggcugcu..........................................................   2       0    seq
    .................................ccugguacucuggcugcuugu.......................................................   9       0    seq
    .................................ccugguacucuggcugcuugug......................................................   41      0    seq
    .................................ccugguacucuggcugcuugugc.....................................................   2       0    seq
    .................................ccugguacucuggcugcuugugA.....................................................   2       1    seq
    .................................ccugguacucuggcugcuugugcuguu.................................................   1       0    seq
    ..................................cugguacucuggcugcuugug......................................................   1       0    seq
    ...................................ugguacucuggcugcuugugc.....................................................   17      0    seq
    ...................................ugguacucuggcugcuugugcu....................................................   19      0    seq
    ...................................ugguacucuggcugcuugugcA....................................................   1       1    seq
    ...................................ugguacucuggcugcuugugcuA...................................................   1       1    seq
    ..................................................................cucauaagcacuaagaguacugg....................   1       0    seq
    ..................................................................cucauaagcacuaagaguacuggu...................   1       0    seq
    ...................................................................ucauaagcacuaagaguacuggu...................   2       0    seq
    .....................................................................Cuaagcacuaagaguacugguag.................   1       1    seq
    ......................................................................uaagcacuaagaguacugg....................   24      0    seq
    ......................................................................uaagcaUuaagaguacugg....................   17      1    seq
    ......................................................................uaagcacuaagaguacuggu...................   1       0    seq
    ......................................................................uaagcaUuaagaguacuggu...................   4       1    seq
    ......................................................................uaagcaUuaagaguacuggua..................   63      1    seq
    ......................................................................Aaagcacuaagaguacugguag.................   6       1    seq
    ......................................................................uaagcacuaagaguacuAguag.................   4       1    seq
    ......................................................................uaagcacuaagaguacugguag.................   2415    0    seq
```

```
                                Star                                 Mature
gguggcaauuggcauguguuguaucuugccuuuccugguacucuggcugcuugugcuguuuuaaagcucauaagcacuaagaguacugguagaggcaaggacuauggcu
......................................................................uaagcacuaGgaguacugguag.................   2      1   seq
......................................................................uaagcaUuaagaguacugguag.................   3027   1   seq
......................................................................uGagcacuaagaguacugguag.................   2      1   seq
......................................................................uaagcacuaagaguacAgguag.................   1      1   seq
......................................................................uaagcacuGagaguacugguag.................   3      1   seq
......................................................................uaagcacuaagaguacuggGag.................   2      1   seq
......................................................................Naagcacuaagaguacugguag.................   2      1   seq
......................................................................uaaCcacuaagaguacugguag.................   1      1   seq
......................................................................Gaagcacuaagaguacugguag.................   3      1   seq
......................................................................uaagcacuaagaguacugguGg.................   1      1   seq
......................................................................uaagcacuaagaguacuUguag.................   2      1   seq
......................................................................uaGgcacuaagaguacugguag.................   1      1   seq
......................................................................uaCgcacuaagaguacugguag.................   1      1   seq
......................................................................uaagcacuaagaguacugUuag.................   1      1   seq
......................................................................uaagcaAuaagaguacugguag.................   2      1   seq
......................................................................uaagcacuaagaguGcugguag.................   1      1   seq
......................................................................uaagcacuaagagAacugguag.................   1      1   seq
......................................................................uaagcacAaagaguacugguag.................   3      1   seq
......................................................................uaagcacuaagaguacugguaA.................   2      1   seq
......................................................................uaaAcacuaagaguacugguaga................   1      1   seq
......................................................................uaagcacuaagaguacAgguaga................   1      1   seq
......................................................................uaagcaUuaagaguacugguaga................   1046   1   seq
......................................................................uaagcacuaagaguacuggGaga................   1      1   seq
......................................................................uaagcacuaagaguacuAguaga................   1      1   seq
......................................................................uaagcacuaagaguacugguaga................   241    0   seq
......................................................................uaagcacuaagaguacugguagC................   1      1   seq
......................................................................uaagcacuaagaguacugguagU................   3      1   seq
......................................................................uaagcacuaagaguacugguaAa................   3      1   seq
......................................................................Aaagcacuaagaguacugguaga................   2      1   seq
......................................................................uaagcaUuaagaguacugguagag...............   3      1   seq
......................................................................uaagcacuaagaguacugguagaA...............   41     1   seq
......................................................................uaagcacuaagaguacugguagagC..............   1      1   seq
......................................................................uaagcacuaagaguacugguagaAg..............   6      1   seq
.......................................................................Uagcacuaagaguacugguag.................   1      1   seq
.......................................................................aagcacuaagaguacugguag.................   2      0   seq
..........................................................................caUuaagaguacugguaga................   1      1   seq
```
