# Supplementary material for: The Small RNA Universe of Capitella teleta
Source: Front Mol Biosci. 2022 Feb 25;9:802814. doi: 10.3389/fmolb.2022.802814 (PMC8915122; doi:10.3389/fmolb.2022.802814)
Supplement: Supplementary file 1 [file DataSheet1.ZIP › Supplement/candidate/CAPTEscaffold_46_3553.pdf]

Provisional ID	:	CAPTEscaffold_46_3553
Score total	:	1.5
Score for star read(s)	:	-1.3
Score for read counts	:	0
Score for mfe	:	1.8
Score for randfold	:	1.6
Score for cons. seed	:	-0.6
Total read count	:	29
Mature read count	:	15
Loop read count	:	0
Star read count	:	14

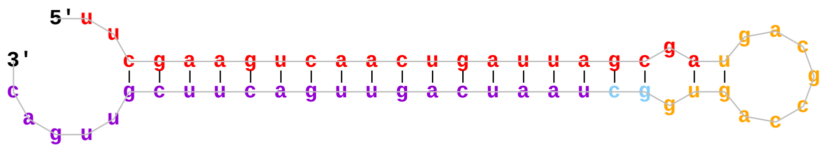

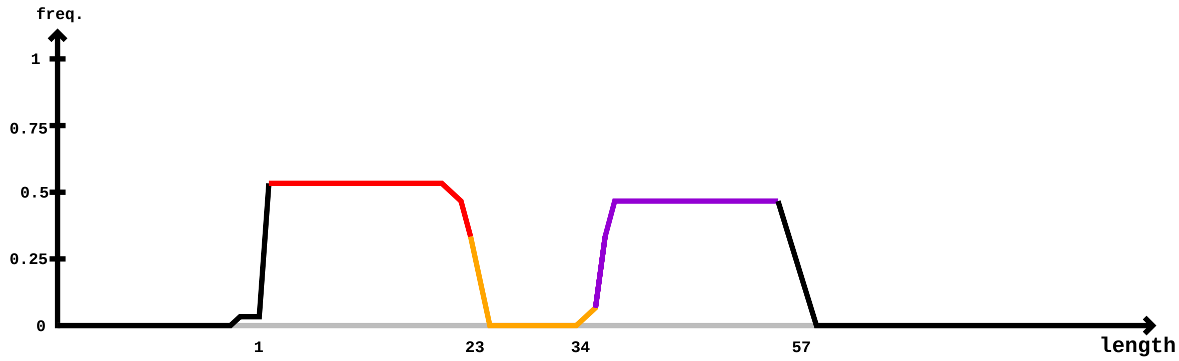

Mature Star

```
5'- uaguaaauugacuaaauuccuucgaagucaacugauuagcgaugacgccaguggcuaaucaguugacuucguugacuaaaucugaggugacuuuuuguccccuuuguuccgu   -3'    obs
    uaguaaauugacuaaauuccuucgaagucaacugauuagcgaugacgccagugggcuaaucaguugacuucguugacuaaaucugaggugacuuuuuguccccuuuguuccgu           exp
    ((((......)))).....(..((((((((((((((((((.((.......)).))))))))))))))))))..).........((((.(((.....))).))))........   reads   mm   sample
    .................uccuucgaagucaacugauuag.........................................................................   1       0    seq
    ....................uucgaagucaacugauuagc........................................................................   1       0    seq
    ....................uucgaagucaacugauuagcg.......................................................................   4       0    seq
    ....................uucAaagucaacugauuagcga......................................................................   1       1    seq
    ....................uucgaagucaacugauuagcga......................................................................   9       0    seq
    .....................................................gcuaaucaguugacuucguugac....................................   1       0    seq
    ......................................................cuaaucaguugacuucguugac....................................   1       0    seq
    .......................................................uaaucaguugacuucguugac....................................   6       0    seq
    .......................................................uaaucaguugacuucguugacu...................................   2       0    seq
    ........................................................aaucaguugacuucguugacu...................................   4       0    seq
```
