# Supplementary material for: The Small RNA Universe of Capitella teleta
Source: Front Mol Biosci. 2022 Feb 25;9:802814. doi: 10.3389/fmolb.2022.802814 (PMC8915122; doi:10.3389/fmolb.2022.802814)
Supplement: Supplementary file 1 [file DataSheet1.ZIP › Supplement/candidate/CAPTEscaffold_281_15882.pdf]

Provisional ID	: CAPTEscaffold_281_15882
Score total	: 40.8
Score for star read(s)	: 3.9
Score for read counts	: 38.9
Score for mfe	: 0.8
Score for randfold	: -2.2
Score for cons. seed	: -0.6
Total read count	: 88
Mature read count	: 87
Loop read count	: 0
Star read count	: 1

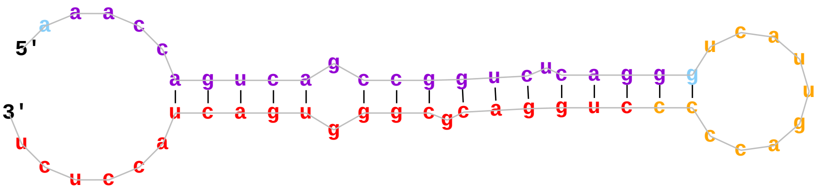

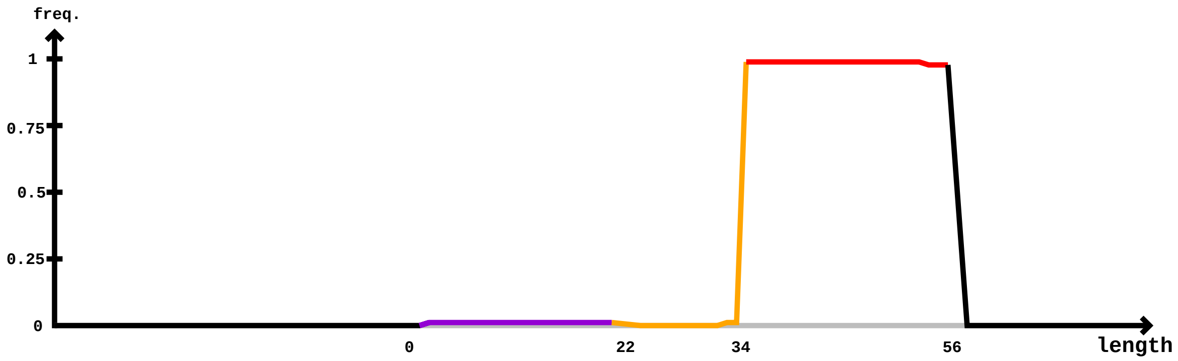

Star **Mature**

```
5'- cgucaaccaaagaaccucacccagaaugccacaucgaaaccagucagccggucucagggucauugaccccccuggacgcgggugacuaccucuacuggucagcugaugcuuuu   -3'   obs
    cgucaaccaaagaaccucacccagaaugccacaucgaaaccagucagccggucucagggucauugaccccccuggacgcgggugacuaccucuacuggucagcugaugcuuuu         exp
    ....................(((...((((....((....(((((.((((((.(((((.........)))))))).))).)))))...))...))))...)))........     reads   mm   sample
    .....................................aaccagucagccggucucagg......................................................     1       0    seq
    ....................................................................cccuggacgcgggugacuacc.......................     1       0    seq
    ......................................................................cuggacgcCggugacuaccucu....................     1       1    seq
    ......................................................................cuggacgcgggugacuaAcucu....................     1       1    seq
    ......................................................................cuggacgcgggugacuaccucu....................     83      0    seq
    ......................................................................cCggacgcgggugacuaccucu....................     1       1    seq
```
