# Supplementary material for: The Small RNA Universe of Capitella teleta
Source: Front Mol Biosci. 2022 Feb 25;9:802814. doi: 10.3389/fmolb.2022.802814 (PMC8915122; doi:10.3389/fmolb.2022.802814)
Supplement: Supplementary file 1 [file DataSheet1.ZIP › Supplement/candidate/CAPTEscaffold_973_30395.pdf]

Provisional ID	:	CAPTEscaffold_973_30395
Score total	:	241.3
Score for star read(s)	:	3.9
Score for read counts	:	234.1
Score for mfe	:	2.2
Score for randfold	:	1.6
Score for cons. seed	:	-0.6
Total read count	:	471
Mature read count	:	449
Loop read count	:	0
Star read count	:	22

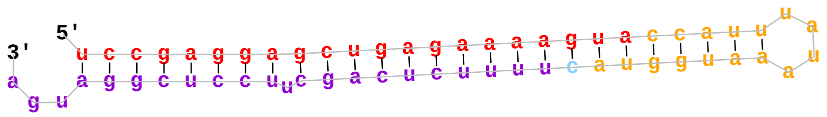

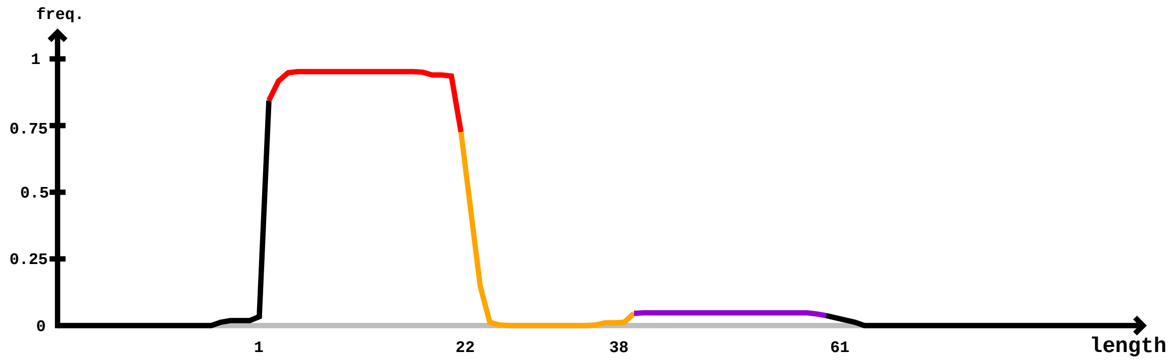

Mature Star

```
5'- acauuacugcuaccaaaccauccgaggagcugagaaaaguaccauuuauaaaugguacuuuucucagcuuccucggaugacucucgagggugugcuaagucaauuacaggu  -3'   obs
    acauuacugcuaccaaaccauccgaggagcugagaaaaguaccauuuauaaaugguacuuuucucagcuuccucggaugacucucgagggugugcuaagucaauuacaggu         exp
    ........((((((....((((((((((((((((((((((((((((....)))))))))))))))))).))))))))))..........)))).))................  reads   mm   sample
    ...............aaccauccgaggagcugagaa...........................................................................  1       0    seq
    ...............aaccauccgaggagcugagaaa..........................................................................  2       0    seq
    ...............aaccauccgaggagcugagaaaag........................................................................  2       0    seq
    ...............aaccauccgaggagcugagaaaaguaccau..................................................................  1       0    seq
    ................accauccgaggagcugagaaa..........................................................................  3       0    seq
    ...................auccgaggagcugagaaaagu.......................................................................  5       0    seq
    ...................auccgaggagcugagaaaagua......................................................................  2       0    seq
    ....................uccgaggagcugagaaaagu.......................................................................  93      0    seq
    ....................uccgaggGgcugagaaaagu.......................................................................  1       1    seq
    ....................uccAaggagcugagaaaagu.......................................................................  1       1    seq
    ....................ucUgaggagcugagaaaagu.......................................................................  1       1    seq
    ....................uccAaggagcugagaaaagua......................................................................  1       1    seq
    ....................uccgaggagcugagaaaGgua......................................................................  1       1    seq
    ....................uccgaggagcugagaaaaguU......................................................................  1       1    seq
    ....................uccgaggagcugagaaaagua......................................................................  151     0    seq
    ....................uccgaggGgcugagaaaagua......................................................................  2       1    seq
    ....................Accgaggagcugagaaaagua......................................................................  2       1    seq
    ....................uccgaggagcugaAaaaagua......................................................................  1       1    seq
    ....................uccgaggagcugagaaaaguaU.....................................................................  7       1    seq
    ....................uccgaggagcugagaaaaguac.....................................................................  106     0    seq
    ....................uccgaggagcugagaaaaguaA.....................................................................  2       1    seq
    ....................uccgaggagcugagaaaaguacc....................................................................  5       0    seq
    ....................uccgaggagcugagaaaaguacA....................................................................  6       1    seq
    ....................uccgaggagcugagaaaaguacU....................................................................  9       1    seq
    .....................ccgaggagcugagaaaaguacc....................................................................  35      0    seq
    ......................cgaggagcugagaaaaguacc....................................................................  10      0    seq
    ......................cAaggagcugagaaaaguacca...................................................................  1       1    seq
    ......................cgaggagcugagaaaaguacca...................................................................  4       0    seq
    .......................gaggagcugagaaaaguac.....................................................................  2       0    seq
    ......................................................guacuuuucucagcuuccucgga..................................  1       0    seq
    .......................................................uacuuuucucagcuuccucggau.................................  3       0    seq
    .......................................................uacuuuucucagcuuccucggauga...............................  1       0    seq
    .........................................................cuuuucucagcuuccucggauga...............................  1       0    seq
```

```
                    Mature                                  Star
acauuacugcuaccaaaccauccgaggagcugagaaaaguaccauuuauaaaugguacuuuucucagcuuccucggaugacucucgagggugugcuaagucaauuacaggu
..........................................................uuuucucagcuuccucAga..................................  1  1  seq
..........................................................uuuucucagcuuccucggauga...............................  4  0  seq
..........................................................uuuucucagcuuccucggaugU...............................  2  1  seq
..........................................................uuuucucagcuuccucggaugac..............................  4  0  seq
..........................................................uuuucucagcuuccucggaugacu.............................  5  0  seq
...........................................................uuucucagcuuccucggaugacu.............................  1  0  seq
```
