# Supplementary material for: The Small RNA Universe of Capitella teleta
Source: Front Mol Biosci. 2022 Feb 25;9:802814. doi: 10.3389/fmolb.2022.802814 (PMC8915122; doi:10.3389/fmolb.2022.802814)
Supplement: Supplementary file 1 [file DataSheet1.ZIP › Supplement/candidate/CAPTEscaffold_32_3388.pdf]

Provisional ID	:	CAPTEscaffold_32_3388
Score total	:	78.4
Score for star read(s)	:	3.9
Score for read counts	:	71.5
Score for mfe	:	2
Score for randfold	:	1.6
Score for cons. seed	:	-0.6
Total read count	:	152
Mature read count	:	135
Loop read count	:	0
Star read count	:	17

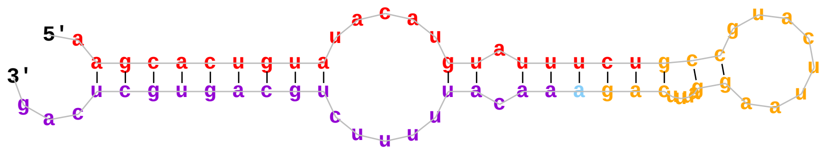

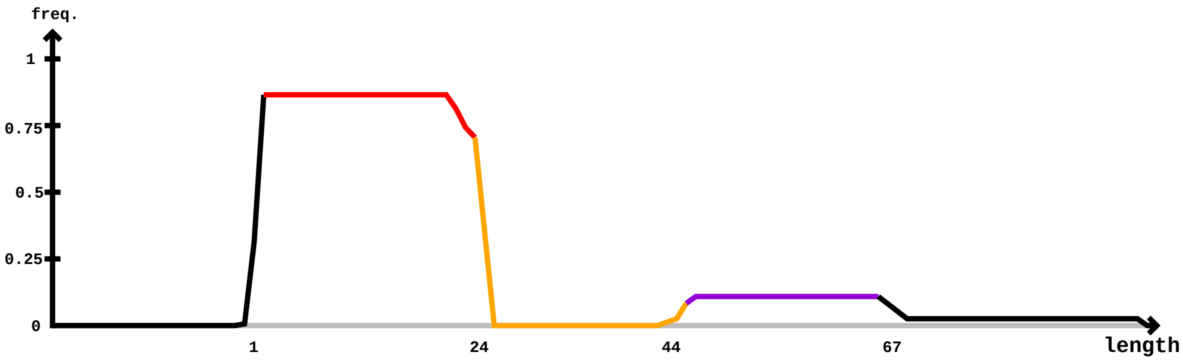


```
                         Mature                                      Star
5'- uuaaguuacagugcucagccaagcacuguauacauguauuucugccguacuuaaggauuucagaaacauuuuucugcagugcucagcugagcacuguacauuaugccacgccu   -3'    obs
    uuaaguuacagugcucagccaagcacuguauacauguauuucugccguacuuaaggauuucagaaacauuuuucugcagugcucagcugagcacuguacauuaugccacgccu          exp
    .(((..(((((((((((((..(((((((((.....((.((((((((........))....)))))).)).....)))))))))..)))))))))))))..)))..........   reads    mm    sample
    ..................ccaagcacuguauacauguauuu........................................................................   1        0     seq
    ...................caagcacuguauacauguauu.........................................................................   8        0     seq
    ...................caagcacuguauacauguauuu........................................................................   10       0     seq
    ...................caagcacuguauacauguauuucu......................................................................   30       0     seq
    ....................aagcacuguauacauguauuuc.......................................................................   6        0     seq
    ....................aagcacAguauacauguauuucu......................................................................   1        1     seq
    ....................aagcacuguauacauguauuucu......................................................................   76       0     seq
    ....................Gagcacuguauacauguauuucu......................................................................   1        1     seq
    ....................aagcacuguauacauguauuucuA.....................................................................   1        1     seq
    ....................aagcacuguauacauguauuucug.....................................................................   1        0     seq
    ..............................................................gaaacauuuuucugcagugcucU............................   2        1     seq
    ...............................................................aaacauuuuucugcagugcucag...........................   1        0     seq
    ...............................................................aaacauuuuucugcagugcucagc..........................   1        0     seq
    ................................................................aacauuuuucugcagugcucag...........................   8        0     seq
    ................................................................aacauuuuucugcagugcucagA..........................   1        1     seq
    .................................................................acauuuuucugcagugcucag...........................   3        0     seq
    .................................................................acauuuuucugcagugcucagc..........................   1        0     seq
    ......................................................................................cuAagcacuguacauuaugccacgcc.   1        1     seq
    ......................................................................................cugagcacuguacauuaugccacgcc.   3        0     seq
```
