# Supplementary material for: The Small RNA Universe of Capitella teleta
Source: Front Mol Biosci. 2022 Feb 25;9:802814. doi: 10.3389/fmolb.2022.802814 (PMC8915122; doi:10.3389/fmolb.2022.802814)
Supplement: Supplementary file 1 [file DataSheet1.ZIP › Supplement/candidate/CAPTEscaffold_96_9145.pdf]

Provisional ID	:	CAPTEscaffold_96_9145
Score total	:	99.6
Score for star read(s)	:	3.9
Score for read counts	:	92.4
Score for mfe	:	2.3
Score for randfold	:	1.6
Score for cons. seed	:	-0.6
Total read count	:	193
Mature read count	:	181
Loop read count	:	0
Star read count	:	12

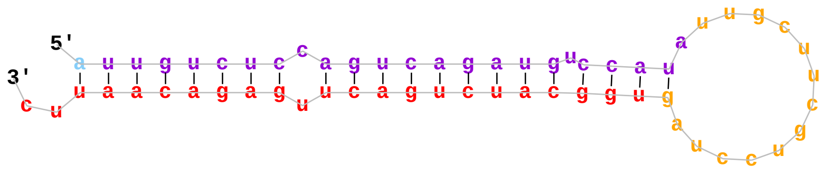

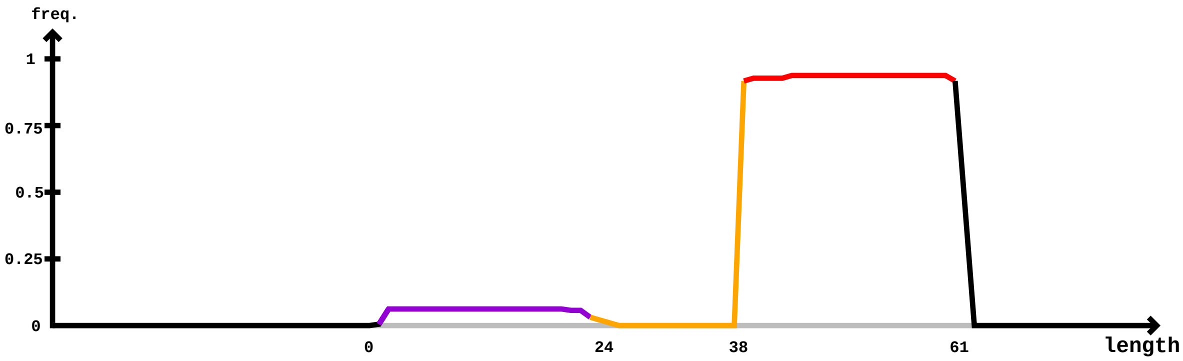

Star Mature

```
5'- gcaucacgauuauaugcugagcaccgguugauauugucuccagucagauguccauauugcuucguccuaguggcaucugacuugagacaauuccaaccggugcaaugaacagu  -3'   obs
    gcaucacgauuauaugcugagcaccgguugauauugucuccagucagauguccauauugcuucguccuaguggcaucugacuugagacaauuccaaccggugcaaugaacagu         exp
    ((((.........))))...((((((((((..((((((((.(((((((((.((((.............))))))))))))).))))))))..)))))))))).........   reads    mm    sample
    ................................auugucuccagucagauguc.............................................................   1        0     seq
    .................................uugucuccagucagaugucca...........................................................   5        0     seq
    .................................uugucuccagucagauguccau..........................................................   1        0     seq
    .................................uugucuccagucagauguccauU.........................................................   3        1     seq
    .................................uugucuccagucagauguccaua.........................................................   2        0     seq
    ......................................................................uggcaucugacuugagacaauu.....................   4        0     seq
    ......................................................................ugAcaucugacuugagacaauuc....................   1        1     seq
    ......................................................................uAgcaucugacuugagacaauuc....................   1        1     seq
    ......................................................................uggcaucugacuugagacaauuc....................   171      0     seq
    .......................................................................ggcaucugacuugagacaauuc....................   2        0     seq
    ...........................................................................ucugacuugagacaauuc....................   2        0     seq
```
