# Supplementary material for: The Small RNA Universe of Capitella teleta
Source: Front Mol Biosci. 2022 Feb 25;9:802814. doi: 10.3389/fmolb.2022.802814 (PMC8915122; doi:10.3389/fmolb.2022.802814)
Supplement: Supplementary file 1 [file DataSheet1.ZIP › Supplement/candidate/CAPTEscaffold_4928_39836.pdf]

Provisional ID	:	CAPTEscaffold_4928_39836
Score total	:	18.2
Score for star read(s)	:	3.9
Score for read counts	:	11.3
Score for mfe	:	1.9
Score for randfold	:	1.6
Score for cons. seed	:	-0.6
Total read count	:	34
Mature read count	:	19
Loop read count	:	0
Star read count	:	15

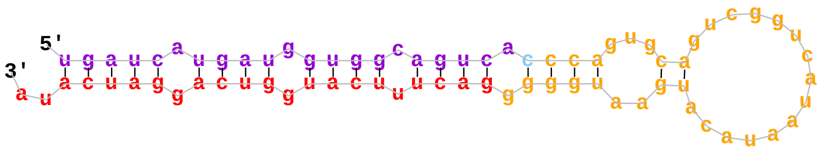

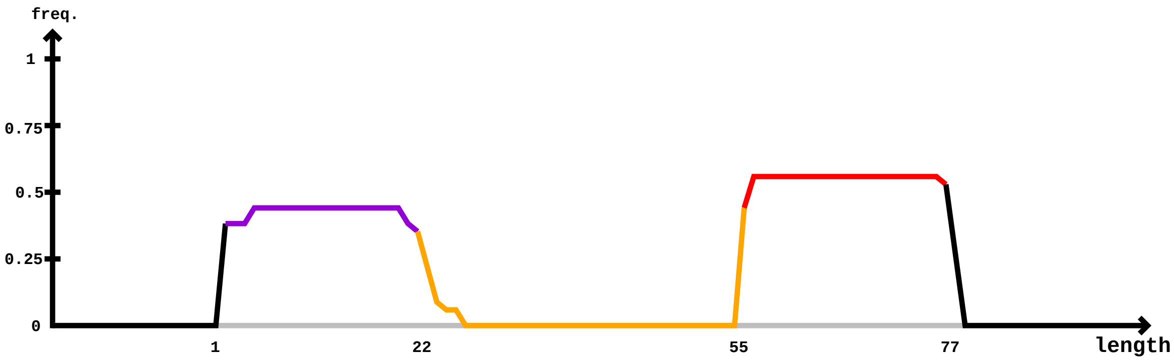

Star **Mature**

```
5'- ucucugcgucuagguaugaucaugaugguggcagucacccagugcagucggucauaauacaugaaugggggacuuucauggucaggaucauaccugaaguagaaaauucaag  -3'   obs
    ucucugcgucuagguaugaucaugaugguggcagucacccagugcagucggucauaauacaugaaugggggacuuucauggucaggaucauaccugaaguagaaaauucaag         exp
    ..(((((.((.((((((((((.((((.((((.((((.((((...((..............))..)))).)))).)))).)))).))))))))))).))))).........   reads   mm   sample
    ................ugaucaugaugguggcagu.............................................................................   2       0    seq
    ................ugaucaugaugguggcaguc............................................................................   1       0    seq
    ................ugaucaugaugguggcaguca...........................................................................   5       0    seq
    ................ugaucaugaugguggcagucac..........................................................................   4       0    seq
    ................ugaucaugaugguggcagucacc.........................................................................   1       0    seq
    ...................ucaugaugguggcagucaccca.......................................................................   2       0    seq
    ......................................................................gacuuucauggucaggaucau.....................   1       0    seq
    ......................................................................gacuuucauggucaggaucaua....................   14      0    seq
    .......................................................................acuuucauggucaggaucaua....................   4       0    seq
```
