# Supplementary material for: The Small RNA Universe of Capitella teleta
Source: Front Mol Biosci. 2022 Feb 25;9:802814. doi: 10.3389/fmolb.2022.802814 (PMC8915122; doi:10.3389/fmolb.2022.802814)
Supplement: Supplementary file 1 [file DataSheet1.ZIP › Supplement/candidate/CAPTEscaffold_281_15857.pdf]

Provisional ID	:	CAPTEscaffold_281_15857
Score total	:	18.2
Score for star read(s)	:	3.9
Score for read counts	:	11.3
Score for mfe	:	1.9
Score for randfold	:	1.6
Score for cons. seed	:	-0.6
Total read count	:	34
Mature read count	:	19
Loop read count	:	0
Star read count	:	15

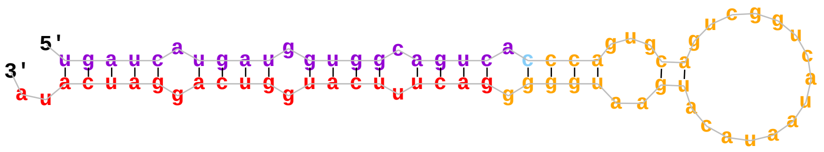

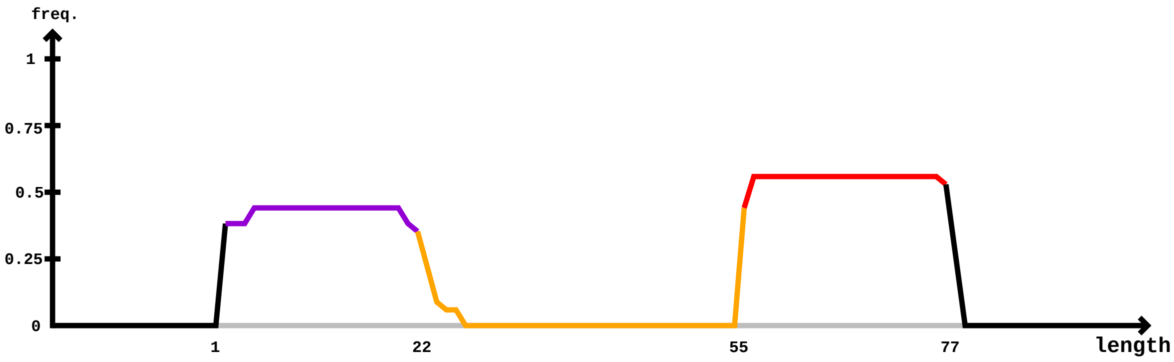


```
                Star                                                   Mature
5'- ucucugcgucuaggua ugaucaugaugguggcaguca cccagugcagucggucauaauacaugaaugggg gacuuucauggucaggaucaua ccugaaguagaaaauucaag   -3'   obs
ucucugcgucuagguaugaucaugaugguggcagucacccagugcagucggucauaauacaugaauggggacuuucauggucaggaucauaccugaaguagaaaauucaag               exp
..(((((.((.((((((((((.((((.((((.((((.((((...((..............))..)))).)))).)))).)))).)))))))))))).)))))..........   reads   mm   sample
................ugaucaugaugguggcagu.............................................................................   2       0    seq
................ugaucaugaugguggcaguc............................................................................   1       0    seq
................ugaucaugaugguggcaguca...........................................................................   5       0    seq
................ugaucaugaugguggcagucac..........................................................................   4       0    seq
................ugaucaugaugguggcagucacc.........................................................................   1       0    seq
...................ucaugaugguggcagucaccca.......................................................................   2       0    seq
......................................................................gacuuucauggucaggaucau.....................   1       0    seq
......................................................................gacuuucauggucaggaucaua....................   14      0    seq
.......................................................................acuuucauggucaggaucaua....................   4       0    seq
```
