# Supplementary material for: The Small RNA Universe of Capitella teleta
Source: Front Mol Biosci. 2022 Feb 25;9:802814. doi: 10.3389/fmolb.2022.802814 (PMC8915122; doi:10.3389/fmolb.2022.802814)
Supplement: Supplementary file 1 [file DataSheet1.ZIP › Supplement/candidate/CAPTEscaffold_463_22033.pdf]

| | | |
|---|---|---|
| Provisional ID | : | CAPTEscaffold_463_22033 |
| Score total | : | 1.6 |
| Score for star read(s) | : | -1.3 |
| Score for read counts | : | 0 |
| Score for mfe | : | 1.9 |
| Score for randfold | : | 1.6 |
| Score for cons. seed | : | -0.6 |
| Total read count | : | 37 |
| Mature read count | : | 22 |
| Loop read count | : | 0 |
| Star read count | : | 15 |

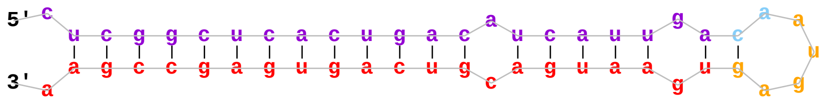


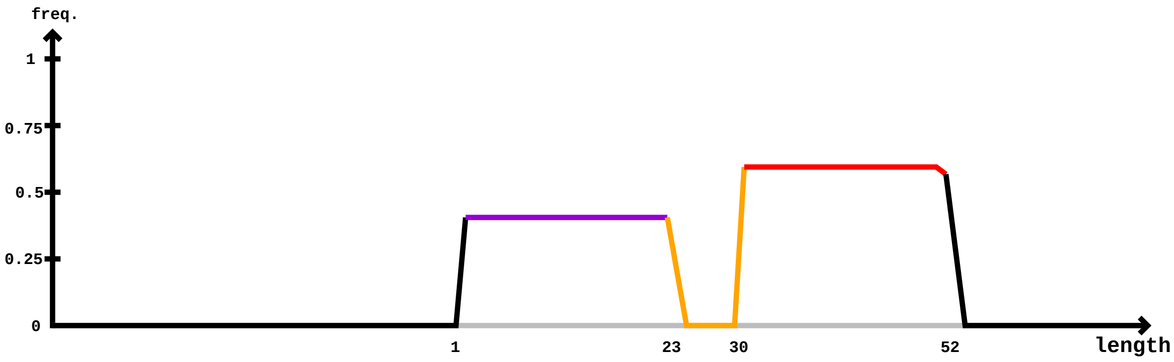


```
                                         Star                       Mature
5'- gagccuaucaacuuaucucaaggguucaaagcaaacccccacucggcucacugacaucauugacaaugagugaaugacgucagugagccgaaugacguguucguuuccaaaa   -3'   obs
    gagccuaucaacuuaucucaaggguucaaagcaaaccccccacucggcucacugacaucauugacaaugagugaaugacgucagugagccgaaugacguguucguuuccaaaa         exp
    ((((((...............)))))).((((.((((..((.(((((((((((((.(((((.((.....)).))))).))))))))))))).))..).))).))))......   reads   mm   sample
    .........................................cucggcucacugacaucauuga.................................................   15      0    seq
    .....................................................................ugaaugacgucagugagccga......................   1       0    seq
    .....................................................................ugaaugacgucagugagccgaa.....................   21      0    seq
```
