# Supplementary material for: The Small RNA Universe of Capitella teleta
Source: Front Mol Biosci. 2022 Feb 25;9:802814. doi: 10.3389/fmolb.2022.802814 (PMC8915122; doi:10.3389/fmolb.2022.802814)
Supplement: Supplementary file 1 [file DataSheet1.ZIP › Supplement/candidate/CAPTEscaffold_20545_46059.pdf]

Provisional ID	:	CAPTEscaffold_20545_46059
Score total	:	24.3
Score for star read(s)	:	3.9
Score for read counts	:	23.1
Score for mfe	:	0.1
Score for randfold	:	-2.2
Score for cons. seed	:	-0.6
Total read count	:	57
Mature read count	:	50
Loop read count	:	0
Star read count	:	7

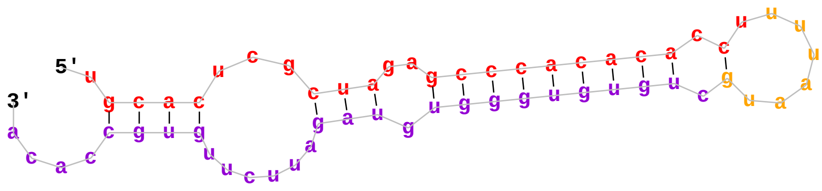

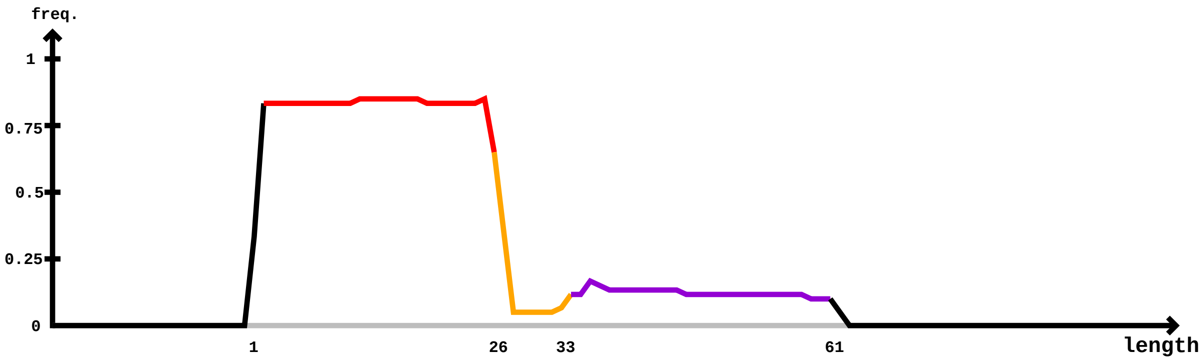

Mature Star

```
5'- aacauuaacaaaguuucgagugcacucgcuagagcccacacaccuuuuaaugcuguguggguguagauucuugugccacaguaauucagcaacuucuccccguuguuucauuauc   -3'    obs
    aacauuaacaaaguuucgagugcacucgcuagagcccacacaccuuuuaaugcuguguggguguagauucuugugccacaguaauucagcaacuucuccccguuguuucauuauc          exp
    ...................((((((...(((..(((((((((.(.......).))))))))).)))......))).))).(((((..((((((........))))))..))))).  reads    mm    sample
    ...................gugcacucgcuagagccc..............................................................................  2        0     seq
    ...................gugcacucgcuagagcccacacacc.......................................................................  5        0     seq
    ...................gGgcacucgcuagagcccacacacc.......................................................................  1        1     seq
    ...................gugcacucgcuagagcccacacaccu......................................................................  11       0     seq
    ...................gGgcacucgcuagagcccacacaccu......................................................................  1        1     seq
    ....................ugcacucgcuagagcccacacacc.......................................................................  6        0     seq
    ....................ugcacucgcuagagcccacacaccu......................................................................  24       0     seq
    ...............................agagcccacacaccuuuuaaugcugu..........................................................  1        0     seq
    ....................................acacaccuuuuaaugcug.............................................................  1        0     seq
    ..........................................cuuuuaaugcugugugggugu....................................................  1        0     seq
    ...................................................gcuguguggguguagauucuugugccaca...................................  1        0     seq
    ....................................................cuguguggguguagauucuugugccaca...................................  3        0     seq
    ......................................................guguggguguagauucuugugcc......................................  1        0     seq
    ......................................................guguggguguagauucuugugccaca...................................  2        0     seq
```
