# Supplementary material for: The Small RNA Universe of Capitella teleta
Source: Front Mol Biosci. 2022 Feb 25;9:802814. doi: 10.3389/fmolb.2022.802814 (PMC8915122; doi:10.3389/fmolb.2022.802814)
Supplement: Supplementary file 1 [file DataSheet1.ZIP › Supplement/candidate/CAPTEscaffold_60_5428.pdf]

Provisional ID	:	CAPTEscaffold_60_5428
Score total	:	107.8
Score for star read(s)	:	3.9
Score for read counts	:	102.1
Score for mfe	:	0.8
Score for randfold	:	1.6
Score for cons. seed	:	-0.6
Total read count	:	212
Mature read count	:	206
Loop read count	:	0
Star read count	:	6

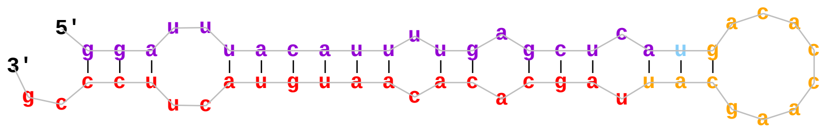

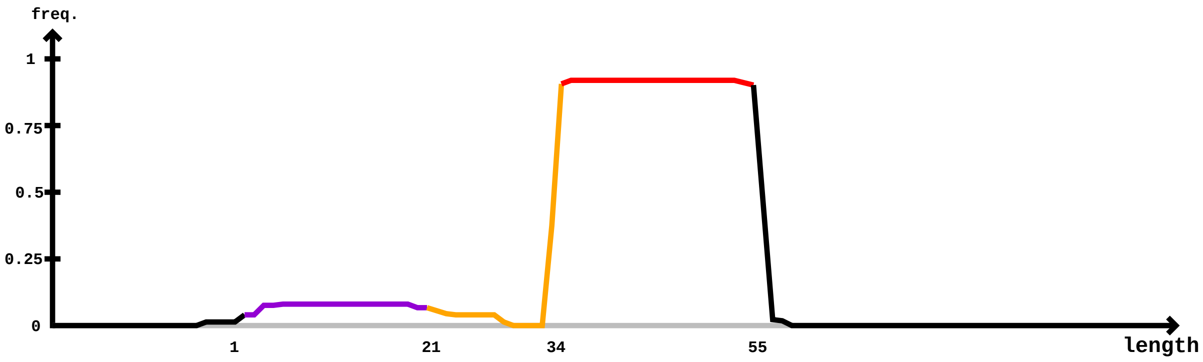

Star Mature

```
5'- ugcauagauuauauuuggggauuuacauuuugagcucaugacaccaagcauuagcacacaauguacuucccgaaauauaggcuucucgcuucgugauuauuuccacuguguacuu   -3'   obs
    ugcauagauuauauuuggggauuuacauuuugagcucaugacaccaagcauuagcacacaauguacuucccgaaauauaggcuucucgcuucgugauuauuuccacuguguacuu         exp
    (((((((..(((((((.((((..((((((.((.(((.(((........))).))).)).))))))..)))).)))))))(((.....)))...............)))))))...   reads   mm   sample
    ..............uuggggauuuaAauuuugagcu...............................................................................   3       1    seq
    ..................ggauuuacauuuugagcuca.............................................................................   3       0    seq
    ..................ggauuuacauuuugagcucau............................................................................   2       0    seq
    ..................ggauuuacauuuugagcucaug...........................................................................   1       0    seq
    ....................auuuacauuuugagcucaugacacc......................................................................   5       0    seq
    ....................auuuacauuuugagcucaugacacca.....................................................................   3       0    seq
    ......................uuacauuuugagcucaugacacc......................................................................   1       0    seq
    ..................................................uuagcacacaauguacuucc.............................................   1       0    seq
    ..................................................uuagcacacaauguacuuccc............................................   1       0    seq
    ..................................................uuagcacacaauguacuucAcg...........................................   1       1    seq
    ..................................................uuagcacacaauguacuucccU...........................................   5       1    seq
    ..................................................uuagcacacaauguacuucccg...........................................   71      0    seq
    ..................................................Auagcacacaauguacuucccg...........................................   1       1    seq
    ..................................................uuagcacacaauguacuucccga..........................................   3       0    seq
    ..................................................uuagcacUcaauguacuucccgaaa........................................   1       1    seq
    ...................................................uagcacacaauguacuucc.............................................   1       0    seq
    ...................................................uagcacacaauguacuuccc............................................   1       0    seq
    ...................................................uagcacacaauguacuucccg...........................................   94      0    seq
    ...................................................uagcacacaauguacuucccU...........................................   1       1    seq
    ...................................................uagcacacaauguacuucccgU..........................................   12      1    seq
    ...................................................uagcacacaauguacuucccga..........................................   6       0    seq
    ...................................................uagcacacaauguacuucccgaC.........................................   1       1    seq
    ...................................................uagcacacaauguacuucccgaaa........................................   3       0    seq
    ....................................................agcacacaauguacuucccgU..........................................   2       1    seq
    ....................................................agcacacaauguacuucccga..........................................   1       0    seq
```
