# Supplementary material for: The Small RNA Universe of Capitella teleta
Source: Front Mol Biosci. 2022 Feb 25;9:802814. doi: 10.3389/fmolb.2022.802814 (PMC8915122; doi:10.3389/fmolb.2022.802814)
Supplement: Supplementary file 1 [file DataSheet1.ZIP › Supplement/candidate/CAPTEscaffold_452_22339.pdf]

Provisional ID	:	CAPTEscaffold_452_22339
Score total	:	10.1
Score for star read(s)	:	3.9
Score for read counts	:	2.2
Score for mfe	:	3
Score for randfold	:	1.6
Score for cons. seed	:	-0.6
Total read count	:	16
Mature read count	:	8
Loop read count	:	0
Star read count	:	8

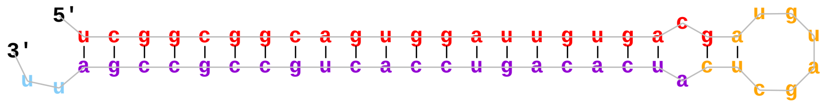

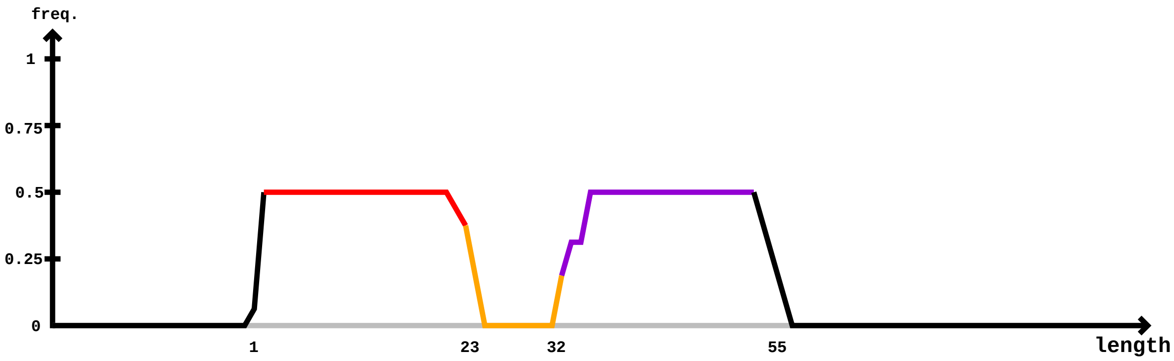

Mature Star

```
5'- ucuugaccacaccaaagagaucggcggcaguggauugugacgauguagcucaucacaguccacugccgccgauuucuuugguguggucaagauauaucucgguggcugcaau  -3'  obs
    ucuugaccacaccaaagagaucggcggcaguggauugugacgauguagcucaucacaguccacugccgccgauuucuuugguguggucaagauauaucucgguggcugcaau        exp
    ((((((((((((((((((((((((((((((((((((((((.((......)).)))))))))))))))))))))))))))))))))))))))))....................     reads    mm    sample
    ...................aucggcggcaguggauugugG........................................................................     1        1     seq
    ....................ucggcggcaguggauugugaU.......................................................................     1        1     seq
    ....................ucggcggcaguggauugugacg......................................................................     6        0     seq
    ...................................................aucacaguccacugccgccga........................................     3        0     seq
    ....................................................ucacaguccacuAccgccgauu......................................     2        1     seq
    ......................................................acaguccacuAccgccgauu......................................     3        1     seq
```
