# Supplementary material for: The Small RNA Universe of Capitella teleta
Source: Front Mol Biosci. 2022 Feb 25;9:802814. doi: 10.3389/fmolb.2022.802814 (PMC8915122; doi:10.3389/fmolb.2022.802814)
Supplement: Supplementary file 1 [file DataSheet1.ZIP › Supplement/candidate/CAPTEscaffold_324_18354.pdf]

| | | |
|---|---|---|
| Provisional ID | : | CAPTEscaffold_324_18354 |
| Score total | : | 1.5 |
| Score for star read(s) | : | -1.3 |
| Score for read counts | : | 0 |
| Score for mfe | : | 1.8 |
| Score for randfold | : | 1.6 |
| Score for cons. seed | : | -0.6 |
| Total read count | : | 6560 |
| Mature read count | : | 6395 |
| Loop read count | : | 0 |
| Star read count | : | 165 |

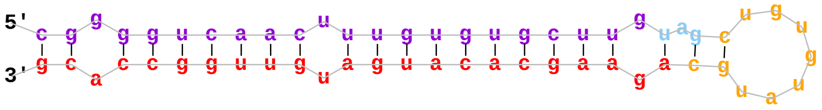

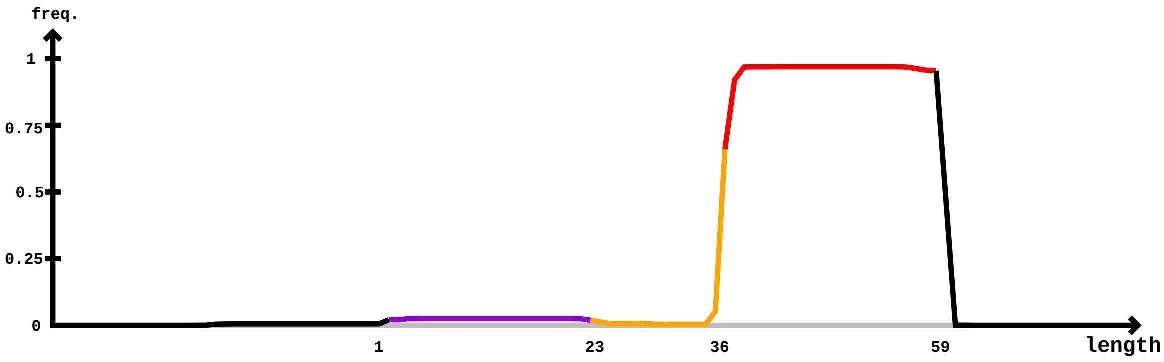


```
                                  Star                              Mature
5'- uuucagguuugccuauauauacuugggcgccacugcggggucaacuuugugugcuuguagcuguguaugcagaagcacaugauguuggccacgccgagacgucauccaauucg  -3'   obs
    uuucagguuugccuauauauacuugggcgccacugcggggucaacuuugugugcuuguagcuguguaugcagaagcacaugauguuggccacgccgagacgucauccaauucg         exp
    .....(((..(((((......))))))))...(((.(((((((.((((((((((.(.((.......))).)))))))))).))))))).)))...................   reads   mm   sample
    .............uauauacuugggcgccacug..............................................................................   1       0    seq
    ..............auauacuugggcgccacug..............................................................................   2       0    seq
    ..............auauacuugggcgccacugcg............................................................................   1       0    seq
    ...............Aauacuugggcgccacug..............................................................................   1       1    seq
    ...............uauacuugggcgccacug..............................................................................   10      0    seq
    ...............uauacuugggcgccacugc.............................................................................   1       0    seq
    ...............uauacuuggAcgccacugc.............................................................................   1       1    seq
    ...............uauacuuggAcgccacugcg............................................................................   2       1    seq
    ...............uauacuugggcgccacugcg............................................................................   9       0    seq
    ................auacuugggcgccacugc.............................................................................   1       0    seq
    ................auacuugggcgccacugcg............................................................................   4       0    seq
    .................uacuugggcgccacugcg............................................................................   2       0    seq
    ................................gcggggucaacuuugugugcu..........................................................   1       0    seq
    .................................cggggucaacuuugugugc...........................................................   1       0    seq
    .................................cggggucaacuuugugugcu..........................................................   4       0    seq
    .................................cggggucaacuuugugugcuu.........................................................   34      0    seq
    .................................cAgggucaacuuugugugcuu.........................................................   1       1    seq
    .................................cggggucaacuuugugugcuug........................................................   41      0    seq
    .................................Nggggucaacuuugugugcuug........................................................   1       1    seq
    .................................cggggGcaacuuugugugcuug........................................................   1       1    seq
    .................................cggggucaacuuugugugcuuU........................................................   5       1    seq
    .................................cggggucaacuAugugugcuug........................................................   1       1    seq
    .................................cggggucaacuuugugugcuuC........................................................   11      1    seq
    .................................cggggucaacuuugugugcuugu.......................................................   10      0    seq
    .................................cggggucaacuuugugugcuugA.......................................................   7       1    seq
    ..................................ggggucaacuuugugugcuugu.......................................................   2       0    seq
    ...................................gggucaUcuuugugugcuugu.......................................................   1       1    seq
    ...................................gggucaacuuugugugcuugu.......................................................   1       0    seq
    ...................................gggucaUcuuugugugcuugua......................................................   1       1    seq
    ...................................gggucaacuuugugugcuugua......................................................   1       0    seq
    ...................................gggucaacuuugugugcuuguagU....................................................   1       1    seq
    ...................................gggucaacuuugugugcuuguagc....................................................   7       0    seq
    ...................................gggucaacuuugugAgcuuguagc....................................................   1       1    seq
```

```
                                 Star                              Mature
uuucagguuugccuauauacuugggcgccacugcggggucaacuuugugugcuuguagcuguguaugcagaagcacaugauguuggccacgccgagacgucauccaauucg
...................................gggucaUcuuugugugcuuguagc....................................................    10    1    seq
...................................gggucaacuuugugugcuuguagcu...................................................    10    0    seq
...................................gggucaacuuugugugcuuguagcug..................................................    7     0    seq
...................................gggucaacuuugugugcuuguagcuU..................................................    1     1    seq
...................................gggucaacuuugugugcuuguagcugu.................................................    1     0    seq
...................................gggucaacuuugugugcuuguagcuCu.................................................    1     1    seq
...................................gggucaUcuuugugugcuuguagcugugu...............................................    1     1    seq
.....................................gucaacuuugugugcuuguagcu...................................................    3     0    seq
.........................................................gcuguguaugcGgaagcacaugauguuggc........................    1     1    seq
...........................................................uguguaugcagaagcacaugauguuggcc.......................    18    0    seq
...........................................................uguguaugcagaagcacaugauguuggccU......................    3     1    seq
...........................................................uguguaugcagaagcacaugauguuggcca......................    1     0    seq
...........................................................uguguaugcagaagcacaugauguuggccac.....................    2     0    seq
...............................................................uaugcagaagcacaugauguuggcc.......................    1     0    seq
................................................................augcagaagcacaugauguuggcc.......................    1     0    seq
...................................................................cagaagcacaugauguuggcc.......................    16    0    seq
...................................................................cagaagcacauAauguuggcca......................    1     1    seq
...................................................................cagaagcacaugauguugUcca......................    1     1    seq
...................................................................cagaagcacaugauguuggcca......................    18    0    seq
...................................................................cagaagcacaugauguuggccac.....................    4     0    seq
...................................................................cagaagUacaugauguuggccacg....................    1     1    seq
...................................................................cagaagcacaugauguuggccacU....................    3     1    seq
...................................................................cagaagcacaugauguuAgccacg....................    1     1    seq
...................................................................cagaagcacauAauguuggccacg....................    1     1    seq
...................................................................cagaagcacaugauguuggcAacg....................    4     1    seq
...................................................................cagaagcacaugauguuggccacg....................    264   0    seq
...................................................................cagaagAacaugauguuggccacg....................    1     1    seq
...................................................................cagaagcacaugauguuggccacgA...................    3     1    seq
....................................................................agaagcacaugauguuggc........................    5     0    seq
....................................................................agaagcacaugauguuggcc.......................    2     0    seq
....................................................................agaagcacaugauguuggcca......................    10    0    seq
....................................................................agaagcacaugauguuggccac.....................    3     0    seq
....................................................................aNaagcacaugauguuggccacg....................    1     1    seq
....................................................................agaagcacaugaugAuggccacg....................    3     1    seq
....................................................................agaagcacaugauguugAccacg....................    1     1    seq
....................................................................agaagcacauUauguuggccacg....................    1     1    seq
....................................................................agaagcacaugauguCggccacg....................    1     1    seq
....................................................................agaaUcacaugauguuggccacg....................    1     1    seq
....................................................................agaagcacaugauguuggccacA....................    1     1    seq
....................................................................agaagcacaugauguuggccacC....................    1     1    seq
....................................................................agaagcacaugauguuCgccacg....................    1     1    seq
....................................................................agaagcacaugauguuUgccacg....................    1     1    seq
....................................................................agaagcacaugauguuggccacg....................    3851  0    seq
....................................................................agaagcacaugauAuuggccacg....................    1     1    seq
....................................................................agaagcacaugauguuAgccacg....................    2     1    seq
....................................................................aUaagcacaugauguuggccacg....................    1     1    seq
....................................................................agaagcacaugaAguuggccacg....................    1     1    seq
....................................................................agaagcacaugauguAggccacg....................    6     1    seq
....................................................................agaGgcacaugauguuggccacg....................    2     1    seq
....................................................................agaagcacaugauguuggccGcg....................    1     1    seq
....................................................................agaagcacaAgauguuggccacg....................    2     1    seq
....................................................................agaagcacaugauguuggcAacg....................    110   1    seq
....................................................................agaagcacaugaugCuggccacg....................    1     1    seq
....................................................................agaagcacaugauguuggccaUg....................    1     1    seq
....................................................................agaagcacaugauguuggccaAg....................    2     1    seq
....................................................................agaagcacaugauUuuggccacg....................    2     1    seq
....................................................................agaagcGcaugauguuggccacg....................    2     1    seq
....................................................................agaagcacauAauguuggccacg....................    9     1    seq
....................................................................agaagcacaugauguuggUcacg....................    1     1    seq
....................................................................agaagcacaugauguuggGcacg....................    1     1    seq
....................................................................agaagcacaugauguuggccacgA...................    4     1    seq
.....................................................................gaagcacaugauguuggc........................    2     0    seq
.....................................................................gaagcacaugauguuggcc.......................    3     0    seq
.....................................................................gaagcacaugauguuggcca......................    4     0    seq
.....................................................................gaagcacaugauguuggccac.....................    2     0    seq
.....................................................................gaagcGcaugauguuggccacg....................    1     1    seq
.....................................................................gaagcacauCauguuggccacg....................    1     1    seq
.....................................................................gaagcacaugaCguuggccacg....................    1     1    seq
.....................................................................gaagcacaugaugAuggccacg....................    4     1    seq
.....................................................................gaagcacaugauguuggccaGg....................    1     1    seq
```

```
                                 Star                                 Mature
uuucagguuugccuauauacuugggcgccacugcggggucaacuuugugugcuuguagcuguguaugcagaagcacaugauguuggccacgccgagacgucauccaauucg
.....................................................................gaagUacaugauguuggccacg....................    1      1     seq
.....................................................................gaagcacaugauguuggccacg....................    1604   0     seq
.....................................................................gaagcacaugauguAggccacg....................    2      1     seq
.....................................................................gUagcacaugauguuggccacg....................    1      1     seq
.....................................................................gaagcacaugauguuggGcacg....................    1      1     seq
.....................................................................Uaagcacaugauguuggccacg....................    5      1     seq
.....................................................................gaagcacaugauguuCgccacg....................    1      1     seq
.....................................................................gaaUcacaugauguuggccacg....................    1      1     seq
.....................................................................gaagcacGugauguuggccacg....................    1      1     seq
.....................................................................gaagcacaugaugCuggccacg....................    1      1     seq
.....................................................................Caagcacaugauguuggccacg....................    2      1     seq
.....................................................................gaagcacaugauguuggcAacg....................    42     1     seq
.....................................................................gaagcaAaugauguuggccacg....................    1      1     seq
.....................................................................gaagcacauAauguuggccacg....................    3      1     seq
.....................................................................Aaagcacaugauguuggccacg....................    22     1     seq
.....................................................................gaagcacaugauguCggccacg....................    1      1     seq
.....................................................................gaagcacaugauguuggUcacg....................    1      1     seq
.....................................................................gaagcacaugauguuAgccacg....................    1      1     seq
.....................................................................gaagcacaugauguuggccacA....................    1      1     seq
.....................................................................gGagcacaugauguuggccacg....................    1      1     seq
.....................................................................gaagcacaugGuguuggccacg....................    1      1     seq
.....................................................................gaagcacaugauguuggccacUc...................    1      1     seq
.....................................................................gaagcacaugauguuggccacgc...................    1      0     seq
......................................................................aagcacaugauguuggccac.....................    1      0     seq
......................................................................aagcacaugGuguuggccacg....................    1      1     seq
......................................................................aagcacaugauguAggccacg....................    1      1     seq
......................................................................aagcacauAauguuggccacg....................    1      1     seq
......................................................................aagcacaugauguuggccacg....................    319    0     seq
......................................................................aagcacaugauguuggccacgc...................    1      0     seq
.......................................................................agcacaugauguuggccacg....................    1      0     seq
.......................................................................agcacaugauguuggccacgc...................    1      0     seq
.......................................................................agcacaugauguuggccacgccU.................    1      1     seq
.........................................................................cacaugauguuggccacg....................    1      0     seq
.........................................................................cacaugauguuggccacgccg.................    2      0     seq
```
