# Supplementary material for: The Small RNA Universe of Capitella teleta
Source: Front Mol Biosci. 2022 Feb 25;9:802814. doi: 10.3389/fmolb.2022.802814 (PMC8915122; doi:10.3389/fmolb.2022.802814)
Supplement: Supplementary file 1 [file DataSheet1.ZIP › Supplement/candidate/CAPTEscaffold_55_4683.pdf]

Provisional ID : CAPTEscaffold_55_4683
Score total : 364
Score for star read(s) : 3.9
Score for read counts : 356.5
Score for mfe : 2.6
Score for randfold : 1.6
Score for cons. seed : -0.6
Total read count : 711
Mature read count : 702
Loop read count : 0
Star read count : 9

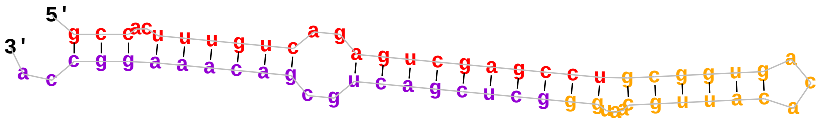

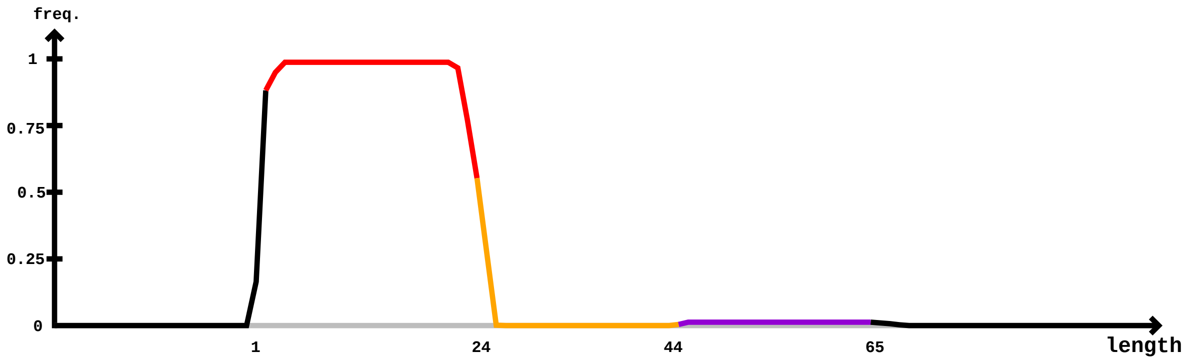


```
                                         Mature                                      Star
5'- aggaggaagcggaggcggccgccacuuugucagagucgagccugcggugacacauugcaauggggcucgacugcgacaaaggccaagccgccuccguagccaagccacugcugc   -3'   obs
    aggaggaagcggaggcggccgccacuuugucagagucgagccugcggugacacauugcaauggggcucgacugcgacaaaggccaagccgccuccguagccaagccacugcugc         exp
    ....((..(((((((((((.(((..((((((..((((((((((((((((...))))))...))))))))))..)))))))))...)))))))))))..)).(((....)))..   reads   mm   sample
    ...................cgccacuuugucagagucgag.........................................................................   1       0    seq
    ...................cgccacuuugucagagucgagc........................................................................   18      0    seq
    ...................cgccacuuugucagagucgagcc.......................................................................   45      0    seq
    ...................cgccacuuugucGgagucgagccu......................................................................   2       1    seq
    ...................cgccacuuugucagagucgagccA......................................................................   5       1    seq
    ...................cgcUacuuugucagagucgagccu......................................................................   1       1    seq
    ...................cgccacuuugucagagucgagccu......................................................................   43      0    seq
    ...................cgccacuuugucagagucgagccuU.....................................................................   2       1    seq
    ....................gccacuuugucagagucgag.........................................................................   14      0    seq
    ....................gccacuuugucagagucgagc........................................................................   118     0    seq
    ....................gccacuuugucagagucgagG........................................................................   1       1    seq
    ....................gccacuCugucagagucgagc........................................................................   1       1    seq
    ....................Cccacuuugucagagucgagc........................................................................   1       1    seq
    ....................gccacuuugucagagucgagcc.......................................................................   109     0    seq
    ....................gccacuuugucGgagucgagcc.......................................................................   1       1    seq
    ....................gccacuuugucGgagucgagccu......................................................................   7       1    seq
    ....................Uccacuuugucagagucgagccu......................................................................   1       1    seq
    ....................gccacuuugucagagucAagccu......................................................................   1       1    seq
    ....................gccacuuugucagagucgagccu......................................................................   245     0    seq
    ....................gccacuuugucagagucgagccA......................................................................   8       1    seq
    ....................gccacuuugucagagucgagccG......................................................................   2       1    seq
    ....................gccacuuugucagagucgagccugG....................................................................   1       1    seq
    .....................ccacuuugucagagucgagccu......................................................................   45      0    seq
    .....................ccacuuugucagagucgagccA......................................................................   1       1    seq
    .....................ccacAuugucagagucgagccu......................................................................   1       1    seq
    .....................ccacuuugucagagucAagccu......................................................................   1       1    seq
    ......................cacuAugucagagucgagccu......................................................................   1       1    seq
    ......................cacuuuAucagagucgagccu......................................................................   1       1    seq
    ......................cacuuugucagagucgagccu......................................................................   25      0    seq
    ...............................................................gcucgacugcgacaaaggccU.............................   2       1    seq
    ...............................................................gcucgacugcgacaaaggcca.............................   1       0    seq
    ................................................................cucgacugcgacaaaggcca.............................   1       0    seq
    ................................................................cucgacugcgacaaaggccaag...........................   3       0    seq
```

```
                    Mature                                    Star
aggaggaagcggaggcggccgccacuuugucagagucgagccugcggugacacauugcaauggcgcucgacugcgacaaaggccaagccgccuccguagccaagccacugcugc
................................................................cucgacugcgacaaaggccaagU..........................    2    1    seq
```
