# Supplementary material for: The Small RNA Universe of Capitella teleta
Source: Front Mol Biosci. 2022 Feb 25;9:802814. doi: 10.3389/fmolb.2022.802814 (PMC8915122; doi:10.3389/fmolb.2022.802814)
Supplement: Supplementary file 1 [file DataSheet1.ZIP › Supplement/candidate/CAPTEscaffold_60_5416.pdf]

Provisional ID	:	CAPTEscaffold_60_5416
Score total	:	107.8
Score for star read(s)	:	3.9
Score for read counts	:	102.1
Score for mfe	:	0.8
Score for randfold	:	1.6
Score for cons. seed	:	-0.6
Total read count	:	212
Mature read count	:	206
Loop read count	:	0
Star read count	:	6

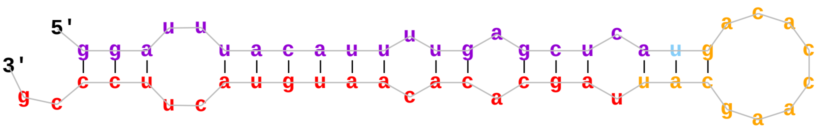

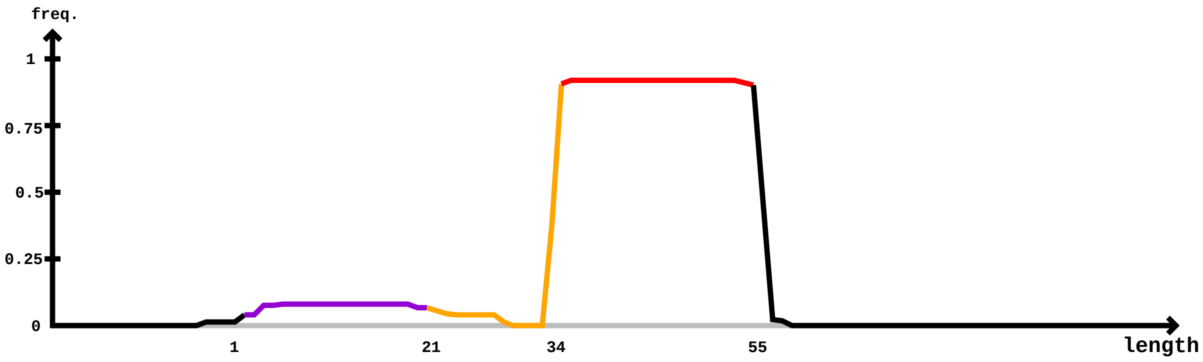

Star Mature

```
5'- ugcauagauuauauuuggggauuuacauuuugagcucaugacaccaagcauuagcacacaauguacuucccgaaauauaggcuucucgcuucgugauuauuuccacuguguacuu   -3'    obs
    ugcauagauuauauuuggggauuuacauuuugagcucaugacaccaagcauuagcacacaauguacuucccgaaauauaggcuucucgcuucgugauuauuuccacuguguacuu          exp
    (((((((..(((((((.((((..((((((.((.(((.(((........))).))).)).))))))..)))).)))))))(((.....)))..............)))))))...    reads      mm        sample
    ..............uuggggauuuaAauuuugagcu.......................................................................    3          1         seq
    ..................ggauuuacauuuugagcuca.............................................................................    3          0         seq
    ..................ggauuuacauuuugagcucau............................................................................    2          0         seq
    ..................ggauuuacauuuugagcucaug...........................................................................    1          0         seq
    ....................auuuacauuuugagcucaugacacc......................................................................    5          0         seq
    ....................auuuacauuuugagcucaugacacca.....................................................................    3          0         seq
    ......................uuacauuuugagcucaugacacc......................................................................    1          0         seq
    ..................................................uuagcacacaauguacuucc.............................................    1          0         seq
    ..................................................uuagcacacaauguacuuccc............................................    1          0         seq
    ..................................................uuagcacacaauguacuucccg...........................................    71         0         seq
    ..................................................uuagcacacaauguacuucAcg...........................................    1          1         seq
    ..................................................Auagcacacaauguacuucccg...........................................    1          1         seq
    ..................................................uuagcacacaauguacuucccU...........................................    5          1         seq
    ..................................................uuagcacacaauguacuucccga..........................................    3          0         seq
    ..................................................uuagcacUcaauguacuucccgaaa........................................    1          1         seq
    ...................................................uagcacacaauguacuucc.............................................    1          0         seq
    ...................................................uagcacacaauguacuuccc............................................    1          0         seq
    ...................................................uagcacacaauguacuucccg...........................................    94         0         seq
    ...................................................uagcacacaauguacuucccU...........................................    1          1         seq
    ...................................................uagcacacaauguacuucccgU..........................................    12         1         seq
    ...................................................uagcacacaauguacuucccga..........................................    6          0         seq
    ...................................................uagcacacaauguacuucccgaC.........................................    1          1         seq
    ...................................................uagcacacaauguacuucccgaaa........................................    3          0         seq
    ....................................................agcacacaauguacuucccga..........................................    1          0         seq
    ....................................................agcacacaauguacuucccgU..........................................    2          1         seq
```
