# Supplementary material for: The Small RNA Universe of Capitella teleta
Source: Front Mol Biosci. 2022 Feb 25;9:802814. doi: 10.3389/fmolb.2022.802814 (PMC8915122; doi:10.3389/fmolb.2022.802814)
Supplement: Supplementary file 1 [file DataSheet1.ZIP › Supplement/homologRecovered/CAPTEscaffold_933_30849.pdf]

Provisional ID	:	CAPTEscaffold_933_30849
Score total	:	611337.8
Score for star read(s)	:	3.9
Score for read counts	:	611327.4
Score for mfe	:	1.9
Score for randfold	:	1.6
Score for cons. seed	:	3
Total read count	:	1199104
Mature read count	:	1198824
Loop read count	:	0
Star read count	:	280

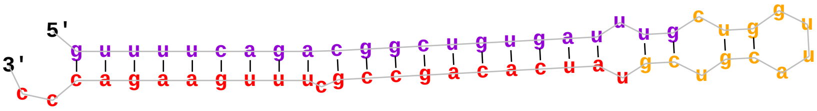

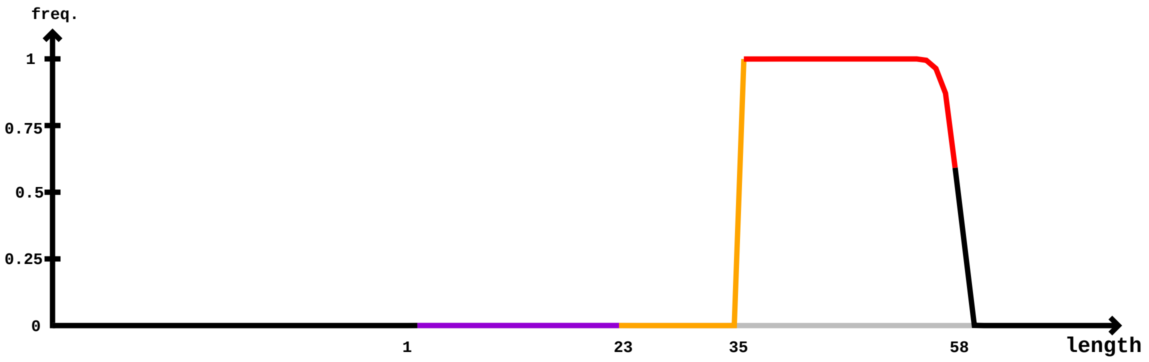

Star Mature

```
5'- ucgugguuauugcagugguuauauagcuuggcgcggguuuucagacggcugugauuugcugguuacgucguaucacagccgcuuugaagaccccugcuuugucuaacga  -3'  obs
    ucgugguuauugcagugguuauauagcuuggcgcggguuuucagacggcugugauuugcugguuacgucguaucacagccgcuuugaagaccccugcuuugucuaacga       exp
    ((((((.((..((((..((((.......))))..(((((((((((((((((((((.((.((....)).)).)).))))))))))).)))))))))))))))..)).)).))))  reads  mm  sample
    ................gguuauauagcuuggcgcgg.........................................................................  2  0  seq
    .................guuauauagcuuggcgcgg.........................................................................  18  0  seq
    ..................uuauauagcuuggcgcgg.........................................................................  12  0  seq
    .................................cggguuuucagacggcuguga.......................................................  2  0  seq
    ..................................ggguuuucagacggcuguga.......................................................  1  0  seq
    ....................................guuuucagacggcugugauu.....................................................  3  0  seq
    ....................................guuuucagacggcugugauuA....................................................  1  1  seq
    ....................................guuuucagacggcugugauuu....................................................  25  0  seq
    ....................................guuuucagacggcugugauuug...................................................  212  0  seq
    ....................................guuuucagacggcuAugauuug...................................................  1  1  seq
    ....................................gGuuucagacggcugugauuug...................................................  1  1  seq
    ....................................guuuucagaAggcugugauuug...................................................  1  1  seq
    ....................................guuuucagacggcugugauuugU..................................................  7  1  seq
    ....................................guuuucagacggcugugauuugcugguuac............................................  1  0  seq
    .....................................uuuucagacggcugugauuugcu.................................................  2  0  seq
    ......................................uuucagacggcugugauuugc..................................................  2  0  seq
    ......................................uuucagacggcugugauuugcu.................................................  16  0  seq
    .......................................uucagacgAcugugauuugc..................................................  1  1  seq
    .......................................uucagacggcugugauuugc..................................................  4  0  seq
    .......................................uucagacggcugugauuugcu.................................................  3  0  seq
    ..........................................................cugguuacgucguaucacagccgcuuuga......................  1  0  seq
    ...............................................................uacgucguaucacagccgcuuuga......................  1  0  seq
    ...............................................................uacgucguaucacagccgcuuugaagaccc................  2  0  seq
    .................................................................cguGguaucacagccgcuuugaagacc.................  3  1  seq
    .................................................................cguGguaucacagccgcuuugaagaccc................  1  1  seq
    ...................................................................uUguaucacagccgcuuuga......................  1  1  seq
    ...................................................................ucguauAacagccgcuuuga......................  1  1  seq
    ...................................................................ucguaucacagccgcuuuga......................  7  0  seq
    ...................................................................ucguaucacagccgcuuugaag....................  3  0  seq
    ...................................................................ucguaucacagccgcuuugaaga...................  1  0  seq
    ...................................................................ucguaucacagccgcuuugaagacc.................  3  0  seq
    ...................................................................ucguaucacagccgcuuugaagaccc................  2  0  seq
    ....................................................................Gguaucacagccgcuuugaagacc.................  1  1  seq
```

Star **Mature**

ucgugguuauugcagugguuauauagcuuggcgcggguuuucagacggcugugauuugcugguuacgucguaucacagccgcuuugaagaccccugcuuugucuaacga			
....Gguaucacagccgcuuugaagaccc................	12	1	seq
....Aguaucacagccgcuuugaagaccc................	1	1	seq
....Uuaucacagccgcuuugaaga...................	4	1	seq
....Cuaucacagccgcuuugaagac..................	13	1	seq
....Cuaucacagccgcuuugaagacc.................	6	1	seq
....Uuaucacagccgcuuugaagacc.................	5	1	seq
....Auaucacagccgcuuugaagacc.................	1	1	seq
....guaucacagccgcuuugaagaccc................	1	0	seq
....Cuaucacagccgcuuugaagaccc................	7	1	seq
....Uuaucacagccgcuuugaagaccc................	10	1	seq
....Auaucacagccgcuuugaagaccc................	2	1	seq
....uaucacagccgcuuugaa.....................	14	0	seq
....uaucacagccgcuuugaC.....................	1	1	seq
....uaucacagccgcuuugaaA....................	4	1	seq
....uaucacagccgcuuuUaag....................	2	1	seq
....uaucacagccgcCuugaag....................	1	1	seq
....uaucacagcAgcuuugaag....................	4	1	seq
....uaucacagccgcuuugaaU....................	2	1	seq
....uaucaAagccgcuuugaag....................	3	1	seq
....uaucGcagccgcuuugaag....................	3	1	seq
....uaucacaCccgcuuugaag....................	1	1	seq
....uaucacagccgcuuGgaag....................	1	1	seq
....uaucacagccgcuGugaag....................	1	1	seq
....uaAcacagccgcuuugaag....................	13	1	seq
....Aaucacagccgcuuugaag....................	26	1	seq
....uauUacagccgcuuugaag....................	1	1	seq
....uaucacaUccgcuuugaag....................	1	1	seq
....uaucacagccAcuuugaag....................	3	1	seq
....uaucacagccgcuuugaag....................	5916	0	seq
....uaucacagccgAuuugaag....................	1	1	seq
....Gaucacagccgcuuugaag....................	2	1	seq
....uGucacagccgcuuugaag....................	2	1	seq
....uaucacagccgcuCugaag....................	1	1	seq
....uaucaUagccgcuuugaag....................	1	1	seq
....uaucacagccgcAuugaag....................	1	1	seq
....uaucacagccgcuuuAaag....................	1	1	seq
....Naucacagccgcuuugaag....................	3	1	seq
....uaucacagccgcuuugaGg....................	1	1	seq
....uaucacagAcgcuuugaag....................	8	1	seq
....uaucacagccgUuuugaag....................	2	1	seq
....uaucacagccgcuuAgaag....................	2	1	seq
....uaucacagcUgcuuugaag....................	1	1	seq
....uaucacagccgcuuCgaag....................	1	1	seq
....uaucacagccgcuuugUaga...................	2	1	seq
....uNucacagccgcuuugaaga...................	3	1	seq
....uaucacagccUcuuugaaga...................	3	1	seq
....uaucacagccgcuCugaaga...................	3	1	seq
....uaucacagcUgcuuugaaga...................	17	1	seq
....uaucacagccgGuuugaaga...................	1	1	seq
....Aaucacagccgcuuugaaga...................	141	1	seq
....uaucUcagccgcuuugaaga...................	3	1	seq
....Caucacagccgcuuugaaga...................	4	1	seq
....uaucacagccgcuuugaaAa...................	2	1	seq
....uaucacagccgcCuugaaga...................	6	1	seq
....uaucGcagccgcuuugaaga...................	11	1	seq
....uaucacagcAgcuuugaaga...................	25	1	seq
....uaucacagccgcuAugaaga...................	16	1	seq
....uaucaUagccgcuuugaaga...................	5	1	seq
....Gaucacagccgcuuugaaga...................	12	1	seq
....uaucacagccgcuuugGaga...................	9	1	seq
....uGucacagccgcuuugaaga...................	3	1	seq
....uaucacagccCcuuugaaga...................	2	1	seq
....uaucacagAcgcuuugaaga...................	29	1	seq
....uauUacagccgcuuugaaga...................	9	1	seq
....uaucacGgccgcuuugaaga...................	7	1	seq
....uaucacagccgcuuugaUga...................	4	1	seq
....uaucaNagccgcuuugaaga...................	1	1	seq
....uauGacagccgcuuugaaga...................	3	1	seq
....uaucacaCccgcuuugaaga...................	8	1	seq
....uaucacagccgcuuuCaaga...................	5	1	seq

```
                                    Star                                Mature
ucgugguuauugcagugguuauauagcuuggcgcggguuuucagacggcugugauuugcugguuacgucguaucacagccgcuuugaagaccccugcuuugucuaacga
......................................................................uaucacaUccgcuuugaaga...................  5       1   seq
......................................................................uaucacagGcgcuuugaaga...................  3       1   seq
......................................................................uauAacagccgcuuugaaga...................  3       1   seq
......................................................................uaucacagccgcuuugaGga...................  6       1   seq
......................................................................uaCcacagccgcuuugaaga...................  1       1   seq
......................................................................Naucacagccgcuuugaaga...................  8       1   seq
......................................................................uaucacagccgcuuugaagU...................  8       1   seq
......................................................................uaucacagccgcuuugaaga...................  36381   0   seq
......................................................................uaucaAagccgcuuugaaga...................  16      1   seq
......................................................................uaucacagccgcuuuAaaga...................  18      1   seq
......................................................................uaucacagcGgcuuugaaga...................  6       1   seq
......................................................................uaucacagcNgcuuugaaga...................  1       1   seq
......................................................................uaucacagccgUuuugaaga...................  3       1   seq
......................................................................uaucacagccgAuuugaaga...................  10      1   seq
......................................................................uaucacagccgcuuugaagG...................  22      1   seq
......................................................................uaucacCgccgcuuugaaga...................  4       1   seq
......................................................................uaucacagccgcuuugaaUa...................  1       1   seq
......................................................................uaucacUgccgcuuugaaga...................  3       1   seq
......................................................................uaucacagccAcuuugaaga...................  1       1   seq
......................................................................uaucNcagccgcuuugaaga...................  1       1   seq
......................................................................uaucacagccgcuuAgaaga...................  13      1   seq
......................................................................uaAcacagccgcuuugaaga...................  42      1   seq
......................................................................uaGcacagccgcuuugaaga...................  1       1   seq
......................................................................uaucacagUcgcuuugaaga...................  36      1   seq
......................................................................uaucacagccgcGuugaaga...................  2       1   seq
......................................................................uaucCcagccgcuuugaaga...................  2       1   seq
......................................................................uUucacagccgcuuugaaga...................  4       1   seq
......................................................................uaucacagccgcAuugaaga...................  6       1   seq
......................................................................uaucacaAccgcuuugaaga...................  3       1   seq
......................................................................uaucacagccgcuuuUaaga...................  4       1   seq
......................................................................uaucacagccgcuuCgaaga...................  2       1   seq
......................................................................uaucacUgccgcuuugaagac..................  7       1   seq
......................................................................uaCcacagccgcuuugaagac..................  4       1   seq
......................................................................uaucUcagccgcuuugaagac..................  17      1   seq
......................................................................uaGcacagccgcuuugaagac..................  3       1   seq
......................................................................uaucacagccgcuuuAaagac..................  59      1   seq
......................................................................uaucacagccgcuuugaagUc..................  6       1   seq
......................................................................uaucacagccgcuuugaagac..................  109734  0   seq
......................................................................uUucacagccgcuuugaagac..................  12      1   seq
......................................................................uaucacagccUcuuugaagac..................  7       1   seq
......................................................................uaucacagccgcuuugaaNac..................  1       1   seq
......................................................................uaucacagccgcuuAgaagac..................  45      1   seq
......................................................................uaucacagccgcuuugaUgac..................  6       1   seq
......................................................................Caucacagccgcuuugaagac..................  12      1   seq
......................................................................uaucacagccgGuuugaagac..................  4       1   seq
......................................................................uaucacagcGgcuuugaagac..................  15      1   seq
......................................................................uaucacagccgcCuugaagac..................  18      1   seq
......................................................................uaucacagccgcuuugaaCac..................  3       1   seq
......................................................................uaucacaCccgcuuugaagac..................  13      1   seq
......................................................................uaucacagccgcuuugaagaN..................  1       1   seq
......................................................................uaucacagccgcuAugaagac..................  21      1   seq
......................................................................uaucacagccgcuuuGGagac..................  10      1   seq
......................................................................uaucCcagccgcuuugaagac..................  4       1   seq
......................................................................uaucacagGcgcuuugaagac..................  3       1   seq
......................................................................uaucacagccgcuuuNaagac..................  1       1   seq
......................................................................uaucaNagccgcuuugaagac..................  5       1   seq
......................................................................uaucacagccgcuuCgaagac..................  17      1   seq
......................................................................uaucaGagccgcuuugaagac..................  2       1   seq
......................................................................uaucacagccgcuCugaagac..................  17      1   seq
......................................................................uaucGcagccgcuuugaagac..................  31      1   seq
......................................................................uaucacagcUgcuuugaagac..................  44      1   seq
......................................................................uaucacagccgcuuuUaagac..................  12      1   seq
......................................................................uaucacagccAcuuugaagac..................  15      1   seq
......................................................................uaucacagccgcuuugaagaU..................  11      1   seq
......................................................................uaucacCgccgcuuugaagac..................  7       1   seq
......................................................................uaucacagccgcGuugaagac..................  5       1   seq
......................................................................uNucacagccgcuuugaagac..................  6       1   seq
......................................................................uaucacagccgcuuugaaUac..................  5       1   seq
......................................................................uaucacagccgcuuuGCagac..................  2       1   seq
......................................................................uaucacaUccgcuuugaagac..................  14      1   seq
```

```
                                    Star                              Mature
ucgugguuauugcagugguuauauagcuuggcgcggguuuucagacggcugugauuugcugguuacgucguaucacagccgcuuugaagaccccugcuuugucuaacga
......................................................................uaucacagccgcuuugaGgac..................  17      1   seq
......................................................................uaucacagcAgcuuugaagac..................  68      1   seq
......................................................................uauGacagccgcuuugaagac..................  8       1   seq
......................................................................uaucacagccgcuuugaagGc..................  12      1   seq
......................................................................uaucacagccgcAuugaagac..................  14      1   seq
......................................................................uaucacagccgcuGugaagac..................  1       1   seq
......................................................................uauUacagccgcuuugaagac..................  21      1   seq
......................................................................uaucacagccCcuuugaagac..................  5       1   seq
......................................................................uaucacGgccgcuuugaagac..................  23      1   seq
......................................................................uaucacaAccgcuuugaagac..................  10      1   seq
......................................................................uGucacagccgcuuugaagac..................  11      1   seq
......................................................................uaucacagccgcuuugaagaA..................  47      1   seq
......................................................................uauAacagccgcuuugaagac..................  23      1   seq
......................................................................uaucaAagccgcuuugaagac..................  69      1   seq
......................................................................uaucaUagccgcuuugaagac..................  10      1   seq
......................................................................uaucacagccgcuuugaaAac..................  2       1   seq
......................................................................uaucacagUcgcuuugaagac..................  34      1   seq
......................................................................Naucacagccgcuuugaagac..................  25      1   seq
......................................................................uaucacagccgUuuugaagac..................  11      1   seq
......................................................................uaucacagccgcuuuCaagac..................  12      1   seq
......................................................................uaucacagccgAuuugaagac..................  24      1   seq
......................................................................uaucacagcNgcuuugaagac..................  2       1   seq
......................................................................uaucacagccgcuuugUagac..................  6       1   seq
......................................................................Aaucacagccgcuuugaagac..................  504     1   seq
......................................................................uaAcacagccgcuuugaagac..................  128     1   seq
......................................................................uaucacagccgcuuugaagaG..................  2       1   seq
......................................................................uaucacagAcgcuuugaagac..................  52      1   seq
......................................................................Gaucacagccgcuuugaagac..................  45      1   seq
......................................................................uaGcacagccgcuuugaagacc.................  3       1   seq
......................................................................uaucaAagccgcuuugaagacc.................  143     1   seq
......................................................................uaucacagGcgcuuugaagacc.................  12      1   seq
......................................................................uaucacagccgcuuugaagaGc.................  14      1   seq
......................................................................uaucacagccUcuuugaagacc.................  31      1   seq
......................................................................uGucacagccgcuuugaagacc.................  40      1   seq
......................................................................uaucacagccgcuCugaagacc.................  36      1   seq
......................................................................uaucacagccgcuuugaagacA.................  381     1   seq
......................................................................uNucacagccgcuuugaagacc.................  15      1   seq
......................................................................uaNcacagccgcuuugaagacc.................  1       1   seq
......................................................................uaucacaCccgcuuugaagacc.................  32      1   seq
......................................................................uaucacagccgcCuugaagacc.................  238     1   seq
......................................................................uaucacCgccgcuuugaagacc.................  8       1   seq
......................................................................uaucacagccCcuuugaagacc.................  6       1   seq
......................................................................uaCcacagccgcuuugaagacc.................  111     1   seq
......................................................................uaucacagccgcuuugaaNacc.................  1       1   seq
......................................................................uauAacagccgcuuugaagacc.................  72      1   seq
......................................................................uaucacagccgcuuugaaCacc.................  14      1   seq
......................................................................uaucacagccgcuuGgaagacc.................  6       1   seq
......................................................................Aaucacagccgcuuugaagacc.................  1227    1   seq
......................................................................uaucacagccgcuGugaagacc.................  6       1   seq
......................................................................uaucacagccgcuuugaagaNc.................  2       1   seq
......................................................................uaucacagccgcuuugaaUacc.................  19      1   seq
......................................................................uaucUcagccgcuuugaagacc.................  35      1   seq
......................................................................uaucacagccgcuuugaagaUc.................  29      1   seq
......................................................................uaucacagccgcuuuCaagacc.................  33      1   seq
......................................................................uaucacagccgcuuugGagacc.................  53      1   seq
......................................................................uaucacagccgcAuugaagacc.................  42      1   seq
......................................................................uaucacagcNgcuuugaagacc.................  1       1   seq
......................................................................uauUacagccgcuuugaagacc.................  74      1   seq
......................................................................uaucacagcUgcuuugaagacc.................  202     1   seq
......................................................................uaucacagccgcuuugaagacU.................  553     1   seq
......................................................................uaucacagccgcuuugaagacN.................  1       1   seq
......................................................................uaucacagccgcuuugaCgacc.................  4       1   seq
......................................................................uaucacagccgcuuugUagacc.................  33      1   seq
......................................................................uaucacagccgcuuugaagacc.................  329315  0   seq
......................................................................uaucacagccgcuuugaaAacc.................  25      1   seq
......................................................................uaucacagcGgcuuugaagacc.................  25      1   seq
......................................................................uaucacUgccgcuuugaagacc.................  16      1   seq
......................................................................uaucacagccgcuNugaagacc.................  1       1   seq
......................................................................uaucaGagccgcuuugaagacc.................  10      1   seq
......................................................................uaucacagccgcuAugaagacc.................  63      1   seq
```

```
                                    Star                      Mature
ucgugguuauugcagugguuauauagcuuggcgcggguuuucagacggcugugauuugcugguuacgucguaucacagccgcuuugaagaccccugcuuugucuaacga
......................................................................uaucacagccgcuuugaagacG.................	28	1	seq
......................................................................uaucacagccgcuuCgaagacc.................	51	1	seq
......................................................................uUucacagccgcuuugaagacc.................	29	1	seq
......................................................................uaucacagccgcuuugaagUcc.................	43	1	seq
......................................................................uaucacagccgcuuugaagCcc.................	13	1	seq
......................................................................uaucacaAccgcuuugaagacc.................	33	1	seq
......................................................................uaAcacagccgcuuugaagacc.................	306	1	seq
......................................................................uaucacaUccgcuuugaagacc.................	20	1	seq
......................................................................uaucacagccgcuuAgaagacc.................	154	1	seq
......................................................................uaucacNgccgcuuugaagacc.................	1	1	seq
......................................................................uaucacagUcgcuuugaagacc.................	59	1	seq
......................................................................uaucacagccgcGuugaagacc.................	18	1	seq
......................................................................uaucacagccgcuuugCagacc.................	3	1	seq
......................................................................uaucacagccgGuuugaagacc.................	7	1	seq
......................................................................uaucacagccAcuuugaagacc.................	40	1	seq
......................................................................uaucaNagccgcuuugaagacc.................	12	1	seq
......................................................................uaucacagccgcuuugaUgacc.................	6	1	seq
......................................................................uaucacGgccgcuuugaagacc.................	89	1	seq
......................................................................uaucacagcAgcuuugaagacc.................	203	1	seq
......................................................................uauGacagccgcuuugaagacc.................	9	1	seq
......................................................................uaucacagAcgcuuugaagacc.................	117	1	seq
......................................................................Naucacagccgcuuugaagacc.................	97	1	seq
......................................................................uaucacagccgcuuugaagGcc.................	43	1	seq
......................................................................uaucacagccgcuuugaagaAc.................	52	1	seq
......................................................................uaucacagccgcuuuUaagacc.................	26	1	seq
......................................................................uaucacagccgAuuugaagacc.................	90	1	seq
......................................................................uaucCcagccgcuuugaagacc.................	13	1	seq
......................................................................uaucacagccgcuuuAaagacc.................	211	1	seq
......................................................................Gaucacagccgcuuugaagacc.................	110	1	seq
......................................................................uaucacagccgUuuugaagacc.................	94	1	seq
......................................................................uaucacagccgcuuugaGgacc.................	78	1	seq
......................................................................uaucaUagccgcuuugaagacc.................	35	1	seq
......................................................................Caucacagccgcuuugaagacc.................	39	1	seq
......................................................................uaucGcagccgcuuugaagacc.................	69	1	seq
......................................................................uaAcacagccgcuuugaagaccc................	445	1	seq
......................................................................uaucUcagccgcuuugaagaccc................	70	1	seq
......................................................................uaucacagccgcuuugNagaccc................	2	1	seq
......................................................................uaucacaNccgcuuugaagaccc................	1	1	seq
......................................................................uaucGcagccgcuuugaagaccc................	129	1	seq
......................................................................uaucacagccgcuuuUaagaccc................	47	1	seq
......................................................................uaucacagccgcuuugaaUaccc................	110	1	seq
......................................................................uaucacagccgcuuGgaagaccc................	11	1	seq
......................................................................uaucacagccgcuuugaagUccc................	75	1	seq
......................................................................uaucacCgccgcuuugaagaccc................	18	1	seq
......................................................................uaucacagccgcuuugaaAaccc................	52	1	seq
......................................................................uaucacagccgcuuugaagaccG................	123	1	seq
......................................................................uaucacagcNgcuuugaagaccc................	4	1	seq
......................................................................uaucacagccUcuuugaagaccc................	40	1	seq
......................................................................uaucaNagccgcuuugaagaccc................	19	1	seq
......................................................................Naucacagccgcuuugaagaccc................	155	1	seq
......................................................................uaucacagccgcuuugaaCaccc................	12	1	seq
......................................................................uaucacagccgcuuugaagacAc................	1415	1	seq
......................................................................uGucacagccgcuuugaagaccc................	74	1	seq
......................................................................uaucCcagccgcuuugaagaccc................	22	1	seq
......................................................................uaucacagccgcuuugaagaccU................	5815	1	seq
......................................................................uaucacagccgcuuugaUgaccc................	43	1	seq
......................................................................uauAacagccgcuuugaagaccc................	118	1	seq
......................................................................uaucacagccgNuuugaagaccc................	1	1	seq
......................................................................uaucacagccgcuuugaagGccc................	70	1	seq
......................................................................Gaucacagccgcuuugaagaccc................	262	1	seq
......................................................................uaucacagccgcuuugGagaccc................	86	1	seq
......................................................................uaucaAagccgcuuugaagaccc................	189	1	seq
......................................................................uaucacagccgAuuugaagaccc................	140	1	seq
......................................................................uaucacagccgGuuugaagaccc................	12	1	seq
......................................................................uaucacagcUgcuuugaagaccc................	299	1	seq
......................................................................uaucacagccgUuuugaagaccc................	39	1	seq
......................................................................uaucacagccgcGuugaagaccc................	38	1	seq
......................................................................uaucacagccgcuuugaagaNcc................	7	1	seq
......................................................................uaucacagccgcuuugaagaccN................	10	1	seq
......................................................................uUucacagccgcuuugaagaccc................	56	1	seq
```

Star **Mature**

```
ucgugguuauugcagugguuauauagcuuggcgcggguuuucagacggcugugauuugcugguuacgucguaucacagccgcuuugaagaccccugcuuugucuaacga
......................................................................uaucacagccgcuCugaagaccc................  76      1  seq
......................................................................uaucacagccgcuuugUagaccc................  54      1  seq
......................................................................uaucacagccgcuuugaaNaccc................  1       1  seq
......................................................................uaucaUagccgcuuugaagaccc................  88      1  seq
......................................................................Aaucacagccgcuuugaagaccc................  1874    1  seq
......................................................................uaucacagccgcuuugaCgaccc................  3       1  seq
......................................................................uaucacagAcgcuuugaagaccc................  171     1  seq
......................................................................uaucacagGcgcuuugaagaccc................  34      1  seq
......................................................................uaucacagccgcuuugCagaccc................  1       1  seq
......................................................................uaucacagccgcuuuCaagaccc................  55      1  seq
......................................................................uaucacagccgcuuCgaagaccc................  86      1  seq
......................................................................uaucacagccgcuuugaagaccA................  675     1  seq
......................................................................uaucacaCccgcuuugaagaccc................  61      1  seq
......................................................................uaucacUgccgcuuugaagaccc................  33      1  seq
......................................................................uaucaGagccgcuuugaagaccc................  21      1  seq
......................................................................uauGacagccgcuuugaagaccc................  21      1  seq
......................................................................uaucacagccgcCuugaagaccc................  282     1  seq
......................................................................uaCcacagccgcuuugaagaccc................  44      1  seq
......................................................................uaucacagccgcuuugaagaAcc................  131     1  seq
......................................................................uaucacGgccgcuuugaagaccc................  122     1  seq
......................................................................uaNcacagccgcuuugaagaccc................  3       1  seq
......................................................................uaucacagccgcAuugaagaccc................  66      1  seq
......................................................................uaucacagccgcuAugaagaccc................  107     1  seq
......................................................................uauUacagccgcuuugaagaccc................  123     1  seq
......................................................................uaucacagccAcuuugaagaccc................  74      1  seq
......................................................................uaucacagccgcuuugaagaGcc................  27      1  seq
......................................................................uaucacagcGgcuuugaagaccc................  46      1  seq
......................................................................uaucacaUccgcuuugaagaccc................  41      1  seq
......................................................................uaucacagccgcuuugaagaccc................  557967  0  seq
......................................................................uaGcacagccgcuuugaagaccc................  7       1  seq
......................................................................uaucacagccCcuuugaagaccc................  13      1  seq
......................................................................uaucacagccgcuuAgaagaccc................  241     1  seq
......................................................................uNucacagccgcuuugaagaccc................  18      1  seq
......................................................................uaucacagccgcuuugaGgaccc................  117     1  seq
......................................................................uaucacagccgcuuugaagacUc................  198     1  seq
......................................................................uaucacagcAgcuuugaagaccc................  306     1  seq
......................................................................uaucacagccgcuuuAaagaccc................  384     1  seq
......................................................................Caucacagccgcuuugaagaccc................  71      1  seq
......................................................................uaucacagccgcuuugaagacGc................  26      1  seq
......................................................................uaucacaAccgcuuugaagaccc................  63      1  seq
......................................................................uaucacagccgcuGugaagaccc................  12      1  seq
......................................................................uaucacagccgcuuugaagCccc................  52      1  seq
......................................................................uCucacagccgcuuugaagaccc................  1       1  seq
......................................................................uaucacagUcgcuuugaagaccc................  52      1  seq
......................................................................uaucacagccgcuuugaagaUcc................  134     1  seq
......................................................................uaucacagccgcuuugaagacccG...............  1129    1  seq
......................................................................uaucacagccgcuuugaagacccU...............  130766  1  seq
......................................................................uaucacagccgcuuugaagacccA...............  1823    1  seq
......................................................................uauUacagccgcuuugaagacccc...............  1       1  seq
......................................................................Naucacagccgcuuugaagacccc...............  1       1  seq
......................................................................uaucacagccgcuuugaagacccc...............  338     0  seq
......................................................................uaucacagccgcuuugaagaccUc...............  1       1  seq
......................................................................uaucacagccgcuuugaagaccccA..............  3       1  seq
......................................................................Gaucacagccgcuuugaagaccccu..............  1       1  seq
......................................................................uaucacagccgcuuugaagacccAu..............  152     1  seq
......................................................................uaucacagccgcuuugaagacccUu..............  628     1  seq
......................................................................uaucacagcAgcuuugaagaccccu..............  1       1  seq
......................................................................uaucacagccgcuuugaagaccccu..............  78      0  seq
......................................................................uaucacagccgcuuugaagaccccuU.............  2       1  seq
.......................................................................aucacagccgcuuugaagac..................  4       0  seq
.......................................................................Uucacagccgcuuugaagac..................  1       1  seq
.......................................................................aucacagccgcuuugaagacc.................  29      0  seq
.......................................................................aCcacagccgcuuugaagaccc................  1       1  seq
.......................................................................aucacagccgcuuugaagaccc................  13      0  seq
.......................................................................aucacagccgcuuugaagacccU...............  12      1  seq
........................................................................ucacagccgcuuugaagacc.................  17      0  seq
........................................................................Ncacagccgcuuugaagacc.................  1       1  seq
........................................................................ucacagccgcuuugaagaccc................  4       0  seq
........................................................................ucacagccgcuuugaagacccU...............  10      1  seq
.........................................................................cacagccgcuuugaagac..................  12      0  seq
```

Star **Mature**

```
ucgugguuauugcagugguuauauagcuuggcgcggguuuucagacggcugugauuugcugguuacgucguaucacagccgcuuugaagaccccugcuuugucuaacga
.........................................................................cacagccgcuuugaagacc.................     1     0     seq
.........................................................................cacagccgcuuugaagaccc................     8     0     seq
.........................................................................cacagccgcuuugaagacAc................     1     1     seq
.........................................................................cacagccgcuuugaagacccU...............     1     1     seq
.........................................................................cacagccgcuuugaagaccccu..............     84    0     seq
.........................................................................cacagccgcuuugaagaccccug.............     3     0     seq
..........................................................................acagccgcuuugaagacc.................     93    0     seq
..........................................................................acagccgcuuugaagaccc................     28    0     seq
..........................................................................acagccgcuuugaagacccU...............     10    1     seq
..........................................................................acagccgcuuugaagaccccu..............     4     0     seq
..........................................................................acagccgcuuugaagaccccug.............     3     0     seq
...........................................................................cagccgcuuugaagaccc................     15    0     seq
...........................................................................cagccgcuuugaagacccU...............     1     1     seq
```
