# Supplementary material for: The Small RNA Universe of Capitella teleta
Source: Front Mol Biosci. 2022 Feb 25;9:802814. doi: 10.3389/fmolb.2022.802814 (PMC8915122; doi:10.3389/fmolb.2022.802814)
Supplement: Supplementary file 1 [file DataSheet1.ZIP › Supplement/homologRecovered/CAPTEscaffold_522_23009.pdf]

Provisional ID	:	CAPTEscaffold_522_23009
Score total	:	45543.9
Score for star read(s)	:	3.9
Score for read counts	:	45533.1
Score for mfe	:	2.3
Score for randfold	:	1.6
Score for cons. seed	:	3
Total read count	:	89323
Mature read count	:	89315
Loop read count	:	0
Star read count	:	8

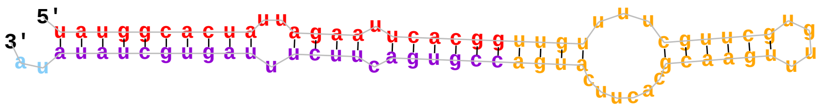

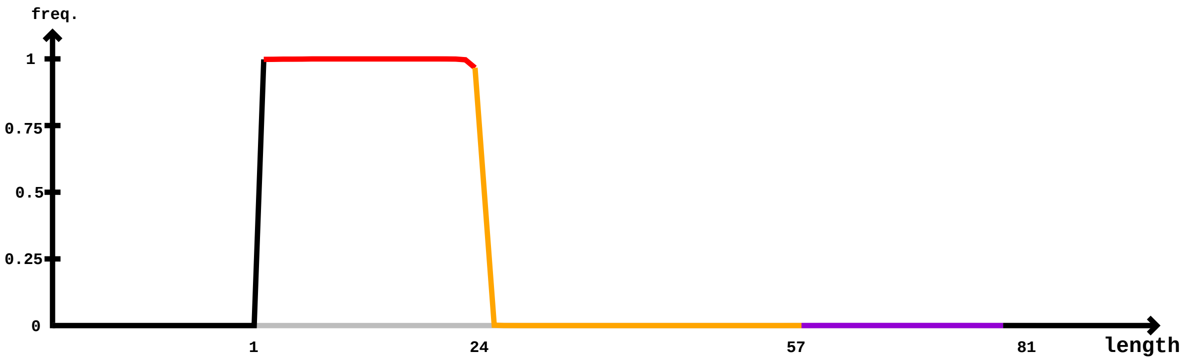

Mature **Star**

```
5'- acgcuuuguuucucgccaucuauggcacuauuagaauucacgguuguuuucguucguguuugaacgcacuucaugaccgugacuucuuuagugcuauauauggcgagaacgcu   -3'    obs
    acgcuuuguuucucgccaucuauggcacuauuagaauucacgguuguuuucguucguguuugaacgcacuucaugaccgugacuucuuuagugcuauauauggcgagaacgcu          exp
    .......((((((((((((.((((((((((..((((.((((((((((...((((((....))))))......)))))))))).)))).)))))))))).)))))))))).)).    reads    mm    sample
    .cgcuuuguuucucAccauc.............................................................................................    1        1     seq
    ...................cuauggcacuauuagaauucacgg......................................................................    1        0     seq
    ....................uauggcacuauuagaauu...........................................................................    6        0     seq
    ....................uauggcacuauuagaauuc..........................................................................    9        0     seq
    ....................Aauggcacuauuagaauuca.........................................................................    1        1     seq
    ....................uauggcacuauuagaauuca.........................................................................    29       0     seq
    ....................uauggcacuauuagaauucaA........................................................................    1        1     seq
    ....................uGuggcacuauuagaauucac........................................................................    3        1     seq
    ....................uauggcGcuauuagaauucac........................................................................    1        1     seq
    ....................uauggcacuGuuagaauucac........................................................................    1        1     seq
    ....................uauggcacuauuagaauucac........................................................................    233      0     seq
    ....................uauggcacuauuagaaCucac........................................................................    1        1     seq
    ....................Aauggcacuauuagaauucac........................................................................    2        1     seq
    ....................uauggcacuauuaAaauucacg.......................................................................    1        1     seq
    ....................uauggcacuauuagaauucaUg.......................................................................    1        1     seq
    ....................uauggcacuauuagaaCucacg.......................................................................    1        1     seq
    ....................uauggcacuauCagaauucacg.......................................................................    2        1     seq
    ....................uauggcacuauuagaauucacg.......................................................................    2627     0     seq
    ....................uauggcacuGuuagaauucacg.......................................................................    7        1     seq
    ....................uauggcacuauuCgaauucacg.......................................................................    1        1     seq
    ....................uauggcacuauuUgaauucacg.......................................................................    1        1     seq
    ....................uGuggcacuauuagaauucacg.......................................................................    12       1     seq
    ....................uauggcacuauuagaauuAacg.......................................................................    2        1     seq
    ....................uauggcacuauuagaauucaAg.......................................................................    3        1     seq
    ....................uauggcacuauAagaauucacg.......................................................................    1        1     seq
    ....................uauggcacuauuGgaauucacg.......................................................................    1        1     seq
    ....................uauggcacuauuagaauucacA.......................................................................    1        1     seq
    ....................uauggcacuauuagGauucacg.......................................................................    2        1     seq
    ....................uauggcacCauuagaauucacg.......................................................................    1        1     seq
    ....................uUuggcacuauuagaauucacg.......................................................................    1        1     seq
    ....................uauAgcacuauuagaauucacg.......................................................................    2        1     seq
    ....................uauggcacuauuagaauucaGg.......................................................................    1        1     seq
    ....................uauggcacGauuagaauucacg.......................................................................    1        1     seq
```

Mature Star

```
acgcuuuguuucucgccaucuauggcacuauuagaauucacgguuguuuucguucguguuugaacgcacuucaugaccgugacuucuuuagugcuauauauggcgagaacgcu
....................uaAggcacuauuagaauucacg.......................................................................   2      1   seq
....................uaugUcacuauuagaauucacg.......................................................................   1      1   seq
....................Aauggcacuauuagaauucacg.......................................................................   8      1   seq
....................uauggGacuauuagaauucacg.......................................................................   1      1   seq
....................uauggcacuauuagaauGcacg.......................................................................   1      1   seq
....................uauggcGcuauuagaauucacg.......................................................................   1      1   seq
....................uauggcaGuauuagaauucacgg......................................................................   3      1   seq
....................uauggcacuauuagaGuucacgg......................................................................   12     1   seq
....................uauggcacuauuagaauucaGgg......................................................................   16     1   seq
....................Cauggcacuauuagaauucacgg......................................................................   10     1   seq
....................uauggcacuaGuagaauucacgg......................................................................   1      1   seq
....................uauggcaAuauuagaauucacgg......................................................................   12     1   seq
....................uauggcCcuauuagaauucacgg......................................................................   1      1   seq
....................uauggcacuauAagaauucacgg......................................................................   22     1   seq
....................Aauggcacuauuagaauucacgg......................................................................   188    1   seq
....................uauggcacuauuagaauucaUgg......................................................................   23     1   seq
....................uaugUcacuauuagaauucacgg......................................................................   9      1   seq
....................Gauggcacuauuagaauucacgg......................................................................   17     1   seq
....................uauggcacuauuagaauucacAg......................................................................   181    1   seq
....................uUuggcacuauuagaauucacgg......................................................................   6      1   seq
....................uauggcacuauuagaUuucacgg......................................................................   10     1   seq
....................uauggcacuaCuagaauucacgg......................................................................   5      1   seq
....................uauggAacuauuagaauucacgg......................................................................   19     1   seq
....................uauggcacuauuagGauucacgg......................................................................   11     1   seq
....................uauggcacuauuagaauuCUcgg......................................................................   6      1   seq
....................uauggcacAauuagaauucacgg......................................................................   5      1   seq
....................uauggcacGauuagaauucacgg......................................................................   4      1   seq
....................uauggcGcuauuagaauucacgg......................................................................   27     1   seq
....................uGuggcacuauuagaauucacgg......................................................................   347    1   seq
....................uauggcacuauuagUauucacgg......................................................................   1      1   seq
....................uauggcacuauuagaauucacCg......................................................................   9      1   seq
....................uauggcacuauuagaauucacgg......................................................................   82483  0   seq
....................uauggcacuauuagaauucacgA......................................................................   38     1   seq
....................uauggcacuauuagaauuAacgg......................................................................   36     1   seq
....................uauggcacuauuagaauucacUg......................................................................   17     1   seq
....................uNuggcacuauuagaauucacgg......................................................................   4      1   seq
....................uauggcacuauuagaauGacgg.......................................................................   11     1   seq
....................uauggcacuauuagaauucGcgg......................................................................   76     1   seq
....................uauggcacCauuagaauucacgg......................................................................   6      1   seq
....................uauggcacuauuagaauucacgC......................................................................   20     1   seq
....................uaugAcacuauuagaauucacgg......................................................................   18     1   seq
....................uauggcacuauuagaauucaAgg......................................................................   26     1   seq
....................uauggcacuauuagaauucCcgg......................................................................   2      1   seq
....................Nauggcacuauuagaauucacgg......................................................................   31     1   seq
....................uaNggcacuauuagaauucacgg......................................................................   1      1   seq
....................uauggUacuauuagaauucacgg......................................................................   25     1   seq
....................uauggcacuauuagaauucacgU......................................................................   7      1   seq
....................uauCgcacuauuagaauucacgg......................................................................   5      1   seq
....................uaugCcacuauuagaauucacgg......................................................................   2      1   seq
....................uauAgcacuauuagaauucacgg......................................................................   85     1   seq
....................uauggcacuauuaUaauucacgg......................................................................   4      1   seq
....................uauggcacuauuagaaCucacgg......................................................................   7      1   seq
....................uauggcacuauuaCaauucacgg......................................................................   3      1   seq
....................uauggcacuauuUgaauucacgg......................................................................   6      1   seq
....................uaAggcacuauuagaauucacgg......................................................................   58     1   seq
....................uauggcacuGuuagaauucacgg......................................................................   133    1   seq
....................uauggGacuauuagaauucacgg......................................................................   11     1   seq
....................uauggcUcuauuagaauucacgg......................................................................   7      1   seq
....................uaCggcacuauuagaauucacgg......................................................................   5      1   seq
....................uauggcacuauCagaauucacgg......................................................................   13     1   seq
....................uauggcaUuauuagaauucacgg......................................................................   12     1   seq
....................uauggcacuauuaAaauucacgg......................................................................   37     1   seq
....................uauggcacuauuagaauAcacgg......................................................................   24     1   seq
....................uauggcacuauuagaauGcacgg......................................................................   9      1   seq
....................uauUgcacuauuagaauucacgg......................................................................   9      1   seq
....................uauggcacuauuCgaauucacgg......................................................................   2      1   seq
....................uauggcacuauuGgaauucacgg......................................................................   22     1   seq
....................uauggcacuauuagaaGucacgg......................................................................   8      1   seq
....................uauggcacuauuagaauCcacgg......................................................................   7      1   seq
....................uauggcacuauuagaaAucacgg......................................................................   25     1   seq
```

```
                    Mature                                                 Star
acgcuuuguuucucgccaucuauggcacuauuagaauucacgguuguuuucguucguguuugaacgcacuucaugaccgugacuucuuuagugcuauauauggcgagaacgcu
....................uauggcacuUuuagaauucacgg......................................................................    9      1    seq
....................uauggcacuaAuagaauucacgg......................................................................    33     1    seq
....................uauggcacuauuagaauuUacgg......................................................................    62     1    seq
....................uaugAcacuauuagaauucacggu.....................................................................    1      1    seq
....................uauggUacuauuagaauucacggu.....................................................................    1      1    seq
....................uauggcacuauuagaauucacggG.....................................................................    13     1    seq
....................uauggcacuauuagaauucacggC.....................................................................    1      1    seq
....................uauggcacuauuagaauucacggu.....................................................................    396    0    seq
....................uauggcacuauuagaauucacggN.....................................................................    1      1    seq
....................uauggcacuauuagaauucacggA.....................................................................    1355   1    seq
....................uauggcacuauCagaauucacggu.....................................................................    1      1    seq
....................uGuggcacuauuagaauucacggu.....................................................................    1      1    seq
....................uauggcacuauuGgaauucacggu.....................................................................    1      1    seq
....................Nauggcacuauuagaauucacggu.....................................................................    2      1    seq
....................Aauggcacuauuagaauucacggu.....................................................................    2      1    seq
....................uauggcacuauuagaauucacgguA....................................................................    38     1    seq
....................uauggcacuauuagaauucacgguu....................................................................    20     0    seq
....................uauggcacuauuagaauucacggAu....................................................................    6      1    seq
....................uauggcacuauuagaauucacgguuU...................................................................    6      1    seq
....................uauggcacuauuagaauucacgguGg...................................................................    1      1    seq
....................uauggcacuauuagaauucacgguuguuu................................................................    1      0    seq
....................uauggcacuauuagaauucacgguuguuuu...............................................................    2      0    seq
....................uauggcacuauuagaauucacgguuguuuuc..............................................................    1      0    seq
.....................auggcacuauuagaauucac........................................................................    1      0    seq
.....................auggcacuauuagaauucacgg......................................................................    4      0    seq
.....................Uuggcacuauuagaauucacgg......................................................................    1      1    seq
.....................auggcacuauuagaauucacggu.....................................................................    17     0    seq
.....................Guggcacuauuagaauucacggu.....................................................................    1      1    seq
.....................auggcacuauuagaauucacgguA....................................................................    1      1    seq
......................uggcacuauuagaauucacgg......................................................................    12     0    seq
......................uCgcacuauuagaauucacgg......................................................................    1      1    seq
......................uggcacuauuagaauucacggu.....................................................................    2      0    seq
......................uggcacuauuagaauucacgguu....................................................................    36     0    seq
......................uggcacuauuagaauucacgguuA...................................................................    3      1    seq
......................uggcacuauuagaauucacgguuAu..................................................................    1      1    seq
........................gcacuauuagaauucacgg......................................................................    15     0    seq
........................gcacuaGuagaauucacgg......................................................................    2      1    seq
.........................cacuauuagaauucacgg......................................................................    49     0    seq
.........................caAuauuagaauucacgg......................................................................    1      1    seq
.........................cacuauuagaauucacggu.....................................................................    3      0    seq
.........................cacuauuagaauucacggA.....................................................................    1      1    seq
.........................cacuauuagaauucacgguu....................................................................    2      0    seq
..........................acuauuagaauucacggA.....................................................................    1      1    seq
........................................................................augaccgugacuucuuuagugcuauau..............    1      0    seq
.........................................................................ugaccgugacuucuuuagugcu..................    2      0    seq
...........................................................................accgugacuucuuuagugcua.................    1      0    seq
...........................................................................accgugacuucuuuagugcuau................    1      0    seq
...........................................................................accgugacuucuuuagugcuauU...............    1      1    seq
...........................................................................accgugacuucuuuagugcuauau..............    1      0    seq
............................................................................ccgugacuucuuuagugcuaua...............    2      0    seq
.............................................................................cgugacuucuuuagugcuaua...............    2      0    seq
.............................................................................cgugacuucuuuagugcuauaua.............    2      0    seq
..............................................................................gugacuucuuuagugcuauaua.............    1      0    seq
...............................................................................ugacuucuuuagugcuauau..............    1      0    seq
```
