# Supplementary material for: The Small RNA Universe of Capitella teleta
Source: Front Mol Biosci. 2022 Feb 25;9:802814. doi: 10.3389/fmolb.2022.802814 (PMC8915122; doi:10.3389/fmolb.2022.802814)
Supplement: Supplementary file 1 [file DataSheet1.ZIP › Supplement/homologRecovered/CAPTEscaffold_12_1274.pdf]

Provisional ID	:	CAPTEscaffold_12_1274
Score total	:	1023.3
Score for star read(s)	:	3.9
Score for read counts	:	1013.1
Score for mfe	:	1.7
Score for randfold	:	1.6
Score for cons. seed	:	3
Total read count	:	1999
Mature read count	:	1732
Loop read count	:	113
Star read count	:	154

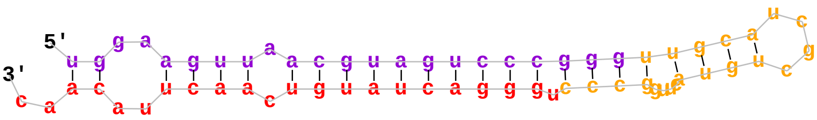

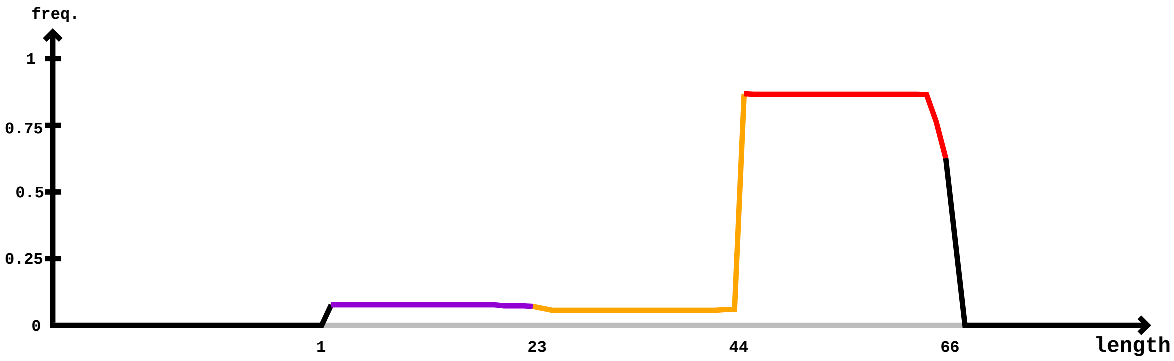


```
                                    Star                                       Mature
5'- acucaucagucgcuccguguucgaggcuggaaguuaacguagucccggguugcaucgcuguacuuggcccugggacuaugucaacuuacaacugcgcucacgauuuuccuuc  -3'  obs
    acucaucagucgcuccguguucgaggcuggaaguuaacguagucccggguugcaucgcuguacuuggcccugggacuaugucaacuuacaacugcgcucacgauuuuccuuc       exp
    ...............((((..((.((.((..((((.((((((((((((((((((....))))....)))).)))))))))).))))..)).)).))..))))..........   reads   mm   sample
    ...........................uggaaguuaacguagucc...................................................................   8       0    seq
    ...........................uggaaguuaacguagucccgg................................................................   4       0    seq
    ...........................uggaaguuaacguaguccUggg...............................................................   1       1    seq
    ...........................uggaaguuaacguagucccggg...............................................................   109     0    seq
    ...........................uAgaaguuaacguagucccggg...............................................................   1       1    seq
    ...........................uggaaguuaacguagucccgggA..............................................................   30      1    seq
    ...........................uggaaguuaacguagucccgggu..............................................................   1       0    seq
    .................................................Augcaucgcuguacuuggccc..........................................   1       1    seq
    .................................................uugcaucgcuguacuuggccc..........................................   107     0    seq
    .................................................uugcaucgcuguacuuggcccu.........................................   5       0    seq
    ....................................................................ccugggacuaugucaacuuac.......................   1       0    seq
    ....................................................................ccugggacuaugucaacuuaca......................   2       0    seq
    ....................................................................Ucugggacuaugucaacuuacaac....................   2       1    seq
    .....................................................................cugggacuaugucaacuuacaa.....................   1       0    seq
    ......................................................................ugggacuaugucaacuuac.......................   2       0    seq
    ......................................................................uAggacuaugucaacuuaca......................   1       1    seq
    ......................................................................ugggacuaugucaacuuaca......................   197     0    seq
    ......................................................................Agggacuaugucaacuuaca......................   1       1    seq
    ......................................................................uAggacuaugucaacuuacaa.....................   2       1    seq
    ......................................................................ugggacuaugucaacuuacaU.....................   1       1    seq
    ......................................................................ugggacuaugucaacuuacaa.....................   271     0    seq
    ......................................................................ugggacuaugucaacuuacaac....................   1106    0    seq
    ......................................................................ugggacuaugucaGcuuacaac....................   1       1    seq
    ......................................................................ugggacuaugucaacuuacGac....................   1       1    seq
    ......................................................................ugggacuaugucaacuuGcaac....................   1       1    seq
    ......................................................................ugggacuaugucaacuuacaacA...................   3       1    seq
    ......................................................................ugggacuauCucaacuuacaac....................   1       1    seq
    ......................................................................ugAgacuaugucaacuuacaac....................   1       1    seq
    ......................................................................ugggacuaugucaacuuacaaU....................   11      1    seq
    ......................................................................ugggacuauguUaacuuacaac....................   1       1    seq
    ......................................................................ugggacuaugucaaAuuacaac....................   1       1    seq
    ......................................................................ugggacuauguAaacuuacaac....................   1       1    seq
    ......................................................................ugggacuaugucaUcuuacaac....................   1       1    seq
```

```
                             Star                                          Mature
acucaucagucgcuccguguucgaggcuggaaguuaacguagucccggguugcaucgcuguacuuggcccugggacuaugucaacuuacaacugcgcucacgauuuuccuuc
......................................................................ugggacuaugucaacuuUcaac....................    1    1    seq
......................................................................uggAacuaugucaacuuacaac....................    1    1    seq
......................................................................ugggacuaCgucaacuuacaac....................    1    1    seq
......................................................................uAggacuaugucaacuuacaac....................    2    1    seq
......................................................................Agggacuaugucaacuuacaac....................    4    1    seq
......................................................................ugggacuaugucaacuuacaacG...................    1    1    seq
......................................................................ugggacuaugucaacuuacaacu...................   45    0    seq
......................................................................ugggacuaugucaacuuacaacA...................   66    1    seq
.......................................................................gggGcuaugucaacuuaca......................    1    1    seq
```
