# Supplementary material for: The Small RNA Universe of Capitella teleta
Source: Front Mol Biosci. 2022 Feb 25;9:802814. doi: 10.3389/fmolb.2022.802814 (PMC8915122; doi:10.3389/fmolb.2022.802814)
Supplement: Supplementary file 1 [file DataSheet1.ZIP › Supplement/homologRecovered/CAPTEscaffold_365_20227.pdf]

Provisional ID	:	CAPTEscaffold_365_20227
Score total	:	10.2
Score for star read(s)	:	3.9
Score for read counts	:	0.1
Score for mfe	:	1.6
Score for randfold	:	1.6
Score for cons. seed	:	3
Total read count	:	12
Mature read count	:	4
Loop read count	:	0
Star read count	:	8

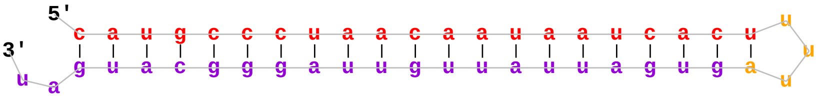

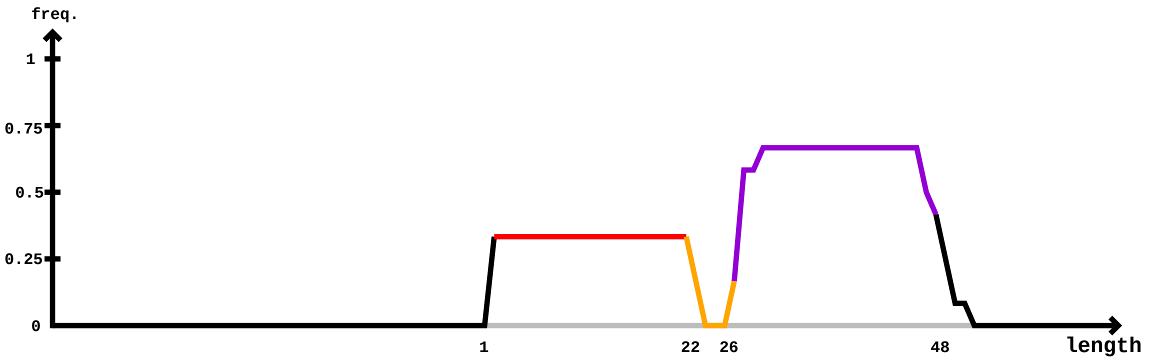

Mature Star

```
5'- ucgguuguuaucgcuaauacaucuuucucugaauaccacauaugcaugcccuaacaauaaucacuuuuagugauuauuguuagggcaugaucggaauuuuagaauuuca  -3'  obs
    ucgguuguuaucgcuaauacaucuuucucugaauaccacauaugcaugcccuaacaauaaucacuuuuagugauuauuguuagggcaugaucggaauuuuagaauuuca       exp
    .((((....))))..............((((((..((.......(((((((((((((((((((((...)))))))))))))))))))))...))...)))))).......  reads  mm  sample
    ............................................caugcccuaacaauaaucacu............................................  4      0   seq
    .....................................................................gugauuauuguuagggcaugau..................  2      0   seq
    ......................................................................ugauuauuguuagggcaug....................  2      0   seq
    ......................................................................ugauuauuguuagggcauga...................  1      0   seq
    ......................................................................ugauuauuguuagggcaugCu..................  1      1   seq
    ......................................................................ugauuauuguuagggcaugauc.................  1      0   seq
    ........................................................................auuauuguuagggcaugaucgU...............  1      1   seq
```
