# Supplementary material for: The Small RNA Universe of Capitella teleta
Source: Front Mol Biosci. 2022 Feb 25;9:802814. doi: 10.3389/fmolb.2022.802814 (PMC8915122; doi:10.3389/fmolb.2022.802814)
Supplement: Supplementary file 1 [file DataSheet1.ZIP › Supplement/homologRecovered/CAPTEscaffold_823_29161.pdf]

Provisional ID	:	CAPTEscaffold_823_29161
Score total	:	639698.9
Score for star read(s)	:	3.9
Score for read counts	:	639688.4
Score for mfe	:	2
Score for randfold	:	1.6
Score for cons. seed	:	3
Total read count	:	1254733
Mature read count	:	1254686
Loop read count	:	0
Star read count	:	47

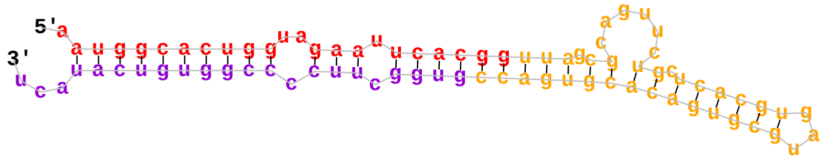

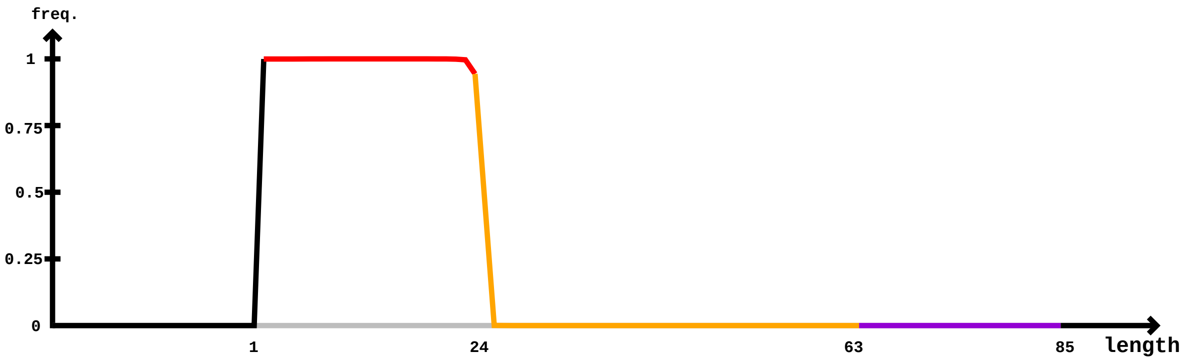

Mature   Star

```
5'- uuuacgcgucuguuaccgauaauggcacugguagaauucacgguuagcgcaguucugcucacgugaugcgugacacgugaccguggcuuccccggugucauacuuggugccag   -3'    obs
    uuuacgcgucuguuaccgauaauggcacugguagaauucacgguuagcgcaguucugcucacgugaugcgugacacgugaccguggcuuccccggugucauacuuggugccag          exp
    .........(((.((((((..((((((((((..(((.(((((((((.((......((.((((((...))))))))))))))))))))).))).))))))))))..)))))).)))   reads   mm   sample
    uuuacgcgucuguuaccgau.............................................................................................   3       0    seq
    .uuacgcgucuguuaccgau.............................................................................................   11      0    seq
    ..................aAaauggcacugguagaauucacgg......................................................................   1       1    seq
    ...................Caauggcacugguagaauucacgg......................................................................   29      1    seq
    ...................Aaauggcacugguagaauucacgg......................................................................   11      1    seq
    ....................aauggcacugguagaauC...........................................................................   1       1    seq
    ....................aauggcacugguagaauu...........................................................................   223     0    seq
    ....................aauggcacugguagaauuc..........................................................................   12      0    seq
    ....................aauggcGcugguagaauuca.........................................................................   1       1    seq
    ....................aauggcacugguagaauuAa.........................................................................   2       1    seq
    ....................Gauggcacugguagaauuca.........................................................................   1       1    seq
    ....................aauAgcacugguagaauuca.........................................................................   2       1    seq
    ....................aauggcacugguagaauuUa.........................................................................   2       1    seq
    ....................aauggcacuggAagaauuca.........................................................................   2       1    seq
    ....................aauggcacugguagaauAca.........................................................................   1       1    seq
    ....................aauggcUcugguagaauuca.........................................................................   1       1    seq
    ....................aauggcacugguagaauuca.........................................................................   970     0    seq
    ....................aauggcacugguagaUuuca.........................................................................   1       1    seq
    ....................aauggcacuAguagaauuca.........................................................................   1       1    seq
    ....................aauggcacugguagaauucG.........................................................................   2       1    seq
    ....................aaAggcacugguagaauuca.........................................................................   1       1    seq
    ....................aauggcacugguagGauuca.........................................................................   1       1    seq
    ....................aauggcacuUguagaauucac........................................................................   1       1    seq
    ....................aauggcacuggAagaauucac........................................................................   1       1    seq
    ....................aauggcacuAguagaauucac........................................................................   7       1    seq
    ....................aauggcacugguagUauucac........................................................................   1       1    seq
    ....................aauggcacugguGgaauucac........................................................................   2       1    seq
    ....................aauggcacugguagaauucaA........................................................................   3       1    seq
    ....................aaAggcacugguagaauucac........................................................................   2       1    seq
    ....................aUuggcacugguagaauucac........................................................................   1       1    seq
    ....................aauggcacugguagaauAcac........................................................................   2       1    seq
    ....................aauggcacugguagaauuUac........................................................................   10      1    seq
    ....................aauggGacugguagaauucac........................................................................   1       1    seq
```

```
                    Mature                                                       Star
uuuacgcgucuguuaccgauaauggcacugguagaauucacgguuagcgcaguucugcucacgugaugcgugacacgugaccguggcuuccccggugucauacuuggugccag
....................aaugAcacugguagaauucac........................................................................ 2     1   seq
....................aauCgcacugguagaauucac........................................................................ 1     1   seq
....................aauggcacugguagaauucGc........................................................................ 1     1   seq
....................aauggcacugUuagaauucac........................................................................ 1     1   seq
....................aauggcacugguagaauucUc........................................................................ 4     1   seq
....................aauAgcacugguagaauucac........................................................................ 5     1   seq
....................aauggcacugguagaauucac........................................................................ 3077  0   seq
....................aauggcacugguagaauGcac........................................................................ 1     1   seq
....................aauggcacugguagaaAucac........................................................................ 1     1   seq
....................Cauggcacugguagaauucac........................................................................ 4     1   seq
....................Gauggcacugguagaauucac........................................................................ 2     1   seq
....................aauggcacugguaAaauucac........................................................................ 1     1   seq
....................aauggcacugguagaauuAac........................................................................ 1     1   seq
....................aGuggcacugguagaauucac........................................................................ 1     1   seq
....................aauggcaGugguagaauucac........................................................................ 2     1   seq
....................aauggcacugguagGauucac........................................................................ 2     1   seq
....................aauggAacugguagaauucac........................................................................ 1     1   seq
....................aauggcacugguagaauucaG........................................................................ 1     1   seq
....................aauggcacuggCagaauucac........................................................................ 1     1   seq
....................aauggNacugguagaauucacg....................................................................... 1     1   seq
....................aauggcGcugguagaauucacg....................................................................... 22    1   seq
....................aauggcacugguagaauucacU....................................................................... 7     1   seq
....................aauggcacugguagaUuucacg....................................................................... 5     1   seq
....................aauggcacugguagaauuAacg....................................................................... 30    1   seq
....................aauggcacugguagaaAucacg....................................................................... 15    1   seq
....................aauggcacugguagaauucUcg....................................................................... 4     1   seq
....................aauggcacugguUgaauucacg....................................................................... 8     1   seq
....................aauggcacuAguagaauucacg....................................................................... 164   1   seq
....................aauggcacuggAagaauucacg....................................................................... 39    1   seq
....................aauggcacuggGagaauucacg....................................................................... 1     1   seq
....................aaugCcacugguagaauucacg....................................................................... 1     1   seq
....................aauggcacugguagaauucaUg....................................................................... 9     1   seq
....................aauggcacugguGgaauucacg....................................................................... 18    1   seq
....................Uauggcacugguagaauucacg....................................................................... 4     1   seq
....................aGuggcacugguagaauucacg....................................................................... 6     1   seq
....................aauggcacGgguagaauucacg....................................................................... 4     1   seq
....................aauAgcacugguagaauucacg....................................................................... 71    1   seq
....................aauggcacugUuagaauucacg....................................................................... 8     1   seq
....................aauggcUcugguagaauucacg....................................................................... 11    1   seq
....................aauUgcacugguagaauucacg....................................................................... 3     1   seq
....................aauggcacugguagaaGucacg....................................................................... 2     1   seq
....................aauggUacugguagaauucacg....................................................................... 5     1   seq
....................aauggcacugguagaauAcacg....................................................................... 24    1   seq
....................aauggcacugguagaauCcacg....................................................................... 4     1   seq
....................aauggcacugguagaauucaAg....................................................................... 18    1   seq
....................aauggcacugguagaaCucacg....................................................................... 5     1   seq
....................aauggcacuggCagaauucacg....................................................................... 6     1   seq
....................aaugAcacugguagaauucacg....................................................................... 7     1   seq
....................aUuggcacugguagaauucacg....................................................................... 3     1   seq
....................aauggcaAugguagaauucacg....................................................................... 4     1   seq
....................aauggcacugguagaauucGcg....................................................................... 17    1   seq
....................aauggcaUugguagaauucacg....................................................................... 3     1   seq
....................aauggcacugguagGauucacg....................................................................... 11    1   seq
....................aauggcacugAuagaauucacg....................................................................... 14    1   seq
....................Nauggcacugguagaauucacg....................................................................... 28    1   seq
....................aauggcacAgguagaauucacg....................................................................... 17    1   seq
....................aauggcacuCguagaauucacg....................................................................... 11    1   seq
....................aauggcacugguagaauuUacg....................................................................... 46    1   seq
....................aauggcacugguaUaauucacg....................................................................... 4     1   seq
....................aaGggcacugguagaauucacg....................................................................... 1     1   seq
....................aauggcacuUguagaauucacg....................................................................... 7     1   seq
....................aaugUcacugguagaauucacg....................................................................... 9     1   seq
....................aauggcacugguaCaauucacg....................................................................... 4     1   seq
....................aauggGacugguagaauucacg....................................................................... 12    1   seq
....................aauggcCcugguagaauucacg....................................................................... 1     1   seq
....................aauggcacugguagaauucacA....................................................................... 32    1   seq
....................aauggcacugguagUauucacg....................................................................... 5     1   seq
....................aauggcacugguaAaauucacg....................................................................... 21    1   seq
....................aauCgcacugguagaauucacg....................................................................... 7     1   seq
....................aauggAacugguagaauucacg....................................................................... 16    1   seq
```

Mature **Star**

uuuacgcgucuguuaccgauaauggcacugguagaauucacgguuagcgcaguucugcucacgugaugcgugacacgugaccguggcuuccccggugucauacuuggugccag

....................aauggcacugguagaauucacC...	5	1	seq
....................aauggcacugguagaauuGacg...	8	1	seq
....................aauggcaGugguagaauucacg...	4	1	seq
....................aaAggcacugguagaauucacg...	47	1	seq
....................aauggcacugguagaauGcacg...	7	1	seq
....................aauggcacugCuagaauucacg...	4	1	seq
....................Gauggcacugguagaauucacg...	110	1	seq
....................aaCggcacugguagaauucacg...	5	1	seq
....................aauggcacugguagaauucCcg...	1	1	seq
....................aauggcacugguagaauucacg...	65104	0	seq
....................aauggcacugguagaauucaGg...	28	1	seq
....................aauggcacugguagaGuucacg...	14	1	seq
....................aauggcacCgguagaauucacg...	10	1	seq
....................aauggcacugguagaUuucacgg..	139	1	seq
....................aauggcacugguagCauucacgg..	4	1	seq
....................aauggcacugguaUaauucacgg..	79	1	seq
....................aauggcacuUguagaauucacgg..	133	1	seq
....................aauggcacugguagaauucacgU..	455	1	seq
....................aauggcacugguagaauucacgN..	6	1	seq
....................aauggcacugguagaauGcacgg..	56	1	seq
....................aauggcacugguagaauCcacgg..	117	1	seq
....................aaNggcacugguagaauucacgg..	5	1	seq
....................aauggcacugguagaauucCcgg..	26	1	seq
....................aauggcacugguagUauucacgg..	42	1	seq
....................aauggcacuggCagaauucacgg..	158	1	seq
....................aaugAcacugguagaauucacgg..	210	1	seq
....................aauggNacugguagaauucacgg..	14	1	seq
....................aauggcacugguaAaauucacgg..	317	1	seq
....................aauUgcacugguagaauucacgg..	85	1	seq
....................aNuggcacugguagaauucacgg..	53	1	seq
....................aauggcacuNguagaauucacgg..	3	1	seq
....................aauggcacGgguagaauucacgg..	58	1	seq
....................aGuggcacugguagaauucacgg..	190	1	seq
....................aauggcacugguaCaauucacgg..	46	1	seq
....................aauggcacugCuagaauucacgg..	57	1	seq
....................Nauggcacugguagaauucacgg..	342	1	seq
....................aauggcaAugguagaauucacgg..	110	1	seq
....................aauggcacugguagaauucaAgg..	264	1	seq
....................aauggcacugguagaaGucacgg..	59	1	seq
....................aauggcacugguUgaauucacgg..	138	1	seq
....................aUuggcacugguagaauucacgg..	99	1	seq
....................aaugCcacugguagaauucacgg..	62	1	seq
....................aaCggcacugguagaauucacgg..	129	1	seq
....................aauggcCcugguagaauucacgg..	33	1	seq
....................aauggcacugguagaauucacgA..	707	1	seq
....................aauggcacugguagaauAcacgg..	278	1	seq
....................Cauggcacugguagaauucacgg..	11	1	seq
....................aauggcacuCguagaauucacgg..	109	1	seq
....................aauggcGcugguagaauucacgg..	344	1	seq
....................aauggcacugguagaCuucacgg..	7	1	seq
....................aCuggcacugguagaauucacgg..	2	1	seq
....................aauggcacugguGgaauucacgg..	283	1	seq
....................aauggcacAgguagaauucacgg..	227	1	seq
....................aauggcacuAguagaauucacgg..	1962	1	seq
....................aauggcUcugguagaauucacgg..	155	1	seq
....................aauggcacugguagaauuUacgg..	809	1	seq
....................aauggGacugguagaauucacgg..	107	1	seq
....................aauggAacugguagaauucacgg..	284	1	seq
....................aauggcacCgguagaauucacgg..	165	1	seq
....................aauggcacugguaNaauucacgg..	1	1	seq
....................aauggcacuggGagaauucacgg..	16	1	seq
....................aauggcacugguagaauucacgg..	1145036	0	seq
....................aauggcacugguagaauucaGgg..	118	1	seq
....................aauggcacugguagaauucUcgg..	132	1	seq
....................aaugUcacugguagaauucacgg..	95	1	seq
....................aauggcacugguagaaAucacgg..	282	1	seq
....................aauggcacuggAagaauucacgg..	481	1	seq
....................aauAgcacugguagaauucacgg..	979	1	seq
....................aauggcacugguagaauucacCg..	80	1	seq
....................aauggcacugguagaaNucacgg..	3	1	seq

```
                    Mature                                                         Star
uuuacgcgucuguuaccgauaauggcacugguagaauucacgguuagcgcaguucugcucacgugaugcgugacacgugaccguggcuuccccggugucauacuuggugccag
....................aaAggcacugguagaauucacgg......................................................................    786      1    seq
....................aauggcacugguagaGuucacgg......................................................................    237      1    seq
....................aauggcacugguagaauucaNgg......................................................................    1        1    seq
....................aauggcacugguagaauucaUgg......................................................................    296      1    seq
....................aauggcaGugguagaauucacgg......................................................................    51       1    seq
....................aauggcacugguagaauucacNg......................................................................    1        1    seq
....................aauggcacugAuagaauucacgg......................................................................    208      1    seq
....................aauggcacugguagaauucacgC......................................................................    433      1    seq
....................aauggcaUugguagaauucacgg......................................................................    88       1    seq
....................aauggcacugguagaauucacAg......................................................................    1879     1    seq
....................aauggcacugguagaauucacUg......................................................................    197      1    seq
....................aauggcacugguCgaauucacgg......................................................................    8        1    seq
....................aauCgcacugguagaauucacgg......................................................................    105      1    seq
....................aauggcacugguagaauuNacgg......................................................................    1        1    seq
....................Gauggcacugguagaauucacgg......................................................................    1849     1    seq
....................aauggcacugguagaauucGcgg......................................................................    317      1    seq
....................aauggcacugguagaaCucacgg......................................................................    118      1    seq
....................aauggcacugguagaauuAacgg......................................................................    520      1    seq
....................aauggUacugguagaauucacgg......................................................................    203      1    seq
....................aauggcacugguagaauucNcgg......................................................................    1        1    seq
....................aauggcacugguagGauucacgg......................................................................    136      1    seq
....................aauggcacugguagaauuGacgg......................................................................    117      1    seq
....................Uauggcacugguagaauucacgg......................................................................    82       1    seq
....................aauggcacugUuagaauucacgg......................................................................    125      1    seq
....................aaGggcacugguagaauucacgg......................................................................    20       1    seq
....................aauggcacugguagaauucaUggu.....................................................................    2        1    seq
....................aauggcGcugguagaauucacggu.....................................................................    1        1    seq
....................aauggcUcugguagaauucacggu.....................................................................    2        1    seq
....................aauAgcacugguagaauucacggu.....................................................................    3        1    seq
....................aauggcacugguaAaauucacggu.....................................................................    2        1    seq
....................aauggcacuggCagaauucacggu.....................................................................    1        1    seq
....................aauggcacugguGgaauucacggu.....................................................................    2        1    seq
....................aauggcacugguagaauucacCgu.....................................................................    1        1    seq
....................aauggcacugguagaauucGcggu.....................................................................    3        1    seq
....................aauggcacugguagaauucacgUu.....................................................................    4        1    seq
....................aauggcacugCuagaauucacggu.....................................................................    1        1    seq
....................aauggcacugAuagaauucacggu.....................................................................    1        1    seq
....................aauggcacugguagaauucacggC.....................................................................    23       1    seq
....................aauggcacugguagaauucacAgu.....................................................................    3        1    seq
....................aauUgcacugguagaauucacggu.....................................................................    1        1    seq
....................aauggcacugguagaauuUacggu.....................................................................    8        1    seq
....................aauggcacugUuagaauucacggu.....................................................................    1        1    seq
....................aauggcacuAguagaauucacggu.....................................................................    5        1    seq
....................aauggcacugguagaauCcacggu.....................................................................    1        1    seq
....................aauggGacugguagaauucacggu.....................................................................    1        1    seq
....................aauggcacAgguagaauucacggu.....................................................................    1        1    seq
....................aauggcacugguagaauucacgCu.....................................................................    3        1    seq
....................aauggcacugguUgaauucacggu.....................................................................    1        1    seq
....................aauggcacugguagaauAcacggu.....................................................................    1        1    seq
....................aauggcacugguagaauuAacggu.....................................................................    2        1    seq
....................aauggcacugguagaaAucacggu.....................................................................    3        1    seq
....................aauggcacugguagaauucacggG.....................................................................    227      1    seq
....................aauggcacugguagaauucacggN.....................................................................    2        1    seq
....................aauggUacugguagaauucacggu.....................................................................    2        1    seq
....................aauggcacugguagaauucacggA.....................................................................    14233    1    seq
....................Nauggcacugguagaauucacggu.....................................................................    2        1    seq
....................aauggcacugguagaauucacgAu.....................................................................    1        1    seq
....................Gauggcacugguagaauucacggu.....................................................................    4        1    seq
....................aauggcacugguagaGuucacggu.....................................................................    3        1    seq
....................aauggcacugguagaauucacggu.....................................................................    4405     0    seq
....................aauggcacugguagaauucacgguC....................................................................    3        1    seq
....................aaAggcacugguagaauucacgguu....................................................................    1        1    seq
....................aauggcacugguagaauuUacgguu....................................................................    1        1    seq
....................aauggcacugguagaauucacggGu....................................................................    1        1    seq
....................aauggcacugguagaauucacggAu....................................................................    31       1    seq
....................aauggcacugguagaauucacgCuu....................................................................    1        1    seq
....................aauggcacugguagaauucacgguu....................................................................    284      0    seq
....................aauggcacugguagaauucacgUuu....................................................................    2        1    seq
....................Gauggcacugguagaauucacgguu....................................................................    1        1    seq
....................aauggcacugguagaauucacgguA....................................................................    63       1    seq
```

Mature **Star**

```
uuuacgcgucuguuaccgauaauggcacugguagaauucacgguuagcgcaguucugcucacgugaugcgugacacgugaccguggcuuccccggugucauacuuggugccag
....................aauggcacugguagaauucacgguG....................................................................  1    1  seq
....................aauggcacugguagaauucacggCu....................................................................  2    1  seq
....................aUuggcacugguagaauucacgguu....................................................................  1    1  seq
....................aauggcacuAguagaauucacgguu....................................................................  2    1  seq
....................aauggcacugguagaauucacggAua...................................................................  5    1  seq
....................aauggcacugguagaauucacgguAa...................................................................  214  1  seq
....................aauggcacugguagaauucacgguuU...................................................................  19   1  seq
....................aauggcacugguagaauucacgguuC...................................................................  1    1  seq
....................aauggcacugguagaauucacgguua...................................................................  11   0  seq
....................aauggcacugguagaauucacgguuaU..................................................................  1    1  seq
....................aauggcacugguagaauucacgguuagc.................................................................  1    0  seq
....................aauggcacugguagaauucacgguuagcgc...............................................................  1    0  seq
.....................auggcacugguagCauucacgg......................................................................  1    1  seq
.....................auggcacugguagaauuAacgg......................................................................  1    1  seq
.....................auggcGcugguagaauucacgg......................................................................  1    1  seq
.....................aAggcacugguagaauucacgg......................................................................  5    1  seq
.....................auggcacugguagaauucacgg......................................................................  42   0  seq
.....................augCcacugguagaauucacgg......................................................................  1    1  seq
.....................auggcUcugguagaauucacgg......................................................................  1    1  seq
.....................auggcacugguagaauucacggu.....................................................................  6    0  seq
.....................auggcacugguagaauucacggA.....................................................................  2    1  seq
.....................auggcacugguagaauucacgguAa...................................................................  1    1  seq
......................uggcacugguagaauucacgg......................................................................  8    0  seq
......................uggcacugguagaauucacggA.....................................................................  1    1  seq
.......................ggcacugguagaauucacgg......................................................................  6    0  seq
........................gcacugguagaauucacg.......................................................................  5    0  seq
........................gcacugguagaauucacgg......................................................................  256  0  seq
........................gcacugCuagaauucacgg......................................................................  1    1  seq
........................Ucacugguagaauucacgg......................................................................  1    1  seq
........................gcacuAguagaauucacgg......................................................................  2    1  seq
........................gcacugguagaauucacAg......................................................................  1    1  seq
........................gcacugguagaauucacggA.....................................................................  5    1  seq
........................gcacugguagaauucacgguA....................................................................  1    1  seq
.........................cacugguagaauucacgg......................................................................  235  0  seq
.........................caAugguagaauucacgg......................................................................  1    1  seq
.........................cacugguaAaauucacgg......................................................................  1    1  seq
.........................cacugguagaauucacgU......................................................................  1    1  seq
.........................cacugguagaauucacUgu.....................................................................  14   1  seq
.........................cacugguagaauuUacggu.....................................................................  1    1  seq
.........................cacugguagaauucacggA.....................................................................  7    1  seq
..........................acugguagaauucacUgu.....................................................................  2    1  seq
..........................acugguagaauucacggu.....................................................................  3    0  seq
..........................acugguagaauucacggA.....................................................................  2    1  seq
...........................cugguagaauucacgguua...................................................................  1    0  seq
........................................................................acacgugaccguggcuucccU....................  1    1  seq
.................................................................................cguggcuuccccggugucauacu.........  1    0  seq
..................................................................................guggcuuccccggugucauacu.........  35   0  seq
...................................................................................uggcuuccccggugucauacu.........  11   0  seq
```
