# Supplementary material for: The Small RNA Universe of Capitella teleta
Source: Front Mol Biosci. 2022 Feb 25;9:802814. doi: 10.3389/fmolb.2022.802814 (PMC8915122; doi:10.3389/fmolb.2022.802814)
Supplement: Supplementary file 1 [file DataSheet1.ZIP › Supplement/homologRecovered/CAPTEscaffold_70_6932.pdf]

Provisional ID : CAPTEscaffold_70_6932
Score total : 1079.4
Score for star read(s) : 3.9
Score for read counts : 1069.2
Score for mfe : 1.7
Score for randfold : 1.6
Score for cons. seed : 3
Total read count : 2109
Mature read count : 2083
Loop read count : 0
Star read count : 26

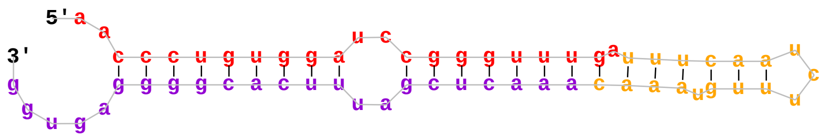

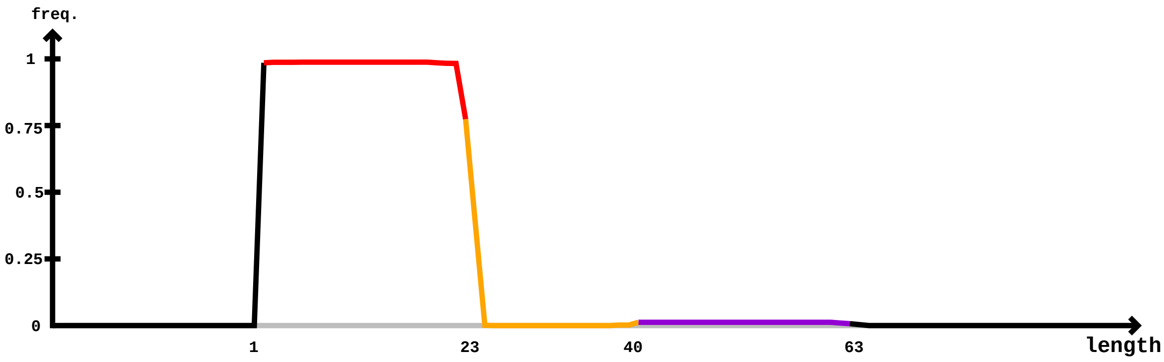

Mature Star

```
5'- gaccuauuugccugcgucuaaacccuguggauccggguuugauuucaaucuuuguaaacaaacucgauuucacggggagugggucaggugaaucacagcaacuuuuuauu  -3'   obs
    gaccuauuugccugcgucuaaacccuguggauccggguuugauuucaaucuuuguaaacaaacucgauuucacggggagugggucaggugaaucacagcaacuuuuuauu         exp
    .....((((((((..((((..(((((((((..((((((((.((((((...))).))))))))))..)))))))))..))))..))))))))................   reads   mm   sample
    ....................aacccuguggauccgggG.........................................................................   1       1    seq
    ....................Gacccuguggauccgggu.........................................................................   1       1    seq
    ....................aacccuguggauccgggu.........................................................................   3       0    seq
    ....................aacccuguggauccggguu........................................................................   4       0    seq
    ....................aacccuguggauccggguuu.......................................................................   1       0    seq
    ....................aacccuguggGuccggguuug......................................................................   1       1    seq
    ....................aacccuguggUuccggguuug......................................................................   1       1    seq
    ....................aacccuguAgauccggguuug......................................................................   1       1    seq
    ....................Nacccuguggauccggguuug......................................................................   1       1    seq
    ....................aacccuguggauccggguuug......................................................................   433     0    seq
    ....................aacccuguggauAcggguuug......................................................................   1       1    seq
    ....................aacccuguggauccgggGuug......................................................................   1       1    seq
    ....................aacccugAggauccggguuug......................................................................   1       1    seq
    ....................aaccUuguggauccggguuug......................................................................   1       1    seq
    ....................aacccuguggaCccggguuuga.....................................................................   1       1    seq
    ....................aaccUuguggauccggguuuga.....................................................................   1       1    seq
    ....................aacccuguggauccggguAuga.....................................................................   1       1    seq
    ....................aacAcuguggauccggguuuga.....................................................................   2       1    seq
    ....................aacUcuguggauccggguuuga.....................................................................   1       1    seq
    ....................aacccuguggauccggguuuAa.....................................................................   2       1    seq
    ....................aacccuguggauccggguuugU.....................................................................   27      1    seq
    ....................aacccuguAgauccggguuuga.....................................................................   7       1    seq
    ....................aacccugAggauccggguuuga.....................................................................   2       1    seq
    ....................aacccuguggaAccggguuuga.....................................................................   1       1    seq
    ....................aacccuguggauccggguuuga.....................................................................   1555    0    seq
    ....................aUcccuguggauccggguuuga.....................................................................   1       1    seq
    ....................Gacccuguggauccggguuuga.....................................................................   3       1    seq
    ....................aacccuguggauccgAguuuga.....................................................................   2       1    seq
    ....................aacccugugAauccggguuuga.....................................................................   1       1    seq
    ....................aacccuguggauccAgguuuga.....................................................................   3       1    seq
    ....................aacccuguggauccggguCuga.....................................................................   1       1    seq
    ....................aacccuguggauccggguuCga.....................................................................   1       1    seq
    ....................aacccuguggauccggguuugG.....................................................................   5       1    seq
```

Mature **Star**

gaccuauuugccugcgucuaaacccuguggauccggguuugauuucaaucuuuguaaacaaacucgauuucacggggaguggggucaggugaaucacagcaacuuuuuauu			
....................aacccuguggauccggguuugau..	2	0	seq
....................aacccuguggauccggguuugaA..	1	1	seq
....................aacccuguggauccggguuugUu..	3	1	seq
....................aacccuguggauccggguuugaG..	3	1	seq
....................aacccuguggauccggguuugauu...	1	0	seq
.....................acccuguggauccggguuuga...	2	0	seq
.....................acccuguggauccggguuugau..	1	0	seq
.....................acccuguggauccggguuugauu...	1	0	seq
........................cuguggauccggguuuga...	1	0	seq
....acaaacucgauuucacggggagu...............................	4	0	seq
....aaacucgauuucacggggagu...............................	2	0	seq
....aaacucgauuucacggggagug..............................	5	0	seq
....aaacucgauuucacggggagugU.............................	14	1	seq
....aaacucgauuucacggggagugg.............................	1	0	seq
