# Supplementary material for: The Small RNA Universe of Capitella teleta
Source: Front Mol Biosci. 2022 Feb 25;9:802814. doi: 10.3389/fmolb.2022.802814 (PMC8915122; doi:10.3389/fmolb.2022.802814)
Supplement: Supplementary file 1 [file DataSheet1.ZIP › Supplement/homologRecovered/CAPTEscaffold_384_18638.pdf]

Provisional ID	:	CAPTEscaffold_384_18638
Score total	:	59473.2
Score for star read(s)	:	3.9
Score for read counts	:	59462.6
Score for mfe	:	2.2
Score for randfold	:	1.6
Score for cons. seed	:	3
Total read count	:	116645
Mature read count	:	116581
Loop read count	:	5
Star read count	:	59

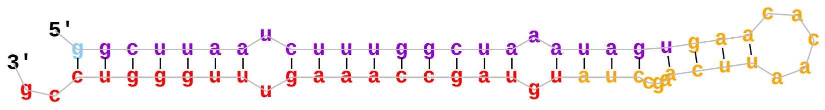

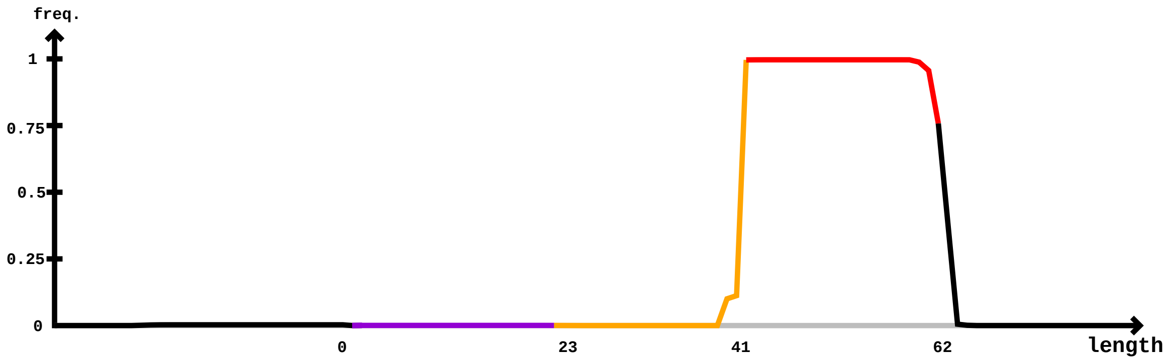

Star Mature

```
5'- gaucgggauuccauaugccaucggaggcgggcuuaaucuuuggcuaaauagugaacacaauucaagccuauguagccaaaguuuggguccgucucuauggcuaccucaucg  -3'   obs
    gaucgggauuccauaugccaucggaggcgggcuuaaucuuuggcuaaauagugaacacaauucaagccuauguagccaaaguuuggguccgucucuauggcuaccucaucg        exp
    (((.(((.........(((((.((((((((((((((.(((((((((.((((((((.....))))...)))).))))))))).))))))))))))))))))))..))).))).  reads   mm   sample
    ......gauuccauaugccaucggaggcg..................................................................................  1       0    seq
    .......auuccauaugccaucggaggcg..................................................................................  122     0    seq
    .......auuccauaugccaucggaggcgg.................................................................................  2       0    seq
    ........uucUauaugccaucggaggcg..................................................................................  1       1    seq
    ........Auccauaugccaucggaggcg..................................................................................  1       1    seq
    ........uuccauaugccaucggaggcg..................................................................................  140     0    seq
    .........Accauaugccaucggaggcg..................................................................................  1       1    seq
    .........uccauaugccaucggaggcg..................................................................................  65      0    seq
    ..........ccauaugccaucggaggcg..................................................................................  2       0    seq
    .............................ggcuuaaucuuuggcuaaaua.............................................................  10      0    seq
    ..............................gcuuaaucuuuggcuaaauag............................................................  1       0    seq
    ..............................gcuuaaucuuuggcuaaauagu...........................................................  47      0    seq
    ..............................Ucuuaaucuuuggcuaaauagu...........................................................  1       1    seq
    ...................................................ugaacacaauucaagccu..........................................  5       0    seq
    ....................................................................uauguagccaaaguuugg.........................  3       0    seq
    ....................................................................uauguagccaaaguuuggA........................  3       1    seq
    ....................................................................uauguagccaaaguuuggg........................  1       0    seq
    ....................................................................uauguagccaaaguuugggu.......................  302     0    seq
    ....................................................................uauguagccaaaguuuAggu.......................  1       1    seq
    ....................................................................Aauguagccaaaguuugggu.......................  1       1    seq
    ....................................................................uauguagccaaagAuugggu.......................  1       1    seq
    ....................................................................uauguagccaaaguuuggguA......................  1       1    seq
    ....................................................................uauguagccaaaguuuggguc......................  1339    0    seq
    ....................................................................Cauguagccaaaguuuggguc......................  1       1    seq
    ....................................................................uaAguagccaaaguuuggguc......................  2       1    seq
    ....................................................................Aauguagccaaaguuuggguc......................  2       1    seq
    ....................................................................uauguagccaaaguuugUguc......................  1       1    seq
    ....................................................................uauguagcUaaaguuuggguc......................  2       1    seq
    ....................................................................uauguagccaaaguuuggguU......................  26      1    seq
    ....................................................................uauguagccaaaguCuggguc......................  2       1    seq
    ....................................................................uauguagccaaaguuuAgguc......................  2       1    seq
    ....................................................................uauguaUccaaaguuuggguc......................  1       1    seq
    ....................................................................uauguagccaaaguuuggCuc......................  1       1    seq
```

Star **Mature**

```
gaucgggauuccauaugccaucggaggcgggcuuaaucuuuggcuaaaauagugaacacaauucaagccuauguagccaaaguuuggguccgucucuauggcuaccucaucg
.....................................................................uauguagccaaGguuuggguc......................	1	1	seq
.....................................................................uauguagccaaaguuugggGc......................	1	1	seq
.....................................................................uauguagccaaaguuuggggucU.....................	212	1	seq
.....................................................................uauguagccaaaguuuggguAc.....................	10	1	seq
.....................................................................uauguagccaaaAuuugggucc.....................	1	1	seq
.....................................................................uauguagccaaaUuuugggucc.....................	1	1	seq
.....................................................................uauguagccaaaguAugggucc.....................	3	1	seq
.....................................................................uauguagccaaaguuuUggucc.....................	4	1	seq
.....................................................................uauguagccaaagGuugggucc.....................	1	1	seq
.....................................................................uaCguagccaaaguuugggucc.....................	2	1	seq
.....................................................................uaAguagccaaaguuugggucc.....................	4	1	seq
.....................................................................uauguaUccaaaguuugggucc.....................	1	1	seq
.....................................................................uauguaAccaaaguuugggucc.....................	1	1	seq
.....................................................................uaNguagccaaaguuugggucc.....................	1	1	seq
.....................................................................uauUuagccaaaguuugggucc.....................	1	1	seq
.....................................................................uUuguagccaaaguuugggucc.....................	4	1	seq
.....................................................................uauguagccaaaguuugggucA.....................	4	1	seq
.....................................................................uauguaCccaaaguuugggucc.....................	1	1	seq
.....................................................................uauguagccaaaguuugggucG.....................	4	1	seq
.....................................................................uauguagccUaaguuugggucc.....................	3	1	seq
.....................................................................uauguagccaaaguuuAggucc.....................	21	1	seq
.....................................................................uauguagccaaaguuuggguNc.....................	1	1	seq
.....................................................................uauguagccaaGguuugggucc.....................	4	1	seq
.....................................................................uauguagccaaaguuCgggucc.....................	1	1	seq
.....................................................................uauguagccaaaguuuggAucc.....................	3	1	seq
.....................................................................uauguagAcaaaguuugggucc.....................	5	1	seq
.....................................................................uauguagccaaaguuGgggucc.....................	1	1	seq
.....................................................................uauguagcAaaaguuugggucc.....................	2	1	seq
.....................................................................uauguagccaaaguuugggGcc.....................	1	1	seq
.....................................................................uauguagccaaagAuugggucc.....................	1	1	seq
.....................................................................uauguagcUaaaguuugggucc.....................	4	1	seq
.....................................................................uauguagccaaaguCugggucc.....................	1	1	seq
.....................................................................uauguagccaaaguuAgggucc.....................	5	1	seq
.....................................................................uaugCagccaaaguuugggucc.....................	1	1	seq
.....................................................................uauguagccaGaguuugggucc.....................	2	1	seq
.....................................................................Cauguagccaaaguuugggucc.....................	1	1	seq
.....................................................................Gauguagccaaaguuugggucc.....................	7	1	seq
.....................................................................uaugAagccaaaguuugggucc.....................	8	1	seq
.....................................................................uauguagccaaaguuugAgucc.....................	6	1	seq
.....................................................................uauguagcGaaaguuugggucc.....................	1	1	seq
.....................................................................uauguGgccaaaguuugggucc.....................	5	1	seq
.....................................................................Nauguagccaaaguuugggucc.....................	3	1	seq
.....................................................................Aauguagccaaaguuugggucc.....................	25	1	seq
.....................................................................uauguagccaaaguuugggucc.....................	9119	0	seq
.....................................................................uauguagccaaaguuuggguUc.....................	2	1	seq
.....................................................................uaugGagccaaaguuugggucc.....................	1	1	seq
.....................................................................uauguNgccaaaguuugggucc.....................	1	1	seq
.....................................................................uauguagccaaaguuuggguccU....................	507	1	seq
.....................................................................uauguagccaaaguuuggguccA....................	9	1	seq
.....................................................................uauguagccaaaguuuggguccg....................	7	0	seq
.....................................................................uauguagccaaaguuuggguccUu...................	21	1	seq
.....................................................................uauguagccaaaguuuggguccAu...................	1	1	seq
......................................................................auguagccaaaguuugggu.......................	8	0	seq
......................................................................Guguagccaaaguuuggguc......................	1	1	seq
......................................................................auguagccaaagGuuggguc......................	1	1	seq
......................................................................auguagccaaaguuuggguc......................	157	0	seq
......................................................................augAagccaaaguuugggucc.....................	1	1	seq
......................................................................aAguagccaaaguuugggucc.....................	1	1	seq
......................................................................auguagccaaaguuuggguUc.....................	1	1	seq
......................................................................auguagccaaGguuugggucc.....................	3	1	seq
......................................................................auguagccaaaguuGgggucc.....................	1	1	seq
......................................................................auguagccaaaguCugggucc.....................	1	1	seq
......................................................................Nuguagccaaaguuugggucc.....................	2	1	seq
......................................................................auguagccaaaguuugggucU.....................	12	1	seq
......................................................................auguagccaaaguuugggucc.....................	1050	0	seq
......................................................................Cuguagccaaaguuugggucc.....................	1	1	seq
......................................................................Guguagccaaaguuugggucc.....................	1	1	seq
......................................................................auguagAcaaaguuugggucc.....................	4	1	seq
......................................................................Cuguagccaaaguuuggguccg....................	3	1	seq
......................................................................auguagccaaaguuuggguccA....................	1	1	seq
```

Star Mature

```
gaucgggauuccauaugccaucggaggcgggcuuaaucuuuggcuaaaauagugaacacaauucaagccuauguagccaaaguuuggguccgucucuauggcuaccucaucg
......................................................................auguagccaaaguuuggguccC....................    1       1   seq
......................................................................auguagccaaaguuuggguccU....................    42      1   seq
......................................................................auguagccUaaguuuggguccg....................    1       1   seq
......................................................................auguagccaaaguuuggguccg....................    97      0   seq
......................................................................auguagccaaaguuuggguccUu...................    3       1   seq
......................................................................auguagccaaaguuuggguccgu...................    2       0   seq
......................................................................auguagccaaaguuuggguccguU..................    1       1   seq
.......................................................................ugAagccaaaguuugggu.......................    1       1   seq
.......................................................................uguagccaaaguuugggu.......................    699     0   seq
.......................................................................uguaAccaaaguuugggu.......................    1       1   seq
.......................................................................uguagAcaaaguuugggu.......................    1       1   seq
.......................................................................uguagccaaaguuugggA.......................    2       1   seq
.......................................................................Aguagccaaaguuugggu.......................    1       1   seq
.......................................................................uguagccaaaguuugggG.......................    4       1   seq
.......................................................................Gguagccaaaguuugggu.......................    1       1   seq
.......................................................................uguagccaaaguuugAguc......................    2       1   seq
.......................................................................uguagcGaaaguuuggguc......................    1       1   seq
.......................................................................uguagcAaaaguuuggguc......................    3       1   seq
.......................................................................uguagccaaaguuuAgguc......................    5       1   seq
.......................................................................uguagccaaaguuugCguc......................    1       1   seq
.......................................................................uguagccaaagAuuggguc......................    2       1   seq
.......................................................................uguagccaaaguuuggguU......................    4       1   seq
.......................................................................Aguagccaaaguuuggguc......................    11      1   seq
.......................................................................uguagcUaaaguuuggguc......................    2       1   seq
.......................................................................Gguagccaaaguuuggguc......................    2       1   seq
.......................................................................uguagccaaaguuuggguc......................    2089    0   seq
.......................................................................uguagAcaaaguuuggguc......................    3       1   seq
.......................................................................uguagccUaaguuuggguc......................    1       1   seq
.......................................................................uguagccGaaguuuggguc......................    1       1   seq
.......................................................................uAuagccaaaguuuggguc......................    1       1   seq
.......................................................................uguagccaaaguuugggAc......................    5       1   seq
.......................................................................ugAagccaaaguuuggguc......................    2       1   seq
.......................................................................uguagccaaaguuuUgguc......................    2       1   seq
.......................................................................uguagccaaaguuugUguc......................    1       1   seq
.......................................................................uguagccaaaguCugggucc.....................    13      1   seq
.......................................................................uguagccaaaguAugggucc.....................    2       1   seq
.......................................................................uguaUccaaaguuugggucc.....................    1       1   seq
.......................................................................uguagcAaaaguuugggucc.....................    20      1   seq
.......................................................................uguagccaaaguuugggAcc.....................    2       1   seq
.......................................................................uguagccaGaguuugggucc.....................    2       1   seq
.......................................................................Aguagccaaaguuugggucc.....................    29      1   seq
.......................................................................uguagccaaGguuugggucc.....................    1       1   seq
.......................................................................uguagccaUaguuugggucc.....................    2       1   seq
.......................................................................uguagccaaaguuCgggucc.....................    4       1   seq
.......................................................................uguagccaaaguuugggucc.....................    12430   0   seq
.......................................................................uguagccaaaguuugggucU.....................    46      1   seq
.......................................................................uUuagccaaaguuugggucc.....................    1       1   seq
.......................................................................uguagccaaUguuugggucc.....................    1       1   seq
.......................................................................uguagccaaaguuuggUucc.....................    1       1   seq
.......................................................................uguGgccaaaguuugggucc.....................    1       1   seq
.......................................................................uguagccaaaguuuggguUc.....................    3       1   seq
.......................................................................uguagccaaagAuugggucc.....................    4       1   seq
.......................................................................uguagccaaaguuugggucA.....................    8       1   seq
.......................................................................uguaAccaaaguuugggucc.....................    2       1   seq
.......................................................................Gguagccaaaguuugggucc.....................    10      1   seq
.......................................................................uguagccUaaguuugggucc.....................    1       1   seq
.......................................................................ugAagccaaaguuugggucc.....................    8       1   seq
.......................................................................Nguagccaaaguuugggucc.....................    3       1   seq
.......................................................................uguagccaaagGuugggucc.....................    1       1   seq
.......................................................................uguagccaaaguuAgggucc.....................    1       1   seq
.......................................................................uguUgccaaaguuugggucc.....................    1       1   seq
.......................................................................uguagccaaCguuugggucc.....................    1       1   seq
.......................................................................uguagccaaaguuuCggucc.....................    2       1   seq
.......................................................................uguagccaaaguuuAggucc.....................    21      1   seq
.......................................................................uguagccaaaguuuUggucc.....................    2       1   seq
.......................................................................uguagccaaaguuuggAucc.....................    2       1   seq
.......................................................................uCuagccaaaguuugggucc.....................    1       1   seq
.......................................................................uguagccaaaguuugggCcc.....................    1       1   seq
.......................................................................uguagNcaaaguuugggucc.....................    1       1   seq
.......................................................................uguagccaaagCuugggucc.....................    3       1   seq
```

Star Mature

```
gaucgggauuccauaugccaucggaggcgggcuuaaucuuuggcuaaauagugaacacaauucaagccuauguagccaaaguuuggguccgucucuauggcuaccucaucg
......................................................................uguagAcaaaguuugggucc.....................  9      1  seq
......................................................................uguagcUaaaguuugggucc.....................  5      1  seq
......................................................................uguagGcaaaguuugggucc.....................  1      1  seq
......................................................................uAuagccaaaguuugggucc.....................  1      1  seq
......................................................................uguagccaaaguuugggucG.....................  4      1  seq
......................................................................Cguagccaaaguuugggucc.....................  1      1  seq
......................................................................uguCgccaaaguuugggucc.....................  1      1  seq
......................................................................uguagccaaaguuugAgucc.....................  4      1  seq
......................................................................Nguagccaaaguuuggguccg....................  27     1  seq
......................................................................uguagccaaagAuuggguccg....................  13     1  seq
......................................................................uguagccaaaguuugggucGg....................  5      1  seq
......................................................................uNuagccaaaguuuggguccg....................  6      1  seq
......................................................................uguagccaaaguuuCgguccg....................  2      1  seq
......................................................................uguagccaaaguuugCguccg....................  2      1  seq
......................................................................uguagNcaaaguuuggguccg....................  2      1  seq
......................................................................uguagccaaagCuuggguccg....................  5      1  seq
......................................................................uguagccaaaguuuggguGcg....................  3      1  seq
......................................................................uguagcGaaaguuuggguccg....................  5      1  seq
......................................................................ugCagccaaaguuuggguccg....................  7      1  seq
......................................................................uguagccaaaguuuggguUcg....................  18     1  seq
......................................................................Gguagccaaaguuuggguccg....................  71     1  seq
......................................................................uguagccaaaguuuggAuccg....................  8      1  seq
......................................................................uguagccaaaguuugggucUg....................  88     1  seq
......................................................................uguagcAaaaguuuggguccg....................  108    1  seq
......................................................................uguaAccaaaguuuggguccg....................  12     1  seq
......................................................................uguagcUaaaguuuggguccg....................  40     1  seq
......................................................................uAuagccaaaguuuggguccg....................  15     1  seq
......................................................................uguagccaaUguuuggguccg....................  2      1  seq
......................................................................uguagccUaaguuuggguccg....................  3      1  seq
......................................................................uguagccaaaguuuggguccC....................  78     1  seq
......................................................................uguagccaaaguuuUgguccg....................  21     1  seq
......................................................................uguaUccaaaguuuggguccg....................  5      1  seq
......................................................................Cguagccaaaguuuggguccg....................  10     1  seq
......................................................................Aguagccaaaguuuggguccg....................  181    1  seq
......................................................................uguagccaaGguuuggguccg....................  9      1  seq
......................................................................uguagGcaaaguuuggguccg....................  3      1  seq
......................................................................uguagUcaaaguuuggguccg....................  3      1  seq
......................................................................uguagAcaaaguuuggguccg....................  45     1  seq
......................................................................uguagccaaaguuuggguccg....................  79822  0  seq
......................................................................uguagccCaaguuuggguccg....................  1      1  seq
......................................................................uguaCccaaaguuuggguccg....................  4      1  seq
......................................................................uguagccaaaguuugggCccg....................  5      1  seq
......................................................................uguagccGaaguuuggguccg....................  12     1  seq
......................................................................uguagccaaaguuuggguAcg....................  8      1  seq
......................................................................uguagccaaaguuuAgguccg....................  161    1  seq
......................................................................uguagccaaaguuugUguccg....................  5      1  seq
......................................................................uguagccaaaguuCggguccg....................  17     1  seq
......................................................................uguagccaGaguuuggguccg....................  14     1  seq
......................................................................uguagccaaaguuugggAccg....................  21     1  seq
......................................................................uCuagccaaaguuuggguccg....................  11     1  seq
......................................................................uguagccaaaAuuuggguccg....................  3      1  seq
......................................................................ugGagccaaaguuuggguccg....................  6      1  seq
......................................................................uguagccaaaguAuggguccg....................  6      1  seq
......................................................................uguagccaaaguuugAguccg....................  53     1  seq
......................................................................uguagccaaaguGuggguccg....................  6      1  seq
......................................................................uguagccaaaguuugggucAg....................  66     1  seq
......................................................................uguGgccaaaguuuggguccg....................  12     1  seq
......................................................................uguagccaaaguuuggUuccg....................  4      1  seq
......................................................................uguagccaaaguuuggguccA....................  131    1  seq
......................................................................uguagccaaaguuugggGccg....................  6      1  seq
......................................................................uguagccaaaguuGggguccg....................  4      1  seq
......................................................................uguagccaaaguCuggguccg....................  9      1  seq
......................................................................uguUgccaaaguuuggguccg....................  2      1  seq
......................................................................uguagccaaaguuuggCuccg....................  5      1  seq
......................................................................uguagccaUaguuuggguccg....................  3      1  seq
......................................................................uguagccaaaguuAggguccg....................  8      1  seq
......................................................................uguagccaaagGuuggguccg....................  4      1  seq
......................................................................ugAagccaaaguuuggguccg....................  75     1  seq
......................................................................uguagccaaaguuuggguccU....................  1649   1  seq
......................................................................uUuagccaaaguuuggguccg....................  9      1  seq
```

```
                              Star                                    Mature
gaucgggauuccauaugccaucggaggcgggcuuaaucuuuggcuaaaauagugaacacaauucaagccuauguagccaaaguuuggguccgucucuauggcuaccucaucg
.......................................................................uguagccaCaguuuggguccgu...................    1      1     seq
.......................................................................uguUgccaaaguuuggguccgu...................    1      1     seq
.......................................................................uguagccaaaguCuggguccgu...................    1      1     seq
.......................................................................uguagccaaaguuugggucUgu...................    4      1     seq
.......................................................................uguagccaaaguuuggguccgG...................    26     1     seq
.......................................................................Aguagccaaaguuuggguccgu...................    6      1     seq
.......................................................................uguagccaaaguGuggguccgu...................    1      1     seq
.......................................................................uguagccaaaguuugggAccgu...................    2      1     seq
.......................................................................uguagccaaaguuugggGccgu...................    1      1     seq
.......................................................................uguagccaaaguuuggguGcgu...................    1      1     seq
.......................................................................uguagccGaaguuuggguccgu...................    1      1     seq
.......................................................................ugAagccaaaguuuggguccgu...................    2      1     seq
.......................................................................uguagccaaaguuuggguccgu...................    3746   0     seq
.......................................................................uguagccaaGguuuggguccgu...................    1      1     seq
.......................................................................uguagccaaaguuuggguAcgu...................    4      1     seq
.......................................................................uguagccaaagAuuggguccgu...................    1      1     seq
.......................................................................uguagccaaaguuuggguccUu...................    32     1     seq
.......................................................................uguagccaaaguuugggucAgu...................    4      1     seq
.......................................................................Gguagccaaaguuuggguccgu...................    1      1     seq
.......................................................................uguagccaaaguuuggguccCu...................    2      1     seq
.......................................................................uguagcUaaaguuuggguccgu...................    1      1     seq
.......................................................................uguagccaaaguuugUguccgu...................    1      1     seq
.......................................................................uguagccUaaguuuggguccgu...................    1      1     seq
.......................................................................uguagccaaaguuuggguccgA...................    438    1     seq
.......................................................................uguagccaaaguuuAgguccgu...................    4      1     seq
.......................................................................uguagccaaaguuuggguccAu...................    2      1     seq
.......................................................................uguagccaaaguuugAguccgu...................    3      1     seq
.......................................................................uguagcAaaaguuuggguccgu...................    1      1     seq
.......................................................................uguagAcaaaguuuggguccgu...................    2      1     seq
.......................................................................uguagccaaaguuuggAuccgu...................    1      1     seq
.......................................................................uguagccaaaAuuuggguccgu...................    1      1     seq
.......................................................................Nguagccaaaguuuggguccgu...................    1      1     seq
.......................................................................uguagccaaaguuuggguccgC...................    72     1     seq
.......................................................................uguagccaaaguuuggguccguA..................    16     1     seq
.......................................................................uguagccaaaguuuggguccguU..................    460    1     seq
.......................................................................uguagccaaaguuuggguccguAu.................    7      1     seq
.......................................................................uguagccaaaguuuggguccguGu.................    2      1     seq
.......................................................................uguagccaaaguuuggguccguUu.................    113    1     seq
.......................................................................uguagccaaaguuuggguccgucu.................    1      0     seq
.......................................................................uguagccaaaguuuggguccgAcu.................    2      1     seq
........................................................................guaAccaaaguuuggguccg....................    2      1     seq
........................................................................guagccaaaguuuggguccg....................    26     0     seq
........................................................................guagccaaaguuuUgguccg....................    1      1     seq
........................................................................guagccaaaguuuAgguccg....................    1      1     seq
........................................................................guaUccaaaguuuggguccg....................    1      1     seq
........................................................................guagccaaaguuuggguccguU..................    8      1     seq
.........................................................................uagccaaaguuugggucc.....................    1      0     seq
.........................................................................uagccaaaguuuggguccg....................    4      0     seq
```
