# Supplementary material for: The Small RNA Universe of Capitella teleta
Source: Front Mol Biosci. 2022 Feb 25;9:802814. doi: 10.3389/fmolb.2022.802814 (PMC8915122; doi:10.3389/fmolb.2022.802814)
Supplement: Supplementary file 1 [file DataSheet1.ZIP › Supplement/homologRecovered/CAPTEscaffold_36111_47537.pdf]

Provisional ID	:	CAPTEscaffold_36111_47537
Score total	:	5.2
Score for star read(s)	:	-1.3
Score for read counts	:	0
Score for mfe	:	1.9
Score for randfold	:	1.6
Score for cons. seed	:	3
Total read count	:	42345
Mature read count	:	42307
Loop read count	:	2
Star read count	:	36

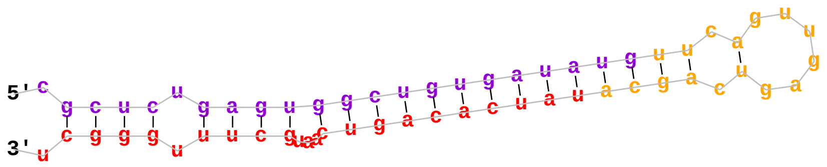

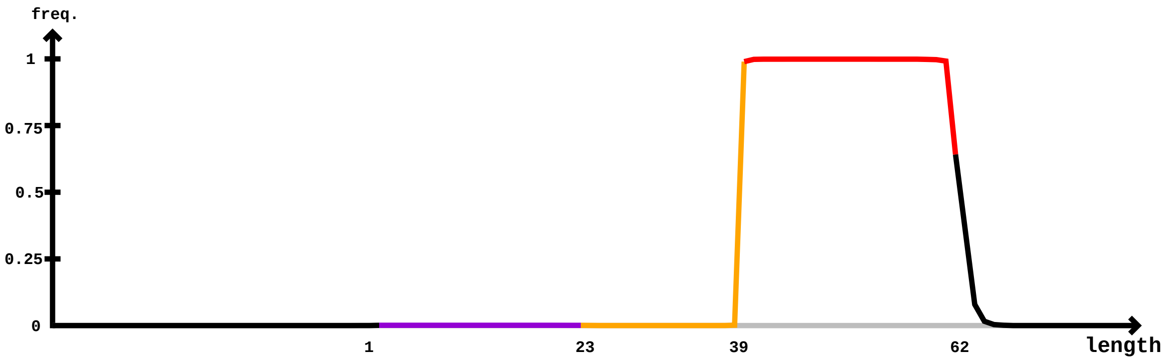

Star **Mature**

```
5'- uaauugaauugcuugcuuuugcgaauauucugcgcucugaguggcugugauauguucaguugagucagcauaucacagucaaugcuuugggcucagaauauucugcucucu -3'  obs
    uaauugaauugcuugcuuuugcgaauauucugcgcucugaguggcugugauauguucaguugagucagcauaucacagucaaugcuuugggcucagaauauucugcucucu       exp
    uaauugaauugcuugcuuuugcgaauauucugcgcucugaguggcugugauauguucaguugagucagcauaucacagucaaugcuuugggcucagaauauucugcucucu       known
    ...................((((((((((((.((((.((((((((((((((((((.(......).)))))))))))))))...)))).)))).))))))))).)).....    reads   mm   sample

    ..............................ugcgcucugaguggcugugau............................................................   3   0   seq
    ..............................ugcgcucugaguggcugugaua...........................................................   3   0   seq
    ................................cgcucugaguggcugugau............................................................   1   0   seq
    ................................cgcucugaguggcugugaua...........................................................   2   0   seq
    ................................cgcucugaguggcugugauau..........................................................   2   0   seq
    ................................cgcucugaguggUugugauaug.........................................................   1   1   seq
    ................................cgcucugaguggcugugauaug.........................................................   29  0   seq
    ................................cgcucugaguggcugugauauguu.......................................................   1   0   seq
    ....................................................uguucaguugagucagcaua.......................................   2   0   seq
    ......................................................uucaguugagucagcauaucacagucaaug...........................   1   0   seq
    ....................................................................cauaucacagucaaugcuuugggc...................   1   0   seq
    ....................................................................cauaucacagucaaugcuuugggcu..................   3   0   seq
    .....................................................................auaucacagucaaugcuuugggc...................   12  0   seq
    .....................................................................auaucacagucaaugcuuugggA...................   1   1   seq
    .....................................................................Cuaucacagucaaugcuuugggc...................   3   1   seq
    .....................................................................auaucacagucaaugcuuugAgcu..................   1   1   seq
    .....................................................................Guaucacagucaaugcuuugggcu..................   1   1   seq
    .....................................................................Cuaucacagucaaugcuuugggcu..................   3   1   seq
    .....................................................................auaucacagucaaugcuuugggcu..................   19  0   seq
    .....................................................................auaucacagucaaugcuuugggcuU.................   2   1   seq
    .....................................................................auaucacagucaaugcuuugggcuc.................   2   0   seq
    .....................................................................auaucacagucaaugcuuugggcucU................   5   1   seq
    .....................................................................auaucacagucaaugcuuugggcuca................   1   0   seq
    .....................................................................auaucacagucaaugcuuugggcucaAa..............   1   1   seq
    ......................................................................uaucacagucaaugcuuu.......................   1   0   seq
    ......................................................................uaucacagucaaugcuuug......................   36  0   seq
    ......................................................................uaucacagucaaugcuuuAg.....................   1   1   seq
    ......................................................................uaucacagucaaugcuuugg.....................   41  0   seq
    ......................................................................uaucacagucaaugcuuuggA....................   3   1   seq
    ......................................................................uaucacagucaaugcuuuggC....................   3   1   seq
    ......................................................................uaucacagucaaugcuuuggg....................   216 0   seq
```

```
                    Star                                  Mature
uaauugaauugcuugcuuuugcgaauauucugcgcucugaguggcugugauauguucaguugagucagcauaucacagucaaugcuuugggcucagaauauucugcucucu
......................................................................uaucacagucaaugcuuuAgg....................    1       1       seq
......................................................................uaucacagucaaAgcuuuggg....................    1       1       seq
......................................................................uaucacUgucaaugcuuugggc...................    2       1       seq
......................................................................uaucacagucaaugcuuugggA...................    13      1       seq
......................................................................uaucacagucaaugcuuAgggc...................    5       1       seq
......................................................................uauUacagucaaugcuuugggc...................    6       1       seq
......................................................................uaucacagucaaugGuuugggc...................    1       1       seq
......................................................................uaucacagucaaGgcuuugggc...................    1       1       seq
......................................................................uaucacagucaauCcuuugggc...................    3       1       seq
......................................................................uaucacagucaaAgcuuugggc...................    5       1       seq
......................................................................uaucacagucaaugcuuuggCc...................    2       1       seq
......................................................................uauAacagucaaugcuuugggc...................    3       1       seq
......................................................................uaucacagucaaugcuuuAggc...................    48      1       seq
......................................................................uaucacagCcaaugcuuugggc...................    1       1       seq
......................................................................uaucacagucaaugcAuugggc...................    4       1       seq
......................................................................uaCcacagucaaugcuuugggc...................    1       1       seq
......................................................................Naucacagucaaugcuuugggc...................    2       1       seq
......................................................................uaucUcagucaaugcuuugggc...................    1       1       seq
......................................................................uaucacagucaaugcuuuggUc...................    5       1       seq
......................................................................Gaucacagucaaugcuuugggc...................    9       1       seq
......................................................................Aaucacagucaaugcuuugggc...................    41      1       seq
......................................................................uaucacagucaauAcuuugggc...................    2       1       seq
......................................................................uaucacagAcaaugcuuugggc...................    2       1       seq
......................................................................uaucaUagucaaugcuuugggc...................    1       1       seq
......................................................................uaucacagucaaugcuuCgggc...................    1       1       seq
......................................................................uaucacagucaaugcuuugggc...................    14501   0       seq
......................................................................uaucacaguAaaugcuuugggc...................    1       1       seq
......................................................................uUucacagucaaugcuuugggc...................    2       1       seq
......................................................................uaucacagucaaugcuuuggAc...................    1       1       seq
......................................................................uaucacaguGaaugcuuugggc...................    1       1       seq
......................................................................uaucacagucaaugcGuugggc...................    1       1       seq
......................................................................uaucacagucaaugcuuuCggc...................    1       1       seq
......................................................................uaucacagucaaugcuAugggc...................    3       1       seq
......................................................................uaucacagucaaugcuuugUgc...................    1       1       seq
......................................................................uaucacagucaaugcuGugggc...................    1       1       seq
......................................................................uaucacagucGaugcuuugggc...................    4       1       seq
......................................................................uaucacagucaaCgcuuugggc...................    2       1       seq
......................................................................uaucaAagucaaugcuuugggc...................    8       1       seq
......................................................................uaucacagucaaugcuuugggU...................    8       1       seq
......................................................................Caucacagucaaugcuuugggc...................    4       1       seq
......................................................................uaucacaguUaaugcuuugggc...................    8       1       seq
......................................................................uaucacagucaaugcuCugggc...................    4       1       seq
......................................................................uaucacagucaGugcuuugggc...................    3       1       seq
......................................................................uaucacagucaaugUuuugggc...................    2       1       seq
......................................................................uaucacGgucaaugcuuugggc...................    3       1       seq
......................................................................uaucacagucaaugcuuugAgc...................    15      1       seq
......................................................................uaucacagucaaugcCuugggc...................    7       1       seq
......................................................................uaucacagucaaugAuuugggc...................    6       1       seq
......................................................................uaucGcagucaaugcuuugggc...................    6       1       seq
......................................................................uaAcacagucaaugcuuugggc...................    15      1       seq
......................................................................uaucacagucaaugcuuGgggc...................    1       1       seq
......................................................................uaucacaCucaaugcuuugggc...................    2       1       seq
......................................................................uGucacagucaaugcuuugggc...................    1       1       seq
......................................................................uaucacagucaaugcuuugUgcu..................    3       1       seq
......................................................................uaucacUgucaaugcuuugggcu..................    6       1       seq
......................................................................uaucacagucaaugcuuugAgcu..................    15      1       seq
......................................................................uaucacagucaaugcuCugggcu..................    4       1       seq
......................................................................uaucacagucaaugcuuGgggcu..................    3       1       seq
......................................................................uaucacagucaauUcuuugggcu..................    1       1       seq
......................................................................uaucacagucaaugcuuugggcA..................    64      1       seq
......................................................................uaucacagAcaaugcuuugggcu..................    6       1       seq
......................................................................uauGacagucaaugcuuugggcu..................    4       1       seq
......................................................................uaucacagucaaugcuuuCggcu..................    1       1       seq
......................................................................uaucaUagucaaugcuuugggcu..................    3       1       seq
......................................................................uaucacagucaauAcuuugggcu..................    3       1       seq
......................................................................uaucacagucaaCgcuuugggcu..................    2       1       seq
......................................................................uaucacagucaaugcuAugggcu..................    4       1       seq
......................................................................uauAacagucaaugcuuugggcu..................    1       1       seq
......................................................................uaucacaguGaaugcuuugggcu..................    3       1       seq
......................................................................uaucacagucaaugcuuCgggcu..................    3       1       seq
```

```
                                Star                                      Mature
uaauugaauugcuugcuuuugcgaauauucugcgcucugaguggcugugauauguucaguugagucagcauaucacagucaaugcuuugggcucagaauauucugcucucu
......................................................................uaucacagucaaugcuuugggAu..................    5        1        seq
......................................................................uaucacagucaaugcuGugggcu..................    1        1        seq
......................................................................uaucacagGcaaugcuuugggcu..................    1        1        seq
......................................................................uaucacagucaaugcuuuggUcu..................    1        1        seq
......................................................................uaCcacagucaaugcuuugggcu..................    3        1        seq
......................................................................uaAcacagucaaugcuuugggcu..................    20       1        seq
......................................................................uaucacagucaaugcAuugggcu..................    13       1        seq
......................................................................uaucUcagucaaugcuuugggcu..................    5        1        seq
......................................................................uaucacagucaaugcuuAgggcu..................    8        1        seq
......................................................................Naucacagucaaugcuuugggcu..................    8        1        seq
......................................................................Aaucacagucaaugcuuugggcu..................    77       1        seq
......................................................................uaucacaguUaaugcuuugggcu..................    7        1        seq
......................................................................uaucacCgucaaugcuuugggcu..................    2        1        seq
......................................................................uaucacagucaaugcuuugggcG..................    72       1        seq
......................................................................uaucacagucaGugcuuugggcu..................    5        1        seq
......................................................................uaucacaAucaaugcuuugggcu..................    5        1        seq
......................................................................uaucacaUucaaugcuuugggcu..................    2        1        seq
......................................................................uauUacagucaaugcuuugggcu..................    12       1        seq
......................................................................uaucacagCcaaugcuuugggcu..................    3        1        seq
......................................................................uaucacagucaaugAuuugggcu..................    6        1        seq
......................................................................uNucacagucaaugcuuugggcu..................    1        1        seq
......................................................................uaucacagucaUugcuuugggcu..................    2        1        seq
......................................................................uaucacagucaaugGuuugggcu..................    1        1        seq
......................................................................uaucacGgucaaugcuuugggcu..................    6        1        seq
......................................................................uaucacagucaaugcuuugCgcu..................    1        1        seq
......................................................................uaucaAagucaaugcuuugggcu..................    5        1        seq
......................................................................uaucacagucaaugcCuugggcu..................    10       1        seq
......................................................................uaucacagucGaugcuuugggcu..................    4        1        seq
......................................................................Caucacagucaaugcuuugggcu..................    5        1        seq
......................................................................uaucacagucaaugcuuugggcC..................    4        1        seq
......................................................................uaucacagucaaugcGuugggcu..................    1        1        seq
......................................................................uaucacagucaaugcuuuUggcu..................    2        1        seq
......................................................................uaucacagucaaugcuuuAggcu..................    83       1        seq
......................................................................uGucacagucaaugcuuugggcu..................    2        1        seq
......................................................................uaucacagucaaGgcuuugggcu..................    1        1        seq
......................................................................uaucacagucaaugcuuuggAcu..................    3        1        seq
......................................................................uaucacagucaauCcuuugggcu..................    1        1        seq
......................................................................uaucaGagucaaugcuuugggcu..................    2        1        seq
......................................................................uaucacagucUaugcuuugggcu..................    1        1        seq
......................................................................Gaucacagucaaugcuuugggcu..................    9        1        seq
......................................................................uaucacagucaaAgcuuugggcu..................    8        1        seq
......................................................................uaucacagucaaugcuuugggcu..................    19686    0        seq
......................................................................uaucGcagucaaugcuuugggcu..................    4        1        seq
......................................................................uaucacaguNaaugcuuugggcu..................    1        1        seq
......................................................................uaucacaguAaaugcuuugggcu..................    9        1        seq
......................................................................uaucacagucaaugUuuugggcu..................    1        1        seq
......................................................................uaucacaCucaaugcuuugggcu..................    3        1        seq
......................................................................uaucacagucaaugcuuugggcAc.................    1        1        seq
......................................................................uaucacagucaaugAuuugggcuc.................    3        1        seq
......................................................................uaucacaguAaaugcuuugggcuc.................    1        1        seq
......................................................................uaucacagucaaAgcuuugggcuc.................    4        1        seq
......................................................................uaucacaguUaaugcuuugggcuc.................    2        1        seq
......................................................................uaucacagucaaugcAuugggcuc.................    1        1        seq
......................................................................Caucacagucaaugcuuugggcuc.................    1        1        seq
......................................................................uaucacagucaaugcuuugggUuc.................    1        1        seq
......................................................................uauUacagucaaugcuuugggcuc.................    2        1        seq
......................................................................uaucacagucaaugcuuAgggcuc.................    2        1        seq
......................................................................uaucacUgucaaugcuuugggcuc.................    1        1        seq
......................................................................uaucacagucaaugcuuuAggcuc.................    10       1        seq
......................................................................uaucacagucaaugcGuugggcuc.................    1        1        seq
......................................................................Aaucacagucaaugcuuugggcuc.................    13       1        seq
......................................................................uaucacagucaaugUuuugggcuc.................    1        1        seq
......................................................................uaucacagucaaugcuuCgggcuc.................    2        1        seq
......................................................................uaucacagucaauUcuuugggcuc.................    2        1        seq
......................................................................uaucacagucaaugcuuugggAuc.................    1        1        seq
......................................................................uaucacagucaaugcuuugAgcuc.................    3        1        seq
......................................................................uaucacagucaaugcuAugggcuc.................    2        1        seq
......................................................................uaucUcagucaaugcuuugggcuc.................    1        1        seq
......................................................................uaucacagucaaugcuuugggcuG.................    1        1        seq
......................................................................uaucacGgucaaugcuuugggcuc.................    3        1        seq
```

Star **Mature**

uaauugaauugcuugcuuuugcgaauauucugcgcucugaguggcugugauauguucaguugagucagcauaucacagucaaugcuuugggcucagaauauucugcucucu

....uaucacagucaaugcuuugggcuA.................	11	1	seq
....uaucGcagucaaugcuuugggcuc.................	1	1	seq
....uaucacagucaaugcuuugggcuU.................	477	1	seq
....Naucacagucaaugcuuugggcuc.................	1	1	seq
....uaucacagucaaugcuuugggcuc.................	2701	0	seq
....uaAcacagucaaugcuuugggcuc.................	1	1	seq
....uaucacagAcaaugcuuugggcuc.................	1	1	seq
....uauUacagucaaugcuuugggcuca................	1	1	seq
....uaAcacagucaaugcuuugggcuca................	2	1	seq
....Aaucacagucaaugcuuugggcuca................	3	1	seq
....uaucGcagucaaugcuuugggcuca................	1	1	seq
....uauAacagucaaugcuuugggcuca................	1	1	seq
....uaucacagucaaugcCuugggcuca................	1	1	seq
....uaucacagucaauAcuuugggcuca................	1	1	seq
....uaucacagAcaaugcuuugggcuca................	1	1	seq
....uaucacagucaaugcuuugggcucC................	14	1	seq
....uaucacagucaaugcuuuggUcuca................	1	1	seq
....uaucacagucaaugcuuuggCcuca................	1	1	seq
....uaucacagucaaugcuuugggcucG................	7	1	seq
....uaucacagucaaugcuuugggcucU................	1232	1	seq
....uaucacagucaaugcuuuAggcuca................	4	1	seq
....uUucacagucaaugcuuugggcuca................	1	1	seq
....uaucaUagucaaugcuuugggcuca................	1	1	seq
....uaucacagucaaugcAuugggcuca................	2	1	seq
....uaucacagucaaugcuuCgggcuca................	1	1	seq
....uaucacagucaaugAuuugggcuca................	1	1	seq
....uaucacagucaaugcuuugggcuUa................	5	1	seq
....Naucacagucaaugcuuugggcuca................	1	1	seq
....uaucacagucaaugcuuugAgcuca................	1	1	seq
....uaucacagucaaugcuuugggcuca................	1344	0	seq
....uaucacagucaaugcuuugggcucag...............	2	0	seq
....uaucacagucaaugcuuugggcucaA...............	47	1	seq
....uaucacagucaaugcuuugggcucaC...............	11	1	seq
....uaucacagucaaugcuuugggcucaU...............	460	1	seq
....uaucacagucaaugcuuugggcucagU..............	1	1	seq
....uaucacagucaaugcuuugggcucaUa..............	4	1	seq
....uaucacagucaaugcuuugggcucaCa..............	1	1	seq
....uaucacagucaaugcuuugggcucaAa..............	91	1	seq
....uaucacagucaaugcuuugggcucaAaa.............	50	1	seq
....uaucacagucaaugcuuugggcucaAaau............	2	1	seq
....Uucacagucaaugcuuugggc...................	1	1	seq
....aucacagucaaugcuuugggc...................	50	0	seq
....aucacagucaaugcuuugggcA..................	2	1	seq
....aucacagucaaugcuuugggcu..................	213	0	seq
....aucacaCucaaugcuuugggcu..................	1	1	seq
....Gucacagucaaugcuuugggcu..................	2	1	seq
....aucacagucaaugcuuugggcuc.................	45	0	seq
....aucacagucaaugcuuugggcuU.................	8	1	seq
....aucacagucaaugcuuugggcucU................	13	1	seq
....aucacagucaaugcuuugggcuca................	19	0	seq
....aucacagucaaugcuuugggcucaU...............	3	1	seq
....aucacagucaaugcuuugggcucaA...............	1	1	seq
....ucacagucaaugcuuugggc...................	1	0	seq
....ucacagucaaugcuuugggcu..................	14	0	seq
....ucacagucaaugcuuugggcuc.................	5	0	seq
....ucacagucaaugcuuugggcucU................	1	1	seq
....ucacagucaaugcuuugggcuca................	3	0	seq
....ucacagucaaugcuuugggcucag...............	3	0	seq
....ucacagucaaugcuuugggcucaga..............	4	0	seq
....ucacagucaaugcuuugggcucagU..............	1	1	seq
....acagucaaugcuuugggcu..................	1	0	seq
....acagucaaugcuuugggcuc.................	1	0	seq
....cagucaaugcuuugggcuca................	2	0	seq
