# Supplementary material for: The Small RNA Universe of Capitella teleta
Source: Front Mol Biosci. 2022 Feb 25;9:802814. doi: 10.3389/fmolb.2022.802814 (PMC8915122; doi:10.3389/fmolb.2022.802814)
Supplement: Supplementary file 1 [file DataSheet1.ZIP › Supplement/homologRecovered/CAPTEscaffold_413_21046.pdf]

Provisional ID	:	CAPTEscaffold_413_21046
Score total	:	230.1
Score for star read(s)	:	3.9
Score for read counts	:	219.9
Score for mfe	:	1.8
Score for randfold	:	1.6
Score for cons. seed	:	3
Total read count	:	443
Mature read count	:	441
Loop read count	:	0
Star read count	:	2

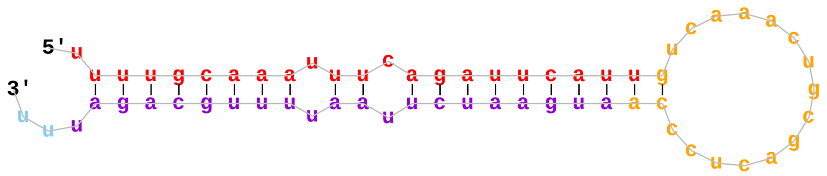

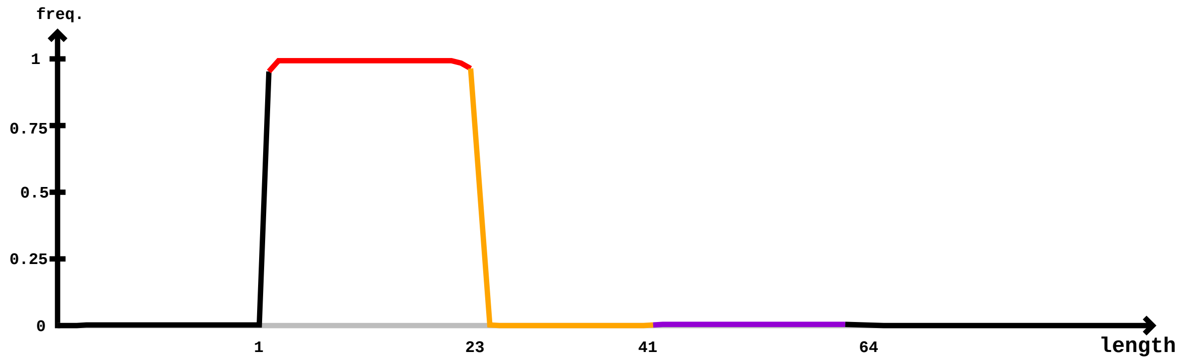

Mature **Star**

```
5'- agucuaugcagggguucugauuuugcaaauuucagauucauugucaaacugcgacucccaaugaaucuuaauuuugcagauuuaggacccguggcuguucaucgcaugccgg  -3'   obs
    agucuaugcagggguucugauuuugcaaauuucagauucauugucaaacugcgacucccaaugaaucuuaauuuugcagauuuaggacccguggcuguucaucgcaugccgg         exp
    ...........(((((((((.((((((((.((.((((((((((...............)))))))))).)).)))))))).))))))))).(((((((.....))).)))).   reads   mm   sample
    .gucuaugcagggguucuga............................................................................................   1       0    seq
    ....................uuuugcaaauuucagauuca........................................................................   4       0    seq
    ....................Auuugcaaauuucagauucau.......................................................................   1       1    seq
    ....................uuuugcaaauuucagauucau.......................................................................   8       0    seq
    ....................uuuugcaaauuucagauucauu......................................................................   405     0    seq
    ....................Auuugcaaauuucagauucauu......................................................................   2       1    seq
    ....................uuuugcaaauuucaAauucauu......................................................................   1       1    seq
    ....................uuuugcaaauuucGgauucauu......................................................................   1       1    seq
    ....................uuuugcaaauuucagauucauug.....................................................................   1       0    seq
    .....................uuugcaaauuucagauucauu......................................................................   4       0    seq
    .....................uuugcaaauuucagauuUauug.....................................................................   1       1    seq
    .....................uuugcaaauuucagauucauug.....................................................................   12      0    seq
    .....................uuugcaaauuucagauucauugu....................................................................   1       0    seq
    ............................................................augaaucuuaauuuugcagau...............................   1       0    seq
    .............................................................ugaaucuuaauuuugcagauuu.............................   1       0    seq
```
