# Supplementary material for: The Small RNA Universe of Capitella teleta
Source: Front Mol Biosci. 2022 Feb 25;9:802814. doi: 10.3389/fmolb.2022.802814 (PMC8915122; doi:10.3389/fmolb.2022.802814)
Supplement: Supplementary file 1 [file DataSheet1.ZIP › Supplement/homologRecovered/CAPTEscaffold_154_12127.pdf]

Provisional ID	:	CAPTEscaffold_154_12127
Score total	:	22503
Score for star read(s)	:	3.9
Score for read counts	:	22492.6
Score for mfe	:	1.9
Score for randfold	:	1.6
Score for cons. seed	:	3
Total read count	:	44130
Mature read count	:	44128
Loop read count	:	1
Star read count	:	1

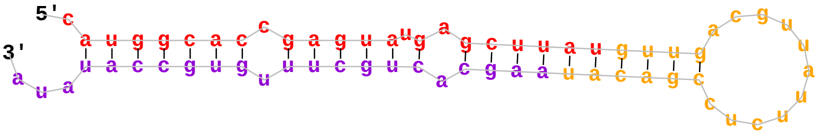

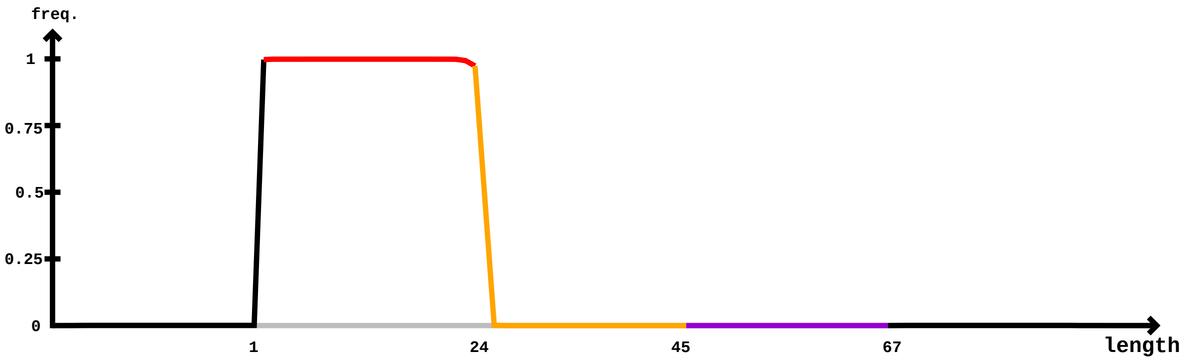

Mature **Star**

```
5'- gaaaucggccgucguacguccauggcaccgaguaugagcuuauguugacguuauucuccgacauaagcacugcuuugugccauauacguaugccaggcaaaucacagcaaagg  -3'   obs
    gaaaucggccgucguacguccauggcaccgaguaugagcuuauguugacguuauucuccgacauaagcacugcuuugugccauauacguaugccaggcaaaucacagcaaagg        exp
    .......(((..(((((((..(((((((.(((((.(.((((((((((..........)))))))))).)))))).)))))))..)))))))...)))................  reads   mm   sample
    Aaaaucggccgucguacguc.............................................................................................  1       1    seq
    gaaaucggccgucguacguc.............................................................................................  4       0    seq
    .aaaucggccgucguacguc.............................................................................................  12      0    seq
    ..aaucggccgucguacguc.............................................................................................  9       0    seq
    ...................ccauggcaccgaguaugagcuuau......................................................................  1       0    seq
    ....................cauggcaccgaguaugag...........................................................................  2       0    seq
    ....................cauggcaccgaguaugagc..........................................................................  2       0    seq
    ....................cauggcaccgaguaugagcu.........................................................................  7       0    seq
    ....................cauggcaccgaguaugagcuG........................................................................  1       1    seq
    ....................cauggcaccgaguaugagcuu........................................................................  219     0    seq
    ....................cauggcaccgaguauAagcuu........................................................................  1       1    seq
    ....................cauggcaccAaguaugagcuua.......................................................................  8       1    seq
    ....................Uauggcaccgaguaugagcuua.......................................................................  2       1    seq
    ....................cUuggcaccgaguaugagcuua.......................................................................  1       1    seq
    ....................cauggcaccgaguauAagcuua.......................................................................  5       1    seq
    ....................cauggcaccgaguaugagcuua.......................................................................  863     0    seq
    ....................cauggcaccgaguaugagcuCa.......................................................................  1       1    seq
    ....................cauggcaccgaguaAgagcuuau......................................................................  12      1    seq
    ....................cauggcaccgaguaugagcuGau......................................................................  5       1    seq
    ....................cauggcaccgaguaugaAcuuau......................................................................  1       1    seq
    ....................cauggcaccgaguGugagcuuau......................................................................  3       1    seq
    ....................cauggcaccgGguaugagcuuau......................................................................  9       1    seq
    ....................Aauggcaccgaguaugagcuuau......................................................................  16      1    seq
    ....................cauggcaccgaguaCgagcuuau......................................................................  2       1    seq
    ....................cauggcaccgaguaugagcuCau......................................................................  19      1    seq
    ....................cauggGaccgaguaugagcuuau......................................................................  2       1    seq
    ....................cauggcaccAaguaugagcuuau......................................................................  322     1    seq
    ....................cauAgcaccgaguaugagcuuau......................................................................  47      1    seq
    ....................cauggcaccgaguaugaUcuuau......................................................................  4       1    seq
    ....................cauggcaccgaguaugagcuuGu......................................................................  9       1    seq
    ....................cauggcaccgaguaugagGuuau......................................................................  3       1    seq
    ....................cauggcaccgaguaugagAuuau......................................................................  7       1    seq
    ....................cauggcaccgaguaugagcGuau......................................................................  7       1    seq
```

Mature Star

```
gaaaucggccgucguacguccauggcaccgaguaugagcuuauguugacguuauucuccgacauaagcacugcuuugugccauauacguaugccaggcaaaucacagcaaagg
....................cauggcaccgagAaugagcuuau......................................................................	4	1	seq
....................cauggcaccgaguaugCgcuuau......................................................................	3	1	seq
....................caugAcaccgaguaugagcuuau......................................................................	9	1	seq
....................caAggcaccgaguaugagcuuau......................................................................	33	1	seq
....................cauggcaAcgaguaugagcuuau......................................................................	4	1	seq
....................caGggcaccgaguaugagcuuau......................................................................	2	1	seq
....................cauggcaccgaguUugagcuuau......................................................................	3	1	seq
....................cauggcaccCaguaugagcuuau......................................................................	4	1	seq
....................cauggcaUcgaguaugagcuuau......................................................................	4	1	seq
....................cauggAaccgaguaugagcuuau......................................................................	5	1	seq
....................Gauggcaccgaguaugagcuuau......................................................................	3	1	seq
....................cauggcaGcgaguaugagcuuau......................................................................	3	1	seq
....................cauggcaccgaAuaugagcuuau......................................................................	4	1	seq
....................cauggcaccgaguaugagUuuau......................................................................	5	1	seq
....................cauggcaccgaguaugagcuuaA......................................................................	37	1	seq
....................cauUgcaccgaguaugagcuuau......................................................................	5	1	seq
....................cauggcaccgaguauUagcuuau......................................................................	2	1	seq
....................cauggcaccgaguaugagcCuau......................................................................	4	1	seq
....................cauggcaccgaguaugagcuuCu......................................................................	2	1	seq
....................caCggcaccgaguaugagcuuau......................................................................	5	1	seq
....................cauggcCccgaguaugagcuuau......................................................................	1	1	seq
....................cauggcaccgaguCugagcuuau......................................................................	1	1	seq
....................cauggcaccgaguaugagcAuau......................................................................	9	1	seq
....................cauggcaccgaUuaugagcuuau......................................................................	3	1	seq
....................cauggcaccgaCuaugagcuuau......................................................................	3	1	seq
....................cauggcaccgaguaugGgcuuau......................................................................	5	1	seq
....................cGuggcaccgaguaugagcuuau......................................................................	5	1	seq
....................cauggcaccgaguauCagcuuau......................................................................	3	1	seq
....................cauggcacGgaguaugagcuuau......................................................................	1	1	seq
....................cauggcaccgaguaugUgcuuau......................................................................	7	1	seq
....................cauggcaccgaguaugagcuuUu......................................................................	4	1	seq
....................cauggcaccgagGaugagcuuau......................................................................	4	1	seq
....................cauggcaccgaguaGgagcuuau......................................................................	6	1	seq
....................cauggcaccgaguaugagcuAau......................................................................	14	1	seq
....................cauggcaccUaguaugagcuuau......................................................................	2	1	seq
....................cauggcaccgaguaugagcuuaG......................................................................	2	1	seq
....................cauggcaccgaguauAagcuuau......................................................................	129	1	seq
....................cauggUaccgaguaugagcuuau......................................................................	11	1	seq
....................cauggcacUgaguaugagcuuau......................................................................	37	1	seq
....................cauggcaccgagCaugagcuuau......................................................................	5	1	seq
....................cauCgcaccgaguaugagcuuau......................................................................	5	1	seq
....................cauggcaccgaguaugaCcuuau......................................................................	3	1	seq
....................caugUcaccgaguaugagcuuau......................................................................	2	1	seq
....................caugCcaccgaguaugagcuuau......................................................................	2	1	seq
....................cauggcaccgaguaugagcuuau......................................................................	41295	0	seq
....................cauggcacAgaguaugagcuuau......................................................................	11	1	seq
....................Nauggcaccgaguaugagcuuau......................................................................	11	1	seq
....................Uauggcaccgaguaugagcuuau......................................................................	87	1	seq
....................cauggcUccgaguaugagcuuau......................................................................	2	1	seq
....................cauggcGccgaguaugagcuuau......................................................................	14	1	seq
....................cauggcaccgaguaugagcuuaC......................................................................	6	1	seq
....................cauggcaccgUguaugagcuuau......................................................................	3	1	seq
....................cauggcaccgaguaugagcuuauC.....................................................................	2	1	seq
....................cauggcaccgaguauAagcuuaug.....................................................................	1	1	seq
....................cauggcaccgaguaugagcuuauU.....................................................................	110	1	seq
....................cauggcaccgaguaugagcuuauA.....................................................................	192	1	seq
....................Uauggcaccgaguaugagcuuaug.....................................................................	1	1	seq
....................cauggcaccgaguaugagcuuaug.....................................................................	309	0	seq
....................cauggcaccgaguaugagcuGaug.....................................................................	1	1	seq
....................cauggcaccAaguaugagcuuaug.....................................................................	1	1	seq
....................cauggcaccgaguaugagcuuauUu....................................................................	8	1	seq
....................cauggcaccgaguaugagcuuauCu....................................................................	1	1	seq
....................cauggcaccgaguaugagcuuaugA....................................................................	6	1	seq
....................cauggcaccgaguaugagcuuauAu....................................................................	4	1	seq
....................cauggcaccgaguaugagcuuaugu....................................................................	7	0	seq
....................cauggcaccgaguaugagcuuauUuu...................................................................	2	1	seq
.....................auggcaccAaguaugagcuuau......................................................................	1	1	seq
.....................auggcaccgaguaugagcuuau......................................................................	62	0	seq
.....................auggcaccgaguaugagcuuaug.....................................................................	1	0	seq
.........................caccgaguaugagcuuau......................................................................	4	0	seq
```

```
                    Mature                                          Star
gaaaucggccgucguacgucCAUGGCACCGAGUAUGAGCUUAUguugacguuauucuccgacauAAGCACUGCUUUGUGCCAUAUAcguaugccaggcaaaucacagcaaagg
............................cgaguaugagcuuauguugac................................................................  1   0   seq
............................................uugacguuauucuccgaca..................................................  1   0   seq
................................................................aagcacugcuuugugccauaua...........................  1   0   seq
......................................................................................cguaugccaggcaaaucac........  15  0   seq
......................................................................................cguaugccaggcaaaucaca.......  1   0   seq
......................................................................................cguaugccaggcaaaucacag......  1   0   seq
......................................................................................cguaugccaggcaaaucacagc.....  4   0   seq
```
