# Supplementary material for: The Small RNA Universe of Capitella teleta
Source: Front Mol Biosci. 2022 Feb 25;9:802814. doi: 10.3389/fmolb.2022.802814 (PMC8915122; doi:10.3389/fmolb.2022.802814)
Supplement: Supplementary file 1 [file DataSheet1.ZIP › Supplement/homologRecovered/CAPTEscaffold_14_1884.pdf]

| | | |
|---|---|---|
| Provisional ID | : | CAPTEscaffold_14_1884 |
| Score total | : | 2463888.7 |
| Score for star read(s) | : | 3.9 |
| Score for read counts | : | 2463878.4 |
| Score for mfe | : | 1.9 |
| Score for randfold | : | 1.6 |
| Score for cons. seed | : | 3 |
| Total read count | : | 4832803 |
| Mature read count | : | 4832233 |
| Loop read count | : | 101 |
| Star read count | : | 469 |

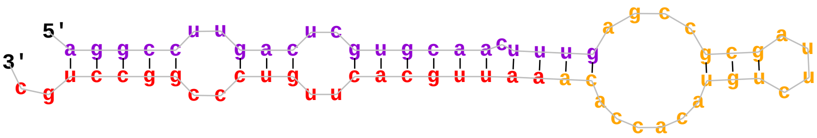


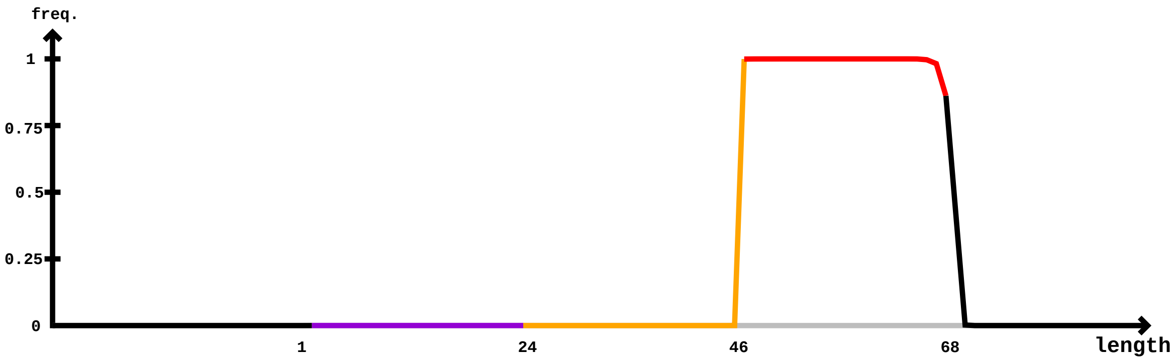


```
                      Star                                         Mature
5'- gagaugacugcacuugacuacaaguaggccuugacucgugcaacuuugagccgcgauucuguacaccacaaauugcacuugucccggccugccuguggucaugccucgcagc  -3'  obs
    gagaugacugcacuugacuacaaguaggccuugacucgugcaacuuugagccgcgauucuguacaccacaaauugcacuugucccggccugccuguggucaugccucgcagc       exp
    gagaugacugcacuugacuacaaguaggccuugacucgugcaacuuugagccgcgauucuguacaccacaaauugcacuugucccggccugccuguggucaugccucgcagc       known
    .......((((...((((((((.(((((((..(((..((((((.((((....(((....)))......))))))))))..)))..))))))).)))))))).......))))).   reads   mm   sample

    ......................aguaggccuugacucgugcaac....................................................................   3     0    seq
    ........................uaggccuugacucgugcaac....................................................................   1     0    seq
    ........................uaggccuugacucgugcaacu...................................................................   3     0    seq
    ........................uaggccuugacucgugcaacuu..................................................................   4     0    seq
    .........................aggccuuCacucgugcaacu...................................................................   1     1    seq
    .........................Gggccuugacucgugcaacu...................................................................   2     1    seq
    .........................aggccuugacuAgugcaacu...................................................................   1     1    seq
    .........................aggccuugacucgugcaacG...................................................................   2     1    seq
    .........................Uggccuugacucgugcaacu...................................................................   1     1    seq
    .........................aggccuugacucguCcaacu...................................................................   1     1    seq
    .........................aggccuugacucgugcaacu...................................................................   125   0    seq
    .........................aggccuugacucgugcaacuu..................................................................   3     0    seq
    .........................aggccuugacucgugcaacuuu.................................................................   12    0    seq
    .........................aggccuugacucgugcaacuuug................................................................   154   0    seq
    .........................aggccuugacuUgugcaacuuug................................................................   1     1    seq
    .........................aggccuugacucgugcGacuuug................................................................   1     1    seq
    .........................Gggccuugacucgugcaacuuug................................................................   1     1    seq
    .........................aggccuugacucgugcaacuuuU................................................................   1     1    seq
    .........................aggccuugacucgugcaacuAug................................................................   2     1    seq
    .........................aggccuugacucgugcaacuuugU...............................................................   77    1    seq
    .........................aggccuugacucgugcaacuuuga...............................................................   62    0    seq
    .........................aggccuugacucgugcaacuuugaA..............................................................   7     1    seq
    ...........................gccuugacucgugcaacuuuga...............................................................   7     0    seq
    ................................................Ggccgcgauucuguacaccaca..........................................   2     1    seq
    ................................................agccgcgauucuguGcaccaca..........................................   1     1    seq
    ................................................agccgcgauucuguacaccaca..........................................   98    0    seq
    .....................................................................aaauugcacuugucccggccu......................   17    0    seq
    .....................................................................aaauugcacuugucccggccug.....................   8     0    seq
    .....................................................................aaUuugcacuugucccggccug.....................   1     1    seq
    .....................................................................aaUuugcacuugucccggccugc....................   3     1    seq
    .....................................................................aaauugcacuugucccggccugU....................   1     1    seq
```

**Star** **Mature**

```
gagaugacugcacuugacuacaaguaggccuugacucgugcaacuuugagccgcgauucuguacaccacaaauugcacuugucccggccugccuggugucaugccucgcagc
.....................................................................aaauugcacuugucccggccugA....................	5	1	seq
.....................................................................aaauugcacuugucccggccugc....................	466	0	seq
......................................................................aauugcacuAgucccggc........................	4	1	seq
......................................................................aauAgcacuugucccggc........................	1	1	seq
......................................................................aauugcacuugucccggc........................	622	0	seq
......................................................................Gauugcacuugucccggc........................	2	1	seq
......................................................................aauugcacuugucccUgc........................	2	1	seq
......................................................................aauugcacuugucccggA........................	18	1	seq
......................................................................aauugUacuugucccggc........................	2	1	seq
......................................................................aauugcaAuugucccggc........................	1	1	seq
......................................................................Gauugcacuugucccggcc.......................	12	1	seq
......................................................................aauugcacuuguccGggcc.......................	1	1	seq
......................................................................aauugcaAuugucccggcc.......................	2	1	seq
......................................................................aauugcacuugucccggAc.......................	2	1	seq
......................................................................aauugcacuugucccggcA.......................	4	1	seq
......................................................................aauugcacuuguAccggcc.......................	3	1	seq
......................................................................aauugcacuugucccUgcc.......................	1	1	seq
......................................................................Nauugcacuugucccggcc.......................	2	1	seq
......................................................................aGuugcacuugucccggcc.......................	2	1	seq
......................................................................aauugcacuuguccAggcc.......................	4	1	seq
......................................................................aauugcacuugAcccggcc.......................	5	1	seq
......................................................................aaCugcacuugucccggcc.......................	1	1	seq
......................................................................aUuugcacuugucccggcc.......................	2	1	seq
......................................................................aauuUcacuugucccggcc.......................	1	1	seq
......................................................................aauugcacuugucccggUc.......................	1	1	seq
......................................................................aauugcacuuguUccggcc.......................	4	1	seq
......................................................................aauugcacuCgucccggcc.......................	1	1	seq
......................................................................aauCgcacuugucccggcc.......................	1	1	seq
......................................................................Uauugcacuugucccggcc.......................	86	1	seq
......................................................................aauugcUcuugucccggcc.......................	1	1	seq
......................................................................aauAgcacuugucccggcc.......................	3	1	seq
......................................................................aauugcacuugucccAgcc.......................	7	1	seq
......................................................................aauugAacuugucccggcc.......................	8	1	seq
......................................................................aauugcacuugucAcggcc.......................	11	1	seq
......................................................................aauugcacuugucccgAcc.......................	1	1	seq
......................................................................aauugcaGuugucccggcc.......................	1	1	seq
......................................................................aauugcacuuguccUggcc.......................	6	1	seq
......................................................................aaAugcacuugucccggcc.......................	15	1	seq
......................................................................aauugUacuugucccggcc.......................	2	1	seq
......................................................................aauugcacuuAucccggcc.......................	1	1	seq
......................................................................aauugcacuugucccggcG.......................	2	1	seq
......................................................................aauugcacuugucccggGc.......................	2	1	seq
......................................................................aauuAcacuugucccggcc.......................	1	1	seq
......................................................................aauugcacCugucccggcc.......................	1	1	seq
......................................................................aauugcacuuUucccggcc.......................	1	1	seq
......................................................................aauugcacAugucccggcc.......................	3	1	seq
......................................................................aauugcacGugucccggcc.......................	2	1	seq
......................................................................aauugcacuugucccggcc.......................	13915	0	seq
......................................................................aauugcacuuCucccggcc.......................	1	1	seq
......................................................................aauugcacuAgucccggcc.......................	16	1	seq
......................................................................aauugcacuugucccggcU.......................	26	1	seq
......................................................................aauugcacuugucUcggcc.......................	9	1	seq
......................................................................aauugcacuugucccgUcc.......................	1	1	seq
......................................................................aauugcGcuugucccggcc.......................	5	1	seq
......................................................................aauugcacuugCcccggcc.......................	1	1	seq
......................................................................aauugcacuugucccgCcc.......................	2	1	seq
......................................................................aaAugcacuugucccggccu......................	80	1	seq
......................................................................aauugcacCugucccggccu......................	3	1	seq
......................................................................aauugcacuuguccUggccu......................	57	1	seq
......................................................................Uauugcacuugucccggccu......................	3	1	seq
......................................................................aauugcacuugucAcggccu......................	32	1	seq
......................................................................aauugcacGugucccggccu......................	5	1	seq
......................................................................aGuugcacuugucccggccu......................	2	1	seq
......................................................................aauugcacuugucccggcUu......................	13	1	seq
......................................................................aauugcaAuugucccggccu......................	7	1	seq
......................................................................aauugcGcuugucccggccu......................	21	1	seq
......................................................................aauugcacuugGcccggccu......................	5	1	seq
......................................................................aNuugcacuugucccggccu......................	2	1	seq
......................................................................aauugcacuugucNcggccu......................	1	1	seq
......................................................................aauugcacuugucccAgccu......................	24	1	seq
```

```
                        Star                                         Mature
gagaugacugcacuugacuacaaguaggccuugacucgugcaacuuugagccgcgauucuguacaccacaaauugcacuugucccggccugccuguggucaugccucgcagc
......................................................................aauuUcacuugucccggccu......................    4       1       seq
......................................................................aauugcacuugucGcggccu......................    4       1       seq
......................................................................aauugcacuuguccAggccu......................    20      1       seq
......................................................................aauugcacuugucccgCccu......................    4       1       seq
......................................................................aauugcacuugucccCgccu......................    3       1       seq
......................................................................aauugAacuugucccggccu......................    23      1       seq
......................................................................aauugcacuGgucccggccu......................    8       1       seq
......................................................................aauugcacuugucccUgccu......................    5       1       seq
......................................................................aauGgcacuugucccggccu......................    1       1       seq
......................................................................aauugcacuugCcccggccu......................    8       1       seq
......................................................................aauugcacAugucccggccu......................    17      1       seq
......................................................................aauugcacuugucccggUcu......................    8       1       seq
......................................................................aauugcacuuAucccggccu......................    11      1       seq
......................................................................aauugcacuuguGccggccu......................    6       1       seq
......................................................................aauugcacuugAcccggccu......................    35      1       seq
......................................................................aauAgcacuugucccggccu......................    29      1       seq
......................................................................aauuCcacuugucccggccu......................    3       1       seq
......................................................................Gauugcacuugucccggccu......................    50      1       seq
......................................................................aauugcacuugucccgUccu......................    4       1       seq
......................................................................aauugGacuugucccggccu......................    5       1       seq
......................................................................aauugcaGuugucccggccu......................    3       1       seq
......................................................................aauugcacuuUucccggccu......................    14      1       seq
......................................................................aauugcacuugucccggcAu......................    7       1       seq
......................................................................aauugcacuugucccggcGu......................    3       1       seq
......................................................................aauugcacuugucUcggccu......................    124     1       seq
......................................................................aauugcacuugucccggAcu......................    12      1       seq
......................................................................aauugcUcuugucccggccu......................    6       1       seq
......................................................................aauugcacuCgucccggccu......................    7       1       seq
......................................................................aauugcCcuugucccggccu......................    3       1       seq
......................................................................aauugcacuugucccggccC......................    12      1       seq
......................................................................Nauugcacuugucccggccu......................    26      1       seq
......................................................................aauugcacuAgucccggccu......................    88      1       seq
......................................................................aauugcacuugucccggccu......................    69534   0       seq
......................................................................aauugcacuugucccgAccu......................    10      1       seq
......................................................................aauugcacuugucccggccA......................    165     1       seq
......................................................................aauugcacuuguUccggccu......................    18      1       seq
......................................................................aauugUacuugucccggccu......................    10      1       seq
......................................................................aauugcacuugucccggccG......................    267     1       seq
......................................................................aauugcacuuguAccggccu......................    28      1       seq
......................................................................aauugcaUuugucccggccu......................    4       1       seq
......................................................................aauugcacuuguccGggccu......................    9       1       seq
......................................................................aauugcacuuCucccggccu......................    3       1       seq
......................................................................aCuugcacuugucccggccu......................    1       1       seq
......................................................................aUuugcacuugucccggccu......................    4       1       seq
......................................................................aauugcacuugucccggGcu......................    2       1       seq
......................................................................aaGugcacuugucccggccu......................    1       1       seq
......................................................................aauuAcacuugucccggccu......................    8       1       seq
......................................................................aaCugcacuugucccggccu......................    7       1       seq
......................................................................Cauugcacuugucccggccu......................    2       1       seq
......................................................................aauCgcacuugucccggccu......................    12      1       seq
......................................................................aauugNacuugucccggccug.....................    27      1       seq
......................................................................aauugUacuugucccggccug.....................    88      1       seq
......................................................................aauugcacuugucccgUccug.....................    32      1       seq
......................................................................aauugcacuugucccggccuN.....................    3       1       seq
......................................................................aauugcacuugucccggcAug.....................    87      1       seq
......................................................................aNuugcacuugucccggccug.....................    11      1       seq
......................................................................aauugcacuugucccggccuU.....................    252     1       seq
......................................................................aauugcacGugucccggccug.....................    55      1       seq
......................................................................aauugcCcuugucccggccug.....................    31      1       seq
......................................................................aauugcacuuguUccggccug.....................    66      1       seq
......................................................................Gauugcacuugucccggccug.....................    724     1       seq
......................................................................aauugcacuugucccggccug.....................    571031  0       seq
......................................................................aauugcacuuguccAggccug.....................    222     1       seq
......................................................................aauuAcacuugucccggccug.....................    67      1       seq
......................................................................aauCgcacuugucccggccug.....................    73      1       seq
......................................................................aauugcacuugucccgCccug.....................    31      1       seq
......................................................................aauugcacuugucccggccGg.....................    4124    1       seq
......................................................................aauugcacuuguccUggccug.....................    354     1       seq
......................................................................aaNugcacuugucccggccug.....................    1       1       seq
......................................................................aauugcacuuguGccggccug.....................    35      1       seq
```

Star Mature

| | | | |
|---|---|---|---|
| gagaugacugcacuugacuacaaguaggccuugacucgugcaacuuugagccgcgauucuguacaccacaaauugcacuugucccggccugccuguggucaugccucgcagc | | | |
| ......................................................................aauuUcacuugucccggccug..................... | 47 | 1 | seq |
| ......................................................................aauugcacuAgucccggccug..................... | 271 | 1 | seq |
| ......................................................................aauugcacuugGcccggccug..................... | 26 | 1 | seq |
| ......................................................................aauugcacuCgucccggccug..................... | 67 | 1 | seq |
| ......................................................................aauugcacuugucccggGcug..................... | 51 | 1 | seq |
| ......................................................................aauugcacuugucccggccCg..................... | 82 | 1 | seq |
| ......................................................................aauugcacuugucccggccuC..................... | 152 | 1 | seq |
| ......................................................................aauugGacuugucccggccug..................... | 37 | 1 | seq |
| ......................................................................aCuugcacuugucccggccug..................... | 1 | 1 | seq |
| ......................................................................Uauugcacuugucccggccug..................... | 51 | 1 | seq |
| ......................................................................aauugcacuugucccgNccug..................... | 1 | 1 | seq |
| ......................................................................aauugcGcuugucccggccug..................... | 122 | 1 | seq |
| ......................................................................aauugcacuugucccAgccug..................... | 207 | 1 | seq |
| ......................................................................aGuugcacuugucccggccug..................... | 53 | 1 | seq |
| ......................................................................aauugcacuNgucccggccug..................... | 4 | 1 | seq |
| ......................................................................aauugcacuugucccCgccug..................... | 24 | 1 | seq |
| ......................................................................aauugcacuuNucccggccug..................... | 2 | 1 | seq |
| ......................................................................aauugcacuugucUcggccug..................... | 464 | 1 | seq |
| ......................................................................aauugcacuugucccNgccug..................... | 1 | 1 | seq |
| ......................................................................aauAgcacuugucccggccug..................... | 188 | 1 | seq |
| ......................................................................aauugcacuuAucccggccug..................... | 98 | 1 | seq |
| ......................................................................aaGugcacuugucccggccug..................... | 15 | 1 | seq |
| ......................................................................aauugcacuugucccgAccug..................... | 64 | 1 | seq |
| ......................................................................aaCugcacuugucccggccug..................... | 28 | 1 | seq |
| ......................................................................Nauugcacuugucccggccug..................... | 139 | 1 | seq |
| ......................................................................aauugcNcuugucccggccug..................... | 3 | 1 | seq |
| ......................................................................aauugcacuugucGcggccug..................... | 50 | 1 | seq |
| ......................................................................aaAugcacuugucccggccug..................... | 560 | 1 | seq |
| ......................................................................aauugcacuugucccUgccug..................... | 32 | 1 | seq |
| ......................................................................aauugcacuugucccggcGug..................... | 40 | 1 | seq |
| ......................................................................aauugcacuuCucccggccug..................... | 26 | 1 | seq |
| ......................................................................aauugcacuugucccggUcug..................... | 71 | 1 | seq |
| ......................................................................aauugcacuugAcccggccug..................... | 226 | 1 | seq |
| ......................................................................aauugcacuuUucccggccug..................... | 34 | 1 | seq |
| ......................................................................aauugcacuuguAccggccug..................... | 182 | 1 | seq |
| ......................................................................aauugcacuugucccggAcug..................... | 109 | 1 | seq |
| ......................................................................aauugAacuugucccggccug..................... | 317 | 1 | seq |
| ......................................................................aauugcacuugucccggcUug..................... | 121 | 1 | seq |
| ......................................................................aauuNcacuugucccggccug..................... | 2 | 1 | seq |
| ......................................................................aauuCcacuugucccggccug..................... | 24 | 1 | seq |
| ......................................................................aauugcacuGgucccggccug..................... | 13 | 1 | seq |
| ......................................................................aauugcaGuugucccggccug..................... | 16 | 1 | seq |
| ......................................................................aauugcacuuguccGggccug..................... | 71 | 1 | seq |
| ......................................................................aauugcaAuugucccggccug..................... | 77 | 1 | seq |
| ......................................................................aauugcacuugucccggccuA..................... | 91 | 1 | seq |
| ......................................................................aUuugcacuugucccggccug..................... | 43 | 1 | seq |
| ......................................................................aauugcaUuugucccggccug..................... | 20 | 1 | seq |
| ......................................................................aauugcacAugucccggccug..................... | 96 | 1 | seq |
| ......................................................................aauugcacCugucccggccug..................... | 126 | 1 | seq |
| ......................................................................aauugcacuugucAcggccug..................... | 436 | 1 | seq |
| ......................................................................aauugcacuugucccggccAg..................... | 108 | 1 | seq |
| ......................................................................aauugcacuugCcccggccug..................... | 161 | 1 | seq |
| ......................................................................aauugcUcuugucccggccug..................... | 51 | 1 | seq |
| ......................................................................Cauugcacuugucccggccug..................... | 8 | 1 | seq |
| ......................................................................aauGgcacuugucccggccug..................... | 7 | 1 | seq |
| ......................................................................aaGugcacuugucccggccugc.................... | 38 | 1 | seq |
| ......................................................................aauugcacuugucccggccNgc.................... | 6 | 1 | seq |
| ......................................................................aauugcacuugucNcggccugc.................... | 10 | 1 | seq |
| ......................................................................aauugcacAugucccggccugc.................... | 718 | 1 | seq |
| ......................................................................aauugcacuugucccAgccugc.................... | 1663 | 1 | seq |
| ......................................................................aaAugcacuugucccggccugc.................... | 3930 | 1 | seq |
| ......................................................................aauugcacuugucccggcUugc.................... | 742 | 1 | seq |
| ......................................................................aauugcacuugucccggccuNc.................... | 34 | 1 | seq |
| ......................................................................aauugcacuuguUccggccugc.................... | 936 | 1 | seq |
| ......................................................................aauugcaGuugucccggccugc.................... | 168 | 1 | seq |
| ......................................................................aauugcacuugucccggccugU.................... | 47228 | 1 | seq |
| ......................................................................aauugAacuugucccggccugc.................... | 2138 | 1 | seq |
| ......................................................................Cauugcacuugucccggccugc.................... | 78 | 1 | seq |
| ......................................................................aauugcaNuugucccggccugc.................... | 3 | 1 | seq |
| ......................................................................aauugcacuugucAcggccugc.................... | 2790 | 1 | seq |

```
                               Star                                         Mature
gagaugacugcacuugacuacaaguaggccuugacucgugcaacuuugagccgcgauucuguacaccacaaauugcacuugucccggccugccuguggucaugccucgcagc
......................................................................aauugcacuugucccCgccugc....................    223       1          seq
......................................................................aauugNacuugucccggccugc....................    132       1          seq
......................................................................aauugcacuugucccgUccugc....................    315       1          seq
......................................................................aauugcacuugucccgAccugc....................    629       1          seq
......................................................................aauugcacuugucccggccAgc....................    738       1          seq
......................................................................aauugcacGugucccggccugc....................    296       1          seq
......................................................................aauuAcacuugucccggccugc....................    564       1          seq
......................................................................aauugcacuuguccGggccugc....................    384       1          seq
......................................................................aauugcacuugucccggccGgc....................    213       1          seq
......................................................................aauugcacuAgucccggccugc....................    2123      1          seq
......................................................................aNuugcacuugucccggccugc....................    115       1          seq
......................................................................aauugcacuuUucccggccugc....................    353       1          seq
......................................................................aauCgcacuugucccggccugc....................    731       1          seq
......................................................................aauugcacuugucUcggccugc....................    3309      1          seq
......................................................................aauugcacCugucccggccugc....................    2085      1          seq
......................................................................aUuugcacuugucccggccugc....................    274       1          seq
......................................................................aauugcacuugucccgNccugc....................    6         1          seq
......................................................................aauugcacuugucccggccuUc....................    274       1          seq
......................................................................aauugcacuugAcccggccugc....................    1931      1          seq
......................................................................aCuugcacuugucccggccugc....................    12        1          seq
......................................................................aauugcacuugucccggUcugc....................    424       1          seq
......................................................................aauugcacuGgucccggccugc....................    279       1          seq
......................................................................aauugcacuugucccggccugA....................    5093      1          seq
......................................................................aauugcacuugucccggccuAc....................    504       1          seq
......................................................................aauugcacuugucccggcAugc....................    541       1          seq
......................................................................aauugcaAuugucccggccugc....................    520       1          seq
......................................................................aauuUcacuugucccggccugc....................    496       1          seq
......................................................................aauAgcacuugucccggccugc....................    1536      1          seq
......................................................................aauugcacuugucccUgccugc....................    231       1          seq
......................................................................aauGgcacuugucccggccugc....................    108       1          seq
......................................................................aauugcacuuguAccggccugc....................    1393      1          seq
......................................................................aauugcacuugucccggccuCc....................    252       1          seq
......................................................................aauugcacuugucccggGcugc....................    322       1          seq
......................................................................aauugcacuuAucccggccugc....................    610       1          seq
......................................................................aauugcacuuCucccggccugc....................    239       1          seq
......................................................................aauugcacuugucGcggccugc....................    334       1          seq
......................................................................aauuCcacuugucccggccugc....................    224       1          seq
......................................................................aauugcacuugGcccggccugc....................    212       1          seq
......................................................................aauugcacuugucccggAcugc....................    779       1          seq
......................................................................Gauugcacuugucccggccugc....................    5091      1          seq
......................................................................aauugcacuCgucccggccugc....................    611       1          seq
......................................................................aauugcacuugucccggcNugc....................    6         1          seq
......................................................................aauugcacuuNucccggccugc....................    7         1          seq
......................................................................Uauugcacuugucccggccugc....................    403       1          seq
......................................................................aGuugcacuugucccggccugc....................    379       1          seq
......................................................................aauugcacuugCcccggccugc....................    694       1          seq
......................................................................aauugcacuugucccggccugG....................    1620      1          seq
......................................................................aauugcGcuugucccggccugc....................    1242      1          seq
......................................................................aauugcacNugucccggccugc....................    10        1          seq
......................................................................aauugcacuugucccNgccugc....................    6         1          seq
......................................................................aauugcacuuguccAggccugc....................    1374      1          seq
......................................................................aauugcaUuugucccggccugc....................    272       1          seq
......................................................................Nauugcacuugucccggccugc....................    995       1          seq
......................................................................aaNugcacuugucccggccugc....................    15        1          seq
......................................................................aauugcNcuugucccggccugc....................    2         1          seq
......................................................................aauugcCcuugucccggccugc....................    252       1          seq
......................................................................aauugcacuugucccggccugN....................    11        1          seq
......................................................................aaCugcacuugucccggccugc....................    399       1          seq
......................................................................aauugcacuuguGccggccugc....................    265       1          seq
......................................................................aauugcacuugucccggccCgc....................    540       1          seq
......................................................................aauugcacuNgucccggccugc....................    33        1          seq
......................................................................aauugGacuugucccggccugc....................    225       1          seq
......................................................................aauugcacuugucccgCccugc....................    214       1          seq
......................................................................aauugcacuuguccUggccugc....................    2520      1          seq
......................................................................aauugUacuugucccggccugc....................    484       1          seq
......................................................................aauugcacuuguccNggccugc....................    1         1          seq
......................................................................aauugcUcuugucccggccugc....................    549       1          seq
......................................................................aauugcacuugucccggcGugc....................    245       1          seq
......................................................................aauugcacuugucccggccugc....................    4031036   0          seq
......................................................................Gauugcacuugucccggccugcc...................    1         1          seq
```

```
                         Star                                            Mature
gagaugacugcacuugacuacaaguaggccuugacucgugcaacuuugagccgcgauucuguacaccacaaauugcacuugucccggccugccuguggucaugccucgcagc
..........................................................................aaAugcacuugucccggccugcc...................    1       1    seq
..........................................................................aauugcacuugucccggccuUcc...................    1       1    seq
..........................................................................aauugcacuugucccggccugUc...................    1       1    seq
..........................................................................aauugcacuugucccggccugcA...................    10731   1    seq
..........................................................................aGuugcacuugucccggccugcc...................    1       1    seq
..........................................................................aauugcacuugucUcggccugcc...................    1       1    seq
..........................................................................aUuugcacuugucccggccugcc...................    1       1    seq
..........................................................................aauugcacuuguccUggccugcc...................    1       1    seq
..........................................................................aauugcacuuguccAggccugcc...................    1       1    seq
..........................................................................aauugcacuugAcccggccugcc...................    1       1    seq
..........................................................................aauugcacuugucccggccugcc...................    665     0    seq
..........................................................................aauugcacuugucccggccugcN...................    7       1    seq
..........................................................................aauugcGcuugucccggccugcc...................    1       1    seq
..........................................................................aauugcacuugucccggccugcG...................    329     1    seq
..........................................................................aauugcacuugucUcggccugccu..................    1       1    seq
..........................................................................aauugGacuugucccggccugccu..................    1       1    seq
..........................................................................aauugcacuugucccggccugccA..................    10      1    seq
..........................................................................aauugcacuugucccggccugcUu..................    9253    1    seq
..........................................................................aauugcacuugucccggccugcGu..................    17      1    seq
..........................................................................aauugcacuugucccggccugccu..................    79      0    seq
..........................................................................aauugcacuugucccggccugcAu..................    452     1    seq
..........................................................................aauugcacuugucccggccugccG..................    1       1    seq
..........................................................................aauugcacuugucccggccugcUug.................    2       1    seq
...........................................................................aAugcacuugucccggcc.......................    1       1    seq
...........................................................................auugcacuugucccggcc.......................    62      0    seq
...........................................................................auugcacuugucccggccu......................    35      0    seq
...........................................................................auugcacuugucccggccG......................    1       1    seq
...........................................................................aAugcacuugucccggccug.....................    10      1    seq
...........................................................................auugcacuugucccggccug.....................    101     0    seq
...........................................................................auAgcacuugucccggccugc....................    2       1    seq
...........................................................................auugcacuugAcccggccugc....................    2       1    seq
...........................................................................auugcacuugucccggcUugc....................    1       1    seq
...........................................................................auugcacuuguccGggccugc....................    2       1    seq
...........................................................................auugcacuugucccgAccugc....................    1       1    seq
...........................................................................auugcacuugucccggAcugc....................    1       1    seq
...........................................................................auugcacuugucccggccugc....................    1606    0    seq
...........................................................................auugcacAugucccggccugc....................    2       1    seq
...........................................................................auugcacuugucAcggccugc....................    3       1    seq
...........................................................................auugcUcuugucccggccugc....................    1       1    seq
...........................................................................Guugcacuugucccggccugc....................    25      1    seq
...........................................................................auugcacuuAucccggccugc....................    1       1    seq
...........................................................................auugcacuugucccggccugU....................    1       1    seq
...........................................................................aAugcacuugucccggccugc....................    39      1    seq
...........................................................................aGugcacuugucccggccugc....................    1       1    seq
...........................................................................auugcacuuguAccggccugc....................    1       1    seq
...........................................................................auugcacuugucccggccugcA...................    115     1    seq
...........................................................................auugcacuugucccggccugcc...................    13      0    seq
...........................................................................auugcacuuguccUggccugcc...................    1       1    seq
...........................................................................auugcacuugucccggccugcUu..................    209     1    seq
...........................................................................auugcacuugucccggccugccA..................    1       1    seq
...........................................................................auugcacuugucccggccugccu..................    33      0    seq
............................................................................uugcacuugucccggccu......................    11      0    seq
............................................................................uugcacuugucccggccug.....................    1       0    seq
............................................................................uAgcacuugucccggccugc....................    1       1    seq
............................................................................uugcacuugucccggccugU....................    1       1    seq
............................................................................Augcacuugucccggccugc....................    1       1    seq
............................................................................uugcacuugucAcggccugc....................    1       1    seq
............................................................................uugcacuuguccUggccugc....................    1       1    seq
............................................................................uugcacuAgucccggccugc....................    2       1    seq
............................................................................uugcacuugucccggccugc....................    298     0    seq
............................................................................uugcacuugucccggccugcA...................    1       1    seq
.............................................................................ugcacuugucccggccugc....................    27      0    seq
.............................................................................ugcacuugucccggccugcc...................    21      0    seq
.............................................................................ugcacuugucccggccugccA..................    3       1    seq
.............................................................................ugcacuugucccggccugccu..................    98      0    seq
.............................................................................ugcacuugucccUgccugccu..................    1       1    seq
.............................................................................ugcacuugCcccggccugccug.................    2       1    seq
.............................................................................Agcacuugucccggccugccug.................    1       1    seq
.............................................................................ugcacuugucccggccugccGg.................    1       1    seq
.............................................................................ugcacuugucccggccugccug.................    96      0    seq
```

```
                         Star                                          Mature
gagaugacugcacuugacuacaaguaggccuugacucgugcaacuuugagccgcgauucuguacaccacaaauugcacuugucccggccugccuguggucaugccucgcagc
.........................................................................ugcacuugucccggccugccugA................    3    1    seq
..........................................................................gcacuugucccggccugU....................    1    1    seq
..........................................................................gcacuugucccggccugc....................  188    0    seq
.............................................................................cuugucccggccugccug.................    1    0    seq
```
