# Supplementary material for: The Small RNA Universe of Capitella teleta
Source: Front Mol Biosci. 2022 Feb 25;9:802814. doi: 10.3389/fmolb.2022.802814 (PMC8915122; doi:10.3389/fmolb.2022.802814)
Supplement: Supplementary file 1 [file DataSheet1.ZIP › Supplement/homologRecovered/CAPTEscaffold_70_6936.pdf]

Provisional ID	:	CAPTEscaffold_70_6936
Score total	:	1170625.6
Score for star read(s)	:	3.9
Score for read counts	:	1170615.7
Score for mfe	:	1.4
Score for randfold	:	1.6
Score for cons. seed	:	3
Total read count	:	2296124
Mature read count	:	2295978
Loop read count	:	0
Star read count	:	146

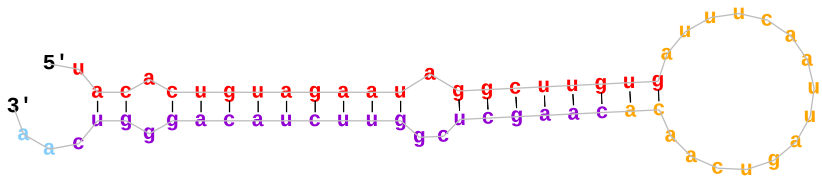

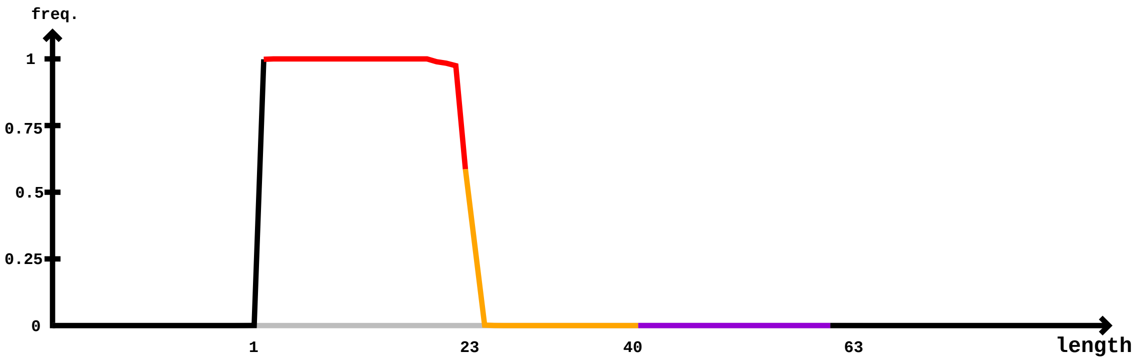

Mature **Star**

```
5'- gaguuuuagcaauaaccauguacacuguagaauaggcuugugauuucaauuagucaacacaagcucgguucuacagggucaaugggcauugugacuuucaucuccaug  -3'  obs
    gaguuuuagcaauaaccauguacacuguagaauaggcuugugauuucaauuagucaacacaagcucgguucuacagggucaaugggcauugugacuuucaucuccaug       exp
    (((((...(((((..((((..((.(((((((((.((((((((..............))))))))..))))))))).))..))))..))))))))))............   reads   mm   sample
    .................Guguacacuguagaauaggcuu.....................................................................   29      1    seq
    .................auCuacacuguagaauaggcuugu...................................................................   10      1    seq
    .................auCuacacuguagaauaggcuugug..................................................................   42      1    seq
    ..................uCuacacuguagaauaggcu......................................................................   1       1    seq
    ..................uguacacuguagaauaggcuug....................................................................   1       0    seq
    ..................uCuacacuguagaauaggcuugu...................................................................   38      1    seq
    ..................uCuacacuguagaauaggcuugug..................................................................   38      1    seq
    ..................Gguacacuguagaauaggcuugug..................................................................   4       1    seq
    ..................uAuacacuguagaauaggcuugug..................................................................   1       1    seq
    ...................Cuacacuguagaauaggc.......................................................................   3       1    seq
    ...................guacacuguagaauaggcu......................................................................   2       0    seq
    ...................Auacacuguagaauaggcuu.....................................................................   1       1    seq
    ...................guacacuguagaauaggcuu.....................................................................   6       0    seq
    ...................Cuacacuguagaauaggcuu.....................................................................   1       1    seq
    ...................Cuacacuguagaauaggcuug....................................................................   5       1    seq
    ...................guacacuguagaauaggcuug....................................................................   15      0    seq
    ...................Cuacacuguagaauaggcuugu...................................................................   233     1    seq
    ...................Auacacuguagaauaggcuugu...................................................................   7       1    seq
    ...................guacacuguagaauaggcuugu...................................................................   21      0    seq
    ...................Uuacacuguagaauaggcuugug..................................................................   4       1    seq
    ...................guacacuguagaauaggcuugug..................................................................   9       0    seq
    ...................Cuacacuguagaauaggcuugug..................................................................   147     1    seq
    ...................Auacacuguagaauaggcuugug..................................................................   10      1    seq
    ...................Cuacacuguagaauaggcuuguga.................................................................   7       1    seq
    ...................guacacuguagaauaggcuugugU.................................................................   1       1    seq
    ....................Cacacuguagaauaggcu......................................................................   5       1    seq
    ....................uacacuguaCaauaggcu......................................................................   3       1    seq
    ....................uacacuguagaauaggcG......................................................................   29      1    seq
    ....................Gacacuguagaauaggcu......................................................................   11      1    seq
    ....................uacacuguagaGuaggcu......................................................................   1       1    seq
    ....................uacacuguagaauaUgcu......................................................................   2       1    seq
    ....................uacacuguagaauaggcu......................................................................   23877   0    seq
    ....................uacacuguagaauaggAu......................................................................   9       1    seq
```

```
                    Mature                                    Star
gaguuuuagcauaaccauguacacuguagaauaggcuugugauuucaauuagucaacacaagcucgguucuacagggucaaugggcauugugacuuucaucuccaug
...................uNcacuguagaauaggcu......................................................................   2      1   seq
...................uacacCguagaauaggcu......................................................................   4      1   seq
...................uacacugAagaauaggcu......................................................................   19     1   seq
...................uGcacuguagaauaggcu......................................................................   7      1   seq
...................uaAacuguagaauaggcu......................................................................   15     1   seq
...................uacacuguagaauaggcC......................................................................   65     1   seq
...................uUcacuguagaauaggcu......................................................................   4      1   seq
...................uacacuCuagaauaggcu......................................................................   4      1   seq
...................uacacuguagaauaggGu......................................................................   2      1   seq
...................uacacuguGgaauaggcu......................................................................   5      1   seq
...................uacacuAuagaauaggcu......................................................................   2      1   seq
...................uacacuguagaauaggUu......................................................................   2      1   seq
...................uacacuguagaauagUcu......................................................................   3      1   seq
...................uacacuguagaauagAcu......................................................................   2      1   seq
...................uacUcuguagaauaggcu......................................................................   1      1   seq
...................uacacuguaAaauaggcu......................................................................   5      1   seq
...................uacacuguagaauaAgcu......................................................................   6      1   seq
...................uacacuguagaauUggcu......................................................................   2      1   seq
...................uacacuguUgaauaggcu......................................................................   4      1   seq
...................uacacuguagaaGaggcu......................................................................   1      1   seq
...................uacacuguagaauaggcA......................................................................   34     1   seq
...................uacacuguagGauaggcu......................................................................   2      1   seq
...................uacGcuguagaauaggcu......................................................................   4      1   seq
...................uacaGuguagaauaggcu......................................................................   1      1   seq
...................uacacugCagaauaggcu......................................................................   2      1   seq
...................uacacuguagaauagCcu......................................................................   1      1   seq
...................uacacuUuagaauaggcu......................................................................   5      1   seq
...................uacacNguagaauaggcu......................................................................   1      1   seq
...................uacacAguagaauaggcu......................................................................   18     1   seq
...................uacacuguagaauGggcu......................................................................   2      1   seq
...................uacacuguagaaAaggcu......................................................................   9      1   seq
...................uacacuguagaUuaggcu......................................................................   1      1   seq
...................Aacacuguagaauaggcu......................................................................   221    1   seq
...................uacaAuguagaauaggcu......................................................................   3      1   seq
...................Nacacuguagaauaggcu......................................................................   9      1   seq
...................uacacuguaUaauaggcu......................................................................   2      1   seq
...................uacacGguagaauaggcu......................................................................   1      1   seq
...................uUcacuguagaauaggcuu.....................................................................   1      1   seq
...................uacacuguGgaauaggcuu.....................................................................   7      1   seq
...................uacacuguagaauaggcuu.....................................................................   12364  0   seq
...................Gacacuguagaauaggcuu.....................................................................   9      1   seq
...................uacacuguagaauagAcuu.....................................................................   1      1   seq
...................uacacuguagaauaggcGu.....................................................................   9      1   seq
...................uacacuguagaauaggAuu.....................................................................   2      1   seq
...................uacUcuguagaauaggcuu.....................................................................   1      1   seq
...................uacGcuguagaauaggcuu.....................................................................   3      1   seq
...................uacacuguagaauaggcuA.....................................................................   79     1   seq
...................uacacuguUgaauaggcuu.....................................................................   1      1   seq
...................uacacuAuagaauaggcuu.....................................................................   1      1   seq
...................uGcacuguagaauaggcuu.....................................................................   1      1   seq
...................uacacuguagaauaggcuC.....................................................................   4      1   seq
...................Cacacuguagaauaggcuu.....................................................................   4      1   seq
...................uacacuguagaauaAgcuu.....................................................................   1      1   seq
...................uacacuguaAaauaggcuu.....................................................................   4      1   seq
...................Aacacuguagaauaggcuu.....................................................................   119    1   seq
...................uacacuguagaauGggcuu.....................................................................   2      1   seq
...................Nacacuguagaauaggcuu.....................................................................   2      1   seq
...................uacacuguagaauaggUuu.....................................................................   1      1   seq
...................uacacuguagaauaggcCu.....................................................................   4      1   seq
...................uacacuguagaauUggcuu.....................................................................   1      1   seq
...................uacacuguagaGuaggcuu.....................................................................   3      1   seq
...................uacacuguaUaauaggcuu.....................................................................   1      1   seq
...................uaUacuguagaauaggcuu.....................................................................   2      1   seq
...................uacacuUuagaauaggcuu.....................................................................   1      1   seq
...................uacacuguagaauaggcuG.....................................................................   6      1   seq
...................uacacCguagaauaggcuu.....................................................................   1      1   seq
...................uaAacuguagaauaggcuu.....................................................................   10     1   seq
...................uacacuguagaaGaggcuu.....................................................................   2      1   seq
...................uacacuguagaaAaggcuu.....................................................................   5      1   seq
...................uacacuguagaauaggcAu.....................................................................   7      1   seq
```

Mature **Star**

```
gaguuuuagcaauaaccauguacacuguagaauaggcuugugauuucaauuagucaacacaagcucgguucuacagggucaaugggcauugugacuuucaucuccaug
....................uacaAuguagaauaggcuu.....................................................................     2      1    seq
....................uacacGguagaauaggcuu.....................................................................     1      1    seq
....................uacacuguagUauaggcuu.....................................................................     3      1    seq
....................uacacuguaCaauaggcuu.....................................................................     1      1    seq
....................uacacAguagaauaggcuu.....................................................................     9      1    seq
....................uacacuguagGauaggcuu.....................................................................     1      1    seq
....................uacacugCagaauaggcuu.....................................................................     2      1    seq
....................uacacugAagaauaggcuu.....................................................................     6      1    seq
....................uacacuguagaauaggcuuA....................................................................     14     1    seq
....................uacCcuguagaauaggcuug....................................................................     1      1    seq
....................uacacuguagaauaggcuug....................................................................     20648  0    seq
....................uacacCguagaauaggcuug....................................................................     1      1    seq
....................uacacuguUgaauaggcuug....................................................................     1      1    seq
....................uacacuguagaauaAgcuug....................................................................     1      1    seq
....................uacacuguagaauaggcGug....................................................................     1      1    seq
....................uacacuguagGauaggcuug....................................................................     2      1    seq
....................uacacuguagaauaggcuAg....................................................................     4      1    seq
....................uacacuguaCaauaggcuug....................................................................     1      1    seq
....................Aacacuguagaauaggcuug....................................................................     206    1    seq
....................Nacacuguagaauaggcuug....................................................................     6      1    seq
....................uacacuguagaauGggcuug....................................................................     1      1    seq
....................uacacuguagaauagUcuug....................................................................     2      1    seq
....................uUcacuguagaauaggcuug....................................................................     2      1    seq
....................uacacAguagaauaggcuug....................................................................     13     1    seq
....................uacacuguagaUuaggcuug....................................................................     2      1    seq
....................uacacuguagaaGaggcuug....................................................................     2      1    seq
....................uacacuCuagaauaggcuug....................................................................     4      1    seq
....................uacacugAagaauaggcuug....................................................................     9      1    seq
....................uacacuUuagaauaggcuug....................................................................     5      1    seq
....................uaAacuguagaauaggcuug....................................................................     8      1    seq
....................uacacuguaAaauaggcuug....................................................................     14     1    seq
....................uacacuguagaaAaggcuug....................................................................     8      1    seq
....................uacUcuguagaauaggcuug....................................................................     5      1    seq
....................uacacuguagaauaggcCug....................................................................     5      1    seq
....................uaUacuguagaauaggcuug....................................................................     2      1    seq
....................uacacuguagaauaggcuGg....................................................................     12     1    seq
....................uacacugGagaauaggcuug....................................................................     2      1    seq
....................uacacuguagUauaggcuug....................................................................     2      1    seq
....................uGcacuguagaauaggcuug....................................................................     3      1    seq
....................uacacuguagaauaggUuug....................................................................     1      1    seq
....................uacacuguagaaCaggcuug....................................................................     3      1    seq
....................Gacacuguagaauaggcuug....................................................................     15     1    seq
....................uacaAuguagaauaggcuug....................................................................     4      1    seq
....................uacacuguagaauaggcuuC....................................................................     1      1    seq
....................uacacugCagaauaggcuug....................................................................     3      1    seq
....................uacacuguagaauagAcuug....................................................................     3      1    seq
....................uacacuguagaauaggGuug....................................................................     2      1    seq
....................uacacuguGgaauaggcuug....................................................................     6      1    seq
....................uacacuguagaauaggcAug....................................................................     6      1    seq
....................uacacuguagaauaggAuug....................................................................     8      1    seq
....................uacacuguagaGuaggcuug....................................................................     1      1    seq
....................uaGacuguagaauaggcuug....................................................................     1      1    seq
....................uacacuguaUaauaggcuug....................................................................     1      1    seq
....................uacacuguagaauaggcuCg....................................................................     2      1    seq
....................uacacuguagaauaggcuuU....................................................................     106    1    seq
....................uacacuguagaauagCcuug....................................................................     1      1    seq
....................uacacuguagaauaUgcuug....................................................................     1      1    seq
....................Cacacuguagaauaggcuug....................................................................     3      1    seq
....................uacacuAuagaauaggcuug....................................................................     6      1    seq
....................uacaGuguagaauaggcuugu...................................................................     41     1    seq
....................uacacuguagaauaUgcuugu...................................................................     50     1    seq
....................uacacCguagaauaggcuugu...................................................................     113    1    seq
....................uacacuguagCauaggcuugu...................................................................     7      1    seq
....................uacacuguagaGuaggcuugu...................................................................     139    1    seq
....................uacacuguagaauaggAuugu...................................................................     285    1    seq
....................uacacuguagaauaggcuuCu...................................................................     63     1    seq
....................uacCcuguagaauaggcuugu...................................................................     21     1    seq
....................uacacuguagaauaggUuugu...................................................................     76     1    seq
....................uUcacuguagaauaggcuugu...................................................................     87     1    seq
....................uacacuAuagaauaggcuugu...................................................................     117    1    seq
```

Mature **Star**

```
gaguuuuagcaauaaccauguacacuguagaauaggcuugugauuucaauuagucaacacaagcucgguucuacagggucaaugggcauugugacuuucaucuccaug
....................uacacuguagaauaggcAugu...................................................................   273      1   seq
....................uacacuguagaauNggcuugu...................................................................   1        1   seq
....................uacacuguagaauaggcuuUu...................................................................   71       1   seq
....................uacacuguCgaauaggcuugu...................................................................   4        1   seq
....................uacacuguagaauUggcuugu...................................................................   91       1   seq
....................Cacacuguagaauaggcuugu...................................................................   127      1   seq
....................uacacuguagaauagUcuugu...................................................................   93       1   seq
....................uacacuguagaauagCcuugu...................................................................   36       1   seq
....................uacacuguagaauaCgcuugu...................................................................   39       1   seq
....................uacacuguagaauaAgcuugu...................................................................   168      1   seq
....................Gacacuguagaauaggcuugu...................................................................   552      1   seq
....................uacacugCagaauaggcuugu...................................................................   136      1   seq
....................uaAacuguagaauaggcuugu...................................................................   340      1   seq
....................Aacacuguagaauaggcuugu...................................................................   8471     1   seq
....................uacacuguagaaAaggcuugu...................................................................   304      1   seq
....................uacacuguagaauaggcuugG...................................................................   590      1   seq
....................uNcacuguagaauaggcuugu...................................................................   32       1   seq
....................uacacuguagUauaggcuugu...................................................................   69       1   seq
....................uaUacuguagaauaggcuugu...................................................................   80       1   seq
....................uacacuguagaUuaggcuugu...................................................................   96       1   seq
....................uacacuguagaauaggcuugu...................................................................   871928   0   seq
....................uCcacuguagaauaggcuugu...................................................................   3        1   seq
....................uacacugAagaauaggcuugu...................................................................   544      1   seq
....................uacacuguagaaGaggcuugu...................................................................   62       1   seq
....................uaGacuguagaauaggcuugu...................................................................   27       1   seq
....................uacacuguagaauagAcuugu...................................................................   114      1   seq
....................uacacuguagaauaggcuCgu...................................................................   98       1   seq
....................uacUcuguagaauaggcuugu...................................................................   100      1   seq
....................uacacuguagaauaggcuAgu...................................................................   316      1   seq
....................uacaAuguagaauaggcuugu...................................................................   103      1   seq
....................uacacuCuagaauaggcuugu...................................................................   81       1   seq
....................uacacuguagaCuaggcuugu...................................................................   4        1   seq
....................uacacuguUgaauaggcuugu...................................................................   71       1   seq
....................uacGcuguagaauaggcuugu...................................................................   156      1   seq
....................uacacuguaUaauaggcuugu...................................................................   80       1   seq
....................uacacAguagaauaggcuugu...................................................................   536      1   seq
....................uacacuguagaaCaggcuugu...................................................................   85       1   seq
....................uacacuguaCaauaggcuugu...................................................................   70       1   seq
....................uacacuUuagaauaggcuugu...................................................................   135      1   seq
....................uacacuguagaauaggcCugu...................................................................   96       1   seq
....................uGcacuguagaauaggcuugu...................................................................   157      1   seq
....................uacaUuguagaauaggcuugu...................................................................   52       1   seq
....................uacacuguagaauaggcuGgu...................................................................   69       1   seq
....................Nacacuguagaauaggcuugu...................................................................   257      1   seq
....................uacacuguagaauaggcuugC...................................................................   88       1   seq
....................uacacuguGgaauaggcuugu...................................................................   160      1   seq
....................uacacuguagaauaggGuugu...................................................................   54       1   seq
....................uacacugGagaauaggcuugu...................................................................   27       1   seq
....................uaNacuguagaauaggcuugu...................................................................   4        1   seq
....................uacacuguagaauaggcuugA...................................................................   757      1   seq
....................uacacuguagaauaggcGugu...................................................................   81       1   seq
....................uacacuguagaauaggcuuAu...................................................................   95       1   seq
....................uacacNguagaauaggcuugu...................................................................   4        1   seq
....................uacacuguaAaauaggcuugu...................................................................   259      1   seq
....................uacacuguagaauGggcuugu...................................................................   111      1   seq
....................uacacGguagaauaggcuugu...................................................................   31       1   seq
....................uacacuguagGauaggcuugu...................................................................   89       1   seq
....................uacacuguaNaauaggcuugu...................................................................   3        1   seq
....................uacacuguagaauCggcuugu...................................................................   6        1   seq
....................Aacacuguagaauaggcuugug..................................................................   8331     1   seq
....................uacacuguagaauaggcAugug..................................................................   413      1   seq
....................uCcacuguagaauaggcuugug..................................................................   7        1   seq
....................uaGacuguagaauaggcuugug..................................................................   35       1   seq
....................uaNacuguagaauaggcuugug..................................................................   5        1   seq
....................uNcacuguagaauaggcuugug..................................................................   26       1   seq
....................uacacuguagaauaUgcuugug..................................................................   63       1   seq
....................uacacuguagaaAaggcuugug..................................................................   437      1   seq
....................uacacugCagaauaggcuugug..................................................................   142      1   seq
....................uacacuguaUaauaggcuugug..................................................................   88       1   seq
....................uGcacuguagaauaggcuugug..................................................................   134      1   seq
```

Mature **Star**

```
gaguuuuagcaauaaccauguacacuguagaauaggcuugugauuucaauuagucaacacaagcucgguucuacagggucaaugggcauugugacuuucaucuccaug
....................uacacuguagaauaAgcuugug..................................................................  206     1  seq
....................uacacuguaAaauaggcuugug..................................................................  344     1  seq
....................uacacuUuagaauaggcuugug..................................................................  135     1  seq
....................uacacugGagaauaggcuugug..................................................................  30      1  seq
....................uacacuguagaauaggcGugug..................................................................  149     1  seq
....................uacacuguagaauaggcuuguA..................................................................  1923    1  seq
....................uacacuAuagaauaggcuugug..................................................................  151     1  seq
....................uacacuguagaauaggcuugAg..................................................................  623     1  seq
....................Cacacuguagaauaggcuugug..................................................................  180     1  seq
....................uacacCguagaauaggcuugug..................................................................  110     1  seq
....................uacacuguagaauaggNuugug..................................................................  1       1  seq
....................uaUacuguagaauaggcuugug..................................................................  197     1  seq
....................uacacuguagaCuaggcuugug..................................................................  4       1  seq
....................uacacuguagaauaggcuuguU..................................................................  10021   1  seq
....................uacacuguagaaCaggcuugug..................................................................  86      1  seq
....................uacacuguagaauCggcuugug..................................................................  8       1  seq
....................uacacuguagaauGggcuugug..................................................................  106     1  seq
....................uacacNguagaauaggcuugug..................................................................  8       1  seq
....................uacacGguagaauaggcuugug..................................................................  27      1  seq
....................uacacuguagaauaCgcuugug..................................................................  34      1  seq
....................uacacuguagaauaggGuugug..................................................................  84      1  seq
....................uacacuguagaauagUcuugug..................................................................  128     1  seq
....................uacacuguGgaauaggcuugug..................................................................  196     1  seq
....................uacacuguagUauaggcuugug..................................................................  90      1  seq
....................uacacuguagaauaggcuugug..................................................................  995741  0  seq
....................uacacuguagCauaggcuugug..................................................................  10      1  seq
....................uacacuguagaauagAcuugug..................................................................  136     1  seq
....................uacacuguagaauaggcuCgug..................................................................  162     1  seq
....................uacacuguagaauaggcuAgug..................................................................  388     1  seq
....................uacacugAagaauaggcuugug..................................................................  662     1  seq
....................uacacuguagaauaggcuuCug..................................................................  80      1  seq
....................uacaUuguagaauaggcuugug..................................................................  71      1  seq
....................uacacuguagaauNggcuugug..................................................................  1       1  seq
....................uacacuguagaauaggcuGgug..................................................................  74      1  seq
....................uacGcuguagaauaggcuugug..................................................................  151     1  seq
....................uacCcuguagaauaggcuugug..................................................................  37      1  seq
....................uacaGuguagaauaggcuugug..................................................................  39      1  seq
....................uaAacuguagaauaggcuugug..................................................................  384     1  seq
....................uacacuguagaauaggUuugug..................................................................  71      1  seq
....................uacacuguagaaGaggcuugug..................................................................  62      1  seq
....................uacacuguagaauaggcuugCg..................................................................  136     1  seq
....................uacacuguCgaauaggcuugug..................................................................  8       1  seq
....................uacacuguagaUuaggcuugug..................................................................  85      1  seq
....................uacaAuguagaauaggcuugug..................................................................  122     1  seq
....................Nacacuguagaauaggcuugug..................................................................  294     1  seq
....................uUcacuguagaauaggcuugug..................................................................  96      1  seq
....................uacacuguagaGuaggcuugug..................................................................  137     1  seq
....................uacacuguagaauaggcCugug..................................................................  104     1  seq
....................uacUcuguagaauaggcuugug..................................................................  108     1  seq
....................uacacuguagaauaggcuugGg..................................................................  236     1  seq
....................uacacuguagaauaggAuugug..................................................................  418     1  seq
....................uacacuguagaauaggcuugNg..................................................................  1       1  seq
....................uacacuCuagaauaggcuugug..................................................................  69      1  seq
....................uacacuguagaauaggcuuUug..................................................................  119     1  seq
....................Gacacuguagaauaggcuugug..................................................................  534     1  seq
....................uacacuguaCaauaggcuugug..................................................................  82      1  seq
....................uacacuguagaauagCcuugug..................................................................  47      1  seq
....................uacacAguagaauaggcuugug..................................................................  609     1  seq
....................uacacuguUgaauaggcuugug..................................................................  106     1  seq
....................uacacuguagaauaggcuuguC..................................................................  159     1  seq
....................uacacuguagaauUggcuugug..................................................................  95      1  seq
....................uacacuguagaauaggcuuguN..................................................................  1       1  seq
....................uacacuguagaauaggcuuAug..................................................................  138     1  seq
....................uacacuguagGauaggcuugug..................................................................  110     1  seq
....................uacacuguagaauaggcuugAga.................................................................  42      1  seq
....................uacacuguagaauaggcGuguga.................................................................  8       1  seq
....................Aacacuguagaauaggcuuguga.................................................................  606     1  seq
....................uacacuguagaauaggcuuguga.................................................................  65908   0  seq
....................uacacuguagaauaAgcuuguga.................................................................  15      1  seq
....................uNcacuguagaauaggcuuguga.................................................................  3       1  seq
```

```
                    Mature                                  Star
gaguuuuagcaauaaccauguacacuguagaauaggcuugugauuucaauuagucaacacaagcucgguucuacaggguc aaugggcauugugacuuucaucuccaug
....................uaUacuguagaauaggcuuguga.................................................................    4        1    seq
....................uacacugAagaauaggcuuguga.................................................................    30       1    seq
....................uacaGuguagaauaggcuuguga.................................................................    2        1    seq
....................uacacugGagaauaggcuuguga.................................................................    1        1    seq
....................uacacuguagaauagCcuuguga.................................................................    3        1    seq
....................uacacuguagaauaggcuuguCa.................................................................    6        1    seq
....................uacacuguagaauaggcAuguga.................................................................    24       1    seq
....................uacacuguagaauaggcuugGga.................................................................    6        1    seq
....................uacacuguagaauaUgcuuguga.................................................................    2        1    seq
....................uacacuguagaauaggGuuguga.................................................................    3        1    seq
....................uacCcuguagaauaggcuuguga.................................................................    1        1    seq
....................uacGcuguagaauaggcuuguga.................................................................    15       1    seq
....................uacacuguagaauaggcuuUuga.................................................................    12       1    seq
....................uacacuguagaauaggcuGguga.................................................................    2        1    seq
....................uacUcuguagaauaggcuuguga.................................................................    14       1    seq
....................uacacuCuagaauaggcuuguga.................................................................    4        1    seq
....................uacacuguagaaCaggcuuguga.................................................................    8        1    seq
....................uacacuguagaauaggcuuAuga.................................................................    10       1    seq
....................uacacuguagaauaggcCuguga.................................................................    8        1    seq
....................uacacuUuagaauaggcuuguga.................................................................    10       1    seq
....................uacacGguagaauaggcuuguga.................................................................    4        1    seq
....................uacacuguagaauaggcuugugG.................................................................    6962     1    seq
....................uacacuguagaauaggAuuguga.................................................................    29       1    seq
....................uaAacuguagaauaggcuuguga.................................................................    22       1    seq
....................uacacuguagaauaggcuugugC.................................................................    23636    1    seq
....................uacacuguagaaAaggcuuguga.................................................................    37       1    seq
....................uacacuguagaauaggcuugugU.................................................................    215253   1    seq
....................uacacuguagGauaggcuuguga.................................................................    6        1    seq
....................uacaAuguagaauaggcuuguga.................................................................    6        1    seq
....................uacacuguagaauaggcuAguga.................................................................    29       1    seq
....................uacacuguagaaGaggcuuguga.................................................................    6        1    seq
....................uacacuguagaauaggUuuguga.................................................................    3        1    seq
....................uacacuguagUauaggcuuguga.................................................................    6        1    seq
....................uacacCguagaauaggcuuguga.................................................................    4        1    seq
....................uacacuguagaauaggcuugugN.................................................................    3        1    seq
....................uacacuguagaauagAcuuguga.................................................................    8        1    seq
....................uacacuAuagaauaggcuuguga.................................................................    8        1    seq
....................uacacugCagaauaggcuuguga.................................................................    10       1    seq
....................uGcacuguagaauaggcuuguga.................................................................    8        1    seq
....................uacacuguagaauaggcuuguAa.................................................................    262      1    seq
....................Nacacuguagaauaggcuuguga.................................................................    12       1    seq
....................uacacuguagaauaggcuuCuga.................................................................    4        1    seq
....................Gacacuguagaauaggcuuguga.................................................................    43       1    seq
....................uacacuguagaauaggcuuguUa.................................................................    516      1    seq
....................uacacuguaAaauaggcuuguga.................................................................    18       1    seq
....................uacacuguaCaauaggcuuguga.................................................................    7        1    seq
....................uacacuguagaauaggcuugCga.................................................................    8        1    seq
....................Cacacuguagaauaggcuuguga.................................................................    14       1    seq
....................uacacuguagaGuaggcuuguga.................................................................    8        1    seq
....................uacacuguGgaauaggcuuguga.................................................................    15       1    seq
....................uacacuguaUaauaggcuuguga.................................................................    7        1    seq
....................uacacuguagaauaCgcuuguga.................................................................    4        1    seq
....................uacacuguagaauagUcuuguga.................................................................    10       1    seq
....................uacacuguagaauUggcuuguga.................................................................    8        1    seq
....................uacacuguUgaauaggcuuguga.................................................................    8        1    seq
....................uacaUuguagaauaggcuuguga.................................................................    3        1    seq
....................uUcacuguagaauaggcuuguga.................................................................    4        1    seq
....................uacacuguagaauGggcuuguga.................................................................    3        1    seq
....................uacacuguagaUuaggcuuguga.................................................................    3        1    seq
....................uacacAguagaauaggcuuguga.................................................................    52       1    seq
....................uacacuguagaauaggcuCguga.................................................................    9        1    seq
....................uacacuguagaauaggcuugugUu................................................................    1469     1    seq
....................uacacAguagaauaggcuugugau................................................................    1        1    seq
....................uacacuguagaaAaggcuugugau................................................................    1        1    seq
....................uacacuguagaauaggcuugugGu................................................................    5        1    seq
....................uacacuguagaauagAcuugugau................................................................    1        1    seq
....................uacacuguagaauaggcuugAgau................................................................    1        1    seq
....................uacacuguagaauaggcuugugCu................................................................    325      1    seq
....................Aacacuguagaauaggcuugugau................................................................    5        1    seq
....................uacacuguagaauaggcuugugaA................................................................    816      1    seq
```

```
                   Mature              Star
gaguuuuagcaauaaccauguacacuguagaauaggcuugugauuucaauuagucaacacaagcucgguucuacaggggucaaugggcauugugacuuucaucuccaug
....................uacacuguagaauaggcuuguUau................................................................	87	1	seq
....................uacacuguagaauaggcuugugaC................................................................	22	1	seq
....................uacacuguagaauaggcuuguAau................................................................	19	1	seq
....................uacacuguagUauaggcuugugau................................................................	1	1	seq
....................uacacuguagaauaggcuugugaG................................................................	28	1	seq
....................uacacuguagaauaggcuugugau................................................................	496	0	seq
....................uacacuguagaauaggcuugugauu...............................................................	48	0	seq
....................uacacuguagaauaggcuugugauC...............................................................	1	1	seq
....................uacacuguagaauaggcuugugauA...............................................................	3	1	seq
....................uacacuguagaauaggcuugugaAu...............................................................	23	1	seq
....................uacacuguagaauaggcuugugCuu...............................................................	21	1	seq
....................uacacuguagaauaggcuugugUuu...............................................................	425	1	seq
....................uacacuguagaauaggcuugugaCu...............................................................	6	1	seq
....................uacacuguagaauaggcuugugaGu...............................................................	9	1	seq
....................uacacuguagaauaggcuuguUauu...............................................................	4	1	seq
....................uacacuguagaauaggcuugugUuuu..............................................................	23	1	seq
....................uacacuguagaauaggcuugugaAuu..............................................................	3	1	seq
....................uacacuguagaauaggcuugugCuuu..............................................................	2	1	seq
....................uacacuguagaauaggcuuguUauuu..............................................................	1	1	seq
....................uacacuguagaauaggcuugugauuu..............................................................	5	0	seq
....................uacacuguagaauaggcuugugauuuca............................................................	1	0	seq
....................uacacuguagaauaggcuugugauuucaa...........................................................	1	0	seq
....................uacacuguagaauaggcuugugauuucaau..........................................................	11	0	seq
....................uacacuguagaauaggcuugugauuucaauu.........................................................	30	0	seq
....................uacacuguagaauaggcuugAgauuucaauu.........................................................	1	1	seq
....................uacacuguagaauaggcuugugauuucaaAu.........................................................	1	1	seq
.....................acacAguagaauaggcuu.....................................................................	4	1	seq
.....................acacuguagaauaggcuu.....................................................................	10	0	seq
.....................acacuguagaauaggcuug....................................................................	13	0	seq
.....................Ucacuguagaauaggcuug....................................................................	1	1	seq
.....................acacuguagaauaUgcuug....................................................................	1	1	seq
.....................acacAguagaauaggcuug....................................................................	8	1	seq
.....................acacuguagaauaggcGugu...................................................................	1	1	seq
.....................acacAguagaauaggcuugu...................................................................	457	1	seq
.....................acacuguagaauaggcAugu...................................................................	1	1	seq
.....................acacuguaAaauaggcuugu...................................................................	1	1	seq
.....................acacuguagaauaggcuugu...................................................................	889	0	seq
.....................acacGguagaauaggcuugu...................................................................	1	1	seq
.....................acacuguagaaAaggcuugu...................................................................	2	1	seq
.....................Ucacuguagaauaggcuugu...................................................................	3	1	seq
.....................acacugGagaauaggcuugu...................................................................	1	1	seq
.....................acacuguagaauaUgcuugu...................................................................	3	1	seq
.....................Gcacuguagaauaggcuugu...................................................................	1	1	seq
.....................acacuguagaaAaggcuugug..................................................................	3	1	seq
.....................acacuguaUaauaggcuugug..................................................................	1	1	seq
.....................acacuguagaauaggcuAgug..................................................................	1	1	seq
.....................acacAguagaauaggcuugug..................................................................	483	1	seq
.....................acacuAuagaauaggcuugug..................................................................	1	1	seq
.....................acacuguagGauaggcuugug..................................................................	1	1	seq
.....................Ucacuguagaauaggcuugug..................................................................	5	1	seq
.....................acacuguagaauaggcuuguU..................................................................	23	1	seq
.....................acacuguagaauaggcuugAg..................................................................	1	1	seq
.....................acacuguagaauaggcuuguA..................................................................	1	1	seq
.....................Gcacuguagaauaggcuugug..................................................................	3	1	seq
.....................acacuguagaauaggcuugug..................................................................	1197	0	seq
.....................acacuguagaauaUgcuugug..................................................................	1	1	seq
.....................acacuguagaauaggcuCgug..................................................................	1	1	seq
.....................acacuguagaauaggcuugugC.................................................................	18	1	seq
.....................acacAguagaauaggcuuguga.................................................................	37	1	seq
.....................acacuguagaauaggcuugugU.................................................................	251	1	seq
.....................acacuguagaauaggcuuguga.................................................................	124	0	seq
.....................acacuguagaauaggcuugugG.................................................................	8	1	seq
.....................acacuguagaauaggcuugugaA................................................................	3	1	seq
......................cacuguagaauaggcuugu...................................................................	12	0	seq
......................Uacuguagaauaggcuugu...................................................................	2	1	seq
......................cacuguagaauaggcuugug..................................................................	17	0	seq
......................Uacuguagaauaggcuugug..................................................................	5	1	seq
......................cacuguagaauaggcuugugC.................................................................	2	1	seq
......................cacuguagaauaggcuugugG.................................................................	1	1	seq
......................cacuguagaauaggcuugugU.................................................................	5	1	seq
```

Mature Star

```
gaguuuuagcaauaaccauguacacuguagaauaggcuugugauuucaauuagucaacacaagcucgguucuacagggucaaugggcauugugacuuucaucuccaug
......................cacuguagaauaggcuuguga.................................................................	9	0	seq
.......................acuguagaauaggcuugug..................................................................	3	0	seq
........................cuguagaauaggcuugug..................................................................	8	0	seq
..........................................................aGaagcucgguucuacagg...............................	1	1	seq
...........................................................caagcucgguucuacagg...............................	1	0	seq
...........................................................caagcucgguucuacaggguc............................	59	0	seq
...........................................................caagcucgguucuacaggguca...........................	32	0	seq
...........................................................caagcucgguucuacagggucU...........................	1	1	seq
...........................................................caagcucgguucuacaAggucaa..........................	1	1	seq
...........................................................caagcucggAucuacagggucaa..........................	1	1	seq
...........................................................caagcucgguucuacagggucaa..........................	30	0	seq
...........................................................caagcucgguucuacagggucaaC.........................	1	1	seq
...........................................................caagcucgguucuacagggucaau.........................	14	0	seq
...........................................................caagcucgguucuacagggucaaAg........................	1	1	seq
...........................................................caagcucgguucuacagggucaauU........................	1	1	seq
............................................................aagcucgguucuacaggguca...........................	3	0	seq
..................................................................................ugggcauugugacuuucauc......	1	0	seq
```
