# Supplementary material for: The Small RNA Universe of Capitella teleta
Source: Front Mol Biosci. 2022 Feb 25;9:802814. doi: 10.3389/fmolb.2022.802814 (PMC8915122; doi:10.3389/fmolb.2022.802814)
Supplement: Supplementary file 1 [file DataSheet1.ZIP › Supplement/homologRecovered/CAPTEscaffold_17_2731.pdf]

Provisional ID	:	CAPTEscaffold_17_2731
Score total	:	16324.8
Score for star read(s)	:	3.9
Score for read counts	:	16314
Score for mfe	:	2.2
Score for randfold	:	1.6
Score for cons. seed	:	3
Total read count	:	32011
Mature read count	:	14803
Loop read count	:	0
Star read count	:	17208

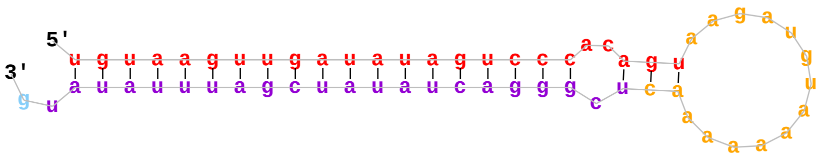

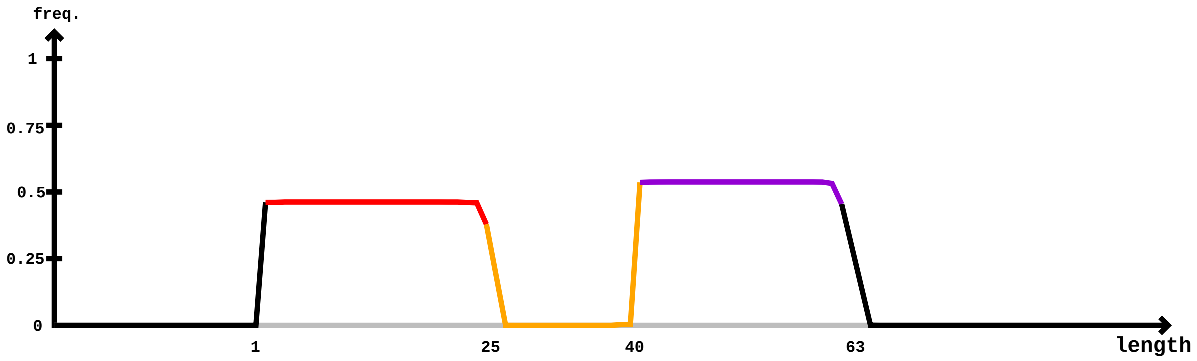


```
                           Mature                                      Star
5'- ucgccuuguuaaggucgacguguaaguugauauagucccacaguaagauguaaaaaaacucgggacuauaucgauuuauaugucgaucuuggcaccgccacucacgggaaauua   -3'   obs
    ucgccuuguuaaggucgacguguaaguugauauagucccacaguaagauguaaaaaaacucgggacuauaucgauuuauaugucgaucuuggcaccgccacucacgggaaauua         exp
    ......(((((((((((((((((((((((((((((((((..(((.............))).)))))))))))))))))))))))))))))))))(((.......)))....... reads   mm   sample
    ..gccuuguuaaggucgacg.............................................................................................. 1      0    seq
    ....................uguaaguugauauagucc............................................................................ 2      0    seq
    ....................uguaaguugauauaguccc........................................................................... 3      0    seq
    ....................uguaaguugauauagucccac......................................................................... 52     0    seq
    ....................uguaaguugauauaAucccac......................................................................... 1      1    seq
    ....................uguaaguugaGauagucccac......................................................................... 1      1    seq
    ....................uguaaguugauauagucccaca........................................................................ 42     0    seq
    ....................uguaaguugauauagucccacG........................................................................ 1      1    seq
    ....................uguaaguugauauagucccacU........................................................................ 3      1    seq
    ....................Aguaaguugauauagucccacag....................................................................... 4      1    seq
    ....................uguaaguugauaCagucccacag....................................................................... 1      1    seq
    ....................uguaaCuugauauagucccacag....................................................................... 1      1    seq
    ....................uUuaaguugauauagucccacag....................................................................... 1      1    seq
    ....................uguaaguugauauagucccacGg....................................................................... 2      1    seq
    ....................Gguaaguugauauagucccacag....................................................................... 3      1    seq
    ....................uguaaguugauauGgucccacag....................................................................... 1      1    seq
    ....................ugAaaguugauauagucccacag....................................................................... 2      1    seq
    ....................uguaaguugauauagucccacag....................................................................... 2509   0    seq
    ....................Nguaaguugauauagucccacag....................................................................... 1      1    seq
    ....................uguaaguugauauagAcccacag....................................................................... 2      1    seq
    ....................uguaaguugauauagucUcacag....................................................................... 3      1    seq
    ....................uguaaguugauGuagucccacag....................................................................... 1      1    seq
    ....................uguaaguugauauagucAcacag....................................................................... 1      1    seq
    ....................uguaaguugauauagucccaAag....................................................................... 1      1    seq
    ....................uguaaguugauauagucccCcag....................................................................... 1      1    seq
    ....................uguaaguugauauaUucccacag....................................................................... 1      1    seq
    ....................uguaaguugauauaguccUacag....................................................................... 21     1    seq
    ....................uguaagAugauauagucccacag....................................................................... 5      1    seq
    ....................uguaaguuAauauagucccacag....................................................................... 5      1    seq
    ....................uguaaguugauauagucccUcag....................................................................... 1      1    seq
    ....................uguaaguCgauauagucccacag....................................................................... 1      1    seq
    ....................uguaaguugauauagucccGcag....................................................................... 1      1    seq
    ....................uguaaguugauauaguccGacagu...................................................................... 1      1    seq
```

Mature Star

```
ucgccuuguuaaggucgacguguaaguugauauagucccacaguaagauguaaaaaaacucgggacuauaucgauuuauaugucgaucuuggcaccgccacucacgggaaauua
....................uguaaguugauaGagucccacagu......................................................................	1	1	seq
....................uguaaguuAauauagucccacagu......................................................................	6	1	seq
....................uguaaguugauauaguAccacagu......................................................................	4	1	seq
....................Aguaaguugauauagucccacagu......................................................................	23	1	seq
....................uguaaguugauauaguUccacagu......................................................................	1	1	seq
....................uguaaguugauauagucccacagG......................................................................	2	1	seq
....................Nguaaguugauauagucccacagu......................................................................	4	1	seq
....................uCuaaguugauauagucccacagu......................................................................	1	1	seq
....................ugAaaguugauauagucccacagu......................................................................	20	1	seq
....................uguaaguugauauagGcccacagu......................................................................	1	1	seq
....................uguaaguugauauagucccacagA......................................................................	9	1	seq
....................uguaaguugauauaguccUacagu......................................................................	10	1	seq
....................uguaaguugauauagAcccacagu......................................................................	9	1	seq
....................uguaaguugauauagucccaUagu......................................................................	1	1	seq
....................uguaaguugaGauagucccacagu......................................................................	3	1	seq
....................Gguaaguugauauagucccacagu......................................................................	3	1	seq
....................uguaaguuCauauagucccacagu......................................................................	1	1	seq
....................uguaaguugauauaguGccacagu......................................................................	2	1	seq
....................uguaaguugauauGgucccacagu......................................................................	2	1	seq
....................uguaaguugauaAagucccacagu......................................................................	1	1	seq
....................uguaagGugauauagucccacagu......................................................................	1	1	seq
....................uAuaaguugauauagucccacagu......................................................................	3	1	seq
....................uguaaguAgauauagucccacagu......................................................................	7	1	seq
....................uguaaguugCuauagucccacagu......................................................................	1	1	seq
....................ugCaaguugauauagucccacagu......................................................................	2	1	seq
....................uguaaguugauGuagucccacagu......................................................................	4	1	seq
....................uguaaguugauauagucGcacagu......................................................................	2	1	seq
....................uguaaguugauauagucccacaCu......................................................................	2	1	seq
....................uguaaguugauauagucUcacagu......................................................................	17	1	seq
....................uguaagAugauauagucccacagu......................................................................	6	1	seq
....................uguaaguugauauaguccAacagu......................................................................	6	1	seq
....................uguaaguCgauauagucccacagu......................................................................	1	1	seq
....................uguaaguugauauagucccacagu......................................................................	11753	0	seq
....................uguaaAuugauauagucccacagu......................................................................	1	1	seq
....................uguaGguugauauagucccacagu......................................................................	4	1	seq
....................uguaaguugNuauagucccacagu......................................................................	1	1	seq
....................uguaaguugauaCagucccacagu......................................................................	4	1	seq
....................Cguaaguugauauagucccacagu......................................................................	2	1	seq
....................uguaaguugauauagucccacGgu......................................................................	3	1	seq
....................uguaaguugauauagucAcacagu......................................................................	17	1	seq
....................uguaaguugUuauagucccacagu......................................................................	2	1	seq
....................uguaaguugauauagucccacagC......................................................................	1	1	seq
....................uguaaguugauauaAucccacagu......................................................................	1	1	seq
....................uguaaguugauauagCccacagu......................................................................	2	1	seq
....................uguaaguugauauUgucccacagu......................................................................	1	1	seq
....................uguaaguugauauagucccUcagu......................................................................	1	1	seq
....................uguGaguugauauagucccacagu......................................................................	6	1	seq
....................uguaaguugaAauagucccacagu......................................................................	4	1	seq
....................uUuaaguugauauagucccacagu......................................................................	4	1	seq
....................uguaaguugauauagucccacaAu......................................................................	1	1	seq
....................uguaaguugauauagucccacagua.....................................................................	11	0	seq
....................uguaaguugauauagucccacaguU.....................................................................	106	1	seq
....................uguaaguugauauagucccacaguaU....................................................................	2	1	seq
....................uguaaguugauauagucccacaguaagaug................................................................	1	0	seq
.....................guaaguugauauagucccacagu......................................................................	1	0	seq
......................uaaguugauauagucccacag.......................................................................	3	0	seq
......................uaaguugauauagucccacagu......................................................................	41	0	seq
.........................................................acucgggacuauaucgauuuau...................................	79	0	seq
.........................................................acucgggacuauauUgauuuau...................................	1	1	seq
.........................................................acucgggacuGuaucgauuuau...................................	1	1	seq
.........................................................Gcucgggacuauaucgauuuau...................................	1	1	seq
.........................................................acucgggacuauaucgauuuauU..................................	1	1	seq
..........................................................cucgggacuauaucgauuuau...................................	5	0	seq
..........................................................cucgggacuauaucAauuuaua..................................	1	1	seq
..........................................................cucgggacuauaucgauuuaua..................................	34	0	seq
..........................................................cucgggacuauaucgauuuauau.................................	3	0	seq
...........................................................ucgggacuauaucgauuua....................................	10	0	seq
...........................................................Acgggacuauaucgauuuau...................................	1	1	seq
...........................................................ucgggacuauaucgauuuau...................................	81	0	seq
...........................................................uAgggacuauaucgauuuaua..................................	1	1	seq
```

Mature   Star

```
ucgccuuguuaaggucgacguguaaguugauauagucccacaguaagauguaaaaaaacucgggacuauaucgauuuauaugucgaucuuggcaccgccacucacgggaaauua
...........................................................ucgggacuauaucgGuuuaua..................................   1       1       seq
...........................................................Ccgggacuauaucgauuuaua..................................   1       1       seq
...........................................................ucgggacCauaucgauuuaua..................................   1       1       seq
...........................................................ucgggacuauaucCauuuaua..................................   1       1       seq
...........................................................ucAggacuauaucgauuuaua..................................   6       1       seq
...........................................................ucgggacuauaAcgauuuaua..................................   1       1       seq
...........................................................ucgggacuauaucgauAuaua..................................   1       1       seq
...........................................................ucgggacuauaucgauuuaua..................................   2376    0       seq
...........................................................ucgggacuauauAgauuuaua..................................   1       1       seq
...........................................................Gcgggacuauaucgauuuaua..................................   7       1       seq
...........................................................ucgggacuauUucgauuuaua..................................   1       1       seq
...........................................................ucgggacuauauUgauuuaua..................................   4       1       seq
...........................................................uUgggacuauaucgauuuaua..................................   4       1       seq
...........................................................ucgggacuauaucgauuuaCa..................................   1       1       seq
...........................................................ucgggacuauGucgauuuaua..................................   2       1       seq
...........................................................Acgggacuauaucgauuuaua..................................   6       1       seq
...........................................................ucgggacuauaucgaAuuaua..................................   1       1       seq
...........................................................ucgggacuauaucAauuuaua..................................   7       1       seq
...........................................................ucgggacuaAaucgauuuaua..................................   2       1       seq
...........................................................ucgAgacuauaucgauuuaua..................................   7       1       seq
...........................................................ucgggacuauGucgauuuauau.................................   1       1       seq
...........................................................ucgggacuaAaucgauuuauau.................................   3       1       seq
...........................................................ucgggacuauaucgauuuGuau.................................   1       1       seq
...........................................................Gcgggacuauaucgauuuauau.................................   18      1       seq
...........................................................ucgCgacuauaucgauuuauau.................................   1       1       seq
...........................................................Ccgggacuauaucgauuuauau.................................   2       1       seq
...........................................................uUgggacuauaucgauuuauau.................................   23      1       seq
...........................................................ucAggacuauaucgauuuauau.................................   17      1       seq
...........................................................ucgggacuauaucgauuuUuau.................................   1       1       seq
...........................................................ucgAgacuauaucgauuuauau.................................   32      1       seq
...........................................................ucgggacuauaucgauGuauau.................................   2       1       seq
...........................................................ucgggacuauaucgauAuauau.................................   3       1       seq
...........................................................ucgggacuauaucgauuuauau.................................   11028   0       seq
...........................................................ucgggacuauaAcgauuuauau.................................   6       1       seq
...........................................................ucgggaUuauaucgauuuauau.................................   1       1       seq
...........................................................ucgggaGuauaucgauuuauau.................................   2       1       seq
...........................................................ucgggacuauaucgauuAauau.................................   2       1       seq
...........................................................ucgggacuauCucgauuuauau.................................   1       1       seq
...........................................................ucgggacuauaucgauuCauau.................................   1       1       seq
...........................................................ucgggacuauaucgauuuaAau.................................   3       1       seq
...........................................................ucgggacuauauGgauuuauau.................................   2       1       seq
...........................................................ucgggacuauauUgauuuauau.................................   21      1       seq
...........................................................ucgggacuauaucgaGuuauau.................................   1       1       seq
...........................................................ucgggGcuauaucgauuuauau.................................   3       1       seq
...........................................................ucgggacCauaucgauuuauau.................................   1       1       seq
...........................................................Ncgggacuauaucgauuuauau.................................   3       1       seq
...........................................................Acgggacuauaucgauuuauau.................................   45      1       seq
...........................................................ucgggacuauaucgauuuauUu.................................   2       1       seq
...........................................................ucgggacAauaucgauuuauau.................................   1       1       seq
...........................................................ucgggacuauaucgauuuCuau.................................   1       1       seq
...........................................................ucgggacuauaucgauuuauaA.................................   1       1       seq
...........................................................ucgggacuGuaucgauuuauau.................................   1       1       seq
...........................................................ucgggacuauauAgauuuauau.................................   6       1       seq
...........................................................ucgggacuauaucAauuuauau.................................   35      1       seq
...........................................................uAgggacuauaucgauuuauau.................................   2       1       seq
...........................................................ucggAacuauaucgauuuauau.................................   1       1       seq
...........................................................ucgggacuauaucgauuuauGu.................................   1       1       seq
...........................................................ucgggacuauaucgaAuuauau.................................   2       1       seq
...........................................................ucgggacuauaCcgauuuauau.................................   4       1       seq
...........................................................ucggUacuauaucgauuuauau.................................   3       1       seq
...........................................................ucgggUcuauaucgauuuauau.................................   2       1       seq
...........................................................ucgggacuauUucgauuuauau.................................   1       1       seq
...........................................................ucgggacuauaucgGuuuauau.................................   1       1       seq
...........................................................ucgAgacuauaucgauuuauaug................................   1       1       seq
...........................................................ucgggacuauauUgauuuauaug................................   1       1       seq
...........................................................ucgggacuauauAgauuuauaug................................   2       1       seq
...........................................................ucgggaAuauaucgauuuauaug................................   1       1       seq
...........................................................ucgggacuauaucgauuuauauU................................   42      1       seq
...........................................................uGgggacuauaucgauuuauaug................................   1       1       seq
...........................................................Gcgggacuauaucgauuuauaug................................   3       1       seq
```

```
                    Mature                                  Star
ucgccuuguuaaggucgacguguaaguugauauagucccacaguaagauguaaaaaaaacucgggacuauaucgauuuauaugucgaucuuggcaccgccacucacgggaaauua
............................................................ucgCgacuauaucgauuuauaug................................ 1    1  seq
............................................................ucgggacuauaucgaGuuauaug................................ 1    1  seq
............................................................ucgggacuauaucAauuuauaug................................ 1    1  seq
............................................................ucgggacuauaucgauuuauaug................................ 782  0  seq
............................................................ucgggacuauaucgauuuauauA................................ 66   1  seq
............................................................ucgggaAuauaucgauuuauaugu............................... 1    1  seq
............................................................ucgggUcuauaucgauuuauaugu............................... 1    1  seq
............................................................ucgggacuauaucgauuuaGaugu............................... 1    1  seq
............................................................ucgggacuauGucgauuuauaugu............................... 2    1  seq
............................................................ucgggacuauaucAauuuauaugu............................... 1    1  seq
............................................................ucgggacuauaucgauuuauaugC............................... 1    1  seq
............................................................ucgggacuauaucgauCuauaugu............................... 2    1  seq
............................................................uUgggacuauaucgauuuauaugu............................... 2    1  seq
............................................................ucgggacuauaucgauuuauaugu............................... 1831 0  seq
............................................................ucgggacuGuaucgauuuauaugu............................... 1    1  seq
............................................................Acgggacuauaucgauuuauaugu............................... 6    1  seq
............................................................ucgAgacuauaucgauuuauaugu............................... 2    1  seq
............................................................ucgggacuauaucgaAuuauaugu............................... 1    1  seq
............................................................ucgggacuauaucgauuuaCaugu............................... 1    1  seq
............................................................ucAggacuauaucgauuuauaugu............................... 6    1  seq
............................................................ucgggacuauauAgauuuauaugu............................... 2    1  seq
............................................................Gcgggacuauaucgauuuauaugu............................... 3    1  seq
............................................................Ccgggacuauaucgauuuauaugu............................... 2    1  seq
............................................................ucgggacuauaGcgauuuauaugu............................... 1    1  seq
............................................................ucgggacuauaucgauuuauauAu............................... 1    1  seq
............................................................ucgggGcuauaucgauuuauaugu............................... 1    1  seq
............................................................ucgggacuauaucgGuuuauaugu............................... 1    1  seq
............................................................ucgggacuauaucgauuuauaugA............................... 432  1  seq
............................................................ucgggacuauauUgauuuauaugu............................... 1    1  seq
............................................................ucgggacuauaucgauuuauauguA.............................. 6    1  seq
............................................................ucgggacuauaucgauuuauauguU.............................. 6    1  seq
.............................................................cCggacuauaucgauuuaua.................................. 1    1  seq
.............................................................cgggacuauaucgauuuaua.................................. 3    0  seq
.............................................................cgggacuauaucgauuuauau................................. 27   0  seq
.............................................................cgggacuauaucgauuuauaug................................ 6    0  seq
.............................................................cgggacuauaucgauuuauaugu............................... 6    0  seq
..............................................................gggacuauaucgauuuauau................................. 5    0  seq
..............................................................gggacuauaucgauuuauaug................................ 1    0  seq
...............................................................ggacuauaucgauuuauaug................................ 1    0  seq
...............................................................ggacuauaucgauuuauaugu............................... 3    0  seq
```
