# Supplementary material for: The Small RNA Universe of Capitella teleta
Source: Front Mol Biosci. 2022 Feb 25;9:802814. doi: 10.3389/fmolb.2022.802814 (PMC8915122; doi:10.3389/fmolb.2022.802814)
Supplement: Supplementary file 1 [file DataSheet1.ZIP › Supplement/homologRecovered/CAPTEscaffold_876_29818.pdf]

Provisional ID	:	CAPTEscaffold_876_29818
Score total	:	2334.6
Score for star read(s)	:	3.9
Score for read counts	:	2324.4
Score for mfe	:	1.7
Score for randfold	:	1.6
Score for cons. seed	:	3
Total read count	:	4571
Mature read count	:	4487
Loop read count	:	0
Star read count	:	84

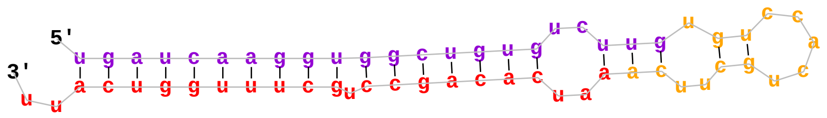

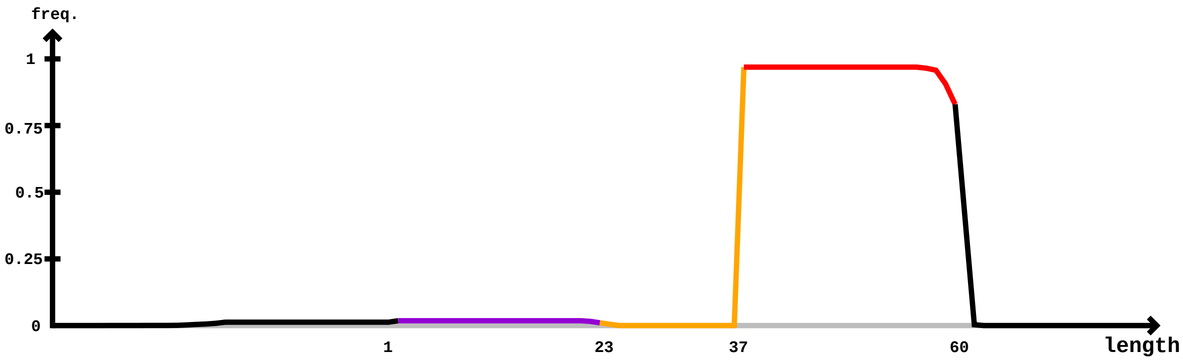

Star Mature

```
5'- cugcucuuccucacccucauugagaaccugcacuugaucaaggugggcugugucuuguguccacugcuucaaaucacagccugcuuuggucauuugcugguuuuucaucaccuu  -3'   obs
    cugcucuuccucacccucauugagaaccugcacuugaucaaggugggcugugucuuguguccacugcuucaaaucacagccugcuuuggucauuugcugguuuuucaucaccuu         exp
    cugcucuuccucacccucauugagaaccugcacuugaucaaggugggcugugucuuguguccacugcuucaaaucacagccugcuuuggucauuugcugguuuuucaucaccuu         known
    .....................(((((((.(((..(((((((((((((((((..(((.((.....))..)))..))))))).))))))))))..))).)))))))......... reads  mm  sample

    ....ucuuccucacccucauugagaaccugcacu............................................................................... 1     0   seq
    ........ccucacccucauugagaaccugcacu............................................................................... 1     0   seq
    ...........cacccucauugagaaccugcacu............................................................................... 2     0   seq
    ............acccucauugagaaccugcacu............................................................................... 7     0   seq
    .............cccucauugagaaccugcacu............................................................................... 9     0   seq
    ..............ccucauugagaaccugcacu............................................................................... 7     0   seq
    ...............cucauugagaaccugcacu............................................................................... 12    0   seq
    ................ucauugagaaccugcacu............................................................................... 20    0   seq
    ..................................ugaucaaggugggcugugucu........................................................... 11    0   seq
    ..................................ugaucaaggugggcugugucuu.......................................................... 25    0   seq
    ..................................ugaucaaggugggcugugucuAg......................................................... 1     1   seq
    ..................................ugaucaaggAggcugugucuug......................................................... 1     1   seq
    ..................................Agaucaaggugggcugugucuug......................................................... 1     1   seq
    ..................................ugaucaaggugggcugugucuug......................................................... 43    0   seq
    ..................................ugaucaaggugggcugugucuugA........................................................ 2     1   seq
    ......................................................................aaucacagccugcuuuggu........................ 19    0   seq
    ......................................................................aaucacagccugcuuugguc....................... 35    0   seq
    ......................................................................aaucacagccugcuuugguAa...................... 2     1   seq
    ......................................................................aaucacagccugcuuuggguca...................... 233   0   seq
    ......................................................................aaucacagccugcuuAgguca...................... 1     1   seq
    ......................................................................aauUacagccugcuuuggguca...................... 1     1   seq
    ......................................................................aaucacagccugcuuuggucau..................... 349   0   seq
    ......................................................................Gaucacagccugcuuuggucau..................... 1     1   seq
    ......................................................................aaucacagccugcuuuAgucau..................... 2     1   seq
    ......................................................................aaucacagccugcuuugguGau..................... 1     1   seq
    ......................................................................Uaucacagccugcuuuggucau..................... 1     1   seq
    ......................................................................aaucacagccugAuuuggucauu.................... 3     1   seq
    ......................................................................aaucacagccugcuuuggucauA.................... 13    1   seq
    ......................................................................Gaucacagccugcuuuggucauu.................... 1     1   seq
    ......................................................................aaucacagccugcuAuggucauu.................... 1     1   seq
    ......................................................................aaucacagccugcuuCggucauu.................... 2     1   seq
```

```
                                  Star                               Mature
cugcucuuccucacccucauugagaaccugcacuugaucaagguggcugugucuuguguccacugcuucaaaucacagccugcuuuggucauuugcugguuuuucaucaccuu
......................................................................aauAacagccugcuuuggucauu....................	3	1	seq
......................................................................aaucacagcAugcuuuggucauu....................	1	1	seq
......................................................................aaucacagccugGuuuggucauu....................	1	1	seq
......................................................................aaucUcagccugcuuuggucauu....................	1	1	seq
......................................................................aaAcacagccugcuuuggucauu....................	7	1	seq
......................................................................aaucacagccugcuuugguUauu....................	1	1	seq
......................................................................aaucacagccugcuuugUucauu....................	2	1	seq
......................................................................aaucacagccugcCuuggucauu....................	2	1	seq
......................................................................aaucacagccuUcuuuggucauu....................	2	1	seq
......................................................................Naucacagccugcuuuggucauu....................	3	1	seq
......................................................................aaucacagccugcuuuggucGuu....................	1	1	seq
......................................................................aaucacagccugcuuuggucauG....................	5	1	seq
......................................................................aaucacagccugcuuuggucaAu....................	3	1	seq
......................................................................aaucacagccugcuuuggucauC....................	3	1	seq
......................................................................aaucacagccugcuuugAucauu....................	1	1	seq
......................................................................aaucGcagccugcuuuggucauu....................	1	1	seq
......................................................................aaucacagccugcuuuAgucauu....................	11	1	seq
......................................................................aaucacagAcugcuuuggucauu....................	1	1	seq
......................................................................aaucaUagccugcuuuggucauu....................	1	1	seq
......................................................................aaucaGagccugcuuuggucauu....................	1	1	seq
......................................................................aaucacagccugcuuugguAauu....................	3	1	seq
......................................................................aaucacagccugcuuuggucauu....................	3706	0	seq
......................................................................aaucacagccAgcuuuggucauu....................	2	1	seq
......................................................................aaucacagccugcuuGggucauu....................	1	1	seq
......................................................................aaucacagccugcuuAggucauu....................	2	1	seq
......................................................................aaucacagccugcuuuggucauuu...................	32	0	seq
......................................................................aaucacagccugcuuuggucauuC...................	5	1	seq
......................................................................aaucacagccugcuuuAgucauuu...................	1	1	seq
......................................................................aaucacagccugcuuuggucauuA...................	4	1	seq
......................................................................aaucacagccugcuuuggucauuuU..................	14	1	seq
......................................................................aaucacagccugcuuuggucauuAg..................	1	1	seq
```
