# Supplementary material for: The Small RNA Universe of Capitella teleta
Source: Front Mol Biosci. 2022 Feb 25;9:802814. doi: 10.3389/fmolb.2022.802814 (PMC8915122; doi:10.3389/fmolb.2022.802814)
Supplement: Supplementary file 1 [file DataSheet1.ZIP › Supplement/homologRecovered/CAPTEscaffold_26_2058.pdf]

Provisional ID	:	CAPTEscaffold_26_2058
Score total	:	6.7
Score for star read(s)	:	3.9
Score for read counts	:	0.6
Score for mfe	:	1.4
Score for randfold	:	-2.2
Score for cons. seed	:	3
Total read count	:	13
Mature read count	:	11
Loop read count	:	0
Star read count	:	2

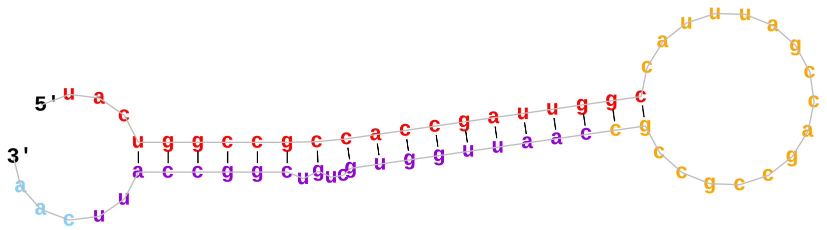

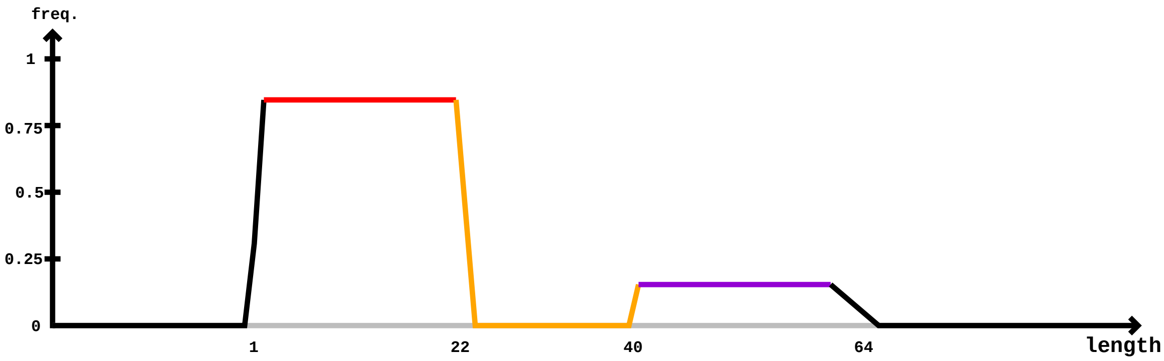

Mature **Star**

```
5'- ccggagccgccgucgauauguacuggccgccaccgauuggccauuuagccagccgccgccaauuggugcugucggccauucaauaucagccgaucacugucaacagcagcu   -3'    obs
    ccggagccgccgucgauauguacuggccgccaccgauuggccauuuagccagccgccgccaauuggugcugucggccauucaauaucagccgaucacugucaacagcagcu          exp
    .(((.....)))..(((((....((((((((((((((((((................)))))))))))..).))))))....)))))........((((.....))))..   reads      mm         sample
    ...................guacuggccgccaccgauuggc......................................................................   4          0          seq
    ....................uacuggccgccaccgauuggc......................................................................   7          0          seq
    ...........................................................caauuggugcugucggccaGu...............................   2          1          seq
```
