# Supplementary material for: The Small RNA Universe of Capitella teleta
Source: Front Mol Biosci. 2022 Feb 25;9:802814. doi: 10.3389/fmolb.2022.802814 (PMC8915122; doi:10.3389/fmolb.2022.802814)
Supplement: Supplementary file 1 [file DataSheet1.ZIP › Supplement/homologRecovered/CAPTEscaffold_508_21982.pdf]

Provisional ID	:	CAPTEscaffold_508_21982
Score total	:	5.9
Score for star read(s)	:	-1.3
Score for read counts	:	0
Score for mfe	:	2.6
Score for randfold	:	1.6
Score for cons. seed	:	3
Total read count	:	186
Mature read count	:	130
Loop read count	:	0
Star read count	:	56

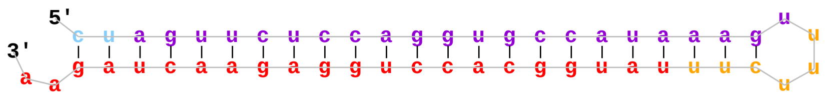

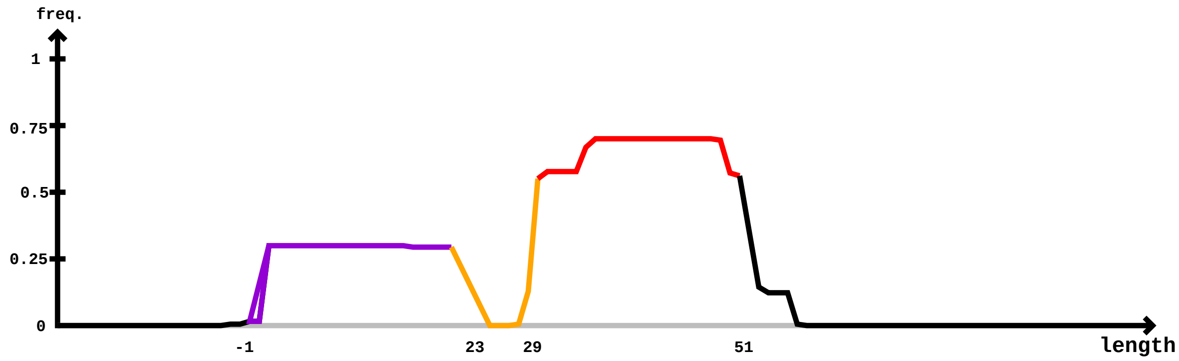

Star **Mature**

```
5'- acagagaacaaccaaauucuaguucuccaggugccauaaaguuuucuuuauggcaccuggagaacuagaauuagaaaaucaaauuuugccaauuuuuauuggcaaaauuuga   -3'   obs
    acagagaacaaccaaauucuaguucuccaggugccauaaaguuuucuuuauggcaccuggagaacuagaauuagaaaaucaaauuuugccaauuuuuauuggcaaaauuuga         exp
    ..............(((((((((((((((((((((((((((....)))))))))))))))))))))))))))......(((((((((((((((....)))))))))))))))  reads   mm   sample
    ................uucuaguucuccaggugcc.............................................................................  1       0    seq
    ..................cuaguucuccaggugccauaaagu......................................................................  2       0    seq
    ....................aguucuccaggugccauaaagu......................................................................  53      0    seq
    ..............................................uuuGuggcaccuggagaacua.............................................  1       1    seq
    ...............................................uuGuggcaccuggagaacuag............................................  23      1    seq
    ................................................uGuggcaccuggagaacuaga...........................................  2       1    seq
    ................................................uGuggcaccuggagaacuagaa..........................................  76      1    seq
    ................................................uGuggcaccuggagaacuagaau.........................................  1       1    seq
    .................................................Guggcaccuggagaacuagaau.........................................  1       1    seq
    .................................................Guggcaccuggagaacuagaauu........................................  4       1    seq
    .....................................................caccuggagaacuagaauuaga.....................................  15      0    seq
    .....................................................caccuggagaacuagaauuagU.....................................  1       1    seq
    .....................................................caccuggagaacuagaauuagaa....................................  1       0    seq
    ......................................................accuggagaacuagaauuaga.....................................  6       0    seq
```
