# Supplementary material for: The Small RNA Universe of Capitella teleta
Source: Front Mol Biosci. 2022 Feb 25;9:802814. doi: 10.3389/fmolb.2022.802814 (PMC8915122; doi:10.3389/fmolb.2022.802814)
Supplement: Supplementary file 1 [file DataSheet1.ZIP › Supplement/homologRecovered/CAPTEscaffold_746_25427.pdf]

Provisional ID	:	CAPTEscaffold_746_25427
Score total	:	5.2
Score for star read(s)	:	-1.3
Score for read counts	:	0
Score for mfe	:	1.9
Score for randfold	:	1.6
Score for cons. seed	:	3
Total read count	:	3901
Mature read count	:	3898
Loop read count	:	0
Star read count	:	3

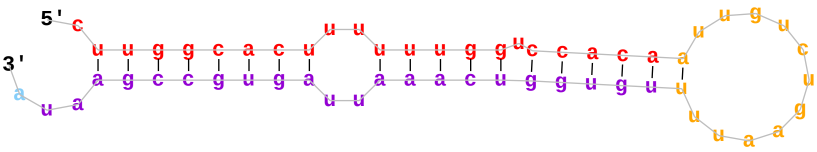

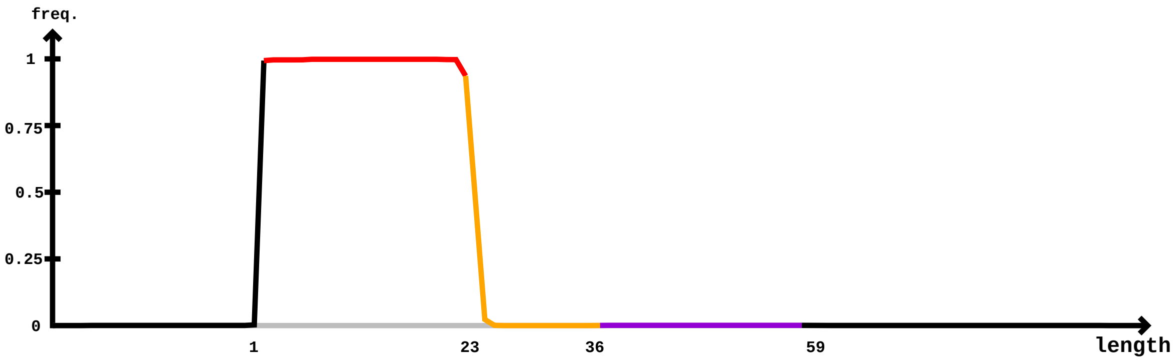

Mature Star

```
5'- gaaaggugguugucagguuacuuggcacuuuuuugguccacaauugucugaauuuuguggucaaauuagugccgaauaacuuggcagugcuuugcuuucuauugacugaugu   -3'    obs
    gaaaggugguugucagguuacuuggcacuuuuuugguccacaauugucugaauuuuguggucaaauuagugccgaauaacuuggcagugcuuugcuuucuauugacugaugu          exp
    (((((..((((((((((((.((((((((..(((((.((((((...........))))))))))..)))))))).))))))))))..)))...))))).............    reads    mm    sample
    ..aaggugguugucagguua............................................................................................    2    0    seq
    ...................acuuggcacuuuuuugguccaca......................................................................    7    0    seq
    ...................acuuggcacuuuuuugguccacaa.....................................................................    1    0    seq
    ....................cuuggcacuuuuuuggucc.........................................................................    4    0    seq
    ....................cuuggcacuuuuuuggucca........................................................................    1    0    seq
    ....................cuuggcacuAuuuugguccac.......................................................................    1    1    seq
    ....................cuuggcacuuuuuuAguccac.......................................................................    1    1    seq
    ....................cuuAgcacuuuuuugguccac.......................................................................    1    1    seq
    ....................cuuggcacuuuuuugguccac.......................................................................    236    0    seq
    ....................cuuUgcacuuuuuugguccaca......................................................................    1    1    seq
    ....................NuuggcacuuuuuugguccacA......................................................................    1    1    seq
    ....................cuuggAacuuuuuugguccaca......................................................................    1    1    seq
    ....................Auuggcacuuuuuugguccaca......................................................................    2    1    seq
    ....................cuCggcacuuuuuugguccaca......................................................................    2    1    seq
    ....................cuuggcacuuuuuugguccaca......................................................................    2539    0    seq
    ....................cuuggcacCuuuuugguccaca......................................................................    5    1    seq
    ....................cuuggcacuuuuuugguccaAa......................................................................    1    1    seq
    ....................cuuggcacGuuuuugguccaca......................................................................    1    1    seq
    ....................cuuggcacuuuuuuggucAaca......................................................................    1    1    seq
    ....................cuuggcacuuuuuugguccaGa......................................................................    1    1    seq
    ....................cuuggcacuuuAuugguccaca......................................................................    2    1    seq
    ....................cuuggUacuuuuuugguccaca......................................................................    2    1    seq
    ....................cuuggcacuAuuuugguccaca......................................................................    3    1    seq
    ....................cuuggcacuuuuuuggucGaca......................................................................    1    1    seq
    ....................cuuAgcacuuuuuugguccaca......................................................................    3    1    seq
    ....................cuuggcacuuuuuugguccacG......................................................................    2    1    seq
    ....................cuuggcacAuuuuugguccaca......................................................................    1    1    seq
    ....................cuuggcacuuuuuugAuccaca......................................................................    1    1    seq
    ....................cuuggcacuuuuuugUuccaca......................................................................    1    1    seq
    ....................cuuggcacuuuuuuggAccaca......................................................................    5    1    seq
    ....................cuuggcacuuuuuuCguccaca......................................................................    1    1    seq
    ....................cuuggcGcuuuuuugguccaca......................................................................    1    1    seq
    ....................cuuggcacuuAuuugguccaca......................................................................    1    1    seq
```

```
                    Mature                  Star
gaaaggugguugucagguuacuuggcacuuuuuugguccacaauugucugaauuuuguggucaaauuagugccgaauaacuuggcagugcuuugcuuucuauugacugaugu
....................cuuggcacuuuuuugguccacU......................................................................    16    1    seq
....................cuuggcacuuuuAugguccaca......................................................................    1     1    seq
....................cuuggcacuuuuuuggucUaca......................................................................    8     1    seq
....................cAuggcacuuuuuugguccaca......................................................................    2     1    seq
....................cuuggcacuuuuuuAguccaca......................................................................    2     1    seq
....................Uuuggcacuuuuuugguccacaa.....................................................................    1     1    seq
....................cuuggcacuuuuuugguccacaC.....................................................................    1     1    seq
....................cuuggcacuAuuuugguccacaa.....................................................................    1     1    seq
....................cuuCgcacuuuuuugguccacaa.....................................................................    1     1    seq
....................cuuAgcacuuuuuugguccacaa.....................................................................    1     1    seq
....................cuuggcacuuuuuugguccaGaa.....................................................................    1     1    seq
....................cuuggcacuuuuuugguccacaU.....................................................................    11    1    seq
....................cuuggcacuuuuuuggucUacaa.....................................................................    2     1    seq
....................cuuggcacuuuuuuggAccacaa.....................................................................    1     1    seq
....................cuuggcacuuuuuugguccacaa.....................................................................    913   0    seq
....................cuuggcacuuuuuugguccacaaA....................................................................    35    1    seq
....................cuuggcacuuuuuugguccacaaC....................................................................    6     1    seq
....................cuuggcacuuuuuugguccacaau....................................................................    41    0    seq
....................cuuggcacuuuuuugguccacaUu....................................................................    1     1    seq
....................cuuggcacuuuuuugguccacaaAu...................................................................    2     1    seq
....................cuuggcacuuuuuugguccacaauu...................................................................    1     0    seq
.....................Cuggcacuuuuuugguccaca......................................................................    1     1    seq
.....................uuggcacuuuuuugguccaca......................................................................    4     0    seq
.....................uuggcacuuuuuugguccacaa.....................................................................    2     0    seq
.....................uuggcacuuuuuugguccacaau....................................................................    3     0    seq
........................gcacuuuuuugguccaca......................................................................    1     0    seq
.........................cacuuuuuugguccacaa.....................................................................    8     0    seq
.......................................................uguggucaaauuagugccgaau...................................    2     0    seq
........................................................guggucaaauuagugccgaau...................................    1     0    seq
..............................................................................acuuggcagugcuuugcuuucuauugacug....    1     0    seq
```
