# Supplementary material for: The Small RNA Universe of Capitella teleta
Source: Front Mol Biosci. 2022 Feb 25;9:802814. doi: 10.3389/fmolb.2022.802814 (PMC8915122; doi:10.3389/fmolb.2022.802814)
Supplement: Supplementary file 1 [file DataSheet1.ZIP › Supplement/confident/CAPTEscaffold_488_22757.pdf]

Provisional ID : CAPTEscaffold_488_22757
Score total : 3813.8
Score for star read(s) : 3.9
Score for read counts : 3807.5
Score for mfe : 1.4
Score for randfold : 1.6
Score for cons. seed : -0.6
Total read count : 7480
Mature read count : 3847
Loop read count : 0
Star read count : 3633

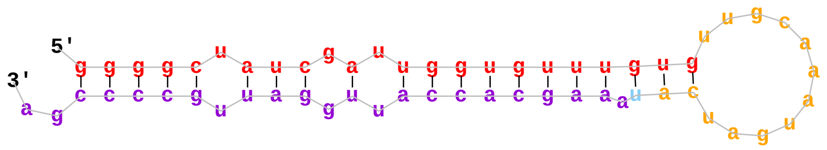

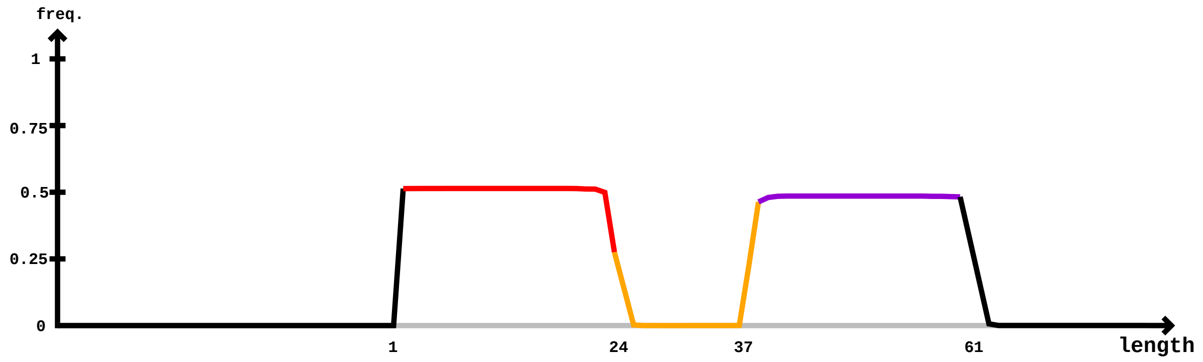


```
                                                   Mature                                Star
5'- cauccuaauuuucggagauauuugucucaguuuuggggcuaucgauugguguuuguguugcaaaugaucauaaagcaccauuggauugccccgacauuggaaucugcuaucauu  -3'   obs
    cauccuaauuuucggagauauuugucucaguuuuggggcuaucgauugguguuuguguugcaaaugaucauaaagcaccauuggauugccccgacauuggaaucugcuaucauu         exp
    ..(((........)))((((....((.((((.(((((((.(((.(.(((((((((((..........))).)))))))).).))).))))))).))))))......))))...  reads      mm        sample
    ..................................ggggcuaucgauuggugu..............................................................  2          0         seq
    ..................................ggggcuaucgauugguguu.............................................................  13         0         seq
    ..................................ggggcuaucgauugguguuu............................................................  3          0         seq
    ..................................Agggcuaucgauugguguuug...........................................................  1          1         seq
    ..................................ggggcuaucgauugguguuuA...........................................................  1          1         seq
    ..................................ggggcuaucgauAgguguuug...........................................................  1          1         seq
    ..................................ggggcuaucgauugguguuug...........................................................  89         0         seq
    ..................................ggggGuaucgauugguguuugu..........................................................  1          1         seq
    ..................................ggggcuaucgauuggugAuugu..........................................................  2          1         seq
    ..................................ggggcuauUgauugguguuugu..........................................................  4          1         seq
    ..................................Ngggcuaucgauugguguuugu..........................................................  1          1         seq
    ..................................ggggcuaucgauuAguguuugu..........................................................  5          1         seq
    ..................................ggggcuaucgauugguguuCgu..........................................................  1          1         seq
    ..................................ggggcuaucgauAgguguuugu..........................................................  1          1         seq
    ..................................ggggcuaucgauugguguuAgu..........................................................  1          1         seq
    ..................................ggggcuaucgauugguguuuCu..........................................................  1          1         seq
    ..................................ggggcuauAgauugguguuugu..........................................................  1          1         seq
    ..................................ggggcuaucgauugguguuugu..........................................................  1645       0         seq
    ..................................ggggcuaucAauugguguuugu..........................................................  5          1         seq
    ..................................ggggcuaucgauuggAguuugu..........................................................  1          1         seq
    ..................................ggggcuaucgauugguguuugG..........................................................  1          1         seq
    ..................................Agggcuaucgauugguguuugu..........................................................  8          1         seq
    ..................................ggggcuaucgauugguguuugA..........................................................  1          1         seq
    ..................................gAggcuaucgauugguguuugug.........................................................  5          1         seq
    ..................................Ugggcuaucgauugguguuugug.........................................................  2          1         seq
    ..................................ggggcuaucgauugguguuAgug.........................................................  1          1         seq
    ..................................ggggcuaucgauuggugugAugug........................................................  1          1         seq
    ..................................ggggcuaucgauugguguuuguU.........................................................  4          1         seq
    ..................................ggggcuaucgauugUuguuugug.........................................................  1          1         seq
    ..................................ggggcuaucCauugguguuugug.........................................................  1          1         seq
    ..................................ggggcuaucgauugguguCugug.........................................................  1          1         seq
    ..................................ggggcuaucgauuggAguuugug.........................................................  3          1         seq
    ..................................Agggcuaucgauugguguuugug.........................................................  14         1         seq
```

Mature   Star

```
cauccuaauuuucggagauauuugucucaguuuuggggcuaucgauugguguuuguguugcaaaugaucauaaagcaccauuggauugccccgacauuggaaucugcuaucauu
..................................ggggcuaucgauugguguuuguA.........................................................	4	1	seq
..................................ggggcuaucgauugguguuugug.........................................................	1944	0	seq
..................................ggCgcuaucgauugguguuugug.........................................................	1	1	seq
..................................ggggcuaucgaAugguguuugug.........................................................	1	1	seq
..................................ggggcuaucgauugguAuuugug.........................................................	1	1	seq
..................................ggAgcuaucgauugguguuugug.........................................................	2	1	seq
..................................ggggcuaucAauugguguuugug.........................................................	10	1	seq
..................................ggggcuauAgauugguguuugug.........................................................	1	1	seq
..................................ggggcuaucgauugguguuCgug.........................................................	1	1	seq
..................................ggggcuauUgauugguguuugug.........................................................	6	1	seq
..................................ggggcuaucgauuAguguuugug.........................................................	6	1	seq
..................................ggggcuaucgauugguguuuAug.........................................................	1	1	seq
..................................ggggcuaucgauugguguuugugC........................................................	1	1	seq
..................................ggggcuaucgauugguguuugugA........................................................	6	1	seq
..................................ggggcuaucgauugguguuugugu........................................................	17	0	seq
..................................ggggcuaucgauugguguuugugG........................................................	2	1	seq
..................................ggggcuaucgauugguguuuguguA.......................................................	8	1	seq
..................................ggggcuaucgauugguguuuguguu.......................................................	8	0	seq
..................................ggggcuaucgauugguguuuguguugc.....................................................	1	0	seq
...................................gggcuaucgauuAguguuugug.........................................................	1	1	seq
...................................gggcuaucgauugguguuugug.........................................................	1	0	seq
....................................ggcuaucgauugguguuugu..........................................................	1	0	seq
.......................................uaucgauugguguuugug.........................................................	1	0	seq
................................................................ugaucauaaagcaccauuggauugccccga....................	1	0	seq
.....................................................................auaaagcaccauuggauugccccgaU...................	1	1	seq
......................................................................uaaagcaccauuggauugc.........................	6	0	seq
......................................................................uaaagcaccauuggauugccc.......................	5	0	seq
......................................................................uaaagcaccauAggauugccc.......................	1	1	seq
......................................................................uaaagcaccauuggauugcccc......................	6	0	seq
......................................................................uaaagcaccauuggauugccccg.....................	20	0	seq
......................................................................uaaagcaccauuggauugccccgG....................	7	1	seq
......................................................................uaaagcaccauuggaAugccccga....................	1	1	seq
......................................................................uaaagcaccaAuggauugccccga....................	1	1	seq
......................................................................uaaagcaccauuggauugccccga....................	1534	0	seq
......................................................................uaaagcaccauuggauugccccAa....................	3	1	seq
......................................................................uaaagcaccauuggauugccAcga....................	1	1	seq
......................................................................uaaagcaccauuggauugcccAga....................	1	1	seq
......................................................................uaaagcaccaGuggauugccccga....................	1	1	seq
......................................................................uaaagcaccauuggauugccccgC....................	8	1	seq
......................................................................uaaagcacUauuggauugccccga....................	1	1	seq
......................................................................Aaaagcaccauuggauugccccga....................	7	1	seq
......................................................................uaaagcaccauAggauugccccga....................	2	1	seq
......................................................................uaaagcaccauuggauugccccgU....................	4	1	seq
......................................................................uaaagcaccauuggauAgccccga....................	1	1	seq
......................................................................uaaagcaccauuAgauugccccga....................	5	1	seq
......................................................................uaaagcaccUuuggauugccccga....................	1	1	seq
......................................................................uaaagcaccauuggauugcccUga....................	1	1	seq
......................................................................uaaagcGccauuggauugccccga....................	1	1	seq
......................................................................uaaagcaccauuggauugccUcga....................	2	1	seq
......................................................................uaaagcacAauuggauugccccga....................	4	1	seq
......................................................................uaaagcaccGuuggauugccccga....................	5	1	seq
......................................................................uaaagcaccauuggauugccccCa....................	2	1	seq
......................................................................uaaagcaccauuggauugAcccga....................	1	1	seq
......................................................................uaaagcaccauuggauugccccgCc...................	1	1	seq
......................................................................uaaagcaccauuggauugccccgaA...................	13	1	seq
......................................................................uaaagcaccauuggauugccccgaAa..................	39	1	seq
.......................................................................aaagcaccauuggauugc.........................	1	0	seq
.......................................................................aaagcaccGuuggauugccc.......................	2	1	seq
.......................................................................aaagcaccGuuggauugcccc......................	2	1	seq
.......................................................................aaagcaccGuuggauugccccg.....................	2	1	seq
.......................................................................aaagcaccauuggauugccccg.....................	1	0	seq
.......................................................................aaagcaccUuuggauugccccga....................	1	1	seq
.......................................................................Gaagcaccauuggauugccccga....................	4	1	seq
.......................................................................Naagcaccauuggauugccccga....................	2	1	seq
.......................................................................aaagcaccauuAgauugccccga....................	1	1	seq
.......................................................................aaagcaccauuggauugccccgU....................	1	1	seq
.......................................................................aaagcaccauuggauAgccccga....................	1	1	seq
.......................................................................aaagcaccGuuggauugccccga....................	1674	1	seq
.......................................................................aaagcaccauuggauugccccga....................	84	0	seq
.......................................................................Caagcaccauuggauugccccga....................	2	1	seq
```

```
                                  Mature                                 Star
cauccuaauuuucggagauauuugucucaguuuuggggcuaucgauugguguuuguguugcaaaugaucauaaagcaccauuggauugccccgacauuggaaucugcuaucauu
.......................................................................aaagcaccauuggauugccccgaAa..................   4    1    seq
........................................................................aagcaccauuggauugccccg.....................   1    0    seq
........................................................................aagcaccauuggauugcccUga....................   1    1    seq
........................................................................aagcaccauAggauugccccga....................   2    1    seq
........................................................................aagcaccauuggauugccccga....................   112  0    seq
........................................................................Uagcaccauuggauugccccga....................   1    1    seq
........................................................................aagcaccauuggauugccccAa....................   3    1    seq
........................................................................aagcaccauuggauugccccgaA...................   1    1    seq
........................................................................aagcaccauuggauugccccgaAa..................   3    1    seq
.........................................................................agcaccauuggauugccccg.....................   1    0    seq
.........................................................................Ggcaccauuggauugccccga....................   1    1    seq
.........................................................................agcaccauuggauugccccga....................   29   0    seq
.........................................................................agcaccauuggauugccccgaA...................   2    1    seq
.........................................................................agcaccauuggauugccccgaU...................   1    1    seq
.........................................................................agcaccauuggauugccccgaAa..................   1    1    seq
..........................................................................gcaccauuggauugccccgaU...................   5    1    seq
..........................................................................gcaccauuggauugccccgaA...................   1    1    seq
..............................................................................................cauuggaaucugcuauca..   1    0    seq
..............................................................................................cauuggaaucugcuaucauu   1    0    seq
```
