# Supplementary material for: The Small RNA Universe of Capitella teleta
Source: Front Mol Biosci. 2022 Feb 25;9:802814. doi: 10.3389/fmolb.2022.802814 (PMC8915122; doi:10.3389/fmolb.2022.802814)
Supplement: Supplementary file 1 [file DataSheet1.ZIP › Supplement/confident/CAPTEscaffold_488_22743.pdf]

Provisional ID	:	CAPTEscaffold_488_22743
Score total	:	20828.9
Score for star read(s)	:	3.9
Score for read counts	:	20821.9
Score for mfe	:	2.1
Score for randfold	:	1.6
Score for cons. seed	:	-0.6
Total read count	:	40853
Mature read count	:	40568
Loop read count	:	0
Star read count	:	285

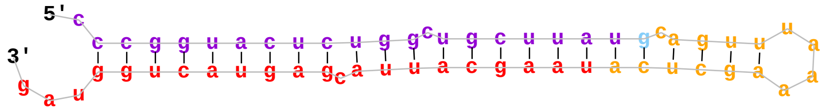

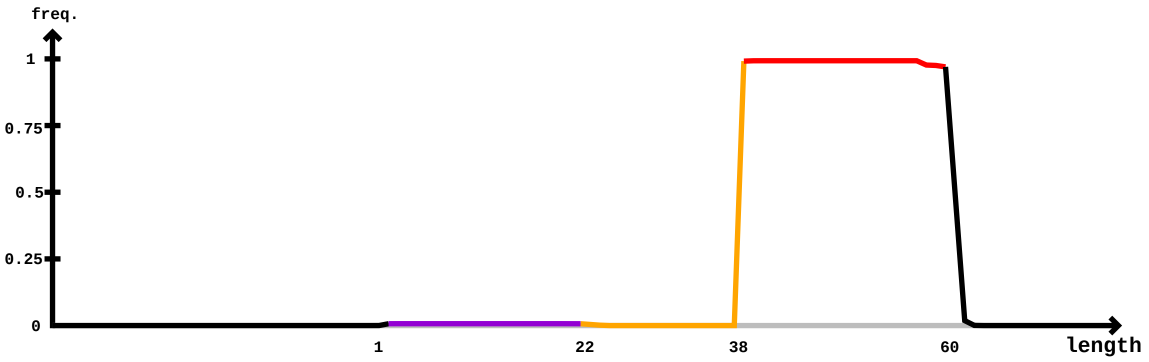


```
                                    Star                               Mature
5'- gacuuagucagacacugaucgggcuuugcuuuucccgguacucuggcugcuuaugcaguuuaaaagcucauaagcauuacgaguacugguagaggcagggucuaugacu   -3'   obs
    gacuuagucagacacugaucgggcuuugcuuuucccgguacucuggcugcuuaugcaguuuaaaagcucauaagcauuacgaguacugguagaggcagggucuaugacu         exp
    ......(((((...))))).((((((((((((.(((((((((((.((((((((.((((....)))))))))))))))).))))))))).))))))))))))......   reads   mm   sample
    ............cacugaucgggcCuugcuuuu............................................................................   1       1    seq
    ................................ucccgguacucuggcugcuu.........................................................   1       0    seq
    ................................ucccgguacucuggcugcuuau.......................................................   2       0    seq
    .................................cccgguacucuggcugcuu.........................................................   1       0    seq
    .................................cccgAuacucuggcugcuua........................................................   4       1    seq
    .................................cccgguacucuggcugcuua........................................................   3       0    seq
    .................................cccgCuacucuggcugcuuau.......................................................   1       1    seq
    .................................cccgguacucuggcugcuuau.......................................................   50      0    seq
    .................................cccgAuacucuggcugcuuau.......................................................   55      1    seq
    .................................cccgguacucuggcugcuuauU......................................................   3       1    seq
    .................................cccgguacucuggcugcuuauA......................................................   3       1    seq
    .................................cccgAuacucuggcugcuuaug......................................................   14      1    seq
    .................................cccgguacucuggcugcuuaug......................................................   84      0    seq
    .................................cccgguacucuggcugcuuaugA.....................................................   55      1    seq
    .................................cccgguacucuggcugcuuaugU.....................................................   4       1    seq
    .................................cccgguacucuggcugcuuaugc.....................................................   1       0    seq
    .................................cccgguacucuggcugcuuaugAa....................................................   2       1    seq
    ..................................ccgAuacucuggcugcuuau.......................................................   1       1    seq
    ....................................gAuacucuggcugcuuau.......................................................   1       1    seq
    .............................................................aaaagcucauaagcauuacgaguacugguag.................   1       0    seq
    ......................................................................uaagcauuacgaguacug.....................   2       0    seq
    ......................................................................uaagcauCacgaguacugg....................   2       1    seq
    ......................................................................uaagcauuacgaUuacugg....................   1       1    seq
    ......................................................................uaagcauuacgaguacugg....................   624     0    seq
    ......................................................................uaagcauuacgGguacugg....................   1       1    seq
    ......................................................................uaagcauuacAaguacugg....................   5       1    seq
    ......................................................................Aaagcauuacgaguacugg....................   1       1    seq
    ......................................................................uaagcauuaAgaguacugg....................   17      1    seq
    ......................................................................uaagcaAuacgaguacugg....................   1       1    seq
    ......................................................................uaagcauuacgaguacugU....................   1       1    seq
    ......................................................................uaagcauuacgaguacuAg....................   3       1    seq
    ......................................................................Caagcauuacgaguacugg....................   1       1    seq
    ......................................................................uaagcauuaAgaguacuggu...................   4       1    seq
```

```
                                Star                                Mature
gacuuagucagacacugaucgggcuuugcuuuucccgguacucuggcugcuuaugcaguuuaaaagcucauaagcauuacgaguacugguagaggcagggucuaugacu
......................................................................uaagcauuacgaguacuggu...................   57     0   seq
......................................................................uaagcauuacgaguacuggA...................   1      1   seq
......................................................................uaagcauuacgaguacuggG...................   2      1   seq
......................................................................uaagcauuaAgaguacuggua..................   63     1   seq
......................................................................uaagcauuacUaguacuggua..................   1      1   seq
......................................................................uaagcauuacAaguacuggua..................   3      1   seq
......................................................................uaagcauuacgaguacuggua..................   145    0   seq
......................................................................uaagcauuacgaCuacuggua..................   1      1   seq
......................................................................Gaagcauuacgaguacuggua..................   1      1   seq
......................................................................uaagcauuacgaguacuAgua..................   1      1   seq
......................................................................uaagcauuacgaguacugguG..................   1      1   seq
......................................................................uaagcauuGcgaguacuggua..................   1      1   seq
......................................................................uaaUcauuacgaguacugguag.................   1      1   seq
......................................................................uaagcauCacgaguacugguag.................   3      1   seq
......................................................................Aaagcauuacgaguacugguag.................   126    1   seq
......................................................................uCagcauuacgaguacugguag.................   1      1   seq
......................................................................Naagcauuacgaguacugguag.................   12     1   seq
......................................................................uaagUauuacgaguacugguag.................   2      1   seq
......................................................................uaagcauuacgGguacugguag.................   2      1   seq
......................................................................uaagcauuacgaguaAugguag.................   3      1   seq
......................................................................uaagcauuacgaguacugguaC.................   37     1   seq
......................................................................uaaCcauuacgaguacugguag.................   1      1   seq
......................................................................uaagcUuuacgaguacugguag.................   2      1   seq
......................................................................uaagcauuacgaUuacugguag.................   1      1   seq
......................................................................uaagcauuacgaguacugAuag.................   4      1   seq
......................................................................uaagcauuacgaguacugguUg.................   4      1   seq
......................................................................uaagcauuacgagAacugguag.................   11     1   seq
......................................................................uaGgcauuacgaguacugguag.................   1      1   seq
......................................................................uaagcauAacgaguacugguag.................   18     1   seq
......................................................................uaagcauuacgaguGcugguag.................   6      1   seq
......................................................................uaagcauuGcgaguacugguag.................   7      1   seq
......................................................................uaagcauGacgaguacugguag.................   1      1   seq
......................................................................uGagcauuacgaguacugguag.................   16     1   seq
......................................................................uaagcauuacgaguacugCuag.................   1      1   seq
......................................................................uaagcauuacgagCacugguag.................   4      1   seq
......................................................................uaagcauuacUaguacugguag.................   1      1   seq
......................................................................uaagcauuacgaguacugguag.................   30599  0   seq
......................................................................uaagcaGuacgaguacugguag.................   3      1   seq
......................................................................uaagcauuacgaguacuCguag.................   3      1   seq
......................................................................Gaagcauuacgaguacugguag.................   13     1   seq
......................................................................uaUgcauuacgaguacugguag.................   1      1   seq
......................................................................uaaAcauuacgaguacugguag.................   6      1   seq
......................................................................uaagcauuacgaguacuggCag.................   2      1   seq
......................................................................Caagcauuacgaguacugguag.................   6      1   seq
......................................................................uaagcauuacgaguacuggGag.................   7      1   seq
......................................................................uaagcauuacAaguacugguag.................   166    1   seq
......................................................................uaagcaAuacgaguacugguag.................   30     1   seq
......................................................................uaagcauuacgaguaUugguag.................   2      1   seq
......................................................................uaagcauuacgaguacugUuag.................   1      1   seq
......................................................................uaagcauuacgaguacugguGg.................   41     1   seq
......................................................................uaagcauuacgaguacCgguag.................   4      1   seq
......................................................................uaagcauuacgaguacAgguag.................   11     1   seq
......................................................................uaagcauuacgaguacuAguag.................   39     1   seq
......................................................................uaagcauuacgaAuacugguag.................   2      1   seq
......................................................................uNagcauuacgaguacugguag.................   4      1   seq
......................................................................uaagcauuacgaguacugguaU.................   3      1   seq
......................................................................uaagcauuacgaguUcugguag.................   3      1   seq
......................................................................uaagcauuaAgaguacugguag.................   3027   1   seq
......................................................................uaagcauuaUgaguacugguag.................   3      1   seq
......................................................................uaagcauuacgaguacuUguag.................   2      1   seq
......................................................................uaagcauuacCaguacugguag.................   1      1   seq
......................................................................uaagcauuacgaguacuggAag.................   7      1   seq
......................................................................uaagcauuaGgaguacugguag.................   4      1   seq
......................................................................uaagcauuacgaguaGugguag.................   5      1   seq
......................................................................uaagcauuacgagGacugguag.................   1      1   seq
......................................................................uaagGauuacgaguacugguag.................   3      1   seq
......................................................................uaagcauuacgaguacugguaA.................   42     1   seq
......................................................................uaagcGuuacgaguacugguag.................   6      1   seq
......................................................................uaagcauuacgUguacugguag.................   2      1   seq
......................................................................uaagGauuacgaguacugguaga................   1      1   seq
```

```
                                 Star                                Mature
gacuuagucagacacugaucgggcuuugcuuuucccgguacucuggcugcuuaugcaguuuaaaagcucauaagcauuacgaguacugguagaggcagggucuaugacu
......................................................................uaagcauuacAaguacugguaga................    9     1     seq
......................................................................uaagcaAuacgaguacugguaga................    2     1     seq
......................................................................uaagcauuacgaguacuAguaga................    1     1     seq
......................................................................uaagcauuacgaguGcugguaga................    1     1     seq
......................................................................Aaagcauuacgaguacugguaga................    17    1     seq
......................................................................uaagcauuacgaguaAugguaga................    1     1     seq
......................................................................uaagcauuaAgaguacugguaga................    1046  1     seq
......................................................................uaagcauuacgaguacugguaga................    3277  0     seq
......................................................................uaagcauuacgaguacuggAaga................    1     1     seq
......................................................................uaagcauuacgaguacugguGga................    1     1     seq
......................................................................uaagcauuacgaUuacugguaga................    1     1     seq
......................................................................uaagcaGuacgaguacugguaga................    1     1     seq
......................................................................uaagcauAacgaguacugguaga................    1     1     seq
......................................................................uaagcauuaGgaguacugguaga................    1     1     seq
......................................................................uaagcauuacgaguacugguaAa................    7     1     seq
......................................................................uaagcauuacgaguaUugguaga................    2     1     seq
......................................................................uaagcauuacgaguacAgguaga................    2     1     seq
......................................................................uaaAcauuacgaguacugguaga................    1     1     seq
......................................................................uaagcauuacgaguacugguagU................    128   1     seq
......................................................................uaagcauuacgaguacugguagC................    3     1     seq
......................................................................uaagcauuacgaguacugguagaC...............    4     1     seq
......................................................................uaagcauuacgaguacugguagag...............    2     0     seq
......................................................................uaagcauuaAgaguacugguagag...............    3     1     seq
......................................................................uaagcauuacgaguacugguagaA...............    717   1     seq
......................................................................uaagcauuacgaguacugguagaU...............    2     1     seq
......................................................................uaagcauuacgaguacugguagagA..............    8     1     seq
......................................................................uaagcauuacgaguacugguagaAg..............    17    1     seq
.......................................................................aagcauuacAaguacugguag.................    2     1     seq
.......................................................................aagcauuacgaguacugguag.................    35    0     seq
.......................................................................Uagcauuacgaguacugguag.................    2     1     seq
.......................................................................aagcauuacgaguacugguaga................    11    0     seq
.......................................................................aagcauuacgaguacugguagaU...............    1     1     seq
........................................................................agcauuacgaguacugguaga................    5     0     seq
..........................................................................cauuaAgaguacugguaga................    1     1     seq
```
