# Supplementary material for: The Small RNA Universe of Capitella teleta
Source: Front Mol Biosci. 2022 Feb 25;9:802814. doi: 10.3389/fmolb.2022.802814 (PMC8915122; doi:10.3389/fmolb.2022.802814)
Supplement: Supplementary file 1 [file DataSheet1.ZIP › Supplement/confident/CAPTEscaffold_298_16211.pdf]

Provisional ID	:	CAPTEscaffold_298_16211
Score total	:	188.6
Score for star read(s)	:	3.9
Score for read counts	:	181.6
Score for mfe	:	2.1
Score for randfold	:	1.6
Score for cons. seed	:	-0.6
Total read count	:	368
Mature read count	:	308
Loop read count	:	0
Star read count	:	60

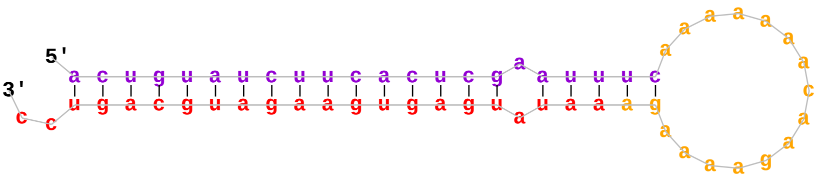

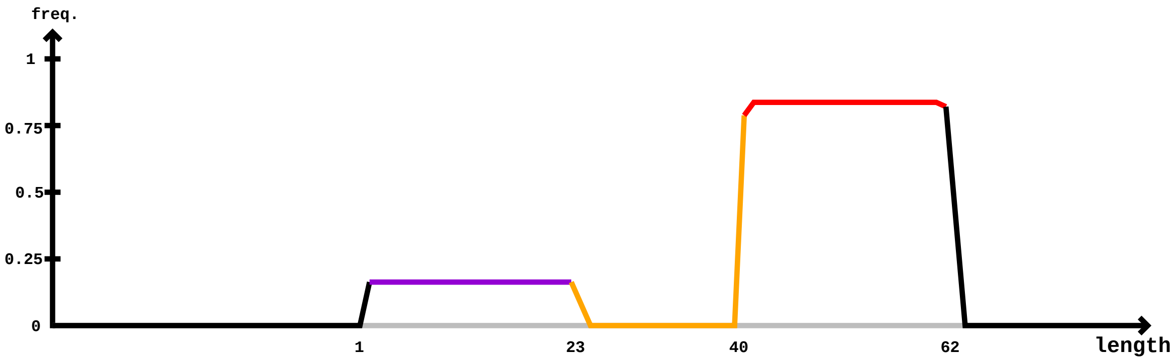


```
                                       Star                                   Mature
5'- cuccaccguuguuguucucgcguggaaauggacuguaucuucacucgaauuucaaaaaaacaagaaaagaaauaugagugaagaugcaguccauuuccauuguuuauacguc   -3'    obs
    cuccaccguuguuguucucgcguggaaauggacuguaucuucacucgaauuucaaaaaaacaagaaaagaaauaugagugaagaugcaguccauuuccauuguuuauacguc          exp
    .............((....((((((((((((((((((((((((((((.(((((...............))))).))))))))))))))))))))))))).)))....))...   reads      mm        sample
    ...............................acuguaucuucacCcgaauuuc...........................................................   1          1         seq
    ...............................acuguaucuucacucgaauuuc...........................................................   57         0         seq
    ...............................Gcuguaucuucacucgaauuuc...........................................................   1          1         seq
    ...............................acugAaucuucacucgaauuuc...........................................................   1          1         seq
    ......................................................................aauaugagugaagaugcaguc.....................   6          0         seq
    ......................................................................aauaugagugaagaugcagucc....................   275        0         seq
    ......................................................................aauaugagugaagaugcaAucc....................   1          1         seq
    ......................................................................aauGugagugaagaugcagucc....................   1          1         seq
    ......................................................................aauaugagugaagaugcagCcc....................   1          1         seq
    ......................................................................aauauAagugaagaugcagucc....................   1          1         seq
    ......................................................................aauaugagugaagaCgcagucc....................   1          1         seq
    ......................................................................aauaugagGgaagaugcagucc....................   1          1         seq
    ......................................................................aauaugagugaagaugcaguccU...................   3          1         seq
    .......................................................................auauAagugaagaugcagucc....................   1          1         seq
    .......................................................................auaugagugaagaugcagucc....................   17         0         seq
```
