# Supplementary material for: The Small RNA Universe of Capitella teleta
Source: Front Mol Biosci. 2022 Feb 25;9:802814. doi: 10.3389/fmolb.2022.802814 (PMC8915122; doi:10.3389/fmolb.2022.802814)
Supplement: Supplementary file 1 [file DataSheet1.ZIP › Supplement/confident/CAPTEscaffold_636_25192.pdf]

Provisional ID	:	CAPTEscaffold_636_25192
Score total	:	459.8
Score for star read(s)	:	3.9
Score for read counts	:	463
Score for mfe	:	-4.3
Score for randfold	:	-2.2
Score for cons. seed	:	-0.6
Total read count	:	920
Mature read count	:	912
Loop read count	:	0
Star read count	:	8

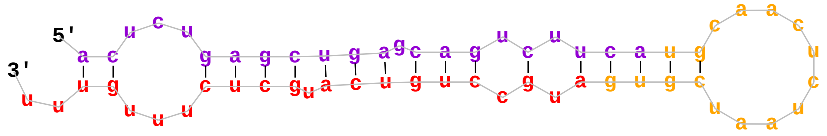

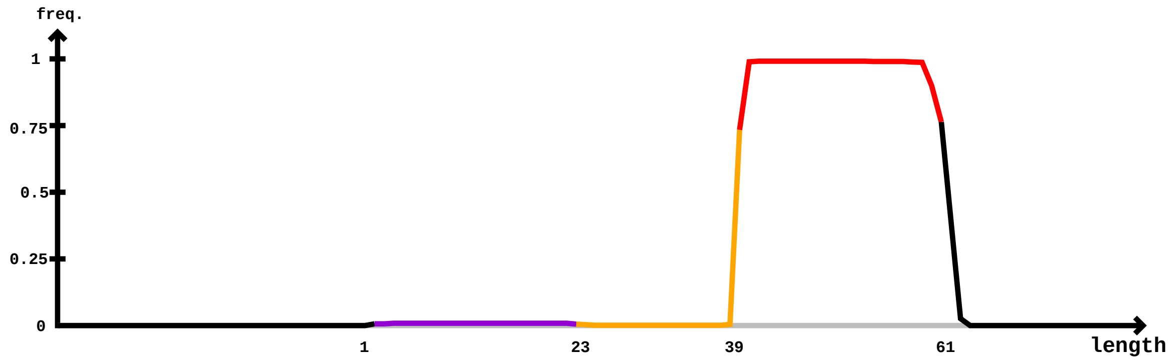


```
                                       Star                                             Mature
5'- ccuucauugcuuguucauuuuguugagccauacucugagcugagcagucuucaugcaacucuaaucgugaugccugucaugcucuuuguuugauucacuuuucucacaucu  -3'   obs
    ccuucauugcuuguucauuuuguugagccauacucugagcugagcagucuucaugcaacucuaaucgugaugccugucaugcucuuuguuugauucacuuuucucacaucu         exp
    ...........(((((......)))))((.((...(((((((.(((.(.(((((..........))))).).)))))).))))...)).))....................   reads   mm   sample
    ...............................acucugagcugagcagucuuc...........................................................   3       0    seq
    ...............................acucugagcugagcagucuuca..........................................................   3       0    seq
    .................................ucugagcugagcagucuucau.........................................................   2       0    seq
    .....................................................ugcaacucuaaucgugaugccugucaugcu............................   1       0    seq
    ....................................................................gaugccugucaugcucuuuguu.....................   2       0    seq
    ....................................................................Caugccugucaugcucuuuguuu....................   1       1    seq
    .....................................................................augccugucaugcucuuu........................   2       0    seq
    .....................................................................augccugucaugcucuuug.......................   1       0    seq
    .....................................................................augccugucaugcucuuugu......................   73      0    seq
    .....................................................................augccugucaugcucuuAgu......................   1       1    seq
    .....................................................................Gugccugucaugcucuuugu......................   1       1    seq
    .....................................................................augccugucaugcucuAugu......................   1       1    seq
    .....................................................................augccugucaugcucuuuguu.....................   104     0    seq
    .....................................................................augccugucaugcucuuuUuu.....................   1       1    seq
    .....................................................................augccugucaugcuUuuuguu.....................   1       1    seq
    .....................................................................auAccugucaugcucuuuguuu....................   1       1    seq
    .....................................................................Gugccugucaugcucuuuguuu....................   1       1    seq
    .....................................................................augccugucauUcucuuuguuu....................   1       1    seq
    .....................................................................augccugucaugcucuuuguuu....................   443     0    seq
    .....................................................................augccuguUaugcucuuuguuu....................   1       1    seq
    .....................................................................augccugucaugcCcuuuguuu....................   1       1    seq
    .....................................................................augccAgucaugcucuuuguuu....................   1       1    seq
    .....................................................................augccugucaugcucuuuguuC....................   1       1    seq
    .....................................................................augccuguGaugcucuuuguuu....................   1       1    seq
    .....................................................................augccugucaugcAcuuuguuu....................   1       1    seq
    .....................................................................augccugucaugcucuuuguuuA...................   10      1    seq
    .....................................................................augccugucaugcucuuuguuuU...................   5       1    seq
    .....................................................................augccugucaugcucuuuguuuga..................   1       0    seq
    .....................................................................augccugucaugcucuuuguuuAa..................   19      1    seq
    ......................................................................ugccugucaugcucuuugu......................   5       0    seq
    ......................................................................ugccugucaugcucuuuguu.....................   17      0    seq
    ......................................................................ugccugucaugcuUuuuguuu....................   1       1    seq
    ......................................................................ugccugucaugcucuAuguuu....................   1       1    seq
```

Star **Mature**

```
ccuucauugcuuguucauuuuguugagccauacucugagcugagcagucuucaugcaacucuaaucgugaugccugucaugcucuuuguuugauucacuuuucucacaucu
......................................................................ugcUugucaugcucuuuguuu...................     1     1     seq
......................................................................ugccugucaugcucuuuguuu...................     194   0     seq
......................................................................ugccugAcaugcucuuuguuu...................     1     1     seq
......................................................................ugccugucaugcucuuuguuuU..................     1     1     seq
......................................................................ugccugucaugcucuuuguuuA..................     10    1     seq
......................................................................ugccugucaugcucuuuguuuAa.................     3     1     seq
......................................................................ugccugucaugcucuuuguuuCa.................     1     1     seq
.......................................................................gccugucaugcucuuuguuu...................     2     0     seq
```
