# Supplementary material for: The Small RNA Universe of Capitella teleta
Source: Front Mol Biosci. 2022 Feb 25;9:802814. doi: 10.3389/fmolb.2022.802814 (PMC8915122; doi:10.3389/fmolb.2022.802814)
Supplement: Supplementary file 1 [file DataSheet1.ZIP › Supplement/confident/CAPTEscaffold_1278_33446.pdf]

Provisional ID	:	CAPTEscaffold_1278_33446
Score total	:	108069.2
Score for star read(s)	:	3.9
Score for read counts	:	108062.7
Score for mfe	:	1.6
Score for randfold	:	1.6
Score for cons. seed	:	-0.6
Total read count	:	211972
Mature read count	:	211900
Loop read count	:	0
Star read count	:	72

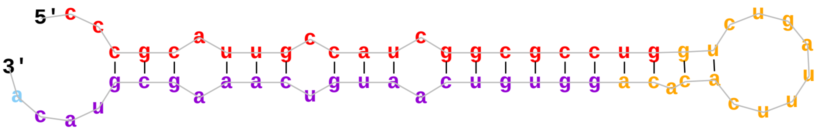

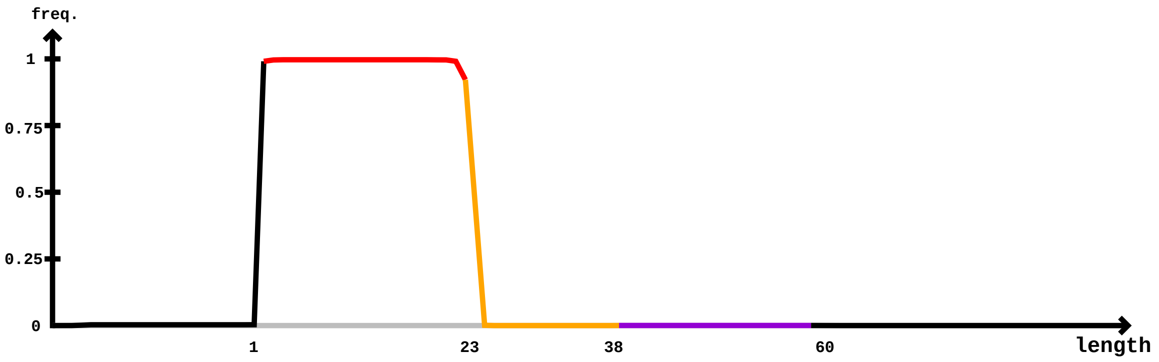

Mature   Star

```
5'- cucugaaugaccuugcauugcccgcauugccaucggcgccuggucugauuucacacaggugucaaugucaaagcguacaaugugacgucaauucgcuucauuuauaugau  -3'  obs
    cucugaaugaccuugcauugcccgcauugccaucggcgccuggucugauuucacacaggugucaaugucaaagcguacaaugugacgucaauucgcuucauuuauaugau        exp
    ...((((((((.((((((((..(((.(((.(((.((((((((((........)).)))))))).))).))).)))..)))))))).))).)))))..((((....)))).   reads  mm  sample
    cucuAaaugaccuugcauug..........................................................................................   2      1   seq
    cucugaaugaccuugcauug..........................................................................................   29     0   seq
    .ucugaaugaccuugcauu...........................................................................................   2      0   seq
    .ucugaaugaccuugcauuA..........................................................................................   1      1   seq
    .ucugaaugacAuugcauug..........................................................................................   1      1   seq
    .ucugaaugaccuugcauug..........................................................................................   255    0   seq
    .ucugaGugaccuugcauug..........................................................................................   1      1   seq
    .ucugaaAgaccuugcauug..........................................................................................   1      1   seq
    .Acugaaugaccuugcauug..........................................................................................   1      1   seq
    ..cugaaugaccuGgcauug..........................................................................................   1      1   seq
    ..cugaaugaccuugcaAug..........................................................................................   1      1   seq
    ..cugaaugaccuugcauCg..........................................................................................   1      1   seq
    ..cugaaugacAuugcauug..........................................................................................   1      1   seq
    ..cugaaugaccAugcauug..........................................................................................   1      1   seq
    ..cugaaugaccuugcauug..........................................................................................   336    0   seq
    ..cugaaugaccuugAauug..........................................................................................   1      1   seq
    ...................Ccccgcauugccaucggcgccug....................................................................   46     1   seq
    ...................gcccgcauugccaucggcgccug....................................................................   1      0   seq
    ....................cccgcauugccaucggcg........................................................................   87     0   seq
    ....................ccAgcauugccaucggcg........................................................................   1      1   seq
    ....................cccgcauugccaucggcgc.......................................................................   34     0   seq
    ....................Nccgcauugccaucggcgcc......................................................................   1      1   seq
    ....................cccgcauugcUaucggcgcc......................................................................   2      1   seq
    ....................cccgcauugccaucggcgcc......................................................................   1060   0   seq
    ....................Accgcauugccaucggcgcc......................................................................   1      1   seq
    ....................cccgcauugcAaucggcgcc......................................................................   1      1   seq
    ....................cccgcauugccaGcggcgcc......................................................................   1      1   seq
    ....................cccgcauugccauUggcgcc......................................................................   2      1   seq
    ....................cccgcaCugccaucggcgcc......................................................................   1      1   seq
    ....................cccgcauugccaucAgcgcc......................................................................   1      1   seq
    ....................cccgcauugccaucUgcgcc......................................................................   1      1   seq
    ....................cccAcauugccaucggcgcc......................................................................   1      1   seq
    ....................cUcgcauugccaucggcgcc......................................................................   1      1   seq
```

Mature Star

```
cucugaaugaccuugcauugcccgcauugccaucggcgccuggucugauuucacacaggugucaaugucaaagcguacaaugugacgucaauucgcuucauuuauaugau
....................cccgcaAugccaucggcgcc......................................................................	1	1	seq
....................Uccgcauugccaucggcgccu.....................................................................	13	1	seq
....................cccgcauugccaucUgcgccu.....................................................................	1	1	seq
....................cccgcauugccaucgCcgccu.....................................................................	1	1	seq
....................cccgcUuugccaucggcgccu.....................................................................	2	1	seq
....................cccgcauugccaucggcUccu.....................................................................	1	1	seq
....................cccgcauugccCucggcgccu.....................................................................	1	1	seq
....................cccgcauugccaucggcgcAu.....................................................................	7	1	seq
....................cccgcauugccaucggUgccu.....................................................................	1	1	seq
....................cccgcauCgccaucggcgccu.....................................................................	4	1	seq
....................cccgcauugccaucggcgccC.....................................................................	8	1	seq
....................cccgcGuugccaucggcgccu.....................................................................	1	1	seq
....................cccgcauugcUaucggcgccu.....................................................................	5	1	seq
....................Gccgcauugccaucggcgccu.....................................................................	1	1	seq
....................cccgcauugccaucggcgGcu.....................................................................	1	1	seq
....................cccgcauugccGucggcgccu.....................................................................	2	1	seq
....................cccgcauugccaucgAcgccu.....................................................................	3	1	seq
....................cccgcauugccaucggcgccA.....................................................................	117	1	seq
....................cccgcauugccaucAgcgccu.....................................................................	11	1	seq
....................ccAgcauugccaucggcgccu.....................................................................	7	1	seq
....................cccgcauugcAaucggcgccu.....................................................................	2	1	seq
....................Nccgcauugccaucggcgccu.....................................................................	8	1	seq
....................cccgcaCugccaucggcgccu.....................................................................	1	1	seq
....................cUcgcauugccaucggcgccu.....................................................................	8	1	seq
....................cccgcauugccaucggcgccG.....................................................................	183	1	seq
....................Accgcauugccaucggcgccu.....................................................................	12	1	seq
....................cccgcauugccaucggcgUcu.....................................................................	2	1	seq
....................cccgcauugcGaucggcgccu.....................................................................	2	1	seq
....................cccgcauugUcaucggcgccu.....................................................................	2	1	seq
....................cccgcauugccaCcggcgccu.....................................................................	1	1	seq
....................cccgcauugccaGcggcgccu.....................................................................	1	1	seq
....................cccgcauugNcaucggcgccu.....................................................................	1	1	seq
....................cccgcauugccauAggcgccu.....................................................................	2	1	seq
....................cccgcauugccauUggcgccu.....................................................................	8	1	seq
....................cccgUauugccaucggcgccu.....................................................................	9	1	seq
....................ccUgcauugccaucggcgccu.....................................................................	16	1	seq
....................ccGgcauugccaucggcgccu.....................................................................	1	1	seq
....................cccgcauugccaucggcgcUu.....................................................................	1	1	seq
....................cccgcauugccaucggcgccu.....................................................................	14212	0	seq
....................cccgcauAgccaucggcgccu.....................................................................	6	1	seq
....................cccgcauugccaucggAgccu.....................................................................	2	1	seq
....................cccgcauuCccaucggcgccu.....................................................................	4	1	seq
....................cAcgcauugccaucggcgccu.....................................................................	1	1	seq
....................cccgcauugAcaucggcgccu.....................................................................	3	1	seq
....................cccgcauuAccaucggcgccu.....................................................................	12	1	seq
....................cccgcauugccaucggGgccu.....................................................................	2	1	seq
....................cccgcaAugccaucggcgccu.....................................................................	13	1	seq
....................cccgcauugccaucggcgAcu.....................................................................	2	1	seq
....................cccgcauugccaAcggcgccu.....................................................................	6	1	seq
....................cccgAauugccaucggcgccu.....................................................................	3	1	seq
....................cccgGauugccaucggcgccug....................................................................	8	1	seq
....................ccAgcauugccaucggcgccug....................................................................	80	1	seq
....................cAcgcauugccaucggcgccug....................................................................	50	1	seq
....................cccgcGuugccaucggcgccug....................................................................	26	1	seq
....................cccCcauugccaucggcgccug....................................................................	8	1	seq
....................cccgcauugccauUggcgccug....................................................................	202	1	seq
....................Gccgcauugccaucggcgccug....................................................................	14	1	seq
....................cccgcauugccaucggcUccug....................................................................	9	1	seq
....................cccgcauugccaCcggcgccug....................................................................	32	1	seq
....................cccgcauugccaucggcgccuA....................................................................	60	1	seq
....................cccgcauuAccaucggcgccug....................................................................	30	1	seq
....................cccgAauugccaucggcgccug....................................................................	34	1	seq
....................cccgcauugccaucggcAccug....................................................................	13	1	seq
....................cccgcauugccaucggGgccug....................................................................	19	1	seq
....................cccgcauugccaucggcgccCg....................................................................	40	1	seq
....................cGcgcauugccaucggcgccug....................................................................	7	1	seq
....................cccgcauGgccaucggcgccug....................................................................	1	1	seq
....................cccgcauugccCucggcgccug....................................................................	2	1	seq
....................cccgcauugccaucggNgccug....................................................................	2	1	seq
....................cccgcauCgccaucggcgccug....................................................................	28	1	seq
```

Mature Star

```
cucugaaugaccuugcauugcccgcauugccaucggcgccuggucugauuucacacaggugucaaugucaaagcguacaaugugacgucaauucgcuucauuuauaugau
....................cccgcauugccaucgUcgccug....................................................................    11      1    seq
....................cccUcauugccaucggcgccug....................................................................    7       1    seq
....................Uccgcauugccaucggcgccug....................................................................    153     1    seq
....................ccUgcauugccaucggcgccug....................................................................    163     1    seq
....................cccgcauugccaucCgcgccug....................................................................    4       1    seq
....................cNcgcauugccaucggcgccug....................................................................    4       1    seq
....................cccgcauugccaGcggcgccug....................................................................    9       1    seq
....................cccgcauugccaucggcgccuU....................................................................    89      1    seq
....................cccgcauugccaucggcgGcug....................................................................    7       1    seq
....................cccgcaAugccaucggcgccug....................................................................    117     1    seq
....................cccgcaGugccaucggcgccug....................................................................    9       1    seq
....................cccgcauugccaucggcgccAg....................................................................    51      1    seq
....................cccgcauugccUucggcgccug....................................................................    17      1    seq
....................cccgcauugccaucggcgccug....................................................................    181549  0    seq
....................cccgcauugccauGggcgccug....................................................................    3       1    seq
....................cccgcauugGcaucggcgccug....................................................................    15      1    seq
....................ccNgcauugccaucggcgccug....................................................................    1       1    seq
....................cccgcauugccaucggcCccug....................................................................    5       1    seq
....................cccgcauuCccaucggcgccug....................................................................    27      1    seq
....................cccgcauugccaucggcgcUug....................................................................    40      1    seq
....................cccgcauugccaucggcgAcug....................................................................    41      1    seq
....................cccgcauugccaAcggcgccug....................................................................    40      1    seq
....................cccgcauugAcaucggcgccug....................................................................    60      1    seq
....................Nccgcauugccaucggcgccug....................................................................    58      1    seq
....................cccgcauugccaucggcgUcug....................................................................    14      1    seq
....................cccgcauugccaucggcgccGg....................................................................    2629    1    seq
....................cccgcUuugccaucggcgccug....................................................................    15      1    seq
....................cccgcauugccaucggcgccNg....................................................................    2       1    seq
....................cccgcauugcGaucggcgccug....................................................................    20      1    seq
....................cccgcCuugccaucggcgccug....................................................................    12      1    seq
....................cccgcauugccaucggcgccuC....................................................................    15      1    seq
....................ccGgcauugccaucggcgccug....................................................................    8       1    seq
....................cccgcaCugccaucggcgccug....................................................................    12      1    seq
....................cccgcauugccaucggcgcGug....................................................................    6       1    seq
....................cccgcauAgccaucggcgccug....................................................................    133     1    seq
....................cccgcauugcAaucggcgccug....................................................................    55      1    seq
....................cUcgcauugccaucggcgccug....................................................................    87      1    seq
....................cccAcauugccaucggcgccug....................................................................    38      1    seq
....................cccgcauuUccaucggcgccug....................................................................    7       1    seq
....................cccgcauugccauNggcgccug....................................................................    1       1    seq
....................cccgcauugccaucggUgccug....................................................................    31      1    seq
....................cccgcauugUcaucggcgccug....................................................................    29      1    seq
....................cccgcauugccaucggcgcAug....................................................................    103     1    seq
....................cccgcaNugccaucggcgccug....................................................................    1       1    seq
....................cccgcauugcUaucggcgccug....................................................................    79      1    seq
....................cccgcauugccaucgCcgccug....................................................................    11      1    seq
....................cccgcNuugccaucggcgccug....................................................................    5       1    seq
....................cccgcauugccaucggcgccuN....................................................................    1       1    seq
....................cccgcauugccaucgAcgccug....................................................................    13      1    seq
....................cccgcauugccauAggcgccug....................................................................    37      1    seq
....................Accgcauugccaucggcgccug....................................................................    51      1    seq
....................cccgcauugccaucggAgccug....................................................................    41      1    seq
....................cccgUauugccaucggcgccug....................................................................    23      1    seq
....................cccgcauugccaucAgcgccug....................................................................    115     1    seq
....................cccgcauugccGucggcgccug....................................................................    27      1    seq
....................cccgcauugccaucUgcgccug....................................................................    13      1    seq
....................cccgcauugccaucggcgccugA...................................................................    1043    1    seq
....................cccgcauugccaucggcgccugN...................................................................    1       1    seq
....................cccgcauugccaucggcgccugU...................................................................    6611    1    seq
....................cccgcauugccaucggcgccugC...................................................................    8       1    seq
....................cccgcauugccaucggcgccugg...................................................................    67      0    seq
....................cccgcauugccaucggcgccugAu..................................................................    5       1    seq
....................cccgcauugccaucggcgccugUu..................................................................    196     1    seq
....................cccgcauugccaucggcgccuggA..................................................................    2       1    seq
.....................ccgcauugccaucggcgcc......................................................................    1       0    seq
.....................ccgcauugccaucggcgccu.....................................................................    21      0    seq
.....................ccgcauugccaucggcgccG.....................................................................    2       1    seq
.....................cUgcauugccaucggcgccug....................................................................    5       1    seq
.....................ccgcauugcUaucggcgccug....................................................................    1       1    seq
.....................ccgcauugccaucAgcgccug....................................................................    1       1    seq
```

```
                    Mature                               Star
cucugaaugaccuugcauugcccgcauugccaucggcgccuggucugauuucacacaggugucaaugucaaagcguacaaugugacgucaauucgcuucauuuauaugau
.....................ccAcauugccaucggcgccug....................................................................  1    1  seq
.....................Ncgcauugccaucggcgccug....................................................................  1    1  seq
.....................ccgcauuAccaucggcgccug....................................................................  1    1  seq
.....................ccgcauAgccaucggcgccug....................................................................  1    1  seq
.....................ccgcauugccaucggcgccug....................................................................  859  0  seq
.....................ccCcauugccaucggcgccug....................................................................  3    1  seq
.....................ccgcauugccaAcggcgccug....................................................................  1    1  seq
.....................ccgcauugccaucggcgcGug....................................................................  1    1  seq
.....................ccgcauugcAaucggcgccug....................................................................  2    1  seq
.....................ccgcauugccaucggcgccGg....................................................................  8    1  seq
.....................ccgcauugccaucggcgcAug....................................................................  2    1  seq
.....................ccgcauugccaucggcgccugg...................................................................  4    0  seq
.....................ccgcauugccaucggcgccugU...................................................................  144  1  seq
......................cgcauugccaucggcgccu.....................................................................  3    0  seq
......................cgcauugccaucggAgccug....................................................................  1    1  seq
......................cgUauugccaucggcgccug....................................................................  1    1  seq
......................cgcauugccaucggcgccGg....................................................................  5    1  seq
......................cCcauugccaucggcgccug....................................................................  4    1  seq
......................cgcauugccaucggcgUcug....................................................................  1    1  seq
......................cgcauugccaucggcgccug....................................................................  155  0  seq
.......................gcauugccaucggcgccug....................................................................  2    0  seq
..........................uugccaucggcgccugguc.................................................................  1    0  seq
.........................................................ggugucaaugucaaagcguac................................  57   0  seq
.........................................................ggugucaaugucaaagcguaca...............................  15   0  seq
...............................................................................augugacgucaauucgcuu............  1    0  seq
```
