# Supplementary material for: The Small RNA Universe of Capitella teleta
Source: Front Mol Biosci. 2022 Feb 25;9:802814. doi: 10.3389/fmolb.2022.802814 (PMC8915122; doi:10.3389/fmolb.2022.802814)
Supplement: Supplementary file 1 [file DataSheet1.ZIP › Supplement/confident/CAPTEscaffold_488_22755.pdf]

Provisional ID	:	CAPTEscaffold_488_22755
Score total	:	1680.5
Score for star read(s)	:	3.9
Score for read counts	:	1674.4
Score for mfe	:	1.2
Score for randfold	:	1.6
Score for cons. seed	:	-0.6
Total read count	:	3296
Mature read count	:	2311
Loop read count	:	0
Star read count	:	985

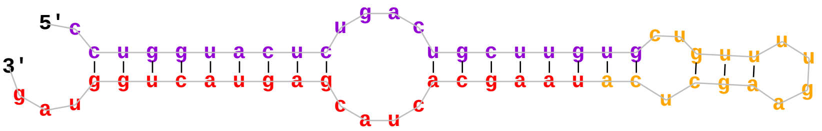

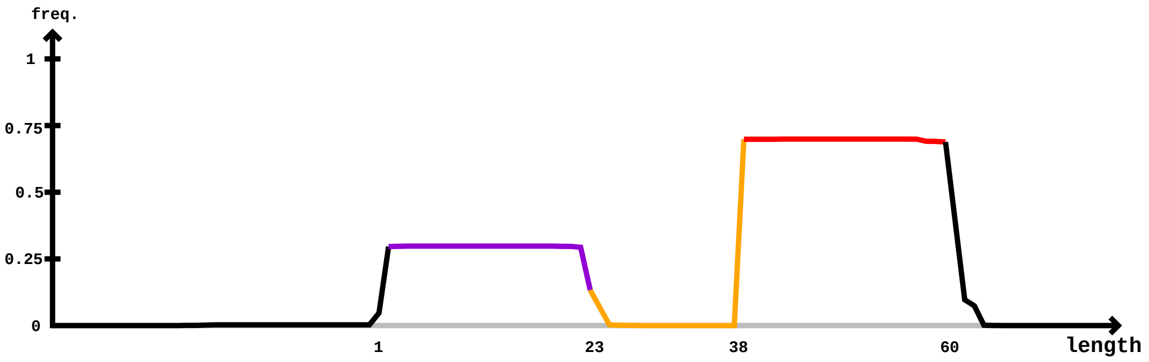

Star Mature

```
5'- uggugguauuugacgcuguucggucuugccuuuccugguacucugacugcuugugcuguuuugaagcucauaagcacuacgaguacugguagagacagggucuaaaacu  -3'   obs
    uggugguauuugacgcuguucggucuugccuuuccugguacucugacugcuugugcuguuuugaagcucauaagcacuacgaguacugguagagacagggucuaaaacu         exp
    .((((........))))....(((((((.((((.(((((((((....((((((((..(((....))).))))))))....))))))))).)))).)))))))....... reads  mm  sample
    ............acgcuguucggucuugccuuu............................................................................ 2      0   seq
    ..............gcuguucggucuugccuuu............................................................................ 3      0   seq
    ..............gcuguuGggucuugccuuu............................................................................ 1      1   seq
    ...............cuguucggucuugccuuu............................................................................ 3      0   seq
    ................................uccugguacucugacugcu.......................................................... 1      0   seq
    ................................uccugguacucugacugcuug........................................................ 5      0   seq
    ................................uccugguacucugacugcuugA....................................................... 1      1   seq
    ................................uccugguacucugacugcuugu....................................................... 140    0   seq
    .................................ccugguacucugacugcu.......................................................... 2      0   seq
    .................................ccugguacucugacugcuu......................................................... 1      0   seq
    .................................ccugguacucugacugcuug........................................................ 6      0   seq
    .................................ccugguacucCgacugcuugu....................................................... 1      1   seq
    .................................ccugguacucugacugcuugu....................................................... 389    0   seq
    .................................ccugguacucugacugcuCgug...................................................... 1      1   seq
    .................................ccugguacucCgacugcuugug...................................................... 5      1   seq
    .................................ccugguacucugacugcuGgug...................................................... 1      1   seq
    .................................ccuggAacucugacugcuugug...................................................... 1      1   seq
    .................................ccuAguacucugacugcuugug...................................................... 2      1   seq
    .................................ccugguacucugacugcuugug...................................................... 383    0   seq
    .................................ccugguacucugacugcuugugc..................................................... 4      0   seq
    .................................ccugguacucugacugcuugugA..................................................... 26     1   seq
    .................................ccugguacucugacugcuugugG..................................................... 3      1   seq
    .................................ccugguacucugacugcuugugcu.................................................... 3      0   seq
    .................................ccugguacucugacugcuugugcug................................................... 1      0   seq
    .................................ccugguacucugacugcuugugcugu.................................................. 2      0   seq
    .................................ccugguacucugacugcuugugcuguu................................................. 1      0   seq
    ..................................cugguacucugacugcuugu....................................................... 1      0   seq
    ..................................cugguacucugacugcuugug...................................................... 1      0   seq
    ..................................cugguacucugacugcuugugA..................................................... 1      1   seq
    ...................................ugguacucugacugcuugug...................................................... 1      0   seq
    ...................................ugguacucugacugcuugugcu.................................................... 2      0   seq
    ......................................................................uaagcacuacgaguacug..................... 1      0   seq
    ......................................................................uaagcacuacgaguacugg.................... 25     0   seq
```

```
                                Star                                 Mature
uggugguauuugacgcuguucggucuugccuuuccugguacucugacugcuugugcuguuuugaagcucauaagcacuacgaguacugguagagacagggucuaaaacu
......................................................................uaagcaAuacgaguacugg....................    1     1    seq
......................................................................uaagcacuacgaguacuggu...................    2     0    seq
......................................................................uaagcacuacgaguacuAgua..................    2     1    seq
......................................................................uaagcacuacgaguacuggua..................    5     0    seq
......................................................................uaagcacuacgaguacuAguag.................    2     1    seq
......................................................................uaagcacuacgaguacugguag.................    1735  0    seq
......................................................................uaagcaGuacgaguacugguag.................    3     1    seq
......................................................................Aaagcacuacgaguacugguag.................    5     1    seq
......................................................................uaagcacuacgaguacugguaA.................    3     1    seq
......................................................................uaagcacuacgaguaAugguag.................    1     1    seq
......................................................................uaagcacuacgaguacugguaC.................    1     1    seq
......................................................................uaagcacuacgaguacugguGg.................    8     1    seq
......................................................................uaagcacuacAaguacugguag.................    5     1    seq
......................................................................uaagcacuaGgaguacugguag.................    2     1    seq
......................................................................uaagcaAuacgaguacugguag.................    30    1    seq
......................................................................uaagAacuacgaguacugguag.................    1     1    seq
......................................................................uaagcacCacgaguacugguag.................    1     1    seq
......................................................................uaagcacuacgaguacugguagU................    5     1    seq
......................................................................uaagcacuacAaguacugguaga................    1     1    seq
......................................................................uaagcacuacgaguacugguaga................    145   0    seq
......................................................................uaagcaAuacgaguacugguaga................    2     1    seq
......................................................................Aaagcacuacgaguacugguaga................    1     1    seq
......................................................................uaagcaGuacgaguacugguaga................    1     1    seq
......................................................................uaagcacuacgaguacugAuaga................    1     1    seq
......................................................................uaagcacuacgaguacugguagaA...............    72    1    seq
......................................................................uaagcacuacgaguacugguagaC...............    3     1    seq
......................................................................uaagcacuacgaguacugguagaCa..............    2     1    seq
......................................................................uaagcaUuacgaguacugguagaga..............    8     1    seq
......................................................................uaagcacuacgaguacugguagaAa..............    231   1    seq
......................................................................uaagcacuacgaguacugguagaAac.............    3     1    seq
......................................................................uaagcacuacgaguacugguagaAaca............    1     1    seq
..........................................................................cacuacgaguacugguag.................    2     0    seq
```
