# Supplementary material for: The Small RNA Universe of Capitella teleta
Source: Front Mol Biosci. 2022 Feb 25;9:802814. doi: 10.3389/fmolb.2022.802814 (PMC8915122; doi:10.3389/fmolb.2022.802814)
Supplement: Supplementary file 1 [file DataSheet1.ZIP › Supplement/confident/CAPTEscaffold_191_13165.pdf]

Provisional ID	:	CAPTEscaffold_191_13165
Score total	:	115.8
Score for star read(s)	:	3.9
Score for read counts	:	109.7
Score for mfe	:	1.2
Score for randfold	:	1.6
Score for cons. seed	:	-0.6
Total read count	:	227
Mature read count	:	178
Loop read count	:	0
Star read count	:	49

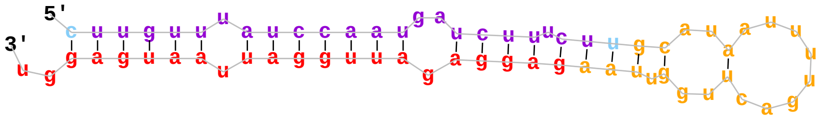

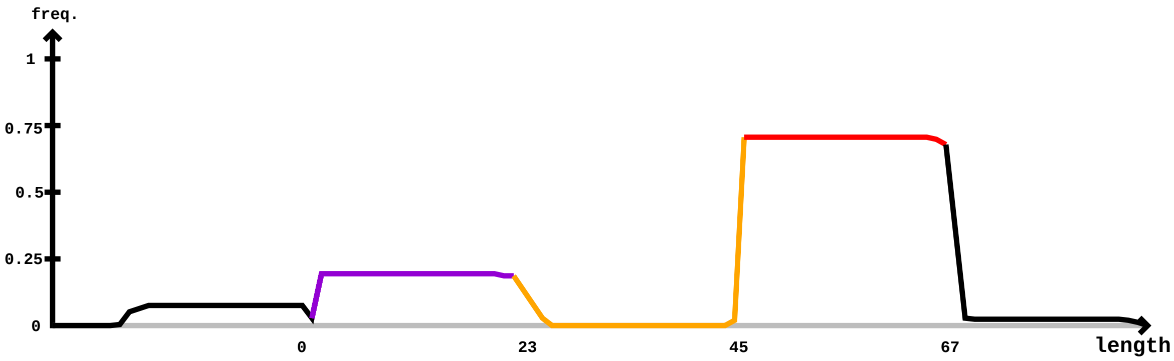

Star Mature

```
5'- aucaaagugacugcugcacucugaccuuguuuauccaaugaucuuucuugcauaauuuugacuugguuaagaggagauuggauuaaugaggugagacugcagacaugaaaac  -3'   obs
    aucaaagugacugcugcacucugaccuuguuuauccaaugaucuuucuugcauaauuuugacuugguuaagaggagauuggauuaaugaggugagacugcagacaugaaaac         exp
    ...........(((((((.(((.((((((((.(((((((..((((.(((((..(........)..).)))))))).)))))))).)))))))).))).))))).)).......   reads   mm   sample
    .....agugacugcugcacucugacc......................................................................................   1       0    seq
    ......gugacugcugcacucugac.......................................................................................   12      0    seq
    .......ugacugcugcacucugacc......................................................................................   3       0    seq
    ........gacugcugcacucugacc......................................................................................   3       0    seq
    ..........................uuguuuauccaaugaucuu...................................................................   2       0    seq
    ..........................uuguuuauccaaugaucuuucu................................................................   21      0    seq
    ..........................uuguuuauccaaugaucuuucuu...............................................................   19      0    seq
    ..........................uuguuuauccaaugaucuuucuug..............................................................   7       0    seq
    .....................................................................agaggagauuggauuaaugag......................   1       0    seq
    .....................................................................agaggagauuggauuaaugagU.....................   2       1    seq
    .....................................................................agaggagauuggauuaaugagg.....................   2       0    seq
    ......................................................................gaggagauuggauuaaugag......................   1       0    seq
    ......................................................................gaggagauuggauuaaugagg.....................   1       0    seq
    ......................................................................gaggagauuggauuaaugaggu....................   169     0    seq
    ......................................................................gaggagauuggauuaaugagguA...................   1       1    seq
    ......................................................................gaggagauuggauuaaugagguAa..................   1       1    seq
    ............................................................................................gagacugcagacaugaaa..   1       0    seq
    ............................................................................................gagacugcagacaugaaaa.   2       0    seq
    ............................................................................................gagacugcagacaugaaaaA   1       1    seq
    ............................................................................................gagacugcagacaugaaaac   2       0    seq
```
