# Supplementary material for: The Small RNA Universe of Capitella teleta
Source: Front Mol Biosci. 2022 Feb 25;9:802814. doi: 10.3389/fmolb.2022.802814 (PMC8915122; doi:10.3389/fmolb.2022.802814)
Supplement: Supplementary file 1 [file DataSheet1.ZIP › Supplement/confident/CAPTEscaffold_183_11947.pdf]

Provisional ID	:	CAPTEscaffold_183_11947
Score total	:	1080
Score for star read(s)	:	3.9
Score for read counts	:	1074.3
Score for mfe	:	0.8
Score for randfold	:	1.6
Score for cons. seed	:	-0.6
Total read count	:	2119
Mature read count	:	2106
Loop read count	:	0
Star read count	:	13

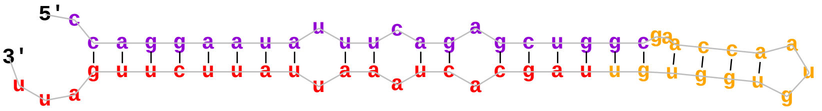

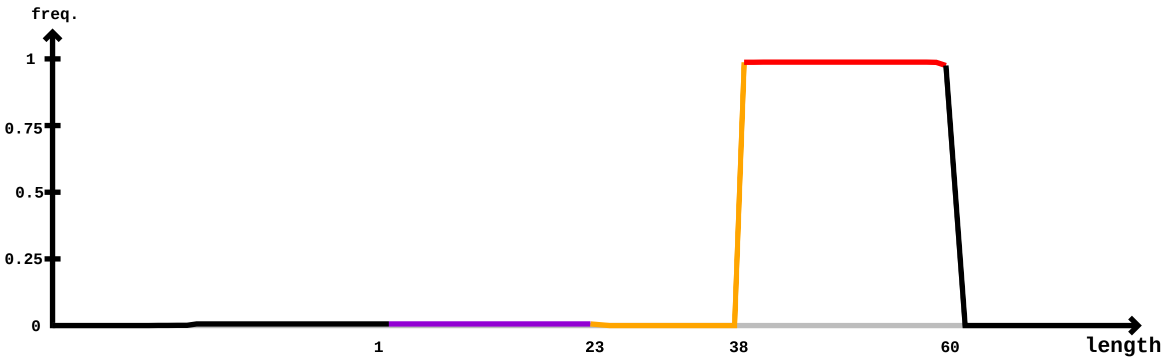

Star Mature

```
5'- caauucaaaaagagagcaugcuagaugaccacaccaggaauauuucagagcuggcgaaccaauguggugguuagcacuaaaauuauucuugauuugguuucuaagcaguacau  -3'   obs
    caauucaaaaagagagcaugcuagaugaccacaccaggaauauuucagagcuggcgaaccaauguggugguuagcacuaaaauuauucuugauuugguuucuaagcaguacau        exp
    ................((.(((((((.(((((...((((((((.((.((.((((((..((((...)))))))))).)).)).))))))))...)))))))).))))))....   reads   mm   sample
    .........aagagagcaugcuagaugaccaca...............................................................................   1       0    seq
    ...........gagagcaugcuagaugaccaca...............................................................................   1       0    seq
    .............gagcaugcuagaugaccaca...............................................................................   11      0    seq
    .................................ccaggaauauuucagagcuggc.........................................................   9       0    seq
    .................................ccaggaauauuucagagcuggcA........................................................   4       1    seq
    ......................................................................uagcacuaaaauuauucuuga.....................   2       0    seq
    ......................................................................uagcacuaaaauuauucuugau....................   25      0    seq
    ......................................................................uagcacuaaaauuauucuugauC...................   2       1    seq
    ......................................................................uagcacuaaauAauucuugauu...................   3       1    seq
    ......................................................................uNgcacuaaaauuauucuugauu...................   1       1    seq
    ......................................................................uagcacuaaauuGuucuugauu...................   1       1    seq
    ......................................................................uagcacuaaaauuauucuugaAu...................   1       1    seq
    ......................................................................uagUacuaaaauuauucuugauu...................   1       1    seq
    ......................................................................uagcacuGaauuauucuugauu...................   5       1    seq
    ......................................................................uagcaAuaaauuauucuugauu...................   1       1    seq
    ......................................................................uagcacuaaaauuauucuugauA...................   1       1    seq
    ......................................................................uagcacuaaaauuauucuCgauu...................   2       1    seq
    ......................................................................uagcacuaaaauuauucuuAauu...................   2       1    seq
    ......................................................................uagcacuaaaauuauucuugauu...................   2042    0    seq
    ......................................................................uagcacuaaaauuauAcuugauu...................   1       1    seq
    ......................................................................Aagcacuaaaauuauucuugauu...................   13      1    seq
    ......................................................................uagcacuaaaauuauucuugauuA..................   2       1    seq
    ........................................................................gcacuaaaauuauucuugauu...................   1       0    seq
```
