# Supplementary material for: The Small RNA Universe of Capitella teleta
Source: Front Mol Biosci. 2022 Feb 25;9:802814. doi: 10.3389/fmolb.2022.802814 (PMC8915122; doi:10.3389/fmolb.2022.802814)
Supplement: Supplementary file 1 [file DataSheet1.ZIP › Supplement/confident/CAPTEscaffold_50_4938.pdf]

Provisional ID	:	CAPTEscaffold_50_4938
Score total	:	343.9
Score for star read(s)	:	3.9
Score for read counts	:	336.6
Score for mfe	:	2.4
Score for randfold	:	1.6
Score for cons. seed	:	-0.6
Total read count	:	672
Mature read count	:	559
Loop read count	:	0
Star read count	:	113

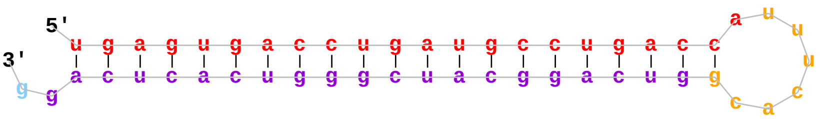

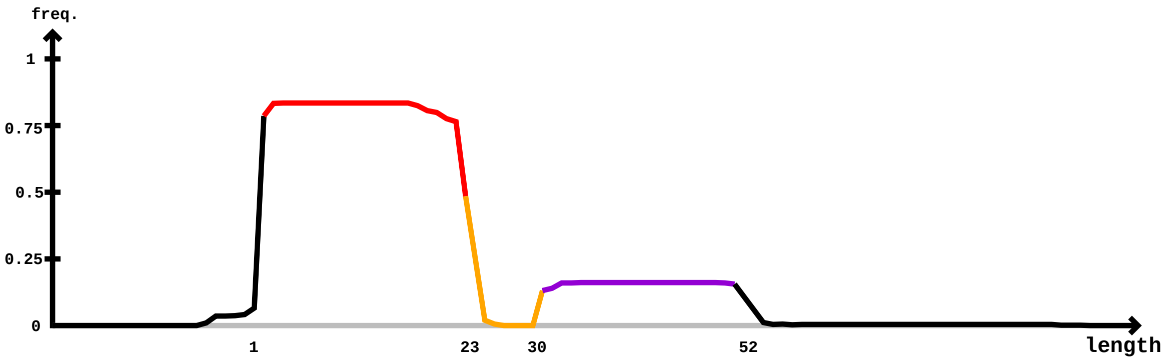

Mature **Star**

```
5'- caguuggcucagccucaccuugagugaccugaugccugaccauuucacggucaggcaucgggucacucaggcagaguguccuccugucagcaucgaugucauugacuacac   -3'   obs
    caguuggcucagccucaccuugagugaccugaugccugaccauuucacggucaggcaucgggucacucaggcagaguguccuccugucagcaucgaugucauugacuacac         exp
    ..((((((...(((((..((((((((((((((((((((((((.......))))))))))))))))))))))))..))).)).......)))))).(((((...)))))......    reads   mm   sample
    ..............ucaccuugagugaccugaugcc...........................................................................    5       0    seq
    ..............ucaccuugagugaccugaugccuA.........................................................................    2       1    seq
    ...............caccuugagugacUugaugcc...........................................................................    1       1    seq
    ...............caccuugagugaccugaugccG..........................................................................    1       1    seq
    ...............caccuugagugaccugaugccu..........................................................................    12      0    seq
    ...............caccuugagugaccugaugccug.........................................................................    2       0    seq
    ...............caccuugagugaccugaugccugaAcau....................................................................    2       1    seq
    .................ccuugagugaccugaugcc...........................................................................    1       0    seq
    ..................cuugagugaccugaugccuga........................................................................    1       0    seq
    ..................cuugagugaccugaugccugacA......................................................................    2       1    seq
    ...................uugagugaccugaugccuga........................................................................    2       0    seq
    ...................uugagugaccugaugccugac.......................................................................    1       0    seq
    ...................uugagugaccugaugccugacc......................................................................    12      0    seq
    ...................uugagugaccugaugccugaAc......................................................................    1       1    seq
    ...................uugagugaccugaugccugacca.....................................................................    1       0    seq
    ....................ugagugaccugaugccug.........................................................................    1       0    seq
    ....................ugagugaccugaugccugG........................................................................    1       1    seq
    ....................ugagugaccugaugccuga........................................................................    12      0    seq
    ....................ugagugaccugaugccugac.......................................................................    7       0    seq
    ....................ugagugaccugaugccugacc......................................................................    171     0    seq
    ....................ugagugaccugaugccugacA......................................................................    1       1    seq
    ....................Agagugaccugaugccugacc......................................................................    1       1    seq
    ....................ugagugaccugaugccuCacc......................................................................    1       1    seq
    ....................ugagugGccugaugccugacca.....................................................................    1       1    seq
    ....................ugagugaccugaugccugaccU.....................................................................    5       1    seq
    ....................ugagugCccugaugccugacca.....................................................................    1       1    seq
    ....................ugagugaccugaugccugacca.....................................................................    256     0    seq
    ....................ugagugaccugaugccugaccC.....................................................................    9       1    seq
    ....................ugaguAaccugaugccugacca.....................................................................    1       1    seq
    ....................uCagugaccugaugccugacca.....................................................................    1       1    seq
    ....................ugagugaccugaugccugaccaG....................................................................    1       1    seq
    ....................ugagugaccugaugccugaccaA....................................................................    19      1    seq
    ....................ugagugaccugaugccugaccau....................................................................    2       0    seq
```

Mature **Star**

```
caguuggcucagccucaccuugagugaccugaugccugaccauuucacggucaggcaucgggucacucaggcagaguguccuccugucagcaucgaugucauugacuacac
....................ugagugaccugaugccugaccaAu...................................................................	5	1	seq
....................ugagugaccugaugccugaccauA...................................................................	1	1	seq
....................ugagugaccugaugccugaccauu...................................................................	4	0	seq
....................ugagugaccugaugccugaccauCu..................................................................	2	1	seq
....................ugagugaccugaugccugaccauuA..................................................................	1	1	seq
....................ugagugaccugaugccugaccauuu..................................................................	1	0	seq
.....................gagugaccugaugccugacc......................................................................	7	0	seq
.....................gagugaccugaugccugaccU.....................................................................	1	1	seq
.....................gagugaccugaugccugaccC.....................................................................	1	1	seq
.....................gagugaccugaugccugacca.....................................................................	23	0	seq
.....................Aagugaccugaugccugacca.....................................................................	1	1	seq
......................agugaccugaugccugaccau....................................................................	1	0	seq
.................................................gucaggcaucgggucacuc...........................................	1	0	seq
.................................................gucaggcaucgggucacuca..........................................	3	0	seq
.................................................gucaggcaucgggucacucag.........................................	64	0	seq
.................................................gucaggcaucgggucacucaC.........................................	1	1	seq
.................................................gucaggcaucgggucacucaU.........................................	1	1	seq
.................................................gucaAgcaucgggucacucagg........................................	2	1	seq
.................................................gucaggcaucgggucacucagA........................................	4	1	seq
.................................................gucaggcaucgggucacucagU........................................	1	1	seq
.................................................gucaggcaucgggucacucagg........................................	15	0	seq
..................................................ucaggcaucgggucacucaggA.......................................	2	1	seq
..................................................ucaggcaucgggucacucaggca......................................	2	0	seq
..................................................ucaggcaucgggucacucaggcU......................................	1	1	seq
..................................................ucaggcaucgggucacucaggcaga....................................	1	0	seq
...................................................caggcaucgggucacucaggc.......................................	12	0	seq
...................................................caggcaucgggucacucaggcU......................................	1	1	seq
...................................................caggcaucgggucacucaggca......................................	1	0	seq
.....................................................Agcaucgggucacucaggcaga....................................	1	1	seq
........................................................................agaguguccuccugucagcaucgaugucauu........	1	0	seq
..........................................................................aguguccuccugucagcaucgaugucauu........	1	0	seq
............................................................................uguccuccugucagcaucgaugucauugac.....	1	0	seq
```
