# Supplementary material for: The Small RNA Universe of Capitella teleta
Source: Front Mol Biosci. 2022 Feb 25;9:802814. doi: 10.3389/fmolb.2022.802814 (PMC8915122; doi:10.3389/fmolb.2022.802814)
Supplement: Supplementary file 1 [file DataSheet1.ZIP › Supplement/confident/CAPTEscaffold_184_13098.pdf]

Provisional ID	:	CAPTEscaffold_184_13098
Score total	:	10900.5
Score for star read(s)	:	3.9
Score for read counts	:	10894.1
Score for mfe	:	1.5
Score for randfold	:	1.6
Score for cons. seed	:	-0.6
Total read count	:	21380
Mature read count	:	14754
Loop read count	:	0
Star read count	:	6626

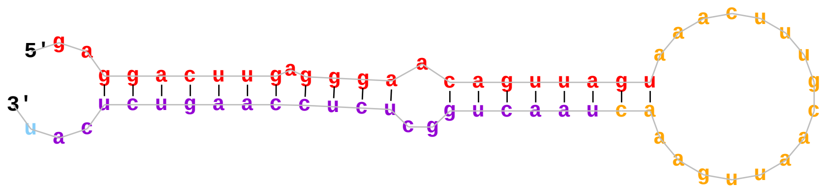

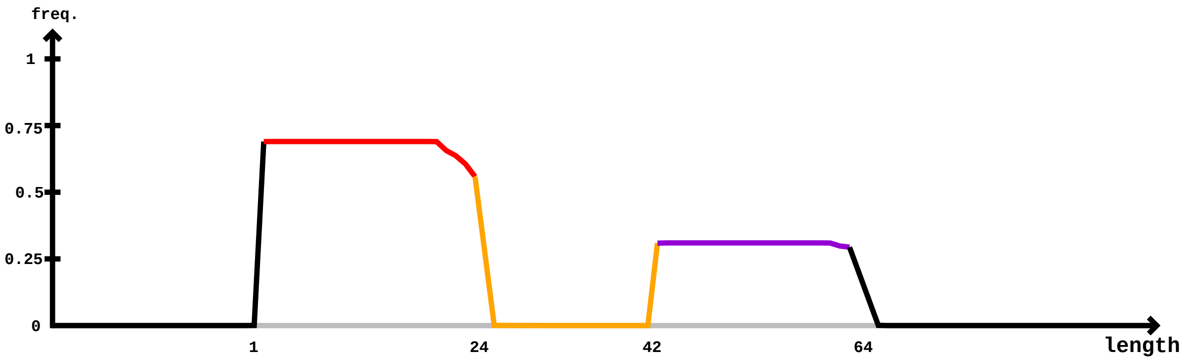

Mature **Star**

```
5'- ggaggaagguggugucuggugaggacuugagggaacaguuaguaaacuuugcaauugaaacuaacuggcucuccaagucucauugcaucccaccacgaaucaacaaugauuau  -3'   obs
    ggaggaagguggugucugguggaggacuugagggaacaguuaguaaacuuugcaauugaaacuaacuggcucuccaagucucauugcaucccaccacgaaucaacaaugauuau        exp
    .......((((.((.(((((((.(((((.((((.((((((((..................))))))))..))))))))))))))).)).)))))...(((((....)))))..   reads    mm     sample
    ...................ugaggacuugagggaacagu..........................................................................   1        0      seq
    ...................ugaggacuugagggaacaguu.........................................................................   1        0      seq
    ...................Cgaggacuugagggaacaguu.........................................................................   2        1      seq
    ...................ugaggacuugagggaacaguuU........................................................................   2        1      seq
    ...................ugaggacuugagggaacaguuag.......................................................................   1        0      seq
    ...................ugaggacuugagggaacaguuagu......................................................................   2        0      seq
    ....................gGggacuugagggaacag...........................................................................   1        1      seq
    ....................gaggacuugagggaacag...........................................................................   5        0      seq
    ....................gaggacuugaggAaacagu..........................................................................   3        1      seq
    ....................Uaggacuugagggaacagu..........................................................................   2        1      seq
    ....................gaggacAugagggaacagu..........................................................................   1        1      seq
    ....................Naggacuugagggaacagu..........................................................................   1        1      seq
    ....................Aaggacuugagggaacagu..........................................................................   6        1      seq
    ....................gaggacuugagggaacagu..........................................................................   697      0      seq
    ....................gaggacuugaAggaacagu..........................................................................   1        1      seq
    ....................gaggacuAgagggaacagu..........................................................................   1        1      seq
    ....................gaggacuugagAgaacagu..........................................................................   2        1      seq
    ....................gaggacuugagAgaacaguu.........................................................................   1        1      seq
    ....................gaggacuugaAggaacaguu.........................................................................   1        1      seq
    ....................gaAgacuugagggaacaguu.........................................................................   1        1      seq
    ....................gaggacuugagggaacaguA.........................................................................   1        1      seq
    ....................gaggacuugagggaaGaguu.........................................................................   1        1      seq
    ....................gaggacuCgagggaacaguu.........................................................................   1        1      seq
    ....................gaggacuugagggaacaguu.........................................................................   406      0      seq
    ....................Caggacuugagggaacaguu.........................................................................   1        1      seq
    ....................gaggacuuAagggaacaguu.........................................................................   1        1      seq
    ....................Uaggacuugagggaacaguu.........................................................................   1        1      seq
    ....................Uaggacuugagggaacaguua........................................................................   2        1      seq
    ....................gaggacuugagggaacaguua........................................................................   638      0      seq
    ....................gaggacuugaAggaacaguua........................................................................   1        1      seq
    ....................gaggacuuAagggaacaguua........................................................................   1        1      seq
    ....................gaggacuugagAgaacaguua........................................................................   3        1      seq
    ....................gaggUcuugagggaacaguua........................................................................   1        1      seq
```

```
                    Mature                                      Star
ggaggaagguggugucugguga ggacuugagggaacaguuaguaaacuuugcaauugaaacuaacuggcucuccaagucucauugcaucccaccacgaaucaacaaugauuau
....................gaggacuAgagggaacaguua........................................................................  1      1      seq
....................gaggacuugagggaacaguCa........................................................................  1      1      seq
....................gaggacuugagggaacaguuU........................................................................  2      1      seq
....................gaAgacuugagggaacaguua........................................................................  1      1      seq
....................Naggacuugagggaacaguua........................................................................  1      1      seq
....................Aaggacuugagggaacaguua........................................................................  4      1      seq
....................gGggacuugagggaacaguuag.......................................................................  1      1      seq
....................gaggacuugagUgaacaguuag.......................................................................  1      1      seq
....................gaggacuugagAgaacaguuag.......................................................................  1      1      seq
....................gaggacuugagCgaacaguuag.......................................................................  2      1      seq
....................gaggacuuAagggaacaguuag.......................................................................  1      1      seq
....................gaggacAugagggaacaguuag.......................................................................  1      1      seq
....................gaggacuugagggaacaguuaU.......................................................................  32     1      seq
....................gaggacuugagggaacagGuag.......................................................................  1      1      seq
....................gaggacuugaggUaacaguuag.......................................................................  1      1      seq
....................gaggacuCgagggaacaguuag.......................................................................  1      1      seq
....................gaggacuugaggAaacaguuag.......................................................................  1      1      seq
....................gaAgacuugagggaacaguuag.......................................................................  2      1      seq
....................gaggacuugagggaacaguuUg.......................................................................  1      1      seq
....................gaggacuugagggaacaguuaA.......................................................................  4      1      seq
....................Uaggacuugagggaacaguuag.......................................................................  2      1      seq
....................Aaggacuugagggaacaguuag.......................................................................  1      1      seq
....................gaggacuugagggaacaguuag.......................................................................  962    0      seq
....................Naggacuugagggaacaguuag.......................................................................  1      1      seq
....................gaggaUuugagggaacaguuagu......................................................................  4      1      seq
....................gaCgacuugagggaacaguuagu......................................................................  1      1      seq
....................gaggacuugagggaaAaguuagu......................................................................  2      1      seq
....................gaggacuugagggaGcaguuagu......................................................................  2      1      seq
....................gaggacuugGgggaacaguuagu......................................................................  3      1      seq
....................gaggacuCgagggaacaguuagu......................................................................  1      1      seq
....................gagCacuugagggaacaguuagu......................................................................  1      1      seq
....................gaggacuugUgggaacaguuagu......................................................................  1      1      seq
....................gaggUcuugagggaacaguuagu......................................................................  1      1      seq
....................gaggacuugagggaacaguuagC......................................................................  4      1      seq
....................gaggacuugagggaacUguuagu......................................................................  1      1      seq
....................gGggacuugagggaacaguuagu......................................................................  3      1      seq
....................gaggacuuAagggaacaguuagu......................................................................  5      1      seq
....................Aaggacuugagggaacaguuagu......................................................................  25     1      seq
....................Uaggacuugagggaacaguuagu......................................................................  13     1      seq
....................gUggacuugagggaacaguuagu......................................................................  2      1      seq
....................gaggacuugaUggaacaguuagu......................................................................  2      1      seq
....................gaggacAugagggaacaguuagu......................................................................  14     1      seq
....................gaggacuugagggaaUaguuagu......................................................................  1      1      seq
....................gaggacuGgagggaacaguuagu......................................................................  1      1      seq
....................gaggacuugaggUaacaguuagu......................................................................  2      1      seq
....................gaggacuugagCgaacaguuagu......................................................................  1      1      seq
....................gaggacuugagUgaacaguuagu......................................................................  2      1      seq
....................gaggacuugagggaacaguuaCu......................................................................  1      1      seq
....................gaggacuugagggUacaguuagu......................................................................  2      1      seq
....................gaggacuugagggaacaguGagu......................................................................  1      1      seq
....................gaggGcuugagggaacaguuagu......................................................................  1      1      seq
....................Caggacuugagggaacaguuagu......................................................................  5      1      seq
....................gaggacCugagggaacaguuagu......................................................................  1      1      seq
....................gaggacuugagggaacaguuUgu......................................................................  1      1      seq
....................gaggacuugagggaacagAuagu......................................................................  3      1      seq
....................gaggacuugagggGacaguuagu......................................................................  3      1      seq
....................gagAacuugagggaacaguuagu......................................................................  2      1      seq
....................gaggacuugCgggaacaguuagu......................................................................  1      1      seq
....................gaggacuugagggaacaguuagu......................................................................  11670  0      seq
....................gaggacuugagggaacaguuaUu......................................................................  2      1      seq
....................gaggacuugaggAaacaguuagu......................................................................  10     1      seq
....................gaAgacuugagggaacaguuagu......................................................................  14     1      seq
....................gaggaGuugagggaacaguuagu......................................................................  1      1      seq
....................gaggacuugagggaacaCuuagu......................................................................  1      1      seq
....................gaggaNuugagggaacaguuagu......................................................................  2      1      seq
....................gaggacuugagggaaGaguuagu......................................................................  1      1      seq
....................gaggacuugagggaacaguuagA......................................................................  26     1      seq
....................gaggacuugagAgaacaguuagu......................................................................  14     1      seq
....................gaggacGugagggaacaguuagu......................................................................  2      1      seq
....................Naggacuugagggaacaguuagu......................................................................  5      1      seq
```

```
                    Mature                                  Star
ggaggaagguggugucuggugaggacuugagggaacaguuaguaaacuuugcaauugaaacuaacuggcucuccaagucucauugcaucccaccacgaaucaacaaugauuau
....................gaggaAuugagggaacaguuagu......................................................................     3     1     seq
....................gaggacuugagggaacaguuGgu......................................................................     1     1     seq
....................gaggacuugaAggaacaguuagu......................................................................     9     1     seq
....................gaggacuugagggaacaguAagu......................................................................     1     1     seq
....................gaggacuuUagggaacaguuagu......................................................................     3     1     seq
....................gaggacuugagggaacaguuagG......................................................................     5     1     seq
....................gaggacuAgagggaacaguuagu......................................................................     11    1     seq
....................gaggacuugagggaacaguuagua.....................................................................     10    0     seq
....................gaggacuugagggaacaguuaguU.....................................................................     21    1     seq
....................gaggacuugagggaacaguuaguaaa...................................................................     2     0     seq
....................gaggacuugagggaacaguuaguaaac..................................................................     4     0     seq
....................gaggacuugagggaacaguuaguaaacu.................................................................     1     0     seq
....................gaggacuugagggaacaguuaguaaacuuu...............................................................     1     0     seq
....................gaggacuugagggaacaguuaguGaacuuu...............................................................     1     1     seq
.....................aggacuugagggaacaguua........................................................................     2     0     seq
.....................aggacuugagggaacaguuagu......................................................................     3     0     seq
......................ggacuugagggaacaguuagu......................................................................     1     0     seq
............................................................uaacuggcucuccaaguc...................................     11    0     seq
............................................................uaacuAgcucuccaagucu..................................     1     1     seq
............................................................uaacuggcucuccaagucu..................................     236   0     seq
............................................................uaacuggcucuccaagAcuc.................................     1     1     seq
............................................................Aaacuggcucuccaagucuc.................................     2     1     seq
............................................................uaacuggcAcuccaagucuc.................................     1     1     seq
............................................................uaacuggcucuccaagucuc.................................     84    0     seq
............................................................uaacugUcucuccaagucuc.................................     1     1     seq
............................................................uGacuggcucuccaagucuca................................     1     1     seq
............................................................uaacuggcucuccaagCcuca................................     1     1     seq
............................................................uaacuggcucuccaagucuUa................................     1     1     seq
............................................................uaUcuggcucuccaagucuca................................     1     1     seq
............................................................Naacuggcucuccaagucuca................................     2     1     seq
............................................................uaacuggcucuccaagucucU................................     45    1     seq
............................................................uaacuggcucucUaagucuca................................     4     1     seq
............................................................uaacuggcucuccaGgucuca................................     2     1     seq
............................................................uaacuggcucuccaagucuca................................     2872  0     seq
............................................................Caacuggcucuccaagucuca................................     2     1     seq
............................................................Aaacuggcucuccaagucuca................................     17    1     seq
............................................................uaacuggcucuAcaagucuca................................     1     1     seq
............................................................uaacuAgcucuccaagucuca................................     2     1     seq
............................................................uaacuggcGcuccaagucuca................................     1     1     seq
............................................................uaacuUgcucuccaagucuca................................     1     1     seq
............................................................uaacuggcCcuccaagucuca................................     1     1     seq
............................................................uaacuggcucuccaagucucG................................     1     1     seq
............................................................uNacuggcucuccaagucuca................................     1     1     seq
............................................................uaacuggcucAccaagucuca................................     1     1     seq
............................................................uaacuggcucGccaagucuca................................     1     1     seq
............................................................Gaacuggcucuccaagucuca................................     2     1     seq
............................................................uaGcuggcucuccaagucuca................................     2     1     seq
............................................................uaacuggcuUuccaagucuca................................     2     1     seq
............................................................uaacuggcuAuccaagucuca................................     2     1     seq
............................................................uaacAggcucuccaagucuca................................     2     1     seq
............................................................uaacuggcucucAaagucuca................................     2     1     seq
............................................................uaacuggcucuccaagucucaA...............................     1     1     seq
............................................................uaacuCgcucuccaagucucau...............................     1     1     seq
............................................................uaacuggcucuccaGgucucau...............................     1     1     seq
............................................................uaacuggcucucAaagucucau...............................     1     1     seq
............................................................uaacuggGucuccaagucucau...............................     1     1     seq
............................................................uaaAuggcucuccaagucucau...............................     2     1     seq
............................................................uaacuAgcucuccaagucucau...............................     1     1     seq
............................................................uaacuggcucuAcaagucucau...............................     1     1     seq
............................................................uaacAggcucuccaagucucau...............................     3     1     seq
............................................................Aaacuggcucuccaagucucau...............................     7     1     seq
............................................................uaacuggcucuccaaCucucau...............................     2     1     seq
............................................................uaacuggcucucUaagucucau...............................     1     1     seq
............................................................uGacuggcucuccaagucucau...............................     1     1     seq
............................................................uUacuggcucuccaagucucau...............................     1     1     seq
............................................................uaUcuggcucuccaagucucau...............................     1     1     seq
............................................................uaacuggcucCccaagucucau...............................     1     1     seq
............................................................uaacuggcucuccaagucucaC...............................     1     1     seq
............................................................uaacuggcucuUcaagucucau...............................     1     1     seq
............................................................uaacuggcucuccaaguUucau...............................     1     1     seq
```

```
                    Mature                  Star
ggaggaagguggugucuggugaggacuugagggaacaguuaguaaacuuugcaauugaaacuaacuggcucuccaagucucauugcaucccaccacgaaucaacaaugauuau
.............................................................uaacuggcucAccaagucucau..............................    2     1    seq
.............................................................uaacuggcucuccaagucucau..............................    1376  0    seq
.............................................................uaacuggcucuccaagucucUu..............................    2     1    seq
.............................................................uaacuggcucuccaCgucucau..............................    1     1    seq
.............................................................uaacuggcucuccaagucAcau..............................    1     1    seq
.............................................................uaacuggcuUuccaagucucau..............................    1     1    seq
.............................................................uaacuggcuAuccaagucucauu.............................    1     1    seq
.............................................................uaacuggcucCccaagucucauu.............................    1     1    seq
.............................................................uaacuggcucuccaagCcucauu.............................    3     1    seq
.............................................................uaacGggcucuccaagucucauu.............................    1     1    seq
.............................................................uaacuggcucuccaagucucauA.............................    157   1    seq
.............................................................Aaacuggcucuccaagucucauu.............................    14    1    seq
.............................................................uaacuggcucuccaagucucaCu.............................    1     1    seq
.............................................................uaacuggcucucAaagucucauu.............................    3     1    seq
.............................................................uaacuggcucuccaaguAucauu.............................    1     1    seq
.............................................................uaacuAgcucuccaagucucauu.............................    1     1    seq
.............................................................uaacuggcucuccaagucucauG.............................    1     1    seq
.............................................................uaacuggcucuccaaguUucauu.............................    1     1    seq
.............................................................uaacugAcucuccaagucucauu.............................    2     1    seq
.............................................................Naacuggcucuccaagucucauu.............................    1     1    seq
.............................................................uaacuggcucuccUagucucauu.............................    1     1    seq
.............................................................uaacuggcucuccaagucucauC.............................    2     1    seq
.............................................................uaacuggcucuccaagucucUuu.............................    5     1    seq
.............................................................uaacuggcucucUaagucucauu.............................    1     1    seq
.............................................................uaacuggUucuccaagucucauu.............................    1     1    seq
.............................................................uaacuggcucuccaagAcucauu.............................    2     1    seq
.............................................................uaacuggcucuAcaagucucauu.............................    1     1    seq
.............................................................uaacugUcucuccaagucucauu.............................    1     1    seq
.............................................................uaacuggcucuccaagucucauu.............................    1670  0    seq
.............................................................uaacuggcucuccaagucucaAu.............................    1     1    seq
.............................................................uaacuggcucuccaagucucauuU............................    15    1    seq
..............................................................aacuggcucuccaagucuca...............................    4     0    seq
..............................................................Uacuggcucuccaagucuca...............................    1     1    seq
..............................................................aacuggcucuccaagucucauA.............................    2     1    seq
..............................................................aacuggcucuccaagucucauu.............................    12    0    seq
```
