# Supplementary material for: The Small RNA Universe of Capitella teleta
Source: Front Mol Biosci. 2022 Feb 25;9:802814. doi: 10.3389/fmolb.2022.802814 (PMC8915122; doi:10.3389/fmolb.2022.802814)
Supplement: Supplementary file 1 [file DataSheet1.ZIP › Supplement/confident/CAPTEscaffold_183_11950.pdf]

Provisional ID	:	CAPTEscaffold_183_11950
Score total	:	133104
Score for star read(s)	:	3.9
Score for read counts	:	133098.1
Score for mfe	:	1
Score for randfold	:	1.6
Score for cons. seed	:	-0.6
Total read count	:	261078
Mature read count	:	260646
Loop read count	:	0
Star read count	:	432

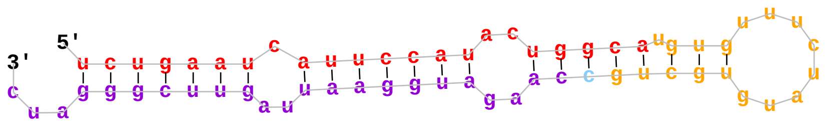

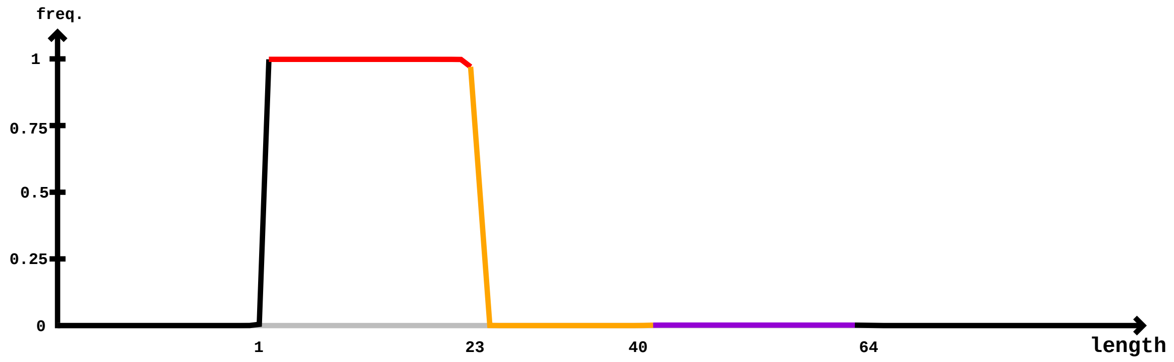

Mature **Star**

```
5'- uacaugcuugaugaucauauucugaaucauuccauacuggcauguguuucuaugugcugccaagauggaauuaguucgggaucugucucaaaagcacugcacguuucaauu   -3'   obs
    uacaugcuugaugaucauauucugaaucauuccauacuggcauguguuucuaugugcugccaagauggaauuaguucgggaucugucucaaaagcacugcacguuucaauu         exp
    ..(((((((..(((.((.(((((((((.(((((((..(((((.(((........))))))))..)))))))..))))))))).))..))).))))).))............   reads   mm   sample
    ..................auucugaaucauuccauacuggc......................................................................   37   0   seq
    ..................Guucugaaucauuccauacuggc......................................................................   1   1   seq
    ..................auucugaaucauuccauacuggca.....................................................................   19   0   seq
    ..................Guucugaaucauuccauacuggca.....................................................................   1   1   seq
    ..................auucugaaucauuccauacuggcaA....................................................................   5   1   seq
    ...................uucugaaucauuccauac..........................................................................   4   0   seq
    ...................uucugaaucauuccauacugg.......................................................................   8   0   seq
    ...................uucuAaaucauuccauacuggc......................................................................   1   1   seq
    ...................uucugaaucauuccauacuggc......................................................................   568   0   seq
    ...................uucugaaucauAccauacuggc......................................................................   1   1   seq
    ...................uucugaaucauuccaAacuggc......................................................................   2   1   seq
    ...................uucugaaucauuccauacAggc......................................................................   1   1   seq
    ...................uucugaGucauuccauacuggc......................................................................   1   1   seq
    ...................uucugaaucauuccauacCggc......................................................................   1   1   seq
    ...................uucugaaucauuccauacuAgc......................................................................   1   1   seq
    ...................uucugaaucauucGauacuggc......................................................................   1   1   seq
    ...................uucugaaucauuccauacuAgca.....................................................................   1   1   seq
    ...................uucugaaucauuccauacugUca.....................................................................   1   1   seq
    ...................uucugaaucauuccauacuggcU.....................................................................   4   1   seq
    ...................uucugaaucauuccauacuggca.....................................................................   409   0   seq
    ...................uucuAaaucauuccauacuggca.....................................................................   1   1   seq
    ...................Cucugaaucauuccauacuggca.....................................................................   6   1   seq
    ...................Gucugaaucauuccauacuggca.....................................................................   1   1   seq
    ...................uucugaaucauuccauacuggcau....................................................................   1   0   seq
    ...................uucugaaucauuccauacuggcaA....................................................................   140   1   seq
    ...................uucugaaucauuccauacuggcaAg...................................................................   2   1   seq
    ....................ucugaaucauuccauacu.........................................................................   32   0   seq
    ....................ucugaaucaAuccauacu.........................................................................   1   1   seq
    ....................ucugaaucauucGauacu.........................................................................   1   1   seq
    ....................ucugaaucauuccauacug........................................................................   11   0   seq
    ....................ucuAaaucauuccauacugg.......................................................................   1   1   seq
    ....................ucugaaucGuuccauacugg.......................................................................   1   1   seq
    ....................ucugaaucauuccauacugC.......................................................................   1   1   seq
```

```
                    Mature                                    Star
uacaugcuugaugaucauauucugaaucauuccauacuggcauguguuucuaugugcugccaagauggaauuaguucgggaucugucucaaaagcacugcacguuucaauu
....................ucugaaucauuccauacugA.......................................................................   2      1   seq
....................ucugaaucauuccauacugU.......................................................................   1      1   seq
....................ucCgaaucauuccauacugg.......................................................................   1      1   seq
....................ucugaaucauuccauacugg.......................................................................   85     0   seq
....................ucugaaucauuccauacugCc......................................................................   1      1   seq
....................ucugaaAcauuccauacuggc......................................................................   3      1   seq
....................ucugaaucauAccauacuggc......................................................................   2      1   seq
....................ucugaaCcauuccauacuggc......................................................................   3      1   seq
....................ucugaaucauuUcauacuggc......................................................................   10     1   seq
....................ucugaaucauuAcauacuggc......................................................................   5      1   seq
....................ucugaaucauuccauacuggU......................................................................   1      1   seq
....................ucugaauUauuccauacuggc......................................................................   4      1   seq
....................ucugaaucaAuccauacuggc......................................................................   4      1   seq
....................ucugaaucauuccauacuggc......................................................................   6471   0   seq
....................ucugaaucauuGcauacuggc......................................................................   1      1   seq
....................ucugaaucauuccaAacuggc......................................................................   2      1   seq
....................ucugaGucauuccauacuggc......................................................................   1      1   seq
....................Ncugaaucauuccauacuggc......................................................................   5      1   seq
....................Gcugaaucauuccauacuggc......................................................................   5      1   seq
....................ucCgaaucauuccauacuggc......................................................................   2      1   seq
....................ucugaaucauuccauacuAgc......................................................................   6      1   seq
....................ucuAaaucauuccauacuggc......................................................................   15     1   seq
....................Ccugaaucauuccauacuggc......................................................................   1      1   seq
....................ucuUaaucauuccauacuggc......................................................................   1      1   seq
....................ucugaaucauuccauacAggc......................................................................   5      1   seq
....................ucAgaaucauuccauacuggc......................................................................   5      1   seq
....................ucugaaucauuccauacCggc......................................................................   1      1   seq
....................ucugaaucauuccaCacuggc......................................................................   1      1   seq
....................ucugaaucaGuccauacuggc......................................................................   1      1   seq
....................ucugaaucauuccUuacuggc......................................................................   1      1   seq
....................ucugaaucauuccauacuggA......................................................................   8      1   seq
....................ucugaUucauuccauacuggc......................................................................   1      1   seq
....................ucugGaucauuccauacuggc......................................................................   1      1   seq
....................ucugaaucauuccauacugAc......................................................................   1      1   seq
....................Acugaaucauuccauacuggc......................................................................   21     1   seq
....................ucugaaucauuccauacugUc......................................................................   1      1   seq
....................ucugaaucauuCAauacuggc......................................................................   5      1   seq
....................ucugaaucUuuccauacuggc......................................................................   1      1   seq
....................ucugaaucauuccauacAggca.....................................................................   71     1   seq
....................ucugaaucauuCAauacuggca.....................................................................   88     1   seq
....................ucugaUucauuccauacuggca.....................................................................   17     1   seq
....................ucugaaucauuccauacugAca.....................................................................   37     1   seq
....................ucugaaucauuccauacuggcU.....................................................................   74     1   seq
....................ucugUaucauuccauacuggca.....................................................................   16     1   seq
....................ucugaaucauuAcauacuggca.....................................................................   107    1   seq
....................ucugaaGcauuccauacuggca.....................................................................   15     1   seq
....................ucugaaucauuccCuacuggca.....................................................................   8      1   seq
....................ucugaaucauAccauacuggca.....................................................................   81     1   seq
....................ucuUaaucauuccauacuggca.....................................................................   20     1   seq
....................ucAgaaucauuccauacuggca.....................................................................   306    1   seq
....................ucugaaucauuUcauacuggca.....................................................................   39     1   seq
....................ucNgaaucauuccauacuggca.....................................................................   3      1   seq
....................ucugaauAauuccauacuggca.....................................................................   48     1   seq
....................ucugaaucauuGcauacuggca.....................................................................   22     1   seq
....................ucugaaucaAuccauacuggca.....................................................................   170    1   seq
....................ucugaaucauuccauUcuggca.....................................................................   23     1   seq
....................ucugaauGauuccauacuggca.....................................................................   3      1   seq
....................ucugaaucauuccauacuggcC.....................................................................   32     1   seq
....................Ncugaaucauuccauacuggca.....................................................................   62     1   seq
....................ucugaaucauuccauacuUgca.....................................................................   9      1   seq
....................ucCgaaucauuccauacuggca.....................................................................   46     1   seq
....................ucugaaucauuccauacuAgca.....................................................................   167    1   seq
....................uNugaaucauuccauacuggca.....................................................................   9      1   seq
....................ucugaauUauuccauacuggca.....................................................................   123    1   seq
....................ucugaaucauuccauaGuggca.....................................................................   9      1   seq
....................ucugaaucauCccauacuggca.....................................................................   36     1   seq
....................ucugaaucauuccauacCggca.....................................................................   25     1   seq
....................ucugaaucauuccauacugUca.....................................................................   22     1   seq
....................ucugaaucauuccaGacuggca.....................................................................   11     1   seq
....................ucugaaucauuccauacuggAa.....................................................................   37     1   seq
```

Mature **Star**

```
uacaugcuugaugaucauauucugaaucauuccauacuggcauguguuucuaugugcugccaagauggaauuaguucgggaucugucucaaaagcacugcacguuucaauu
....................ucugaaucauGccauacuggca.....................................................................	6	1	seq
....................ucGgaaucauuccauacuggca.....................................................................	14	1	seq
....................ucugaaucauuccaCacuggca.....................................................................	23	1	seq
....................Acugaaucauuccauacuggca.....................................................................	715	1	seq
....................ucugaaCcauuccauacuggca.....................................................................	26	1	seq
....................ucuCaaucauuccauacuggca.....................................................................	19	1	seq
....................ucugaaucauuccauacuggGa.....................................................................	35	1	seq
....................ucugaaAcauuccauacuggca.....................................................................	98	1	seq
....................ucugaaucauuccauacuCgca.....................................................................	11	1	seq
....................ucugaaucauuccauCcuggca.....................................................................	9	1	seq
....................ucugaaucauuccUuacuggca.....................................................................	19	1	seq
....................ucugaaucauuccauaUuggca.....................................................................	16	1	seq
....................ucugaaucauuGcauacuggca.....................................................................	18	1	seq
....................uGugaaucauuccauacuggca.....................................................................	8	1	seq
....................ucuAaaucauuccauacuggca.....................................................................	415	1	seq
....................ucugaaucaGuccauacuggca.....................................................................	5	1	seq
....................ucugCaucauuccauacuggca.....................................................................	3	1	seq
....................ucugaaucauuccauaAuggca.....................................................................	19	1	seq
....................uAugaaucauuccauacuggca.....................................................................	36	1	seq
....................ucugaaucaCuccauacuggca.....................................................................	23	1	seq
....................ucugaaucUuuccauacuggca.....................................................................	18	1	seq
....................ucugaGucauuccauacuggca.....................................................................	39	1	seq
....................ucugaaucauuccauacugCca.....................................................................	432	1	seq
....................ucugaaucauuccGuacuggca.....................................................................	34	1	seq
....................Gcugaaucauuccauacuggca.....................................................................	110	1	seq
....................ucugaaucCuuccauacuggca.....................................................................	2	1	seq
....................ucugaaucauuccauacuggca.....................................................................	206842	0	seq
....................uUugaaucauuccauacuggca.....................................................................	32	1	seq
....................ucugaaucauucUauacuggca.....................................................................	288	1	seq
....................ucugaaucauuccauacGggca.....................................................................	9	1	seq
....................ucugaaucauuccauacuggUa.....................................................................	43	1	seq
....................Ccugaaucauuccauacuggca.....................................................................	28	1	seq
....................ucugaaucGuuccauacuggca.....................................................................	31	1	seq
....................ucugaaucauuccauGcuggca.....................................................................	29	1	seq
....................ucugaaucauuccaAacuggca.....................................................................	49	1	seq
....................ucugaaucauuccauacuggcG.....................................................................	141	1	seq
....................ucugGaucauuccauacuggca.....................................................................	32	1	seq
....................ucugaaucGuuccauacuggcau....................................................................	1	1	seq
....................Acugaaucauuccauacuggcau....................................................................	1	1	seq
....................ucugaaucauuccauacuggcaG....................................................................	19	1	seq
....................ucugaaucauuccauacuggcau....................................................................	169	0	seq
....................ucugaaucauuccauacuggcaC....................................................................	234	1	seq
....................ucugaaucauuccauacuggcaA....................................................................	40742	1	seq
....................ucugaaucauuccauacuggcaCg...................................................................	1	1	seq
....................ucugaaucauuccauacuggcauC...................................................................	4	1	seq
....................ucugaaucauuccauacuggcauU...................................................................	30	1	seq
....................ucugaaucauuccauacuggcauA...................................................................	36	1	seq
....................ucugaaucauuccauacuggcaAg...................................................................	23	1	seq
....................ucugaaucauuccauacuggcauAu..................................................................	1	1	seq
....................ucugaaucauuccauacuggcauUu..................................................................	1	1	seq
....................ucugaaucauucUauacuggcauguguuucu............................................................	1	1	seq
.....................cugaaucauuccauacuggc......................................................................	1	0	seq
.....................cugaaucauuccauacuggca.....................................................................	1	0	seq
......................ugaaucauuccauacuggca.....................................................................	13	0	seq
......................uCaaucauuccauacuggca.....................................................................	1	1	seq
......................ugaaucauuccauacuggcaA....................................................................	2	1	seq
......................ugaaucauuccauacuggcaug...................................................................	2	0	seq
.........................................................ugccaagauggaauuaguucggC...............................	1	1	seq
...........................................................ccaagauggaauuaguucggCa..............................	1	1	seq
...........................................................ccaagauggaauuaguucgggau.............................	40	0	seq
...........................................................ccaagauggaauuaguucggCau.............................	56	1	seq
...........................................................ccaagauggaauuaguucgggauA............................	1	1	seq
...........................................................ccaagauggaauuaguucgggauc............................	1	0	seq
...........................................................ccaagauggaauuaguucgggaucA...........................	1	1	seq
............................................................caagauggaauuaguucggga..............................	1	0	seq
............................................................caagauggaauuaguucgggau.............................	36	0	seq
............................................................caagauggaauuaguucggCau.............................	52	1	seq
............................................................caagauggaauuaguuUgggau.............................	1	1	seq
............................................................caagauggaauuaguucgggauc............................	123	0	seq
............................................................caagauggaauuaguucgUgauc............................	1	1	seq
```

```
                    Mature                                  Star
uacaugcuugaugaucauauucugaaucauuccauacuggcauguguuucuaugugcugccaagauggaauuaguucgggaucugucucaaaagcacugcacguuucaauu
............................................................caagauggaauuaguucgggauU............................ 15 1 seq
............................................................caagauggaauuaguucgggauA............................ 1 1 seq
............................................................caagauggaauuaguucggCauc............................ 3 1 seq
............................................................caagauAgaauuaguucgggauc............................ 2 1 seq
............................................................caagauggaauuaguucgggaucA........................... 1 1 seq
............................................................caagauggaauuaguucggCaucu........................... 1 1 seq
............................................................caagauggaauuaguucgggauAu........................... 1 1 seq
............................................................caagauggaauuaguucgggaucu........................... 57 0 seq
.............................................................aagauggaauuaguucgggau............................. 1 0 seq
.............................................................aagauggaauuaguucggCau............................. 1 1 seq
.............................................................aagauggaauuaguucgggauU............................ 1 1 seq
.............................................................aagauggaauuaguucgggauc............................ 6 0 seq
.............................................................aagauggaauuaguucgggaucu........................... 26 0 seq
..............................................................agauggaauuaguucgggaucu........................... 1 0 seq
```
