# Supplementary material for: The Small RNA Universe of Capitella teleta
Source: Front Mol Biosci. 2022 Feb 25;9:802814. doi: 10.3389/fmolb.2022.802814 (PMC8915122; doi:10.3389/fmolb.2022.802814)
Supplement: Supplementary file 1 [file DataSheet1.ZIP › Supplement/confident/CAPTEscaffold_324_18362.pdf]

Provisional ID	:	CAPTEscaffold_324_18362
Score total	:	173.5
Score for star read(s)	:	3.9
Score for read counts	:	168.9
Score for mfe	:	-0.2
Score for randfold	:	1.6
Score for cons. seed	:	-0.6
Total read count	:	343
Mature read count	:	221
Loop read count	:	0
Star read count	:	122

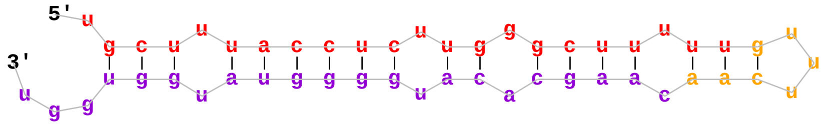

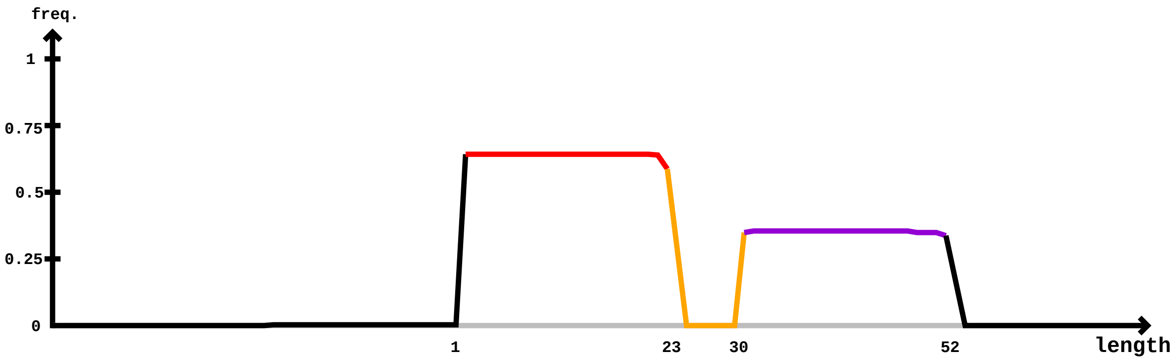


```
                                                 Mature              Star
5'- uccacauuuuaggaugaccaacaauucugcuuggcuuguguugcuuuaccucuugggcuuuuuguuucaacaagcacaugggguaugguggugguaugcccaagucuuugcaa   -3'    obs
    uccacauuuuaggaugaccaacaauucugcuuggcuuguguugcuuuaccucuugggcuuuuuguuucaacaagcacaugggguaugguggugguaugcccaagucuuugcaa          exp
    (((........)))............(((..((((((.((((((.((((((.((.((((.(((...))).)))).)).)))))).))))......)).))))))...))).    reads    mm    sample
    .....................caauucugcuuggcuugugu.......................................................................    1        0     seq
    .........................................ugcuuuaccucuugggcuuu...................................................    1        0     seq
    .........................................ugcuuuaccucuugggcuuuu..................................................    18       0     seq
    .........................................ugcuuuaccucuugggcuuuuu.................................................    150      0     seq
    .........................................ugcuuuaccucuuAggcuuuuu.................................................    2        1     seq
    .........................................ugcuuuaccucuugggUuuuuu.................................................    2        1     seq
    .........................................ugcuuuaccucuugggcuGuuu.................................................    12       1     seq
    .........................................ugcuuAaccucuugggcuuuuu.................................................    1        1     seq
    .........................................ugcuuuaccucuugggcuuuuuU................................................    30       1     seq
    .........................................ugcuuuaccucuugggcuuuuuA................................................    5        1     seq
    ......................................................................caagcacaugggguaugg........................    2        0     seq
    ......................................................................caagcacaugggguauggugg.....................    4        0     seq
    ......................................................................caagcacaugggguaugguggG....................    1        1     seq
    ......................................................................caagcacaugggguaugguAgu....................    48       1     seq
    ......................................................................caagcacaugggguaugguggu....................    60       0     seq
    ......................................................................Gaagcacaugggguaugguggu....................    2        1     seq
    ......................................................................Aaagcacaugggguaugguggu....................    1        1     seq
    ......................................................................caagcacaugggguauggugguA...................    1        1     seq
    ......................................................................caagcacaugggguauggugguU...................    1        1     seq
    .......................................................................aagcacaugggguauggugguA...................    2        1     seq
```
