# Supplementary material for: The Small RNA Universe of Capitella teleta
Source: Front Mol Biosci. 2022 Feb 25;9:802814. doi: 10.3389/fmolb.2022.802814 (PMC8915122; doi:10.3389/fmolb.2022.802814)
Supplement: Supplementary file 1 [file DataSheet1.ZIP › Supplement/confident/CAPTEscaffold_488_22753.pdf]

Provisional ID	:	CAPTEscaffold_488_22753
Score total	:	193144.1
Score for star read(s)	:	3.9
Score for read counts	:	193137.2
Score for mfe	:	2
Score for randfold	:	1.6
Score for cons. seed	:	-0.6
Total read count	:	378842
Mature read count	:	374993
Loop read count	:	0
Star read count	:	3849

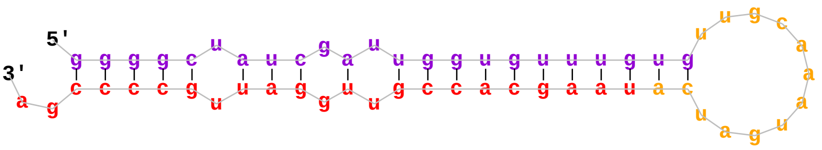

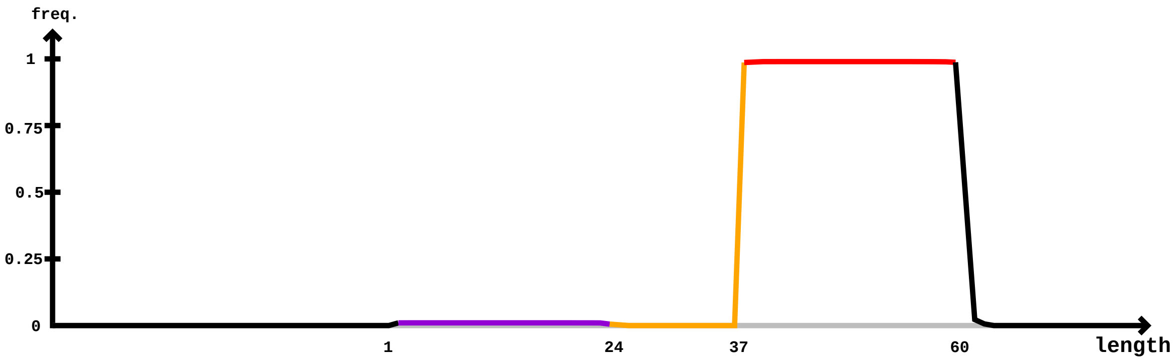


```
                                        Star                                 Mature
5'- cauccuaauuuucggagauguucaucucaguuucggggcuaucgauugguguuuguguugcaaaugaucauaagcaccguuggauugccccgaaauuggaauuugcuaucau   -3'    obs
    cauccuaauuuucggagauguucaucucaguuucggggcuaucgauugguguuuguguugcaaaugaucauaagcaccguuggauugccccgaaauuggaauuugcuaucau          exp
    ..(((........)))(((((...((.((((((((((((.(((.(.(((((((((((...........))))))))))).).))).))))))))))))))....)).)))..    reads    mm    sample
    .................................cggggcuaucgauugguguuugug.......................................................    2        0     seq
    ..................................ggggcuaucgauuggugu............................................................    2        0     seq
    ..................................ggggcuaucgauugguguu...........................................................    13       0     seq
    ..................................ggggcuaucgauugguguuu..........................................................    3        0     seq
    ..................................ggggcuaucgauugguguuug.........................................................    89       0     seq
    ..................................ggggcuaucgauAgguguuug.........................................................    1        1     seq
    ..................................ggggcuaucgauugguguuuA.........................................................    1        1     seq
    ..................................Agggcuaucgauugguguuug.........................................................    1        1     seq
    ..................................ggggcuaucAauugguguuugu........................................................    5        1     seq
    ..................................ggggcuaucgauAgguguuugu........................................................    1        1     seq
    ..................................ggggcuaucgauugguguuAgu........................................................    1        1     seq
    ..................................ggggcuaucgauuggugAuugu........................................................    2        1     seq
    ..................................ggggcuaucgauugguguuugG........................................................    1        1     seq
    ..................................ggggGuaucgauugguguuugu........................................................    1        1     seq
    ..................................Ngggcuaucgauugguguuugu........................................................    1        1     seq
    ..................................ggggcuaucgauugguguuuCu........................................................    1        1     seq
    ..................................Agggcuaucgauugguguuugu........................................................    8        1     seq
    ..................................ggggcuaucgauugguguuugA........................................................    1        1     seq
    ..................................ggggcuaucgauuggAguuugu........................................................    1        1     seq
    ..................................ggggcuauUgauugguguuugu........................................................    4        1     seq
    ..................................ggggcuauAgauugguguuugu........................................................    1        1     seq
    ..................................ggggcuaucgauuAguguuugu........................................................    5        1     seq
    ..................................ggggcuaucgauugguguuCgu........................................................    1        1     seq
    ..................................ggggcuaucgauugguguuugu........................................................    1645     0     seq
    ..................................ggCgcuaucgauugguguuugug.......................................................    1        1     seq
    ..................................ggggcuaucgaAugguguuugug.......................................................    1        1     seq
    ..................................Ugggcuaucgauugguguuugug.......................................................    2        1     seq
    ..................................ggggcuaucgauuAguguuugug.......................................................    6        1     seq
    ..................................ggggcuauAgauugguguuugug.......................................................    1        1     seq
    ..................................ggggcuaucgauugguAuuugug.......................................................    1        1     seq
    ..................................Agggcuaucgauugguguuugug.......................................................    14       1     seq
    ..................................ggggcuaucCauugguguuugug.......................................................    1        1     seq
    ..................................ggggcuaucgauugguguuCgug.......................................................    1        1     seq
```

Star Mature

```
cauccuaauuuucggagauguucaucucaguuucggggcuaucgauugguguuuguguugcaaaugaucauaagcaccguuggauugccccgaaauuggaauuugcuaucau
..................................ggggcuaucgauuggAguuugug.......................................................	3	1	seq
..................................ggggcuaucgauugguguuAgug.......................................................	1	1	seq
..................................ggggcuaucgauugguguuugug.......................................................	1944	0	seq
..................................ggggcuaucgauugUuguuugug.......................................................	1	1	seq
..................................ggAgcuaucgauugguguuugug.......................................................	2	1	seq
..................................ggggcuaucgauugguguAugug.......................................................	1	1	seq
..................................gAggcuaucgauugguguuugug.......................................................	5	1	seq
..................................ggggcuaucgauugguguuuguA.......................................................	4	1	seq
..................................ggggcuaucgauugguguuuAug.......................................................	1	1	seq
..................................ggggcuaucgauugguguCugug.......................................................	1	1	seq
..................................ggggcuaucgauugguguuuguU.......................................................	4	1	seq
..................................ggggcuaucAauugguguuugug.......................................................	10	1	seq
..................................ggggcuauUgauugguguuugug.......................................................	6	1	seq
..................................ggggcuaucgauugguguuugugC......................................................	1	1	seq
..................................ggggcuaucgauugguguuugugA......................................................	6	1	seq
..................................ggggcuaucgauugguguuugugu......................................................	17	0	seq
..................................ggggcuaucgauugguguuugugG......................................................	2	1	seq
..................................ggggcuaucgauugguguuuguguu.....................................................	8	0	seq
..................................ggggcuaucgauugguguuuguguA.....................................................	8	1	seq
..................................ggggcuaucgauugguguuuguguugc...................................................	1	0	seq
...................................gggcuaucgauugguguuugug.......................................................	1	0	seq
...................................gggcuaucgauuAguguuugug.......................................................	1	1	seq
....................................ggcuaucgauugguguuugu........................................................	1	0	seq
.......................................uaucgauugguguuugug.......................................................	1	0	seq
...............................................................augaucauaagcaccguuggau...........................	5	0	seq
................................................................ugaucauaagcaccguuggau...........................	5	0	seq
...................................................................ucauaagcaccguuggauugccA......................	1	1	seq
....................................................................Aauaagcaccguuggauugccccga...................	1	1	seq
.....................................................................Cuaagcaccguuggauugccccga...................	5	1	seq
......................................................................uaagcaccguuggauugc........................	32	0	seq
......................................................................uaagcaccguuggauugcc.......................	38	0	seq
......................................................................uaagcaccguuggauugccA......................	2	1	seq
......................................................................uaagcaccguuAgauugccc......................	1	1	seq
......................................................................uaagcaccguuggauugccc......................	93	0	seq
......................................................................Aaagcaccguuggauugccc......................	2	1	seq
......................................................................uaagcaccguuggauugccAc.....................	2	1	seq
......................................................................uaagcaccguuggauugcccc.....................	131	0	seq
......................................................................uaagcaccguuggauugcccA.....................	1	1	seq
......................................................................uaagcaccguuAgauugcccc.....................	2	1	seq
......................................................................Aaagcaccguuggauugcccc.....................	2	1	seq
......................................................................uaagcaccguuggauugcccAg....................	1	1	seq
......................................................................uaagcaccguuAgauugccccg....................	3	1	seq
......................................................................uaagcacAguuggauugccccg....................	1	1	seq
......................................................................uaagcacUguuggauugccccg....................	1	1	seq
......................................................................uaagcaccguuggauugcccGg....................	2	1	seq
......................................................................uaagcaccguuggauugccccU....................	6	1	seq
......................................................................uaagcaccguuggauugccccg....................	658	0	seq
......................................................................Aaagcaccguuggauugccccg....................	2	1	seq
......................................................................uaagcaccguuggauugUcccg....................	1	1	seq
......................................................................uaagcaccgAuggauugccccg....................	1	1	seq
......................................................................uaagcaccguuggauugccccC....................	3	1	seq
......................................................................uaagcaccguuggauugccAcg....................	1	1	seq
......................................................................uaagcaccguuggauugcccGga...................	42	1	seq
......................................................................uaagcaccguuggauGgccccga...................	3	1	seq
......................................................................uaagcaccguugUauugccccga...................	49	1	seq
......................................................................uaagcaccguuggauugUcccga...................	78	1	seq
......................................................................uaagcaAcguuggauugccccga...................	108	1	seq
......................................................................uaagcaccguuggauugcAccga...................	104	1	seq
......................................................................uaagcacUguuggauugccccga...................	296	1	seq
......................................................................uaagcaccguuAgauugccccga...................	1738	1	seq
......................................................................uaagcaccguuggauugccccCa...................	58	1	seq
......................................................................uaagcaccguuggauuCccccga...................	37	1	seq
......................................................................uaagcaccguuggauugcccUga...................	524	1	seq
......................................................................uaagcaGcguuggauugccccga...................	49	1	seq
......................................................................uaagcaccguugCauugccccga...................	28	1	seq
......................................................................uaagcaUcguuggauugccccga...................	36	1	seq
......................................................................uaagcaccguuggauugccccgN...................	1	1	seq
......................................................................uaagcaccgGuggauugccccga...................	19	1	seq
......................................................................uaCgcaccguuggauugccccga...................	1	1	seq
......................................................................uaagcaccUuuggauugccccga...................	26	1	seq
```

```
                                  Star                             Mature
cauccuaauuuucggagauguucaucucaguuucggggcuaucgauugguguuuguguugcaaaugaucauaagcaccguuggauugccccgaaauuggaauuugcuaucau
......................................................................uaagcaccguuggauugcGccga...................  32      1    seq
......................................................................uaagcaccguuCgauugccccga...................  28      1    seq
......................................................................uaagAaccguuggauugccccga...................  64      1    seq
......................................................................Caagcaccguuggauugccccga...................  35      1    seq
......................................................................uaaUcaccguuggauugccccga...................  28      1    seq
......................................................................uaagcaccguuUgauugccccga...................  41      1    seq
......................................................................uaagcaccguuggauugccAcga...................  611     1    seq
......................................................................uaNgcaccguuggauugccccga...................  2       1    seq
......................................................................uUagcaccguuggauugccccga...................  3       1    seq
......................................................................uaagcNccguuggauugccccga...................  2       1    seq
......................................................................uaagcaccguuggauuAccccga...................  50      1    seq
......................................................................uaagcacGguuggauugccccga...................  10      1    seq
......................................................................uaagcaccguuggGuugccccga...................  53      1    seq
......................................................................uaUgcaccguuggauugccccga...................  14      1    seq
......................................................................uaagcaccgCuggauugccccga...................  36      1    seq
......................................................................uaagcaccCuuggauugccccga...................  16      1    seq
......................................................................Naagcaccguuggauugccccga...................  113     1    seq
......................................................................uaagcaccguuggauugccGcga...................  39      1    seq
......................................................................uaaCcaccguuggauugccccga...................  21      1    seq
......................................................................uaagcCccguuggauugccccga...................  9       1    seq
......................................................................uaagcaccguuggauugccccgG...................  1964    1    seq
......................................................................uaagcaccguuggCuugccccga...................  2       1    seq
......................................................................uaagcacAguuggauugccccga...................  193     1    seq
......................................................................uaagcaccguuggaAugccccga...................  140     1    seq
......................................................................uaagcaccguCggauugccccga...................  63      1    seq
......................................................................uaagcaccgAuggauugccccga...................  207     1    seq
......................................................................uaagcaccguuggauugAcccga...................  68      1    seq
......................................................................uaagcaccguuggauugcUccga...................  130     1    seq
......................................................................uaagcaccguuggauugccccgU...................  246     1    seq
......................................................................Aaagcaccguuggauugccccga...................  1674    1    seq
......................................................................uaagUaccguuggauugccccga...................  39      1    seq
......................................................................uaagcaccguuggauuUccccga...................  30      1    seq
......................................................................uGagcaccguuggauugccccga...................  181     1    seq
......................................................................uaagcaccguuggauAgccccga...................  256     1    seq
......................................................................uaaAcaccguuggauugccccga...................  31      1    seq
......................................................................uaagcaccguuggauugccUcga...................  334     1    seq
......................................................................uaagcaccguuggauCgccccga...................  84      1    seq
......................................................................uaagcaccguAggauugccccga...................  182     1    seq
......................................................................uaagcaccguuggUuugccccga...................  32      1    seq
......................................................................uaagGaccguuggauugccccga...................  18      1    seq
......................................................................uaagcaccguugAauugccccga...................  66      1    seq
......................................................................Gaagcaccguuggauugccccga...................  146     1    seq
......................................................................uaGgcaccguuggauugccccga...................  50      1    seq
......................................................................uaagcGccguuggauugccccga...................  48      1    seq
......................................................................uaagcaccguuggauugcccAga...................  128     1    seq
......................................................................uaagcaccguuggauugccccUa...................  35      1    seq
......................................................................uaagcaccguuggaCugccccga...................  36      1    seq
......................................................................uNagcaccguuggauugccccga...................  21      1    seq
......................................................................uaagcaccguuggaGugccccga...................  4       1    seq
......................................................................uaagcaccguuggauugGcccga...................  17      1    seq
......................................................................uaagcaccguGggauugccccga...................  22      1    seq
......................................................................uaagcaccguuggauugccccga...................  350737  0    seq
......................................................................uaagcaccguuggauugccccgC...................  110     1    seq
......................................................................uaagcUccguuggauugccccga...................  47      1    seq
......................................................................uaagcaccguuAgauugccccgaa..................  12      1    seq
......................................................................uaagcaccguuggauugccccCaa..................  58      1    seq
......................................................................Aaagcaccguuggauugccccgaa..................  11      1    seq
......................................................................uaagcacUguuggauugccccgaa..................  1       1    seq
......................................................................uaagcaccguuggauAgccccgaa..................  1       1    seq
......................................................................uaagAaccguuggauugccccgaa..................  1       1    seq
......................................................................uaagcaccguuggUuugccccgaa..................  1       1    seq
......................................................................uaagcaccguuggaAugccccgaa..................  1       1    seq
......................................................................uaagcaccguuggauugcUccgaa..................  2       1    seq
......................................................................uGagcaccguuggauugccccgaa..................  3       1    seq
......................................................................uaGgcaccguuggauugccccgaa..................  1       1    seq
......................................................................uaagcaccgAuggauugccccgaa..................  1       1    seq
......................................................................uaagcaccguuggauugccccgaU..................  93      1    seq
......................................................................uaagcaccguuUgauugccccgaa..................  1       1    seq
......................................................................uaagcaccgGuggauugccccgaa..................  1       1    seq
......................................................................uaagcaccAuuggauugccccgaa..................  111     1    seq
```

```
                                  Star                                    Mature
cauccuaauuuucggagauguucaucucaguuucggggcuaucgauugguguuuguguugcaaaugaucauaagcaccguuggauugccccgaaauuggaauuugcuaucau
......................................................................uaagcaccguuggauugcccUgaa.................	1	1	seq
......................................................................uaagcaAcguuggauugccccgaa.................	2	1	seq
......................................................................uaagcaccguuggauugccccgaa.................	2124	0	seq
......................................................................uaagcaccguuggauugccccUaa.................	2	1	seq
......................................................................uaagcaccgCuggauugccccgaa.................	2	1	seq
......................................................................uaagcacAguuggauugccccgaa.................	1	1	seq
......................................................................Caagcaccguuggauugccccgaa.................	1	1	seq
......................................................................uaagcaccguuggauugccAcgaa.................	2	1	seq
......................................................................uaagcaccguuggauugccccgUa.................	1	1	seq
......................................................................uaagcaccguAggauugccccgaa.................	1	1	seq
......................................................................uaagcaccguuggauugccUcgaa.................	1	1	seq
......................................................................uaagcacGguuggauugccccgaa.................	1	1	seq
......................................................................uaagcaccguuggauugccccgaG.................	6	1	seq
......................................................................uaagcaccguuggauugcAccgaa.................	1	1	seq
......................................................................uaagcaccguuggauugccccgaC.................	17	1	seq
......................................................................uaagcaccguuggauugccccgCaa................	1	1	seq
......................................................................uaagcaccguAggauugccccgaaa................	4	1	seq
......................................................................uaagcaAcguuggauugccccgaaa................	2	1	seq
......................................................................uaagcaccguuggauugcccUgaaa................	8	1	seq
......................................................................uaagcacUguuggauugccccgaaa................	3	1	seq
......................................................................uaagcaccguuggauugcccAgaaa................	2	1	seq
......................................................................uaagcaccguuggauugccccgaUa................	4	1	seq
......................................................................uaagAaccguuggauugccccgaaa................	1	1	seq
......................................................................uaagcaccguuggauugccccgaCa................	9	1	seq
......................................................................uaagcCccguuggauugccccgaaa................	1	1	seq
......................................................................Naagcaccguuggauugccccgaaa................	1	1	seq
......................................................................uaagcaccguuggauugccccgaaG................	15	1	seq
......................................................................uaagcaccguuAgauugccccgaaa................	22	1	seq
......................................................................uaagcaccguugUauugccccgaaa................	2	1	seq
......................................................................uaagcaccguuggauugccUcgaaa................	4	1	seq
......................................................................uaagcaccguuggauAgccccgaaa................	2	1	seq
......................................................................uaagcaccgAuggauugccccgaaa................	3	1	seq
......................................................................uaagcUccguuggauugccccgaaa................	1	1	seq
......................................................................uaagcaccguuggauugccccCaaa................	24	1	seq
......................................................................uaagcaccguuggauugcAccgaaa................	2	1	seq
......................................................................uaagcacAguuggauugccccgaaa................	2	1	seq
......................................................................uaagcaccguuggauugccccgaaa................	5603	0	seq
......................................................................uaagcGccguuggauugccccgaaa................	1	1	seq
......................................................................uaagcaccguuggauuAccccgaaa................	1	1	seq
......................................................................uaagcaccguuggauugccAcgaaa................	3	1	seq
......................................................................uaagcaccguuggauugGcccgaaa................	1	1	seq
......................................................................uaagcaccguuggaAugccccgaaa................	3	1	seq
......................................................................Caagcaccguuggauugccccgaaa................	3	1	seq
......................................................................Aaagcaccguuggauugccccgaaa................	31	1	seq
......................................................................uaagcaccAuuggauugccccgaaa................	336	1	seq
......................................................................uaagcaccguuggauugccGcgaaa................	1	1	seq
......................................................................Gaagcaccguuggauugccccgaaa................	5	1	seq
......................................................................uaaAcaccguuggauugccccgaaa................	2	1	seq
......................................................................uaagcaccguuggauugccccUaaa................	1	1	seq
......................................................................uaagcaccguuggauugccccgaaU................	14	1	seq
......................................................................uaagcaccguuggauCgccccgaaa................	4	1	seq
......................................................................uaagcaccguuggauugccccgaaC................	34	1	seq
......................................................................uaagcaccguGggauugccccgaaa................	1	1	seq
......................................................................uaagcaccguuUgauugccccgaaa................	2	1	seq
......................................................................uaagcaccguuggGuugccccgaaa................	1	1	seq
......................................................................uaagcaccAuuggauugccccgaaau...............	1	1	seq
......................................................................uaagcaccguuggauugccccgaaaG...............	9	1	seq
......................................................................uaagcaccguuggauugccccgaaaA...............	2293	1	seq
......................................................................uaagcaccguuggauugccccgaaau...............	12	0	seq
......................................................................uaagcaccguuggauugccccgaaaC...............	9	1	seq
......................................................................uaagcaccguuggauugccccgaaaAu..............	5	1	seq
.......................................................................aagcaccguuggauugccccg....................	2	0	seq
.......................................................................aagcaccguuggauuUccccg....................	1	1	seq
.......................................................................aagcaccguuggauugccccga...................	544	0	seq
.......................................................................aagcaccgGuggauugccccga...................	8	1	seq
.......................................................................aagcaccguuggUuugccccga...................	1	1	seq
.......................................................................aagcaccguuAgauugccccga...................	2	1	seq
.......................................................................aagcaccguuggauugAcccga...................	1	1	seq
.......................................................................aUgcaccguuggauugccccga...................	1	1	seq
.......................................................................aagcaccguuggauugccccgG...................	4	1	seq
```

Star Mature

```
cauccuaauuuucggagauguucaucucaguuucggggcuaucgauugguguuugugguugcaaaugaucauaagcaccguuggauugccccgaaauuggaauuugcuaucau
.......................................................................aagcaccguuggauugcccAga...................	1	1	seq
.......................................................................aagcacAguuggauugccccga...................	1	1	seq
.......................................................................aagcaccguuggauugccAcga...................	1	1	seq
.......................................................................aagcaccguuggauugcccUga...................	1	1	seq
.......................................................................aagcaccguuggauugccccUa...................	1	1	seq
.......................................................................aagcaccguuggauugccccgC...................	1	1	seq
.......................................................................aagcaccguuggauugcGccga...................	1	1	seq
.......................................................................aagcaUcguuggauugccccga...................	1	1	seq
.......................................................................aagcaccguuggauugccccCa...................	3	1	seq
.......................................................................aagcaccguuggauuUccccga...................	11	1	seq
.......................................................................aaAcaccguuggauugccccga...................	2	1	seq
.......................................................................aagcaccguuggaAugccccga...................	2	1	seq
.......................................................................aagcaccguugCauugccccga...................	1	1	seq
.......................................................................Uagcaccguuggauugccccga...................	32	1	seq
.......................................................................aagcaccguuggauugccUcga...................	1	1	seq
.......................................................................aagcaccguuggauugccccgaU..................	1	1	seq
.......................................................................aagcaccguuggauugccccgaa..................	5	0	seq
.......................................................................aagcaccAuuggauugccccgaa..................	1	1	seq
.......................................................................Uagcaccguuggauugccccgaa..................	1	1	seq
.......................................................................aagcaccAuuggauugccccgaaa.................	3	1	seq
.......................................................................aagcaccguuggauugccccgaaa.................	4	0	seq
.......................................................................aagcaccguuggauugccccgaaaA................	1	1	seq
........................................................................agcaccguuggauugccc......................	5	0	seq
........................................................................agcaccguuggauugccccg....................	1	0	seq
........................................................................agcaccguuggauugccccU....................	1	1	seq
........................................................................agcaccguuUgauugccccga...................	1	1	seq
........................................................................agcaccguuggauugccccga...................	545	0	seq
........................................................................Ugcaccguuggauugccccga...................	1	1	seq
........................................................................agcaccguuggaAugccccga...................	1	1	seq
........................................................................agcaccguuggauugccccgG...................	3	1	seq
........................................................................Ggcaccguuggauugccccga...................	1	1	seq
........................................................................agcaccguuggauugccAcga...................	1	1	seq
........................................................................agcaccAuuggauugccccgaa..................	2	1	seq
........................................................................agcaccguuggauugccccgaa..................	13	0	seq
........................................................................agcaccguuggauugccccgaU..................	20	1	seq
........................................................................agcaccguuAgauugccccgaa..................	2	1	seq
........................................................................agcaccguuggauugcccUgaa..................	1	1	seq
........................................................................agcaccguuggauugccccgaUa.................	1	1	seq
........................................................................agcaccguuggauugccccgaaU.................	1	1	seq
........................................................................agcaccAuuggauugccccgaaa.................	1	1	seq
........................................................................agcaccguuggauugccccgaaa.................	4	0	seq
........................................................................agcaccguuggauugccccgaaaA................	1	1	seq
.........................................................................gcaccguuggauugccccg....................	1	0	seq
.........................................................................gcaccguuAgauugccccga...................	1	1	seq
.........................................................................gcaccguuggauugccccga...................	18	0	seq
.........................................................................gcaccgAuggauugccccga...................	1	1	seq
.........................................................................gcaccAuuggauugccccgaa..................	1	1	seq
.........................................................................gcaccguuggauugccccgaaa.................	1	0	seq
.........................................................................gcaccguuggauugccccgaaaA................	1	1	seq
..........................................................................caccguuggauugccccga...................	11	0	seq
..........................................................................caccguuggauugccccgaaaA................	2	1	seq
...........................................................................accgAuggauugccccga...................	1	1	seq
...........................................................................accguuggauugccccga...................	19	0	seq
...........................................................................accguuggauugccccgaaa.................	2	0	seq
```
