# Supplementary material for: The Small RNA Universe of Capitella teleta
Source: Front Mol Biosci. 2022 Feb 25;9:802814. doi: 10.3389/fmolb.2022.802814 (PMC8915122; doi:10.3389/fmolb.2022.802814)
Supplement: Supplementary file 1 [file DataSheet1.ZIP › Supplement/confident/CAPTEscaffold_445_21721.pdf]

Provisional ID	:	CAPTEscaffold_445_21721
Score total	:	26185.6
Score for star read(s)	:	3.9
Score for read counts	:	26178.6
Score for mfe	:	2
Score for randfold	:	1.6
Score for cons. seed	:	-0.6
Total read count	:	51360
Mature read count	:	50789
Loop read count	:	0
Star read count	:	571

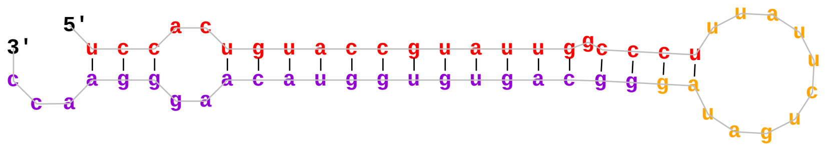

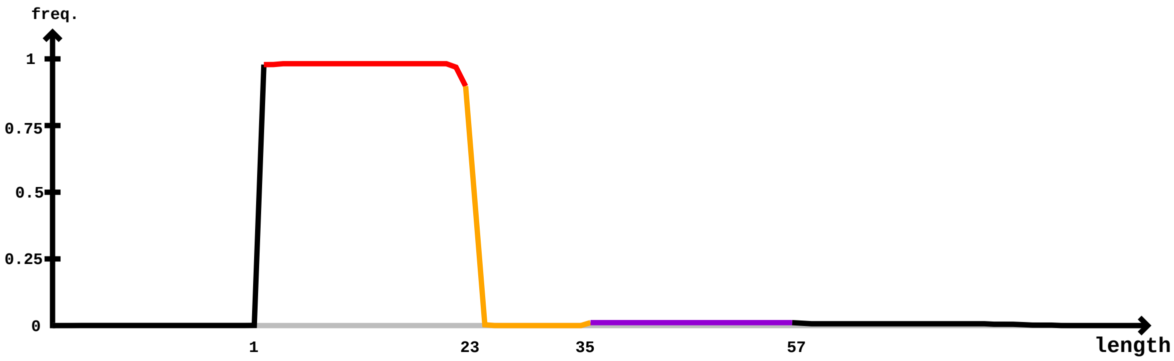

Mature Star

```
5'- uaguucugguaucagcagggUCCACUGUACCGUAUUGGCCCUuuauucugauagggcagugugguacaagggaacccugaggagaccaugggcagcaauuuuuauuucucau -3'   obs
    uaguucugguaucagcagggUCCACUGUACCGUAUUGGCCCUuuauucugauagggcagugugguacaagggaacccugaggagaccaugggcagcaauuuuuauuucucau        exp
    ..((((((((.((..((((((((..((((((((((((.((((..........))))))))))))))))..)).))))))..)).)))).))))...................   reads   mm   sample
    uaguucugguaucagcagAg............................................................................................   1       1    seq
    uaguucugguaucagcaggg............................................................................................   5       0    seq
    .aguucugguaucagcaggg............................................................................................   10      0    seq
    ...................guccacuguaccguauuggccc.......................................................................   2       0    seq
    ...................Cuccacuguaccguauuggcccu......................................................................   11      1    seq
    ...................Cuccacuguaccguauuggcccuu.....................................................................   1       1    seq
    ....................uccacuguaccguauuggc.........................................................................   6       0    seq
    ....................Gccacuguaccguauuggcc........................................................................   1       1    seq
    ....................uccacuguaccguauAggcc........................................................................   1       1    seq
    ....................Accacuguaccguauuggcc........................................................................   3       1    seq
    ....................uccacuguacUguauuggcc........................................................................   1       1    seq
    ....................uccacuguGccguauuggcc........................................................................   1       1    seq
    ....................uccacuguaccguauuggcc........................................................................   645     0    seq
    ....................uccacuguaccguauuggcU........................................................................   2       1    seq
    ....................uccacuguaccguauuggcA........................................................................   10      1    seq
    ....................ucUacuguaccguauuggcc........................................................................   1       1    seq
    ....................uccacuguaAcguauuggcc........................................................................   1       1    seq
    ....................uccacuguaccguauuggccG.......................................................................   1       1    seq
    ....................uccacuguaccgGauuggccc.......................................................................   1       1    seq
    ....................uccacuguaccguaAuggccc.......................................................................   2       1    seq
    ....................ucAacuguaccguauuggccc.......................................................................   1       1    seq
    ....................uccacuguaccguauugAccc.......................................................................   1       1    seq
    ....................uccacuguacUguauuggccc.......................................................................   1       1    seq
    ....................uccacuguaccgAauuggccc.......................................................................   1       1    seq
    ....................uccacuguaccguauuAgccc.......................................................................   2       1    seq
    ....................uccacuguaccguauuggUcc.......................................................................   1       1    seq
    ....................Nccacuguaccguauuggccc.......................................................................   1       1    seq
    ....................uccacuguaccguauuggccA.......................................................................   12      1    seq
    ....................uccacuCuaccguauuggccc.......................................................................   2       1    seq
    ....................uccacuguaccguauuggccU.......................................................................   26      1    seq
    ....................Cccacuguaccguauuggccc.......................................................................   1       1    seq
    ....................Accacuguaccguauuggccc.......................................................................   20      1    seq
    ....................uccacuguacAguauuggccc.......................................................................   1       1    seq
```

```
                    Mature                              Star
uaguucugguaucagcagggUCCACUGUACCGUAUUGGCCCUuuauucugauagggcagugugguacaagggaacccugaggagaccaugggcagcaauuuuuauuucucau
....................uccacuguGccguauuggccc.......................................................................  1     1  seq
....................uccacuguaccguauugCccc.......................................................................  1     1  seq
....................uccacAguaccguauuggccc.......................................................................  2     1  seq
....................uUcacuguaccguauuggccc.......................................................................  1     1  seq
....................Gccacuguaccguauuggccc.......................................................................  4     1  seq
....................uccacugAaccguauuggccc.......................................................................  3     1  seq
....................uccacuguaccguauuUgccc.......................................................................  2     1  seq
....................uccacuguaccguauuggccc.......................................................................  3581  0  seq
....................ucUacuguaccguauuggccc.......................................................................  6     1  seq
....................uccGcuguaccguauuggccc.......................................................................  1     1  seq
....................uccacuAuaccguauuggccc.......................................................................  1     1  seq
....................uccacuguaccguauAggccc.......................................................................  2     1  seq
....................uccacuguaccguauuggcAc.......................................................................  2     1  seq
....................uccGcuguaccguauuggcccu......................................................................  4     1  seq
....................uccacuguaccguauuAgcccu......................................................................  26    1  seq
....................uccacAguaccguauuggcccu......................................................................  22    1  seq
....................uccacuguaccguGuuggcccu......................................................................  3     1  seq
....................uccacuguaccguauuggccAu......................................................................  13    1  seq
....................uccacuguGccguauuggcccu......................................................................  7     1  seq
....................uccacuguaGcguauuggcccu......................................................................  1     1  seq
....................uccacuguaccgAauuggcccu......................................................................  8     1  seq
....................uccacuguaccguauuCgcccu......................................................................  2     1  seq
....................Cccacuguaccguauuggcccu......................................................................  7     1  seq
....................uccaAuguaccguauuggcccu......................................................................  1     1  seq
....................ucUacuguaccguauuggcccu......................................................................  61    1  seq
....................uccacuguaccguauugCcccu......................................................................  6     1  seq
....................uccacuguaccguauugUcccu......................................................................  2     1  seq
....................Nccacuguaccguauuggcccu......................................................................  13    1  seq
....................uccacuguaccCuauuggcccu......................................................................  2     1  seq
....................uccacCguaccguauuggcccu......................................................................  3     1  seq
....................uGcacuguaccguauuggcccu......................................................................  1     1  seq
....................uccacugCaccguauuggcccu......................................................................  4     1  seq
....................uAcacuguaccguauuggcccu......................................................................  7     1  seq
....................uccacuguaccguauGggcccu......................................................................  1     1  seq
....................uccCcuguaccguauuggcccu......................................................................  2     1  seq
....................uccacuguaccguaAuggcccu......................................................................  8     1  seq
....................uccacugGaccguauuggcccu......................................................................  2     1  seq
....................uccacuguaccguauuggccUu......................................................................  22    1  seq
....................uccacuguacUguauuggcccu......................................................................  22    1  seq
....................uccacuguaccguauuggcAcu......................................................................  47    1  seq
....................uccacuguaccgGauuggcccu......................................................................  3     1  seq
....................uccacuguaccguauCggcccu......................................................................  9     1  seq
....................uccacuguaccguauuggcGcu......................................................................  4     1  seq
....................uccacuguaccguauuggcccC......................................................................  37    1  seq
....................ucAacuguaccguauuggcccu......................................................................  8     1  seq
....................uccacuguaccAuauuggcccu......................................................................  6     1  seq
....................uccacugAaccguauuggcccu......................................................................  17    1  seq
....................uUcacuguaccguauuggcccu......................................................................  6     1  seq
....................uccacNguaccguauuggcccu......................................................................  1     1  seq
....................uccacuguaccguauuggccGu......................................................................  2     1  seq
....................uccacuguacAguauuggcccu......................................................................  15    1  seq
....................uccacuguaccguauuUgcccu......................................................................  3     1  seq
....................uNcacuguaccguauuggcccu......................................................................  2     1  seq
....................uccacuUuaccguauuggcccu......................................................................  2     1  seq
....................uccacuguaccUuauuggcccu......................................................................  4     1  seq
....................uccacuguaccguauuggAccu......................................................................  9     1  seq
....................uccacuguaccguaCuggcccu......................................................................  1     1  seq
....................uccacuguaccguauuggUccu......................................................................  3     1  seq
....................Accacuguaccguauuggcccu......................................................................  198   1  seq
....................uccacuguaccguauuggcccA......................................................................  187   1  seq
....................Gccacuguaccguauuggcccu......................................................................  36    1  seq
....................uccaUuguaccguauuggcccu......................................................................  2     1  seq
....................uccacuguaccgCauuggcccu......................................................................  3     1  seq
....................uccacuguUccguauuggcccu......................................................................  6     1  seq
....................uccacuguaccguUuuggcccu......................................................................  4     1  seq
....................uccUcuguaccguauuggcccu......................................................................  3     1  seq
....................uccacuAuaccguauuggcccu......................................................................  8     1  seq
....................uccacuguaccguauuggGccu......................................................................  2     1  seq
....................uccacuguaccguauuggcccG......................................................................  263   1  seq
....................uccacuCuaccguauuggcccu......................................................................  6     1  seq
```

```
                    Mature                            Star
uaguucugguaucagcagggaccacuguaccguauuggcccuuuauucugauagggcagugugguacaagggaacccugaggagaccaugggcagcaauuuuuauuucucau
....................uccacuguaccguauuggcccu......................................................................	38865	0	seq
....................uccacuguaccguauugAcccu......................................................................	14	1	seq
....................uccaGuguaccguauuggcccu......................................................................	1	1	seq
....................uccacuguaccguauuggcUcu......................................................................	9	1	seq
....................uccacuguaAcguauuggcccu......................................................................	9	1	seq
....................uccacuguaccguauAggcccu......................................................................	29	1	seq
....................uccaAuguaccguauuggcccuu.....................................................................	1	1	seq
....................uccacuguaccguauAggcccuu.....................................................................	3	1	seq
....................uccacuguaccguauuAgcccuu.....................................................................	3	1	seq
....................uccacuguaccguaCuggcccuu.....................................................................	1	1	seq
....................uccacuguaccgAauuggcccuu.....................................................................	2	1	seq
....................uccacuguaccguauuggcccuG.....................................................................	5	1	seq
....................uccacugAaccguauuggcccuu.....................................................................	2	1	seq
....................uccacuguaccguauuggcAcuu.....................................................................	7	1	seq
....................uccacuguaccguauuggccUuu.....................................................................	2	1	seq
....................uccacuguaccguauugAcccuu.....................................................................	1	1	seq
....................uccacuguaccguauuggAccuu.....................................................................	3	1	seq
....................uccGcuguaccguauuggcccuu.....................................................................	1	1	seq
....................uccacuguaccguauuggcccuC.....................................................................	2	1	seq
....................uccacuguaccUuauuggcccuu.....................................................................	1	1	seq
....................uccacuguaccguauuggcccAu.....................................................................	3	1	seq
....................uccacuguaccguauuUgcccuu.....................................................................	1	1	seq
....................Nccacuguaccguauuggcccuu.....................................................................	1	1	seq
....................uccacAguaccguauuggcccuu.....................................................................	2	1	seq
....................uccUcuguaccguauuggcccuu.....................................................................	1	1	seq
....................uccacuguaccguauuggcccuA.....................................................................	570	1	seq
....................uccacuguaccguaGuggcccuu.....................................................................	1	1	seq
....................uccacugCaccguauuggcccuu.....................................................................	1	1	seq
....................uccCcuguaccguauuggcccuu.....................................................................	1	1	seq
....................ucAacuguaccguauuggcccuu.....................................................................	1	1	seq
....................Gccacuguaccguauuggcccuu.....................................................................	4	1	seq
....................uccacuguaccAuauuggcccuu.....................................................................	3	1	seq
....................ucUacuguaccguauuggcccuu.....................................................................	4	1	seq
....................uccacuguacAguauuggcccuu.....................................................................	4	1	seq
....................uccacuguaccguauuggcccCu.....................................................................	3	1	seq
....................Accacuguaccguauuggcccuu.....................................................................	32	1	seq
....................uccacuUuaccguauuggcccuu.....................................................................	1	1	seq
....................uccacuguaccguauuggcUcuu.....................................................................	2	1	seq
....................uccacuguaccguauCggcccuu.....................................................................	2	1	seq
....................uccacuguacUguauuggcccuu.....................................................................	1	1	seq
....................uccacuguaccguauuggcccuu.....................................................................	5342	0	seq
....................uccacuguaUcguauuggcccuu.....................................................................	1	1	seq
....................uUcacuguaccguauuggcccuu.....................................................................	1	1	seq
....................uccacuguaccguaAuggcccuu.....................................................................	1	1	seq
....................uccacuguaccguauuCgcccuu.....................................................................	1	1	seq
....................Accacuguaccguauuggcccuuu....................................................................	2	1	seq
....................uccacuguaccguauuggcccuuu....................................................................	140	0	seq
....................ucUacuguaccguauuggcccuuu....................................................................	1	1	seq
....................uccacuguaccguauuggcccuuA....................................................................	2	1	seq
....................uccacuguaccguauuggcccuuC....................................................................	1	1	seq
....................uccacuguaccguauuggcccuAu....................................................................	6	1	seq
....................uccacuguaccguauuggcccuuuU...................................................................	1	1	seq
....................uccacuguaccguauuggcccuAua...................................................................	3	1	seq
....................uccacuguaccguauuggcccuuuUu..................................................................	1	1	seq
.....................ccacuguaccguauuggcc........................................................................	1	0	seq
.....................Ucacuguaccguauuggccc.......................................................................	1	1	seq
.....................ccacuguaccguauuggcccu......................................................................	6	0	seq
.....................Ucacuguaccguauuggcccu......................................................................	6	1	seq
.....................Ucacuguaccguauuggcccuu.....................................................................	1	1	seq
......................cacuguaccguauuggcc........................................................................	2	0	seq
......................cacuguaccguauuggccc.......................................................................	4	0	seq
......................cacuguaccguauuggcccu......................................................................	93	0	seq
......................cacuguaccguauuggcccG......................................................................	1	1	seq
......................cacuguaccguauuggcccuu.....................................................................	56	0	seq
......................cacuguaccguauuggcccuA.....................................................................	2	1	seq
......................................................ggcagugugguacaGgggaacc....................................	1	1	seq
......................................................ggcagugugguaGaagggaacc....................................	1	1	seq
......................................................ggcagugugguacaagAgaacc....................................	1	1	seq
......................................................ggcaguCugguacaagggaacc....................................	1	1	seq
......................................................ggcagugugguacaagggaacc....................................	548	0	seq
```

```
                    Mature                           Star
uaguucugguaucagcagggaccacuguaccguauuggcccuuuauucugauagggcagugugguacaagggaacccugaggagaccaugggcagcaauuuuuauuucucau
......................................................ggcagAgugguacaagggaacc....................................     2     1     seq
......................................................Cgcagugugguacaagggaacc....................................     1     1     seq
......................................................ggcagugugguacaaAggaacc....................................     2     1     seq
......................................................ggcaguguAguacaagggaacc....................................     1     1     seq
......................................................ggcagugugguacaagggaaccU...................................     12    1     seq
.......................................................gcagugugguacaagggaaccU...................................     1     1     seq
............................................................................Uugaggagaccaugggcagc................     1     1     seq
............................................................................cugaggagaccaugggcagc................     90    0     seq
............................................................................cugaggagaccaugggcagcaa..............     9     0     seq
............................................................................cugaggagaccaugggcagcaau.............     77    0     seq
............................................................................cugaggagaccaugggcagcaauA............     84    1     seq
............................................................................cugaggagaccaugggcagcaauuu...........     3     0     seq
............................................................................cugaggagaccaugggcagcaauuA...........     3     1     seq
............................................................................cugaggagaccaugggcagcaauuuA..........     1     1     seq
............................................................................cugaggagaccaugggcagcaauuuu..........     1     0     seq
............................................................................cugaggagaccaugggcagcaauuuuu.........     1     0     seq
............................................................................cugaggagaccaugggcagcaauuuuA.........     76    1     seq
............................................................................cugaggagaccaugggcagcaauuuuuU........     1     1     seq
............................................................................cugaggagaccaugggcagcaauuuuua........     3     0     seq
............................................................................cugaggagaccaugggcagcaauuuuuaA.......     1     1     seq
```
