# Supplementary material for: The Small RNA Universe of Capitella teleta
Source: Front Mol Biosci. 2022 Feb 25;9:802814. doi: 10.3389/fmolb.2022.802814 (PMC8915122; doi:10.3389/fmolb.2022.802814)
Supplement: Supplementary file 1 [file DataSheet1.ZIP › Supplement/confident/CAPTEscaffold_158_9964.pdf]

Provisional ID	:	CAPTEscaffold_158_9964
Score total	:	43592.3
Score for star read(s)	:	3.9
Score for read counts	:	43585.6
Score for mfe	:	1.8
Score for randfold	:	1.6
Score for cons. seed	:	-0.6
Total read count	:	85503
Mature read count	:	61107
Loop read count	:	9
Star read count	:	24387

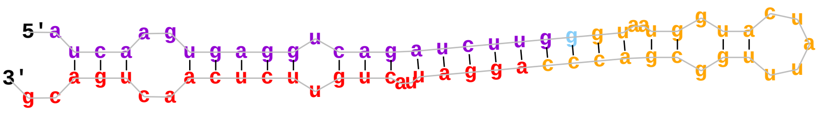

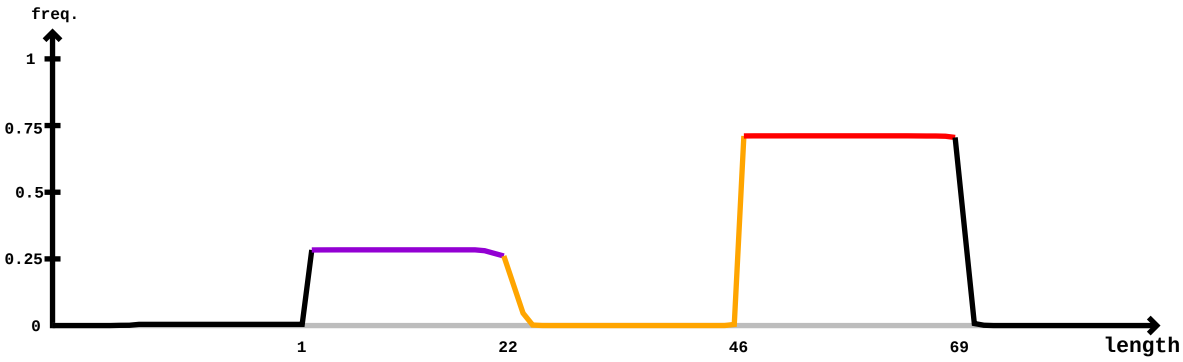


```
                       Star                                        Mature
5'- gugaguguuucuagcgucuuccagcaucaagugaggucagaucuugggguaaugguacuauuuggcgacccaggauuacuguucucaacugacgcuggcagacugcucucuuug  -3'   obs
    gugaguguuucuagcgucuuccagcaucaagugaggucagaucuugggguaaugguacuauuuggcgacccaggauuacuguucucaacugacgcuggcagacugcucucuuug        exp
    ..(((.......(((((((.(((((.(((..(((((.((((((((((((..((.((.....)).)))))))))))..))).)))))..))).))))).)))).))))))....   reads      mm      sample
    ....guguuucuagcgucuuccagc........................................................................................   1          0       seq
    .....Aguuucuagcgucuuccag.........................................................................................   1          1       seq
    .....uguuucuagcgucuuccagc........................................................................................   79         0       seq
    ......guuucuagcgucuuccagc........................................................................................   16         0       seq
    .......Auucuagcgucuuccagc........................................................................................   1          1       seq
    .......uuucuagcgucuuccagc........................................................................................   291        0       seq
    ........................caucaagugaggucagaucuug...................................................................   1          0       seq
    ........................caucaagugaggucagaucuugg..................................................................   1          0       seq
    .........................aucaagugaggucagauA......................................................................   1          1       seq
    .........................aucaUgugaggucagauc......................................................................   1          1       seq
    .........................aucaagugaggucagauc......................................................................   275        0       seq
    .........................aAcaagugaggucagauc......................................................................   1          1       seq
    .........................aucaagugaggucagaucA.....................................................................   3          1       seq
    .........................aAcaagugaggucagaucu.....................................................................   1          1       seq
    .........................aucaagugaggucagCucu.....................................................................   1          1       seq
    .........................aucaagugaggucagaucG.....................................................................   1          1       seq
    .........................aucaagugaggucagauAu.....................................................................   1          1       seq
    .........................aucaagugaggucagaucu.....................................................................   845        0       seq
    .........................aucaagugaggucagaAcu.....................................................................   2          1       seq
    .........................aucGagugaggucagaucu.....................................................................   1          1       seq
    .........................aucaaguCaggucagaucuu....................................................................   1          1       seq
    .........................aucaagugaggucagaucuu....................................................................   810        0       seq
    .........................aucaagugaggAcagaucuu....................................................................   1          1       seq
    .........................aucaagugaggucagCucuu....................................................................   1          1       seq
    .........................auUaagugaggucagaucuu....................................................................   2          1       seq
    .........................aucaagugaggucagGucuu....................................................................   1          1       seq
    .........................aucaagugaggucGgaucuu....................................................................   1          1       seq
    .........................aucaagugagUucagaucuu....................................................................   1          1       seq
    .........................aucaaguAaggucagaucuu....................................................................   1          1       seq
    .........................aucaagugaggucagaucuG....................................................................   1          1       seq
    .........................aucUagugaggucagaucuu....................................................................   1          1       seq
    .........................aucaagAgaggucagaucuu....................................................................   2          1       seq
    .........................aAcaagugaggucagaucuu....................................................................   1          1       seq
```

Star Mature

```
gugaguguuucuagcgucuuccagcaucaagugaggucagaucuuggguaaugguacuauuuggcgacccaggauuacuguucucaacugacgcuggcagacugcucucuuug
.........................aucaagugaggucagaucuA....................................................................	18	1	seq
.........................aucaagugaggucagaAcuug...................................................................	5	1	seq
.........................aucaagugaggucagUucuug...................................................................	2	1	seq
.........................aucaagugaggucagaucNug...................................................................	1	1	seq
.........................aucaagugUggucagaucuug...................................................................	2	1	seq
.........................aucaagugaggucagaucuCg...................................................................	5	1	seq
.........................aucUagugaggucagaucuug...................................................................	1	1	seq
.........................aucaaguAaggucagaucuug...................................................................	10	1	seq
.........................aucaagugagguAagaucuug...................................................................	2	1	seq
.........................Uucaagugaggucagaucuug...................................................................	1	1	seq
.........................aucaGgugaggucagaucuug...................................................................	1	1	seq
.........................aCcaagugaggucagaucuug...................................................................	3	1	seq
.........................aucaagugaggCcagaucuug...................................................................	1	1	seq
.........................aucaagugagCucagaucuug...................................................................	1	1	seq
.........................auAaagugaggucagaucuug...................................................................	6	1	seq
.........................aucaagAgaggucagaucuug...................................................................	16	1	seq
.........................aucGagugaggucagaucuug...................................................................	5	1	seq
.........................aucaagugaggucagaucAug...................................................................	5	1	seq
.........................aucaagugGggucagaucuug...................................................................	1	1	seq
.........................aucaagugaggucagGucuug...................................................................	4	1	seq
.........................aucaUgugaggucagaucuug...................................................................	1	1	seq
.........................aucaagugaggucagaucuGg...................................................................	7	1	seq
.........................Nucaagugaggucagaucuug...................................................................	2	1	seq
.........................aAcaagugaggucagaucuug...................................................................	12	1	seq
.........................aucaagugaggucagauAuug...................................................................	2	1	seq
.........................aucaagugaggucagaucuuC...................................................................	2	1	seq
.........................aucaagugaggAcagaucuug...................................................................	6	1	seq
.........................aucaaAugaggucagaucuug...................................................................	1	1	seq
.........................aucaagugaggucUgaucuug...................................................................	1	1	seq
.........................aucaagugaggucagaCcuug...................................................................	4	1	seq
.........................aucaagugaCgucagaucuug...................................................................	1	1	seq
.........................aucaagugagUucagaucuug...................................................................	1	1	seq
.........................aucaagGgaggucagaucuug...................................................................	2	1	seq
.........................aucaagugaUgucagaucuug...................................................................	2	1	seq
.........................Gucaagugaggucagaucuug...................................................................	6	1	seq
.........................auUaagugaggucagaucuug...................................................................	4	1	seq
.........................aucaagugaggucagaucuuA...................................................................	76	1	seq
.........................aucaaUugaggucagaucuug...................................................................	7	1	seq
.........................aucaagugaAgucagaucuug...................................................................	7	1	seq
.........................aucaagugaggGcagaucuug...................................................................	1	1	seq
.........................aucaagugagguGagaucuug...................................................................	3	1	seq
.........................aucaagugaggucaCaucuug...................................................................	1	1	seq
.........................aucaagugaggucagaucuuU...................................................................	10	1	seq
.........................aucaagugaggucaUaucuug...................................................................	1	1	seq
.........................aucaagugaggucagaucuug...................................................................	10264	0	seq
.........................aucaaguNaggucagaucuug...................................................................	1	1	seq
.........................aucaagugaggucagaucuugC..................................................................	3	1	seq
.........................aucaUgugaggucagaucuugg..................................................................	1	1	seq
.........................aucaagugaggAcagaucuugg..................................................................	5	1	seq
.........................aucaagugaggucagGucuugg..................................................................	2	1	seq
.........................aucaagugagUucagaucuugg..................................................................	1	1	seq
.........................aucaagugaggCcagaucuugg..................................................................	3	1	seq
.........................Gucaagugaggucagaucuugg..................................................................	13	1	seq
.........................aucaagAgaggucagaucuugg..................................................................	6	1	seq
.........................aucaagugagCucagaucuugg..................................................................	1	1	seq
.........................aucaagugaggucagaucuAgg..................................................................	3	1	seq
.........................aucaagugaggucagaucuugg..................................................................	7595	0	seq
.........................aucaagugaggucagaucuCgg..................................................................	1	1	seq
.........................aucaagugagguUagaucuugg..................................................................	3	1	seq
.........................aucaagugaggucagaucuugU..................................................................	126	1	seq
.........................aucaagugaggucagaAcuugg..................................................................	5	1	seq
.........................Nucaagugaggucagaucuugg..................................................................	1	1	seq
.........................aucaagugaggucagaucuuAg..................................................................	24	1	seq
.........................aucaagugaggucagauAuugg..................................................................	1	1	seq
.........................aucaaguAaggucagaucuugg..................................................................	22	1	seq
.........................aCcaagugaggucagaucuugg..................................................................	2	1	seq
.........................aucaagugagguAagaucuugg..................................................................	1	1	seq
.........................aucaGgugaggucagaucuugg..................................................................	1	1	seq
.........................aucaagugaCgucagaucuugg..................................................................	1	1	seq
.........................aucaagugaggucagaucuugA..................................................................	45	1	seq
```

```
                         Star                    Mature
gugaguguuucuagcgucuuccagcaucaagugaggucagaucuuggguaaugguacuauuuggcgacccaggauuacuguucucaacugacgcuggcagacugcucucuuug
.........................aAcaagugaggucagaucuugg..................................................................  12    1  seq
.........................aucaagugaggucagaucuuCg..................................................................  1     1  seq
.........................auAaagugaggucagaucuugg..................................................................  3     1  seq
.........................aucaaguUaggucagaucuugg..................................................................  1     1  seq
.........................aucaagugGggucagaucuugg..................................................................  1     1  seq
.........................aucaagGgaggucagaucuugg..................................................................  1     1  seq
.........................aucaagugUggucagaucuugg..................................................................  1     1  seq
.........................aucaagugaggucagaucGugg..................................................................  2     1  seq
.........................aucaagugaggucagaucCugg..................................................................  2     1  seq
.........................aucaagugagguGagaucuugg..................................................................  1     1  seq
.........................aucaagugaggucGgaucuugg..................................................................  1     1  seq
.........................aucaagugaAgucagaucuugg..................................................................  3     1  seq
.........................aucGagugaggucagaucuugg..................................................................  2     1  seq
.........................auUaagugaggucagaucuugg..................................................................  4     1  seq
.........................aucaagugagguGagaucuuggg.................................................................  1     1  seq
.........................aucaagCgaggucagaucuuggg.................................................................  1     1  seq
.........................aCcaagugaggucagaucuuggg.................................................................  1     1  seq
.........................aucaagAgaggucagaucuuggg.................................................................  1     1  seq
.........................aucaagugaggucagaucuuggA.................................................................  3534  1  seq
.........................aucaagugaggucagaucuuggg.................................................................  150   0  seq
.........................aucaagugaggucagaucuuggU.................................................................  144   1  seq
.........................aucaagugaggucagaucuuggC.................................................................  13    1  seq
.........................aucaagugaggucagaucuugggA................................................................  14    1  seq
.........................aucaagugaggucagaucuuggAu................................................................  39    1  seq
.........................aucaagugaggucagaucuugggu................................................................  74    0  seq
.........................aucaagugaggucagaucuuggUu................................................................  1     1  seq
..........................ucaagugaggucagaucuug...................................................................  1     0  seq
..........................ucaagugaggucagaucuugg..................................................................  1     0  seq
..........................ucaagugaggucagaucuugggu................................................................  14    0  seq
..........................ucaagugaggucagaucuuggguU...............................................................  1     1  seq
...........................caagugaggucagaucuuggg.................................................................  1     0  seq
...........................caagugaggucagaucuugggu................................................................  10    0  seq
...........................caagugaggucagaucuuggguU...............................................................  1     1  seq
............................aagugaggucagaucuug...................................................................  2     0  seq
............................aagugaggucagaucuugg..................................................................  2     0  seq
............................aagugaggucagaucuugggu................................................................  5     0  seq
............................aagugaggucagaucuugggua...............................................................  1     0  seq
...........................................uuggguaaugguacuaCuuggcgaccc...........................................  1     1  seq
...............................................guaaugguacuauuuggcgaccc...........................................  8     0  seq
................................................uaaugguacuauuuggcgacc............................................  1     0  seq
....................................................................ccaggauuacuguucucaacugac.....................  1     0  seq
....................................................................Ucaggauuacuguucucaacugacg....................  5     1  seq
....................................................................ccaggauuacuguucucaacugacg....................  5     0  seq
.....................................................................caggauuacuguucucaacugac.....................  7     0  seq
.....................................................................caggauuacuguucucaacugacg....................  326   0  seq
.....................................................................caggauUcuguucucaacugacg....................  1     1  seq
.....................................................................caggauuacuguucucaacugacgc...................  5     0  seq
......................................................................aggauuacuguucucGac.........................  1     1  seq
......................................................................aggauuacuguucucaac.........................  28    0  seq
......................................................................aggauuacuguucucaacu........................  27    0  seq
......................................................................aggauuacuguucucaacugG......................  2     1  seq
......................................................................aggauuacCguucucaacuga......................  2     1  seq
......................................................................aggauuacuguucucaacuga......................  64    0  seq
......................................................................aggauuacuguucucaacuAa......................  1     1  seq
......................................................................aggauuacugAucucaacuga......................  1     1  seq
......................................................................aAgauuacuguucucaacugac.....................  1     1  seq
......................................................................aggauAacuguucucaacugac.....................  1     1  seq
......................................................................aggauuacuguucucaacugaA.....................  1     1  seq
......................................................................aggaAuacuguucucaacugac.....................  1     1  seq
......................................................................aggauuacuguucuGaacugac.....................  1     1  seq
......................................................................aggauuacuguucucaacugac.....................  373   0  seq
......................................................................aggauuacuguucucGacugac.....................  1     1  seq
......................................................................aggaAuacuguucucaacugacg....................  43    1  seq
......................................................................aggauuacuguucucaacuCacg....................  1     1  seq
......................................................................aggauuacuguucucaacugCcg....................  2     1  seq
......................................................................aggauAacuguucucaacugacg....................  26    1  seq
......................................................................aggauuacugCucucaacugacg....................  6     1  seq
......................................................................aggauuacuguucucaacugaGg....................  5     1  seq
......................................................................Uggauuacuguucucaacugacg....................  3     1  seq
......................................................................aggauuacCguucucaacugacg....................  8     1  seq
```

Star Mature

```
gugaguguuucuagcgucuuccagcaucaagugaggucagaucuugguaaugguacuauuuggcgacccaggauuacuguucucaacugacgcuggcagacugcucucuuug
......................................................................aggauuacuguUucaacugacg....................  7      1  seq
......................................................................aggauuacAguucucaacugacg....................  12     1  seq
......................................................................aggauuaGuguucucaacugacg....................  3      1  seq
......................................................................Nggauuacuguucucaacugacg....................  18     1  seq
......................................................................aggauCacuguucucaacugacg....................  13     1  seq
......................................................................aggauuacuguucucaacugGcg....................  7      1  seq
......................................................................aggauuacuguGcucaacugacg....................  2      1  seq
......................................................................aggauGacuguucucaacugacg....................  1      1  seq
......................................................................aggGuuacuguucucaacugacg....................  16     1  seq
......................................................................agAauuacuguucucaacugacg....................  4      1  seq
......................................................................aggauuacuguucCcaacugacg....................  12     1  seq
......................................................................aggauuacuguucucaGcugacg....................  8      1  seq
......................................................................aggauuacuguucuAaacugacg....................  16     1  seq
......................................................................aggauuacuguucucaacugacC....................  3      1  seq
......................................................................aggauuacugGucucaacugacg....................  7      1  seq
......................................................................aggauuacuAuucucaacugacg....................  7      1  seq
......................................................................aggauNacuguucucaacugacg....................  1      1  seq
......................................................................aggauuacuguucucaacugacA....................  237    1  seq
......................................................................aggauuacuguucucaacugacg....................  56720  0  seq
......................................................................aUgauuacuguucucaacugacg....................  5      1  seq
......................................................................Gggauuacuguucucaacugacg....................  21     1  seq
......................................................................aggauuacuguuGucaacugacg....................  1      1  seq
......................................................................aggauuacugAucucaacugacg....................  28     1  seq
......................................................................aggauuacuguucucaacugaAg....................  13     1  seq
......................................................................aggauuacuguucucaacugacU....................  25     1  seq
......................................................................aggauuacuguucucaacAgacg....................  24     1  seq
......................................................................aAgauuacuguucucaacugacg....................  75     1  seq
......................................................................aggauuacuguucucaacugUcg....................  3      1  seq
......................................................................aggauuUcuguucucaacugacg....................  9      1  seq
......................................................................aggauuacuguucuUaacugacg....................  31     1  seq
......................................................................aggauuacuguucuGaacugacg....................  2      1  seq
......................................................................aggauuaUuguucucaacugacg....................  6      1  seq
......................................................................agUauuacuguucucaacugacg....................  2      1  seq
......................................................................aggauuacuguCcucaacugacg....................  10     1  seq
......................................................................aggauuaAuguucucaacugacg....................  2      1  seq
......................................................................aggauuacuguucucaaUugacg....................  5      1  seq
......................................................................aggauuacuCuucucaacugacg....................  5      1  seq
......................................................................aggauuacuguucucGacugacg....................  5      1  seq
......................................................................aggauuacuguucucaaAugacg....................  9      1  seq
......................................................................aggauuacuguucucaacGgacg....................  3      1  seq
......................................................................aggauuCcuguucucaacugacg....................  1      1  seq
......................................................................aggaGuacuguucucaacugacg....................  6      1  seq
......................................................................aggUuuacuguucucaacugacg....................  7      1  seq
......................................................................aggauuacuguuAucaacugacg....................  10     1  seq
......................................................................aggauuacuguucucaUcugacg....................  5      1  seq
......................................................................aggauuacuguucucUacugacg....................  8      1  seq
......................................................................aggauuacuUuucucaacugacg....................  7      1  seq
......................................................................aggauuacGguucucaacugacg....................  2      1  seq
......................................................................aggaCuacuguucucaacugacg....................  4      1  seq
......................................................................aggauuacuguAcucaacugacg....................  14     1  seq
......................................................................aggauuacuguucucaacuAacg....................  17     1  seq
......................................................................aggauuacuguucucaacCgacg....................  88     1  seq
......................................................................aggauuacuguucucaacuUacg....................  2      1  seq
......................................................................aggauuacuguucAcaacugacg....................  12     1  seq
......................................................................aggauuGcuguucucaacugacg....................  12     1  seq
......................................................................aCgauuacuguucucaacugacg....................  6      1  seq
......................................................................aggauuacuguucucaaGugacg....................  3      1  seq
......................................................................aggauuacuguucucaacugaUg....................  19     1  seq
......................................................................aggaCuacuguucucaacugacgc...................  1      1  seq
......................................................................aggauuacuguucucaacugacgc...................  1787   0  seq
......................................................................aggauuacAguucucaacugacgc...................  2      1  seq
......................................................................aggauuacCguucucaacugacgc...................  1      1  seq
......................................................................aggauuacugAucucaacugacgc...................  1      1  seq
......................................................................aAgauuacuguucucaacugacgc...................  2      1  seq
......................................................................aggauuacuguucucaacugaAgc...................  1      1  seq
......................................................................aggaAuacuguucucaacugacgc...................  1      1  seq
......................................................................aggauuacuguucucaacugacgU...................  58     1  seq
......................................................................aggauuacuguucucaacuAacgc...................  1      1  seq
......................................................................aggauuacuguucucaacugacgG...................  1      1  seq
......................................................................aggGuuacuguucucaacugacgc...................  1      1  seq
```

Star Mature

```
gugaguguuucuagcgucuuccagcaucaagugaggucagaucuuggguaaugguacuauuuggcgacccaggauuacuguucucaacugacgcuggcagacugcucucuuug
......................................................................aggauuacuguucuAaacugacgc...................   1    1   seq
......................................................................aggauuacuAuucucaacugacgc...................   1    1   seq
......................................................................aggauuacuguucucaacAgacgc...................   2    1   seq
......................................................................aggauuacGguucucaacugacgcu..................   1    1   seq
......................................................................aggauuacuguucucaacugacgcA..................   330  1   seq
......................................................................aggauuacuguucucaacugacgcu..................   229  0   seq
......................................................................aggauuacuguucucaacugacgcC..................   3    1   seq
......................................................................Uggauuacuguucucaacugacgcu..................   1    1   seq
......................................................................aggauuacuguucucaacugacgcuU.................   99   1   seq
.......................................................................ggauuacuguucucaacugacg....................   17   0   seq
.......................................................................Agauuacuguucucaacugacg....................   2    1   seq
........................................................................gauuacuguucucaacugacgc...................   1    0   seq
.........................................................................auuacuguucucaacugacg....................   2    0   seq
..........................................................................uuacuguucucaacugacg....................   1    0   seq
...........................................................................uacuguucucaacugacg....................   10   0   seq
..............................................................................................uggcagacugcucucuuug   2    0   seq
```
