# Supplementary material for: The Small RNA Universe of Capitella teleta
Source: Front Mol Biosci. 2022 Feb 25;9:802814. doi: 10.3389/fmolb.2022.802814 (PMC8915122; doi:10.3389/fmolb.2022.802814)
Supplement: Supplementary file 1 [file DataSheet1.ZIP › Supplement/confident/CAPTEscaffold_488_22723.pdf]

Provisional ID	:	CAPTEscaffold_488_22723
Score total	:	5510.1
Score for star read(s)	:	3.9
Score for read counts	:	5503.2
Score for mfe	:	2
Score for randfold	:	1.6
Score for cons. seed	:	-0.6
Total read count	:	10806
Mature read count	:	9821
Loop read count	:	0
Star read count	:	985

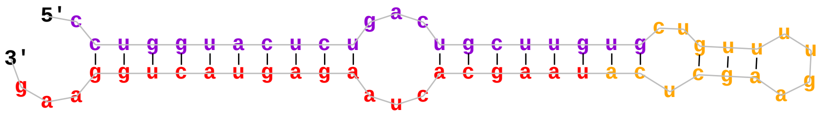

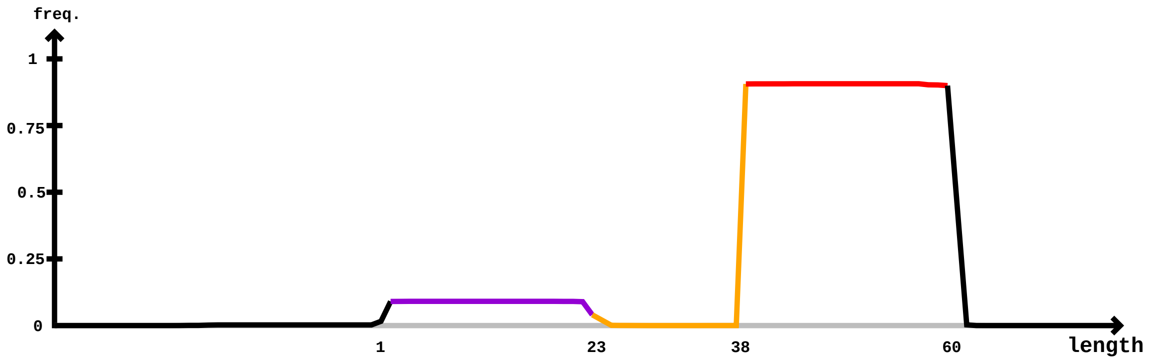

Star Mature

```
5'- augguuauucgacaaugaucgggucuugccuuuccugguacucugacugcuugugcuguuuugaagcucauaagcacuaagaguacuggaagaggcagggucuaaaacu  -3'  obs
    augguuauucgacaaugaucgggucuugccuuuccugguacucugacugcuugugcuguuuugaagcucauaagcacuaagaguacuggaagaggcagggucuaaaacu        exp
    ..(((((((....)))))))(((((((((((((.((((((((((...((((((((..(((....))).))))))))...)))))))))).)))))))))))))......   reads  mm  sample
    ...........acaaugaucgggucuugccuuu............................................................................   1      0   seq
    ............caaugaucAggucuugccuuu............................................................................   1      1   seq
    ............caaugaucgggucuugccuuu............................................................................   4      0   seq
    ..............augaucgggucuugccuuu............................................................................   14     0   seq
    ...............ugaucgggucuugccuuu............................................................................   6      0   seq
    ................................uccugguacucugacugcu..........................................................   1      0   seq
    ................................uccugguacucugacugcuug........................................................   5      0   seq
    ................................uccugguacucugacugcuugA.......................................................   1      1   seq
    ................................uccugguacucugacugcuugu.......................................................   140    0   seq
    .................................ccugguacucugacugcu..........................................................   2      0   seq
    .................................ccugguacucugacugcuu.........................................................   1      0   seq
    .................................ccugguacucugacugcuug........................................................   6      0   seq
    .................................ccugguacucugacugcuugu.......................................................   389    0   seq
    .................................ccugguacucCgacugcuugu.......................................................   1      1   seq
    .................................ccugguacucugacugcuugug......................................................   383    0   seq
    .................................ccugguacucugacugcuGgug......................................................   1      1   seq
    .................................ccugguacucugacugcuCgug......................................................   1      1   seq
    .................................ccugguacucCgacugcuugug......................................................   5      1   seq
    .................................ccuAguacucugacugcuugug......................................................   2      1   seq
    .................................ccuggAacucugacugcuugug......................................................   1      1   seq
    .................................ccugguacucugacugcuugugc.....................................................   4      0   seq
    .................................ccugguacucugacugcuugugA.....................................................   26     1   seq
    .................................ccugguacucugacugcuugugG.....................................................   3      1   seq
    .................................ccugguacucugacugcuugugcu....................................................   3      0   seq
    .................................ccugguacucugacugcuugugcug...................................................   1      0   seq
    .................................ccugguacucugacugcuugugcugu..................................................   2      0   seq
    .................................ccugguacucugacugcuugugcuguu.................................................   1      0   seq
    ..................................cugguacucugacugcuugu.......................................................   1      0   seq
    ..................................cugguacucugacugcuugug......................................................   1      0   seq
    ..................................cugguacucugacugcuugugA.....................................................   1      1   seq
    ...................................ugguacucugacugcuugug......................................................   1      0   seq
    ...................................ugguacucugacugcuugugcu....................................................   2      0   seq
    ..................................................................cucauaagcacuaagaguacugg....................   1      0   seq
```

Star   Mature

```
augguuauucgacaaugaucgggucuugccuuuccugguacucugacugcuuguggcuguuuugaagcucauaagcacuaagaguacuggaagaggcagggucuaaaacu
......................................................................uaagcacuaagaguacugg....................    24      0       seq
......................................................................uaagcaUuaagaguacugg....................    17      1       seq
......................................................................uaagcacuaagaguacugga...................    7       0       seq
......................................................................uaagcacuaagaguacuggaa..................    27      0       seq
......................................................................uaagcGcuaagaguacuggaag.................    1       1       seq
......................................................................uaagcacuaagGguacuggaag.................    1       1       seq
......................................................................Naagcacuaagaguacuggaag.................    5       1       seq
......................................................................uaagcacuaagaguacuggaGg.................    2       1       seq
......................................................................uaagcacuaagaguacugCaag.................    1       1       seq
......................................................................uaagcacuaagaCuacuggaag.................    2       1       seq
......................................................................uaagcUcuaagaguacuggaag.................    3       1       seq
......................................................................uaagcacCaagaguacuggaag.................    2       1       seq
......................................................................uaagcacuaaAaguacuggaag.................    4       1       seq
......................................................................uaagcacuaagaguacAggaag.................    5       1       seq
......................................................................Caagcacuaagaguacuggaag.................    2       1       seq
......................................................................uaagcacuaagaguacCggaag.................    1       1       seq
......................................................................uaagcacuaagaguaUuggaag.................    1       1       seq
......................................................................uaagcacuaagaguacGggaag.................    1       1       seq
......................................................................Gaagcacuaagaguacuggaag.................    5       1       seq
......................................................................uaagcacuGagaguacuggaag.................    11      1       seq
......................................................................uaagcaUuaagaguacuggaag.................    4       1       seq
......................................................................uaGgcacuaagaguacuggaag.................    2       1       seq
......................................................................uaagGacuaagaguacuggaag.................    1       1       seq
......................................................................uaagcacuaagaguacuggGag.................    2       1       seq
......................................................................Aaagcacuaagaguacuggaag.................    53      1       seq
......................................................................uaagcacuaagaguaAuggaag.................    1       1       seq
......................................................................uCagcacuaagaguacuggaag.................    1       1       seq
......................................................................uaagcacuaagaUuacuggaag.................    2       1       seq
......................................................................uaagcacuaagaguacuggaUg.................    1       1       seq
......................................................................uaaUcacuaagaguacuggaag.................    2       1       seq
......................................................................uaagcacuaagaguacuggaag.................    9447    0       seq
......................................................................uaagcaGuaagaguacuggaag.................    1       1       seq
......................................................................uaagcacuaagaguacugAaag.................    2       1       seq
......................................................................uaagcacuaagaguacugUaag.................    1       1       seq
......................................................................uaagcacuaagaguacuggaaC.................    5       1       seq
......................................................................uaagcacuaagaguacuggaaU.................    1       1       seq
......................................................................uaaAcacuaagaguacuggaag.................    2       1       seq
......................................................................uaagcacuaaUaguacuggaag.................    1       1       seq
......................................................................uaagcacuaagUguacuggaag.................    1       1       seq
......................................................................uaagcacuaagaguacuAgaag.................    21      1       seq
......................................................................uaagcacuaagaguUcuggaag.................    1       1       seq
......................................................................uaagcacuaagagAacuggaag.................    6       1       seq
......................................................................uaagcacAaagaguacuggaag.................    3       1       seq
......................................................................uaagcacuaagaguacuggaaA.................    10      1       seq
......................................................................uNagcacuaagaguacuggaag.................    1       1       seq
......................................................................uaagcacuaagagCacuggaag.................    1       1       seq
......................................................................uGagcacuaagaguacuggaag.................    1       1       seq
......................................................................uaagcacuaagaguacuggGaga................    1       1       seq
......................................................................uaagcacuaagaguacuggaagC................    3       1       seq
......................................................................uaagcacuaagaguacuggaagG................    1       1       seq
......................................................................uaagcacuaagaguacuggaaga................    74      0       seq
......................................................................uaagcaUuaagaguacuggaaga................    1       1       seq
......................................................................uaagcacuaagaguacuggaagU................    9       1       seq
......................................................................uaagcacuaagaguacuggaagaC...............    2       1       seq
......................................................................uaagcacuaagaguacuggaagaA...............    26      1       seq
......................................................................uaagcacuaagaguacuggaagagA..............    1       1       seq
.......................................................................aagcacuaagaguacuggaag.................    3       0       seq
.........................................................................gcacuaagaguacuggaag.................    1       0       seq
..........................................................................acuaagaguacuggaaga.................    3       0       seq
```
