# Supplementary material for: The Small RNA Universe of Capitella teleta
Source: Front Mol Biosci. 2022 Feb 25;9:802814. doi: 10.3389/fmolb.2022.802814 (PMC8915122; doi:10.3389/fmolb.2022.802814)
Supplement: Supplementary file 1 [file DataSheet1.ZIP › Supplement/confident/CAPTEscaffold_15480_45258.pdf]

Provisional ID	:	CAPTEscaffold_15480_45258
Score total	:	1680.5
Score for star read(s)	:	3.9
Score for read counts	:	1674.4
Score for mfe	:	1.2
Score for randfold	:	1.6
Score for cons. seed	:	-0.6
Total read count	:	3296
Mature read count	:	2311
Loop read count	:	0
Star read count	:	985

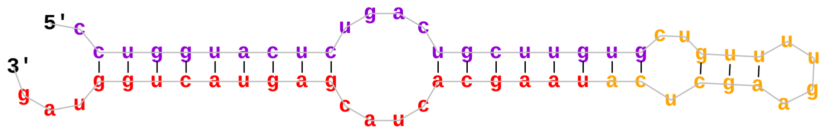

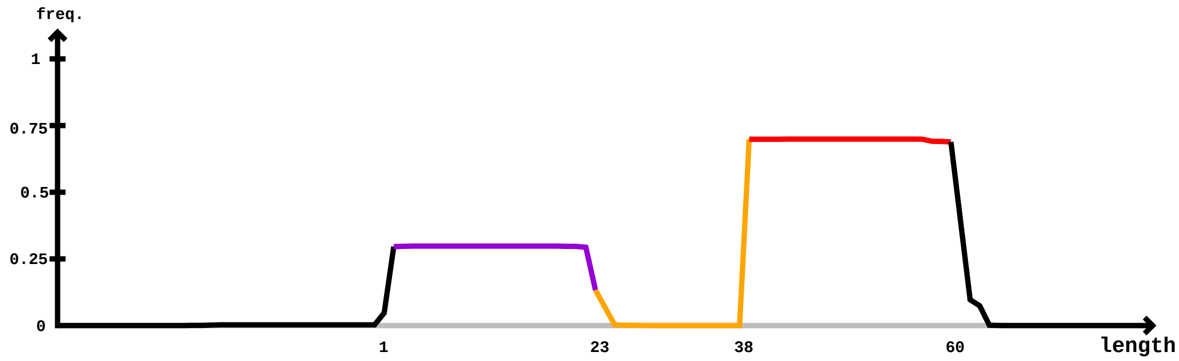

Star Mature

```
5'- uggugguauuugacgcuguucggucuugccuuuccugguacucugacugcuugugcuguuuugaagcucauaagcacuacgaguacugguagagacagggucuaaaacucuu  -3'  obs
    uggugguauuugacgcuguucggucuugccuuuccugguacucugacugcuugugcuguuuugaagcucauaagcacuacgaguacugguagagacagggucuaaaacucuu       exp
    .((((........))))....(((((((.((((.(((((((((....((((((((..(((....))).))))))))....))))))))).)))).)))))))..........  reads  mm  sample
    ............acgcuguucggucuugccuuu...............................................................................  2  0  seq
    ..............gcuguucggucuugccuuu...............................................................................  3  0  seq
    ..............gcuguuGggucuugccuuu...............................................................................  1  1  seq
    ...............cuguucggucuugccuuu...............................................................................  3  0  seq
    ................................uccugguacucugacugcu.............................................................  1  0  seq
    ................................uccugguacucugacugcuug...........................................................  5  0  seq
    ................................uccugguacucugacugcuugA..........................................................  1  1  seq
    ................................uccugguacucugacugcuugu..........................................................  140  0  seq
    .................................ccugguacucugacugcu.............................................................  2  0  seq
    .................................ccugguacucugacugcuu............................................................  1  0  seq
    .................................ccugguacucugacugcuug...........................................................  6  0  seq
    .................................ccugguacucugacugcuugu..........................................................  389  0  seq
    .................................ccugguacucCgacugcuugu..........................................................  1  1  seq
    .................................ccugguacucugacugcuCgug.........................................................  1  1  seq
    .................................ccugguacucCgacugcuugug.........................................................  5  1  seq
    .................................ccuAguacucugacugcuugug.........................................................  2  1  seq
    .................................ccugguacucugacugcuGgug.........................................................  1  1  seq
    .................................ccugguacucugacugcuugug.........................................................  383  0  seq
    .................................ccuggAacucugacugcuugug.........................................................  1  1  seq
    .................................ccugguacucugacugcuugugG........................................................  3  1  seq
    .................................ccugguacucugacugcuugugA........................................................  26  1  seq
    .................................ccugguacucugacugcuugugc........................................................  4  0  seq
    .................................ccugguacucugacugcuugugcu.......................................................  3  0  seq
    .................................ccugguacucugacugcuugugcug......................................................  1  0  seq
    .................................ccugguacucugacugcuugugcugu.....................................................  2  0  seq
    .................................ccugguacucugacugcuugugcuguu....................................................  1  0  seq
    ..................................cugguacucugacugcuugu..........................................................  1  0  seq
    ..................................cugguacucugacugcuugug.........................................................  1  0  seq
    ..................................cugguacucugacugcuugugA........................................................  1  1  seq
    ...................................ugguacucugacugcuugug.........................................................  1  0  seq
    ...................................ugguacucugacugcuugugcu.......................................................  2  0  seq
    ......................................................................uaagcacuacgaguacug........................  1  0  seq
    ......................................................................uaagcaAuacgaguacugg.......................  1  1  seq
```

```
                                  Star                                Mature
uggugguauuugacgcuguucggucuugccuuuccugguacucugacugcuugugcuguuuugaagcucauaagcacuacgaguacugguagagacagggucuaaaacucuu
......................................................................uaagcacuacgaguacugg.......................    25     0     seq
......................................................................uaagcacuacgaguacuggu......................    2      0     seq
......................................................................uaagcacuacgaguacuAgua.....................    2      1     seq
......................................................................uaagcacuacgaguacuggua.....................    5      0     seq
......................................................................uaagcacuacgaguacugguaA....................    3      1     seq
......................................................................uaagcacuacgaguaAugguag....................    1      1     seq
......................................................................uaagcaGuacgaguacugguag....................    3      1     seq
......................................................................uaagcacuacgaguacugguGg....................    8      1     seq
......................................................................uaagcacuacgaguacugguag....................    1735   0     seq
......................................................................uaagcaAuacgaguacugguag....................    30     1     seq
......................................................................uaagcacuacgaguacuAguag....................    2      1     seq
......................................................................uaagcacuacAaguacugguag....................    5      1     seq
......................................................................Aaagcacuacgaguacugguag....................    5      1     seq
......................................................................uaagcacuacgaguacugguaC....................    1      1     seq
......................................................................uaagAacuacgaguacugguag....................    1      1     seq
......................................................................uaagcacCacgaguacugguag....................    1      1     seq
......................................................................uaagcacuaGgaguacugguag....................    2      1     seq
......................................................................Aaagcacuacgaguacugguaga...................    1      1     seq
......................................................................uaagcacuacgaguacugguagU...................    5      1     seq
......................................................................uaagcacuacgaguacugAuaga...................    1      1     seq
......................................................................uaagcaGuacgaguacugguaga...................    1      1     seq
......................................................................uaagcacuacAaguacugguaga...................    1      1     seq
......................................................................uaagcacuacgaguacugguaga...................    145    0     seq
......................................................................uaagcaAuacgaguacugguaga...................    2      1     seq
......................................................................uaagcacuacgaguacugguagaA..................    72     1     seq
......................................................................uaagcacuacgaguacugguagaC..................    3      1     seq
......................................................................uaagcacuacgaguacugguagaAa.................    231    1     seq
......................................................................uaagcaUuacgaguacugguagaga.................    8      1     seq
......................................................................uaagcacuacgaguacugguagaCa.................    2      1     seq
......................................................................uaagcacuacgaguacugguagaAac................    3      1     seq
......................................................................uaagcacuacgaguacugguagaAaca...............    1      1     seq
..........................................................................cacuacgaguacugguag....................    2      0     seq
```
