# Supplementary material for: The Small RNA Universe of Capitella teleta
Source: Front Mol Biosci. 2022 Feb 25;9:802814. doi: 10.3389/fmolb.2022.802814 (PMC8915122; doi:10.3389/fmolb.2022.802814)
Supplement: Supplementary file 1 [file DataSheet1.ZIP › Supplement/confident/CAPTEscaffold_80_7449.pdf]

Provisional ID	:	CAPTEscaffold_80_7449
Score total	:	383.9
Score for star read(s)	:	3.9
Score for read counts	:	376.4
Score for mfe	:	2.6
Score for randfold	:	1.6
Score for cons. seed	:	-0.6
Total read count	:	750
Mature read count	:	693
Loop read count	:	0
Star read count	:	57

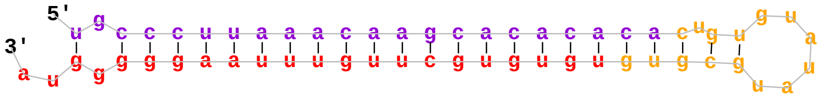

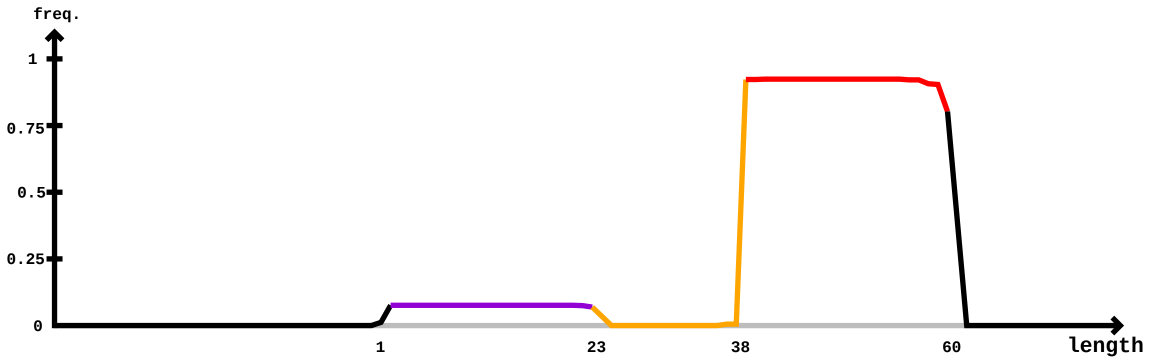

Star Mature

```
5'- gcaguuucgucauaccccaccgugagggggugaugcccuuaaacaagcacacacacuguguauaugcgugugugugcuuguuuaagggggguaacccccucacugaaacc -3'  obs
    gcaguuucgucauaccccaccgugagggggugaugcccuuaaacaagcacacacacuguguauaugcgugugugugcuuguuuaagggggguaacccccucacugaaacc       exp
    ...((((((............((((((((((.((.(((((((((((((((((((((.((......))))))))))))))))))))))))).)).)))))))))))))))).   reads  mm  sample
    ................................augcccuuaaacaagcacaca........................................................   1      0   seq
    ................................augcccuuaaacaagcacacac.......................................................   1      0   seq
    ................................augcccAuaaacaagcacacaca......................................................   1      1   seq
    ................................augcccuuaaacaagcacacaca......................................................   6      0   seq
    .................................ugcccuuaaacaagcGcacac.......................................................   2      1   seq
    .................................ugcccuuaaacaagcacacac.......................................................   1      0   seq
    .................................ugcccuuaaacaGgcacacaca......................................................   1      1   seq
    .................................ugcccuuaaacaagcacacaca......................................................   44     0   seq
    ....................................................................ugugugugcuuguuuaagg......................   2      0   seq
    ....................................................................ugugugugcuuguuuaagggg....................   2      0   seq
    ......................................................................ugugugcuuguuuaagggg....................   9      0   seq
    ......................................................................ugugugcuuguuuaaggggg...................   2      0   seq
    ......................................................................ugugugcuuguuuaagggggu..................   74     0   seq
    ......................................................................Agugugcuuguuuaagggggu..................   1      1   seq
    ......................................................................ugugugcuuguuuaagggggG..................   1      1   seq
    ......................................................................ugugugcuuguuuaaggggguU.................   1      1   seq
    ......................................................................ugugugcuuguuuaaggggguC.................   519    1   seq
    ......................................................................ugugugcuuguuuaagggggua.................   72     0   seq
    ......................................................................ugugugcuuguuuaaggggguCa................   9      1   seq
    ........................................................................ugugcuuguuuaaggggguCa................   1      1   seq
```
