# Supplementary material for: The Small RNA Universe of Capitella teleta
Source: Front Mol Biosci. 2022 Feb 25;9:802814. doi: 10.3389/fmolb.2022.802814 (PMC8915122; doi:10.3389/fmolb.2022.802814)
Supplement: Supplementary file 1 [file DataSheet1.ZIP › Supplement/confident/CAPTEscaffold_324_18313.pdf]

Provisional ID	:	CAPTEscaffold_324_18313
Score total	:	379.1
Score for star read(s)	:	3.9
Score for read counts	:	373.3
Score for mfe	:	0.9
Score for randfold	:	1.6
Score for cons. seed	:	-0.6
Total read count	:	744
Mature read count	:	534
Loop read count	:	0
Star read count	:	210

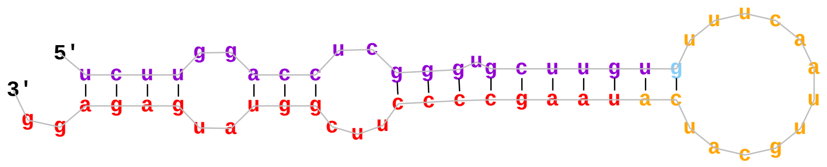

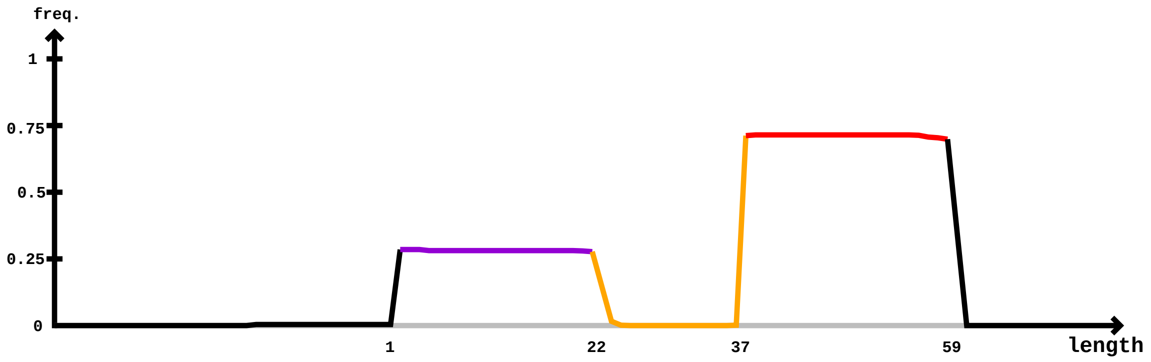


```
                                  Star                                Mature
5'- gaaucaagaauuauguuggguaugauuggcucuuucuuggaccucgggugcuuguguuucaauugcaucauaagccccuucgguaugagagggaacugaugcucagucu  -3'   obs
    gaaucaagaauuauguuggguaugauuggcucuuucuuggaccucgggugcuuguguuucaauugcaucauaagccccuucgguaugagagggaacugaugcucagucu         exp
    ...............((((((...(((((.((((((((..(((..(((.(((((((............))))))))))...)))..)))))))).)))))))))))...    reads    mm    sample
    ...................guaugauuggcucuuucu........................................................................    2        0     seq
    ...................gCaugauuggcucuuucu........................................................................    1        1     seq
    ..................................ucuuggaccucgggugcuu........................................................    1        0     seq
    ..................................ucuuggaccucgggugcuug.......................................................    2        0     seq
    ..................................ucuuggaccucgggugcuugu......................................................    145      0     seq
    ..................................ucuuAgaccucgggugcuugu......................................................    2        1     seq
    ..................................ucuuggaccucgggugcuugA......................................................    2        1     seq
    ..................................ucuuggaGcucgggugcuugug.....................................................    1        1     seq
    ..................................ucuuggaccucgggugcuuguA.....................................................    2        1     seq
    ..................................ucuuggaccucgggugcuugug.....................................................    41       0     seq
    ..................................ucuuggaccucgggugcuuguU.....................................................    1        1     seq
    ..................................Acuuggaccucgggugcuugug.....................................................    1        1     seq
    ..................................ucuuggaccucgggugcuugugA....................................................    7        1     seq
    ..................................ucuuggaccucgggugcuugugG....................................................    2        1     seq
    ..................................ucuuggaccucgggugcuugugu....................................................    2        0     seq
    ..................................ucuuggaccucgggugcuugugAu...................................................    1        1     seq
    .....................................................................Cuaagccccuucgguaugagagg.................    1        1     seq
    ......................................................................uaagccccuucgguauga.....................    1        0     seq
    ......................................................................uaagccccuucgguaugag....................    5        0     seq
    ......................................................................uaagccccuucgguaugaga...................    1        0     seq
    ......................................................................uaagccccuucgguaugagG...................    1        1     seq
    ......................................................................uaagccccuucgguaugagag..................    4        0     seq
    ......................................................................uaagccccuucgguaugagUgg.................    1        1     seq
    ......................................................................uaagccccuucgguaugagagg.................    489      0     seq
    ......................................................................Naagccccuucgguaugagagg.................    1        1     seq
    ......................................................................uaagccccuucgguGugagagg.................    1        1     seq
    ......................................................................uaagccccuucgguaugagagC.................    18       1     seq
    ......................................................................uaagcccUuucgguaugagagg.................    2        1     seq
    ......................................................................uaagccUcuucgguaugagagg.................    2        1     seq
    ......................................................................uaagccccuAcgguaugagagg.................    1        1     seq
    ......................................................................uaagccccuuAgguaugagagg.................    1        1     seq
    ......................................................................Aaagccccuucgguaugagagg.................    2        1     seq
    ......................................................................uaagccccuuGgguaugagagg.................    1        1     seq
```

Star **Mature**

```
gaaucaagaauuauguuggguaugauuggcucuuucuuggaccucgggugcuuguguuucaauugcaucauaagccccuucgguaugagagggaacugaugcucagucu
.......................................................................aagccccuucgguaugagagg.................   2   0   seq
```
