# Supplementary material for: The Small RNA Universe of Capitella teleta
Source: Front Mol Biosci. 2022 Feb 25;9:802814. doi: 10.3389/fmolb.2022.802814 (PMC8915122; doi:10.3389/fmolb.2022.802814)
Supplement: Supplementary file 1 [file DataSheet1.ZIP › Supplement/confident/CAPTEscaffold_324_18317.pdf]

Provisional ID	:	CAPTEscaffold_324_18317
Score total	:	459.5
Score for star read(s)	:	3.9
Score for read counts	:	455.9
Score for mfe	:	-1.2
Score for randfold	:	1.6
Score for cons. seed	:	-0.6
Total read count	:	906
Mature read count	:	685
Loop read count	:	0
Star read count	:	221

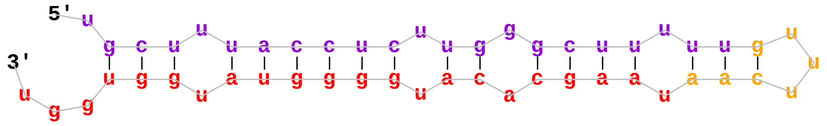

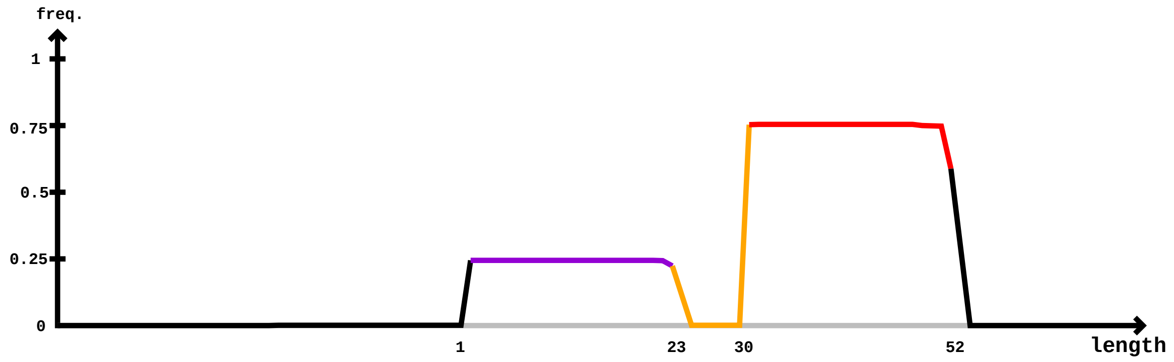

Star **Mature**

```
5'- cuuuuuaauuaagaugaccaacaauucugcuuggcuuguguugcuuuaccucuugggcuuuuuguucaauaagcacaugggguaugguggugugugcccaagucuuugca   -3'   obs
    cuuuuuaauuaagaugaccaacaauucugcuuggcuuguguugcuuuaccucuugggcuuuuuguucaauaagcacaugggguaugguggugugugcccaagucuuugca         exp
    ..........................(((..((((((.((((((.((((((.((.((((.(((...))).)))).)).)))))).))))......)).))))))...)))   reads   mm   sample
    .....................caauucugcuuggcuugugu.....................................................................   1       0    seq
    .........................................ugcuuuaccucuugggcuuu.................................................   1       0    seq
    .........................................ugcuuuaccucuugggcuuuu................................................   18      0    seq
    .........................................ugcuuuaccucuugggUuuuuu...............................................   2       1    seq
    .........................................ugcuuuaccucuugggcuuuuu...............................................   150     0    seq
    .........................................ugcuuuaccucuugggcuGuuu...............................................   12      1    seq
    .........................................ugcuuAaccucuugggcuuuuu...............................................   1       1    seq
    .........................................ugcuuuaccucuuAggcuuuuu...............................................   2       1    seq
    .........................................ugcuuuaccucuugggcuuuuuU..............................................   30      1    seq
    .........................................ugcuuuaccucuugggcuuuuuA..............................................   5       1    seq
    .........................................ugcuuuaccucuugggcuuuuuguucaau........................................   1       0    seq
    .....................................................................uaagcacaugggguaugg.......................   3       0    seq
    .....................................................................uaagcacGugggguaugg.......................   1       1    seq
    .....................................................................uaagcacGugggguauggu......................   1       1    seq
    .....................................................................uaagcacaugggguauggug.....................   1       0    seq
    .....................................................................uaagcacauAggguauggugg....................   1       1    seq
    .....................................................................uaagcacaugggguGuggugg....................   3       1    seq
    .....................................................................uaagcacGugggguauggugg....................   6       1    seq
    .....................................................................uaagcacaugggguauggugg....................   135     0    seq
    .....................................................................uaagcacaugggguaugguAgu...................   1       1    seq
    .....................................................................Aaagcacaugggguaugguggu...................   1       1    seq
    .....................................................................uaagcacauAggguaugguggu...................   1       1    seq
    .....................................................................uaagcacaugggguGugguggu...................   98      1    seq
    .....................................................................uaagcacaugggguaugguggu...................   265     0    seq
    .....................................................................uUagcacaugggguaugguggu...................   1       1    seq
    .....................................................................Gaagcacaugggguaugguggu...................   2       1    seq
    .....................................................................uaagcacGugggguaugguggu...................   131     1    seq
    .....................................................................uaagcacaugggguauggugguA..................   31      1    seq
    .....................................................................uaagcacaugggguauggugguU..................   1       1    seq
    ......................................................................aagcacaugggguauggugguA..................   2       1    seq
```
