# Supplementary material for: The Small RNA Universe of Capitella teleta
Source: Front Mol Biosci. 2022 Feb 25;9:802814. doi: 10.3389/fmolb.2022.802814 (PMC8915122; doi:10.3389/fmolb.2022.802814)
Supplement: Supplementary file 1 [file DataSheet1.ZIP › Supplement/confident/CAPTEscaffold_154_12132.pdf]

Provisional ID	:	CAPTEscaffold_154_12132
Score total	:	181.8
Score for star read(s)	:	3.9
Score for read counts	:	175.5
Score for mfe	:	1.4
Score for randfold	:	1.6
Score for cons. seed	:	-0.6
Total read count	:	356
Mature read count	:	184
Loop read count	:	0
Star read count	:	172

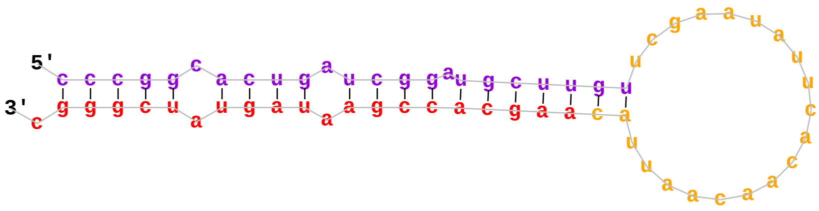

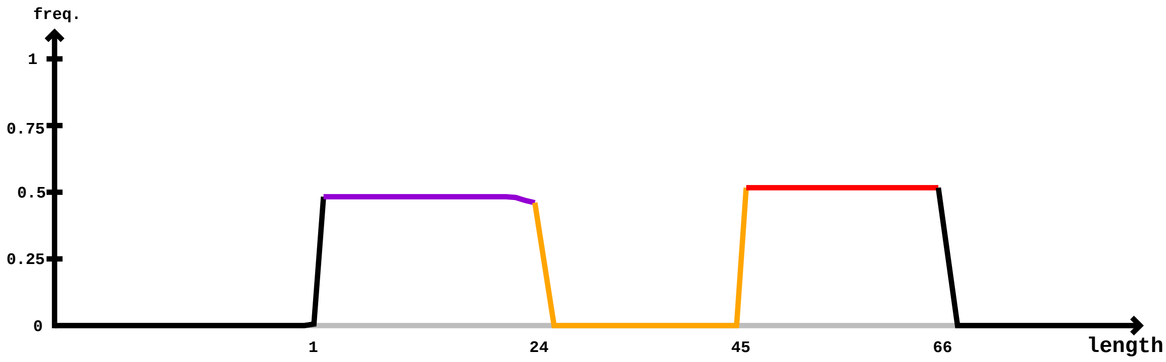

	Star / Mature				
5'-	cuggaaaggacaucgguucguacauucccggcacugaucggaugcuuguucgaauauucacaacaauuacaagcaccgaauaguaucgggcaugguggaaugccacgaucg	-3'	obs		
	cuggaaaggacaucgguucguacauucccggcacugaucggaugcuuguucgaauauucacaacaauuacaagcaccgaauaguaucgggcaugguggaaugccacgaucg		exp		
	.......((((....))))...(((.(((((.((((.((((.(((((((...................))))))))))).)))).))))).)))((((....)))).....	reads		mm	sample
	........................ucccggcacugaucggaugcuu...	1		0	seq
	........................ucccggcacugaucggaugcuugu...	1		0	seq
	..........................cccggcacugaucggaugcu...	1		0	seq
	..........................cccggcacugaucggaugcuu..	2		0	seq
	..........................cccggcacuAaucggaugcuu..	1		1	seq
	..........................cccggcacugaucggaugcuuU...	1		1	seq
	..........................cccggcacugaucggaugcuug...	2		0	seq
	..........................ccAggcacugaucggaugcuugu..	1		1	seq
	..........................cccggcacugaucggaugcuugu..	158		0	seq
	..........................cccAgcacugaucggaugcuugu..	1		1	seq
	..........................cccggcacugaucAgaugcuugu..	2		1	seq
	..........................cccggcacugaucggaugcuuguu...	1		0	seq
	....aagcaccAaauaguaucgggc....................	5		1	seq
	....aagcaccgaauaguaucgggc....................	170		0	seq
	....aagcaccgaauaguauUgggc....................	2		1	seq
	....aagcaccgaauaguaucgggca...................	6		0	seq
	....aagcaccgaauaguaucgggcU...................	1		1	seq
