# Supplementary material for: The Small RNA Universe of Capitella teleta
Source: Front Mol Biosci. 2022 Feb 25;9:802814. doi: 10.3389/fmolb.2022.802814 (PMC8915122; doi:10.3389/fmolb.2022.802814)
Supplement: Supplementary file 1 [file DataSheet1.ZIP › Supplement/confident/CAPTEscaffold_60_5457.pdf]

Provisional ID : CAPTEscaffold_60_5457
Score total : 809.5
Score for star read(s) : 3.9
Score for read counts : 803.6
Score for mfe : 1
Score for randfold : 1.6
Score for cons. seed : -0.6
Total read count : 1588
Mature read count : 1378
Loop read count : 0
Star read count : 210

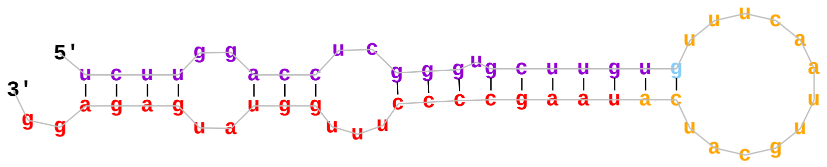

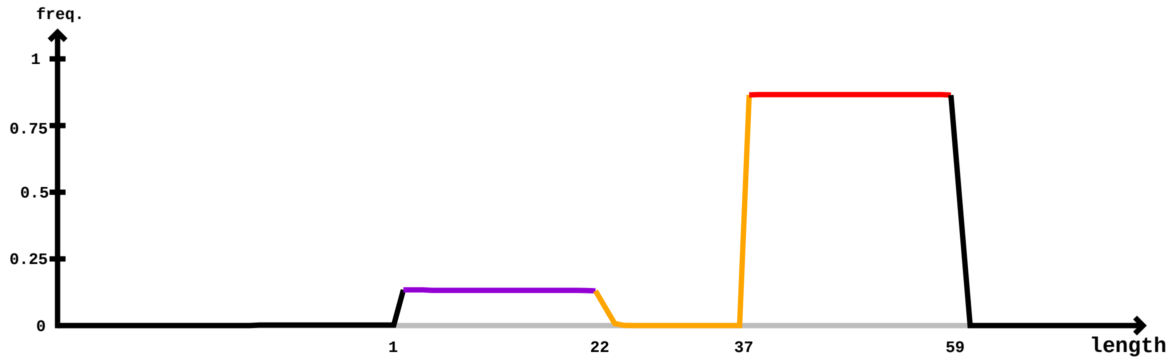

Star Mature

```
5'- gcaucaagaauuauguuuaguaugauuggcucuuucuuggaccucgggugcuuguguuucaauugcaucauaagccccuuugguaugagagggaacugaugcucagucugc -3'  obs
    gcaucaagaauuauguuuaguaugauuggcucuuucuuggaccucgggugcuuguguuucaauugcaucauaagccccuuugguaugagagggaacugaugcucagucugc       exp
    ((((((..(((((((.....)))))))...((((((((..(((..(((.(((((((...........))))))))))...)))..))))))))..))))))..........   reads  mm  sample
    ...................gCaugauuggcucuuucu..........................................................................   1      1   seq
    ...................guaugauuggcucuuucu..........................................................................   2      0   seq
    ..................................ucuuggaccucgggugcuu..........................................................   1      0   seq
    ..................................ucuuggaccucgggugcuug.........................................................   2      0   seq
    ..................................ucuuggaccucgggugcuugA........................................................   2      1   seq
    ..................................ucuuggaccucgggugcuugu........................................................   145    0   seq
    ..................................ucuuAgaccucgggugcuugu........................................................   2      1   seq
    ..................................ucuuggaGcucgggugcuugug.......................................................   1      1   seq
    ..................................ucuuggaccucgggugcuugug.......................................................   41     0   seq
    ..................................Acuuggaccucgggugcuugug.......................................................   1      1   seq
    ..................................ucuuggaccucgggugcuuguU.......................................................   1      1   seq
    ..................................ucuuggaccucgggugcuuguA.......................................................   2      1   seq
    ..................................ucuuggaccucgggugcuugugG......................................................   2      1   seq
    ..................................ucuuggaccucgggugcuugugA......................................................   7      1   seq
    ..................................ucuuggaccucgggugcuugugu......................................................   2      0   seq
    ..................................ucuuggaccucgggugcuugugAu.....................................................   1      1   seq
    ......................................................................uaagccccuuugguaugagag....................   3      0   seq
    ......................................................................uaagccccuuuggGaugagagg...................   1      1   seq
    ......................................................................uaagccccuuugguaugagagA...................   1      1   seq
    ......................................................................uaagcccCGuugguaugagagg...................   1      1   seq
    ......................................................................uGagccccuuugguaugagagg...................   1      1   seq
    ......................................................................uaagccAcuuugguaugagagg...................   3      1   seq
    ......................................................................uaagccccuuugguaugagagg...................   1321   0   seq
    ......................................................................uaagccccuuuAguaugagagg...................   7      1   seq
    ......................................................................Gaagccccuuugguaugagagg...................   1      1   seq
    ......................................................................uaagccccuuugguaCgagagg...................   1      1   seq
    ......................................................................uaagccccuuugguaugagagU...................   2      1   seq
    ......................................................................uaagccccuuugguaugagagC...................   2      1   seq
    ......................................................................uaagccccuuugguaugaUagg...................   1      1   seq
    ......................................................................uaagccccuuugguaugagUgg...................   1      1   seq
    ......................................................................Aaagccccuuugguaugagagg...................   7      1   seq
    ......................................................................uaagcccUuuugguaugagagg...................   2      1   seq
    ......................................................................uaagccccuuugguaugagaAg...................   13     1   seq
```

```
                                  Star                                Mature
gcaucaagaauuauguuuaguaugauuggcucuuucuuggaccucgggugcuuguguuucaauugcaucauaagccccuuugguaugagagggaacugaugcucagucugc
......................................................................uaagccccuuugguauAagagg...................    3    1    seq
......................................................................uaagccccuuGgguaugagagg...................    1    1    seq
......................................................................uaagccccuuAgguaugagagg...................    1    1    seq
......................................................................uaagccccuAugguaugagagg...................    1    1    seq
......................................................................uaagccccuuugguaugagaggA..................    2    1    seq
.......................................................................aagccccuuugguaugagagg...................    1    0    seq
.......................................................................aagccccGuugguaugagagg...................    1    1    seq
```
