# Supplementary material for: The Small RNA Universe of Capitella teleta
Source: Front Mol Biosci. 2022 Feb 25;9:802814. doi: 10.3389/fmolb.2022.802814 (PMC8915122; doi:10.3389/fmolb.2022.802814)
Supplement: Supplementary file 1 [file DataSheet1.ZIP › Supplement/confident/CAPTEscaffold_15480_45260.pdf]

Provisional ID	:	CAPTEscaffold_15480_45260
Score total	:	30413.7
Score for star read(s)	:	3.9
Score for read counts	:	30407.1
Score for mfe	:	1.7
Score for randfold	:	1.6
Score for cons. seed	:	-0.6
Total read count	:	59654
Mature read count	:	55805
Loop read count	:	0
Star read count	:	3849

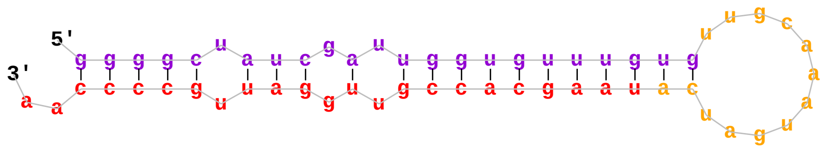

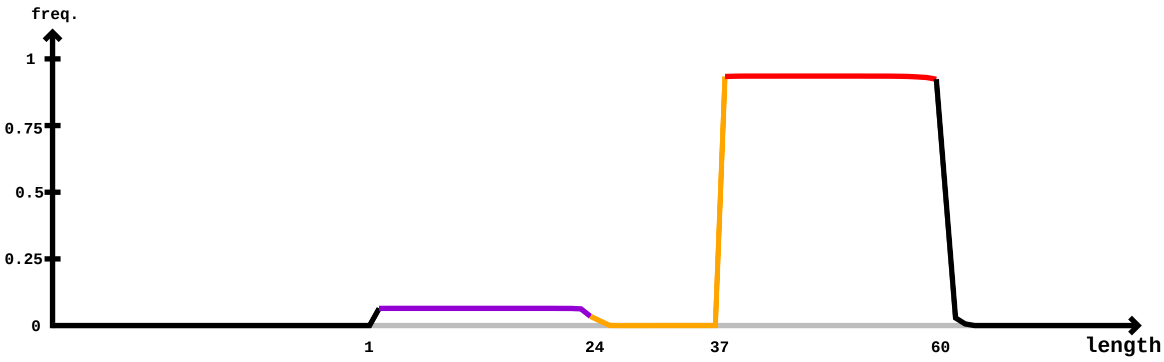

Star **Mature**

```
5'- uccuaauuuucggagauguucaucucaguuucggggcuaucgauugguguuuguguugcaaaugaucauaagcaccguuggauugccccaaaauuggaauuugcuaucauu  -3'  obs
    uccuaauuuucggagauguucaucucaguuucggggcuaucgauugguguuuguguugcaaaugaucauaagcaccguuggauugccccaaaauuggaauuugcuaucauu       exp
    (((........)))(((((...((.((((((.(((((.(((.(.((((((((((.............)))))))))).).))).))))).))))))))....)).)))...   reads   mm   sample
    ...............................cggggcuaucgauugguguuugug........................................................   2       0    seq
    ................................ggggcuaucgauuggugu.............................................................   2       0    seq
    ................................ggggcuaucgauugguguu............................................................   13      0    seq
    ................................ggggcuaucgauugguguuu...........................................................   3       0    seq
    ................................ggggcuaucgauAgguguuug..........................................................   1       1    seq
    ................................ggggcuaucgauugguguuug..........................................................   89      0    seq
    ................................ggggcuaucgauugguguuuA..........................................................   1       1    seq
    ................................Agggcuaucgauugguguuug..........................................................   1       1    seq
    ................................ggggcuaucgauuggugAuugu.........................................................   2       1    seq
    ................................ggggcuauAgauugguguuugu.........................................................   1       1    seq
    ................................ggggcuaucgauugguguuCgu.........................................................   1       1    seq
    ................................ggggcuaucgauuAguguuugu.........................................................   5       1    seq
    ................................ggggGuaucgauugguguuugu.........................................................   1       1    seq
    ................................Ngggcuaucgauugguguuugu.........................................................   1       1    seq
    ................................ggggcuaucgauugguguuugA.........................................................   1       1    seq
    ................................ggggcuaucgauugguguuAgu.........................................................   1       1    seq
    ................................ggggcuaucgauugguguuuCu.........................................................   1       1    seq
    ................................ggggcuaucgauugguguuugG.........................................................   1       1    seq
    ................................ggggcuauUgauugguguuugu.........................................................   4       1    seq
    ................................ggggcuaucgauugguguuugu.........................................................   1645    0    seq
    ................................ggggcuaucAauugguguuugu.........................................................   5       1    seq
    ................................ggggcuaucgauuggAguuugu.........................................................   1       1    seq
    ................................Agggcuaucgauugguguuugu.........................................................   8       1    seq
    ................................ggggcuaucgauAgguguuugu.........................................................   1       1    seq
    ................................ggggcuaucgauugguguuCgug........................................................   1       1    seq
    ................................ggAgcuaucgauugguguuugug........................................................   2       1    seq
    ................................ggggcuauUgauugguguuugug........................................................   6       1    seq
    ................................Ugggcuaucgauugguguuugug........................................................   2       1    seq
    ................................ggggcuaucgauugguguuAgug........................................................   1       1    seq
    ................................ggggcuaucgauugguguuugug........................................................   1944    0    seq
    ................................ggggcuaucgauugguguuuAug........................................................   1       1    seq
    ................................ggCgcuaucgauugguguuugug........................................................   1       1    seq
    ................................ggggcuaucgaAugguguuugug........................................................   1       1    seq
```

```
                                Star                                    Mature
uccuaauuuucggagauguucaucucaguuucggggcuaucgauugguguuuguguugcaaaugaucauaagcaccguuggauugccccaaaauuggaauuugcuaucauu
................................ggggcuaucgauugUuguuugug........................................................ 1	1	seq
................................ggggcuaucgauuAguguuugug........................................................ 6	1	seq
................................ggggcuaucgauugguAuuugug........................................................ 1	1	seq
................................ggggcuaucgauugguguAugug........................................................ 1	1	seq
................................ggggcuaucCauugguguuugug........................................................ 1	1	seq
................................ggggcuauAgauugguguuugug........................................................ 1	1	seq
................................ggggcuaucgauugguguuuguA........................................................ 4	1	seq
................................Agggcuaucgauugguguuugug........................................................ 14	1	seq
................................ggggcuaucgauugguguuuguU........................................................ 4	1	seq
................................ggggcuaucgauuggAguuugug........................................................ 3	1	seq
................................ggggcuaucgauugguguCugug........................................................ 1	1	seq
................................ggggcuaucAauugguguuugug........................................................ 10	1	seq
................................gAggcuaucgauugguguuugug........................................................ 5	1	seq
................................ggggcuaucgauugguguuugugG....................................................... 2	1	seq
................................ggggcuaucgauugguguuugugA....................................................... 6	1	seq
................................ggggcuaucgauugguguuugugC....................................................... 1	1	seq
................................ggggcuaucgauugguguuugugu....................................................... 17	0	seq
................................ggggcuaucgauugguguuuguguA...................................................... 8	1	seq
................................ggggcuaucgauugguguuuguguu...................................................... 8	0	seq
................................ggggcuaucgauugguguuuguguugc.................................................... 1	0	seq
.................................gggcuaucgauuAguguuugug........................................................ 1	1	seq
.................................gggcuaucgauugguguuugug........................................................ 1	0	seq
..................................ggcuaucgauugguguuugu......................................................... 1	0	seq
.....................................uaucgauugguguuugug........................................................ 1	0	seq
.............................................................augaucauaagcaccguuggau............................ 5	0	seq
..............................................................ugaucauaagcaccguuggau............................ 5	0	seq
...............................................................gaucauaagcaccguuggauugccccaa.................... 2	0	seq
.................................................................ucauaagcaccguuggauugccA....................... 1	1	seq
....................................................................uaagcaccguuggauugc......................... 32	0	seq
....................................................................uaagcaccguuggauugcc........................ 38	0	seq
....................................................................uaagcaccguuAgauugccc....................... 1	1	seq
....................................................................uaagcaccguuggauugccc....................... 93	0	seq
....................................................................Aaagcaccguuggauugccc....................... 2	1	seq
....................................................................uaagcaccguuggauugccA....................... 2	1	seq
....................................................................Aaagcaccguuggauugcccc...................... 2	1	seq
....................................................................uaagcaccguuggauugccAc...................... 2	1	seq
....................................................................uaagcaccguuAgauugcccc...................... 2	1	seq
....................................................................uaagcaccguuggauugcccA...................... 1	1	seq
....................................................................uaagcaccguuggauugcccc...................... 131	0	seq
....................................................................uaagcaccguuggauugcccGa..................... 78	1	seq
....................................................................uaagcaccguuggauugcccAa..................... 26	1	seq
....................................................................uaagcaccguuggauugccccC..................... 3	1	seq
....................................................................uaagcaccguuggauugcccca..................... 266	0	seq
....................................................................Gaagcaccguuggauugcccca..................... 1	1	seq
....................................................................uaagcaccguuggauugccccU..................... 6	1	seq
....................................................................uaagcaccguuAgauugcccca..................... 2	1	seq
....................................................................uaagcaccguuggauAgccccaa.................... 39	1	seq
....................................................................uaagcacAguuggauugccccaa.................... 25	1	seq
....................................................................uaagcaccguuggUuugccccaa.................... 2	1	seq
....................................................................uaagcacUguuggauugccccaa.................... 25	1	seq
....................................................................uGagcaccguuggauugccccaa.................... 32	1	seq
....................................................................uaaAcaccguuggauugccccaa.................... 4	1	seq
....................................................................uaagcaccguCggauugccccaa.................... 6	1	seq
....................................................................uNagcaccguuggauugccccaa.................... 3	1	seq
....................................................................uaagcaccguuggauugGcccaa.................... 3	1	seq
....................................................................uaagcaccguGggauugccccaa.................... 1	1	seq
....................................................................uaaCcaccguuggauugccccaa.................... 3	1	seq
....................................................................Gaagcaccguuggauugccccaa.................... 19	1	seq
....................................................................uaagcaccguuggGuugccccaa.................... 7	1	seq
....................................................................uaagcaccguuggCuugccccaa.................... 1	1	seq
....................................................................uaagcaccguuggaGugccccaa.................... 1	1	seq
....................................................................uaagcaccguuggauugccccaa.................... 46608	0	seq
....................................................................uaagcaccgCuggauugccccaa.................... 5	1	seq
....................................................................uaagcaccguuggauuUccccaa.................... 4	1	seq
....................................................................uaagcCccguuggauugccccaa.................... 1	1	seq
....................................................................uaagcaccUuuggauugccccaa.................... 1	1	seq
....................................................................uaGgcaccguuggauugccccaa.................... 8	1	seq
....................................................................uaagcaccguugAauugccccaa.................... 13	1	seq
....................................................................uaagcaAcguuggauugccccaa.................... 15	1	seq
....................................................................uaagcaccguuggauugccUcaa.................... 39	1	seq
```

Star **Mature**

uccuaauuuucggagauguucaucucaguuucggggcuaucgauugguguuuguguugcaaaugaucauaagcaccguuggauugccccaaaauuggaauuugcuaucauu			
....uaagcaccguuCgauugccccaa....................	3	1	seq
....uaagcaccguuUgauugccccaa....................	4	1	seq
....uaagcaccguuggauugccccaG....................	12	1	seq
....uaagcaccguuggaAugccccaa....................	19	1	seq
....uaagcaccguAggauugccccaa....................	25	1	seq
....uaagcaUcguuggauugccccaa....................	2	1	seq
....uaagGaccguuggauugccccaa....................	1	1	seq
....uaagcaccAuuggauugccccaa....................	56	1	seq
....uaagcaccgAuggauugccccaa....................	29	1	seq
....Aaagcaccguuggauugccccaa....................	196	1	seq
....uaagcaccguuggauugccGcaa....................	2	1	seq
....uCagcaccguuggauugccccaa....................	1	1	seq
....Caagcaccguuggauugccccaa....................	5	1	seq
....uaagcaccguuAgauugccccaa....................	221	1	seq
....uaagcaccguuggauugcUccaa....................	16	1	seq
....uaagcaGcguuggauugccccaa....................	4	1	seq
....uaagcaccguuggauugccccaC....................	65	1	seq
....uaagcaccguuggauugcAccaa....................	19	1	seq
....uaagcGccguuggauugccccaa....................	7	1	seq
....uaagcaccguuggauCgccccaa....................	12	1	seq
....uaagcaccguuggauugAcccaa....................	10	1	seq
....uaUgcaccguuggauugccccaa....................	2	1	seq
....uaagcaccguuggauugcccGaa....................	8	1	seq
....uaagcaccguuggauugccccaU....................	23	1	seq
....uaagAaccguuggauugccccaa....................	10	1	seq
....uaagcaccguuggauugcccUaa....................	68	1	seq
....uaagUaccguuggauugccccaa....................	5	1	seq
....uaagcaccguugUauugccccaa....................	3	1	seq
....uaagcaccguuggauugccccUa....................	35	1	seq
....uaagcaccguuggaCugccccaa....................	6	1	seq
....uaagcaccguuggauugccAcaa....................	96	1	seq
....uaagcaccguuggauugccccCa....................	58	1	seq
....Naagcaccguuggauugccccaa....................	16	1	seq
....uaaUcaccguuggauugccccaa....................	5	1	seq
....uaagcaccguuggauugcGccaa....................	1	1	seq
....uaagcaccguuggauugUcccaa....................	10	1	seq
....uaagcaccguugCauugccccaa....................	3	1	seq
....uaagcaccguuggauuAccccaa....................	7	1	seq
....uaagcaccguuggauuCccccaa....................	8	1	seq
....uaagcaccguuggauugcccAaa....................	14	1	seq
....uaagcUccguuggauugccccaa....................	8	1	seq
....uUagcaccguuggauugccccaa....................	1	1	seq
....uaagcaccguuggauGgccccaa....................	1	1	seq
....uaagcaccguuggauugcccUaaa...................	8	1	seq
....uaagcaccguuggauugAcccaaa...................	2	1	seq
....uaagcaccguuggauugUcccaaa...................	1	1	seq
....uaagcaccguuggauugcccAaaa...................	3	1	seq
....uaagcaccguuggauugcUccaaa...................	3	1	seq
....uaagcaccguuggaAugccccaaa...................	1	1	seq
....uaagcaccgAuggauugccccaaa...................	3	1	seq
....uaagcaccguuggauugccGcaaa...................	2	1	seq
....uaaUcaccguuggauugccccaaa...................	2	1	seq
....uaagcUccguuggauugccccaaa...................	1	1	seq
....uaagcaccguugUauugccccaaa...................	1	1	seq
....Naagcaccguuggauugccccaaa...................	1	1	seq
....uaagcaccguCggauugccccaaa...................	1	1	seq
....uaagcaccguuggauugccccaaa...................	5139	0	seq
....uaagcaccguuCgauugccccaaa...................	1	1	seq
....uaagcaccguuggaCugccccaaa...................	1	1	seq
....uaagcaccguuggauugccUcaaa...................	5	1	seq
....uaagcacAguuggauugccccaaa...................	1	1	seq
....uaagcaccguuggauugccccCaa...................	58	1	seq
....Gaagcaccguuggauugccccaaa...................	2	1	seq
....uaagcaccguGggauugccccaaa...................	1	1	seq
....uaagcacUguuggauugccccaaa...................	3	1	seq
....uaagcaccguuggauCgccccaaa...................	1	1	seq
....uaagcaccguuggauuUccccaaa...................	2	1	seq
....uaagcaccguuggauugccccaaU...................	21	1	seq
....uaagcaccguuggauugccccaCa...................	3	1	seq
....uaagcaccguuggUuugccccaaa...................	1	1	seq

Star **Mature**

```
uccuaauuuucggagauguucaucucaguuucggggcuaucgauugguguuugugugcaaaugaucauaagcaccguuggauugccccaaaauuggaauuugcuaucauu
....................................................................uaagcaccguuggauugccAcaaa..................   10     1   seq
....................................................................uaagcaccguuggauugccccaaC..................   7      1   seq
....................................................................uaagcaccguuggaGugccccaaa..................   1      1   seq
....................................................................uaagcaccguuggauugccccaGa..................   1      1   seq
....................................................................uaagcaccguugCauugccccaaa..................   1      1   seq
....................................................................uaagcaccguuggauugccccUaa..................   2      1   seq
....................................................................uaagcaccguuAgauugccccaaa..................   16     1   seq
....................................................................uaagUaccguuggauugccccaaa..................   3      1   seq
....................................................................uaagcaccguuggauugcccGaaa..................   3      1   seq
....................................................................uaagcaccguuggauAgccccaaa..................   7      1   seq
....................................................................Aaagcaccguuggauugccccaaa..................   22     1   seq
....................................................................uaagcaccgCuggauugccccaaa..................   2      1   seq
....................................................................uaagcaccguAggauugccccaaa..................   2      1   seq
....................................................................uaagcaccguuggauugccccaaG..................   6      1   seq
....................................................................uaagcaccguuggauugccccaaaa.................   1262   0   seq
....................................................................uaagcaccguuggauugccccaaUa.................   1      1   seq
....................................................................uaagcaccguuggauugccAcaaaa.................   3      1   seq
....................................................................uaagcaccguAggauugccccaaaa.................   2      1   seq
....................................................................uaagcaccgAuggauugccccaaaa.................   1      1   seq
....................................................................uaagcGccguuggauugccccaaaa.................   1      1   seq
....................................................................uaagcaccAuuggauugccccaaaa.................   3      1   seq
....................................................................uaagcaccguuggauugccccaaaU.................   5      1   seq
....................................................................uaagcaccguuAgauugccccaaaa.................   4      1   seq
....................................................................uaagcaccguuggauugccccaaGa.................   1      1   seq
....................................................................uaagcaccguuggauuCccccaaaa.................   1      1   seq
....................................................................uaagcaccguuggauugccccaaaG.................   9      1   seq
....................................................................Aaagcaccguuggauugccccaaaa.................   3      1   seq
....................................................................uaagcaccguuggauugcccUaaaa.................   2      1   seq
....................................................................uaagcaccguuggauugccGcaaaa.................   1      1   seq
....................................................................uaagcaccguuggauugcAccaaaa.................   1      1   seq
....................................................................uaagcaccguuggauugccccaaaC.................   12     1   seq
....................................................................uaagcaccguuggauugccccaCaa.................   2      1   seq
....................................................................uaagcaccguuggauugccUcaaaa.................   1      1   seq
....................................................................uaagcaccguCggauugccccaaaa.................   1      1   seq
....................................................................uaagcacUguuggauugccccaaaa.................   1      1   seq
....................................................................uaagcUccguuggauugccccaaaa.................   1      1   seq
....................................................................uaagcaccguuggauugccccUaaa.................   1      1   seq
....................................................................uaagcaccguuggauugccccCaaa.................   24     1   seq
....................................................................uaagcaccguuggauugccccaaaaA................   356    1   seq
....................................................................uaagcaccguuggauugccccaaaaC................   6      1   seq
....................................................................uaagcaccguuggauugccccaaaau................   2      0   seq
....................................................................uaagcaccguuggauugccccaaaaG................   4      1   seq
....................................................................uaagcaccguuggauugccccaaaaAu...............   1      1   seq
.....................................................................aagcaccguuggauugcccGa....................   1      1   seq
.....................................................................aagcaccguuggauugccccaa...................   37     0   seq
.....................................................................aagcaccAuuggauugccccaa...................   3      1   seq
.....................................................................aagcaccguuggauugccccCa...................   3      1   seq
.....................................................................Gagcaccguuggauugccccaa...................   1      1   seq
.....................................................................Uagcaccguuggauugccccaa...................   2      1   seq
.....................................................................aagcaccguuggauugccccUa...................   1      1   seq
.....................................................................aagcaccguuggauuUccccaa...................   1      1   seq
.....................................................................aagcaccguuggauugccccaaa..................   5      0   seq
.....................................................................aaUcaccguuggauugccccaaa..................   1      1   seq
.....................................................................aagcaccguuggauugccccaaaa.................   1      0   seq
......................................................................agcaccguuggauugccc......................   5      0   seq
......................................................................agcaccguuggauugccccU....................   1      1   seq
......................................................................agcaccguuggauugccccaa...................   4      0   seq
......................................................................aCcaccguuggauugccccaa...................   2      1   seq
......................................................................agcaccUuuggauugccccaa...................   1      1   seq
......................................................................agcaccguuggauugccccaaa..................   13     0   seq
......................................................................agcaccguuggauugccccaaU..................   2      1   seq
.......................................................................gcaccguuggauugccccaa...................   2      0   seq
.......................................................................gcaccguuggauugccccaaa..................   1      0   seq
.........................................................................accguuggauugccccaaa..................   1      0   seq
.........................................................................accguuggauugccccaaaa.................   1      0   seq
..........................................................................ccguuggauugccccaaa..................   3      0   seq
```
