# Supplementary material for: The Small RNA Universe of Capitella teleta
Source: Front Mol Biosci. 2022 Feb 25;9:802814. doi: 10.3389/fmolb.2022.802814 (PMC8915122; doi:10.3389/fmolb.2022.802814)
Supplement: Supplementary file 1 [file DataSheet1.ZIP › Supplement/confident/CAPTEscaffold_488_22719.pdf]

Provisional ID	:	CAPTEscaffold_488_22719
Score total	:	1185.8
Score for star read(s)	:	3.9
Score for read counts	:	1178.8
Score for mfe	:	2
Score for randfold	:	1.6
Score for cons. seed	:	-0.6
Total read count	:	2324
Mature read count	:	1310
Loop read count	:	0
Star read count	:	1014

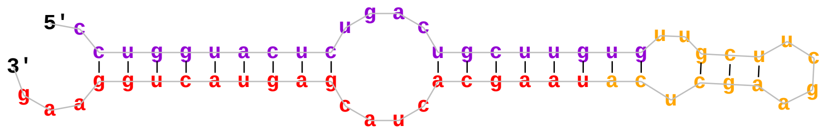

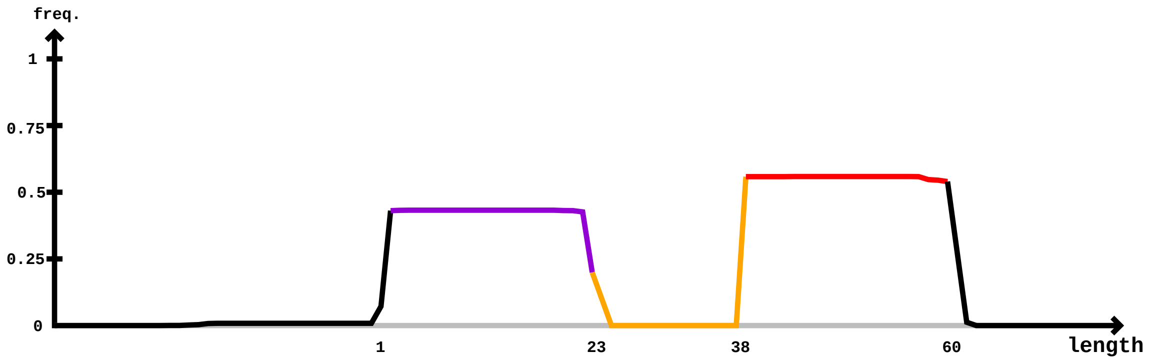

Star **Mature**

```
5'- uggugguaguugacgcuguuuggucuugccuuuccugguacucugacugcuuguguugcuucgaagcucauaagcacuacgaguacuggaagaggcagggucuaaaacu  -3'   obs
    uggugguaguugacgcuguuuggucuugccuuuccugguacucugacugcuuguguugcuucgaagcucauaagcacuacgaguacuggaagaggcagggucuaaaacu         exp
    .((((........)))).(((((((((((((((.(((((((((....((((((((..(((....))).))))))))....))))))))).)))))))))).)))))...   reads   mm   sample
    ..........ugacgcuguuuggucuugccuuu............................................................................   1       0    seq
    ............acgcuguuuggucuugccuuu............................................................................   4       0    seq
    .............cgcuguuuggucuugccuuu............................................................................   3       0    seq
    ..............gcuguuuggucuugccuuu............................................................................   9       0    seq
    ..............gcuguuGggucuugccuuu............................................................................   1       1    seq
    ...............cuguuuggucuugccuuu............................................................................   2       0    seq
    ................................uccugguacucugacugcu..........................................................   1       0    seq
    ................................uccugguacucugacugcuug........................................................   5       0    seq
    ................................uccugguacucugacugcuugu.......................................................   140     0    seq
    ................................uccugguacucugacugcuugA.......................................................   1       1    seq
    ................................uccugguacucugacugcuugugu.....................................................   1       0    seq
    ................................uccugguacucugGcugcuugugu.....................................................   1       1    seq
    .................................ccugguacucugacugcu..........................................................   2       0    seq
    .................................ccugguacucugacugcuu.........................................................   1       0    seq
    .................................ccugguacucugacugcuug........................................................   6       0    seq
    .................................ccugguacucCgacugcuugu.......................................................   1       1    seq
    .................................ccugguacucugacugcuugu.......................................................   389     0    seq
    .................................ccugguacucCgacugcuugug......................................................   5       1    seq
    .................................ccuAguacucugacugcuugug......................................................   2       1    seq
    .................................ccugguacucugacugcuGgug......................................................   1       1    seq
    .................................ccugguacucugacugcuugug......................................................   383     0    seq
    .................................ccuggAacucugacugcuugug......................................................   1       1    seq
    .................................ccugguacucugacugcuCgug......................................................   1       1    seq
    .................................ccugguacucugacugcuugugA.....................................................   26      1    seq
    .................................ccugguacucugacugcuugugG.....................................................   3       1    seq
    .................................ccugguacucugacugcuugugu.....................................................   39      0    seq
    .................................ccugguacucAgacugcuugugu.....................................................   1       1    seq
    ..................................cugguacucugacugcuugu.......................................................   1       0    seq
    ..................................cugguacucugacugcuugug......................................................   1       0    seq
    ..................................cugguacucugacugcuugugA.....................................................   1       1    seq
    ...................................ugguacucugacugcuugug......................................................   1       0    seq
    ......................................................................uaagcacuacgaguacug.....................   1       0    seq
    ......................................................................uaagcacuacgaguacugg....................   25      0    seq
```

```
                                 Star                                    Mature
uggugguaguugacgcuguuuggucuugccuuuccugguacucugacugcuuguguugcuucgaagcucauaagcacuacgaguacuggaagaggcagggucuaaaacu
......................................................................uaagcaAuacgaguacugg....................	1	1	seq
......................................................................uaagcacuacgaguaUugga...................	1	1	seq
......................................................................uaagcaUuacgaguacugga...................	1	1	seq
......................................................................uaagcacuacgaguacugga...................	3	0	seq
......................................................................uaagcacuacgaguacuggaa..................	9	0	seq
......................................................................uaagcacuacgaguacAggaa..................	1	1	seq
......................................................................uaagcacuacgaguaUuggaa..................	2	1	seq
......................................................................uaagcacAacgaguacuggaag.................	2	1	seq
......................................................................uaagcacuacgaguacugAaag.................	1	1	seq
......................................................................uaagcacuacgaguacuggaaA.................	1	1	seq
......................................................................uaagcacuacAaguacuggaag.................	2	1	seq
......................................................................uaagcacuacgaguaUuggaag.................	531	1	seq
......................................................................uaagcacCacgaguacuggaag.................	1	1	seq
......................................................................uaagcacuacgaguacuCgaag.................	1	1	seq
......................................................................uGagcacuacgaguacuggaag.................	1	1	seq
......................................................................uaagcacuacgaAuacuggaag.................	1	1	seq
......................................................................Aaagcacuacgaguacuggaag.................	3	1	seq
......................................................................uaagcaUuacgaguacuggaag.................	7	1	seq
......................................................................uaagcacuacgaguacuggaag.................	669	0	seq
......................................................................uaagcacuacgaguacuggaaga................	15	0	seq
......................................................................uaagcaUuacgaguacuggaaga................	1	1	seq
......................................................................uaagcacuacgaguaUuggaaga................	1	1	seq
......................................................................uaagcacuacgaguacuggaagaA...............	28	1	seq
...........................................................................acuacgaguacuggaagaA...............	1	1	seq
```
