# Supplementary material for: The Small RNA Universe of Capitella teleta
Source: Front Mol Biosci. 2022 Feb 25;9:802814. doi: 10.3389/fmolb.2022.802814 (PMC8915122; doi:10.3389/fmolb.2022.802814)
Supplement: Supplementary file 1 [file DataSheet1.ZIP › Supplement/confident/CAPTEscaffold_488_22731.pdf]

Provisional ID	:	CAPTEscaffold_488_22731
Score total	:	8646.5
Score for star read(s)	:	3.9
Score for read counts	:	8639.6
Score for mfe	:	1.9
Score for randfold	:	1.6
Score for cons. seed	:	-0.6
Total read count	:	16958
Mature read count	:	12239
Loop read count	:	0
Star read count	:	4719

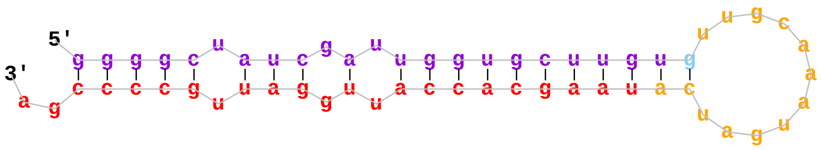

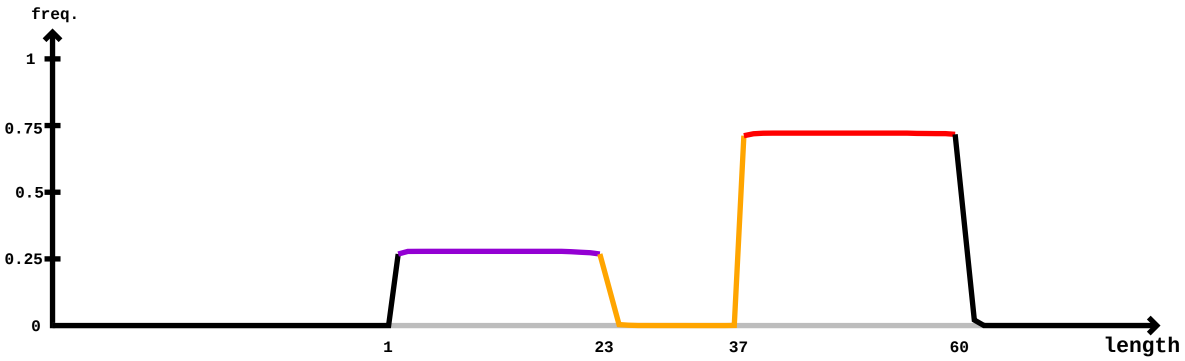


```
                                         Star                                     Mature
5'- uacuuuaauuucuggagauauucaucucaguuucggggcuaucgauuggugcuuguguugcaaaugaucauaagcaccauuggauugccccgacauuggaaucugcuaucauu   -3'    obs
    uacuuuaauuucuggagauauucaucucaguuucggggcuaucgauuggugcuuguguugcaaaugaucauaagcaccauuggauugccccgacauuggaaucugcuaucauu          exp
    ............(.((((.....((.((((.(((((((.(((.(.((((((((((((..........)))))))))))).).))).))))))).)))))))))).).......   reads    mm    sample
    .................................cggggcuaucgauuggugcuugug........................................................   1        0     seq
    ..................................ggggcuaucgauuggugc.............................................................   21       0     seq
    ..................................ggggcuaucUauuggugc.............................................................   1        1     seq
    ..................................ggggcuaucgauuggugcu............................................................   32       0     seq
    ..................................ggggcuaucgauuggugcuG...........................................................   1        1     seq
    ..................................ggggcuaucgauuggugcuu...........................................................   27       0     seq
    ..................................ggggcuaucgauuggugcuug..........................................................   80       0     seq
    ..................................Agggcuaucgauuggugcuug..........................................................   1        1     seq
    ..................................ggggcuaucgauuggugcuugG.........................................................   3        1     seq
    ..................................ggggcuaucgauuggugcuAgu.........................................................   1        1     seq
    ..................................ggAgcuaucgauuggugcuugu.........................................................   3        1     seq
    ..................................ggggcuaucgauuCgugcuugu.........................................................   1        1     seq
    ..................................ggggcAaucgauuggugcuugu.........................................................   1        1     seq
    ..................................ggggcuaucgauuggugcuugA.........................................................   1        1     seq
    ..................................ggggcuaucgauuggugcuugu.........................................................   2219     0     seq
    ..................................ggggcuaucgaCuggugcuugu.........................................................   1        1     seq
    ..................................ggggcuGucgauuggugcuugu.........................................................   1        1     seq
    ..................................ggggcuaucgUuuggugcuugu.........................................................   1        1     seq
    ..................................ggggcuaucgauuggugcuuUu.........................................................   1        1     seq
    ..................................ggggcuaucgauugAugcuugu.........................................................   2        1     seq
    ..................................gCggcuaucgauuggugcuugu.........................................................   1        1     seq
    ..................................gAggcuaucgauuggugcuugu.........................................................   3        1     seq
    ..................................ggggcuauUgauuggugcuugu.........................................................   8        1     seq
    ..................................ggggcuaucAauuggugcuugu.........................................................   7        1     seq
    ..................................gggAcuaucgauuggugcuugu.........................................................   1        1     seq
    ..................................ggggcuaucgauuggugcuugC.........................................................   1        1     seq
    ..................................ggggcuaucgaGuggugcuugu.........................................................   1        1     seq
    ..................................ggggcuaucgauuggugAuugu.........................................................   2        1     seq
    ..................................Ngggcuaucgauuggugcuugu.........................................................   1        1     seq
    ..................................Ugggcuaucgauuggugcuugu.........................................................   3        1     seq
    ..................................Agggcuaucgauuggugcuugu.........................................................   10       1     seq
    ..................................ggggcuaucgauuAgugcuugu.........................................................   8        1     seq
    ..................................ggggcuaucgauuggAgcuugu.........................................................   2        1     seq
```

Star Mature

```
uacuuuaauuucuggagauauucaucucaguuucggggcuaucgauuggugcuuguguugcaaaugaucauaagcaccauuggauugccccgacauuggaaucugcuaucauu
..................................ggggcuaAcgauuggugcuugu.........................................................	1	1	seq
..................................ggggcuaucgauuggugcuuUug........................................................	1	1	seq
..................................ggggcuaucgGuuggugcuugug........................................................	1	1	seq
..................................ggggcuGucgauuggugcuugug........................................................	1	1	seq
..................................ggggcuaucgauuAgugcuugug........................................................	5	1	seq
..................................Ugggcuaucgauuggugcuugug........................................................	2	1	seq
..................................ggAgcuaucgauuggugcuugug........................................................	2	1	seq
..................................ggggcuaucAauuggugcuugug........................................................	5	1	seq
..................................ggggcuaucgauuggugcuugAg........................................................	1	1	seq
..................................ggggcuaucgauugguUcuugug........................................................	1	1	seq
..................................ggggcuaucgauuggugcuAgug........................................................	1	1	seq
..................................ggggGuaucgauuggugcuugug........................................................	1	1	seq
..................................ggggcuaucgauuggAgcuugug........................................................	2	1	seq
..................................ggggcuaucgauuggugcuuguA........................................................	2	1	seq
..................................ggggcuaucgauuggugcuugCg........................................................	1	1	seq
..................................gAggcuaucgauuggugcuugug........................................................	5	1	seq
..................................Agggcuaucgauuggugcuugug........................................................	11	1	seq
..................................ggggcuaucgauuggugcuugug........................................................	1994	0	seq
..................................ggggcuauUgauuggugcuugug........................................................	4	1	seq
..................................ggggcuaucgauugAugcuugug........................................................	1	1	seq
..................................ggggcuaucgauuggugcuugugu.......................................................	22	0	seq
..................................ggggcuaucgauuggugcuugugG.......................................................	1	1	seq
..................................ggggcuaucgauuggugcuugugA.......................................................	15	1	seq
..................................ggggcuaucgauuggugcuugugC.......................................................	2	1	seq
..................................ggggcuaucgauuggugcuuguguu......................................................	6	0	seq
..................................ggggcuaucgauuggugcuuguguG......................................................	1	1	seq
..................................ggggcuaucgauuggugcuuguguC......................................................	1	1	seq
..................................ggggcuaucgauuggugcuuguguA......................................................	10	1	seq
..................................ggggcuaucgauuggugcuuguguug.....................................................	1	0	seq
..................................ggggcuaucgauuggugcuuguguugA....................................................	1	1	seq
..................................ggggcuaucgauuggugcuuguguGgca...................................................	1	1	seq
..................................ggggcuaucgauuggugcuuguguugcaaau................................................	1	0	seq
...................................gggcuaucgauuggugcu............................................................	3	0	seq
...................................gggcuaucgauuggugcuu...........................................................	3	0	seq
...................................gggcuaucgauuggugcuug..........................................................	1	0	seq
...................................gggcuaucgauuggugcuugu.........................................................	13	0	seq
...................................gCgcuaucgauuggugcuugug........................................................	1	1	seq
...................................Aggcuaucgauuggugcuugug........................................................	1	1	seq
...................................gggcuaucgauuggugcuugug........................................................	143	0	seq
....................................ggcuaucgauuggugcuugug........................................................	2	0	seq
.....................................gcuaucgauuggugcuu...........................................................	2	0	seq
.....................................................................auaagcaccauuggauugccccga....................	1	0	seq
.....................................................................aAaagcaccauuggauugccccga....................	7	1	seq
.....................................................................Cuaagcaccauuggauugccccga....................	1	1	seq
......................................................................uaagcaccauuggauugc.........................	18	0	seq
......................................................................uaagcaccaAuggauugc.........................	1	1	seq
......................................................................uaagcaccauuggauugcc........................	5	0	seq
......................................................................uaagcaccauuggauugccc.......................	6	0	seq
......................................................................uaagcaccauuggauugcccc......................	2	0	seq
......................................................................uaagcaccauuggauugccccg.....................	41	0	seq
......................................................................uaGgcaccauuggauugccccg.....................	1	1	seq
......................................................................uaagcacAauuggauugccccga....................	7	1	seq
......................................................................uaagcaGcauuggauugccccga....................	1	1	seq
......................................................................uaagcaccauuCgauugccccga....................	2	1	seq
......................................................................uaagcaccauuggGuugccccga....................	2	1	seq
......................................................................uaagcaccauuggaAugccccga....................	5	1	seq
......................................................................uaagcaccauuggauugccccgG....................	59	1	seq
......................................................................uaagcaccauuggauugccccgC....................	1	1	seq
......................................................................uaagcaccauuggUuugccccga....................	1	1	seq
......................................................................uaagcaccauugCauugccccga....................	2	1	seq
......................................................................uaagAaccauuggauugccccga....................	2	1	seq
......................................................................uaagcaccauuggauugcUccga....................	3	1	seq
......................................................................uaagcaccauuggauugccccAa....................	56	1	seq
......................................................................uaagcaccauuUgauugccccga....................	3	1	seq
......................................................................uaaAcaccauuggauugccccga....................	1	1	seq
......................................................................uaagcaccauuggauuAccccga....................	6	1	seq
......................................................................uaagcaccauAggauugccccga....................	5	1	seq
......................................................................uaagcaccauuggaCugccccga....................	2	1	seq
......................................................................uaagcaccauuggauuCccccga....................	2	1	seq
......................................................................uaagcaccUuuggauugccccga....................	26	1	seq
```

Star Mature

```
uacuuuaauuucuggagauauucaucucaguuucggggcuaucgauuggugcuuguguugcaaaugaucauaagcaccauuggauugccccgacauuggaaucugcuaucauu
......................................................................uaagcaccauuggauugccGcga....................   1       1   seq
......................................................................uaagcaUcauuggauugccccga....................   2       1   seq
......................................................................uaagcaccaCuggauugccccga....................   1       1   seq
......................................................................uaagcaccauuAgauugccccga....................   66      1   seq
......................................................................Gaagcaccauuggauugccccga....................   4       1   seq
......................................................................uaagcaccauuggauugcAccga....................   1       1   seq
......................................................................uaagcacUauuggauugccccga....................   15      1   seq
......................................................................uaagcaccauuggauugccccUa....................   1       1   seq
......................................................................uaagcaccauuggauuUccccga....................   1       1   seq
......................................................................uaagcaccauuggauCgccccga....................   5       1   seq
......................................................................uaagcaccauuggauugccccCa....................   2       1   seq
......................................................................Caagcaccauuggauugccccga....................   2       1   seq
......................................................................uaagcaccauuggauugccccgU....................   5       1   seq
......................................................................uaagcaccauuggauugccUcga....................   12      1   seq
......................................................................uaagcCccauuggauugccccga....................   1       1   seq
......................................................................uaagcaccauuggauugccccga....................   11102   0   seq
......................................................................uaagcaccaAuggauugccccga....................   7       1   seq
......................................................................uNagcaccauuggauugccccga....................   1       1   seq
......................................................................uaagcaccauugAauugccccga....................   2       1   seq
......................................................................uaagcaccauuggauugcccGga....................   2       1   seq
......................................................................uaagcaccauugUauugccccga....................   2       1   seq
......................................................................uGagcaccauuggauugccccga....................   4       1   seq
......................................................................uaagcaccauuggauugccAcga....................   16      1   seq
......................................................................uaUgcaccauuggauugccccga....................   1       1   seq
......................................................................uaagcaccauuggauAgccccga....................   6       1   seq
......................................................................uaagcaccauuggauugcccUga....................   17      1   seq
......................................................................uaagcaccauuggauugcccAga....................   7       1   seq
......................................................................uaagcaAcauuggauugccccga....................   4       1   seq
......................................................................uaaUcaccauuggauugccccga....................   1       1   seq
......................................................................uaagcacGauuggauugccccga....................   2       1   seq
......................................................................uaagcaccauuggauugUcccga....................   4       1   seq
......................................................................uaagcaccCuuggauugccccga....................   16      1   seq
......................................................................uaagcGccauuggauugccccga....................   1       1   seq
......................................................................uaagGaccauuggauugccccga....................   2       1   seq
......................................................................uaagcaccauCggauugccccga....................   1       1   seq
......................................................................Naagcaccauuggauugccccga....................   2       1   seq
......................................................................uaagcaccauuggauugccccgaU...................   7       1   seq
......................................................................uaagcaccGuuggauugccccgac...................   17      1   seq
......................................................................uaagcaccauuggauugccccgac...................   4       0   seq
......................................................................uaagcaccauuggauugccccgaA...................   111     1   seq
......................................................................uaagcaccGuuggauugccccgaca..................   9       1   seq
......................................................................uaagcaccauuggauugccccgaca..................   1       0   seq
......................................................................uaagcaccauuggauugccccgaAa..................   336     1   seq
......................................................................uaagcaccauuggauugccccgaAau.................   1       1   seq
.......................................................................aagcaccauuggauugccccg.....................   1       0   seq
.......................................................................Uagcaccauuggauugccccga....................   1       1   seq
.......................................................................aagcaccauAggauugccccga....................   2       1   seq
.......................................................................aagcaccauuggauugccccga....................   112     0   seq
.......................................................................aagcaccauuggauugccccAa....................   3       1   seq
.......................................................................aagcaccauuggauugcccUga....................   1       1   seq
.......................................................................aagcaccauuggauugccccgaA...................   1       1   seq
.......................................................................aagcaccauuggauugccccgaAa..................   3       1   seq
........................................................................agcaccauuggauugccccg.....................   1       0   seq
........................................................................Ggcaccauuggauugccccga....................   1       1   seq
........................................................................agcaccauuggauugccccga....................   29      0   seq
........................................................................agcaccauuggauugccccgaA...................   2       1   seq
........................................................................agcaccauuggauugccccgaU...................   1       1   seq
........................................................................agcaccauuggauugccccgaAa..................   1       1   seq
.........................................................................gcaccauuggauugccccgaU...................   5       1   seq
.........................................................................gcaccauuggauugccccgaA...................   1       1   seq
.............................................................................................cauuggaaucugcuauca..   1       0   seq
.............................................................................................cauuggaaucugcuaucauu   1       0   seq
```
