# Supplementary material for: The Small RNA Universe of Capitella teleta
Source: Front Mol Biosci. 2022 Feb 25;9:802814. doi: 10.3389/fmolb.2022.802814 (PMC8915122; doi:10.3389/fmolb.2022.802814)
Supplement: Supplementary file 1 [file DataSheet1.ZIP › Supplement/confident/CAPTEscaffold_488_22725.pdf]

Provisional ID	:	CAPTEscaffold_488_22725
Score total	:	34.7
Score for star read(s)	:	3.9
Score for read counts	:	29.7
Score for mfe	:	0.1
Score for randfold	:	1.6
Score for cons. seed	:	-0.6
Total read count	:	70
Mature read count	:	62
Loop read count	:	0
Star read count	:	8

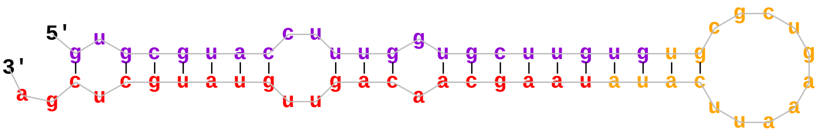

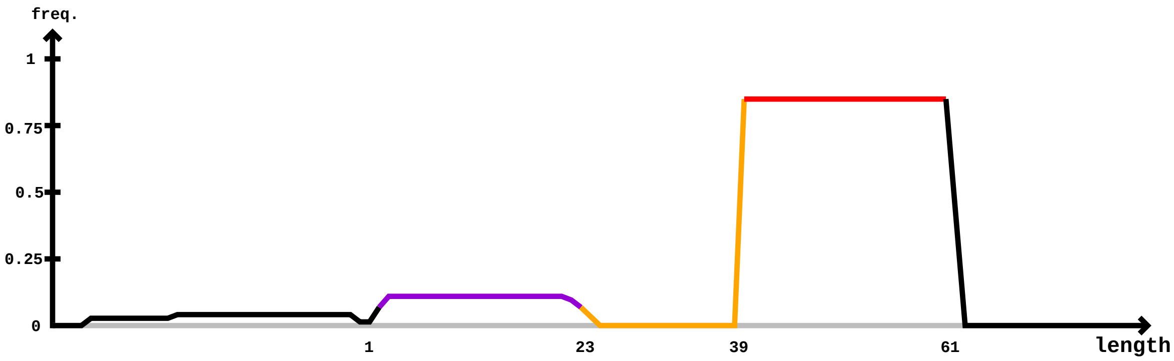

Star Mature

```
5'- agaucuugcauauuuauaucagacuggcucuugugcguaccuuuggugcuuguguugcgcugaaauucauauaagcaacaguuguaugcucgaaagacgauucuguugcaccg  -3'   obs
    agaucuugcauauuuauaucagacuggcucuugugcguaccuuuggugcuuguguugcgcugaaauucauauaagcaacaguuguaugcucgaaagacgauucuguugcaccg         exp
    ......((((.........((((.(.(((.(((.((((((..(((.((((((((((..........)))))))))).)))..)))))).))).)).).).)))).))))...   reads   mm   sample
    ..aucuugcauauuuauaucagacuggcuc..................................................................................   2       0    seq
    ...........auuuauaucagacuggcucuu................................................................................   1       0    seq
    ................................gugcguaccuuuggugcuug............................................................   1       0    seq
    ................................gugcguaUcuuuggugcuugu...........................................................   2       1    seq
    ................................gugcguaUcuuuggugcuugug..........................................................   1       1    seq
    ................................gugcguaccuuuggugcuugug..........................................................   1       0    seq
    .................................ugcguaccuuuggugcuugug..........................................................   1       0    seq
    .................................ugcguaUcuuuggugcuugugu.........................................................   1       1    seq
    .................................ugcguaccuuuggugcuugugu.........................................................   1       0    seq
    ......................................................................uaagcaacaguuguaugcucga....................   60      0    seq
    ......................................................................Aaagcaacaguuguaugcucga....................   2       1    seq
```
