# Supplementary material for: The Small RNA Universe of Capitella teleta
Source: Front Mol Biosci. 2022 Feb 25;9:802814. doi: 10.3389/fmolb.2022.802814 (PMC8915122; doi:10.3389/fmolb.2022.802814)
Supplement: Supplementary file 1 [file DataSheet1.ZIP › Supplement/confident/CAPTEscaffold_60_5441.pdf]

Provisional ID	:	CAPTEscaffold_60_5441
Score total	:	809.5
Score for star read(s)	:	3.9
Score for read counts	:	803.6
Score for mfe	:	1
Score for randfold	:	1.6
Score for cons. seed	:	-0.6
Total read count	:	1588
Mature read count	:	1378
Loop read count	:	0
Star read count	:	210

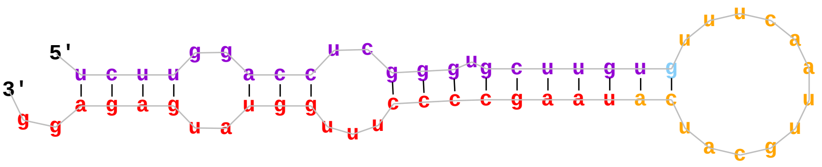

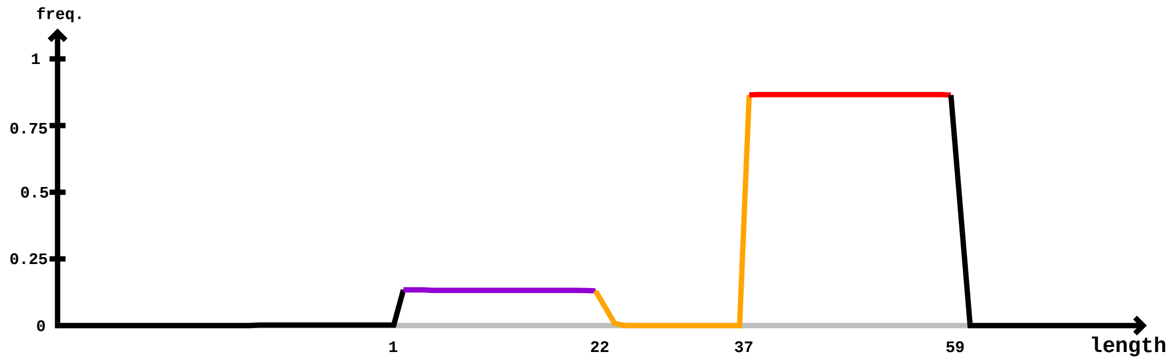


```
                                   Star                               Mature
5'- gcaucaagaauuauguuuaguaugauuggcucuuucuuggaccucgggugcuuguguuucaauugcaucauaagccccuuugguaugagagggaacugaugcucagucugc  -3'   obs
    gcaucaagaauuauguuuaguaugauuggcucuuucuuggaccucgggugcuugugguuucaauugcaucauaagccccuuugguaugagagggaacugaugcucagucugc        exp
    ((((((..(((((((.....)))))))...((((((((..(((..(((.(((((((............))))))))))...)))..))))))))..)))))).........   reads   mm   sample
    ...................guaugauuggcucuuucu..........................................................................   2       0    seq
    ...................gCaugauuggcucuuucu..........................................................................   1       1    seq
    ..................................ucuuggaccucgggugcuu..........................................................   1       0    seq
    ..................................ucuuggaccucgggugcuug.........................................................   2       0    seq
    ..................................ucuuggaccucgggugcuugu........................................................   145     0    seq
    ..................................ucuuggaccucgggugcuugA........................................................   2       1    seq
    ..................................ucuuAgaccucgggugcuugu........................................................   2       1    seq
    ..................................ucuuggaccucgggugcuugug.......................................................   41      0    seq
    ..................................ucuuggaccucgggugcuuguU.......................................................   1       1    seq
    ..................................Acuuggaccucgggugcuugug.......................................................   1       1    seq
    ..................................ucuuggaGcucgggugcuugug.......................................................   1       1    seq
    ..................................ucuuggaccucgggugcuuguA.......................................................   2       1    seq
    ..................................ucuuggaccucgggugcuugugu......................................................   2       0    seq
    ..................................ucuuggaccucgggugcuugugG......................................................   2       1    seq
    ..................................ucuuggaccucgggugcuugugA......................................................   7       1    seq
    ..................................ucuuggaccucgggugcuugugAu.....................................................   1       1    seq
    ......................................................................uaagccccuuugguaugagag....................   3       0    seq
    ......................................................................uaagccccGuugguaugagagg...................   1       1    seq
    ......................................................................uaagccccuuugguaugagagA...................   1       1    seq
    ......................................................................uaagccccuuuAguaugagagg...................   7       1    seq
    ......................................................................uaagccccuuugguaugagUgg...................   1       1    seq
    ......................................................................uaagccccuuGgguaugagagg...................   1       1    seq
    ......................................................................uaagccccuuugguauAagagg...................   3       1    seq
    ......................................................................uaagccccuuugguaugagaAg...................   13      1    seq
    ......................................................................uaagccccuuuggGaugagagg...................   1       1    seq
    ......................................................................uaagccAcuuugguaugagagg...................   3       1    seq
    ......................................................................uaagccccuAugguaugagagg...................   1       1    seq
    ......................................................................uaagccccuuugguaugaUagg...................   1       1    seq
    ......................................................................uaagccccuuugguaCgagagg...................   1       1    seq
    ......................................................................uGagccccuuugguaugagagg...................   1       1    seq
    ......................................................................uaagccccuuugguaugagagC...................   2       1    seq
    ......................................................................Gaagccccuuugguaugagagg...................   1       1    seq
    ......................................................................uaagcccUuuugguaugagagg...................   2       1    seq
```

Star **Mature**

```
gcaucaagaauuauguuuaguaugauuggcucuuucuuggaccucgggugcuuguguuucaauugcaucauaagccccuuugguaugagagggaacugaugcucagucugc
......................................................................uaagccccuuugguaugagagU...................    2      1    seq
......................................................................uaagccccuuugguaugagagg...................    1321   0    seq
......................................................................Aaagccccuuugguaugagagg...................    7      1    seq
......................................................................uaagccccuuAgguaugagagg...................    1      1    seq
......................................................................uaagccccuuugguaugagaggA..................    2      1    seq
.......................................................................aagccccGuugguaugagagg...................    1      1    seq
.......................................................................aagccccuuugguaugagagg...................    1      0    seq
```
