# Supplementary material for: The Small RNA Universe of Capitella teleta
Source: Front Mol Biosci. 2022 Feb 25;9:802814. doi: 10.3389/fmolb.2022.802814 (PMC8915122; doi:10.3389/fmolb.2022.802814)
Supplement: Supplementary file 1 [file DataSheet1.ZIP › Supplement/confident/CAPTEscaffold_62_6279.pdf]

Provisional ID	:	CAPTEscaffold_62_6279
Score total	:	236.7
Score for star read(s)	:	3.9
Score for read counts	:	230.1
Score for mfe	:	1.8
Score for randfold	:	1.6
Score for cons. seed	:	-0.6
Total read count	:	463
Mature read count	:	347
Loop read count	:	0
Star read count	:	116

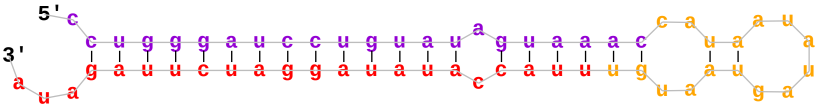

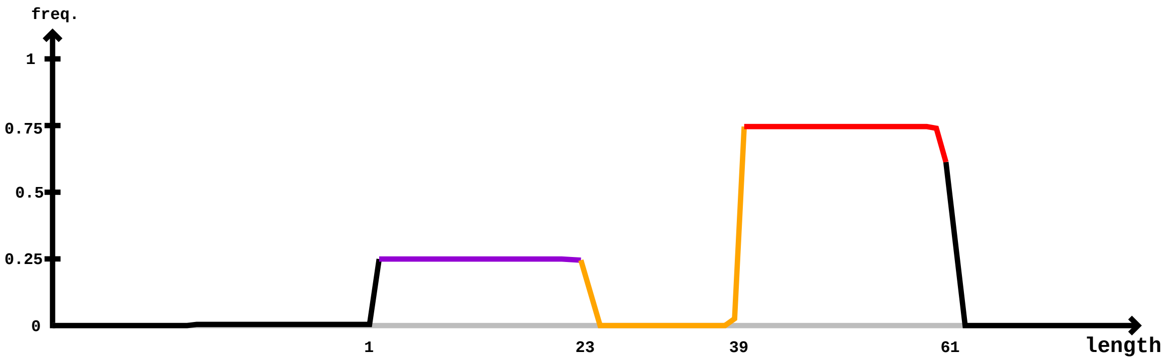


```
                                Star                                  Mature
5'- accauauaccgcauaugauauugauaggccuaccugggauccuguauaguaaaccauaauauaguaauguuuaccauauaggaucuuagauaggccuacauugacaaauuu  -3'  obs
    accauauaccgcauaugauauugauaggccuaccugggauccuguauaguaaaccauaauauaguaauguuuaccauauaggaucuuagauaggccuacauugacaaauuu       exp
    ..(((((.....)))))....((.((((((((.((((((((((((((.((((((..((......))..)))))).)))))))))))))).))))))))))...........   reads   mm   sample
    .............uaugauauugauaggccuac..............................................................................   2       0    seq
    ................................ccugggauccuguauaguaa...........................................................   1       0    seq
    ................................ccugggauccuguauaguaaa..........................................................   1       0    seq
    ................................ccugggauccuguauaguaaac.........................................................   111     0    seq
    ................................ccAgggauccuguauaguaaac.........................................................   1       1    seq
    .................................cugggauccuguauaguaaacc........................................................   2       0    seq
    .....................................................................uuuaccauauaggaucuuagau....................   11      0    seq
    .....................................................................uuuaccauauaggaucuuagaua...................   1       0    seq
    ......................................................................uuaccauauaggaucuuaga.....................   3       0    seq
    ......................................................................uuaccauauaggaucuuagau....................   48      0    seq
    ......................................................................uuaccaAauaggaucuuagaua...................   1       1    seq
    ......................................................................uAaccauauaggaucuuagaua...................   1       1    seq
    ......................................................................uuaccauauaggaucuuagaua...................   274     0    seq
    ......................................................................uuaccauauaggaucuuagauaA..................   8       1    seq
```
