# Supplementary material for: The Small RNA Universe of Capitella teleta
Source: Front Mol Biosci. 2022 Feb 25;9:802814. doi: 10.3389/fmolb.2022.802814 (PMC8915122; doi:10.3389/fmolb.2022.802814)
Supplement: Supplementary file 1 [file DataSheet1.ZIP › Supplement/confident/CAPTEscaffold_324_18315.pdf]

Provisional ID : CAPTEscaffold_324_18315
Score total : 365.3
Score for star read(s) : 3.9
Score for read counts : 358.5
Score for mfe : 1.9
Score for randfold : 1.6
Score for cons. seed : -0.6
Total read count : 715
Mature read count : 379
Loop read count : 0
Star read count : 336

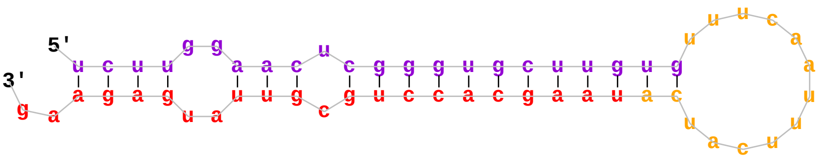

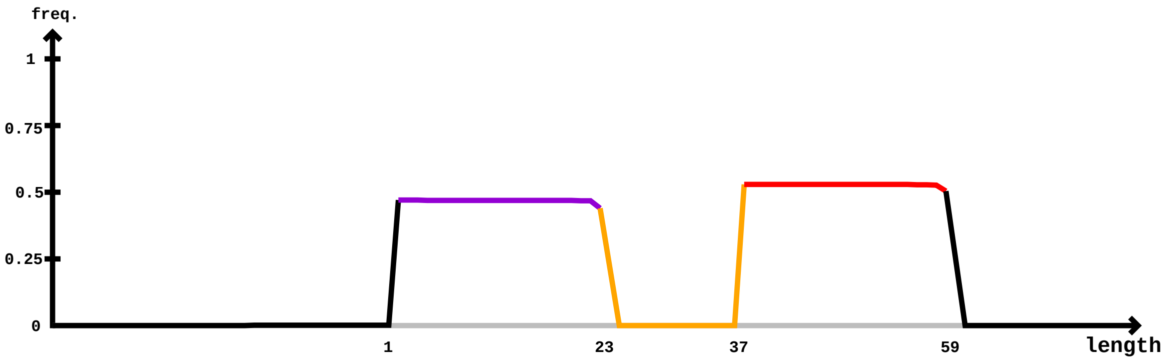


```
                                  Star                                  Mature
5'- ugaucaagaauuguguugaguaugauuggcuuuuucuuggaacucgggugcuuguguuucaauuucaucauaagcaccugcguuaugagaagaagcugaugcuuggucugc   -3'   obs
    ugaucaagaauuguguugaguaugauuggcuuuuucuuggaacucgggugcuuguguuucaauuucaucauaagcaccugcguuaugagaagaagcugaugcuuggucugc         exp
    .(((((((.(((......)))...((((((((((((((..(((.((((((((((((............))))))))))))).)))..)))))))))))))).)))))))...   reads   mm   sample
    ...................guaugauuggcAuuuucu..........................................................................   1       1    seq
    ..................................ucuuggaacuUgggugcuu..........................................................   1       1    seq
    ..................................ucuuggaacucgggugcuugu........................................................   8       0    seq
    ..................................ucuuggaacucAggugcuugu........................................................   1       1    seq
    ..................................ucuuggaacuUgggugcuugu........................................................   11      1    seq
    ..................................ucuuggaacuUgggugcuugug.......................................................   11      1    seq
    ..................................ucuuggaacucgAgugcuugug.......................................................   2       1    seq
    ..................................Ncuuggaacucgggugcuugug.......................................................   2       1    seq
    ..................................ucuuggaGcucgggugcuugug.......................................................   1       1    seq
    ..................................ucuuggaacucgggugcuuguA.......................................................   1       1    seq
    ..................................ucuuggaacucgggAgcuugug.......................................................   1       1    seq
    ..................................ucuuggaacucgggugcuugug.......................................................   280     0    seq
    ..................................ucuuggaacucgggugcuugugG......................................................   2       1    seq
    ..................................ucuuggaacucgggugcuugugA......................................................   15      1    seq
    ......................................................................uaagcaccugcguuauga.......................   1       0    seq
    ......................................................................uaagcaccugcguuaugaga.....................   1       0    seq
    ......................................................................uaagcaccugcguuaugagaa....................   16      0    seq
    ......................................................................uaagcaccugcguuauAagaag...................   1       1    seq
    ......................................................................uaagcaccugcguuaugagaag...................   357     0    seq
    ......................................................................uaagcaccugAguuaugagaag...................   1       1    seq
    ......................................................................uaaAcaccugcguuaugagaag...................   1       1    seq
    ......................................................................uaagcaccugcguuaugagaagU..................   1       1    seq
```
