# Supplementary material for: The Small RNA Universe of Capitella teleta
Source: Front Mol Biosci. 2022 Feb 25;9:802814. doi: 10.3389/fmolb.2022.802814 (PMC8915122; doi:10.3389/fmolb.2022.802814)
Supplement: Supplementary file 1 [file DataSheet1.ZIP › Supplement/confident/CAPTEscaffold_488_22715.pdf]

Provisional ID	:	CAPTEscaffold_488_22715
Score total	:	378
Score for star read(s)	:	3.9
Score for read counts	:	371.3
Score for mfe	:	1.8
Score for randfold	:	1.6
Score for cons. seed	:	-0.6
Total read count	:	740
Mature read count	:	718
Loop read count	:	0
Star read count	:	22

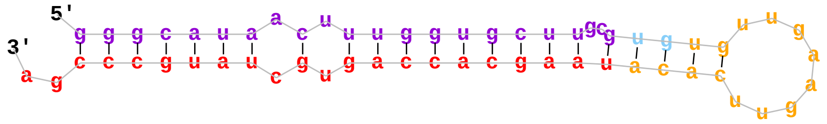

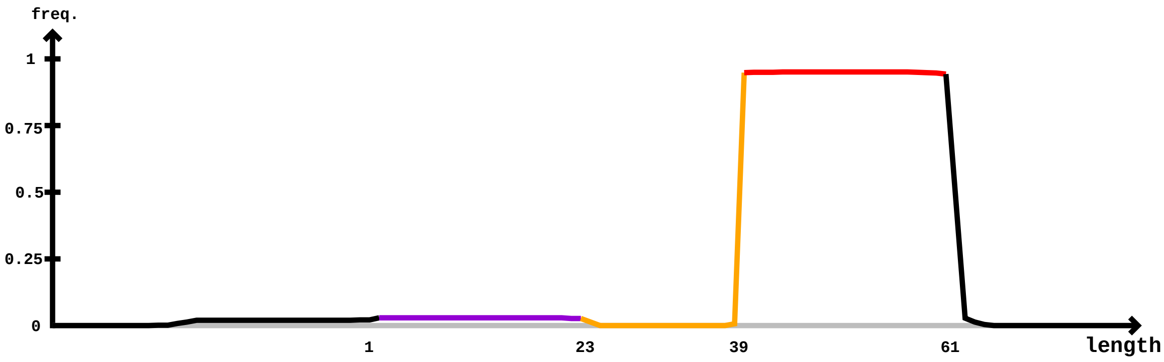


```
                                  Star                                   Mature
5'- auggugcauaauuacaucacgaacuggcucuugggcauaacuuuggugcuugcguguguugaaguucacauaagcaccagugcuaugcccgaaagacgauucuguugcacg  -3'  obs
    auggugcauaauuacaucacgaacuggcucuugggcauaacuuuggugcuugcguguguugaaguucacauaagcaccagugcuaugcccgaaagacgauucuguugcacg       exp
    ...(((((.....(((....(((.((.((.(((((((((.(.(((((((((..(((((........))))))))))))))).).))))))))).)).)).))))))))))).  reads   mm   sample
    .........aauuacaucacgaacuggcucuu...............................................................................  1       0    seq
    ...........uuacaucacgaacuggcucuu...............................................................................  5       0    seq
    ............uacaucacgaacuggcucuu...............................................................................  4       0    seq
    .............acaucacgaacuggcucuu...............................................................................  5       0    seq
    ..............................uugggcauaacuuuggugcuug...........................................................  1       0    seq
    ................................gggAauaacuuuggugcuug...........................................................  1       1    seq
    ................................gggcauaacuuuggugcuugUg.........................................................  20      1    seq
    .....................................................................auaagcaccagugcuaugcccga...................  5       0    seq
    ......................................................................uaagcaccagugcuaugc.......................  1       0    seq
    ......................................................................uaagcaccagugcuaugcc......................  1       0    seq
    ......................................................................uaagcaccagugcuaugccc.....................  1       0    seq
    ......................................................................uaagcaccagugcuaugcccg....................  2       0    seq
    ......................................................................uaagcaccagugcuaAgcccg....................  1       1    seq
    ......................................................................uaagcaccGgugcuaugcccga...................  1       1    seq
    ......................................................................uaagcaccagugcuaugcccAa...................  2       1    seq
    ......................................................................uaagcaGcagugcuaugcccga...................  1       1    seq
    ......................................................................uaagcaccaguAcuaugcccga...................  1       1    seq
    ......................................................................uaagcaccagugcuaugUccga...................  1       1    seq
    ......................................................................uaagcaccagugcuaugcUcga...................  1       1    seq
    ......................................................................uaagcaccagugcuaugccUga...................  1       1    seq
    ......................................................................uaagcacUagugcuaugcccga...................  1       1    seq
    ......................................................................uaagcaccagugcuaugcccgG...................  5       1    seq
    ......................................................................uaagcaccagugcuaugAccga...................  1       1    seq
    ......................................................................uaagcaccagugcuaAgcccga...................  1       1    seq
    ......................................................................uaagcaccagugcuaugcccga...................  662     0    seq
    ......................................................................Aaagcaccagugcuaugcccga...................  3       1    seq
    ......................................................................uaagcaccagugcuaugcccgaC..................  1       1    seq
    ......................................................................uaagcaccagugcuaugcccgaa..................  3       0    seq
    ......................................................................uaagcaccagugcuaugcccgaaa.................  11      0    seq
    ......................................................................uaagcaccagugcuaugcccgaaaA................  6       1    seq
    ......................................................................uaagcaccagugcuaugcccgaaaAa...............  3       1    seq
    .......................................................................aagcaccagugcuaugcccgaaaA................  1       1    seq
    ..........................................................................caccagugcuaugcccga...................  1       0    seq
```

Star Mature

auggugcauaauuacaucacgaacuggcucuugggcauaacuuuggugcuugcgugguguugaaguucacauaagcaccagugcuaugcccgaaagacgauucuguugcacg
