# Supplementary material for: The Small RNA Universe of Capitella teleta
Source: Front Mol Biosci. 2022 Feb 25;9:802814. doi: 10.3389/fmolb.2022.802814 (PMC8915122; doi:10.3389/fmolb.2022.802814)
Supplement: Supplementary file 1 [file DataSheet1.ZIP › Supplement/confident/CAPTEscaffold_472_22934.pdf]

Provisional ID	:	CAPTEscaffold_472_22934
Score total	:	1126.8
Score for star read(s)	:	3.9
Score for read counts	:	1119.7
Score for mfe	:	2.2
Score for randfold	:	1.6
Score for cons. seed	:	-0.6
Total read count	:	2208
Mature read count	:	2171
Loop read count	:	0
Star read count	:	37

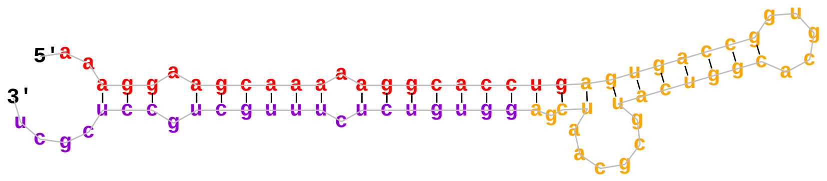

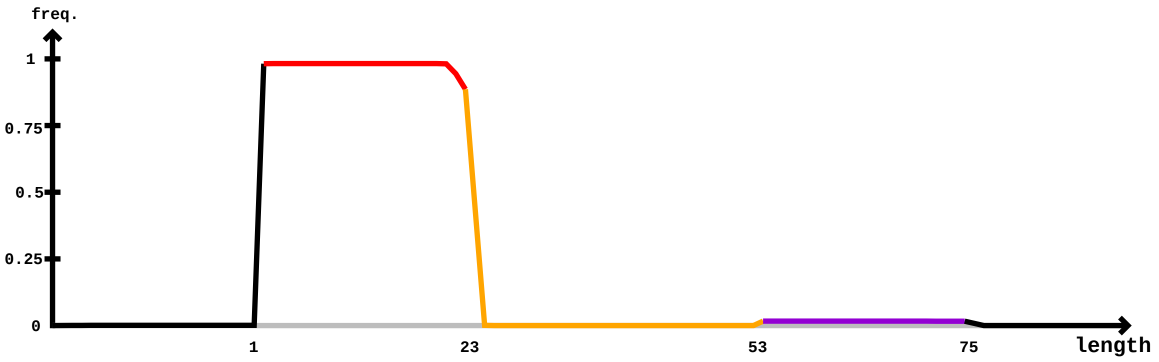

Mature   Star

```
5'- gacacuggucagcggggaggaaaggaagcaaaaaggcaccugagugaccggugcacggucaugcgcaaucgaggugucucuuugcugccucgcuugccuguugguuaaac  -3'  obs
    gacacuggucagcggggaggaaaggaagcaaaaaggcaccugagugaccggugcacggucaugcgcaaucgaggugucucuuugcugccucgcuugccuguugguuaaac       exp
    .....(((((((((((.(((..(((.((((((.(((((((((((((((((.....))))))).......)).)))))))).)))))).)))..))).)))))))))))...   reads   mm   sample
    gacacuggucagcAgggagg..........................................................................................   1       1    seq
    ..cacuggucagcAgggagg..........................................................................................   1       1    seq
    ....................aaaggaagcaaaaaggcac.......................................................................   2       0    seq
    ....................aaaggaagcaaaaaggcGcc......................................................................   1       1    seq
    ....................aaaggaagcaaaaaggcacG......................................................................   1       1    seq
    ....................aaaggaagcaaaaaggcacc......................................................................   80      0    seq
    ....................aaaggaagcaaaaaggcaccu.....................................................................   123     0    seq
    ....................aaaggaagcaaaaaggcacUu.....................................................................   2       1    seq
    ....................aaaggaagAaaaaaggcaccu.....................................................................   1       1    seq
    ....................aaaggaagcaaaaaggcaccG.....................................................................   1       1    seq
    ....................aaaggaagcaaaaaggcaAcu.....................................................................   1       1    seq
    ....................aGaggaagcaaaaaggcaccu.....................................................................   1       1    seq
    ....................Naaggaagcaaaaaggcaccug....................................................................   2       1    seq
    ....................aaaggaagcUaaaaggcaccug....................................................................   1       1    seq
    ....................aaaggaagcaaaaaggUaccug....................................................................   1       1    seq
    ....................aaaggaagcaaaaaggcaccuA....................................................................   4       1    seq
    ....................aaaggaagcaaaaaggcaccGg....................................................................   1       1    seq
    ....................aaaggaagcaaaaaggcaGcug....................................................................   1       1    seq
    ....................aaaggaagcaaaaaggcaccuU....................................................................   5       1    seq
    ....................aaaAgaagcaaaaaggcaccug....................................................................   5       1    seq
    ....................aaaggaagcaaaaaggcaccAg....................................................................   1       1    seq
    ....................aaaggaagcaaaaGggcaccug....................................................................   1       1    seq
    ....................aaagUaagcaaaaaggcaccug....................................................................   1       1    seq
    ....................aaaggaagcaaaaaggcaccuC....................................................................   1       1    seq
    ....................aaaggaagcaaaaaggcaccug....................................................................   1812    0    seq
    ....................Gaaggaagcaaaaaggcaccug....................................................................   1       1    seq
    ....................aGaggaagcaaaaaggcaccug....................................................................   17      1    seq
    ....................aaaggaagcaaaaaggcacAug....................................................................   4       1    seq
    ....................aaaggaagcaaaGaggcaccug....................................................................   1       1    seq
    ....................aaaggaGgcaaaaaggcaccug....................................................................   1       1    seq
    ....................aaaggaagcaaaaagAcaccug....................................................................   1       1    seq
    ....................aaaggaagUaaaaaggcaccug....................................................................   2       1    seq
    ....................aaagAaagcaaaaaggcaccug....................................................................   1       1    seq
```

Mature **Star**

```
gacacuggucagcggggaggaaaggaagcaaaaaggcaccugagugaccggugcacggucaugcgcaaucgaggugucucuuugcugccucgcuugccuguugguuaaac
....................aaaggaagcaaaaaggcUccug....................................................................    1     1     seq
....................aaaggaagcaaaaaggcaccugU...................................................................    1     1     seq
....................aaaggaagcaaaaaggUaccuga...................................................................    1     1     seq
....................aaaggaagcaaaaaggcaccugG...................................................................    1     1     seq
....................aaaggaagcaaaaaggcaccuga...................................................................    87    0     seq
....................aaaggaagcaaaaaggcaccugag..................................................................    1     0     seq
....................aaaggaagcaaaaaggcaccugaA..................................................................    1     1     seq
.....................aaggaagcaaaaaggcacUug....................................................................    1     1     seq
........................................................................ggugucucuuugcugccu....................    1     0     seq
........................................................................ggugucucuuugcugccucgcG................    1     1     seq
........................................................................ggugucucuuugcugccucgcu................    35    0     seq
```
