# Supplementary material for: The Small RNA Universe of Capitella teleta
Source: Front Mol Biosci. 2022 Feb 25;9:802814. doi: 10.3389/fmolb.2022.802814 (PMC8915122; doi:10.3389/fmolb.2022.802814)
Supplement: Supplementary file 1 [file DataSheet1.ZIP › Supplement/confident/CAPTEscaffold_488_22729.pdf]

Provisional ID	:	CAPTEscaffold_488_22729
Score total	:	38.5
Score for star read(s)	:	3.9
Score for read counts	:	36.8
Score for mfe	:	0.6
Score for randfold	:	-2.2
Score for cons. seed	:	-0.6
Total read count	:	84
Mature read count	:	61
Loop read count	:	0
Star read count	:	23

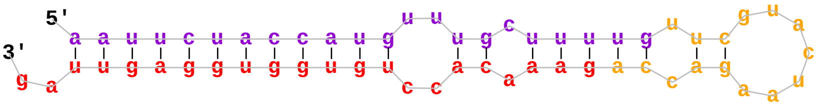

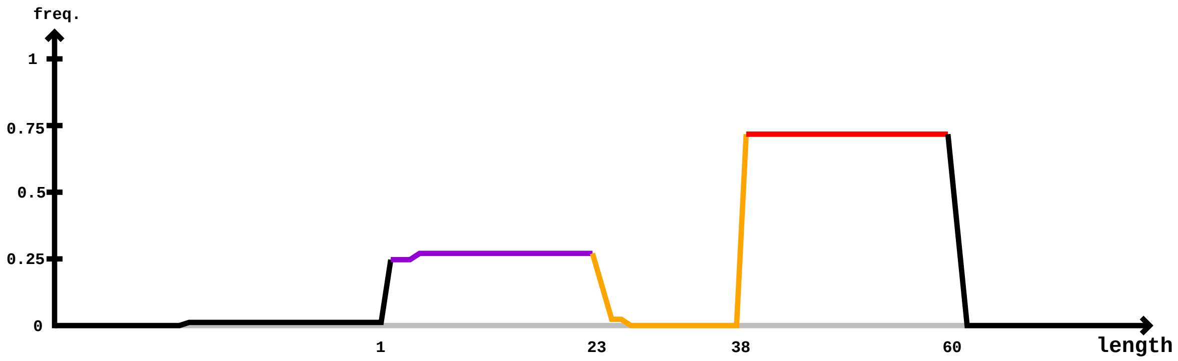


```
                                          Star                                       Mature
5'- agaguagucuucucuuauggcgaaaccucucugaauucuaccauguuugcuuuuguucguacuaagaccagaaacaccugugguggaguuaggggcggggucuaaaucagcu   -3'    obs
    agaguagucuucucuuauggcgaaaccucucugaauucuaccauguuugcuuuuguucguacuaagaccagaaacaccugugguggaguuaggggcggggucuaaaucagcu          exp
    ((((.......))))......((..((.((((.((((((((((((..((.(((((.((.......)).))))).))..)))))))))))))).)))).))..))..........   reads      mm         sample
    ............ucuuauggcgaaaccucucug...............................................................................   1          0          seq
    .................................aauucuaccauguuugcuuuug.........................................................   21         0          seq
    ....................................ucuaccauguuugcuuuuguuc......................................................   2          0          seq
    ......................................................................Uaaacaccugugguggaguuag....................   1          1          seq
    ......................................................................Aaaacaccugugguggaguuag....................   1          1          seq
    ......................................................................gaaacaccugugguggaguuag....................   43         0          seq
    ......................................................................gaaacGccugugguggaguuag....................   1          1          seq
    ......................................................................gaaacaccuguAguggaguuag....................   1          1          seq
    ......................................................................gaaacaccugugguggaguuagU...................   1          1          seq
    ......................................................................gaaacaccugugguggaguuagA...................   13         1          seq
```
