# Supplementary material for: The Small RNA Universe of Capitella teleta
Source: Front Mol Biosci. 2022 Feb 25;9:802814. doi: 10.3389/fmolb.2022.802814 (PMC8915122; doi:10.3389/fmolb.2022.802814)
Supplement: Supplementary file 1 [file DataSheet1.ZIP › Supplement/confident/CAPTEscaffold_154_12129.pdf]

Provisional ID	:	CAPTEscaffold_154_12129
Score total	:	1.8
Score for star read(s)	:	-1.3
Score for read counts	:	0
Score for mfe	:	2.1
Score for randfold	:	1.6
Score for cons. seed	:	-0.6
Total read count	:	120
Mature read count	:	76
Loop read count	:	0
Star read count	:	44

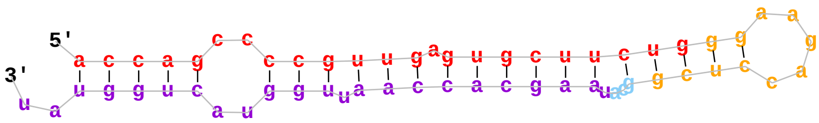

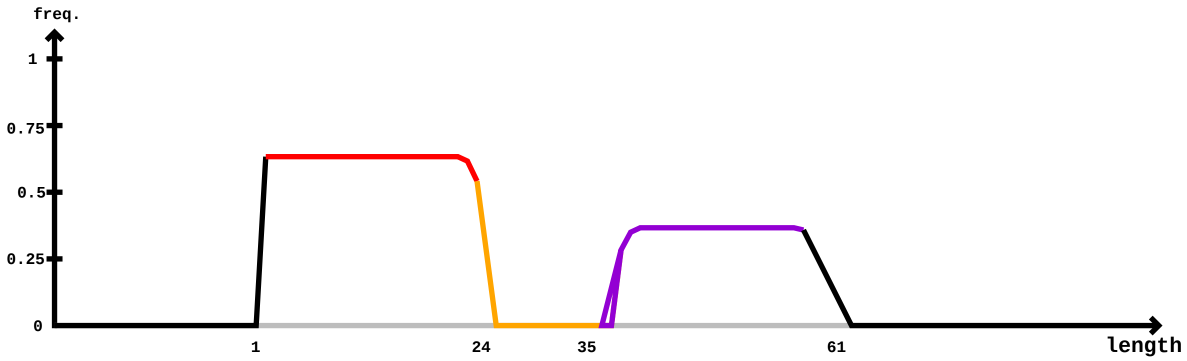

Mature Star

```
5'- aagagagggagagcgucgagaccagccccguugagugcuucugggaagaccucggcauaagcaccaauugguacugguaucgacgcugcucaacgucaucacgcacgucucaa  -3'   obs
    aagagagggagagcgucgagaccagccccguugagugcuucugggaagaccucggcauaagcaccaauugguacugguaucgacgcugcucaacgucaucacgcacgucucaa         exp
    ..((((.((((((((((((.(((((..((((((.(((((((((((.....)))))...))))))))).)))..))))).)))))))).)))..(((....)))..).))))..   reads   mm   sample
    ....................accagccccguugagugcuuc........................................................................   2       0    seq
    ....................accagccccguugagugcuucu.......................................................................   9       0    seq
    ....................acAagccccguugagugcuucug......................................................................   1       1    seq
    ....................Gccagccccguugagugcuucug......................................................................   1       1    seq
    ....................accagcccUguugagugcuucug......................................................................   1       1    seq
    ....................accagccccguugagugcuucug......................................................................   62      0    seq
    .........................................................uaagcaccaauugguacuggua..................................   1       0    seq
    .........................................................uaagcaccaauugguacugguau.................................   31      0    seq
    .........................................................uaagcaccaauugguacugguaC.................................   2       1    seq
    ..........................................................aagcaccaauugguacug.....................................   1       0    seq
    ..........................................................aagcaccaauugguacugguau.................................   5       0    seq
    ..........................................................aagcaccaauugguacugguauU................................   2       1    seq
    ...........................................................agcaccaauugguacugguau.................................   1       0    seq
    ...........................................................agcaccaauugguacugguauU................................   1       1    seq
```
