# Supplementary material for: The Small RNA Universe of Capitella teleta
Source: Front Mol Biosci. 2022 Feb 25;9:802814. doi: 10.3389/fmolb.2022.802814 (PMC8915122; doi:10.3389/fmolb.2022.802814)
Supplement: Supplementary file 1 [file DataSheet1.ZIP › Supplement/confident/CAPTEscaffold_93_7673.pdf]

Provisional ID	:	CAPTEscaffold_93_7673
Score total	:	346.9
Score for star read(s)	:	3.9
Score for read counts	:	341.2
Score for mfe	:	0.8
Score for randfold	:	1.6
Score for cons. seed	:	-0.6
Total read count	:	681
Mature read count	:	558
Loop read count	:	0
Star read count	:	123

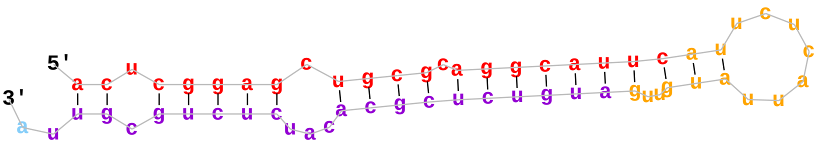

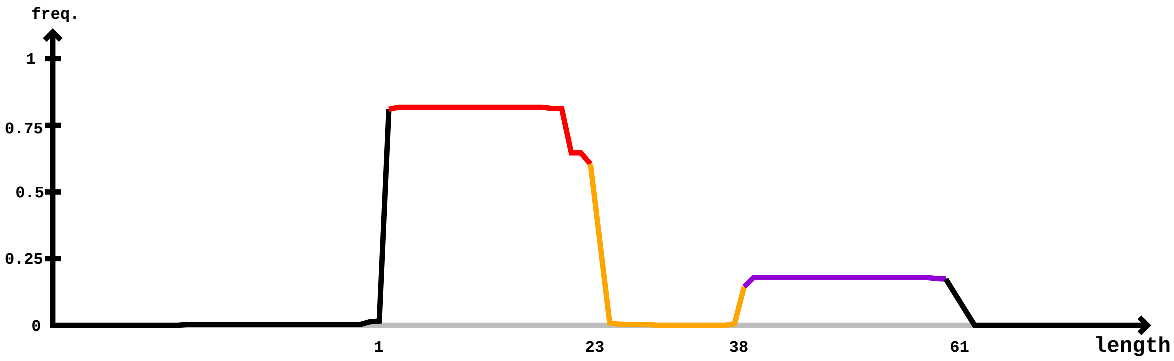

Mature Star

```
5'- gguuuuuuacuauggaucuguagauugagccauacucggagcugcgcaggcauucauucucauuauguugaugucucgcacaucucugcguuagguuuguuuuacuaacaac   -3'   obs
    gguuuuuuacuauggaucuguagauugagccauacucggagcugcgcaggcauucauucucauuauguugaugucucgcacaucucugcguuagguuuguuuuacuaacaac         exp
    (((((........))))).(((((.((((((..((.(((((.((((.((((((((((.......)))..)))))))))))...))))).))..)))))).))))).......    reads   mm   sample
    ............uggaucuguagauugagcc.................................................................................    2       0    seq
    ...............................auacucggagcugcgcagA..............................................................    3       1    seq
    ...............................auacucggagcugcgcaggca............................................................    6       0    seq
    ................................uacucggagcugcgcagAcauu..........................................................    2       1    seq
    .................................acucggagcugcgcaggca............................................................    105     0    seq
    .................................acucggagcugcgcaggcG............................................................    1       1    seq
    .................................acuUggagcugcgcaggca............................................................    1       1    seq
    .................................Gcucggagcugcgcaggca............................................................    1       1    seq
    .................................acucggagcugcgcagAcauu..........................................................    2       1    seq
    .................................acucggagcugcgcaggcauu..........................................................    24      0    seq
    .................................acucggagcugcgcaCgcauuc.........................................................    1       1    seq
    .................................acucggagcugcgcagCcauuc.........................................................    2       1    seq
    .................................acucggagcugcgcaggcauuc.........................................................    271     0    seq
    .................................acucggagcugcgcaggcauuU.........................................................    76      1    seq
    .................................acAcggagcugcgcaggcauuc.........................................................    1       1    seq
    .................................acucggagcAgcgcaggcauuc.........................................................    1       1    seq
    .................................acucggagcugcgcagAcauuc.........................................................    1       1    seq
    .................................Gcucggagcugcgcaggcauuc.........................................................    6       1    seq
    .................................acuUggagcugcgcaggcauuc.........................................................    1       1    seq
    .................................acucggagcugcgcagAcauuca........................................................    2       1    seq
    .................................acucggagcugcgcaggcauucU........................................................    1       1    seq
    .................................acucggagcugcgcaggcauuca........................................................    41      0    seq
    .................................acucggagcugcgcaggcauucau.......................................................    2       0    seq
    .................................acucggagcugcgcaggcauucaA.......................................................    1       1    seq
    .................................acucggagcugcgcaggcauucaAu......................................................    1       1    seq
    .................................acucggagcugcgcaggcauucauucuc...................................................    2       0    seq
    ..................................cucggagcugcgcaggcauu..........................................................    1       0    seq
    ..................................cucggagcugcgcaggcauuc.........................................................    4       0    seq
    .....................................................................gaugucucgcacaucucugcguu....................    2       0    seq
    .....................................................................gaugucucgcacaCcucugcguu....................    2       1    seq
    ......................................................................augucucgcacaucucugcg......................    3       0    seq
    ......................................................................augucucgcacaucucugcgu.....................    1       0    seq
    ......................................................................Gugucucgcacaucucugcguu....................    1       1    seq
```

Mature Star

```
gguuuuuuacuauggaucuguagauugagccauacucggagcugcgcaggcauucauucucauuauguugaugucucgcacaucucugcguuagguuuguuuuacuaacaac
......................................................................augucucgcacaucucugAguu....................	1	1	seq
......................................................................augucucgcGcaucucugcguu....................	2	1	seq
......................................................................augucucgcacaucucugcguu....................	76	0	seq
......................................................................augucucgcacaucucugcguua...................	9	0	seq
......................................................................augucucgcacaucucugcguGa...................	1	1	seq
......................................................................augucucgcacaucAcugcguua...................	1	1	seq
.......................................................................ugucucgcacaucucugcguu....................	12	0	seq
.......................................................................ugucucgcacaucucugcguua...................	12	0	seq
```
