# Supplementary material for: The Small RNA Universe of Capitella teleta
Source: Front Mol Biosci. 2022 Feb 25;9:802814. doi: 10.3389/fmolb.2022.802814 (PMC8915122; doi:10.3389/fmolb.2022.802814)
Supplement: Supplementary file 1 [file DataSheet1.ZIP › Supplement/confident/CAPTEscaffold_19739_45967.pdf]

| | | |
|---|---|---|
| Provisional ID | : | CAPTEscaffold_19739_45967 |
| Score total | : | 2780.1 |
| Score for star read(s) | : | 3.9 |
| Score for read counts | : | 2780.2 |
| Score for mfe | : | -1.1 |
| Score for randfold | : | -2.2 |
| Score for cons. seed | : | -0.6 |
| Total read count | : | 5465 |
| Mature read count | : | 4491 |
| Loop read count | : | 0 |
| Star read count | : | 974 |

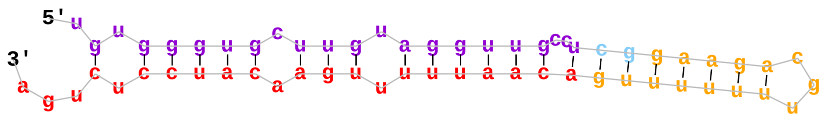


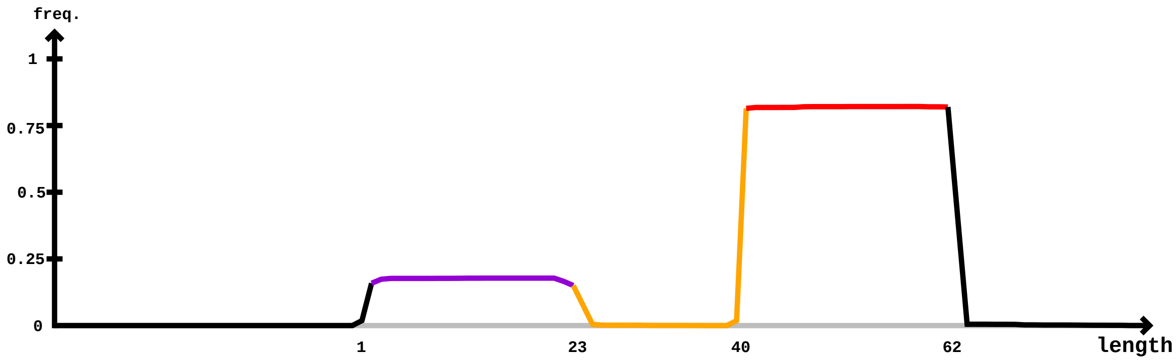


Star Mature

| | | | | |
|---|---|---|---|---|
| 5'- | aaucuuuggaggaggcggccaaaaguuaaaucugugggugcuuguagguugccucggaagacguuuuuuugacaauuuuugaacauccucugaugcuagucucgugaaccaga | -3' | obs | |
| | aaucuuuggaggaggcggccaaaaguuaaaucugugggugcuuguagguugccucggaagacguuuuuuugacaauuuuugaacauccucugaugcuagucucgugaaccaga | | exp | |
| | ....((((((.((((((((..........(((.(.(((((.(((.((((((..((((((((...)))))))))))))).))).))))).).)))))).))))).)...))))) | reads | mm | sample |
| | ...............................Gugugggugcuuguagguugc............................................................. | 2 | 1 | seq |
| | ...............................Gugugggugcuuguagguugcc............................................................ | 25 | 1 | seq |
| | ...............................Gugugggugcuuguagguugccu........................................................... | 73 | 1 | seq |
| | ...............................Uugugggugcuuguagguugccu........................................................... | 1 | 1 | seq |
| | ...............................Gugugggugcuuguagguugccuc.......................................................... | 1 | 1 | seq |
| | ................................ugugggugcuuguagguugc............................................................. | 3 | 0 | seq |
| | ................................uguAggugcuuguagguugcc............................................................ | 1 | 1 | seq |
| | ................................ugugggugcuuguagguugcc............................................................ | 57 | 0 | seq |
| | ................................ugugAgugcuuguagguugcc............................................................ | 1 | 1 | seq |
| | ................................ugugggugcuuguagguugAcu........................................................... | 1 | 1 | seq |
| | ................................ugugggugcuuguagguugccu........................................................... | 650 | 0 | seq |
| | ................................ugugggugcuuguagguugccG........................................................... | 5 | 1 | seq |
| | ................................uguAggugcuuguagguugccu........................................................... | 5 | 1 | seq |
| | ................................Ggugggugcuuguagguugccu........................................................... | 1 | 1 | seq |
| | ................................ugugggugcuuguagguugccC........................................................... | 1 | 1 | seq |
| | ................................Agugggugcuuguagguugccu........................................................... | 1 | 1 | seq |
| | ................................ugugAgugcuuguagguugccu........................................................... | 1 | 1 | seq |
| | ................................ugugggAgcuuguagguugccu........................................................... | 1 | 1 | seq |
| | ................................ugugggugcuuguGgguugccu........................................................... | 1 | 1 | seq |
| | ................................ugugggugcuuguagguugccA........................................................... | 22 | 1 | seq |
| | ................................ugugggugcuuguagguAgccu........................................................... | 1 | 1 | seq |
| | ................................ugugggugcuuguagguugccuc.......................................................... | 8 | 0 | seq |
| | ................................ugugggugcuuguagguugccuU.......................................................... | 3 | 1 | seq |
| | ................................ugugggugcuuguagguugccuA.......................................................... | 1 | 1 | seq |
| | ................................ugugggugcuuguagguugccucA......................................................... | 2 | 1 | seq |
| | ................................ugugggugcuuguagguugccucU......................................................... | 3 | 1 | seq |
| | .................................gugggugcuuguagguugc............................................................. | 61 | 0 | seq |
| | .................................gugggugcuuAuagguugc............................................................. | 1 | 1 | seq |
| | .................................Uugggugcuuguagguugccu........................................................... | 1 | 1 | seq |
| | .................................gugggugcuuguagguugccu........................................................... | 14 | 0 | seq |
| | .................................gugggugcuuguagguugccuc.......................................................... | 4 | 0 | seq |
| | .................................gugggugcuuguagguugccucU......................................................... | 2 | 1 | seq |
| | ..................................ugggugcuuguagguugcc............................................................ | 1 | 0 | seq |

```
                               Star                                   Mature
aaucuuuggaggaggcggccaaaaguuaaucugugggugcuuguagguugccucggaagacguuuuuuugacaauuuuugaacauccucugaugcuagucucgugaaccaga
.................................ugggugcuuguagguugccu...........................................................   1     0   seq
.................................ugggugcuuguagguugccuc..........................................................   3     0   seq
.................................ugggugcuuguagguugccucU.........................................................   3     1   seq
.................................ugggugcuuguagguugccucg.........................................................   7     0   seq
.................................ugggugcuuguagguugccucgg........................................................   1     0   seq
.................................ugggugcuuguagguugccucggaagacguu................................................   1     0   seq
......................................gcuuguagguugccucggaaga....................................................   2     0   seq
........................................uuguagguugccucggaaga....................................................   1     0   seq
.........................................uguagguugccucggaag.....................................................   1     0   seq
.........................................uguagguugccucggaagac...................................................   1     0   seq
...........................................uagguugccucggaagacguuuC..............................................   1     1   seq
.....................................................................gacaauuuuCgaacauccucug.....................   1     1   seq
.....................................................................gacaauuuuCgaacauccucuga....................   100   1   seq
.....................................................................gacaauuuuCgaacauccucugau...................   1     1   seq
......................................................................acaauuuuCgaacauccuc.......................   9     1   seq
......................................................................acaauuuuCgaacauccucug.....................   4     1   seq
......................................................................acaauuuuugaacauccucuga....................   20    0   seq
......................................................................acaauuuuAgaacauccucuga....................   1     1   seq
......................................................................acaauuuuCgaacauccucuga....................   4226  1   seq
......................................................................acaauuuuAgaacauccucugau...................   1     1   seq
......................................................................acaauuuuCgaacauccucugau...................   108   1   seq
......................................................................acaauuuuCgaacauccucugaugcu................   2     1   seq
......................................................................acaauuuuCgaacauccucugaugcuagu.............   1     1   seq
.......................................................................caauuuuCgaacauccucuga....................   10    1   seq
.......................................................................caauuuuCgaacauccucugau...................   7     1   seq
.......................................................................caauuuuCgaacauccucugaugcuagucu...........   1     1   seq
...........................................................................uuuCgaacauccucuga....................   1     1   seq
............................................................................uuCgaacauccucugaugcuag..............   1     1   seq
............................................................................uuCgaacauccucugaugcuagu.............   8     1   seq
............................................................................uuCgaacauccucugaugcuagucu...........   1     1   seq
............................................................................uuCgaacauccucugaugcuagucucgug.......   2     1   seq
............................................................................uuCgaacauccucugaugcuagucucguga......   2     1   seq
.............................................................................uCgaacauccucugaugcuagu.............   1     1   seq
.............................................................................uCgaacauccucugaugcuagucu...........   1     1   seq
.................................................................................acauccucugaugcuagucuGgu........   1     1   seq
.................................................................................acauccucugaugcuagucucgugaGcca..   1     1   seq
.......................................................................................ucugaugcuagucucgugaGc....   1     1   seq
.........................................................................................ugaugcuagucucgugaGcca..   3     1   seq
...........................................................................................augcuagucucgugaGccaga   1     1   seq
```
