# Supplementary material for: The Small RNA Universe of Capitella teleta
Source: Front Mol Biosci. 2022 Feb 25;9:802814. doi: 10.3389/fmolb.2022.802814 (PMC8915122; doi:10.3389/fmolb.2022.802814)
Supplement: Supplementary file 1 [file DataSheet1.ZIP › Supplement/confident/CAPTEscaffold_488_22717.pdf]

Provisional ID	:	CAPTEscaffold_488_22717
Score total	:	193144.1
Score for star read(s)	:	3.9
Score for read counts	:	193137.2
Score for mfe	:	2
Score for randfold	:	1.6
Score for cons. seed	:	-0.6
Total read count	:	378842
Mature read count	:	374993
Loop read count	:	0
Star read count	:	3849

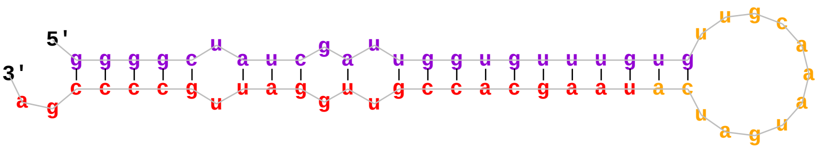

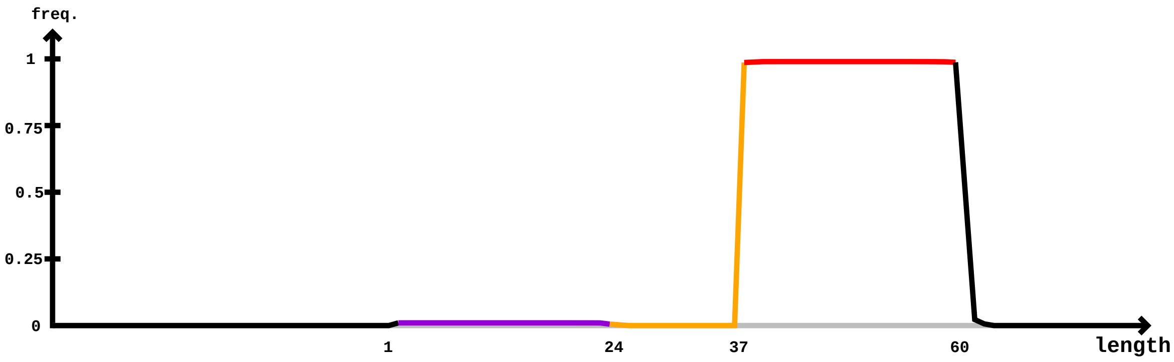

Star Mature

```
5'- caccguaauuuucggagauauucgucucaguuucggggcuaucgauugguguuuguguugcaaaugaucauaagcaccguuggauugccccgaaauuggaaucugcuaucau  -3'   obs
    caccguaauuuucggagauauucgucucaguuucggggcuaucgauugguguuuguguugcaaaugaucauaagcaccguuggauugccccgaaauuggaaucugcuaucau        exp
    ..(((.......))).((((....((.((((((((((((.(((.(.((((((((((...........)))))))))).).))).)))))))))))))......))))..   reads   mm   sample
    ...............agauauucgucucaguuuc..............................................................................   1   0   seq
    .................................cggggcuaucgauugguguuugug.......................................................   2   0   seq
    ..................................ggggcuaucgauuggugu............................................................   2   0   seq
    ..................................ggggcuaucgauugguguu...........................................................   13   0   seq
    ..................................ggggcuaucgauugguguuu..........................................................   3   0   seq
    ..................................ggggcuaucgauAgguguuug.........................................................   1   1   seq
    ..................................ggggcuaucgauugguguuuA.........................................................   1   1   seq
    ..................................ggggcuaucgauugguguuug.........................................................   89   0   seq
    ..................................Agggcuaucgauugguguuug.........................................................   1   1   seq
    ..................................ggggcuaucgauugguguuuCu........................................................   1   1   seq
    ..................................ggggcuaucgauugguguuugG........................................................   1   1   seq
    ..................................ggggcuaucgauuggugAuugu........................................................   2   1   seq
    ..................................ggggcuaucgauugguguuAgu........................................................   1   1   seq
    ..................................ggggcuaucgauugguguuugu........................................................   1645   0   seq
    ..................................ggggcuaucAauugguguuugu........................................................   5   1   seq
    ..................................Ngggcuaucgauugguguuugu........................................................   1   1   seq
    ..................................ggggcuaucgauuAguguuugu........................................................   5   1   seq
    ..................................ggggcuaucgauAgguguuugu........................................................   1   1   seq
    ..................................ggggcuauUgauugguguuugu........................................................   4   1   seq
    ..................................ggggcuaucgauuggAguuugu........................................................   1   1   seq
    ..................................Agggcuaucgauugguguuugu........................................................   8   1   seq
    ..................................ggggGuaucgauugguguuugu........................................................   1   1   seq
    ..................................ggggcuaucgauugguguuCgu........................................................   1   1   seq
    ..................................ggggcuaucgauugguguuugA........................................................   1   1   seq
    ..................................ggggcuauAgauugguguuugu........................................................   1   1   seq
    ..................................ggggcuaucgauugguguuugug.......................................................   1944   0   seq
    ..................................Ugggcuaucgauugguguuugug.......................................................   2   1   seq
    ..................................ggggcuaucgaAugguguuugug.......................................................   1   1   seq
    ..................................ggggcuaucgauugUuguuugug.......................................................   1   1   seq
    ..................................ggggcuauAgauugguguuugug.......................................................   1   1   seq
    ..................................ggCgcuaucgauugguguuugug.......................................................   1   1   seq
    ..................................ggggcuaucAauugguguuugug.......................................................   10   1   seq
    ..................................ggggcuaucgauugguguuuguA.......................................................   4   1   seq
```

```
                                  Star                                Mature
caccguaauuuucggagauauucgucucaguuucggggcuaucgauugguguuuguguugcaaaugaucauaagcaccguuggauugccccgaaauuggaaucugcuaucau
..................................ggggcuauUgauugguguuugug.......................................................    6          1          seq
..................................ggggcuaucgauugguguuuAug.......................................................    1          1          seq
..................................ggggcuaucgauuggAguuugug.......................................................    3          1          seq
..................................ggggcuaucgauuAguguuugug.......................................................    6          1          seq
..................................ggAgcuaucgauugguguuugug.......................................................    2          1          seq
..................................ggggcuaucCauugguguuugug.......................................................    1          1          seq
..................................ggggcuaucgauugguguCugug.......................................................    1          1          seq
..................................ggggcuaucgauugguguuCgug.......................................................    1          1          seq
..................................ggggcuaucgauuggguAuuugug.......................................................    1          1          seq
..................................ggggcuaucgauugguguuAgug.......................................................    1          1          seq
..................................ggggcuaucgauugguguAugug.......................................................    1          1          seq
..................................gAggcuaucgauugguguuugug.......................................................    5          1          seq
..................................Agggcuaucgauugguguuugug.......................................................    14         1          seq
..................................ggggcuaucgauugguguuuguU.......................................................    4          1          seq
..................................ggggcuaucgauugguguuugugC......................................................    1          1          seq
..................................ggggcuaucgauugguguuugugA......................................................    6          1          seq
..................................ggggcuaucgauugguguuugugG......................................................    2          1          seq
..................................ggggcuaucgauugguguuugugu......................................................    17         0          seq
..................................ggggcuaucgauugguguuuguguu.....................................................    8          0          seq
..................................ggggcuaucgauugguguuuguguA.....................................................    8          1          seq
..................................ggggcuaucgauugguguuuguguugc...................................................    1          0          seq
...................................gggcuaucgauuAguguuugug.......................................................    1          1          seq
...................................gggcuaucgauugguguuugug.......................................................    1          0          seq
....................................ggcuaucgauugguguuugu........................................................    1          0          seq
.......................................uaucgauugguguuugug.......................................................    1          0          seq
...............................................................augaucauaagcaccguuggau...........................    5          0          seq
................................................................ugaucauaagcaccguuggau...........................    5          0          seq
...................................................................ucauaagcaccguuggauugccA......................    1          1          seq
....................................................................Aauaagcaccguuggauugccccga...................    1          1          seq
.....................................................................Cuaagcaccguuggauugccccga...................    5          1          seq
......................................................................uaagcaccguuggauugc........................    32         0          seq
......................................................................uaagcaccguuggauugcc.......................    38         0          seq
......................................................................uaagcaccguuAgauugccc......................    1          1          seq
......................................................................Aaagcaccguuggauugccc......................    2          1          seq
......................................................................uaagcaccguuggauugccc......................    93         0          seq
......................................................................uaagcaccguuggauugccA......................    2          1          seq
......................................................................uaagcaccguuAgauugcccc.....................    2          1          seq
......................................................................uaagcaccguuggauugcccA.....................    1          1          seq
......................................................................uaagcaccguuggauugccAc.....................    2          1          seq
......................................................................Aaagcaccguuggauugcccc.....................    2          1          seq
......................................................................uaagcaccguuggauugcccc.....................    131        0          seq
......................................................................uaagcaccguuggauugccccU....................    6          1          seq
......................................................................uaagcaccguuggauugccAcg....................    1          1          seq
......................................................................uaagcaccguuggauugcccGg....................    2          1          seq
......................................................................uaagcaccguuAgauugccccg....................    3          1          seq
......................................................................uaagcacUguuggauugccccg....................    1          1          seq
......................................................................uaagcacAguuggauugccccg....................    1          1          seq
......................................................................uaagcaccguuggauugUcccg....................    1          1          seq
......................................................................uaagcaccguuggauugccccg....................    658        0          seq
......................................................................uaagcaccguuggauugcccAg....................    1          1          seq
......................................................................Aaagcaccguuggauugccccg....................    2          1          seq
......................................................................uaagcaccguuggauugccccC....................    3          1          seq
......................................................................uaagcaccgAuggauugccccg....................    1          1          seq
......................................................................uaagcaccguuggaCugccccga...................    36         1          seq
......................................................................uaagcaccguuCgauugccccga...................    28         1          seq
......................................................................uaUgcaccguuggauugccccga...................    14         1          seq
......................................................................uGagcaccguuggauugccccga...................    181        1          seq
......................................................................uaagUaccguuggauugccccga...................    39         1          seq
......................................................................uaagcaccguuggauAgccccga...................    256        1          seq
......................................................................uaagcaccguuggauugcUccga...................    130        1          seq
......................................................................uaagcaccguuggauugccccUa...................    35         1          seq
......................................................................uaagcaccguuggauugccccga...................    350737     0          seq
......................................................................Aaagcaccguuggauugccccga...................    1674       1          seq
......................................................................uaagcaccguuggauugcccGga...................    42         1          seq
......................................................................uaagcaccUuuggauugccccga...................    26         1          seq
......................................................................uaagcaccguCggauugccccga...................    63         1          seq
......................................................................uaagcaccguuggauugUcccga...................    78         1          seq
......................................................................uaagcaccguuggauugccccgC..................    110        1          seq
......................................................................uaagcaccguuggaAugccccga...................    140        1          seq
......................................................................uaagcaccguuggauCgccccga...................    84         1          seq
```

```
                                  Star                               Mature
caccguaauuuucggagauauucgucucaguuucggggcuaucgauugguguuuguguugcaaaugaucauaagcaccguuggauugccccgaaauuggaaucugcuaucau
......................................................................uaagcaccguuggauugccGcga...................   39     1   seq
......................................................................uaagcaccguuggauuAccccga...................   50     1   seq
......................................................................uaaAcaccguuggauugccccga...................   31     1   seq
......................................................................uaagcaccguuggCuugccccga...................   2      1   seq
......................................................................uaagcaccgGuggauugccccga...................   19     1   seq
......................................................................uaagcaccguuggauuUccccga...................   30     1   seq
......................................................................uaagcaccguuggaGugccccga...................   4      1   seq
......................................................................uaagcaccguuggauugcccUga...................   524    1   seq
......................................................................uaagGaccguuggauugccccga...................   18     1   seq
......................................................................uUagcaccguuggauugccccga...................   3      1   seq
......................................................................uaagcaccguAggauugccccga...................   182    1   seq
......................................................................uaagcaccguuggauugccAcga...................   611    1   seq
......................................................................Gaagcaccguuggauugccccga...................   146    1   seq
......................................................................uaagcaccguuUgauugccccga...................   41     1   seq
......................................................................uaagcaAcguuggauugccccga...................   108    1   seq
......................................................................uaagcaccguuggauugccccgU...................   246    1   seq
......................................................................uaagcaccguuggauugccccgG...................   1964   1   seq
......................................................................uaNgcaccguuggauugccccga...................   2      1   seq
......................................................................uaaCcaccguuggauugccccga...................   21     1   seq
......................................................................uaagcaccguugUauugccccga...................   49     1   seq
......................................................................uaagcNccguuggauugccccga...................   2      1   seq
......................................................................uaagcaccCuuggauugccccga...................   16     1   seq
......................................................................uaagcacAguuggauugccccga...................   193    1   seq
......................................................................uaagcaccgAuggauugccccga...................   207    1   seq
......................................................................uaagcUccguuggauugccccga...................   47     1   seq
......................................................................uNagcaccguuggauugccccga...................   21     1   seq
......................................................................uaGgcaccguuggauugccccga...................   50     1   seq
......................................................................Caagcaccguuggauugccccga...................   35     1   seq
......................................................................uaagcaccguuggauugccccCa...................   58     1   seq
......................................................................uaagcaGcguuggauugccccga...................   49     1   seq
......................................................................uaagcaccguuggauugcccAga...................   128    1   seq
......................................................................uaagcCccguuggauugccccga...................   9      1   seq
......................................................................uaagcacUguuggauugccccga...................   296    1   seq
......................................................................Naagcaccguuggauugccccga...................   113    1   seq
......................................................................uaCgcaccguuggauugccccga...................   1      1   seq
......................................................................uaagcaccguuggauugcAccga...................   104    1   seq
......................................................................uaagcaccguuggauugAcccga...................   68     1   seq
......................................................................uaagcaccguuggauugcGccga...................   32     1   seq
......................................................................uaagcaccgCuggauugccccga...................   36     1   seq
......................................................................uaagcaccguuAgauugccccga...................   1738   1   seq
......................................................................uaagcaUcguuggauugccccga...................   36     1   seq
......................................................................uaagAaccguuggauugccccga...................   64     1   seq
......................................................................uaagcaccguuggauugGcccga...................   17     1   seq
......................................................................uaaUcaccguuggauugccccga...................   28     1   seq
......................................................................uaagcaccguuggUuugccccga...................   32     1   seq
......................................................................uaagcGccguuggauugccccga...................   48     1   seq
......................................................................uaagcaccguuggauuCccccga...................   37     1   seq
......................................................................uaagcaccguugCauugccccga...................   28     1   seq
......................................................................uaagcaccguugAauugccccga...................   66     1   seq
......................................................................uaagcaccguuggauugccUcga...................   334    1   seq
......................................................................uaagcaccguuggauugccccgN...................   1      1   seq
......................................................................uaagcaccguGggauugccccga...................   22     1   seq
......................................................................uaagcaccguuggauGgccccga...................   3      1   seq
......................................................................uaagcaccguuggGugccccga...................   53     1   seq
......................................................................uaagcacGguuggauugccccga...................   10     1   seq
......................................................................uaagcaccguuUgauugccccgaa..................   1      1   seq
......................................................................uaagcaccguuggUuugccccgaa..................   1      1   seq
......................................................................uaagcaAcguuggauugccccgaa..................   2      1   seq
......................................................................uaagcaccguuggauugccUcgaa..................   1      1   seq
......................................................................Caagcaccguuggauugccccgaa..................   1      1   seq
......................................................................uaagcaccgGuggauugccccgaa..................   1      1   seq
......................................................................uaagcaccguuggauugccccgaC..................   17     1   seq
......................................................................uaagAaccguuggauugccccgaa..................   1      1   seq
......................................................................uaagcaccAuuggauugccccgaa..................   111    1   seq
......................................................................uaagcaccguuggauugccccgaa..................   2124   0   seq
......................................................................uaagcacAguuggauugccccgaa..................   1      1   seq
......................................................................uaagcaccguuAgauugccccgaa..................   12     1   seq
......................................................................uaagcaccgCuggauugccccgaa..................   2      1   seq
......................................................................uaagcaccguuggauugccAcgaa..................   2      1   seq
......................................................................uaagcaccguuggauugccccUaa..................   2      1   seq
```

```
                                  Star                      Mature
caccguaauuuucggagauauucgucucaguuucggggcuaucgauugguguuuguguugcaaaugaucauaagcaccguuggauugccccgaaauuggaaucugcuaucau
......................................................................uaagcaccguuggauugccccgaU..................	93	1	seq
......................................................................uaagcaccguuggauugccccgaG..................	6	1	seq
......................................................................Aaagcaccguuggauugccccgaa..................	11	1	seq
......................................................................uaagcaccgAuggauugccccgaa..................	1	1	seq
......................................................................uaagcaccguuggauugccccgUa..................	1	1	seq
......................................................................uaagcaccguuggauugccccCaa..................	58	1	seq
......................................................................uGagcaccguuggauugccccgaa..................	3	1	seq
......................................................................uaagcacUguuggauugccccgaa..................	1	1	seq
......................................................................uaagcacGguuggauugccccgaa..................	1	1	seq
......................................................................uaagcaccguuggauAgccccgaa..................	1	1	seq
......................................................................uaagcaccguuggaAugccccgaa..................	1	1	seq
......................................................................uaagcaccguAggauugccccgaa..................	1	1	seq
......................................................................uaGgcaccguuggauugccccgaa..................	1	1	seq
......................................................................uaagcaccguuggauugcUccgaa..................	2	1	seq
......................................................................uaagcaccguuggauugcccUgaa..................	1	1	seq
......................................................................uaagcaccguuggauugcAccgaa..................	1	1	seq
......................................................................uaagcaccguuggauugcAccgaaa.................	2	1	seq
......................................................................uaagcCccguuggauugccccgaaa.................	1	1	seq
......................................................................uaagcaccguuggauugccccgaaa.................	5603	0	seq
......................................................................uaagcaccguuggauugccccgaUa.................	4	1	seq
......................................................................uaagcaccguuggauugccccgaCa.................	9	1	seq
......................................................................Aaagcaccguuggauugccccgaaa.................	31	1	seq
......................................................................uaagcaccguuggauugccccCaaa.................	24	1	seq
......................................................................uaagcaccguuggauugcccAgaaa.................	2	1	seq
......................................................................uaagAaccguuggauugccccgaaa.................	1	1	seq
......................................................................uaagcaccguuggGuugccccgaaa.................	1	1	seq
......................................................................uaagcUccguuggauugccccgaaa.................	1	1	seq
......................................................................uaaAcaccguuggauugccccgaaa.................	2	1	seq
......................................................................uaagcaccguGggauugccccgaaa.................	1	1	seq
......................................................................uaagcaccgAuggauugccccgaaa.................	3	1	seq
......................................................................uaagcaccguuggauugcccUgaaa.................	8	1	seq
......................................................................Caagcaccguuggauugccccgaaa.................	3	1	seq
......................................................................uaagcaccguugUauugccccgaaa.................	2	1	seq
......................................................................uaagcaccguuggauCgccccgaaa.................	4	1	seq
......................................................................uaagcaccguuggauugccGcgaaa.................	1	1	seq
......................................................................uaagcaccguuggauugccccgCaa.................	1	1	seq
......................................................................uaagcaccguuggauugGcccgaaa.................	1	1	seq
......................................................................uaagcaccguuggauugccccgaaU.................	14	1	seq
......................................................................uaagcaccguuAgauugccccgaaa.................	22	1	seq
......................................................................uaagcaAcguuggauugccccgaaa.................	2	1	seq
......................................................................uaagcacUguuggauugccccgaaa.................	3	1	seq
......................................................................Gaagcaccguuggauugccccgaaa.................	5	1	seq
......................................................................uaagcaccguuggauugccUcgaaa.................	4	1	seq
......................................................................uaagcaccAuuggauugccccgaaa.................	336	1	seq
......................................................................uaagcacAguuggauugccccgaaa.................	2	1	seq
......................................................................uaagcaccguuggauugccccgaaG.................	15	1	seq
......................................................................uaagcaccguuggauugccccUaaa.................	1	1	seq
......................................................................uaagcGccguuggauugccccgaaa.................	1	1	seq
......................................................................uaagcaccguuUgauugccccgaaa.................	2	1	seq
......................................................................uaagcaccguuggauugccccgaaC.................	34	1	seq
......................................................................uaagcaccguuggauuAccccgaaa.................	1	1	seq
......................................................................uaagcaccguuggauugccAcgaaa.................	3	1	seq
......................................................................Naagcaccguuggauugccccgaaa.................	1	1	seq
......................................................................uaagcaccguAggauugccccgaaa.................	4	1	seq
......................................................................uaagcaccguuggaAugccccgaaa.................	3	1	seq
......................................................................uaagcaccguuggauAgccccgaaa.................	2	1	seq
......................................................................uaagcaccAuuggauugccccgaaau................	1	1	seq
......................................................................uaagcaccguuggauugccccgaaaC................	9	1	seq
......................................................................uaagcaccguuggauugccccgaaaG................	9	1	seq
......................................................................uaagcaccguuggauugccccgaaaA................	2293	1	seq
......................................................................uaagcaccguuggauugccccgaaau................	12	0	seq
......................................................................uaagcaccguuggauugccccgaaaAu...............	5	1	seq
.......................................................................aagcaccguuggauuUccccg....................	1	1	seq
.......................................................................aagcaccguuggauugccccg....................	2	0	seq
.......................................................................aagcaccguuggaAugccccga...................	2	1	seq
.......................................................................aagcacAguuggauugccccga...................	1	1	seq
.......................................................................aagcaccguugCauugccccga...................	1	1	seq
.......................................................................aagcaccguuggauugcGccga...................	1	1	seq
.......................................................................Uagcaccguuggauugccccga...................	32	1	seq
.......................................................................aagcaccgGuggauugccccga...................	8	1	seq
```

Star Mature

```
caccguaauuuucggagauauucgucucaguuucggggcuaucgauugguguuuguguugcaaaugaucauaagcaccguuggauugccccgaaauuggaaucugcuaucau
.......................................................................aagcaccguuggauugccUcga...................  1    1  seq
.......................................................................aagcaccguuggauuUccccga...................  11   1  seq
.......................................................................aagcaccguuggauugccAcga...................  1    1  seq
.......................................................................aagcaccguuggauugAcccga...................  1    1  seq
.......................................................................aagcaccguuggauugcccAga...................  1    1  seq
.......................................................................aagcaccguuggauugccccCa...................  3    1  seq
.......................................................................aagcaccguuggUuugccccga...................  1    1  seq
.......................................................................aaAcaccguuggauugccccga...................  2    1  seq
.......................................................................aagcaccguuggauugcccUga...................  1    1  seq
.......................................................................aagcaccguuggauugccccUa...................  1    1  seq
.......................................................................aUgcaccguuggauugccccga...................  1    1  seq
.......................................................................aagcaccguuggauugccccga...................  544  0  seq
.......................................................................aagcaUcguuggauugccccga...................  1    1  seq
.......................................................................aagcaccguuAgauugccccga...................  2    1  seq
.......................................................................aagcaccguuggauugccccgC...................  1    1  seq
.......................................................................aagcaccguuggauugccccgG...................  4    1  seq
.......................................................................aagcaccguuggauugccccgaa..................  5    0  seq
.......................................................................Uagcaccguuggauugccccgaa..................  1    1  seq
.......................................................................aagcaccguuggauugccccgaU..................  1    1  seq
.......................................................................aagcaccAuuggauugccccgaa..................  1    1  seq
.......................................................................aagcaccguuggauugccccgaaa.................  4    0  seq
.......................................................................aagcaccAuuggauugccccgaaa.................  3    1  seq
.......................................................................aagcaccguuggauugccccgaaaA................  1    1  seq
........................................................................agcaccguuggauugccc......................  5    0  seq
........................................................................agcaccguuggauugccccU....................  1    1  seq
........................................................................agcaccguuggauugccccg....................  1    0  seq
........................................................................agcaccguuggaAugccccga...................  1    1  seq
........................................................................agcaccguuggauugccccgG...................  3    1  seq
........................................................................agcaccguuggauugccccga...................  545  0  seq
........................................................................Ggcaccguuggauugccccga...................  1    1  seq
........................................................................Ugcaccguuggauugccccga...................  1    1  seq
........................................................................agcaccguuggauugccAcga...................  1    1  seq
........................................................................agcaccguuUgauugccccga...................  1    1  seq
........................................................................agcaccguuggauugccccgaa..................  13   0  seq
........................................................................agcaccguuggauugccccgaU..................  20   1  seq
........................................................................agcaccguuggauugcccUgaa..................  1    1  seq
........................................................................agcaccguuAgauugccccgaa..................  2    1  seq
........................................................................agcaccAuuggauugccccgaa..................  2    1  seq
........................................................................agcaccguuggauugccccgaaU.................  1    1  seq
........................................................................agcaccguuggauugccccgaaa.................  4    0  seq
........................................................................agcaccguuggauugccccgaUa.................  1    1  seq
........................................................................agcaccAuuggauugccccgaaa.................  1    1  seq
........................................................................agcaccguuggauugccccgaaaA................  1    1  seq
.........................................................................gcaccguuggauugccccg....................  1    0  seq
.........................................................................gcaccgAuggauugccccga...................  1    1  seq
.........................................................................gcaccguuggauugccccga...................  18   0  seq
.........................................................................gcaccguuAgauugccccga...................  1    1  seq
.........................................................................gcaccAuuggauugccccgaa..................  1    1  seq
.........................................................................gcaccguuggauugccccgaaa.................  1    0  seq
.........................................................................gcaccguuggauugccccgaaaA................  1    1  seq
..........................................................................caccguuggauugccccga...................  11   0  seq
..........................................................................caccguuggauugccccgaaaA................  2    1  seq
...........................................................................accgAuggauugccccga...................  1    1  seq
...........................................................................accguuggauugccccga...................  19   0  seq
...........................................................................accguuggauugccccgaaa.................  2    0  seq
```
