# Supplementary material for: The Small RNA Universe of Capitella teleta
Source: Front Mol Biosci. 2022 Feb 25;9:802814. doi: 10.3389/fmolb.2022.802814 (PMC8915122; doi:10.3389/fmolb.2022.802814)
Supplement: Supplementary file 1 [file DataSheet1.ZIP › Supplement/confident/CAPTEscaffold_60_5461.pdf]

Provisional ID : CAPTEscaffold_60_5461
Score total : 809.5
Score for star read(s) : 3.9
Score for read counts : 803.6
Score for mfe : 1
Score for randfold : 1.6
Score for cons. seed : -0.6
Total read count : 1588
Mature read count : 1378
Loop read count : 0
Star read count : 210

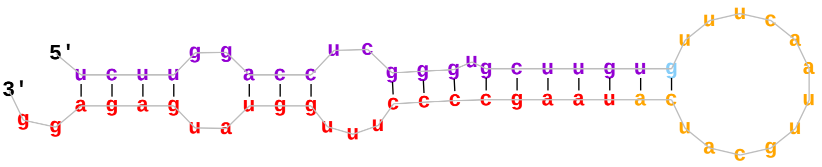

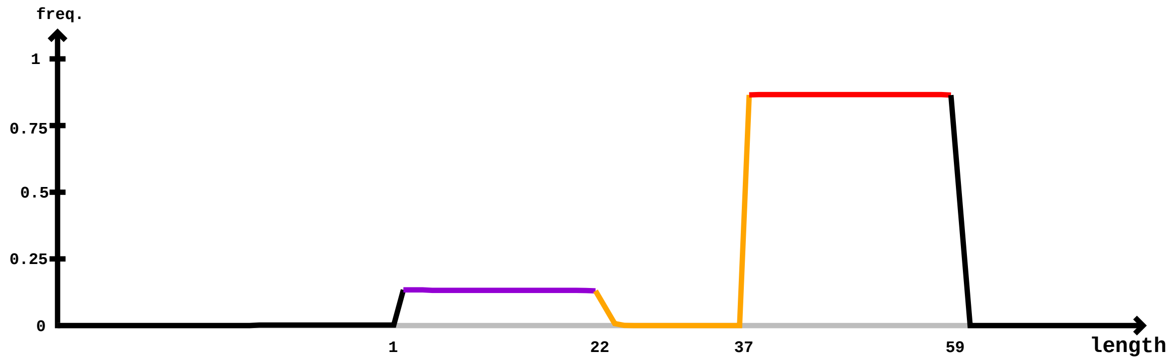

Star Mature

```
5'- gcaucaagaauuauguuuaguaugauuggcucuuucuuggaccucgggugcuuguguuucaauugcaucauaagccccuuugguaugagagggaacugaugcucagucugc   -3'   obs
    gcaucaagaauuauguuuaguaugauuggcucuuucuuggaccucgggugcuuguguuucaauugcaucauaagccccuuugguaugagagggaacugaugcucagucugc          exp
    ((((((..(((((((.....)))))))...((((((((..(((..(((.(((((((...........))))))))))...)))..))))))))..))))))..........   reads    mm    sample
    ...................guaugauuggcucuuucu..........................................................................   2        0     seq
    ...................gCaugauuggcucuuucu..........................................................................   1        1     seq
    ..................................ucuuggaccucgggugcuu..........................................................   1        0     seq
    ..................................ucuuggaccucgggugcuug.........................................................   2        0     seq
    ..................................ucuuggaccucgggugcuugu........................................................   145      0     seq
    ..................................ucuuAgaccucgggugcuugu........................................................   2        1     seq
    ..................................ucuuggaccucgggugcuugA........................................................   2        1     seq
    ..................................ucuuggaccucgggugcuuguU.......................................................   1        1     seq
    ..................................ucuuggaccucgggugcuugug.......................................................   41       0     seq
    ..................................ucuuggaGcucgggugcuugug.......................................................   1        1     seq
    ..................................Acuuggaccucgggugcuugug.......................................................   1        1     seq
    ..................................ucuuggaccucgggugcuuguA.......................................................   2        1     seq
    ..................................ucuuggaccucgggugcuugugA......................................................   7        1     seq
    ..................................ucuuggaccucgggugcuugugG......................................................   2        1     seq
    ..................................ucuuggaccucgggugcuugugu......................................................   2        0     seq
    ..................................ucuuggaccucgggugcuugugAu.....................................................   1        1     seq
    ......................................................................uaagccccuuugguaugagag....................   3        0     seq
    ......................................................................uaagccccuuGgguaugagagg...................   1        1     seq
    ......................................................................Aaagccccuuugguaugagagg...................   7        1     seq
    ......................................................................uGagccccuuugguaugagagg...................   1        1     seq
    ......................................................................uaagccccuuugguaCgagagg...................   1        1     seq
    ......................................................................uaagccAcuuugguaugagagg...................   3        1     seq
    ......................................................................uaagccccGuugguaugagagg...................   1        1     seq
    ......................................................................uaagccccuuugguaugagagU...................   2        1     seq
    ......................................................................uaagccccuuugguaugagUgg...................   1        1     seq
    ......................................................................uaagcccUuuugguaugagagg...................   2        1     seq
    ......................................................................uaagccccuuuggGaugagagg...................   1        1     seq
    ......................................................................uaagccccuuugguaugagagA...................   1        1     seq
    ......................................................................Gaagccccuuugguaugagagg...................   1        1     seq
    ......................................................................uaagccccuuugguauAagagg...................   3        1     seq
    ......................................................................uaagccccuuAgguaugagagg...................   1        1     seq
    ......................................................................uaagccccuuugguaugagaAg...................   13       1     seq
    ......................................................................uaagccccuAugguaugagagg...................   1        1     seq
```

```
                                  Star                                Mature
gcaucaagaauuauguuuaguaugauuggcucuuucuuggaccucgggugcuuguguuucaauugcaucauaagccccuuugguaugagagggaacugaugcucagucugc
......................................................................uaagccccuuuAguaugagagg...................   7      1      seq
......................................................................uaagccccuuugguaugaUagg...................   1      1      seq
......................................................................uaagccccuuugguaugagagg...................   1321   0      seq
......................................................................uaagccccuuugguaugagagC...................   2      1      seq
......................................................................uaagccccuuugguaugagaggA..................   2      1      seq
.......................................................................aagccccuuugguaugagagg...................   1      0      seq
.......................................................................aagccccGuugguaugagagg...................   1      1      seq
```
