# Supplementary material for: The Small RNA Universe of Capitella teleta
Source: Front Mol Biosci. 2022 Feb 25;9:802814. doi: 10.3389/fmolb.2022.802814 (PMC8915122; doi:10.3389/fmolb.2022.802814)
Supplement: Supplementary file 1 [file DataSheet1.ZIP › Supplement/confident/CAPTEscaffold_15480_45256.pdf]

Provisional ID	:	CAPTEscaffold_15480_45256
Score total	:	3813.8
Score for star read(s)	:	3.9
Score for read counts	:	3807.5
Score for mfe	:	1.4
Score for randfold	:	1.6
Score for cons. seed	:	-0.6
Total read count	:	7480
Mature read count	:	3847
Loop read count	:	0
Star read count	:	3633

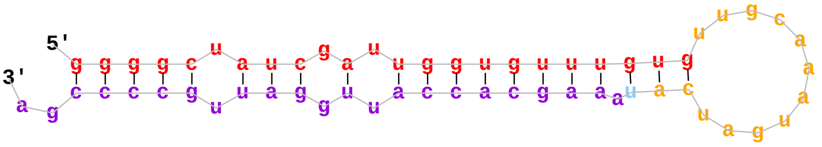

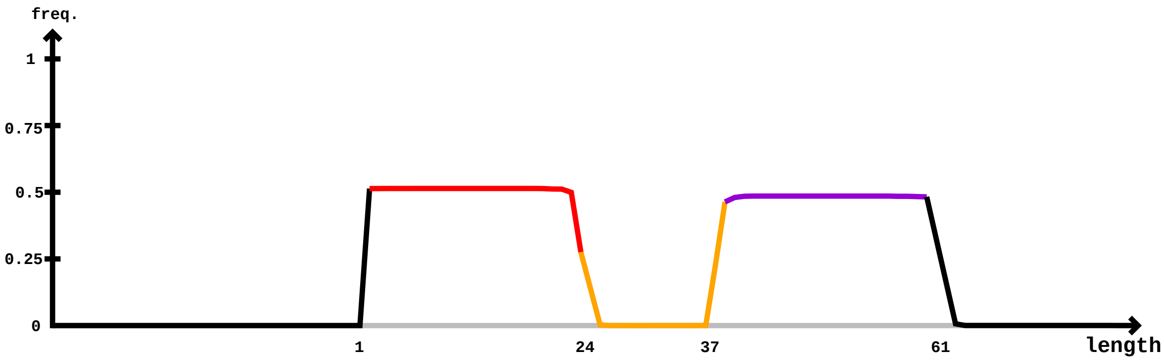

Mature **Star**

```
5'- ccuaauuuucggagauauuugucucaguuuuggggcuaucgauugguguuuguguugcaaaugaucauaaagcaccauuggauugccccgacauuggaaucugcuaucauu  -3'  obs
    ccuaauuuucggagauauuugucucaguuuuggggcuaucgauugguguuuguguugcaaaugaucauaaagcaccauuggauugccccgacauuggaaucugcuaucauu       exp
    ..........(.((((.....((.((((.(((((((.(((.(.(((((((((((...........))).)))))))).).))).))))))).)))))))))).).......   reads    mm    sample
    ...............................ggggcuaucgauuggugu..............................................................   2        0     seq
    ...............................ggggcuaucgauugguguu.............................................................   13       0     seq
    ...............................ggggcuaucgauugguguuu............................................................   3        0     seq
    ...............................ggggcuaucgauAgguguuug...........................................................   1        1     seq
    ...............................ggggcuaucgauugguguuug...........................................................   89       0     seq
    ...............................Agggcuaucgauugguguuug...........................................................   1        1     seq
    ...............................ggggcuaucgauugguguuuA...........................................................   1        1     seq
    ...............................ggggcuaucgauAgguguuugu..........................................................   1        1     seq
    ...............................ggggcuaucgauugguguuugA..........................................................   1        1     seq
    ...............................ggggcuaucgauuggugAuugu..........................................................   2        1     seq
    ...............................ggggGuaucgauugguguuugu..........................................................   1        1     seq
    ...............................ggggcuaucgauugguguuugG..........................................................   1        1     seq
    ...............................ggggcuauUgauugguguuugu..........................................................   4        1     seq
    ...............................ggggcuaucgauugguguuAgu..........................................................   1        1     seq
    ...............................ggggcuaucAauugguguuugu..........................................................   5        1     seq
    ...............................ggggcuaucgauugguguuCgu..........................................................   1        1     seq
    ...............................ggggcuaucgauugguguuuCu..........................................................   1        1     seq
    ...............................ggggcuauAgauugguguuugu..........................................................   1        1     seq
    ...............................Ngggcuaucgauugguguuugu..........................................................   1        1     seq
    ...............................Agggcuaucgauugguguuugu..........................................................   8        1     seq
    ...............................ggggcuaucgauuAguguuugu..........................................................   5        1     seq
    ...............................ggggcuaucgauugguguuugu..........................................................   1645     0     seq
    ...............................ggggcuaucgauuggAguuugu..........................................................   1        1     seq
    ...............................ggggcuaucgauugguguuugug.........................................................   1944     0     seq
    ...............................ggggcuaucgaAugguguuugug.........................................................   1        1     seq
    ...............................Ugggcuaucgauugguguuugug.........................................................   2        1     seq
    ...............................ggggcuaucCauugguguuugug.........................................................   1        1     seq
    ...............................ggggcuaucAauugguguuugug.........................................................   10       1     seq
    ...............................ggggcuaucgauugguAuuugug.........................................................   1        1     seq
    ...............................ggCgcuaucgauugguguuugug.........................................................   1        1     seq
    ...............................gAggcuaucgauugguguuugug.........................................................   5        1     seq
    ...............................ggggcuaucgauugguguuCgug.........................................................   1        1     seq
    ...............................ggggcuauAgauugguguuugug.........................................................   1        1     seq
```

```
                               Mature                               Star
ccuaauuuucggagauauuugucucaguuuuggggcuaucgauugguguuuguguugcaaaugaucauaaagcaccauuggauugccccgacauuggaaucugcuaucauu
...............................ggggcuaucgauugguguuuguA.........................................................  4     1  seq
...............................ggggcuaucgauuggAguuugug.........................................................  3     1  seq
...............................ggggcuaucgauugUuguuugug.........................................................  1     1  seq
...............................Agggcuaucgauugguguuugug.........................................................  14    1  seq
...............................ggggcuaucgauugguguAugug.........................................................  1     1  seq
...............................ggggcuaucgauugguguuuguU.........................................................  4     1  seq
...............................ggAgcuaucgauugguguuugug.........................................................  2     1  seq
...............................ggggcuaucgauuAguguuugug.........................................................  6     1  seq
...............................ggggcuauUgauugguguuugug.........................................................  6     1  seq
...............................ggggcuaucgauugguguuuAug.........................................................  1     1  seq
...............................ggggcuaucgauugguguuAgug.........................................................  1     1  seq
...............................ggggcuaucgauugguguCugug.........................................................  1     1  seq
...............................ggggcuaucgauugguguuugugC........................................................  1     1  seq
...............................ggggcuaucgauugguguuugugA........................................................  6     1  seq
...............................ggggcuaucgauugguguuugugG........................................................  2     1  seq
...............................ggggcuaucgauugguguuugugu........................................................  17    0  seq
...............................ggggcuaucgauugguguuuguguA.......................................................  8     1  seq
...............................ggggcuaucgauugguguuuguguu.......................................................  8     0  seq
...............................ggggcuaucgauugguguuuguguugc.....................................................  1     0  seq
................................gggcuaucgauugguguuugug.........................................................  1     0  seq
................................gggcuaucgauuAguguuugug.........................................................  1     1  seq
.................................ggcuaucgauugguguuugu..........................................................  1     0  seq
....................................uaucgauugguguuugug.........................................................  1     0  seq
.............................................................ugaucauaaagcaccauuggauugccccga....................  1     0  seq
..................................................................auaaagcaccauuggauugccccgaU...................  1     1  seq
...................................................................uaaagcaccauuggauugc.........................  6     0  seq
...................................................................uaaagcaccauuggauugccc.......................  5     0  seq
...................................................................uaaagcaccauAggauugccc.......................  1     1  seq
...................................................................uaaagcaccauuggauugcccc......................  6     0  seq
...................................................................uaaagcaccauuggauugccccg.....................  20    0  seq
...................................................................uaaagcaccauuggauugccccAa....................  3     1  seq
...................................................................uaaagcaccauuggauugccccgC....................  8     1  seq
...................................................................uaaagcaccauuggauAgccccga....................  1     1  seq
...................................................................uaaagcaccauuggauugccAcga....................  1     1  seq
...................................................................uaaagcacAauuggauugccccga....................  4     1  seq
...................................................................uaaagcaccauuAgauugccccga....................  5     1  seq
...................................................................Aaaagcaccauuggauugccccga....................  7     1  seq
...................................................................uaaagcaccUuuggauugccccga....................  1     1  seq
...................................................................uaaagcaccaGuggauugccccga....................  1     1  seq
...................................................................uaaagcGccauuggauugccccga....................  1     1  seq
...................................................................uaaagcaccauuggauugcccAga....................  1     1  seq
...................................................................uaaagcaccauuggauugccUcga....................  2     1  seq
...................................................................uaaagcaccauuggauugAcccga....................  1     1  seq
...................................................................uaaagcaccauuggauugccccgG....................  7     1  seq
...................................................................uaaagcaccauuggauugccccgU....................  4     1  seq
...................................................................uaaagcacUauuggauugccccga....................  1     1  seq
...................................................................uaaagcaccauuggaAugccccga....................  1     1  seq
...................................................................uaaagcaccauAggauugccccga....................  2     1  seq
...................................................................uaaagcaccauuggauugcccUga....................  1     1  seq
...................................................................uaaagcaccauuggauugccccga....................  1534  0  seq
...................................................................uaaagcaccauuggauugccccCa....................  2     1  seq
...................................................................uaaagcaccaAuggauugccccga....................  1     1  seq
...................................................................uaaagcaccGuuggauugccccga....................  5     1  seq
...................................................................uaaagcaccauuggauugccccgaA...................  13    1  seq
...................................................................uaaagcaccauuggauugccccgCc...................  1     1  seq
...................................................................uaaagcaccauuggauugccccgaAa..................  39    1  seq
....................................................................aaagcaccauuggauugc.........................  1     0  seq
....................................................................aaagcaccGuuggauugccc.......................  2     1  seq
....................................................................aaagcaccGuuggauugcccc......................  2     1  seq
....................................................................aaagcaccauuggauugccccg.....................  1     0  seq
....................................................................aaagcaccGuuggauugccccg.....................  2     1  seq
....................................................................Naagcaccauuggauugccccga....................  2     1  seq
....................................................................aaagcaccauuggauugccccga....................  84    0  seq
....................................................................Caagcaccauuggauugccccga....................  2     1  seq
....................................................................aaagcaccUuuggauugccccga....................  1     1  seq
....................................................................aaagcaccauuAgauugccccga....................  1     1  seq
....................................................................aaagcaccGuuggauugccccga....................  1674  1  seq
....................................................................aaagcaccauuggauugccccgU....................  1     1  seq
....................................................................Gaagcaccauuggauugccccga....................  4     1  seq
....................................................................aaagcaccauuggauAgccccga....................  1     1  seq
```

```
                                Mature                                Star
ccuaauuuucggagauauuugucucaguuuuggggcuaucgauugguguuuguguugcaaaugaucauaaagcaccauuggauugccccgacauuggaaucugcuaucauu
...................................................................aaagcaccauuggauugccccgaAa...................	4	1	seq
....................................................................aagcaccauuggauugccccg......................	1	0	seq
....................................................................aagcaccauuggauugcccUga.....................	1	1	seq
....................................................................aagcaccauAggauugccccga.....................	2	1	seq
....................................................................aagcaccauuggauugccccga.....................	112	0	seq
....................................................................Uagcaccauuggauugccccga.....................	1	1	seq
....................................................................aagcaccauuggauugccccAa.....................	3	1	seq
....................................................................aagcaccauuggauugccccgaA....................	1	1	seq
....................................................................aagcaccauuggauugccccgaAa...................	3	1	seq
.....................................................................agcaccauuggauugccccg......................	1	0	seq
.....................................................................Ggcaccauuggauugccccga.....................	1	1	seq
.....................................................................agcaccauuggauugccccga.....................	29	0	seq
.....................................................................agcaccauuggauugccccgaA....................	2	1	seq
.....................................................................agcaccauuggauugccccgaU....................	1	1	seq
.....................................................................agcaccauuggauugccccgaAa...................	1	1	seq
......................................................................gcaccauuggauugccccgaU....................	5	1	seq
......................................................................gcaccauuggauugccccgaA....................	1	1	seq
...........................................................................................cauuggaaucugcuauca..	1	0	seq
...........................................................................................cauuggaaucugcuaucauu	1	0	seq
```
