# Supplementary material for: The Small RNA Universe of Capitella teleta
Source: Front Mol Biosci. 2022 Feb 25;9:802814. doi: 10.3389/fmolb.2022.802814 (PMC8915122; doi:10.3389/fmolb.2022.802814)
Supplement: Supplementary file 1 [file DataSheet1.ZIP › Supplement/confident/CAPTEscaffold_488_22711.pdf]

Provisional ID	:	CAPTEscaffold_488_22711
Score total	:	58220.2
Score for star read(s)	:	3.9
Score for read counts	:	58213.5
Score for mfe	:	1.8
Score for randfold	:	1.6
Score for cons. seed	:	-0.6
Total read count	:	114195
Mature read count	:	114181
Loop read count	:	0
Star read count	:	14

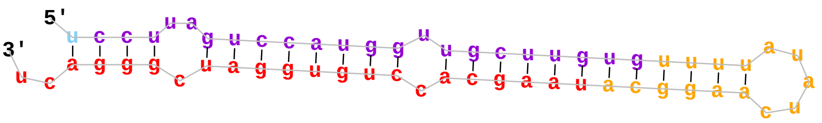

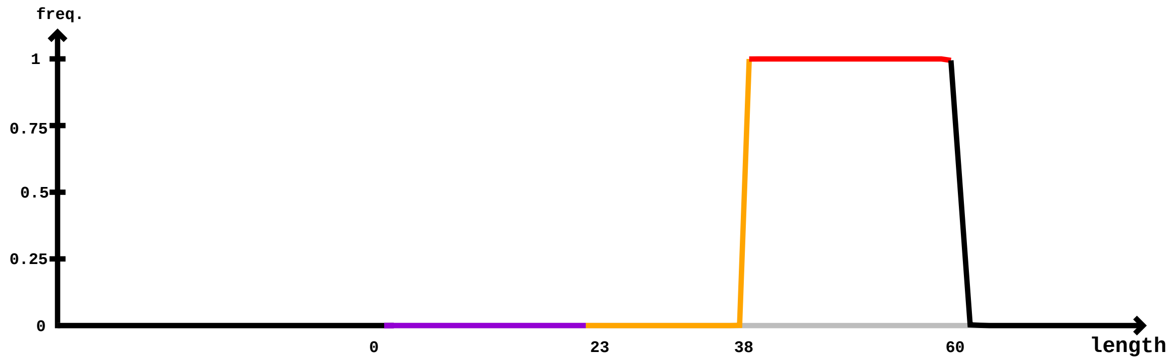

Star Mature

```
5'- ugauguucauaauucugguuaggacuggcuuuuccuuaguccaugguugcuuguguuuuauaucaaggcauaagcaccuguggaucgggacuaagcuguuccuugagcuac  -3'  obs
    ugauguucauaauucugguuaggacuggcuuuuccuuaguccaugguugcuuguguuuuauaucaaggcauaagcaccuguggaucgggacuaagcuguuccuugagcuac        exp
    ....(((((...........((((.((((((.((((..((((((((.((((((((((((.....)))))))))))).)))))))).))))..)))))).)))))))))...  reads  mm  sample
    ............uucugguuaggacuggcuuuu..............................................................................  2  0  seq
    .............ucugguuaggacuggcuuuu..............................................................................  2  0  seq
    .................................ccuuaguccGugguugcuugu.........................................................  1  1  seq
    .................................ccuuagucUaugguugcuugug........................................................  1  1  seq
    .................................ccuuaguccGugguugcuugug........................................................  3  1  seq
    .................................ccuuaguccaugguugcuugug........................................................  4  0  seq
    .................................ccuuaguccaugguugcuugugu.......................................................  2  0  seq
    ..................................cuuaguccaugguugcuugug........................................................  1  0  seq
    ..................................cuuaguccaugguugcuugugu.......................................................  2  0  seq
    .....................................................................auaagcaccuguggaucgggac....................  46  0  seq
    .....................................................................auaagcaccuguggaucgggacu...................  16  0  seq
    .....................................................................Cuaagcaccuguggaucgggacu...................  3  1  seq
    .....................................................................auaagcaccuguggaucgggacA...................  5  1  seq
    .....................................................................auaagcaccuguggaucgAgacua..................  4  1  seq
    .....................................................................auaagcaccuguggaucgggacAa..................  2  1  seq
    .....................................................................auaagcaccuguggaucgggacAaa.................  1  1  seq
    ......................................................................uaagcaccuguggGucggg......................  1  1  seq
    ......................................................................uaagcaccuguggaucggga.....................  3  0  seq
    ......................................................................uaagcaccuguggauAgggac....................  1  1  seq
    ......................................................................uaagcaccuguggauUgggac....................  1  1  seq
    ......................................................................uaagcaccuguggaucgggaU....................  3  1  seq
    ......................................................................uaagcaccuguAgaucgggac....................  1  1  seq
    ......................................................................Gaagcaccuguggaucgggac....................  1  1  seq
    ......................................................................uaagcaccuguggaucgggCc....................  1  1  seq
    ......................................................................Aaagcaccuguggaucgggac....................  2  1  seq
    ......................................................................uaagcaccugAggaucgggac....................  1  1  seq
    ......................................................................uaagcUccuguggaucgggac....................  1  1  seq
    ......................................................................uaagcaccuguggaucgggac....................  553  0  seq
    ......................................................................uaGgcaccuguggaucgggacu...................  20  1  seq
    ......................................................................uaagcaccugugCaucgggacu...................  12  1  seq
    ......................................................................uaagcaccuguggauAgggacu...................  33  1  seq
    ......................................................................uaaAcaccuguggaucgggacu...................  16  1  seq
    ......................................................................uaagcaccuguggaucCggacu...................  9  1  seq
```

```
                                Star                               Mature
ugauguucauaauucugguuaggacuggcuuuuccuuaguccaugguugcuuguguuuuauaucaaggcauaagcaccuguggaucgggacuaagcuguuccuugagcuac
......................................................................uaagcaccuguggGucgggacu...................    18      1    seq
......................................................................uaagcaccuguggaucgCgacu...................    4       1    seq
......................................................................uaagcaccugCggaucgggacu...................    30      1    seq
......................................................................uaagcacGuguggaucgggacu...................    3       1    seq
......................................................................uaUgcaccuguggaucgggacu...................    6       1    seq
......................................................................uaagcUccuguggaucgggacu...................    18      1    seq
......................................................................uaagcaccuguggaucgggaGu...................    9       1    seq
......................................................................uaagcGccuguggaucgggacu...................    17      1    seq
......................................................................uaagcaccuguUgaucgggacu...................    15      1    seq
......................................................................uaagcaccuguggauGgggacu...................    6       1    seq
......................................................................uaagcaccuguggauUgggacu...................    209     1    seq
......................................................................uaagcaccuguggaucAggacu...................    243     1    seq
......................................................................uaagcaccuguggaGcgggacu...................    4       1    seq
......................................................................uaaUcaccuguggaucgggacu...................    9       1    seq
......................................................................uNagcaccuguggaucgggacu...................    4       1    seq
......................................................................uaagcaccuguggaucgUgacu...................    17      1    seq
......................................................................uaagAaccuguggaucgggacu...................    13      1    seq
......................................................................uaagcaccuCuggaucgggacu...................    19      1    seq
......................................................................uaagcaccuguggaucgggacC...................    22      1    seq
......................................................................uaagcaccuguggaAcgggacu...................    28      1    seq
......................................................................uaagcaccugGggaucgggacu...................    7       1    seq
......................................................................uaagcCccuguggaucgggacu...................    5       1    seq
......................................................................uaagGaccuguggaucgggacu...................    8       1    seq
......................................................................uaCgcaccuguggaucgggacu...................    1       1    seq
......................................................................Naagcaccuguggaucgggacu...................    23      1    seq
......................................................................uaagcaccuguggaucgggUcu...................    14      1    seq
......................................................................uaagcaccuguggaucggAacu...................    16      1    seq
......................................................................uaagcaccuguggaucgggGcu...................    23      1    seq
......................................................................uaagcaccCguggaucgggacu...................    18      1    seq
......................................................................uaagcaccugugAaucgggacu...................    20      1    seq
......................................................................uaagcaccuAuggaucgggacu...................    19      1    seq
......................................................................Gaagcaccuguggaucgggacu...................    55      1    seq
......................................................................uaagcaccuguggUucgggacu...................    15      1    seq
......................................................................Aaagcaccuguggaucgggacu...................    511     1    seq
......................................................................uaagcaccuguggaucgggacu...................    109540  0    seq
......................................................................uaagcNccuguggaucgggacu...................    2       1    seq
......................................................................uaagcaccuguggaucgggCcu...................    4       1    seq
......................................................................uaagcaccuguggaucgggaAu...................    18      1    seq
......................................................................uaagcacAuguggaucgggacu...................    50      1    seq
......................................................................uaagcaUcuguggaucgggacu...................    9       1    seq
......................................................................uaagcaccuguggaucgggacA...................    48      1    seq
......................................................................uaagcaccuguggaucgggacG...................    63      1    seq
......................................................................Caagcaccuguggaucgggacu...................    17      1    seq
......................................................................uaagcacUuguggaucgggacu...................    34      1    seq
......................................................................uaagcaccuguAgaucgggacu...................    325     1    seq
......................................................................uaagUaccuguggaucgggacu...................    9       1    seq
......................................................................uaagcaAcuguggaucgggacu...................    90      1    seq
......................................................................uaagcaccuUuggaucgggacu...................    10      1    seq
......................................................................uaagcaGcuguggaucgggacu...................    14      1    seq
......................................................................uaagcaccugAggaucgggacu...................    92      1    seq
......................................................................uUagcaccuguggaucgggacu...................    2       1    seq
......................................................................uaagcaccuguggaucUggacu...................    16      1    seq
......................................................................uaaCcaccuguggaucgggacu...................    4       1    seq
......................................................................uaagcaccuguggaCcgggacu...................    10      1    seq
......................................................................uaagcaccugugUaucgggacu...................    9       1    seq
......................................................................uaagcaccuguggaucggUacu...................    8       1    seq
......................................................................uaagcaccAguggaucgggacu...................    35      1    seq
......................................................................uaagcaccuguCgaucgggacu...................    6       1    seq
......................................................................uGagcaccuguggaucgggacu...................    55      1    seq
......................................................................uaagcaccuguggaucggCacu...................    4       1    seq
......................................................................uaagcaccGguggaucgggacu...................    16      1    seq
......................................................................uaagcaccuguggaucgggaUu...................    13      1    seq
......................................................................uaagcaccuguggaucgggacuU..................    17      1    seq
......................................................................uaagcaccuguggaucAggacua..................    1       1    seq
......................................................................Aaagcaccuguggaucgggacua..................    2       1    seq
......................................................................uaagcaccuguggaAcgggacua..................    1       1    seq
......................................................................uaagcaccuguggaucgAgacua..................    981     1    seq
......................................................................uaagcaccuguAgaucgggacua..................    1       1    seq
......................................................................uaagcaccuguggaucgggacua..................    156     0    seq
......................................................................uaagcaccuguggaucgggacAa..................    3       1    seq
```

```
                                Star                                  Mature
ugauguucauaauucugguuaggacuggcuuuuccuuaguccaugguugcuuguguuuuauaucaaggcauaagcaccuguggaucgggacuaagcuguuccuugagcuac
......................................................................uaagcaccuguggaucgggacuC..................    1     1     seq
......................................................................Gaagcaccuguggaucgggacuaa.................    1     1     seq
......................................................................uaagcaccuguggaucgggacuaU.................    1     1     seq
......................................................................uaagcaccuguggaucgggacuUa.................    1     1     seq
......................................................................uaagcaccuguggaucgggacuCa.................    1     1     seq
......................................................................uaagcaccuguggaucgUgacuaa.................    1     1     seq
......................................................................uaagcaccuguggaucgggacuaC.................    3     1     seq
......................................................................uaagcaccuguggaucgggacuaa.................    97    0     seq
......................................................................uaagcaccuguggaucggUacuaa.................    1     1     seq
......................................................................uaagcaccuguggaucgAgacuaa.................    94    1     seq
......................................................................uaagcaccuguggaucgggacuaag................    1     0     seq
......................................................................uaagcaccuguggaucgggacuaaC................    5     1     seq
......................................................................uaagcaccuguggaucgggacuaaA................    102   1     seq
......................................................................uaagcaccuguggaucgggacuaaAc...............    5     1     seq
.......................................................................Uagcaccuguggaucgggacu...................    15    1     seq
.......................................................................aagcaccuguggaucgggacu...................    14    0     seq
........................................................................agcaccuguggaucgggacu...................    2     0     seq
.........................................................................gcaccuguggaucgggaU....................    1     1     seq
.........................................................................gcaccuguggaucgggacu...................    1     0     seq
..........................................................................caccugCggaucgggacu...................    1     1     seq
..........................................................................caccuguggaucgggacu...................    2     0     seq
..........................................................................caccGguggaucgggacu...................    1     1     seq
```
