# Supplementary material for: The Small RNA Universe of Capitella teleta
Source: Front Mol Biosci. 2022 Feb 25;9:802814. doi: 10.3389/fmolb.2022.802814 (PMC8915122; doi:10.3389/fmolb.2022.802814)
Supplement: Supplementary file 1 [file DataSheet1.ZIP › Supplement/confident/CAPTEscaffold_60_5413.pdf]

Provisional ID	:	CAPTEscaffold_60_5413
Score total	:	375
Score for star read(s)	:	3.9
Score for read counts	:	373.3
Score for mfe	:	0.6
Score for randfold	:	-2.2
Score for cons. seed	:	-0.6
Total read count	:	744
Mature read count	:	534
Loop read count	:	0
Star read count	:	210

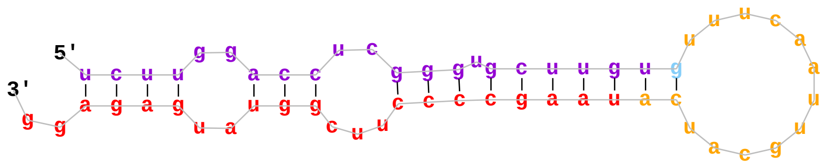

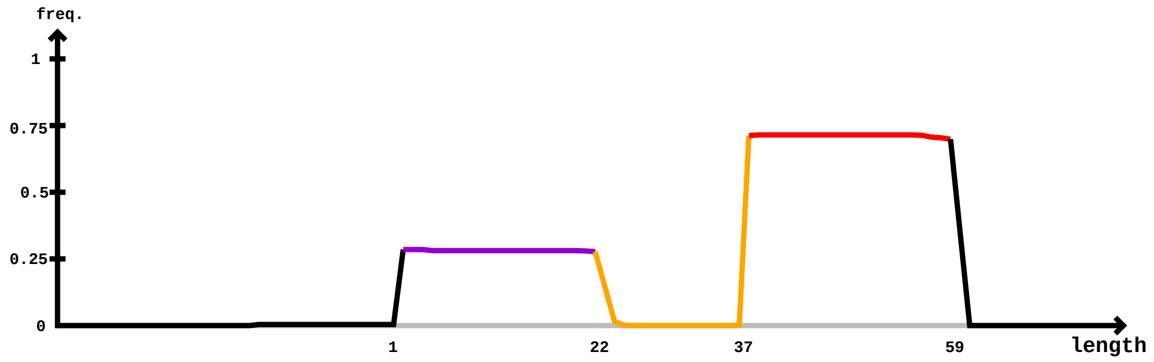


```
                                Star                               Mature
5'- gaaucaagaauuauguuuaguaugauuggcucuuucuuggaccucgggugcuuguguuucaauugcaucauaagccccuucgguaugagagggaacugaugcuuggucu   -3'   obs
    gaaucaagaauuauguuuaguaugauuggcucuuucuuggaccucgggugcuuguguuucaauugcaucauaagccccuucgguaugagagggaacugaugcuuggucu         exp
    ..((((((.((((....))))...(((((.((((((((..(((..(((.(((((((............))))))))))...)))..)))))))).))))).))))))..   reads   mm   sample
    ...................guaugauuggcucuuucu........................................................................   2       0    seq
    ...................gCaugauuggcucuuucu........................................................................   1       1    seq
    ..................................ucuuggaccucgggugcuu........................................................   1       0    seq
    ..................................ucuuggaccucgggugcuug.......................................................   2       0    seq
    ..................................ucuuggaccucgggugcuugA......................................................   2       1    seq
    ..................................ucuuggaccucgggugcuugu......................................................   145     0    seq
    ..................................ucuuAgaccucgggugcuugu......................................................   2       1    seq
    ..................................Acuuggaccucgggugcuugug.....................................................   1       1    seq
    ..................................ucuuggaccucgggugcuuguU.....................................................   1       1    seq
    ..................................ucuuggaccucgggugcuugug.....................................................   41      0    seq
    ..................................ucuuggaccucgggugcuuguA.....................................................   2       1    seq
    ..................................ucuuggaGcucgggugcuugug.....................................................   1       1    seq
    ..................................ucuuggaccucgggugcuugugu....................................................   2       0    seq
    ..................................ucuuggaccucgggugcuugugG....................................................   2       1    seq
    ..................................ucuuggaccucgggugcuugugA....................................................   7       1    seq
    ..................................ucuuggaccucgggugcuugugAu...................................................   1       1    seq
    .....................................................................Cuaagccccuucgguaugagagg.................   1       1    seq
    ......................................................................uaagccccuucgguauga.....................   1       0    seq
    ......................................................................uaagccccuucgguaugag....................   5       0    seq
    ......................................................................uaagccccuucgguaugagG...................   1       1    seq
    ......................................................................uaagccccuucgguaugaga...................   1       0    seq
    ......................................................................uaagccccuucgguaugagag..................   4       0    seq
    ......................................................................uaagccccuucgguaugagagC.................   18      1    seq
    ......................................................................uaagccccuuGgguaugagagg.................   1       1    seq
    ......................................................................Aaagccccuucgguaugagagg.................   2       1    seq
    ......................................................................uaagccccuucgguaugagagg.................   489     0    seq
    ......................................................................uaagccccuucgguGugagagg.................   1       1    seq
    ......................................................................Naagccccuucgguaugagagg.................   1       1    seq
    ......................................................................uaagccccuuAgguaugagagg.................   1       1    seq
    ......................................................................uaagccccuucgguaugagUgg.................   1       1    seq
    ......................................................................uaagcccUuucgguaugagagg.................   2       1    seq
    ......................................................................uaagccccuAcgguaugagagg.................   1       1    seq
    ......................................................................uaagccUcuucgguaugagagg.................   2       1    seq
```

```
                                  Star                                  Mature
gaaucaagaauuauguuuaguaugauuggcucuuucuuggaccucgggugcuuguguuucaauugcaucauaagccccuucgguaugagagggaacugaugcuuggucu
.......................................................................aagccccuucgguaugagagg.................    2    0    seq
```
