# Supplementary material for: The Small RNA Universe of Capitella teleta
Source: Front Mol Biosci. 2022 Feb 25;9:802814. doi: 10.3389/fmolb.2022.802814 (PMC8915122; doi:10.3389/fmolb.2022.802814)
Supplement: Supplementary file 1 [file DataSheet1.ZIP › Supplement/confident/CAPTEscaffold_324_18350.pdf]

Provisional ID	:	CAPTEscaffold_324_18350
Score total	:	172.8
Score for star read(s)	:	3.9
Score for read counts	:	168.9
Score for mfe	:	-0.9
Score for randfold	:	1.6
Score for cons. seed	:	-0.6
Total read count	:	343
Mature read count	:	221
Loop read count	:	0
Star read count	:	122

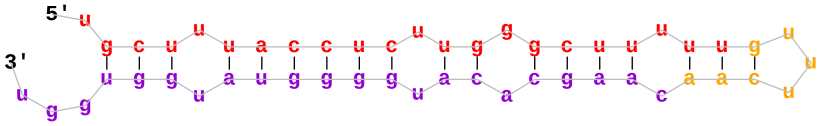

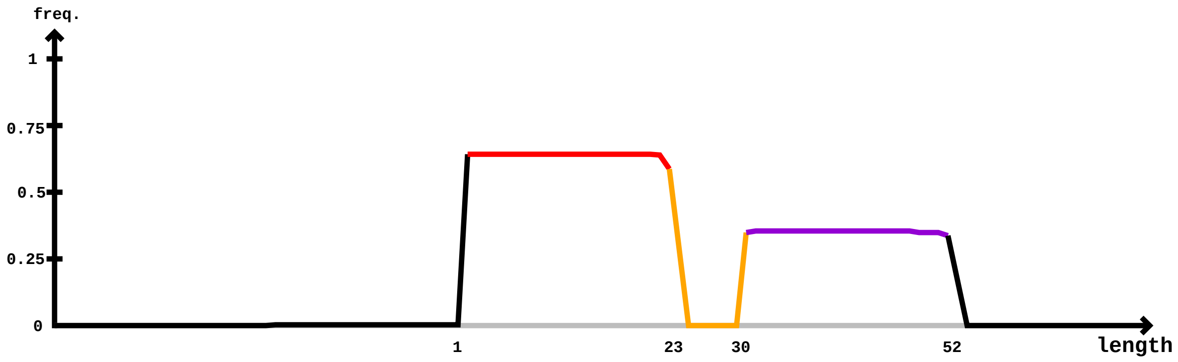

Mature **Star**

```
5'- uccacauuuuaagaugaccaacaauucugcuuggcuugugu ugcuuuaccucuugggcuuuuu guuucaa caagcacaugggguaugguggu guaugcccaagucuuugcaa   -3'    obs
    uccacauuuuaagaugaccaacaauucugcuuggcuuguguugcuuuaccucuugggcuuuuuguuucaacaagcacaugggguaugguggu guaugcccaagucuuugcaa         exp
    ....((((....))))..........(((..((((((.((((((.((((((.((.((((.(((...))).)))).)).)))))).))))......)).))))))...))).   reads    mm    sample
    .....................caauucugcuuggcuugugu.......................................................................   1        0     seq
    .........................................ugcuuuaccucuugggcuuu...................................................   1        0     seq
    .........................................ugcuuuaccucuugggcuuuu..................................................   18       0     seq
    .........................................ugcuuuaccucuuAggcuuuuu.................................................   2        1     seq
    .........................................ugcuuuaccucuugggUuuuuu.................................................   2        1     seq
    .........................................ugcuuuaccucuugggcuGuuu.................................................   12       1     seq
    .........................................ugcuuAaccucuugggcuuuuu.................................................   1        1     seq
    .........................................ugcuuuaccucuugggcuuuuu.................................................   150      0     seq
    .........................................ugcuuuaccucuugggcuuuuuU................................................   30       1     seq
    .........................................ugcuuuaccucuugggcuuuuuA................................................   5        1     seq
    ......................................................................caagcacaugggguaugg........................   2        0     seq
    ......................................................................caagcacaugggguauggugg.....................   4        0     seq
    ......................................................................Gaagcacaugggguaugguggu....................   2        1     seq
    ......................................................................Aaagcacaugggguaugguggu....................   1        1     seq
    ......................................................................caagcacaugggguaugguAgu....................   48       1     seq
    ......................................................................caagcacaugggguaugguggu....................   60       0     seq
    ......................................................................caagcacaugggguauggugguG...................   1        1     seq
    ......................................................................caagcacaugggguauggugguA...................   1        1     seq
    ......................................................................caagcacaugggguauggugguU...................   1        1     seq
    .......................................................................aagcacaugggguauggugguA...................   2        1     seq
```
