# Supplementary material for: The Small RNA Universe of Capitella teleta
Source: Front Mol Biosci. 2022 Feb 25;9:802814. doi: 10.3389/fmolb.2022.802814 (PMC8915122; doi:10.3389/fmolb.2022.802814)
Supplement: Supplementary file 1 [file DataSheet1.ZIP › Supplement/confident/CAPTEscaffold_324_18344.pdf]

Provisional ID	:	CAPTEscaffold_324_18344
Score total	:	197.9
Score for star read(s)	:	3.9
Score for read counts	:	191.8
Score for mfe	:	1.2
Score for randfold	:	1.6
Score for cons. seed	:	-0.6
Total read count	:	388
Mature read count	:	381
Loop read count	:	0
Star read count	:	7

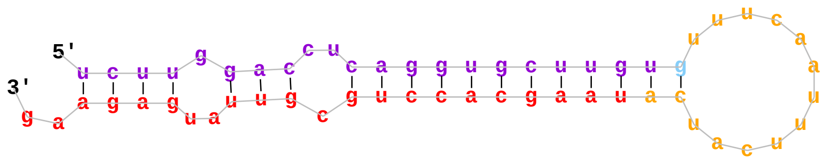

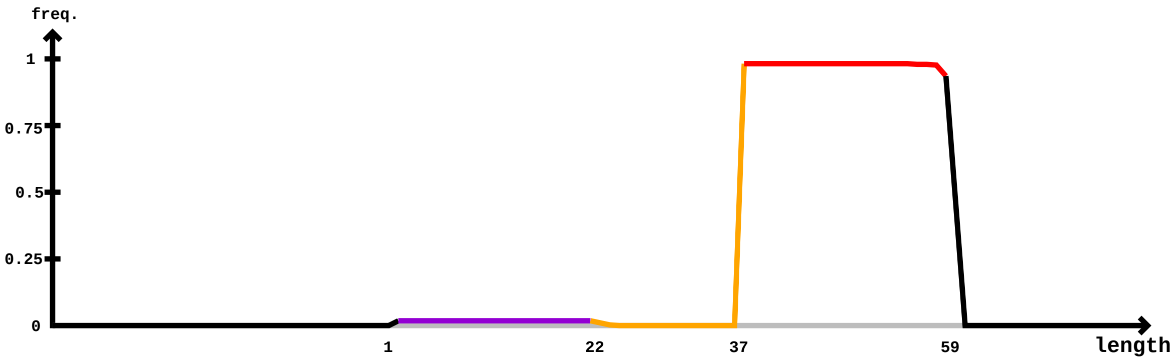


```
                                  Star                   Mature
5'- ugaccaagaauuguguugaguaugaauggcuuuuucuuggaccucaggugcuuguguuucaauuucaucauaagcaccugcguuaugagaaggagcugaagaacugucugcu   -3'   obs
    ugaccaagaauuguguugaguaugaauggcuuuuucuuggaccucaggugcuuguguuucaauuucaucauaagcaccugcguuaugagaaggagcugaagaacugucugcu         exp
    ..(((((........))).))......((((((((((.(((..(((((((((((...........))))))))))).)))..))))))))))....................   reads   mm   sample
    ..................................ucuuggaccucaggugcuugu.........................................................   3       0    seq
    ..................................ucuuggaccucaggugcuugC.........................................................   2       1    seq
    ..................................ucuuggaAcucaggugcuugu.........................................................   1       1    seq
    ..................................ucuuggaccucaggugcuugugG.......................................................   1       1    seq
    ......................................................................uaagcaccugcguuauga........................   1       0    seq
    ......................................................................uaagcaccugcguuaugaga......................   1       0    seq
    ......................................................................uaagcaccugcguuaugagaa.....................   16      0    seq
    ......................................................................uaaAcaccugcguuaugagaag....................   1       1    seq
    ......................................................................uaagcaccugcguuaugagaag....................   357     0    seq
    ......................................................................uaagcaccugAguuaugagaag....................   1       1    seq
    ......................................................................uaagcaccugcguuauAagaag....................   1       1    seq
    ......................................................................uaagcaccugcguuaugagaagU...................   1       1    seq
    ......................................................................uaagcaccugcguuaugagaagg...................   2       0    seq
```
