# Supplementary material for: The Small RNA Universe of Capitella teleta
Source: Front Mol Biosci. 2022 Feb 25;9:802814. doi: 10.3389/fmolb.2022.802814 (PMC8915122; doi:10.3389/fmolb.2022.802814)
Supplement: Supplementary file 1 [file DataSheet1.ZIP › Supplement/confident/CAPTEscaffold_1286_33815.pdf]

Provisional ID	:	CAPTEscaffold_1286_33815
Score total	:	344.3
Score for star read(s)	:	3.9
Score for read counts	:	338.1
Score for mfe	:	1.2
Score for randfold	:	1.6
Score for cons. seed	:	-0.6
Total read count	:	675
Mature read count	:	563
Loop read count	:	0
Star read count	:	112

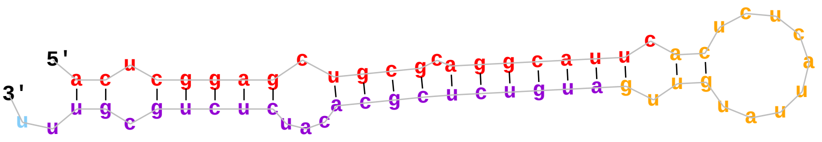

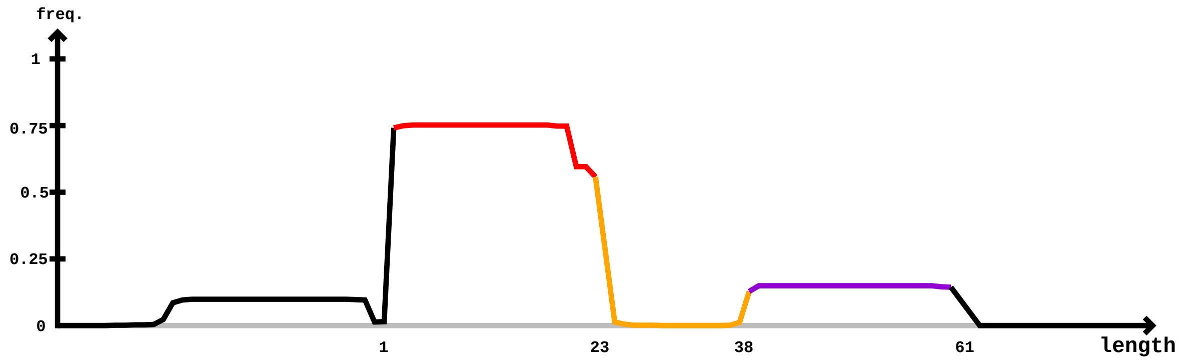

Mature Star

```
5'- uuuuuuuuccccuggaucuguagauugagccauacucggagcugcgcaggcauucacucucauuauguugaugucucgcacaucucugcguuugguuuguuuuacuaacaac   -3'    obs
    uuuuuuuuccccuggaucuguagauugagccauacucggagcugcgcaggcauucacucucauuauguugaugucucgcacaucucugcguuugguuuguuuuacuaacaac          exp
    .......(((...)))...(((((.(((((((.((.(((((.((((.(((((((.((.........)).)))))))))))...))))).)).))))))).))))).......    reads     mm      sample
    ....uuuuccccuggaucuguagauugag...................................................................................    1         0       seq
    ......uuccccuggaucuguagauugagc..................................................................................    1         0       seq
    ........ccccuggaucuguagauugagcca................................................................................    1         0       seq
    .........cccuggaucuguagauugagcc.................................................................................    14        0       seq
    ..........ccuggaucuguagauugagcc.................................................................................    47        0       seq
    ...........cuggaucuguagauuAagcc.................................................................................    1         1       seq
    ...........cuggaucuguagauugagcc.................................................................................    7         0       seq
    ............uggaucuguagauugagcc.................................................................................    2         0       seq
    ...............................auacucggagcugcgcagA..............................................................    3         1       seq
    ...............................auacucggagcugcgcaggca............................................................    6         0       seq
    ................................uacucggagcugcgcagAcauu..........................................................    2         1       seq
    .................................Gcucggagcugcgcaggca............................................................    1         1       seq
    .................................acucggagcugcgcaggcG............................................................    1         1       seq
    .................................acucggagcugcgcaggca............................................................    105       0       seq
    .................................acuUggagcugcgcaggca............................................................    1         1       seq
    .................................acucggagcugcgcagAcauu..........................................................    2         1       seq
    .................................acucggagcugcgcaggcauu..........................................................    24        0       seq
    .................................acucggagcugcgcaCgcauuc.........................................................    1         1       seq
    .................................acucggagcugcgcagCcauuc.........................................................    2         1       seq
    .................................acAcggagcugcgcaggcauuc.........................................................    1         1       seq
    .................................acuUggagcugcgcaggcauuc.........................................................    1         1       seq
    .................................acucggagcugcgcaggcauuU.........................................................    76        1       seq
    .................................acucggagcugcgcagAcauuc.........................................................    1         1       seq
    .................................acucggagcAgcgcaggcauuc.........................................................    1         1       seq
    .................................acucggagcugcgcaggcauuc.........................................................    271       0       seq
    .................................Gcucggagcugcgcaggcauuc.........................................................    6         1       seq
    .................................acucggagcugcgcaggcauucU........................................................    1         1       seq
    .................................acucggagcugcgcaggcauuca........................................................    41        0       seq
    .................................acucggagcugcgcagAcauuca........................................................    2         1       seq
    .................................acucggagcugcgcaggcauucac.......................................................    3         0       seq
    .................................acucggagcugcgcaggcauucaA.......................................................    1         1       seq
    .................................acucggagcugcgcaggcauucaAu......................................................    1         1       seq
    .................................acucggagcugcgcaggcauucacA......................................................    1         1       seq
```

```
                                 Mature                                Star
uuuuuuuuccccuggaucuguagauugagccauacucggagcugcgcaggcauucacucucauuauguugaugucucgcacaucucugcguuugguuuguuuuacuaacaac
.................................acucggagcugcgcaggcauucacucuU...................................................   1   1   seq
..................................cucggagcugcgcaggcauu..........................................................   1   0   seq
..................................cucggagcugcgcaggcauuc.........................................................   4   0   seq
..................................cucggagcugcgcaggcauucac.......................................................   1   0   seq
...................................ucggagcugcgcaggcauucac.......................................................   1   0   seq
...................................ucggagcugcgcaggcauucacu......................................................   1   0   seq
....................................................................ugaugucucgcacaCcucugcguuu...................   1   1   seq
.....................................................................gaugucucgcacaCcucugcguu....................   2   1   seq
.....................................................................gaugucucgcacaucucugcguu....................   2   0   seq
.....................................................................gaugucucgcacaCcucugcguuu...................   3   1   seq
.....................................................................gaugAcucgcacaucucugcguuu...................   1   1   seq
......................................................................augucucgcacaucucugcg......................   3   0   seq
......................................................................augucucgcacaucucugcgu.....................   1   0   seq
......................................................................augucucgcacaucucugAguu....................   1   1   seq
......................................................................augucucgcacaucucugcguu....................   76  0   seq
......................................................................Gugucucgcacaucucugcguu....................   1   1   seq
......................................................................augucucgcGcaucucugcguu....................   2   1   seq
......................................................................augucucgcacaucucugcguuu...................   3   0   seq
.......................................................................ugucucgcacaucucugcguu....................   12  0   seq
.......................................................................ugucucgcacaCcucugcguuu...................   1   1   seq
.......................................................................ugucucgcacaucucugcguuu...................   3   0   seq
```
