# Supplementary figures and images for: The Small RNA Universe of Capitella teleta
Source: Front Mol Biosci. 2022 Feb 25;9:802814. doi: 10.3389/fmolb.2022.802814 (PMC8915122; doi:10.3389/fmolb.2022.802814)

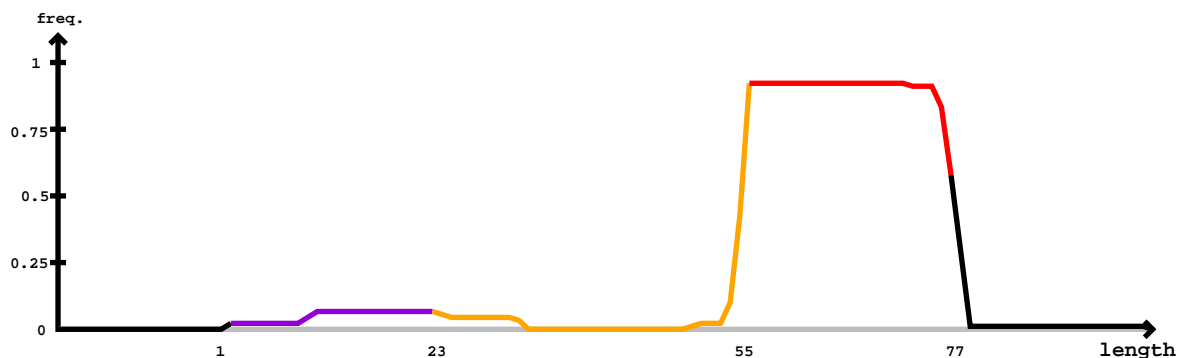

## Mature

[illegible]

Supplement: Supplementary file 1 [file DataSheet1.ZIP › Supplement/candidate/CAPTEscaffold_24_2918.pdf]

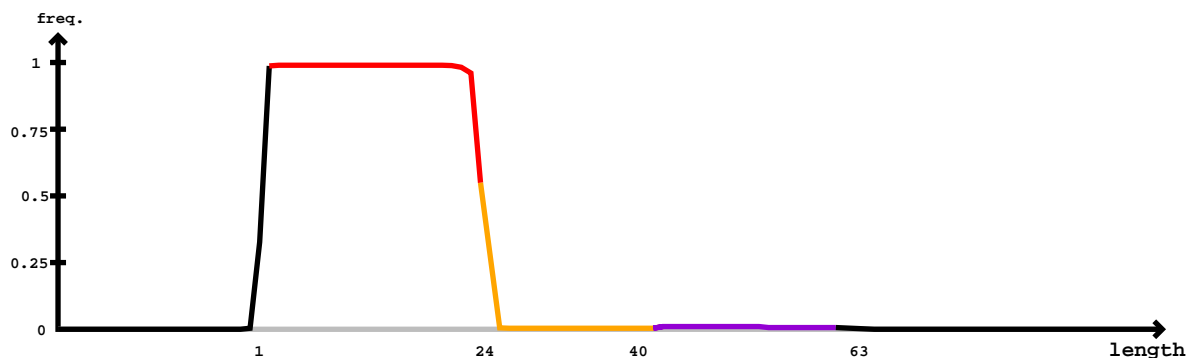

Star

[illegible]

Supplement: Supplementary file 1 [file DataSheet1.ZIP › Supplement/candidate/CAPTEscaffold_228_13639.pdf]

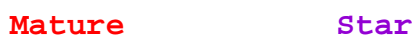[illegible]

Supplement: Supplementary file 1 [file DataSheet1.ZIP › Supplement/candidate/CAPTEscaffold_22815_46346.pdf]

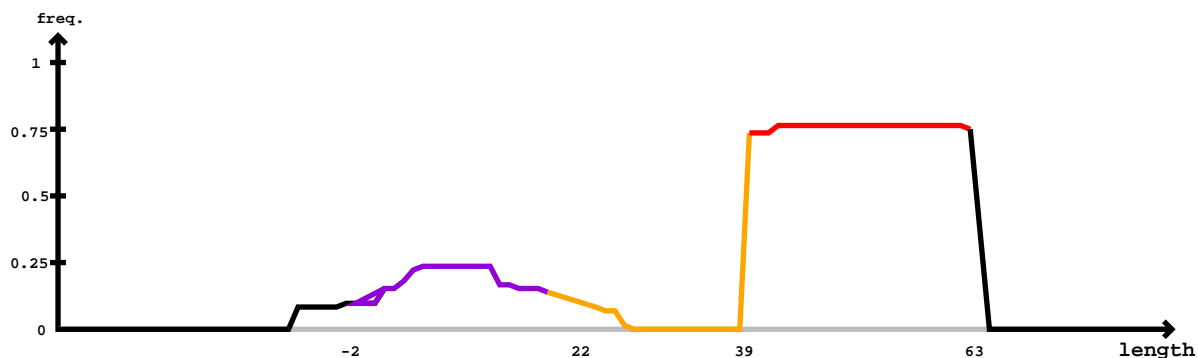

**Mature**

[illegible]

Supplement: Supplementary file 1 [file DataSheet1.ZIP › Supplement/candidate/CAPTEscaffold_302_17352.pdf]

[illegible]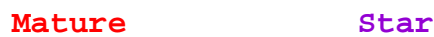[illegible]

Supplement: Supplementary file 1 [file DataSheet1.ZIP › Supplement/candidate/CAPTEscaffold_655_26868.pdf]

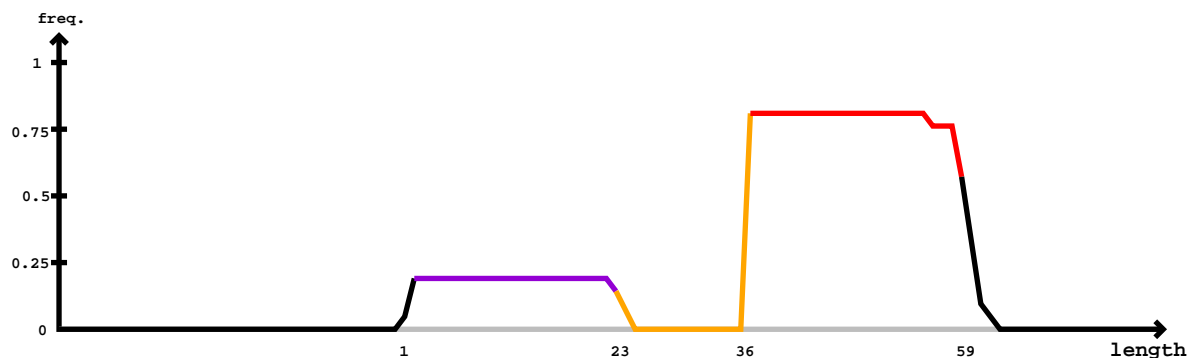

## Mature

[illegible]

Supplement: Supplementary file 1 [file DataSheet1.ZIP › Supplement/candidate/CAPTEscaffold_130_11176.pdf]

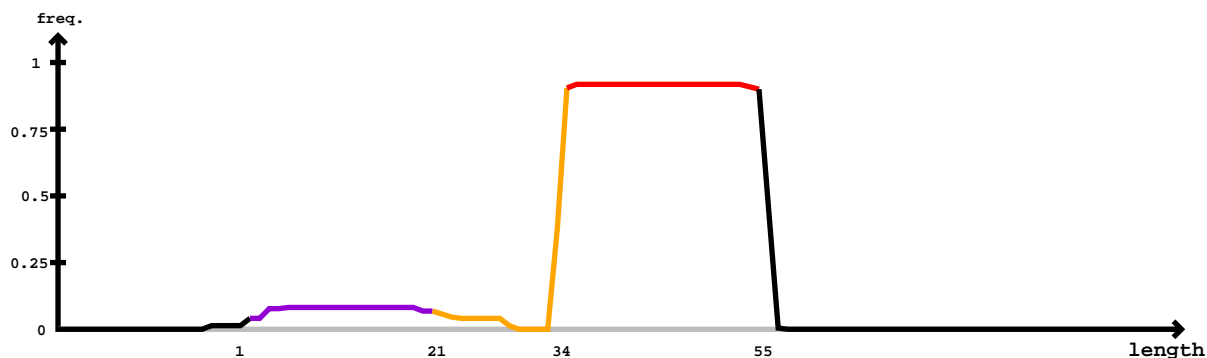

## Mature

[illegible]

Supplement: Supplementary file 1 [file DataSheet1.ZIP › Supplement/candidate/CAPTEscaffold_60_5424.pdf]

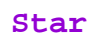[illegible]

Supplement: Supplementary file 1 [file DataSheet1.ZIP › Supplement/candidate/CAPTEscaffold_51_5558.pdf]

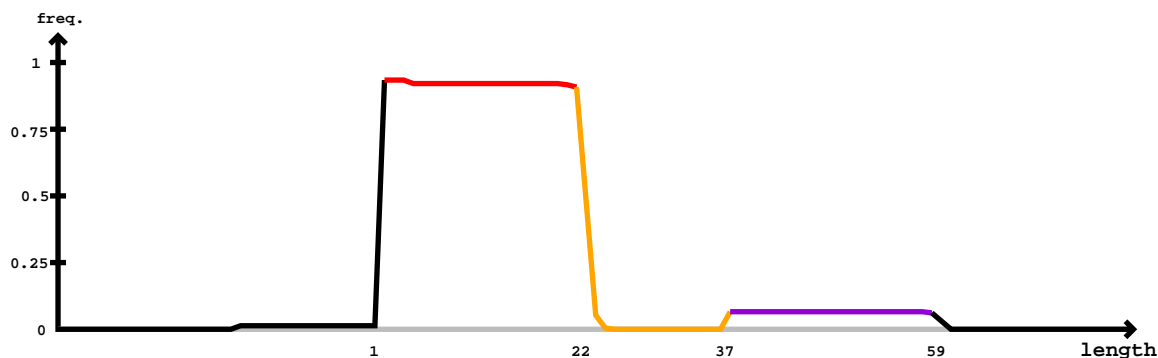

Star

[illegible]

Supplement: Supplementary file 1 [file DataSheet1.ZIP › Supplement/candidate/CAPTEscaffold_324_18360.pdf]

[illegible]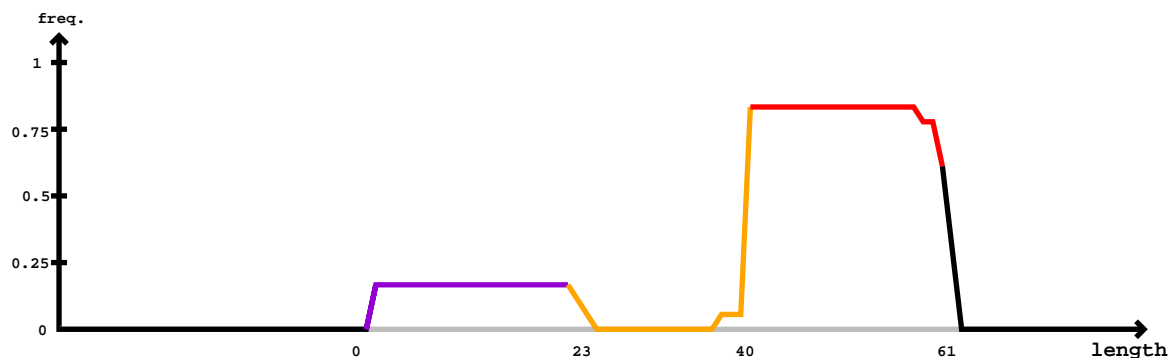

Mature

[illegible]

Supplement: Supplementary file 1 [file DataSheet1.ZIP › Supplement/candidate/CAPTEscaffold_2741_37338.pdf]

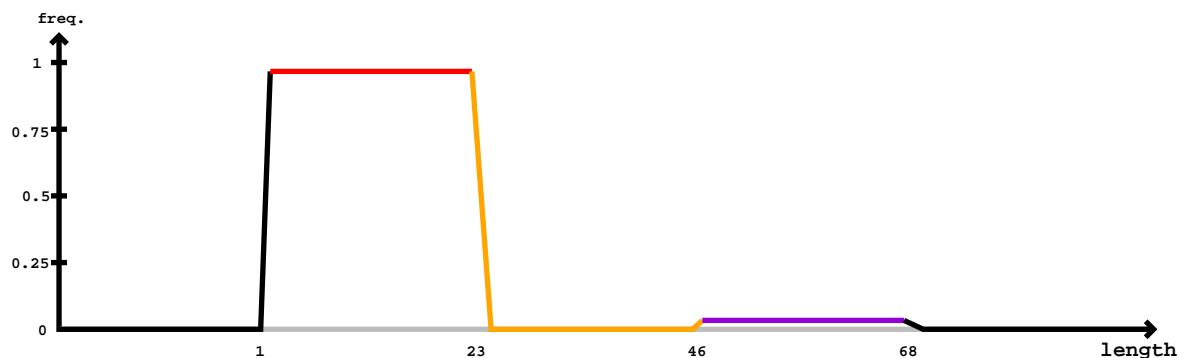

Star

[illegible]

Supplement: Supplementary file 1 [file DataSheet1.ZIP › Supplement/candidate/CAPTEscaffold_759_23463.pdf]

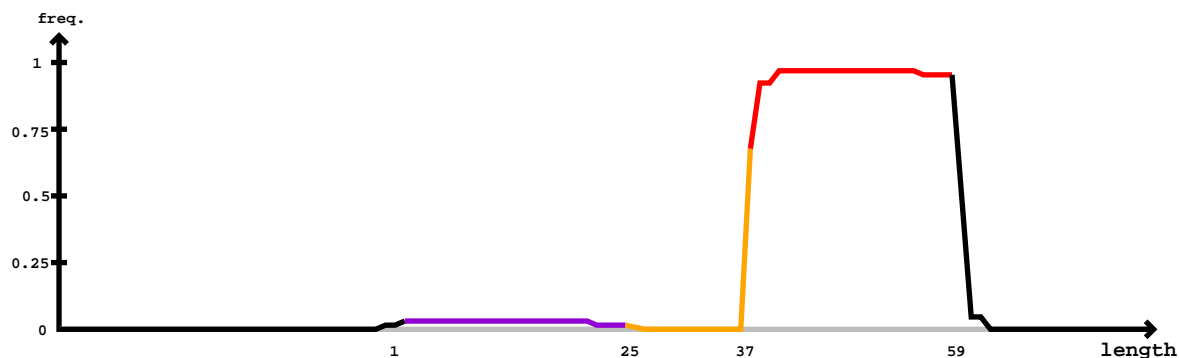

**Mature**

[illegible]

Supplement: Supplementary file 1 [file DataSheet1.ZIP › Supplement/candidate/CAPTEscaffold_123_10154.pdf]

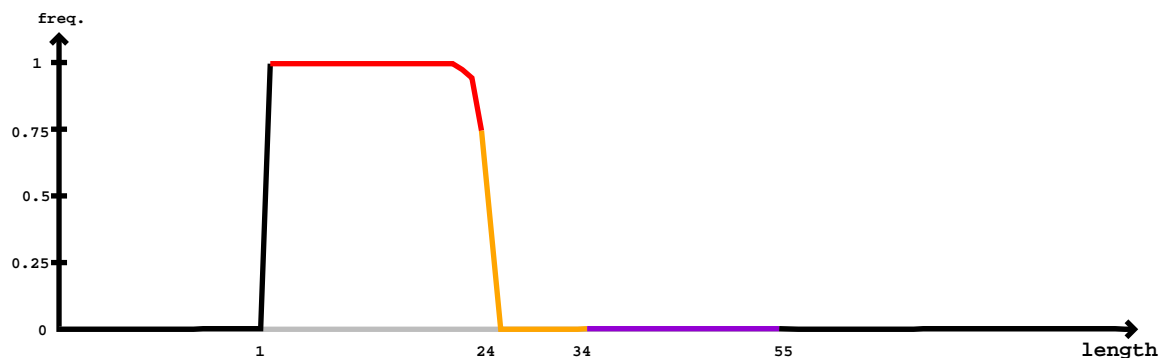

Star

[illegible]

Supplement: Supplementary file 1 [file DataSheet1.ZIP › Supplement/candidate/CAPTEscaffold_251_15906.pdf]

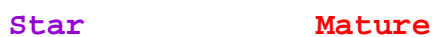[illegible]

Supplement: Supplementary file 1 [file DataSheet1.ZIP › Supplement/candidate/CAPTEscaffold_60_5445.pdf]

[illegible]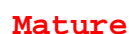[illegible]

Supplement: Supplementary file 1 [file DataSheet1.ZIP › Supplement/candidate/CAPTEscaffold_154_12069.pdf]

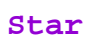[illegible]

Supplement: Supplementary file 1 [file DataSheet1.ZIP › Supplement/candidate/CAPTEscaffold_69_6453.pdf]

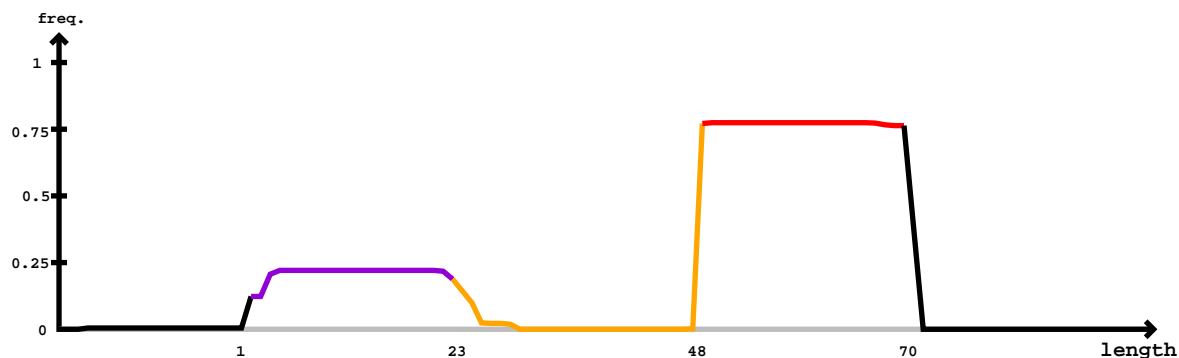

## Mature

[illegible]

Supplement: Supplementary file 1 [file DataSheet1.ZIP › Supplement/candidate/CAPTEscaffold_488_22736.pdf]

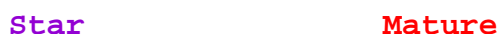[illegible]

Supplement: Supplementary file 1 [file DataSheet1.ZIP › Supplement/candidate/CAPTEscaffold_15480_45261.pdf]

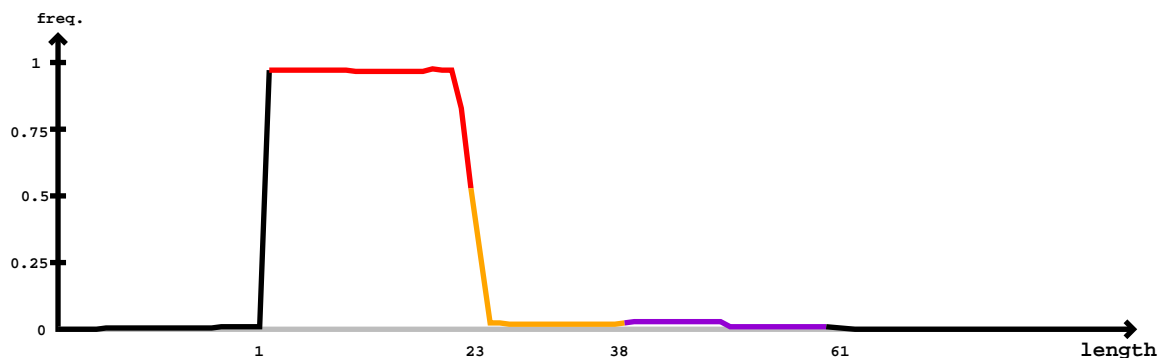

Star

[illegible]

Supplement: Supplementary file 1 [file DataSheet1.ZIP › Supplement/candidate/CAPTEscaffold_757_28366.pdf]

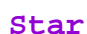[illegible]

Supplement: Supplementary file 1 [file DataSheet1.ZIP › Supplement/candidate/CAPTEscaffold_224_14422.pdf]

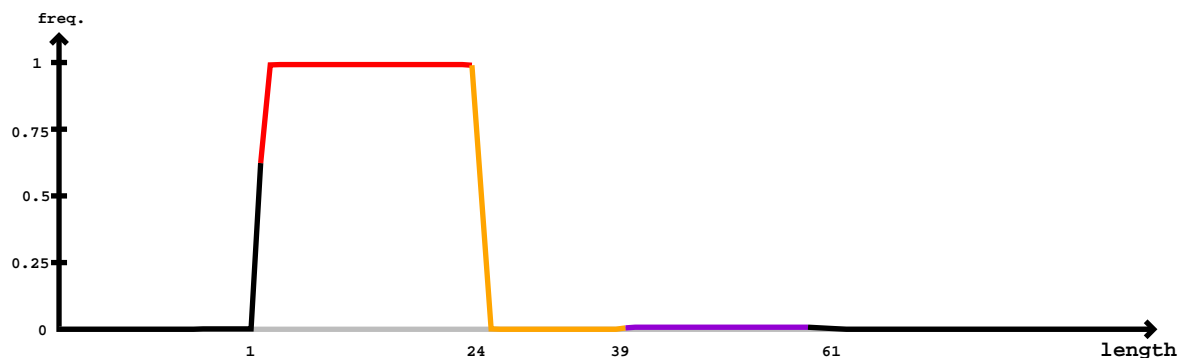

Star

[illegible]

Supplement: Supplementary file 1 [file DataSheet1.ZIP › Supplement/candidate/CAPTEscaffold_69_6469.pdf]

[illegible]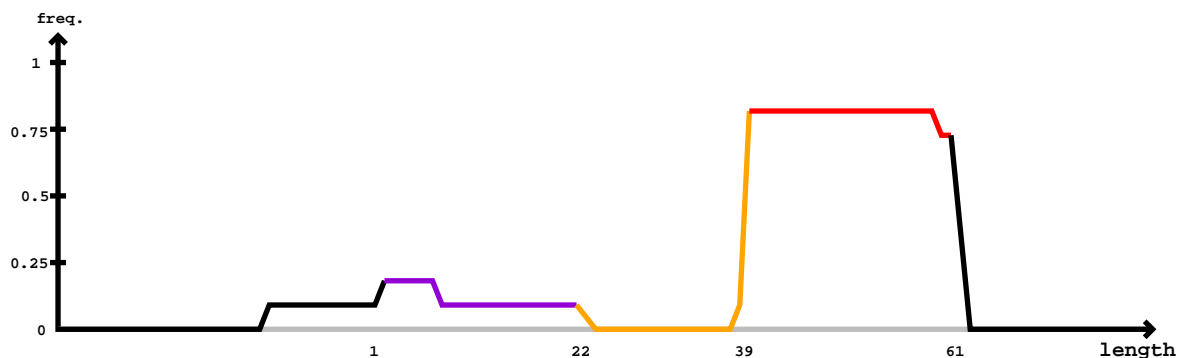

**Mature**

[illegible]

Supplement: Supplementary file 1 [file DataSheet1.ZIP › Supplement/candidate/CAPTEscaffold_65_5264.pdf]

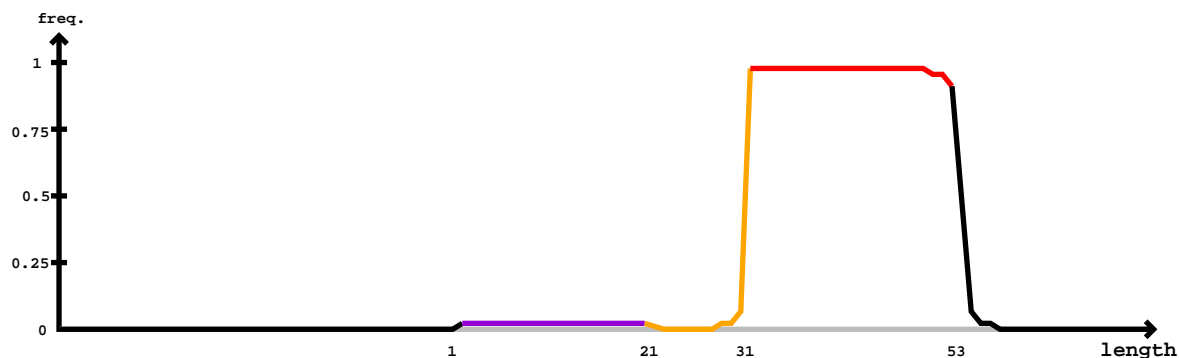

**Star**

[illegible]

Supplement: Supplementary file 1 [file DataSheet1.ZIP › Supplement/candidate/CAPTEscaffold_25974_46680.pdf]

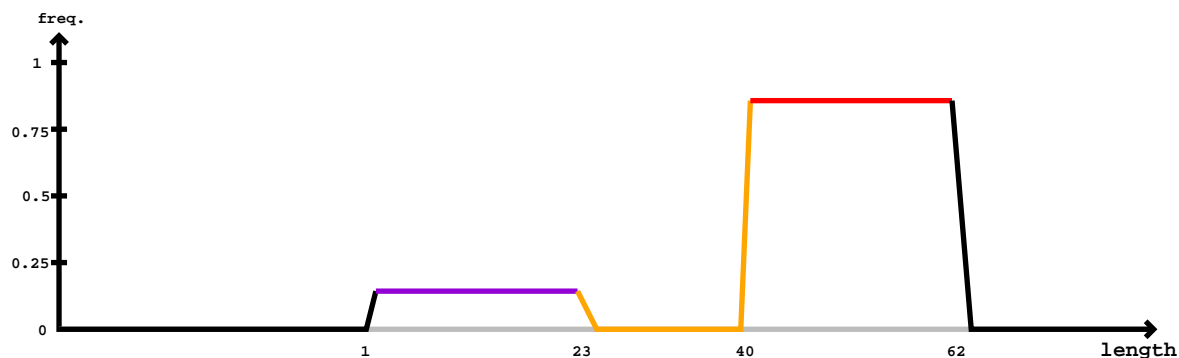

Mature

[illegible]

Supplement: Supplementary file 1 [file DataSheet1.ZIP › Supplement/candidate/CAPTEscaffold_988_28522.pdf]

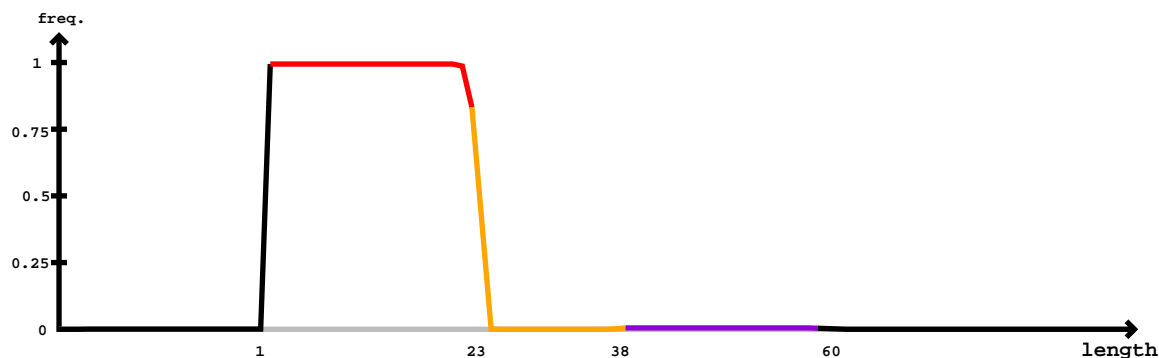

Star

[illegible]

Supplement: Supplementary file 1 [file DataSheet1.ZIP › Supplement/candidate/CAPTEscaffold_234_15398.pdf]

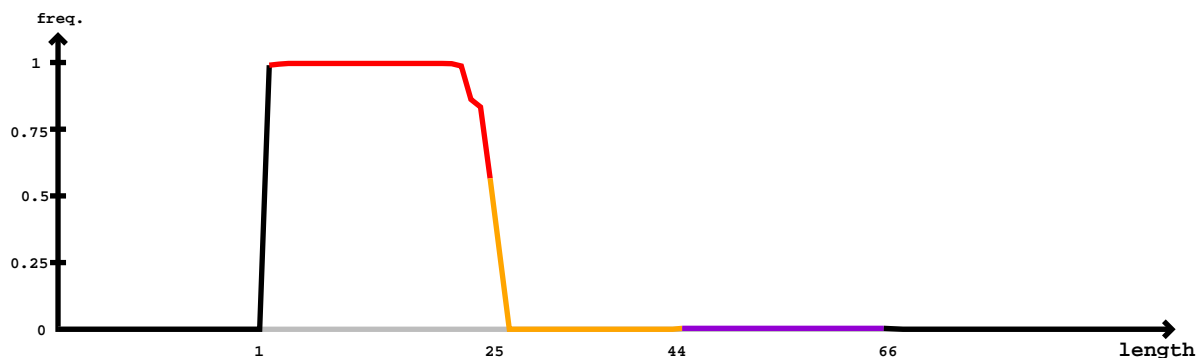

Star

[illegible]

Supplement: Supplementary file 1 [file DataSheet1.ZIP › Supplement/candidate/CAPTEscaffold_1755_32145.pdf]

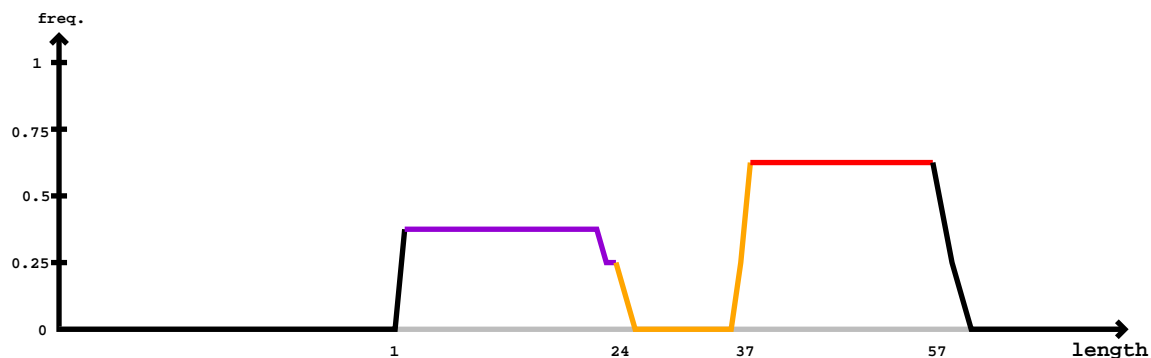

Mature

[illegible]

Supplement: Supplementary file 1 [file DataSheet1.ZIP › Supplement/candidate/CAPTEscaffold_222_14831.pdf]

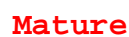[illegible]

Supplement: Supplementary file 1 [file DataSheet1.ZIP › Supplement/candidate/CAPTEscaffold_78_6772.pdf]

[illegible]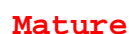[illegible]

Supplement: Supplementary file 1 [file DataSheet1.ZIP › Supplement/candidate/CAPTEscaffold_329_17006.pdf]

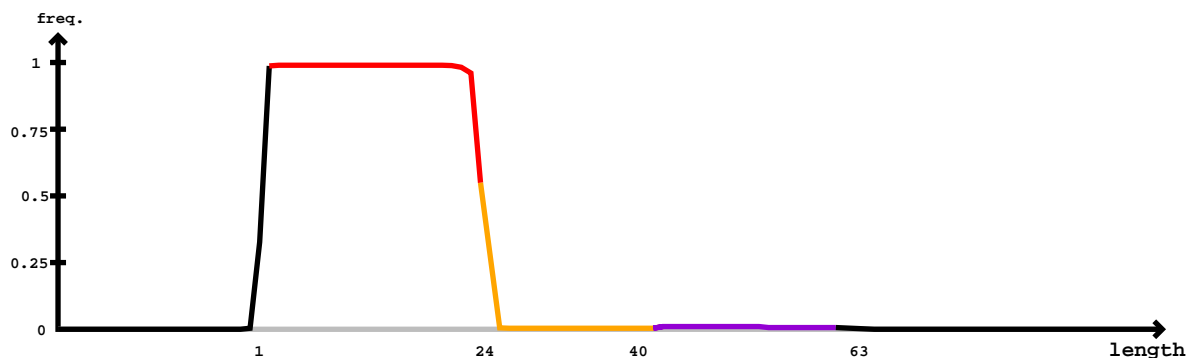

Star

[illegible]

Supplement: Supplementary file 1 [file DataSheet1.ZIP › Supplement/candidate/CAPTEscaffold_71_6097.pdf]

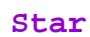[illegible]

Supplement: Supplementary file 1 [file DataSheet1.ZIP › Supplement/candidate/CAPTEscaffold_493_23921.pdf]

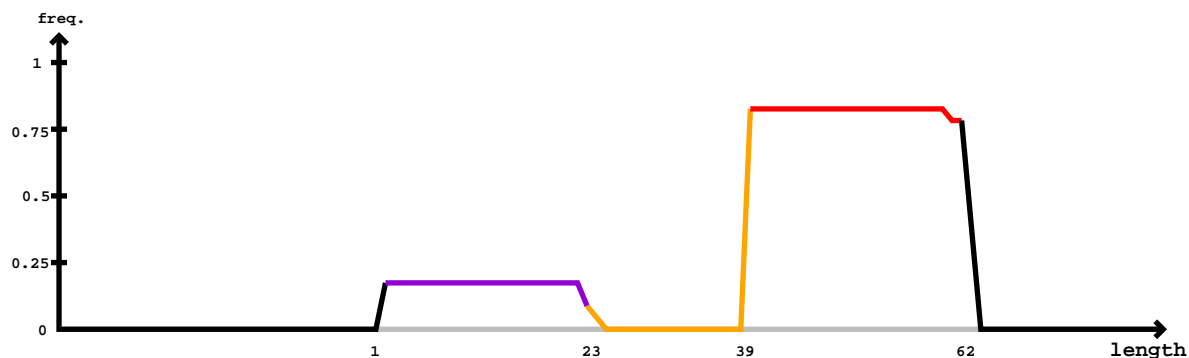

**Mature**

[illegible]

Supplement: Supplementary file 1 [file DataSheet1.ZIP › Supplement/candidate/CAPTEscaffold_344_18510.pdf]

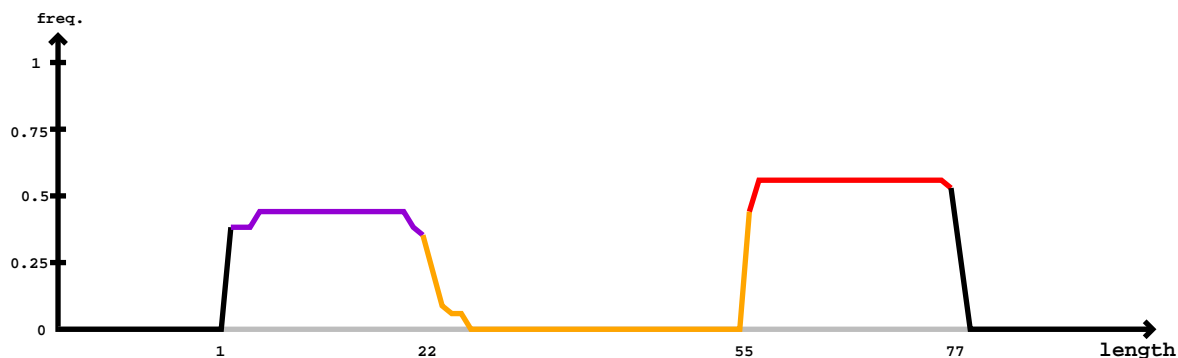

**Mature**

[illegible]

Supplement: Supplementary file 1 [file DataSheet1.ZIP › Supplement/candidate/CAPTEscaffold_276_16091.pdf]

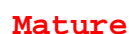[illegible]

Supplement: Supplementary file 1 [file DataSheet1.ZIP › Supplement/candidate/CAPTEscaffold_361_15273.pdf]

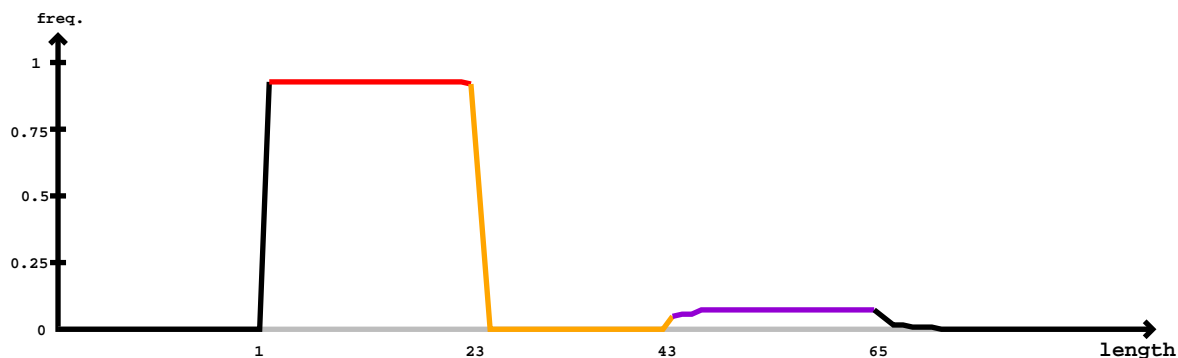

Star

[illegible]

Supplement: Supplementary file 1 [file DataSheet1.ZIP › Supplement/candidate/CAPTEscaffold_146_11393.pdf]

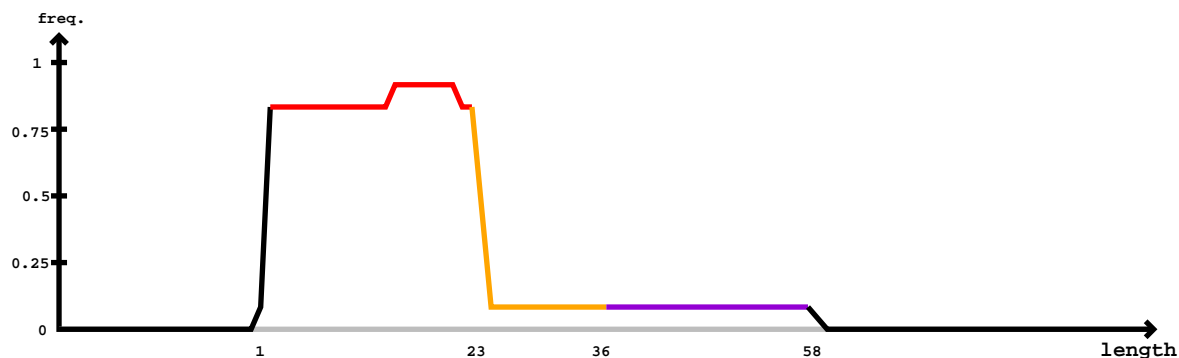

Star

[illegible]

Supplement: Supplementary file 1 [file DataSheet1.ZIP › Supplement/candidate/CAPTEscaffold_284_15974.pdf]

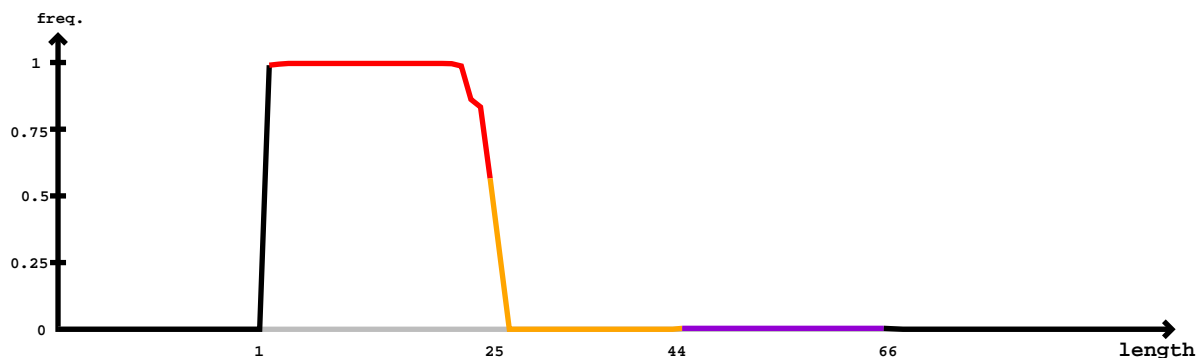

Star

[illegible]

Supplement: Supplementary file 1 [file DataSheet1.ZIP › Supplement/candidate/CAPTEscaffold_9804_43110.pdf]

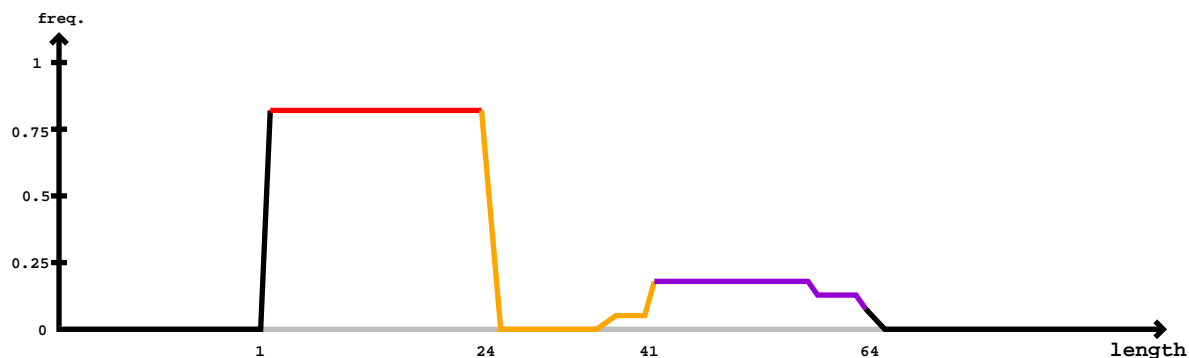

Star

[illegible]

Supplement: Supplementary file 1 [file DataSheet1.ZIP › Supplement/candidate/CAPTEscaffold_475_21078.pdf]

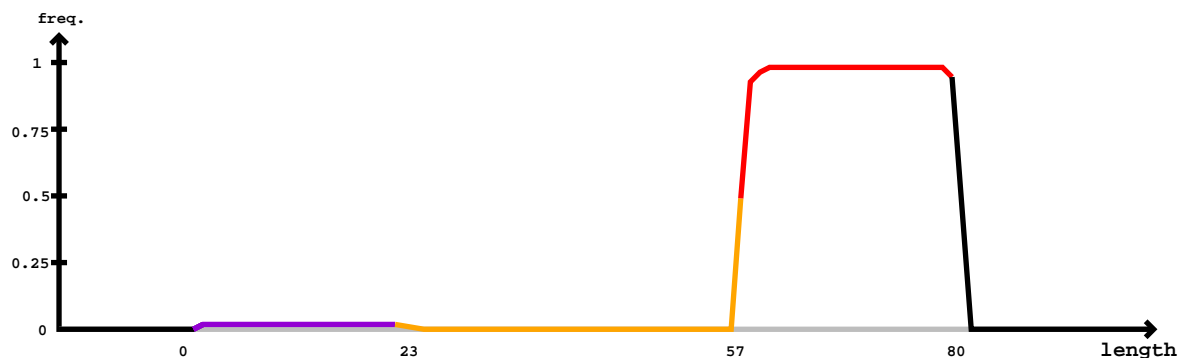

## Mature

Supplement: Supplementary file 1 [file DataSheet1.ZIP › Supplement/candidate/CAPTEscaffold_755_28348.pdf]

5' a u c a a g u g c c u c g a g c u c c u  
3' u a g u u c a c g g a g c u c g a g g a

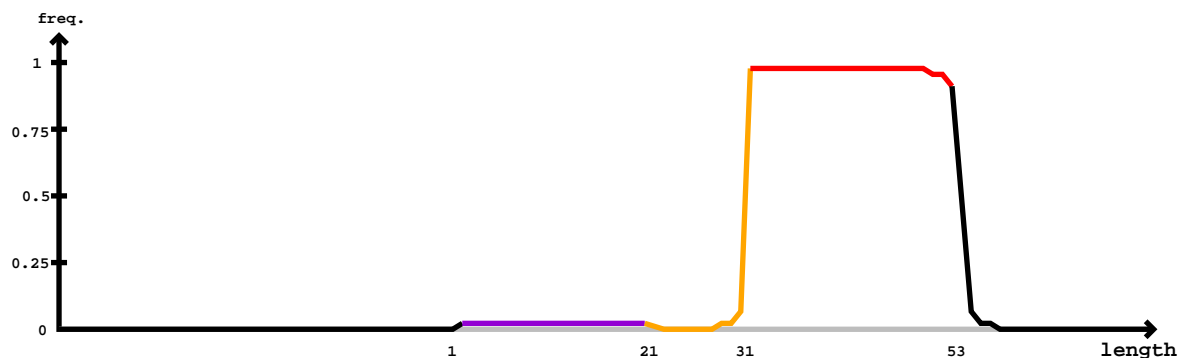[illegible]

Supplement: Supplementary file 1 [file DataSheet1.ZIP › Supplement/candidate/CAPTEscaffold_519_23023.pdf]

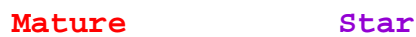[illegible]

Supplement: Supplementary file 1 [file DataSheet1.ZIP › Supplement/candidate/CAPTEscaffold_44_4619.pdf]

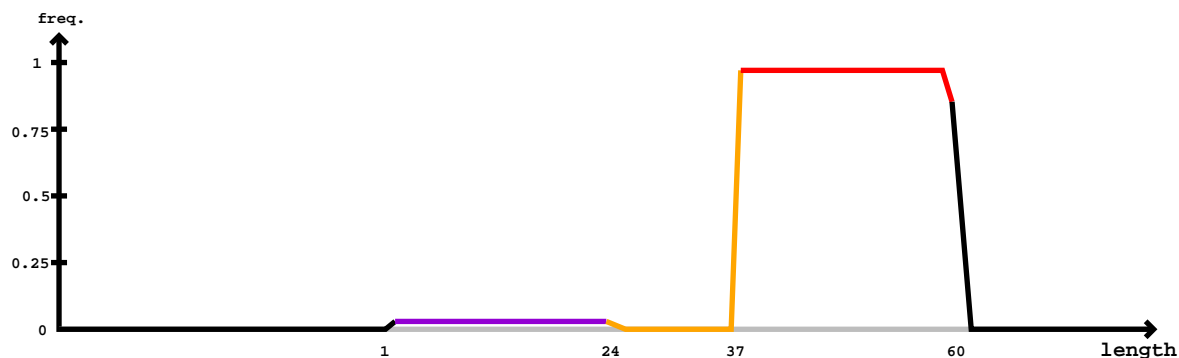

## Mature

[illegible]

Supplement: Supplementary file 1 [file DataSheet1.ZIP › Supplement/candidate/CAPTEscaffold_137_8834.pdf]

[illegible]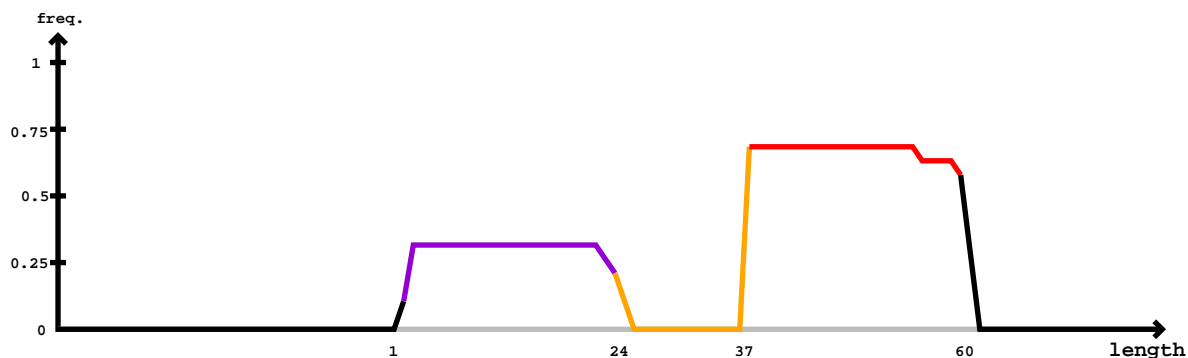

**Mature**

[illegible]

Supplement: Supplementary file 1 [file DataSheet1.ZIP › Supplement/candidate/CAPTEscaffold_488_22749.pdf]

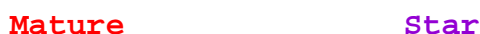[illegible]

Supplement: Supplementary file 1 [file DataSheet1.ZIP › Supplement/candidate/CAPTEscaffold_8946_42599.pdf]

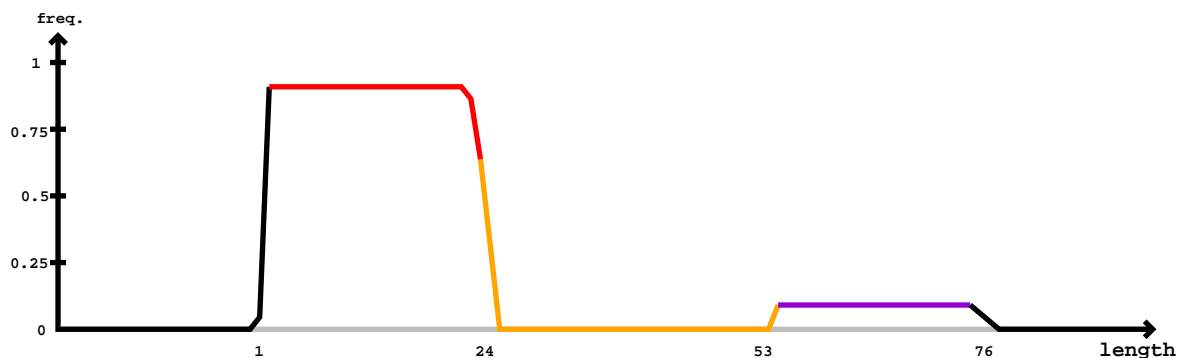

Star

[illegible]

Supplement: Supplementary file 1 [file DataSheet1.ZIP › Supplement/candidate/CAPTEscaffold_1214_32128.pdf]

[illegible]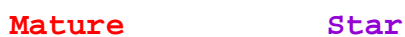[illegible]

Supplement: Supplementary file 1 [file DataSheet1.ZIP › Supplement/candidate/CAPTEscaffold_329_16948.pdf]

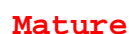[illegible]

Supplement: Supplementary file 1 [file DataSheet1.ZIP › Supplement/candidate/CAPTEscaffold_815_29074.pdf]

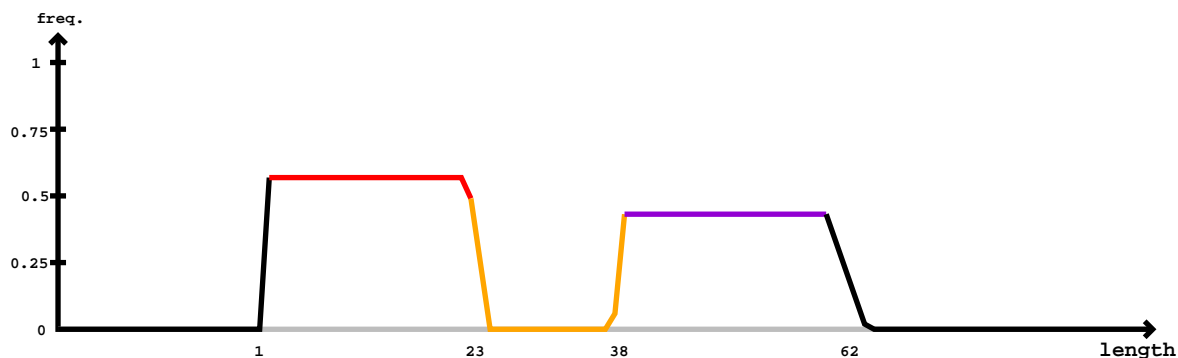

Star

[illegible]

Supplement: Supplementary file 1 [file DataSheet1.ZIP › Supplement/candidate/CAPTEscaffold_2218_35830.pdf]

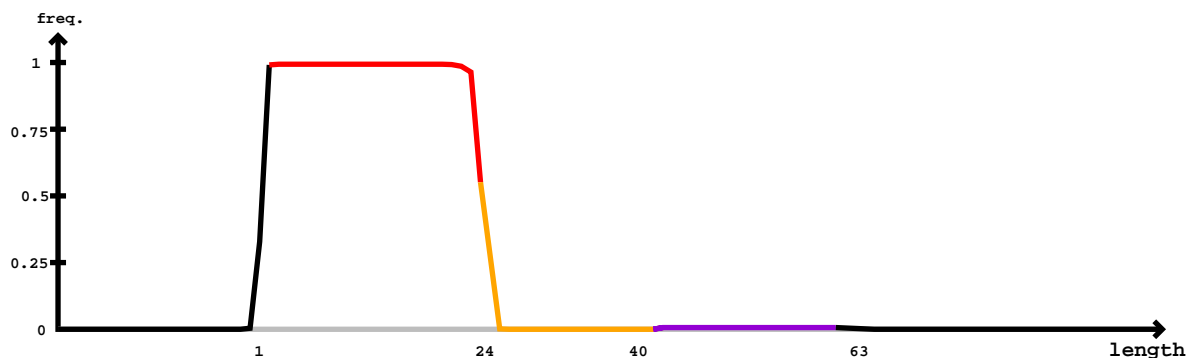

Star

[illegible]

Supplement: Supplementary file 1 [file DataSheet1.ZIP › Supplement/candidate/CAPTEscaffold_27263_46801.pdf]

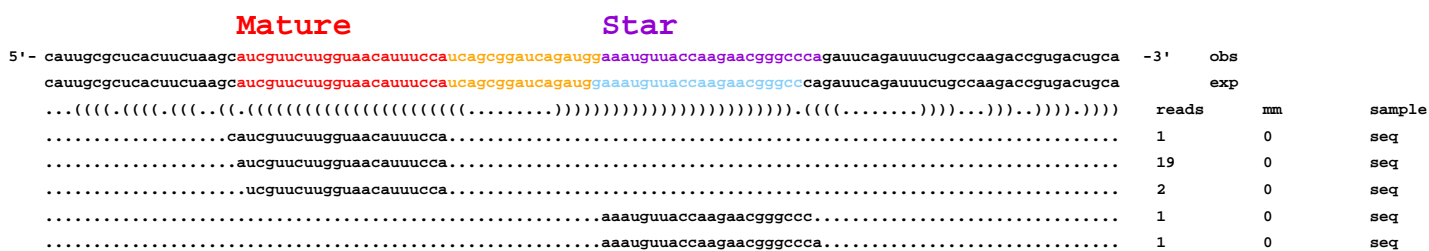

Supplement: Supplementary file 1 [file DataSheet1.ZIP › Supplement/candidate/CAPTEscaffold_3352_39088.pdf]

[illegible]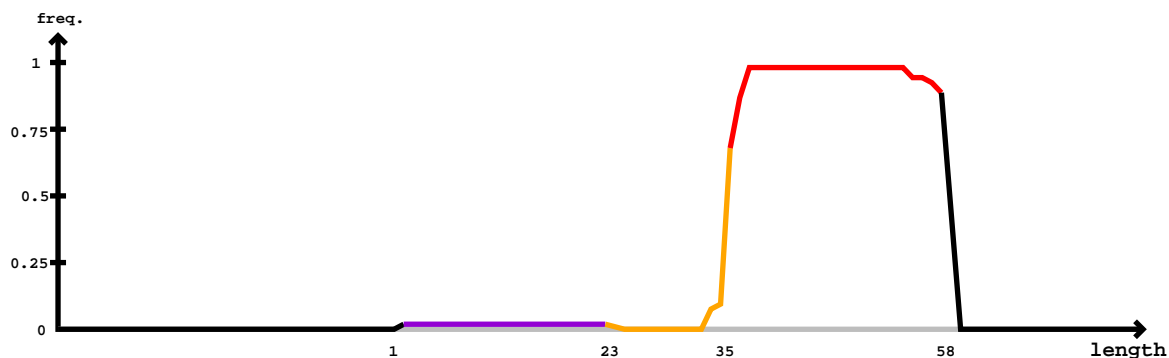

## Mature

[illegible]

Supplement: Supplementary file 1 [file DataSheet1.ZIP › Supplement/candidate/CAPTEscaffold_1153_32192.pdf]

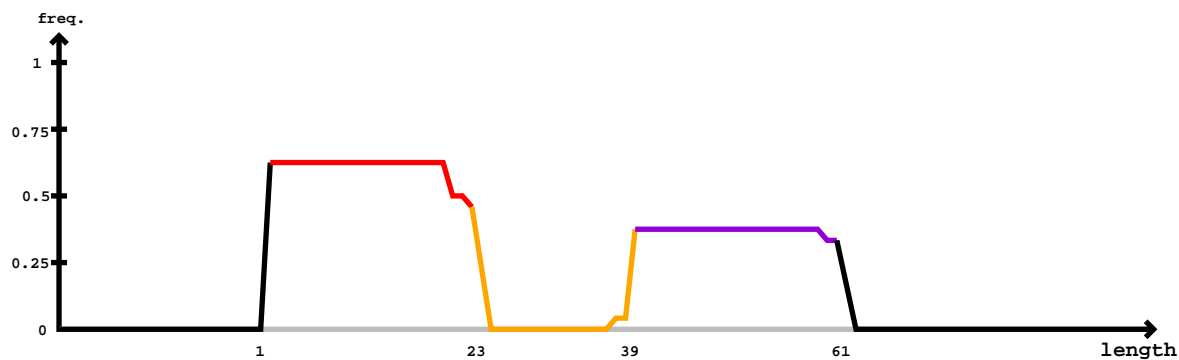

Star

[illegible]

Supplement: Supplementary file 1 [file DataSheet1.ZIP › Supplement/candidate/CAPTEscaffold_107_7101.pdf]

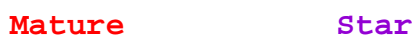[illegible]

Supplement: Supplementary file 1 [file DataSheet1.ZIP › Supplement/candidate/CAPTEscaffold_409_18892.pdf]

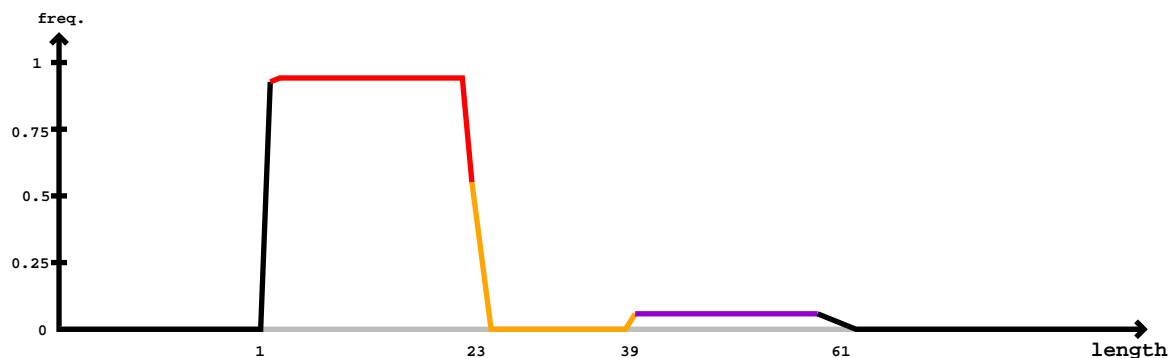

Star

[illegible]

Supplement: Supplementary file 1 [file DataSheet1.ZIP › Supplement/homologRecovered/CAPTEscaffold_192_13660.pdf]

[illegible]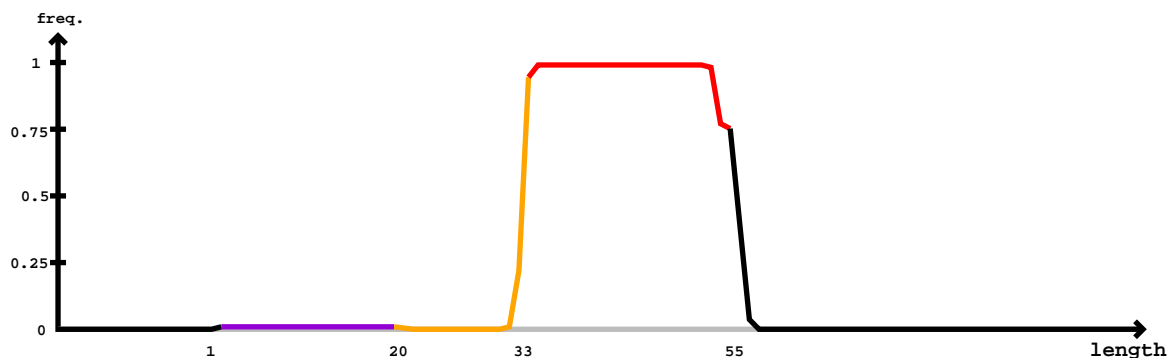

## Mature

[illegible]

Supplement: Supplementary file 1 [file DataSheet1.ZIP › Supplement/homologRecovered/CAPTEscaffold_508_21987.pdf]

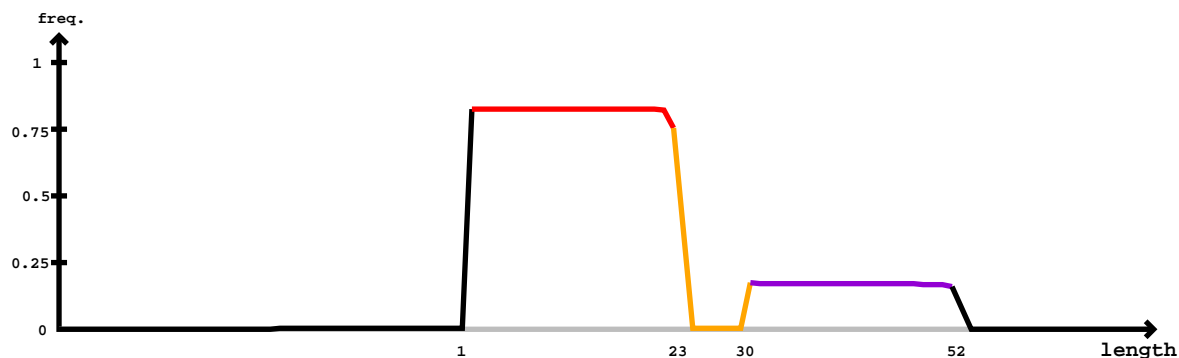[illegible]

Supplement: Supplementary file 1 [file DataSheet1.ZIP › Supplement/confident/CAPTEscaffold_324_18358.pdf]

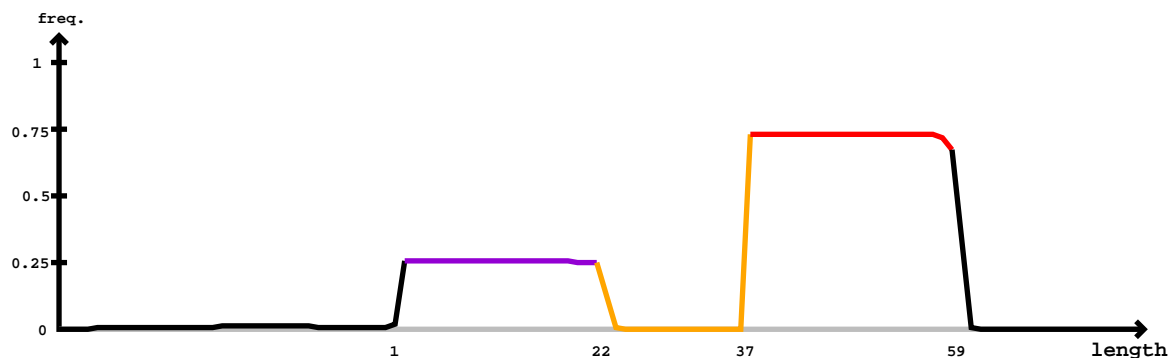

**Mature**

[illegible]

Supplement: Supplementary file 1 [file DataSheet1.ZIP › Supplement/confident/CAPTEscaffold_60_5447.pdf]

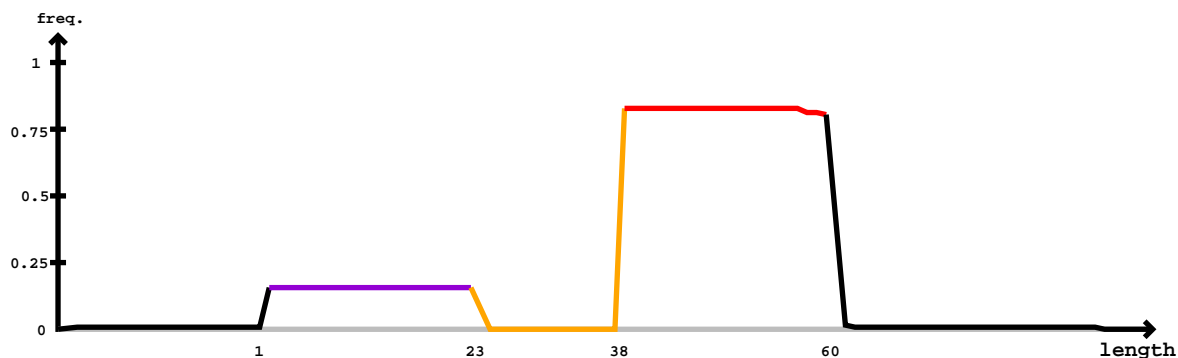

## Mature

[illegible]

Supplement: Supplementary file 1 [file DataSheet1.ZIP › Supplement/confident/CAPTEscaffold_488_22722.pdf]

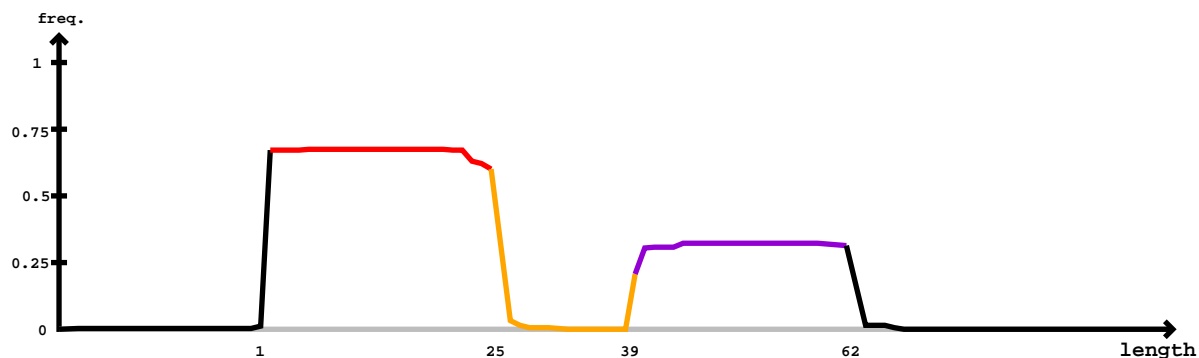

Star

[illegible]

Supplement: Supplementary file 1 [file DataSheet1.ZIP › Supplement/confident/CAPTEscaffold_154_12133.pdf]

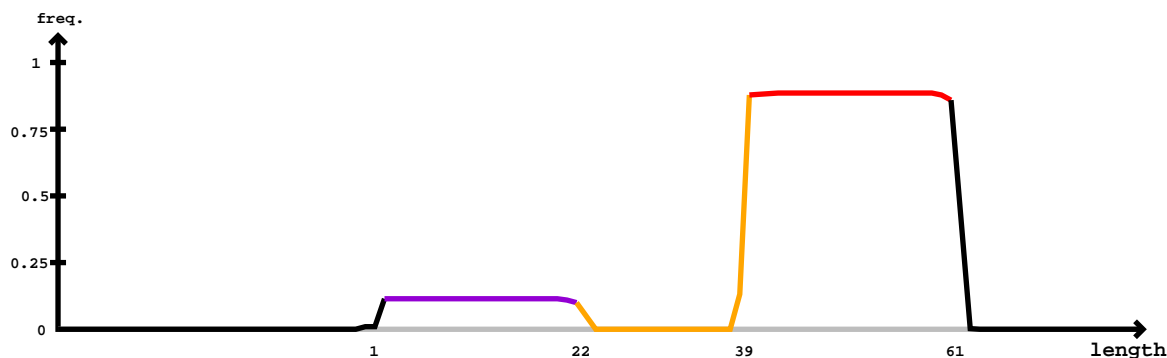

## Mature

[illegible]

Supplement: Supplementary file 1 [file DataSheet1.ZIP › Supplement/confident/CAPTEscaffold_234_15399.pdf]

[illegible]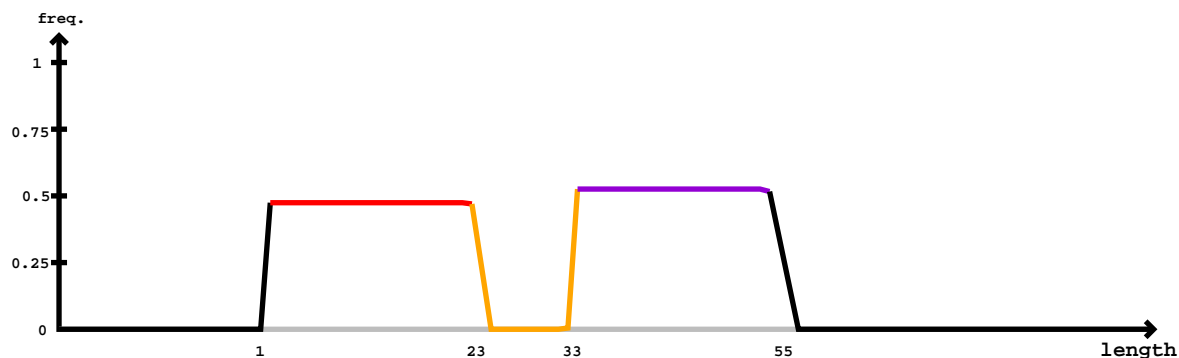

Star

[illegible]

Supplement: Supplementary file 1 [file DataSheet1.ZIP › Supplement/confident/CAPTEscaffold_12304_44298.pdf]

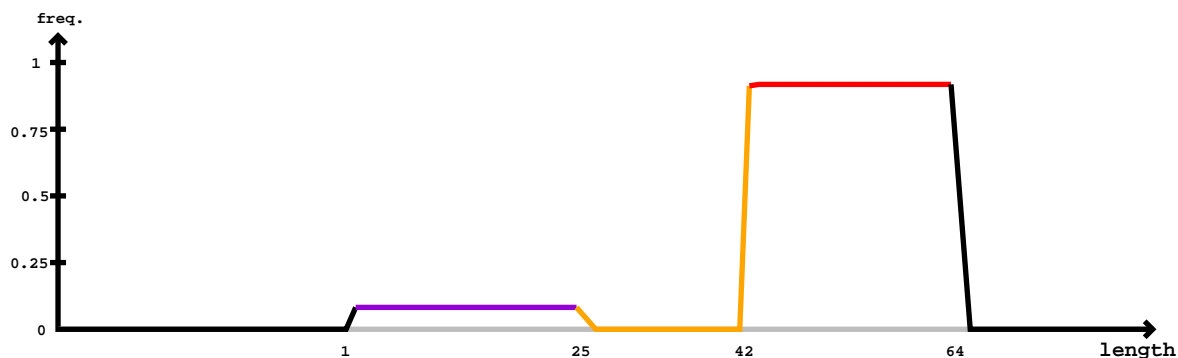

**Mature**

[illegible]

Supplement: Supplementary file 1 [file DataSheet1.ZIP › Supplement/confident/CAPTEscaffold_200_13770.pdf]

[illegible]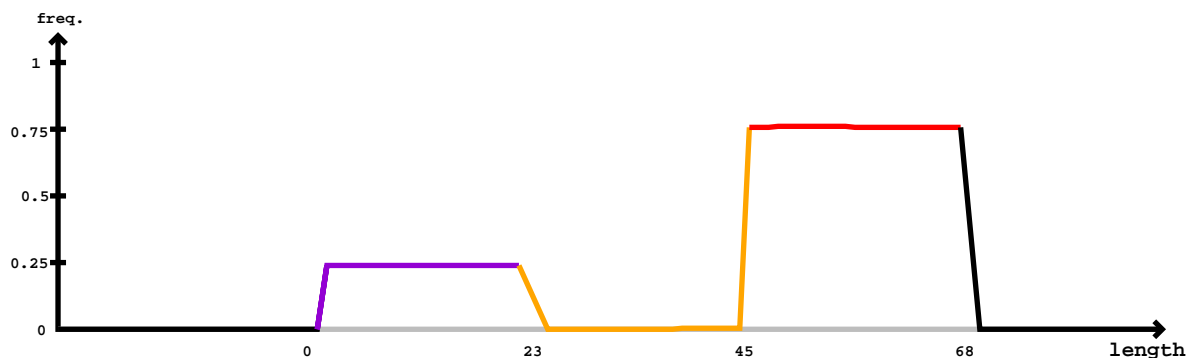

**Mature**

[illegible]

Supplement: Supplementary file 1 [file DataSheet1.ZIP › Supplement/confident/CAPTEscaffold_22_2009.pdf]

[illegible]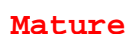[illegible]

Supplement: Supplementary file 1 [file DataSheet1.ZIP › Supplement/confident/CAPTEscaffold_324_18346.pdf]
